# Enantioselective Transfer Hydrogenation of α-Methoxyimino-β-keto Esters

**DOI:** 10.1021/acs.joc.4c00381

**Published:** 2024-08-30

**Authors:** Prabhakara R. Tharra, Jiří Švejkar, Abhijeet S. Jadhav, Marek Nečas, Pavel A. Dub, Mathew D. Halls, Jakub Švenda

**Affiliations:** † Department of Chemistry, Faculty of Science, Masaryk University, Kamenice 5, Brno 625 00, Czech Republic; ‡ International Clinical Research Center, St. Anne’s University Hospital, Pekařská 53, Brno 656 91, Czech Republic; § Schrödinger, Inc., San Diego, California 92121, United States

## Abstract

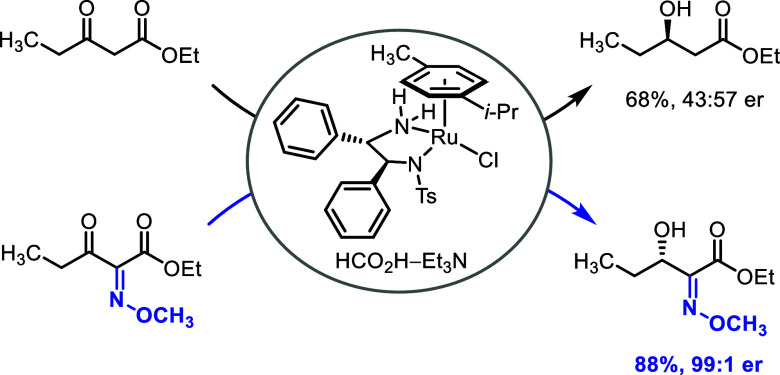

α-Methoxyimino-β-keto esters are reported to undergo highly enantioselective catalytic transfer hydrogenation using the Noyori–Ikariya complex RuCl(*p*-cymene)[(*S*,*S*)-Ts-DPEN] in a mixture of formic acid–triethylamine and dimethylformamide at 25 °C. The experimental study performed on over 25 substrates combined with computational analysis revealed that a *Z*-configured methoxyimino group positioned alpha to a ketone carbonyl leads to higher reactivity and mostly excellent enantioselectivity within this substrate class. Density functional theory calculations of competing transition states were used in rationalizing the origins of enantioselectivity and the possible role of the methoxyimino group in the reaction outcome.

## 1 Introduction

**Scheme 1:**
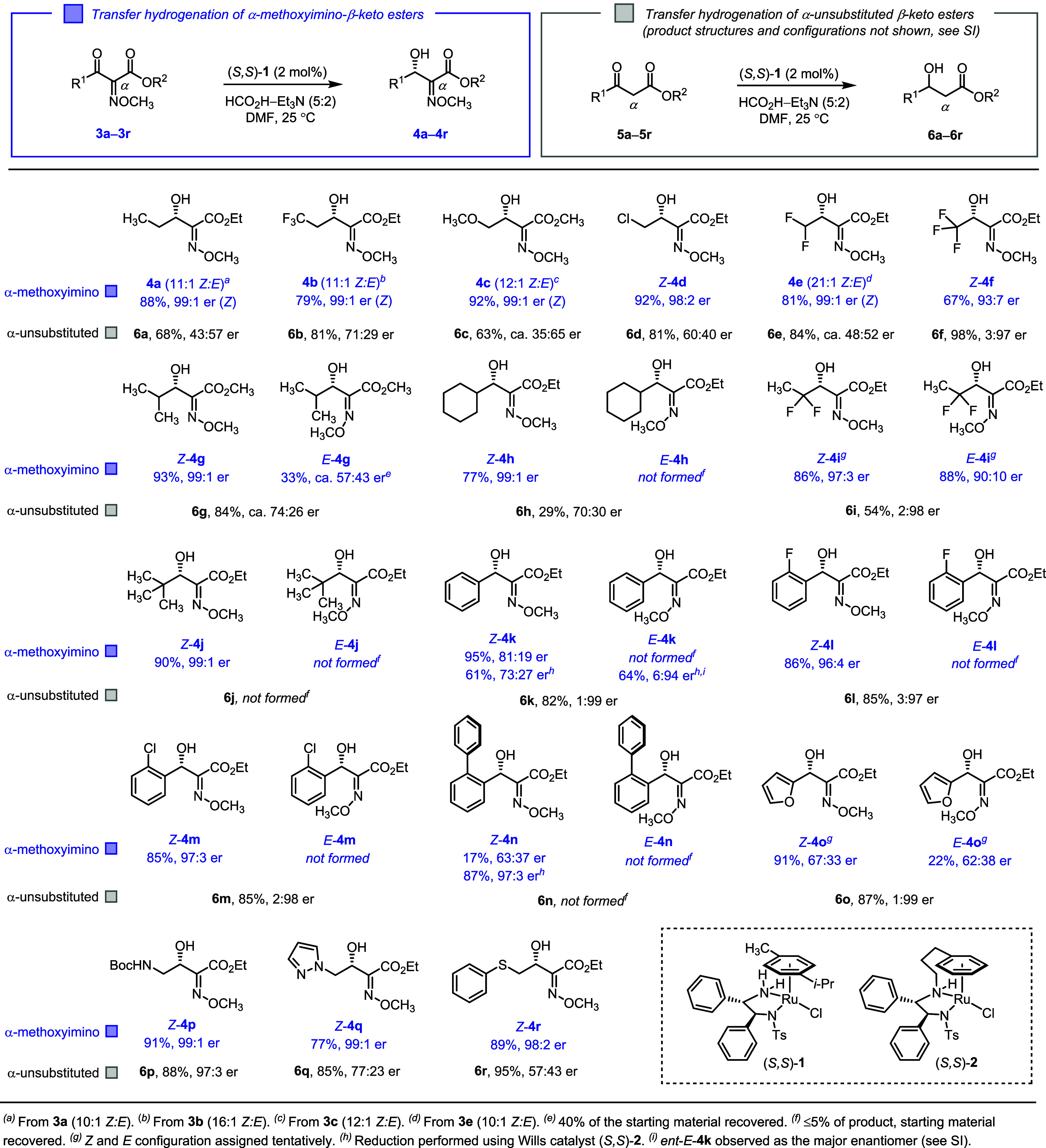
Scope of α-Methoxyimino-β-keto Esters Examined in the Asymmetric Transfer Hydrogenation Using the Complex (*S*,*S*)-**1** (Products **4a**–**4r**, Blue Squares), Shown Alongside Results with α-Unsubstituted-β-keto Esters (Products **6a**–**6r**, Grey Squares)

Asymmetric transfer hydrogenation of prochiral ketones is a powerful method for preparing enantiomerically enriched secondary alcohols.^1^ The chiral ruthenium(II) complex RuCl(*p*-cymene)[(*S*,*S*)-Ts-DPEN] (hereafter (*S*,*S*)-**1**) described by Noyori, Ikariya, and co-workers in 1995^2^ as well as the tethered analog (*S*,*S*)-**2** introduced by Wills in 2004^3^ (Scheme 1) represent examples of practical chemo- and enantioselective precatalysts for this transformation. Employing 2-propanol,^2^ formic acid–triethylamine mixtures,^4^ or aqueous sodium formate^5^ as convenient surrogates for flammable dihydrogen, the so-called Noyori–Ikariya ruthenium catalysts became omnipresent in academia and industry.^1,6−8^ The mechanistic understanding of the Noyori–Ikariya asymmetric transfer hydrogenation, including the basis of enantioselectivity, progressed from the original^9−11^ to the current.^12−17^

Until recently, the scope of the substrates that can be reduced with high enantioselectivity under the catalysis of the Noyori–Ikariya complexes has been limited to ketones bearing electron-rich substituents, such as mono- and di(hetero)aryl ketones as well as alkynyl ketones. Lower enantioselectivities were often seen with prochiral ketones lacking such functionalities.^1,6−8^

α-Heteroatom-substituted ketones are an important subclass of substrates used in asymmetric transfer hydrogenations with Noyori–Ikariya catalysts^18−34^ and include α-heteroatom-substituted β-keto esters well-established in dynamic kinetic resolution.^35−42^ The resulting products are valuable chiral building blocks in synthesis.^43^ The effect of the α-heteroatom substituent on the outcome of transfer hydrogenation can be significant^21^ and nontrivial to deconvolute mechanistically. In the context of developing a modular synthetic route to the bactobolin class of natural antibiotics,^44^ we carried out chemoselective and enantioselective reduction of hitherto unexplored α-methoxyimino-substituted β-keto esters^45,46^ using the commercially available Noyori–Ikariya transfer hydrogenation catalysts. Our preliminary results^44^ suggested that the methoxyimino group facilitates the reduction of these substrates and is beneficial for the stereochemistry-determining step. Here, we set out to better understand the methoxime effect in terms of substrate generality and the underpinning mechanistic details.

## 2 Results and Discussion

We subjected over 25 α-methoxyimino-β-keto esters to transfer hydrogenation catalyzed by the Noyori–Ikariya catalyst (*S*,*S*)-**1**, comparing the reactivity, enantioselectivity, and sense of stereoinduction. Specific substrates examined are depicted in Scheme 1 and were synthesized from the corresponding β-keto esters in two steps involving nitrosation and oxime methylation (see Supporting Information).^47^ In cases where mixtures of *Z* and *E* methoximes occurred, the diastereomers (geometric isomers) were separated by chromatography and subjected to asymmetric transfer hydrogenation individually. Unless noted otherwise, we employed the following reaction conditions: commercially available ruthenium(II) complex (*S*,*S*)-**1** (2 mol %) as the transfer hydrogenation precatalyst, freshly prepared triethylammonium formate (5:2 volume mixture of formic acid and triethylamine, respectively) as the dihydrogen equivalent,^4^ and dimethylformamide as solvent. All reductions were carried out at 25 °C unless noted otherwise. The reaction times varied depending on the specific substrate. For most *Z*-configured methoxime substrates, complete conversion was observed between 15 and 24 h under our standard conditions (see Supporting Information).

To dissect the effect of the α-methoxyimino substituent, we systematically compared the reaction outcomes of α-methoxyimino-substituted β-keto esters (**3a**–**3r**, blue squares) with α-unsubstituted β-keto esters (**5a**–**5r**, gray squares). The sense of stereoinduction for the various α-methoxyimino-substituted products (**4a**–**4r**) was the same as indicated in Scheme 1. In contrast, it varied for the α-unsubstituted products (**6a**–**6r**) as revealed below. Consequently, only the levels of enantioselectivity (er) are listed for the latter group (**6a**–**6r**, Scheme 1), with the full product structures and their absolute configurations provided in Supporting Information.

We first studied asymmetric transfer hydrogenations of α-methoxyimino-β-keto esters containing alkyl substituents at the ketone (R^1^ = alkyl). As shown in Scheme 1, linear, branched, and halogenated alkyl ketones containing the α-methoxyimino group exclusively or predominantly in the *Z* configuration underwent reduction with consistently high enantioselectivities (products **4a**–**j**, 93:7–99:1 er). The α-unsubstituted counterparts lacking the methoxyimino group (gray squares) gave inferior results (products **6a**–**j**). The two exceptions were substrates containing trifluoromethyl and 1,1-difluoroethyl groups (products *Z*-**4f** and *Z*-**4i**). Here, both the α-methoxyimino-substituted and the α-unsubstituted β-keto esters underwent the reduction with comparably high enantioselectivity but, strikingly, with the opposite sense of stereoinduction [e.g., *Z*-**4f** (93:7 er) versus **6f** (3:97 er) and *Z*-**4i** (97:3 er) versus **6i** (2:98 er)]. Within the fluorinated methoxime-free substrates, it is noteworthy that the 1,1-difluoroethyl group in **6i** effectively mimicked the known directing ability of the trifluoromethyl group^48^ in **6f**, whereas the difluoromethyl group did not (**6e**, ca. 48:52 er).

The asymmetric transfer hydrogenation of the isopropyl substrate containing α-methoxyimino group in the *E* configuration was noticeably slower and less enantioselective compared to the corresponding *Z* isomer [*Z*-**4g** (99:1 er, 93% yield after 16 h) versus *E*-**4g** (ca. 57:43 er, 33% yield after 10 days)]. This isomer-dependent effect was even more striking for the cyclohexyl and *tert*-butyl substrates (**3h** and **3j**), where the *Z* isomers afforded products in high yields and enantioselectivity (*Z*-**4h** and *Z*-**4j**), while the *E* isomers did not react (≤5% of anticipated products *E*-**4h** and *E*-**4j**). The α-unsubstituted counterparts were also poorly reactive under the same conditions (products **6h** and **6j**). These experiments clearly show the importance of the methoxyimino group and its configuration for substrate reactivity and enantioselectivity in the transfer hydrogenation (vide infra).

Next, we examined α-methoxyimino-β-keto esters containing aryl substituents at the ketone (R^1^ = aryl). These are interesting substrates due to potential competition between the established directing effect of aryl groups and the herein-studied methoxyimino group. We observed good-to-high enantioselectivity for the *Z*-configured methoxime substrates (products *Z*-**4k**–**m**), while the *E* isomers did not participate in the reduction using (*S*,*S*)-**1** (≤5% of anticipated products *E*-**4k**–**m**). Poor enantioselectivity was recorded for the 2-furyl-substituted substrate having either methoxime configuration [products *Z*-**4o** (67:33 er) and *E*-**4o** (62:38 er)], possibly an interplay between the directing effects of the furan and the methoxyimine. As expected, α-unsubstituted β-aryl β-keto esters underwent highly enantioselective transfer hydrogenation, though the sense of stereoinduction was opposite relative to the methoxyimino-substituted counterparts (**6k**–**m**, **6o**, 3:97–1:99 er). Transfer hydrogenation of the sterically demanding *Z*-configured 2-biphenylyl substrate leading to product *Z*-**4n** was slow and poorly enantioselective (17% yield after 6 days, 63:37 er) under the standard conditions. The substrate was effectively reduced upon switching to the more active Wills catalyst (*S*,*S*)-**2**^3^ (product *Z*-**4n**, 87% yield after 40 h, 97:3 er).

To confirm that the transfer hydrogenation is compatible with other functional groups, we subjected *N*-Boc-aminomethyl-, pyrazolomethyl-, and phenylthiomethyl-substituted *Z*-methoxime substrates **3p**–**r** to our standard conditions using (*S*,*S*)-**1**. The corresponding products *Z*-**4p**–**r** were obtained in good yields and with excellent enantioselectivity (98:2–99:1 er, Scheme 1). Interestingly, the α-unsubstituted *N*-Boc-aminomethyl substrate (**5p**) also displayed excellent enantioselectivity (product **6p**, 97:3 er).

**Scheme 2:**
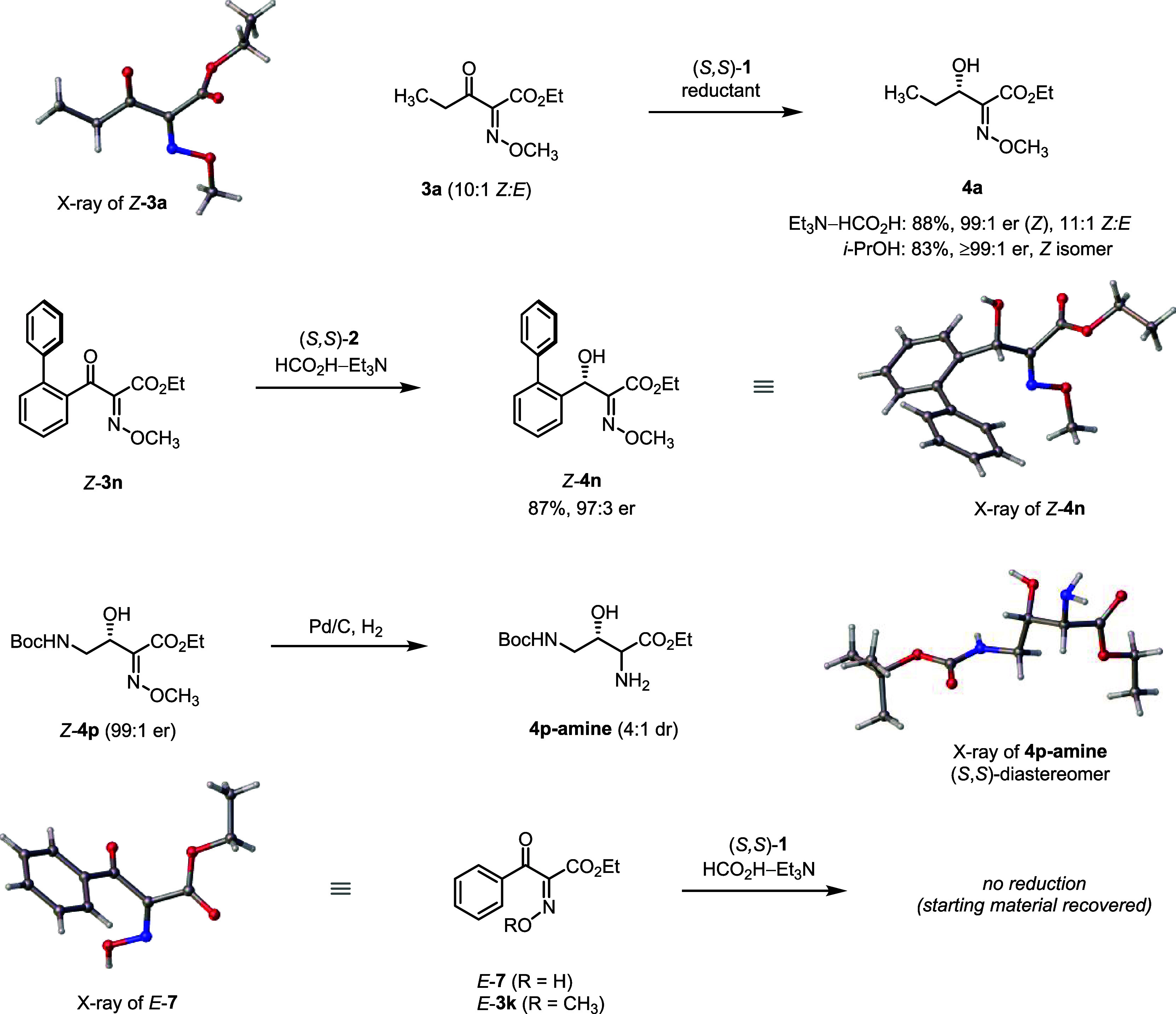
Selected Stereochemical Assignments as Determined by X-ray Crystallographic Analysis

The stereochemical configurations of the reduction products shown in Scheme 1 are based on the following data and observations. (1) We determined the X-ray crystal structure of the ethyl substrate *Z*-**3a** (Scheme 2), for which the *Z* configuration of the methoxyimino group agrees with previous reports.^49^ Substrate **3a**, used as a 10:1 *Z*/*E* mixture, underwent transfer hydrogenation catalyzed by (*S*,*S*)-**1** to give **4a** (11:1 *Z*/*E*) with excellent enantioselectivity (99:1 er, Scheme 2). (2) We obtained the crystal structure of the 2-biphenylyl product *Z*-**4n** (97:3 er using Wills catalyst (*S*,*S*)-**2**), which secured configurations of the secondary alcohol (*S*) and the methoxyimino group (*Z*). (3) Hydrogenation of methoxime *Z*-**4p** (99:1 er) delivered a 4:1 mixture of diastereomeric amines (**4p-amine**), where the absolute configuration of the (*S*,*S*)-diastereomer, presumed major, was established from its crystal structure (Scheme 2). (4) The known oxime *E*-**7** was unambiguously assigned by X-ray crystallography (Scheme 2).^49^ After methylation of oxime *E*-**7**, the corresponding *E* methoxime (*E*-**3k**) did not undergo the transfer hydrogenation catalyzed by (*S*,*S*)-**1** (no conversion under the standard conditions), fully consistent with the outcome reported in Scheme 1.^50^ (5) For several products of the transfer hydrogenation (**4a**–**4c**, **4e**, *Z*-**4f**–**i**, *Z*-**4k**), we removed the methoxyimino group in three steps to obtain the corresponding α-unsubstituted β-hydroxy esters. We note that during this three-step process (see Supporting Information), partial erosion of er for some of the substrates was observed.^51^ Nevertheless, by comparing signs of optical rotation of the products after methoxyimino group removal to those previously reported in the literature or prepared independently via transfer hydrogenation of the corresponding α-unsubstituted β-keto esters, we determined their relative configuration (see Supporting Information). (6) Configurations of four α-methoxyimino-β-hydroxy esters were correlated back after their incorporation into synthetic analogs of bactobolins.^44^ For the remaining examples in Scheme 1, the depicted stereochemical configurations are assumed and, thereby, tentative.

**Scheme 3:**
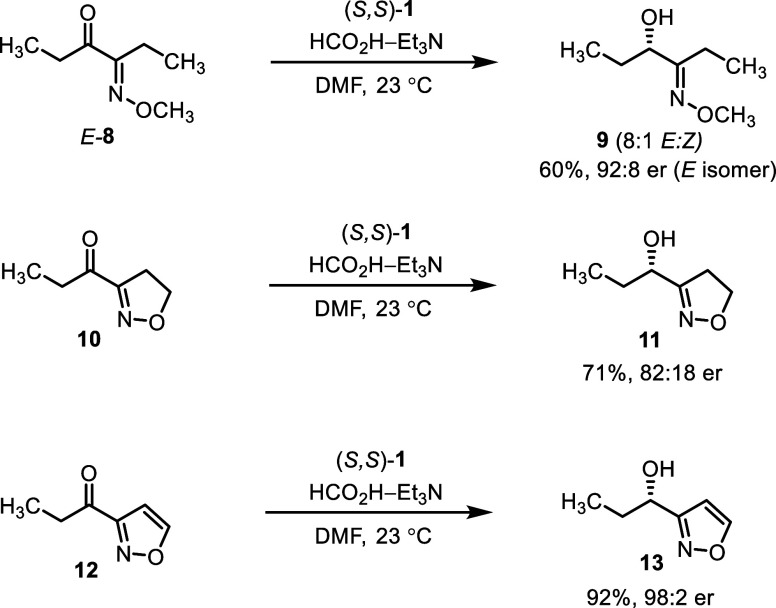
Asymmetric Transfer Hydrogenations of α-(1-Methoxyiminopropyl) (*E*-**8**), Isoxazolinyl (**10**), and Isoxazolyl (**12**) Ketones

To examine whether the effect of the methoxyimino group might be elicited by structurally similar functionalities, we carried out asymmetric transfer hydrogenations of 1-methoxyiminopropyl-, isoxazolinyl-, and isoxazolyl-substituted ketones *E*-**8**, **10**, or **12**, respectively (Scheme 3). As found, the corresponding alcohols **9**, **11**, and **13** were obtained in good-to-high enantioselectivity. Though not described previously, the outcome with the isoxazole-containing substrate **12** having the oxime-like atom arrangement is consistent with the directing effect of (hetero)aryl groups.^52^

The experiments described above firmly established the strong effect of the methoxyimino group, when positioned alpha to a carbonyl, on the reactivity and stereochemical outcome of asymmetric transfer hydrogenation in the presence of Noyori complex (*S*,*S*)-**1**. In considering the plausible origins of the effect, we inspected the literature on α-heteroatom-substituted ketone substrates.^18−42^ In terms of relative reactivity, the electron-withdrawing character of the methoxyimino group is expected to activate the neighboring carbonyl toward reduction. While this effect alone may explain, for example, the higher reactivity of methoxyimino-substituted substrates *Z*-**3h** and *Z*-**3j** relative to their unsubstituted counterparts **5h** and **5j**, the analysis fails with the corresponding *E* methoximes (*E*-**3h** and *E*-**3j**, Scheme 1). Various effects of the methoxyimino functionality could also be invoked in rationalizing the stereochemical outcome of the reduction. For example, the protonated amino group in α-substituted β-keto esters was previously suggested to help with substrate preorganization/activation via intramolecular hydrogen bonding.^36,40^ α-Amido, α-amino, and α-hydroxy ketones were proposed to engage in hydrogen bonding to the *N*-sulfonyl group of the diamine ligand during dynamic kinetic resolution.^30,31,34^ Iminium ions were suggested to participate in analogous hydrogen bonding during enantioselective transfer hydrogenations of imines.^53,54^ The directing effect of the protonated imidazole ring was invoked in rationalizing enantioselectivity of the reduction of *N*-methylimidazoyl aryl ketones.^52^

Based on the above, we first determined whether the neutral or the protonated oxime ether is the predominant reactive form of our substrates. UV–vis absorption spectra of methoxime **3a** (10:1 *Z*/*E*, precursor to **4a**) determined in *N*,*N*-dimethylformamide at varied concentrations of formic acid did not visibly change, supporting the predominant existence of the neutral form of the methoxyimino group. This would corroborate the expected lower basicity of α-methoxyimino ketones.^55^ Also, protonation of oximes and oxime ethers was reported to facilitate their *Z* ↔ *E* isomerization at ambient temperature.^56,57^ With the exception of substrate *E*-**8** (see Scheme 3), we did not observe significant *Z* ↔ *E* isomerization under our standard transfer hydrogenation conditions. Furthermore, the separable *Z* and *E* methoxime isomers often displayed dramatically different reactivity and enantioselectivity (see Scheme 1). Finally, we carried out the transfer hydrogenation of **3a** (10:1 *Z*/*E*) employing 2-propanol instead of triethylammonium formate—conditions, where the neutral form of the methoxyimino group can be assumed—to achieve a virtually identical level of enantioselectivity (≥99:1 er, Scheme 2). The above observations collectively indicate that the mechanism of transfer hydrogenation of substrates studied herein involves the nonprotonated (neutral) form of the methoxyimino group.

To rationalize the absence of *Z* ↔ *E* isomerization, the excellent enantioselectivity for the *Z* isomers, the higher enantioselectivity for the *Z* versus *E* isomers, and the differences in reactivity between these isomers, we performed computational analysis for the ethyl substrate **3a** and catalyst (*S*,*S*)-**1** by static density functional theory (DFT) calculations. The popular hybrid exchange–correlation functional B3LYP^58,59^ (with the global 20% orbital exchange fraction) parametrized via the D3 dispersion model^60^ was used to model *Z* → *E* ground and excited state isomerization of the substrate **3a** as well as stereoselectivity determining transition states leading to four stereoisomers of product **4a**. LACV3P**++//LACVP**+ level coupled with a polarizable continuum model in dimethylformamide was employed. To include the conformational thermostatistics in these calculations, conformational ensembles of transition state structures were generated using the Monte Carlo method based on OPLS4^61^ force fields by considering the 10 lowest conformers per stationary point, each of which was subsequently refined by DFT and Boltzmann averaged to obtain the final properties. The procedure was carried out using the fully automated Schrödinger Reaction Workflow.^62^

The computational analysis revealed that the *Z* ↔ *E* isomerization is indeed highly kinetically and slightly thermodynamically unfavorable (see Supporting Information). Although the *Z* and *E* isomers of ethyl substrate **3a** are separated by only 1.0 kcal/mol, the activation barrier for both thermal and photochemical *Z* ↔ *E* isomerization exceeds 45 kcal/mol. This corroborates the absence of scrambling of the methoxime stereochemistry under catalytic conditions used herein and the realized chromatographic separation of the *Z* and *E* isomers for selected substrates. The energy barrier for the *Z* ↔ *E* photoisomerization of substrate **3a**, which is prohibitively high with visible light, can be possibly overcome using UV light.^63^

**Figure 1:**
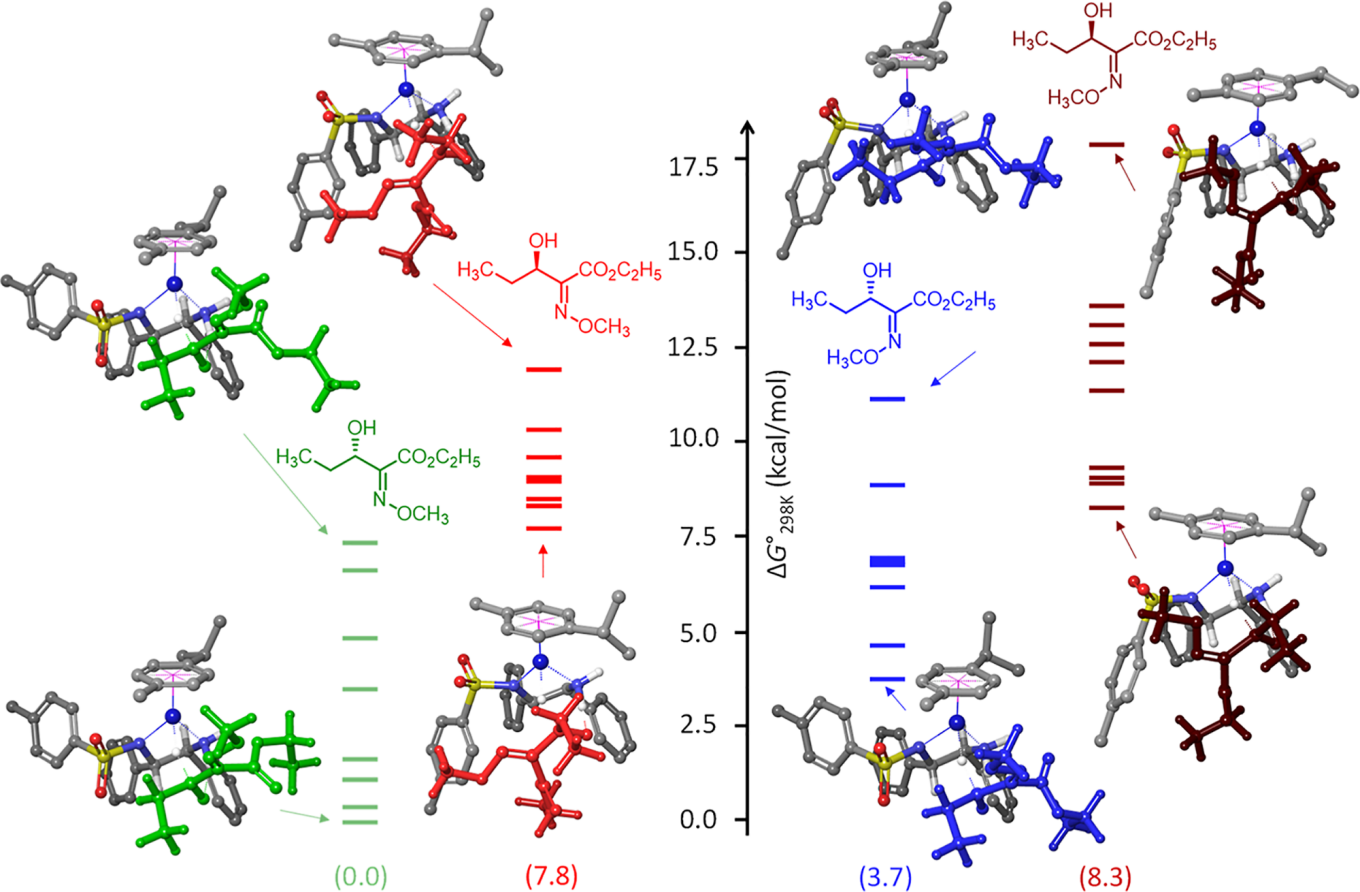
Relative ensembles of transition states free energy profile (limited to 10 conformers per transition state) and optimized geometries for upper/lower-bound transition states in the reduction of substrate **3a** leading to the four stereoisomers of product **4a**. Selected H atoms are omitted for clarity. Boltzmann-averaged relative free energies values are shown in brackets (298 K, 1 M).

Analysis of the conformational ensembles of the transition states leading to four stereoisomers of **4a** predicts that the Noyori–Ikariya catalyst (*S*,*S*)-**1** reduces both the *Z* and *E* isomers of the corresponding precursor substrate **3a** with selectivity toward the *S*-configured products, and with higher levels of enantioselectivity expected for the *Z* isomer (Figure 1). Furthermore, the *Z* isomer should be reduced faster, regardless of enantioselectivity. The slow kinetics of the *E* isomer seems to be due to the steric hindrance of the methoxyimino group. More generally, these data indicate that higher yields and enantioselectivities are to be expected for the *Z* isomer of any R^1^–C(O)–C(=NOMe)–CO_2_R^2^ relative to the *E* isomer under identical conditions (R^1^ is an alkyl group attached to the prochiral carbon atom). This is in excellent agreement with the experimental findings described in Scheme 1.

The degree of enantioselectivity for the *Z* or *E* isomer is expected to be determined by the interaction of the substrate with two spatial regions of the catalyst: the region of the η^6^-arene ligand and the region of the sulfonyl moiety (SO_2_).^16^ Dynamic equilibrium and interplay of attraction and repulsion via various noncovalent interactions within each region lead to stabilization/destabilization of the corresponding diastereomeric transition states and determine the final enantiomer ratio. Examination of transition state ensembles leading to the minor (*R*) enantiomer of **4a** from the *Z* and *E* isomers revealed that the sulfonyl moiety is rotated (along the S–N bond) away from the substrate in most examined conformers (Figure 1). Such structural reorganization has not been observed previously with aryl ketones^16^ and suggests a substantial repulsive SO_2_···substrate interaction, an effect that we attribute to the presence of the locally rigid methoxyimino group within the substrates studied herein. In contrast, the transition state ensembles leading to the major (*S*) enantiomer of **4a** avoid such repulsion and feature attractive interactions between the η^6^-arene ligand and the substrate, e.g., C–H···π(C=N). Noted additional interactions between the chiral ligand and the substrate through N–H···O (where O is OEt or C(O)=O of the ester group) also likely contribute to the high enantioselectivity.

**Scheme 4:**
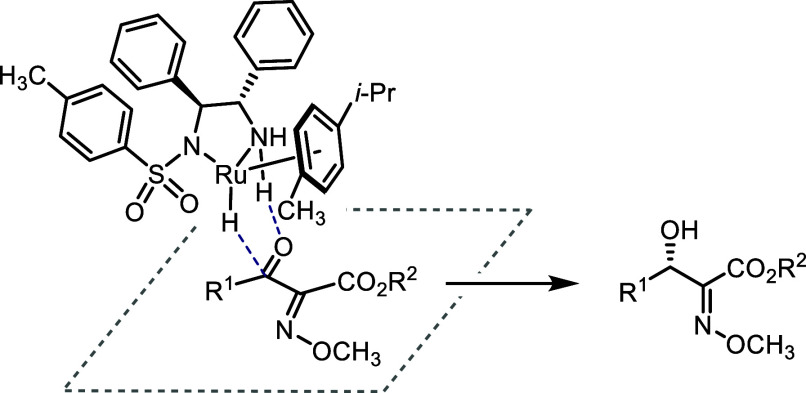
Simplified View of the Catalyst–Substrate Approach in Transfer Hydrogenation of *Z*-Configured α-Methoxyimino-β-keto Esters Using Complex (*S*,*S*)-**1** Leading to the Experimentally Observed Product Stereochemistry

In a simplified view, the configuration of the transfer hydrogenation products obtained with complex (*S*,*S*)-**1** can be arrived at by adopting the coplanar arrangement of a α-methoxyimino-β-keto ester as in Scheme 4, with the methoxyimino group facing the η^6^-arene region of the approaching (*S*,*S*,*R*_Ru_) ruthenium(II) hydride (for detailed 3D renderings of calculated low-energy transition states, see Figure 1).

## 3 Conclusions

In conclusion, we have demonstrated that α-methoxyimino-β-keto esters represent a class of substrates that can be reduced with excellent enantioselectivity in the presence of commercially available Noyori–Ikariya complex (*S*,*S*)-**1**. A properly configured methoxyimino group (*Z* isomer) positioned alpha to an aryl- or alkyl-substituted keto group was shown to direct the stereochemical outcome and facilitate the reduction. Computational analysis was used to rationalize the origin of high enantioselectivity and the observed dramatic differences in reactivity between *Z* and *E* isomers of the methoxyimino group. The work expands the previous list of β-keto ester substrates effective in asymmetric transfer hydrogenation and provides an enantioselective route to functionalized oxime-containing building blocks for organic synthesis.^44,64−66^

## 4 Experimental Section

### 4.1 General Considerations

All reactions were performed in round-bottom flasks fitted with rubber septa under a positive pressure of argon, unless noted otherwise. All reactions were monitored by thin-layer chromatography (TLC) using aluminum plates precoated with silica gel (silica gel 60 F254, Merck) impregnated with a fluorescent indicator. TLC plates were visualized by exposure to ultraviolet light (λ = 254 nm) and/or by submersion in aqueous ceric ammonium molybdate, aqueous potassium permanganate (KMnO_4_), ethanolic phosphomolybdic acid (PMA), ethanolic *p*-anisaldehyde (ANIS) solutions followed by brief heating. All solutions were concentrated by rotary evaporation at 40 °C, unless noted otherwise. Flash-column chromatography (FCC) was performed using silica gel (60 Å, 230–400 mesh, Sigma-Aldrich).

### 4.2 Materials

All reagents purchased from commercial suppliers (Sigma-Aldrich, Acros Organics, Fluorochem) were used without further purification. All solvents were used as received. RuCl(*p*-cymene)[(*S*,*S*)-Ts-DPEN] [(*S*,*S*)-**1**] was purchased from Sigma-Aldrich, while RuCl[(*S*,*S*)-Teth-Ts-DPEN] (Wills catalyst, (*S*,*S*)-**2**) was purchased from Strem Chemicals. Formic acid–triethylamine mixture used in all transfer hydrogenation experiments was prepared fresh prior to the reaction by adding triethylamine to neat formic acid at 0 °C under argon. Caution!*(1) Dimethyl sulfate (DMS) is a potent alkylating and toxic agent. (2) Isopentyl nitrite is a toxic, flammable, and potentially explosive agent*.

### 4.3 Instrumentation

Proton nuclear magnetic resonance (^1^H NMR) spectra were recorded using Bruker AVANCE 500 (500 MHz) or Bruker AVANCE 300 (300 MHz) NMR spectrometers at 30 °C. Proton chemical shifts are expressed in parts per million (ppm, δ scale) and are referenced to residual protium in the NMR solvents (CHCl_3_: δ = 7.27 ppm, CD_2_HOD: δ = 3.21 ppm (quint), (CD_2_H)_2_CO: δ = 2.07 ppm (quint)). Data are represented as follows: chemical shift, multiplicity (s = singlet, d = doublet, t = triplet, q = quartet, quint = quintet, m = multiplet and/or multiple resonances, app = apparent, br = broad), coupling constants (*J*) in Hertz, integration. Carbon nuclear magnetic resonance (^13^C NMR) spectra were recorded using Bruker Avance 500 (126 MHz) or Bruker AVANCE 300 (76 MHz) NMR spectrometers at 30 °C. Carbon chemical shifts are expressed in parts per million (ppm, δ scale) and are referenced to the carbon resonance of the NMR solvent. Fourier transform infrared (FTIR) spectra were obtained using ALPHA Bruker FTIR spectrometer equipped with a diamond ATR adaptor. Optical rotations were measured on AUTOPOL IV polarimeter using a 0.8 mL polarimetric cell at 23 °C (instrument room temperature). Optical rotation data are reported in the following format: specific rotation , concentration (g/100 mL), and solvent. High-resolution mass spectra were obtained on Agilent 6224 Accurate-Mass TOF LC–MS with dual electrospray/chemical ionization mode. HPLC analyses were performed on Thermo 1260 Infinity device or DIONEX Ultimate 3000SD device. GC analyses were performed on Agilent 6850 instrument using BetaDex 120 column (Supelco 24304 Betide 120, column length 30 m, inner diameter 0.25 mm, thickness of stationary phase 0.25 μm. Stationary phase is “nonbonded; 20% permethylated β-cyclodextrin in SPB-35 (poly(35% phenyl/65% dimethylsiloxane) phase).

### 4.4 Representative Procedure for the Synthesis of α-Methoxyimino-β-keto Esters (Substrate **3a**)

A solution of sodium nitrite (700 mg, 10.15 mmol, 1.3 equiv) in water (1.3 mL) was added dropwise to a solution of ethyl 3-oxopentanoate (1.13 g, 7.9 mmol, 1 equiv) in acetic acid (3.2 mL) over 30 min at 0 °C. The resulting mixture was stirred at this temperature for 1 h (TLC: 30% ethyl acetate in hexane; UV, KMnO_4_). Then, the mixture was poured into brine (35 mL) and extracted with ether (3 × 30 mL). The organic extracts were combined and washed with a saturated aqueous solution of sodium hydrogen carbonate (150 mL) to reach pH ∼ 7, and the aqueous phase was extracted again with ether (3 × 35 mL). All organic extracts were combined, dried over anhydrous sodium sulfate, and filtered. The filtrate was concentrated under reduced pressure to yield crude α-hydroxyimino ester (1.14 g, not shown), which was used in the next step without further purification.

Potassium carbonate (1.18 g, 8.5 mmol, 1.3 equiv) was added to a stirred solution of the above-prepared crude α-hydroxyimino ester (1.14 g, 6.6 mmol, 1 equiv) in anhydrous tetrahydrofuran (20 mL) at 0 °C. After 5 min of stirring at 0 °C, dimethyl sulfate (0.56 mL, 5.9 mmol, 0.9 equiv) was added at 0 °C, and the resulting solution was allowed to warm to room temperature and stirred at this temperature for 17 h (TLC: 30% ethyl acetate in hexane). The reaction mixture was filtered, ice-cold brine (40 mL) was added, and the resulting mixture was extracted with dichloromethane (3 × 40 mL). The combined organic phases were dried over anhydrous sodium sulfate, the dried solution was filtered, and the filtrate was concentrated in vacuo. The obtained residue was purified by FCC (gradient elution with 7–8% ethyl acetate in hexane) to provide α-methoxyimino ester **3a** as a colorless oil (1.01 g, 82%, 10:1 mixture of *Z*/*E* isomers). Single crystals of *Z*-**3a** for X-ray analysis were obtained by allowing the 10:1 *Z*/*E* mixture of **3a** to stand neat at 4 °C.

α-Methoxyimino ester **3a**: TLC (30% ethyl acetate in hexane): *R*_f_ (*Z* isomer) = 0.75, *R*_f_ (*E* isomer) = 0.68. ^1^H NMR (500 MHz, CDCl_3_, 10:1 mixture of *Z*/*E* isomers; only signals corresponding to the major isomer are listed) δ: 4.34 (q, *J* = 7.1 Hz, 2H), 4.08 (s, 3H), 2.81 (q, *J* = 7.3 Hz, 2H), 1.33 (t, *J* = 7.1 Hz, 3H), 1.12 (t, *J* = 7.4 Hz, 3H). ^13^C NMR (126 MHz, CDCl_3_, 10:1 mixture of *Z*/*E* isomers; only signals corresponding to the major isomer are listed) δ: 195.9, 161.4, 149.7, 64.4, 62.2, 31.1, 14.2, 7.7. FTIR (neat), cm^–1^: 2984, 2944, 1742, 1693, 1601, 1461, 1371, 1288, 1212, 1087, 1035, 961, 903, 859, 806, 679. HRMS (APCI): Calcd for [C_8_H_13_NO_4_+H]^+^: 188.0917, found: 188.0916.

### 4.5 Representative Procedure for Asymmetric Transfer Hydrogenation of α-Methoxyimino-β-keto Esters (Product **4a**)

A solution of (*S*,*S*)-**1** (34.0 mg, 53.5 μmol, 0.02 equiv) in anhydrous *N*,*N*-dimethylformamide (1.5 mL) was evacuated and backfilled with argon (4 cycles). Then, the above-prepared α-methoxyimino ester **3a** (500 mg, 2.67 mmol, 1 equiv, 10:1 mixture of *Z*/*E* isomers) was added as a solution in anhydrous *N*,*N*-dimethylformamide (0.7 mL), and the mixture was stirred for 5 min in order to obtain a clear solution. Then, argon was bubbled through the solution for 15 min (outlet needle), and a double-layered balloon filled with argon was attached. A mixture of formic acid and triethylamine (5:2 by volume, 1.34 mL) was added, followed by stirring for 16 h at 25 °C (TLC: 30% ethyl acetate in hexane, UV, PMA). Ice-cold water (30 mL) was added, and the resulting mixture was extracted with ethyl acetate (3 × 30 mL). The combined organic phases were washed with brine (25 mL), dried over anhydrous sodium sulfate, the dried solution was filtered, and the filtrate was concentrated in vacuo. The obtained residue was purified by FCC (elution with 25% ethyl acetate in hexane) to provide alcohol **4a** as a gray oil (446 mg, 88%, 11:1 mixture of *Z*/*E* isomers). The enantiomeric purity of **4a** was determined by HPLC (99:1 er).

Product **4a**: TLC (20% ethyl acetate in hexane): *R*_f_ = 0.3. ^1^H NMR (500 MHz, CDCl_3_, 11:1 mixture of *Z*/*E* isomers; only signals corresponding to the major isomer are listed) δ: 4.32 (m, 1H), 4.32 (q, *J* = 7.2 Hz, 2H), 3.90 (s, 3H), 2.47 (br s, 1H), 1.73 (m, 2H), 1.33 (t, *J* = 7.2 Hz, 3H), 0.99 (t, *J* = 7.4 Hz, 3H). ^13^C NMR (126 MHz, CDCl_3_, 11:1 mixture of *Z*/*E* isomers; only signals corresponding to the major isomer are listed) δ: 162.6, 152.6, 72.0, 63.0, 62.0, 27.9, 14.4, 9.4. FTIR (neat), cm^–1^: 3431, 2973, 2940, 1730, 1305, 1199, 1159, 1095, 1033, 982, 884. HRMS (APCI): Calcd for [C_8_H_15_NO_4_+H]^+^: 190.1074, found: 190.1075.

### 4.6 Computational Analysis

DFT calculations were performed with the Jaguar code^67^ (selected jaguar_keywords ’iuhf = 2 nops = 1 maxit = 150 maxitg = 100 iaccg = 2 nofail = 0 isymm = 8 isolv = 7 solvent = dimethylformamide’). Pseudospectral method was used to model thermal and photoexcited *Z*-to-*E* isomerization pathway for **3a** (selected -jaguar_keywords ’iuhf = 2 nops = 0 iacc = 3 maxit = 150 maxitg = 100 iaccg = 2 nofail = 1 isymm = 8 isolv = 7 solvent = dimethylformamide ts_vet_dist_fac0 = 0.7′ for ground state and ’iuhf = 2 nops = 0 iacc = 3 maxit = 150 maxitg = 100 iaccg = 2 nofail = 1 isymm = 8 isolv = 7 solvent = dimethylformamide’ for triplet state). Conformational analysis was performed with MacroModel code^68^ with -dedup_geom_eps 0.25 flag. Reaction Workflow (RXNWF) module of Schrödinger Materials Science Suite^62^ available via graphics user interface was used to automate the calculations. For example, the input to model thermal and photoexcited *Z*-to-*E* isomerization pathway for **3a** is Cartesian coordinates for three and five DFT preoptimized stationary points (minima and transition states), whereas the output is 30 and 50 DFT optimized stationary points (ten lowest energy conformers per stationary point were considered) and Boltzmann-averaged properties, respectively. Likewise, the input for Figure 1 is Cartesian coordinates for four DFT preoptimized transition states, and the output is 40 DFT-optimized transition states and Boltzmann-averaged properties. Because two transition states (out of 40) failed, specifically the highest-energy conformers for the transition states leading to *Z*-(*S*)-**4a** and *Z*-(*R*)-**4a** from *Z*-**3a**, the results are reported for the conformer space of 38.

## Data Availability

The data underlying this study are available in the published article and its Supporting Information.

## Supporting Information Available

Contains detailed synthetic procedures, crystallographic data for compounds *Z*-**3a**, *Z*-**4n**, **4p-amine**, and *E*-**7**, computational data, copies of ^1^H and ^13^C NMR spectra for all new compounds, and copies of HPLC and GC chromatograms (PDF)
