# Supplementary material for: Enantioselective Transfer Hydrogenation of α-Methoxyimino-β-keto Esters
Source: J Org Chem. 2024 Aug 30;89(18):12902–11. doi: 10.1021/acs.joc.4c00381 (PMC11421019; doi:10.1021/acs.joc.4c00381)
Supplement: Supplementary file 1 — jo4c00381_si_001.pdf [file jo4c00381_si_001.pdf]

Supporting Information

Enantioselective Transfer Hydrogenation of α-Methoxyimino-β-Keto Esters

Prabhakara R. Tharra[a,b], Jiří Švejkar[a], Abhijeet S. Jadhav[a], Marek Nečas[a], Pavel A. Dub[c], Mathew D. Halls[c] and Jakub Švenda*[a,b]

[a] Dr. P. R. Tharra, J. Švejkar, Dr. A. S. Jadhav, M. Nečas, Dr. J. Švenda
Department of Chemistry, Faculty of Science, Masaryk University
Kamenice 5, Brno, 625 00, Czech Republic
E-mail: svenda@chemi.muni.cz

[b] Dr. P. R. Tharra, Dr. J. Švenda
International Clinical Research Center, St. Anne's University Hospital
Pekařská 53, Brno, 656 91, Czech Republic

[c] Dr. P. A. Dub, Dr. M. D. Halls
Schrödinger, Inc., San Diego, California 92121, United States

Table of Contents

General experimental procedures S2
Materials S2
Instrumentation S2
Stereochemical assignments S2
Note on reported values of optical rotation S3
Experimental procedures S3
X-ray crystallography S73
Computational analysis S77
Computation data S78
References S189
Copies of ^{1}H and $^{13}C\{^{1}H\}$ NMR spectra S191
Copies of HPLC and GC chromatograms S286

General Experimental Procedures

All reactions were performed in round-bottom flasks fitted with rubber septa under a positive pressure of argon, unless noted otherwise. All reactions were monitored by thin-layer chromatography (TLC) using aluminum plates pre-coated with silica gel (silica gel 60 F254, Merck) impregnated with a fluorescent indicator. TLC plates were visualized by exposure to ultraviolet light (λ = 254 nm) and/or by submersion in aqueous ceric ammonium molybdate (CAM), aqueous potassium permanganate ($KMnO_4$), ethanolic phosphomolybdic acid (PMA), ethanolic *p*-anisaldehyde (ANIS) solutions followed by brief heating. All solutions were concentrated by rotary evaporation at 40 °C, unless noted otherwise. Flash-column chromatography (FCC) was performed using silica gel (60 Å, 230–400 mesh, Sigma-Aldrich).

Materials

All reagents purchased from commercial suppliers (Sigma-Aldrich, Acros Organics, Fluorochem) were used without further purification. All solvents were used as received. RuCl(*p*-cymene)[(*S,S*)-Ts-DPEN] (*S,S*)-**1** was purchased from Sigma-Aldrich, while (*S,S*)-Teth-TsDpen RuCl (Wills catalyst (*S,S*)-**2**) was purchased from Strem Chemicals. Formic acid–triethylamine mixture used in all transfer hydrogenation experiments was prepared fresh prior to the reaction by adding triethylamine to neat formic acid at 0 °C under argon. *Caution! (1) Dimethyl sulfate (DMS) is a potent alkylating agent and toxic. (2) Isopentyl and sodium nitrites are toxic, flammable, and potentially explosive agents.*

Instrumentation

Proton nuclear magnetic resonance (^{1}H NMR) spectra were recorded using Bruker Avance 500 (500 MHz) or Bruker Avance 300 (300 MHz) NMR spectrometers at 30 °C. Proton chemical shifts are expressed in parts per million (ppm, δ scale) and are referenced to residual protium in the NMR solvents ($CHCl_3$: δ = 7.27 ppm, CD_2HOD: δ = 3.21 ppm (quint), $(CD_2H)_2CO$: δ = 2.07 ppm (quint). Data are represented as follows: chemical shift, multiplicity (s = singlet, d = doublet, t = triplet, q = quartet, quint = quintet, m = multiplet and/or multiple resonances, app = apparent, br = broad), coupling constants (*J*) in Hertz, integration. Carbon nuclear magnetic resonance ($^{13}C\{^1H\}$ NMR) spectra were recorded using Bruker Avance 500 (126 MHz) or Bruker Avance 300 (76 MHz) NMR spectrometers at 30 °C. Carbon chemical shifts are expressed in parts per million (ppm, δ scale) and are referenced to the carbon resonance of the NMR solvent. Fourier transform infrared (FTIR) spectra were obtained using ALPHA Bruker FTIR spectrometer equipped with a diamond ATR adaptor. Optical rotations were measured on AUTOPOL IV polarimeter using a 0.8 mL polarimetric cell at 23 °C (instrument room temperature). Optical rotation data are reported in the following format: specific rotation $[\alpha]_\lambda^T$, concentration (g/100 mL), and solvent. High-resolution mass spectra were obtained on Agilent 6224 Accurate-Mass TOF LC-MS with dual electrospray/chemical ionization mode. HPLC analyses were performed on Thermo 1260 Infinity device or DIONEX Ultimate 3000SD device. GC analyses were performed on Agilent 6850 instrument using BetaDex 120 column (Supelco 24304 Betide 120, column length 30 m, inner diameter 0.25 mm, thickness of stationary phase 0.25 μm. Stationary phase is "non-bonded; 20% permethylated β-cyclodextrin in SPB-35 (poly(35% phenyl/65% dimethylsiloxane) phase).

Stereochemical assignments

The *Z* and *E* configurations of methoximes featuring in this study were unambiguously assigned by X-ray crystallography for three examples, specifically ethyl methoxime *Z*-**3a**, biphenyl

methoxime *Z*-**4n**, and phenyl oxime *E*-**7** (precursor to *E*-**3k**). Furthermore, where possible, we compared NMR data of our substrates to oximes or methoximes previously reported in the literature.[1] We have used this data in assuming configurations of other subtrates in Scheme 1 (main manuscript) by analogy. There was a strong correlation between the *Z* configuration of methoximes and the outcome of the asymmetric transfer hydrogenation (high enantioselectivity) across the substrates examined. One exception was the case of difluoroethyl substrate **3i**, where we observed both methoxime isomers to perform comparably well. Depicted *Z* and *E* configurations in this case are therefore tentative.
The absolute configuration of the products of asymmetric transfer hydrogenation reported in Scheme 1 (main manuscript) was assigned by different methods. Firstly, for selected products, we removed the methoxyimino group and compared the sign of optical rotation of the resulting β-hydroxy ester to the product obtained by asymmetric transfer hydrogenation of the corresponding β-keto ester (i.e., substrate without the methoxyimino group) using the same catalyst. For products **6a**, **6d**, **6f**, **6k**, **6m**, **6o**, **6p**, and **6r** the absolute configuration of the β-hydroxy esters is known from the literature.[2] Secondly, we have determined the absolute configuration of the biphenyl-substituted reduction product *Z*-**4n** and also **4p-amine** ((*S,S*) diastereomer) derived from *Z*-**4p** by X-ray crystallography (depicted in Scheme 2 of the main manuscript). Thirdly, we have previously converted *ent*-**4a**, *ent*-**4b**, *ent*-**4e**, and *ent*-**4i** (all obtained by asymmetric transfer hydrogenation of the corresponding methoxyimino substrates using (*R,R*)-**1** as catalyst) to the corresponding bactobolin analogs. Therein, NMR-based correlations were used to trace back the absolute stereochemistry.[3]

Note on the reported values of optical rotation

The values of optical rotation we report in this work frequently do not correspond to the enantiomerically pure compounds and are not intended as reference values. We have used the sign of optical rotation (+ or –) solely to decide whether the absolute configuration of the product prepared by different methods/routes is the same or opposite.

Experimental procedures

Enantioselective transfer hydrogenation of ethyl substrate 3a

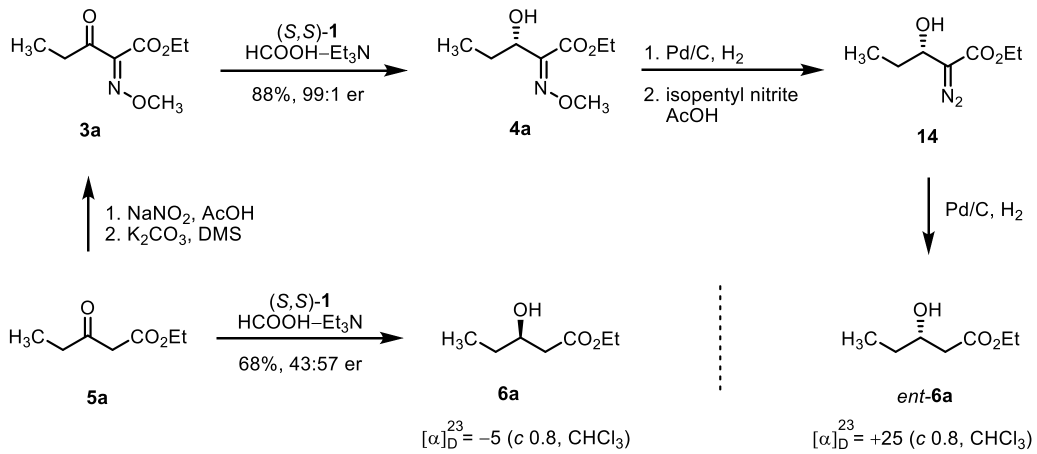

$[\alpha]_D^{23}$ = –5 (*c* 0.8, $CHCl_3$)

$[\alpha]_D^{23}$ = +25 (*c* 0.8, $CHCl_3$)

Synthesis of ethyl 3-oxo-2-(methoxyimino)pentanoate 3a

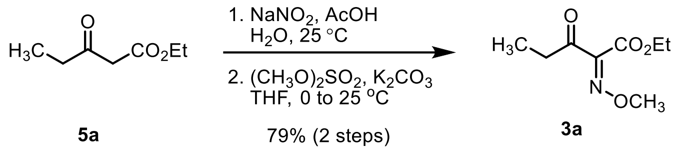

A solution of sodium nitrite (700 mg, 10.15 mmol, 1.3 equiv) in water (1.3 mL) was added dropwise to a solution of ethyl 3-oxopentanoate **5a** (1.13 g, 7.9 mmol, 1 equiv) in acetic acid (3.2 mL) over 30 min at 0 °C. The resulting mixture was stirred at this temperature for 1 h (TLC: 30% ethyl acetate in hexane; UV, $KMnO_4$). Then, the mixture was poured into brine (35 mL) and extracted with ether (3 × 30 mL). The organic extracts were combined and washed with a saturated aqueous solution of sodium hydrogen carbonate (150 mL) to reach pH ~ 7, and the aqueous phase was extracted again with ether (3 × 35 mL). All organic extracts were combined, dried over anhydrous sodium sulfate, and filtered. The filtrate was concentrated under reduced pressure to yield the crude α-hydroxyimino ester[4] (1.14 g, not shown), which was used in the next step without further purification.

Potassium carbonate (1.18 g, 8.5 mmol, 1.3 equiv) was added to a stirred solution of crude α-hydroxyimino ester (1.14 g, 6.6 mmol, 1 equiv) in anhydrous tetrahydrofuran (20 mL) at 0 °C. After 5 min of stirring at 0 °C, dimethyl sulfate (0.56 mL, 5.9 mmol, 0.9 equiv) was added at 0 °C, and the resulting solution was allowed to warm to room temperature and stirred at this temperature for 17 h (TLC: 30% ethyl acetate in hexane). The reaction mixture was filtered, ice-cold brine, 40 mL) was added, and the resulting mixture was extracted with dichloromethane (3 × 40 mL). The combined organic phases were dried over anhydrous sodium sulfate, the dried solution was filtered, and the filtrate was concentrated in vacuo. The obtained residue was purified by FCC (gradient elution with 7–8% ethyl acetate in hexane) to provide α-methoxyimino ester **3a** as a colorless oil (1.01 g, 82%, 10:1 mixture of *Z*:*E* isomers). Single crystals of *Z*-**3a** for X-ray analysis were obtained by allowing the compound stand neat at 4 °C.

<u>α-Methoxyimino ester **3a**:</u> TLC (30% ethyl acetate in hexane): R_f (*Z* isomer) = 0.75, R_f (*E* isomer) = 0.68. ^{1}H NMR (500 MHz, $CDCl_3$, 10:1 mixture of *Z*:*E* isomers; only signals corresponding to the major isomer are listed) δ: 4.34 (q, *J* = 7.1 Hz, 2H), 4.08 (s, 3H), 2.81 (q, *J* = 7.3 Hz, 2H), 1.33 (t, *J* = 7.1 Hz, 3H), 1.12 (t, *J* = 7.4 Hz, 3H). $^{13}C\{^1H\}$ NMR (126 MHz, $CDCl_3$, 10:1 mixture of *Z*:*E* isomers; only signals corresponding to the major isomer are listed) δ: 195.9, 161.4, 149.7, 64.4, 62.2, 31.1, 14.2, 7.7. FTIR (neat), cm^{-1}: 2984, 2944, 1742, 1693, 1601, 1461, 1371, 1288, 1212, 1087, 1035, 961, 903, 859, 806, 679. HRMS (APCI): Calcd for $[C_8H_{13}NO_4+H]^+$: 188.0917, found: 188.0916.

Synthesis of ethyl (*S*)-3-hydroxy-2-(methoxyimino)pentanoate 4a

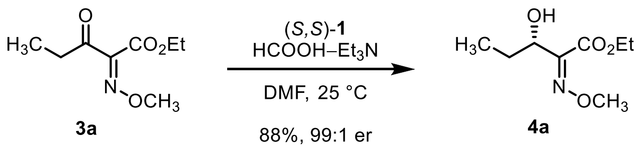

A solution of (*S,S*)-**1** (34.0 mg, 53.5 μmol, 0.02 equiv) in anhydrous *N*,*N*-dimethylformamide (1.5 mL) was evacuated and backfilled with argon (4 cycles). Then, the above-prepared α-methoxyimino ester **3a** (500 mg, 2.67 mmol, 1 equiv, 10:1 mixture of *Z*:*E* isomers) was added as a solution in anhydrous *N*,*N*-dimethylformamide (0.7 mL), and the mixture was stirred for 5 min in order to obtain a clear solution. Then, argon was bubbled through the solution for 15 min (outlet needle), and a double-layered balloon filled with argon was attached. A mixture of formic acid and triethylamine (5:2 by volume, 1.34 mL) was added, followed by stirring for 16 h at 25 °C (TLC: 30% ethyl acetate in hexane, UV, PMA). Then, ice-cold water (30 mL) was added, and the resulting mixture was extracted with ethyl acetate (3 × 30 mL). The combined organic phases were washed with brine (25 mL), dried over anhydrous sodium sulfate, the dried solution was filtered, and the filtrate was concentrated in vacuo. The obtained residue was purified by FCC (elution with 25% ethyl acetate in hexane) to provide alcohol **4a** as a grey oil (446 mg, 88%, 11:1 mixture of *Z*:*E* isomers). The enantiomeric purity of **4a** was determined by HPLC (99:1 er).

Alcohol **4a**: TLC (20% ethyl acetate in hexane): R_f= 0.3. ^{1}H NMR (500 MHz, $CDCl_3$, 11:1 mixture of *Z*:*E* isomers; only signals corresponding to the major isomer are listed) δ: 4.32 (m, 1H), 4.32 (q, *J* = 7.2 Hz, 2H), 3.90 (s, 3H), 2.47 (br s, 1H), 1.73 (m, 2H), 1.33 (t, *J* = 7.2 Hz, 3H), 0.99 (t, *J* = 7.4 Hz, 3H). ^{13}C{^{1}H} NMR (126 MHz, $CDCl_3$, 11:1 mixture of *Z*:*E* isomers; only signals corresponding to the major isomer are listed) δ: 162.6, 152.6, 72.0, 63.0, 62.0, 27.9, 14.4, 9.4. FTIR (neat), cm^{-1}: 3431, 2973, 2940, 1730, 1305, 1199, 1159, 1095, 1033, 982, 884. HRMS (APCI): Calcd for $[C_8H_{15}NO_4+H]^+$: 190.1074, found: 190.1075.

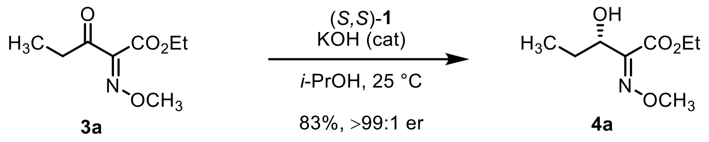

A solution of (*S,S*)-**1** (13.0 mg, 20.4 μmol, 0.02 equiv) in 2-propanol (8.0 mL) was evacuated and backfilled with argon (4 cycles). Then, α-methoxyimino ester **3a** (191 mg, 1.02 mmol, 1 equiv, 10:1 mixture of *Z*:*E* isomers) was added as a solution in 2-propanol (1 mL), and the mixture was stirred for 5 min in order to obtain a clear solution. Then, argon was bubbled through the solution for 15 min (outlet needle), and a double-layered balloon filled with argon was attached. Then, degassed solution of potassium hydroxide (1.7 mg, 30.3 μmol, 0.03 equiv) in 2-propanol (1 mL) was added and the mixture was stirred for 4.5 h at 25 °C (TLC: 20% ethyl acetate in hexane, UV, PMA). Then, the reaction mixture was evaporated in vacuo, and the residue was dissolved in ethyl acetate (10 mL) and water (10 mL). The mixture was stirred for 5 min and then brine (10 mL) was added. The aqueous phase was extracted with ethyl acetate (3 × 25 mL). The combined organic phases were dried over anhydrous sodium sulfate, the dried solution was filtered, and the filtrate was concentrated in vacuo. The obtained residue was purified by FCC (gradient elution with 5–20% ethyl acetate in hexane) to provide alcohol **4a** as an oil (161 mg, 83%, *Z* isomer). The enantiomeric purity of **4a** was determined by HPLC (≥99:1 er).

Synthesis of ethyl (*S*)-2-diazo-3-hydroxypentanoate 14

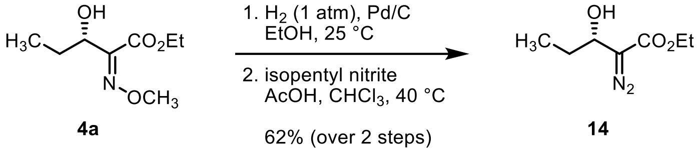

Palladium on charcoal (10% Pd basis; 44 mg) was added to a stirred solution of alcohol **4a** (220 mg, 1.16 mmol, 1 equiv) in ethanol (6.0 mL) at 25 °C. After stirring of the suspension for 10 min, the reaction flask was evacuated, and a double layered balloon filled with hydrogen gas was attached. The mixture was vigorously stirred for 3 days at 25 °C (TLC: 30% ethyl acetate in hexane; ninhydrin). Filtration through a syringe PTFE filter and concentration of the filtrate in vacuo provided a crude amino alcohol (177 mg, not shown), which was used in the next step without further purification.

To a solution of the crude amino alcohol (177 mg, 1.10 mmol, 1 equiv [assumed]) in chloroform (4.0 mL) was added isopentyl nitrite (155 mg, 1.31 mmol, 1.2 equiv) and acetic acid (13 μL, 0.22 mmol, 0.2 equiv) at 25 °C, and the resulting mixture was stirred at 40 °C for 2 h (TLC: 20% ethyl acetate in hexane; ninhydrin). Then, a saturated aqueous solution of sodium hydrogen carbonate (25 mL) was added, and the resulting mixture was extracted with dichloromethane (3 × 20 mL), the organic phase was dried over anhydrous sodium sulfate, the dried solution was filtered, and the filtrate was concentrated in vacuo. The obtained residue was purified by FCC (gradient elution with 12–16% ethyl acetate in hexane) to provide diazo ester **14** as a pale-yellow oil (117 mg, 62%).

Diazo ester **14**: TLC (30% diethyl ether in hexane; ninhydrin): R_f = 0.37. ^{1}H NMR (500 MHz, $CDCl_3$) δ: 4.59 (t, *J* = 6.3 Hz, 1H), 4.24 (q, *J* = 7.1 Hz, 2H), 2.61 (s, 1H), 1.70 (m, 2H), 1.29 (t, *J* = 7.1 Hz, 3H), 1.00 (t, *J* = 7.4 Hz, 3H). ^{13}C{^{1}H} NMR (126 MHz, $CDCl_3$) δ: 166.8, 68.3, 61.1, 27.4, 14.6, 10.1. FTIR (neat), cm^{-1}: 3427, 2970, 2936, 2092, 1672, 1465, 1373, 1293, 1172, 1132, 1045, 1017, 968, 905, 747. HRMS (APCI): Calcd for $[C_7H_{12}N_2O_3+NH_4]^+$: 190.1186, found: 190.1184.

Synthesis of ethyl (*S*)-3-hydroxypentanoate *ent*-6a

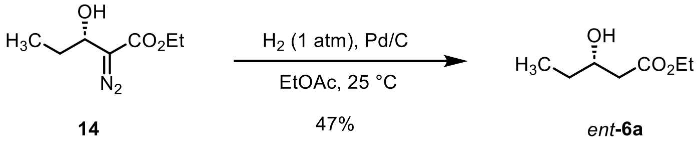

Palladium on charcoal (10% Pd basis; 8 mg) was added to a stirred solution of diazo ester **14** (40 mg, 0.23 mmol, 1 equiv) in ethyl acetate (2.5 mL) at 25 °C. After stirring of the suspension for 10 min, the reaction flask was evacuated, and a double layered balloon filled with hydrogen gas was attached. The mixture was vigorously stirred for 1.5 h at 25 °C (TLC: 20% ethyl acetate in hexane; $KMnO_4$). Filtration through a syringe PTFE filter and concentration of the filtrate in vacuo and subsequent purification by FCC (gradient elution with 2–20% ethyl acetate in hexane) provided alcohol *ent*-**6a** as a colorless oil (16 mg, 47%). NMR data of *ent*-**6a** matched those

obtained for the product of transfer hydrogenation of the corresponding β-keto ester (see below). $[\alpha]_D^{23}$ = +25 (*c* 0.8, $CHCl_3$).

Synthesis of ethyl (*R*)-3-hydroxypentanoate 6a

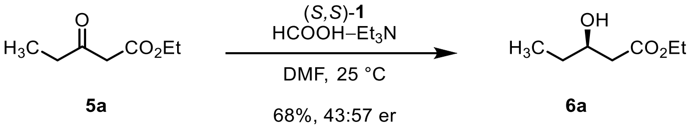

5a **6a**

A solution of (*S,S*)-**1** (7.4 mg, 11.6 μmol, 0.02 equiv) in anhydrous *N,N*-dimethylformamide (1 mL) was evacuated and backfilled with argon (4 cycles). Then, keto ester **5a** (84 mg, 0.58 mmol, 1 equiv) was added as a solution in anhydrous *N,N*-dimethylformamide (0.5 mL), and the mixture was stirred for 5 min in order to obtain a clear solution. Then, argon was bubbled through the solution for 10 min (outlet needle), and a double-layered balloon filled with argon was attached. A mixture of formic acid and triethylamine (5:2 by volume, 0.29 mL) was added, followed by stirring for 23 h at 25 °C (TLC: 25% ethyl acetate in hexane). Then, cold water (20 mL) was added, and the resulting mixture was extracted with ethyl acetate (3 × 25 mL). The organic layer was washed with brine (20 mL), dried over anhydrous sodium sulfate, the dried solution was filtered, and the filtrate was concentrated in vacuo. The obtained residue was purified by FCC (20% ethyl acetate in hexane) to provide alcohol **6a** as a colorless oil (58 mg, 68%). The enantiomeric purity of **6a** was determined by GC analysis (43:57 er).

Alcohol **6a**: $[\alpha]_D^{23}$ = –5 (*c* 0.8, $CHCl_3$). TLC (20% ethyl acetate in hexane; PMA, ninhydrin): R_f = 0.43. ^1H NMR (500 MHz, $CDCl_3$) δ: 4.17 (q, *J* = 7.2 Hz, 2H), 3.92 (m, 1H), 2.93 (s, 1H), 2.50 (dd, *J* = 16.3, 3.2 Hz, 1H), 2.39 (dd, *J* = 16.3, 9.2 Hz, 1H), 1.59–1.44 (m, 2H), 1.27 (t, *J* = 7.2 Hz, 3H), 0.96 (t, *J* = 7.5 Hz, 3H). $^{13}C\{^1H\}$ NMR (126 MHz, $CDCl_3$) δ: 173.2, 69.5, 60.8, 41.0, 29.5, 14.3, 9.9. FTIR (neat), cm^{-1}: 3454, 2962, 2926, 2855, 1734, 1464, 1406, 1373, 1286, 1252, 1179, 1096, 1030, 983. HRMS (APCI): Calcd for $[C_7H_{14}O_3+H]^+$: 147.1013, found: 147.1013.

Enantioselective transfer hydrogenation of trifluorethyl substrate 3b

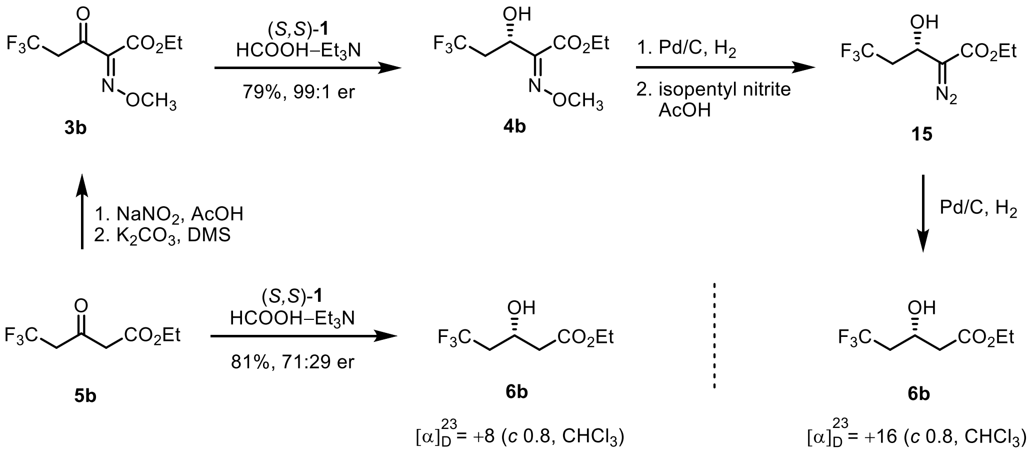

Synthesis of ethyl (*S*)-5,5,5-trifluoro-3-hydroxy-2-(methoxyimino)pentanoate 4b

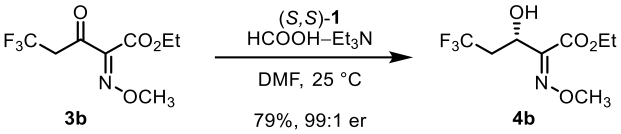

A solution of (*S,S*)-**1** (18.5 mg, 29.1 μmol, 0.02 equiv) in anhydrous *N,N*-dimethylformamide (1.0 mL) was evacuated and backfilled with argon (4 cycles). Then, α-methoxyimino ester **3b** (351 mg, 1.46 mmol, 1 equiv, 16:1 ratio of *Z*:*E* isomers) was added as a solution in anhydrous *N,N*-dimethylformamide (0.7 mL), and the mixture was stirred for 5 min in order to obtain a clear solution. Then, argon was bubbled through the solution for 15 min (outlet needle), and a double-layered balloon filled with argon was attached. A mixture of formic acid and triethylamine (5:2 by volume, 0.73 mL) was added, followed by stirring for 24 h at 25 °C (TLC: 20% ethyl acetate in hexane, UV, PMA). Then, ice-cold water (30 mL) was added, and the resulting mixture was extracted with ethyl acetate (3 × 30 mL). The combined organic phases were washed with brine (25 mL), dried over anhydrous sodium sulfate, the dried solution was filtered, and the filtrate was concentrated in vacuo. The obtained residue was purified by FCC (20% ethyl acetate in hexane) to provide alcohol **4b** as a colorless oil (279 mg, 79%, 11:1 ratio of *Z:E* isomers). The enantiomeric purity of **4b** was determined by HPLC (99:1 er).

Alcohol **4b**: TLC (20% ethyl acetate in hexane; PMA): R_f = 0.3. ^{1}H NMR (500 MHz, $CDCl_3$, 11:1 mixture of *Z*:*E* isomers; only signals corresponding to the major isomer are listed) δ: 4.83 (d, *J* = 8.8 Hz, 1H), 4.35 (q, *J* = 6.9 Hz, 2H), 3.94 (s, 3H), 2.74 – 2.47 (m, 2H), 1.35 (t, *J* = 7.1 Hz, 3H). $^{13}C\{^1H\}$ NMR (126 MHz, $CDCl_3$, 11:1 mixture of *Z*:*E* isomers; only signals corresponding to the major isomer are listed) δ: 161.5, 149.4, 125.9 (q, *J* = 277.1 Hz), 65.7, 63.4, 62.3, 38.8 (q, *J* = 28.0 Hz), 14.2. FTIR (neat), cm^{-1}: 3481, 2986, 2946, 1727, 1373, 1311, 1250, 1202, 1134, 1035. HRMS (APCI): Calcd for $[C_8H_{12}F_3NO_4+H]^+$: 244.0791, found: 244.0790.

Synthesis of ethyl (*S*)-2-diazo-5,5,5-trifluoro-3-hydroxypentanoate 15

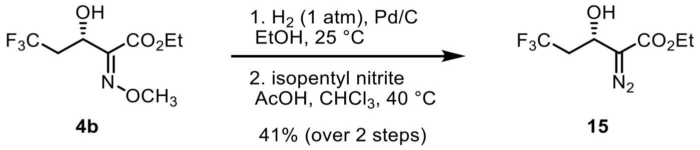

Palladium on charcoal (10% Pd basis; 52 mg) was added to a stirred solution of alcohol **4b** (260 mg, 1.07 mmol, 1 equiv) in ethanol (6.0 mL) at 25 °C. After stirring of the suspension for 10 min, the reaction flask was evacuated, and a double layered balloon filled with hydrogen gas was attached. The mixture was vigorously stirred for 2 days at 25 °C (TLC: 30% ethyl acetate in hexane; ninhydrin). Filtration through a syringe PTFE filter and concentration of the filtrate in vacuo provided a crude amino alcohol (230 mg, not shown), which was used in the next step without further purification.

To a solution of the crude amino alcohol (230 mg, 1.07 mmol, 1 equiv [assumed]) in chloroform (4.5 mL) was added isopentyl nitrite (150 mg, 1.28 mmol, 1.2 equiv) and acetic acid (12 μL, 0.21 mmol, 0.2 equiv) at 25 °C, and the resulting mixture was stirred at 40 °C for 1 h (TLC: 30% ethyl acetate in hexane; ninhydrin). Then, a saturated aqueous solution of sodium hydrogen carbonate (25 mL) was added, and the resulting mixture was extracted with dichloromethane (3 × 25 mL), the organic phase was dried over anhydrous sodium sulfate, the dried solution was

filtered, and the filtrate was concentrated in vacuo. The obtained residue was purified by FCC (15% diethyl ether in hexane) to provide diazo ester **15** as a pale-yellow oil (100 mg, 41%).

Diazo ester **15**: TLC (30% ethyl acetate in hexane; ninhydrin): R_f = 0.5. ^{1}H NMR (500 MHz, $CDCl_3$) δ: 5.00 – 4.97 (m, 1H), 4.26 (q, *J* = 7.1 Hz, 2H), 2.85 (s, 1H), 2.71–2.48 (m, 2H), 1.30 (t, *J* = 7.1 Hz, 3H). $^{13}C\{^1H\}$ NMR (126 MHz, $CDCl_3$) δ: 165.8, 125.3 (q, *J* = 277.0 Hz), 61.5, 39.2 (q, *J* = 27.9 Hz), 14.5. FTIR (neat), cm^{-1}: 3437, 2988, 2098, 1668, 1398, 1375, 1295, 1239, 1096, 1042, 1017, 849, 745, 642. HRMS (APCI): Calcd for $[C_7H_9F_3N_2O_3+Cl]^-$: 261.0259, found: 261.0262.

Synthesis of ethyl (*R*)-5,5,5-trifluoro-3-hydroxypentanoate 6b

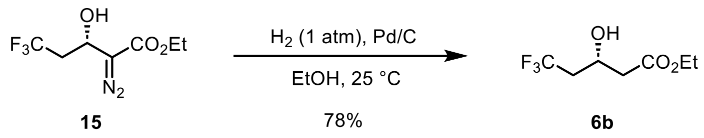

Palladium on charcoal (10% Pd basis; 9 mg) was added to a stirred solution of diazo ester **15** (45 mg, 0.20 mmol, 1 equiv) in ethanol (3 mL) at 25 °C. After stirring of the suspension for 10 min, the reaction flask was evacuated, and a double layered balloon filled with hydrogen gas was attached. The mixture was vigorously stirred for 50 min at 25 °C (TLC: 30% ethyl acetate in hexane; $KMnO_4$). Filtration through a syringe PTFE filter and concentration of the filtrate in vacuo to provide crude mixture, which was then purified by FCC (gradient elution with 10–15% ether in hexane). Alcohol **6b** was obtained as a colorless oil (31 mg, 78%). NMR data of **6b** matched those obtained for the product of transfer hydrogenation of the corresponding β-keto ester (see below). $[\alpha]_D^{23}$ = +16 (*c* 0.8, $CHCl_3$).

Synthesis of ethyl (*R*)-5,5,5-trifluoro-3-hydroxypentanoate 6b

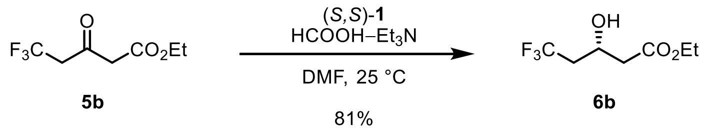

A solution of (*S,S*)-**1** (5.2 mg, 8.2 μmol, 0.02 equiv) in anhydrous *N,N*-dimethylformamide (1 mL) was evacuated and backfilled with argon (4 cycles). Then, keto ester **5b** (81 mg, 0.41 mmol, 1 equiv) was added as a solution in anhydrous *N,N*-dimethylformamide (0.5 mL), and the mixture was stirred for 5 min in order to obtain a clear solution. Then, argon was bubbled through the solution for 10 min (outlet needle), and a double-layered balloon filled with argon was attached. A mixture of formic acid and triethylamine (5:2 by volume, 0.21 mL) was added, followed by stirring for 3.5 h at 25 °C (TLC: 20% ethyl acetate in hexane, $KMnO_4$). Then, cold water (20 mL) was added, and the resulting mixture was extracted with ethyl acetate (3 × 20 mL). The organic layer was washed with brine (15 mL), dried over anhydrous sodium sulfate, the dried solution was filtered, and the filtrate was concentrated in vacuo. The obtained residue (containing starting material and product) was purified by FCC (gradient elution with 15–30% ether in hexane) to provide alcohol **6b** as a colorless oil (66 mg, 81%).

Alcohol **6b**: $[\alpha]_D^{23}$ = +8 (*c* 0.8, $CHCl_3$). TLC (20% ethyl acetate in hexane; $KMnO_4$): R_f = 0.25. ^1H NMR (500 MHz, $CDCl_3$) δ: 4.44 – 4.33 (m, 1H), 4.20 (q, *J* = 7.1 Hz, 2H), 3.22–3.19 (m, 1H), 2.64–2.50 (m, 2H), 2.48–2.23 (m, 2H), 1.29 (t, *J* = 7.1 Hz, 3H). $^{13}C\{^1H\}$ NMR (126 MHz, $CDCl_3$) δ: 172.1, 126.0 (q, *J* = 276.9 Hz), 62.9, 61.2, 40.3 (q, *J* = 27.6 Hz), 14.3. FTIR (neat), cm^{-1}: 3470, 2985, 2925, 2854, 1722, 1378, 1254, 1156, 1146, 1116, 1026, 874.

Conversion of alcohol 6b to 4-nitrobenzoate 16

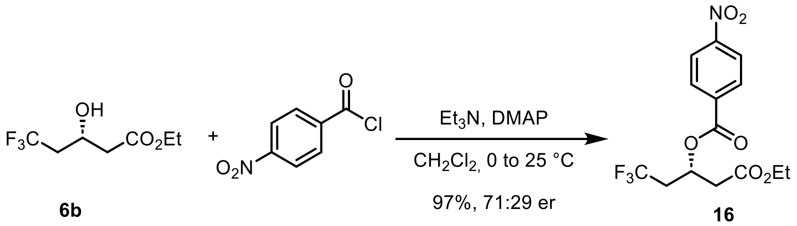

6b **16**

Triethylamine (33 µL, 0.24 mmol, 2.0 equiv) and 4-dimethylaminopyridine (1.5 mg, 12.2 µmol, 0.1 equiv) were added to a stirred solution of alcohol **6b** (24 mg, 0.12 mmol, 1 equiv) in anhydrous dichloromethane (1.0 mL) at 0 °C. After 5 min of stirring at 0 °C, 4-nitrobenzoyl chloride (44.5 mg, 0.24 mmol, 2.0 equiv) was slowly added as a solution in anhydrous dichloromethane (0.3 mL) at 0 °C. The resulting mixture was allowed to warm to 25 °C and stirred at this temperature for 16 h (TLC: 20% ethyl acetate in hexane). Then, water (10 mL) and dichloromethane (8 mL) were added. The mixture was extracted with dichloromethane (3 × 10 mL), the combined organic phases were washed with brine (10 mL), dried over anhydrous magnesium sulfate, and concentrated in vacuo. The obtained residue was purified by FCC (gradient elution with 10–12% ethyl acetate in hexane) to provide 4-nitrobenzoyl ester **16** as a colorless oil (41 mg, 97%). The enantiomeric purity of **16** was determined by HPLC (71:29 er).

4-Nitrobenzoyl ester **16**: TLC (20% ethyl acetate in hexane): R_f = 0.55. ^1H NMR (500 MHz, $CDCl_3$) δ: 8.38–8.25 (m, 2H), 8.24–8.14 (m, 2H), 5.76 (dtd, *J* = 7.5, 6.3, 4.4 Hz, 1H), 4.16 (q, *J* = 7.1 Hz, 2H), 2.86 (qd, *J* = 16.2, 6.3 Hz, 2H), 2.80–2.65 (m, 2H), 1.23 (t, *J* = 7.1 Hz, 3H). $^{13}C\{^1H\}$ NMR (126 MHz, $CDCl_3$) δ: 169.2, 163.6, 151.0, 135.0, 131.0, 125.5 (q, *J* = 277.3 Hz), 123.8, 66.2, 66.2, 61.3, 38.5, 37.6 (q, *J* = 28.9 Hz), 14.2. FTIR (neat), cm^{-1}: 2955, 2921, 2852, 1729, 1608, 1527, 1401, 1349, 1318, 1273, 1251, 1192, 1096, 1014, 873, 833, 717.

Enantioselective transfer hydrogenation of methoxymethyl substrate 3c

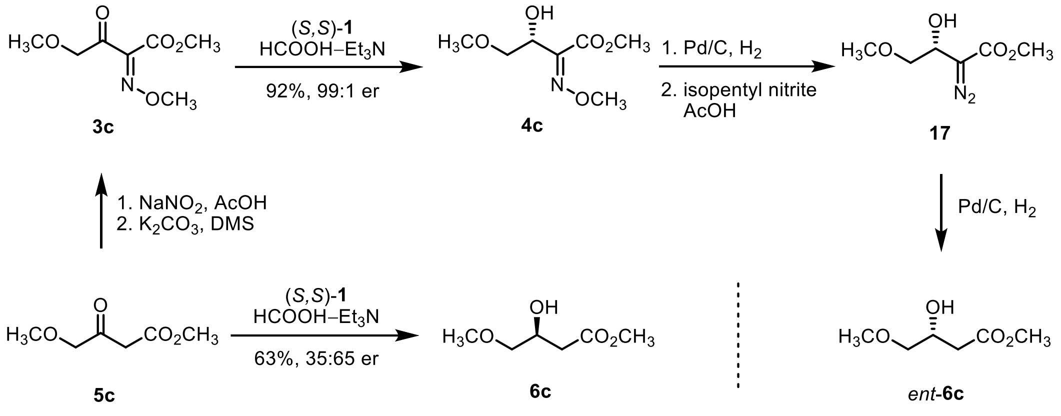

$[\alpha]_D^{23}$ = –4 (*c* 0.9, $CHCl_3$) $[\alpha]_D^{23}$ = +16 (*c* 0.8, $CHCl_3$)

Synthesis of methyl 4-methoxy-2-(methoxyimino)-3-oxobutanoate 3c

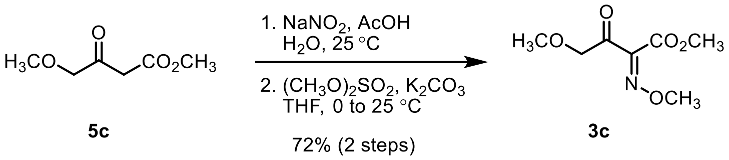

A solution of sodium nitrite (1.13 g, 16.4 mmol, 1.2 equiv) in water (2.5 mL) was added dropwise to a solution of methyl 4-methoxy-3-oxobutanoate **5c** (2.00 g, 13.7 mmol, 1 equiv) in acetic acid (4.5 mL) over 40 min at 0 °C. The resulting mixture was stirred at this temperature for 1.5 h (TLC: 30% ethyl acetate in hexane; UV, PMA). Then, the mixture was poured into brine (80 mL) and extracted with ether (3 × 40 mL). The organic extracts were combined and washed with a saturated aqueous solution of sodium hydrogen carbonate (120 mL) to reach pH ~ 7, and the aqueous phase was extracted again with ether (3 × 40 mL). All organic extracts were combined, dried over anhydrous sodium sulfate, and filtered. The filtrate was concentrated under reduced pressure to yield the crude α-hydroxyimino ester (2.04 g, not shown), which was used in the next step without further purification.

Potassium carbonate (1.79 g, 13.0 mmol, 1.2 equiv) was added to a stirred solution of crude α-hydroxyimino ester (2.04 g, 10.8 mmol, 1 equiv) in anhydrous tetrahydrofuran (35 mL) at 0 °C. After 30 min of stirring at 0 °C, dimethyl sulfate (1.03 mL, 10.8 mmol, 1.0 equiv) was added at 0 °C, and the resulting solution was allowed to warm to room temperature and stirred at this temperature for 60 h (TLC: 30% ethyl acetate in hexane). The reaction mixture was filtered, ice-cold brine (80 mL) was added, and the resulting mixture was extracted with dichloromethane (3 × 80 mL). The combined organic phases were dried over anhydrous sodium sulfate, the dried solution was filtered, and the filtrate was concentrated in vacuo. The obtained residue was purified by FCC (gradient elution with 18–22% ethyl acetate in hexane) to provide α-methoxyimino ester **3c** as a colorless oil (2.01 g, 72%, 12:1 mixture of *Z*:*E* isomers).

α-Methoxyimino ester **3c**: TLC (30% ethyl acetate in hexane): R_f= 0.55. ^1H NMR (500 MHz, $CDCl_3$, 12:1 mixture of *Z*:*E* isomers; only signals corresponding to the major isomer are listed) δ: 4.52 (s, 2H), 4.08 (s, 3H), 3.86 (s, 3H), 3.44 (s, 3H). $^{13}C\{^1H\}$ NMR (126 MHz, $CDCl_3$, 12:1 mixture of *Z*:*E* isomers; only signals corresponding to the major isomer are listed) δ: 190.7, 160.7, 148.2, 73.7,

64.7, 59.7, 52.9. FTIR (neat), cm^{-1}: 3006, 2991, 2945, 1740, 1707, 1601, 1478, 1445, 1406, 1361, 1331, 1278, 1219, 1187, 1151, 1036, 1005, 926, 879, 814, 795. HRMS (APCI): Calcd for $[C_7H_{11}NO_5+H]^+$: 190.0710, found: 190.0709.

Synthesis of methyl (*R*)-3-hydroxy-4-methoxy-2-(methoxyimino)butanoate 4c

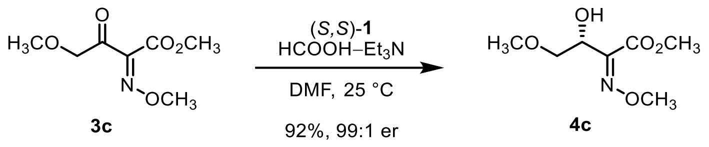

A solution of (*S,S*)-**1** (25.4 mg, 40.0 μmol, 0.02 equiv) in anhydrous *N,N*-dimethylformamide (2.0 mL) was evacuated and backfilled with argon (4 cycles). Then, α-methoxyimino ester **3c** (406 mg, 2.15 mmol, 1 equiv, 12:1 mixture of *Z*:*E* isomers) was added as a solution in anhydrous *N,N*-dimethylformamide (0.7 mL), and the mixture was stirred for 5 min in order to obtain a clear solution. Then, argon was bubbled through the solution for 10 min (outlet needle), and a double-layered balloon filled with argon was attached. A mixture of formic acid and triethylamine (5:2 by volume, 1.0 mL) was added, followed by stirring for 16 h at 25 °C (TLC: 30% ethyl acetate in hexane). Then, ice-cold water (30 mL) was added, and the resulting mixture was extracted with ethyl acetate (3 × 30 mL). The combined organic phases were washed with brine (25 mL), dried over anhydrous sodium sulfate, the dried solution was filtered, and the filtrate was concentrated in vacuo. The obtained residue was purified by FCC (gradient elution with 20–25% ethyl acetate in hexane) to provide alcohol **4c** as a pale-yellow oil (375 mg, 92%, 12:1 mixture of *Z*:*E* isomers). The enantiomeric purity of **4c** was determined by HPLC (99:1 er).

Alcohol **4c**: TLC (30% ethyl acetate in hexane): R_f= 0.4. ^{1}H NMR (500 MHz, $CDCl_3$, 12:1 mixture of *Z*:*E* isomers; only signals corresponding to the major isomer are listed) δ: 4.59 (q, *J* = 5.3 Hz, 1H), 3.91 (s, 3H), 3.84 (s, 3H), 3.60 (d, *J* = 5.4 Hz, 2H), 3.39 (s, 3H), 2.91 (d, *J* = 5.3 Hz, 1H). $^{13}C\{^1H\}$ NMR (126 MHz, $CDCl_3$, 12:1 mixture of *Z*:*E* isomers; only signals corresponding to the major isomer are listed) δ: 162.6, 150.0, 74.1, 69.6, 63.1, 59.4, 52.5. FTIR (neat), cm^{-1}: 3430, 2941, 2903, 2826, 1732, 1628, 1436, 1303, 1200, 1155, 1119, 1031, 958, 920, 887, 809. HRMS (APCI): Calcd for $[C_7H_{13}NO_5+H]^+$: 192.0866, found: 192.0861.

Synthesis of methyl (*R*)-2-diazo-3-hydroxy-4-methoxybutanoate 17

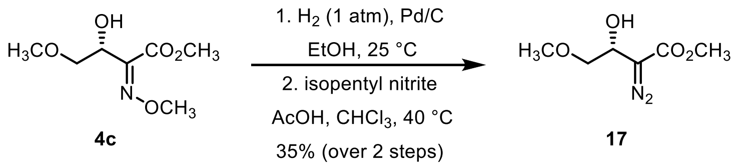

Palladium on charcoal (10% Pd basis; 74 mg) was added to a stirred solution of alcohol **4c** (369 mg, 1.93 mmol, 1 equiv) in anhydrous ethanol (10 mL) at 25 °C. After stirring of the suspension for 10 min, the reaction flask was evacuated, and a double layered balloon filled with hydrogen gas was attached. The mixture was vigorously stirred for 2.5 days at 25 °C (TLC: 35% ethyl acetate in hexane; ninhydrin). Filtration through a syringe PTFE filter and concentration of

the filtrate in vacuo provided a crude amino alcohol (340 mg, not shown), which was used in the next step without further purification.

To a solution of the crude amino alcohol (340 mg, 2.09 mmol, 1 equiv [assumed]) in chloroform (9.0 mL) was added isopentyl nitrite (0.31 mL, 2.31 mmol, 1.1 equiv) and acetic acid (22 μL, 0.38 mmol, 0.2 equiv) at 25 °C, and the resulting mixture was stirred at 40 °C for 2 h (TLC: 25% ethyl acetate in hexane; ninhydrin). Then, a saturated aqueous solution of sodium hydrogen carbonate (20 mL) was added, and the resulting mixture was extracted with dichloromethane (3 × 20 mL), the organic phase was dried over anhydrous sodium sulfate, the dried solution was filtered, and the filtrate was concentrated in vacuo. The obtained residue was purified by FCC (gradient elution with 30–35% diethyl ether in hexane) to provide diazo ester **17** as a pale-yellow oil (110 mg, ca. 35%; contains about 10% of undefined impurities).

Diazo ester **17**: TLC (40% ethyl acetate in hexane; ninhydrin): R_f = 0.35. ^{1}H NMR (500 MHz, $CDCl_3$) δ: 4.77 (dt, *J* = 5.8, 4.4 Hz, 1H), 3.77 (s, 3H), 3.63–3.53 (m, 2H), 3.40 (s, 3H), 2.91 (d, *J* = 4.8 Hz, 1H). $^{13}C\{^1H\}$ NMR (126 MHz, $CDCl_3$) δ: 166.7, 74.1, 65.2, 59.3, 52.1. FTIR (neat), cm^{-1}: 3441, 2953, 2931, 2897, 2098, 1747, 1688, 1438, 1349, 1294, 1194, 1118, 1074, 1030, 966.

Synthesis of methyl (*R*)-3-hydroxy-4-methoxybutanoate *ent*-6c

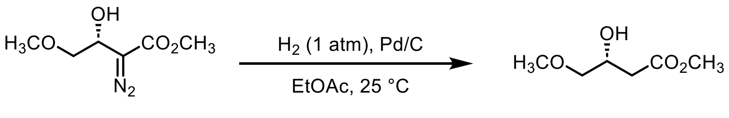

17   *ent*-**6c**

Palladium on charcoal (10% Pd basis; 18 mg) was added to a stirred solution of diazo ester **17** (90 mg, 0.52 mmol, 1 equiv) in anhydrous ethanol (4.5 mL) at 25 °C. After stirring of the suspension for 10 min, the reaction flask was evacuated, and a double layered balloon filled with hydrogen gas was attached. The mixture was vigorously stirred for 1.5 h at 25 °C (TLC: 30% ethyl acetate in hexane; PMA, ninhydrin). Filtration through a syringe PTFE filter and concentration of the filtrate in vacuo. The obtained residue was purified by FCC (gradient elution with 25–30% ethyl acetate in hexane) to provide alcohol *ent*-**6c** as a pale-yellow oil (32 mg, 42%). NMR data of *ent*-**6c** matched those obtained for the product of transfer hydrogenation of the corresponding β-keto ester (see below). $[\alpha]_D^{23}$ = +16 (*c* 0.8, $CHCl_3$).

Synthesis of methyl (*S*)-3-hydroxy-4-methoxybutanoate 6c

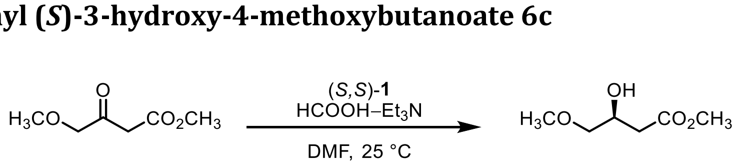

5c   **6c**

A solution of (*S*,*S*)-**1** (12.7 mg, 20.0 μmol, 0.02 equiv) in anhydrous *N*,*N*-dimethylformamide (1.0 mL) was evacuated and backfilled with argon (4 cycles). Then, methyl 4-methoxy-3-oxobutanoate **5c** (146 mg, 1.00 mmol, 1 equiv) was added as a solution in anhydrous *N*,*N*-dimethylformamide (0.4 mL), and the mixture was stirred for 5 min in order to obtain a clear solution. Then, argon was bubbled through the solution for 10 min (outlet needle), and a double-layered balloon filled with argon was attached. A mixture of formic acid and triethylamine (5:2 by

volume, 0.5 mL) was added, followed by stirring for 16 h at 25 °C (TLC: 25% ethyl acetate in hexane). Then, ice-cold water (20 mL) was added, and the resulting mixture was extracted with ethyl acetate (3 × 20 mL). The combined organic phases were washed with brine (20 mL), dried over anhydrous sodium sulfate, the dried solution was filtered, and the filtrate was concentrated in vacuo. The obtained residue was purified by FCC (gradient elution with 20–25% ethyl acetate in hexane) to provide alcohol **6c** as a pale-yellow oil (93 mg, 63%). The enantiomeric purity of **6c** was determined by GC analysis (35:65 er).

Alcohol **6c**: $[\alpha]_D^{23}$ = −4 (*c* 0.9, $CHCl_3$). TLC (35% ethyl acetate in hexane; ninhydrin): R_f = 0.3. ^1H NMR (500 MHz, $CDCl_3$) δ: 4.20 (p, *J* = 5.8 Hz, 1H), 3.70 (s, 3H), 3.49–3.32 (m, 5H), 2.91 (s, 1H), 2.52 (d, *J* = 6.3 Hz, 2H). $^{13}C\{^1H\}$ NMR (126 MHz, $CDCl_3$) δ: 172.6, 75.8, 67.2, 59.3, 51.9, 38.1. FTIR (neat), cm^{-1}: 3448, 2952, 2926, 2895, 2829, 1731, 1438, 1360, 1257, 1195, 1168, 1123, 1078, 1001, 965, 885, 718. HRMS (APCI): Calcd for $[C_6H_{12}O_4+H]^+$: 149.0808, found: 149.0805.

Enantioselective transfer hydrogenation of chloromethyl substrate *Z*-3d

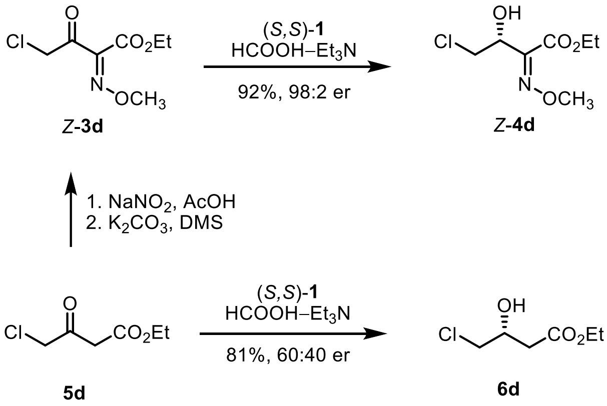

Synthesis of ethyl 4-chloro-2-(methoxyimino)-3-oxobutanoate *Z*-3d

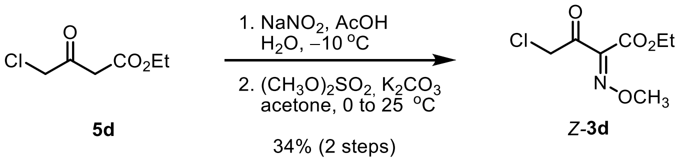

A solution of sodium nitrite (805 mg, 11.67 mmol, 1.2 equiv) in water (4 mL) was added dropwise to a solution of ethyl 4-chloro-3-oxobutanoate **5d** (1.60 g, 9.7 mmol, 1 equiv) in acetic acid (6.0 mL) over 40 min at −10 °C. The resulting mixture was stirred at this temperature for 2 h (TLC: 30% ethyl acetate in hexane; UV, PMA). Then, the mixture was poured into cold water (80 mL) and extracted with ethyl acetate (3 × 40 mL). The organic extracts were combined and washed with a saturated aqueous solution of sodium hydrogen carbonate (120 mL) to reach pH ~ 7, and the aqueous phase was extracted again with ethyl acetate (3 × 40 mL). All organic extracts were combined, dried over anhydrous sodium sulfate, and filtered. The filtrate was concentrated under reduced pressure to yield the crude α-hydroxyimino ester (1.40 g, not shown), which was used in the next step without further purification.

Potassium carbonate (1.30 g, 9.4 mmol, 1.3 equiv) was added to a stirred solution of crude α-hydroxyimino ester (see above, 1.40 g, 7.3 mmol, 1 equiv) in anhydrous acetone (15 mL) at 0

°C. After 30 min of stirring at 0 °C, dimethyl sulfate (0.69 mL, 7.3 mmol, 1.0 equiv) was added at 0 °C, and the resulting solution was allowed to warm to room temperature and stirred at this temperature for 6 h (TLC: 20% ethyl acetate in hexane). The reaction mixture was filtered, ice-cold brine (80 mL) was added, and the resulting mixture was extracted with ethyl acetate (3 × 80 mL). The combined organic phases were washed with brine (80 mL), dried over anhydrous sodium sulfate, the dried solution was filtered, and the filtrate was concentrated in vacuo. The obtained residue was purified by FCC (gradient elution with 12–16% ethyl acetate in hexane) to provide α-methoxyimino ester *Z*-**3d** as a pale-yellow oil (690 mg, 34%).

α-Methoxyimino ester *Z*-**3d**: TLC (20% ethyl acetate in hexane): R_f = 0.5. ^{1}H NMR (500 MHz, $CDCl_3$) δ: 4.57 (s, 2H), 4.35 (q, *J* = 7.1 Hz, 2H), 4.12 (s, 3H), 1.33 (t, *J* = 7.1 Hz, 3H). ^{13}C{^{1}H} NMR (126 MHz, $CDCl_3$) δ: 185.8, 159.9, 148.3, 65.0, 62.6, 44.9, 14.1. FTIR (neat), cm^{-1}: 3007, 2992, 2947, 1736, 1709, 1598, 1472, 1443, 1405, 1367, 1339, 1279, 1209, 1180, 1153, 1034, 1009, 925, 877, 856, 813, 794. HRMS (APCI): Calcd for $[C_7H_{10}ClNO_4+H]^+$: 208.0371, found: 208.0373.

Synthesis of ethyl (*R*)-4-chloro-3-hydroxy-2-(methoxyimino)butanoate *Z*-4d

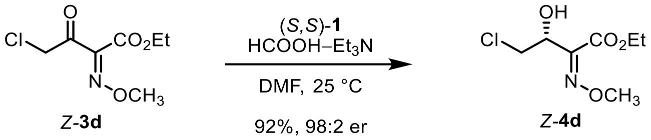

A solution of (*S,S*)-**1** (21.5 mg, 33.8 μmol, 0.02 equiv) in anhydrous *N,N*-dimethylformamide (1.0 mL) was evacuated and backfilled with argon (4 cycles). Then, α-methoxyimino ester *Z*-**3d** (350 mg, 1.69 mmol, 1 equiv, *Z* isomer) was added as a solution in anhydrous *N,N*-dimethylformamide (0.4 mL), and the mixture was stirred for 5 min in order to obtain a clear solution. Then, argon was bubbled through the solution for 10 min (outlet needle), and a double-layered balloon filled with argon was attached. A mixture of formic acid and triethylamine (5:2 by volume, 0.85 mL) was added, followed by stirring for 16 h at 25 °C (TLC: 25% ethyl acetate in hexane). Then, ice-cold water (20 mL) was added, and the resulting mixture was extracted with ethyl acetate (3 × 25 mL). The combined organic phases were washed with brine (20 mL), dried over anhydrous sodium sulfate, the dried solution was filtered, and the filtrate was concentrated in vacuo. The obtained residue was purified by FCC (gradient elution with 15–20% ethyl acetate in hexane) to provide alcohol *Z*-**4d** as a pale-yellow oil (325 mg, 92%, *Z* isomer). The enantiomeric purity of *Z*-**4d** was determined by HPLC (98:2 er).

Alcohol *Z*-**4d**: $[\alpha]_D^{23}$ = −11 (*c* 0.85, $CHCl_3$). TLC (20% ethyl acetate in hexane): R_f = 0.55. ^{1}H NMR (500 MHz, $CDCl_3$) δ: 4.72–4.63 (m, 1H), 4.33 (q, *J* = 7.1 Hz, 2H), 3.94 (s, 3H), 3.81 (dd, *J* = 11.5, 4.2 Hz, 1H), 3.75 (dd, *J* = 11.5, 6.7 Hz, 1H), 3.00 (d, *J* = 5.9 Hz, 1H), 1.34 (t, *J* = 7.1 Hz, 3H). ^{13}C{^{1}H} NMR (126 MHz, $CDCl_3$) δ: 161.5, 148.8, 70.6, 63.4, 62.2, 46.9, 14.2. FTIR (neat), cm^{-1}: 3447, 2954, 2928, 2892, 2825, 1730, 1432, 1368, 1251, 1191, 1165, 1121, 1079, 1011, 968, 881, 719. HRMS (APCI): Calcd for $[C_7H_{12}ClNO_4+H]^+$: 210.0528, found: 210.0527.

Synthesis of ethyl 4-chloro-3-hydroxybutanoate 6d

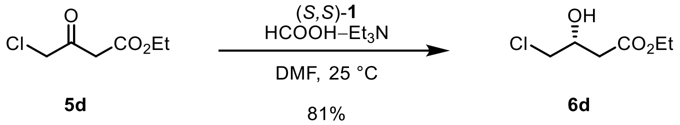

5d **6d**

A solution of (*S,S*)-**1** (16.2 mg, 25.5 µmol, 0.02 equiv) in anhydrous *N,N*-dimethylformamide (1.0 mL) was evacuated and backfilled with argon (4 cycles). Then, ethyl 4-chloro-3-oxobutanoate **5d** (210 mg, 1.28 mmol, 1 equiv) was added as a solution in anhydrous *N,N*-dimethylformamide (0.4 mL), and the mixture was stirred for 5 min in order to obtain a clear solution. Then, argon was bubbled through the solution for 10 min (outlet needle), and a double-layered balloon filled with argon was attached. A mixture of formic acid and triethylamine (5:2 by volume, 0.65 mL) was added, followed by stirring for 16 h at 25 °C (TLC: 25% ethyl acetate in hexane). Then, ice-cold water (25 mL) was added, and the resulting mixture was extracted with ethyl acetate (3 × 25 mL). The combined organic phases were washed with brine (20 mL), dried over anhydrous sodium sulfate, the dried solution was filtered, and the filtrate was concentrated in vacuo. The obtained residue was purified by FCC (gradient elution with 15–20% ethyl acetate in hexane) to provide alcohol **6d** as a pale-yellow oil (172 mg, 81%).

Alcohol **6d**: $[\alpha]_D^{23}$ = +6 (*c* 1.0, $CHCl_3$), [Lit.[2i] $[\alpha]_D^{21}$ = +20.9 (*c* 7.71, $CHCl_3$)]. TLC (20% ethyl acetate in hexane; ninhydrin): R_f = 0.4. ^1H NMR (300 MHz, $CDCl_3$) δ: 4.32–4.11 (m, 3H), 3.65–3.55 (m, 2H), 3.15 (d, *J* = 5.0 Hz, 1H), 2.80–2.52 (m, 2H), 1.28 (t, *J* = 7.2 Hz, 3H). $^{13}C\{^1H\}$ NMR (126 MHz, $CDCl_3$) δ: 171.9, 68.1, 61.1, 48.3, 38.6 (d, *J* = 3.5 Hz), 14.3. FTIR (neat), cm^{-1}: 3466, 2983, 2942, 2905, 1723, 1628, 1444, 1370, 1301, 1244, 1200, 1143, 1090, 1031, 887, 858, 763. HRMS (APCI): Calcd for $[C_6H_{11}ClO_3+H]^+$: 167.0469, found: 167.0472.

Conversion of alcohol 6d to 4-nitrobenzoate 18

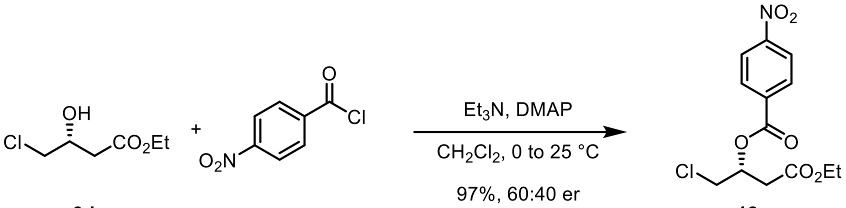

6d **18**

Triethylamine (84 µL, 0.60 mmol, 2.0 equiv) and 4-dimethylaminopyridine (3.7 mg, 30.3 µmol, 0.1 equiv) were added to a stirred solution of alcohol **6d** (50 mg, 0.30 mmol, 1 equiv) in anhydrous dichloromethane (1.5 mL) at 0 °C. After 5 min of stirring at 0 °C, 4-nitrobenzoyl chloride (112 mg, 0.60 mmol, 2.0 equiv) was slowly added as a solution in anhydrous dichloromethane (0.5 mL) at 0 °C. The resulting mixture was allowed to warm to 25 °C and stirred at this temperature for 16 h (TLC: 20% ethyl acetate in hexane). Then, water (10 mL) and dichloromethane (8 mL) were added. The mixture was extracted with dichloromethane (3 × 10 mL), the combined organic phases were washed with brine (10 mL), dried over anhydrous magnesium sulfate, and concentrated in vacuo. The obtained residue was purified by FCC (gradient elution with 10–12% ethyl acetate in hexane) to provide 4-nitrobenzoyl ester **18** as a colorless oil (92 mg, 97%). The enantiomeric purity of **18** was determined by HPLC (60:40 er).

4-Nitrobenzoyl ester **18**: TLC (20% ethyl acetate in hexane): R_f = 0.4. ^1H NMR (500 MHz, $CDCl_3$) δ: 8.34–8.25 (m, 2H), 8.24–8.17 (m, 2H), 5.68 (m, 1H), 4.15 (q, *J* = 7.2 Hz, 2H), 3.90 (dd, *J* = 12.0, 4.6 Hz, 1H), 3.85 (dd, *J* = 12.0, 4.6 Hz, 1H), 2.96–2.87 (m, 2H), 1.23 (t, *J* = 7.1 Hz, 3H). $^{13}C\{^1H\}$ NMR (126

MHz, $CDCl_3$) δ: 169.5, 163.8, 151.0, 135.1, 131.1, 123.8, 70.9, 61.3, 45.1, 36.5, 14.3. HRMS (APCI): Calcd for $[C_{13}H_{14}ClNO_6+H]^+$: 316.0582, found: 316.0579.

Enantioselective transfer hydrogenation of difluoromethyl substrate 3e

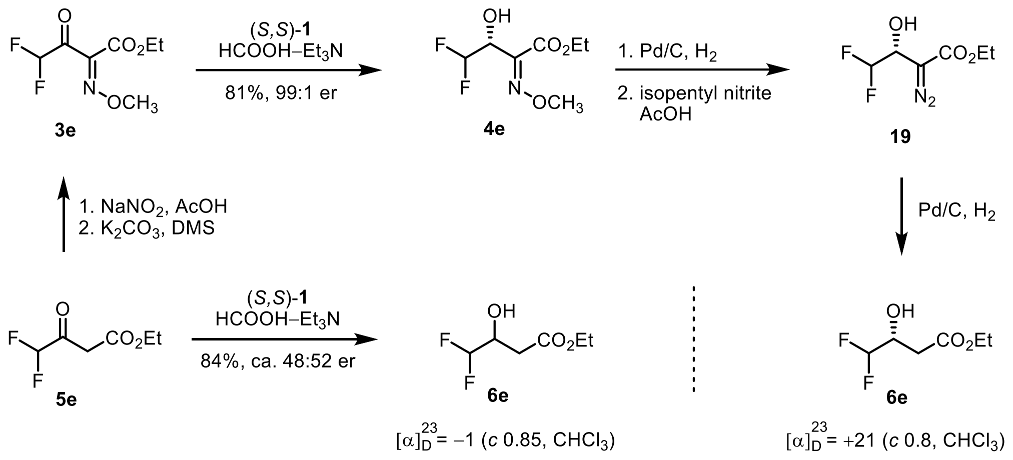

Synthesis of ethyl (*R*)-4,4-difluoro-3-hydroxy-2-(methoxyimino)butanoate 4e

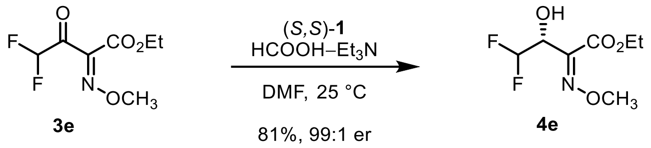

A solution of (*S,S*)-**1** (15.3 mg, 24.1 μmol, 0.02 equiv) in anhydrous *N,N*-dimethylformamide (1.0 mL) was evacuated and backfilled with argon (4 cycles). Then, α-methoxyimino ester **3e**[3] (252 mg, 1.21 mmol, 1 equiv, 10:1 ratio of *Z*:*E* isomers) was added as a solution in anhydrous *N,N*-dimethylformamide (0.5 mL), and the mixture was stirred for 5 min to obtain a clear solution. Then, argon was bubbled through the solution for 15 min (outlet needle), and a double-layered balloon filled with argon was attached. A mixture of formic acid and triethylamine (5:2 by volume, 0.61 mL) was added, followed by stirring for 17 h at 25 °C (TLC: 30% ethyl acetate in hexane, UV, PMA). Then, ice-cold water (30 mL) was added, and the resulting mixture was extracted with ethyl acetate (3 × 30 mL). The combined organic phases were washed

with brine (30 mL), dried over anhydrous sodium sulfate, the dried solution was filtered, and the filtrate was concentrated in vacuo. The obtained residue was purified by FCC (30% ethyl acetate in hexane) to provide alcohol **4e** as a yellow oil (205 mg, 81%, 21:1 mixture of *Z*:*E* isomers). The enantiomeric purity of **4e** was determined by HPLC (99:1 er).

Alcohol **4e**: TLC (30% ethyl acetate in hexane, UV, PMA): R_f = 0.54. ^{1}H NMR (500 MHz, $CDCl_3$, 21:1 mixture of *Z*:*E* isomers; only signals corresponding to the major isomer are listed) δ: 5.91 (td, *J* = 55.0, 4.0 Hz, 1H), 4.64 (td, *J* = 10.4, 3.9 Hz, 1H), 4.34 (q, *J* = 7.1 Hz, 2H), 3.99 (s, 3H), 3.10 (d, *J* = 6.9 Hz, 1H), 1.34 (t, *J* = 7.1 Hz, 3H). ^{13}C{^{1}H} NMR (126 MHz, $CDCl_3$, 21:1 mixture of *Z*:*E* isomers; only signals corresponding to the major isomer are listed) δ: 160.8, 145.0 (t, *J* = 3.6 Hz), 114.2 (t, *J* = 246.1 Hz), 70.8 (t, *J* = 25.9 Hz), 63.8, 62.4, 14.2. FTIR (neat), cm^{-1}: 3487, 2988, 2946, 1725, 1272, 1310, 1201, 1158, 1116, 1036, 859. HRMS (APCI): Calcd for $[C_7H_{11}F_2NO_4+H]^+$: 212.0729, found: 212.0731.

Synthesis of ethyl (*R*)-2-diazo-4,4-difluoro-3-hydroxybutanoate 19

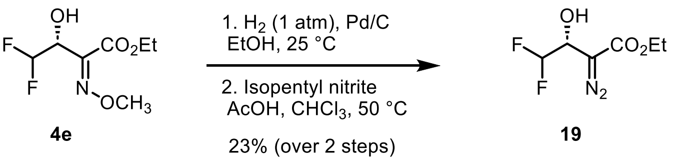

Palladium on charcoal (20% Pd basis; 48 mg) was added to a stirred solution of alcohol **4e** (240 mg, 1.14 mmol, 1 equiv) in ethanol (4.0 mL) at 25 °C. After stirring of the suspension for 10 min, the reaction flask was evacuated, and a double layered balloon filled with hydrogen gas was attached. The mixture was vigorously stirred for 2 days at 25 °C (TLC: 30% ethyl acetate in hexane; ninhydrin). Filtration through a syringe PTFE filter and concentration of the filtrate in vacuo provided a crude amino alcohol (86 mg, not shown), which was used in the next step without further purification.

To a solution of the crude amino alcohol (86 mg, 0.47 mmol, 1 equiv [assumed]) in chloroform (3.0 mL) was added isopentyl nitrite (66 mg, 0.56 mmol, 1.2 equiv) and acetic acid (5 µL, 0.093 mmol, 0.2 equiv) at 25 °C, and the resulting mixture was stirred at 50 °C for 3 h (TLC: 20% ethyl acetate in hexane; ninhydrin). Then, a saturated aqueous solution of sodium hydrogen carbonate (30 mL) was added, and the resulting mixture was extracted with dichloromethane (3 × 25 mL). The organic phase was dried over anhydrous sodium sulfate, the dried solution was filtered, and the filtrate was concentrated in vacuo. The obtained residue was purified by FCC (gradient elution with 2–20% ethyl acetate in hexane) to provide diazo ester **19** as a pale-yellow oil (21 mg, 23%).

Diazo ester **19**: TLC (20% ethyl acetate in hexane; ninhydrin): R_f = 0.35. ^{1}H NMR (500 MHz, $CDCl_3$) δ: 5.92 (t, *J* = 56.9 Hz, 1H), 4.72 (m, 1H), 4.26 (q, *J* = 7.7 Hz, 2H), 3.23 (m, 1H), 1.30 (t, *J* = 7.2 Hz, 3H). ^{13}C{^{1}H} NMR (126 MHz, $CDCl_3$) δ: 165.9, 114.4 (t, *J* = 245.9 Hz), 67.0 (t, *J* = 25.9 Hz), 61.7, 14.5 FTIR (neat), cm^{-1}: 3440, 2987, 2942, 2110, 1674, 1336, 1297, 1121, 1074, 1048, 746. HRMS (APCI): Calcd for $[C_6H_8F_2N_2O_3+Cl]^-$: 229.0197, found: 229.0195.

Synthesis of ethyl (*R*)-4,4-difluoro-3-hydroxybutanoate 6e

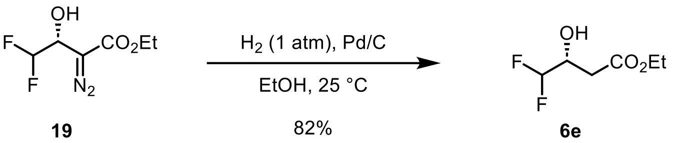

Palladium on charcoal (10% Pd basis; 4.0 mg) was added to a stirred solution of diazo ester **19** (17 mg, 0.09 mmol, 1 equiv) in ethanol (1.2 mL) at 25 °C. After stirring of the suspension for 10 min, the reaction flask was evacuated, and a double layered balloon filled with hydrogen gas was attached. The mixture was vigorously stirred for 1.5 h at 25 °C (TLC: 30% ethyl acetate in hexane; $KMnO_4$). Filtration through a syringe PTFE filter and concentration of the filtrate in vacuo to provide alcohol **6e** as a yellow oil (12 mg, 82%). NMR data of **6e** matched those obtained for the product of transfer hydrogenation of β-keto ester (see below). $[\alpha]_D^{23}$ = +21 (*c* 0.8, $CHCl_3$).

Synthesis of ethyl 4,4-difluoro-3-hydroxybutanoate 6e

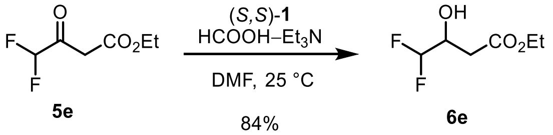

A solution of (*S,S*)-**1** (15.3 mg, 24.1 μmol, 0.02 equiv) in anhydrous *N,N*-dimethylformamide (1.2 mL) was evacuated and backfilled with argon (4 cycles). Then, β-keto ester **5e** (200 mg, 1.20 mmol, 1 equiv) was added as a solution in anhydrous *N,N*-dimethylformamide (0.5 mL), and the mixture was stirred for 5 min in order to obtain a clear solution. Then, argon was bubbled through the solution for 10 min (outlet needle), and a double-layered balloon filled with argon was attached. A mixture of formic acid and triethylamine (5:2 by volume, 0.6 mL) was added, followed by stirring for 16 h at 25 °C (TLC: 25% ethyl acetate in hexane). Then, cold water (20 mL) was added, and the resulting mixture was extracted with ethyl acetate (3 × 20 mL). The organic layer was washed with brine (20 mL), dried over anhydrous sodium sulfate, the dried solution was filtered, and the filtrate was concentrated in vacuo. The obtained residue was purified by FCC (gradient elution with 10–20% ethyl acetate in hexane) to provide alcohol **6e** as a yellow oil (168 mg, 84%).

Alcohol **6e**: $[\alpha]_D^{23}$ = −1 (*c* 0.85, $CHCl_3$). TLC (20% ethyl acetate in hexane): R_f = 0.45. ^1H NMR (500 MHz, $CDCl_3$) δ: 5.79 (td, *J* = 55.8, 3.7 Hz, 1H), 4.24–4.17 (m, 3H), 3.22 (br s, 1H), 2.70–2.58 (m, 2H), 1.29 (t, *J* = 7.1 Hz, 3H). $^{13}C\{^1H\}$ NMR (126 MHz, $CDCl_3$) δ: 171.6, 117.3–113.5 (m), 68.1 (dd, *J* = 25.7, 24.5 Hz), 61.4, 34.4 (app t, *J* = 3.7 Hz), 14.2. FTIR (neat), cm^{-1}: 3452, 2986, 2941, 1719, 1375, 1257, 1223, 1184, 1142, 1097, 1051, 1021. HRMS (APCI): Calcd for $[C_6H_{10}F_2O_3+Cl]^-$: 203.0292, found: 203.0289.

Conversion of alcohol 6e to 4-nitrobenzoate 20

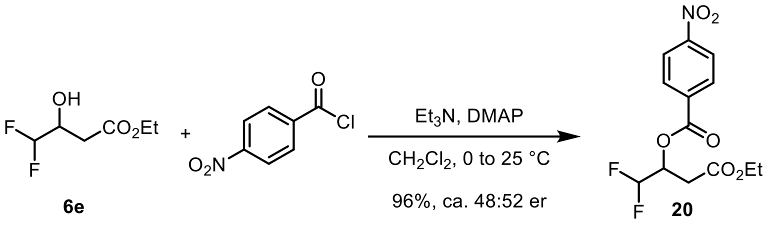

Triethylamine (78 µL, 0.56 mmol, 2.0 equiv) and 4-dimethylaminopyridine (3.4 mg, 27.8 µmol, 0.1 equiv) were added to a stirred solution of alcohol **6e** (47 mg, 0.28 mmol, 1 equiv) in anhydrous dichloromethane (1.5 mL) at 0 °C. After 5 min of stirring at 0 °C, 4-nitrobenzoyl chloride (104 mg, 0.56 mmol, 2.0 equiv) was slowly added as a solution in anhydrous dichloromethane (0.6 mL) at 0 °C. The resulting mixture was allowed to warm to 25 °C and stirred at this temperature for 16 h (TLC: 20% ethyl acetate in hexane). Then, water (10 mL) and dichloromethane (8 mL) were added. The mixture was extracted with dichloromethane (3 × 10 mL), the combined organic phases were washed with brine (10 mL), dried over anhydrous magnesium sulfate, and concentrated in vacuo. The obtained residue was purified by FCC (gradient elution with 10–12% ethyl acetate in hexane) to provide 4-nitrobenzoyl ester **20** as a colorless oil (85 mg, 96%). The enantiomeric purity of **20** was determined by HPLC analysis (ca. 48:52 er).

4-Nitrobenzoyl ester **20**: TLC (20% ethyl acetate in hexane): R_f = 0.5. ^{1}H NMR (300 MHz, $CDCl_3$) δ: 8.43−7.98 (m, 4H), 6.10 (ddd, *J* = 55.5, 54.4, 2.9 Hz, 1H), 5.83−5.61 (m, 1H), 4.16 (q, *J* = 7.1 Hz, 2H), 2.91 (d, *J* = 6.5 Hz, 2H), 1.23 (t, *J* = 7.1 Hz, 3H). $^{13}C\{^1H\}$ NMR (126 MHz, $CDCl_3$) δ: 169.0, 163.5, 151.1, 134.4, 131.2, 123.8, 113.0 (t, *J* = 245.2 Hz), 69.7 (dd, *J* = 26.8, 24.7 Hz), 61.5, 32.8 (t, *J* = 3.3 Hz), 14.2.

Enantioselective transfer hydrogenation of trifluoromethyl substrate 3f

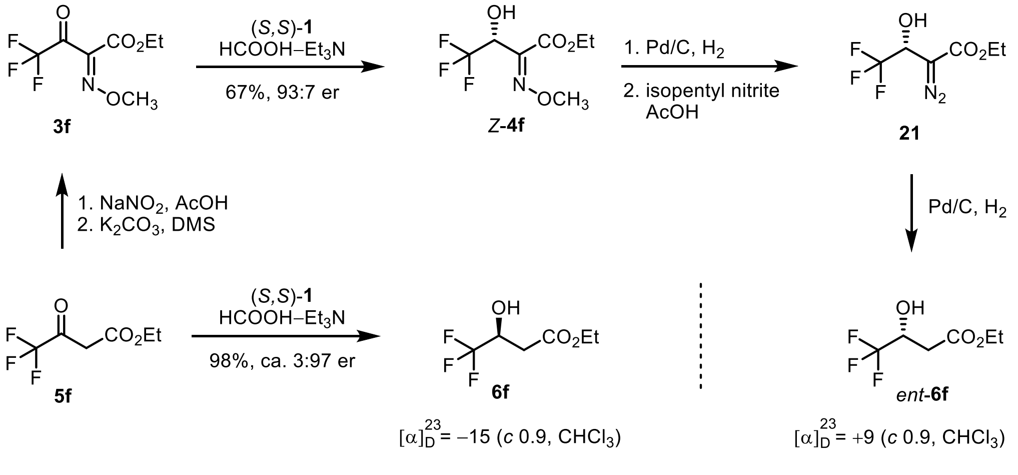

Synthesis of ethyl 4,4,4-trifluoro-2-(methoxyimino)-3-oxobutanoate 3f

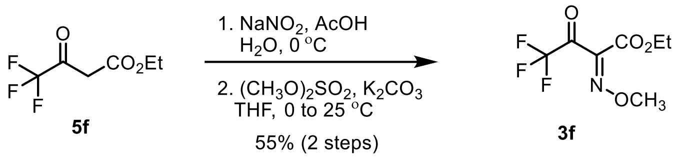

A solution of sodium nitrite (780 mg, 11.30 mmol, 1.3 equiv) in water (1.5 mL) was added dropwise to a solution of ethyl 4,4,4-trifluoro-3-oxobutanoate **5f** (1.6 g, 8.7 mmol, 1 equiv) in acetic acid (4.0 mL) over 30 min at 0 °C. The resulting mixture was stirred at room temperature for 70 min (TLC: 30% ethyl acetate in hexane; UV). Then, the mixture was poured into cold brine (30 mL) and extracted with diethyl ether (3 × 30 mL). The organic extracts were combined and washed with a saturated aqueous solution of sodium hydrogen carbonate (100 mL) to reach pH ~ 7, and the aqueous phase was extracted again with diethyl ether (3 × 30 mL). All organic extracts were combined, dried over anhydrous sodium sulfate, and filtered. The filtrate was concentrated under reduced pressure to yield the crude α-hydroxyimino ester (838 mg, not shown), which was used in the next step without further purification.

Potassium carbonate (436 mg, 3.16 mmol, 1.3 equiv) was added to a stirred solution of crude α-hydroxyimino ester (517 mg, 2.43 mmol, 1 equiv) in tetrahydrofurane (18 mL) at 0 °C. After 5 min of stirring at 0 °C, dimethyl sulfate (214 mg, 1.70 mmol, 0.7 equiv) was added at 0 °C, and the resulting solution was allowed to warm to room temperature and stirred at this temperature for 23 h (TLC: 30% ethyl acetate in hexane). The reaction mixture was filtered, ice-cold brine (30 mL) was added, and the resulting mixture was extracted with dichloromethane (3 × 30 mL). The combined organic phases were dried over anhydrous sodium sulfate, the dried solution was filtered, and the filtrate was concentrated in vacuo. The obtained residue was purified by FCC (gradient elution with 20–40% ethyl acetate in hexane) to provide α-methoxyimino ester **3f** as a pale-yellow oil (305 mg, 55%, 6:1 mixture of *Z*:*E* isomers).

α-Methoxyimino ester **3f**: TLC (30% ethyl acetate in hexane): R_f= 0.65. ^{1}H NMR (500 MHz, $CDCl_3$, 6:1 mixture of *Z*:*E* isomers; only signals corresponding to the major isomer are listed) δ: 4.36 (q, *J* = 7.1 Hz, 2H), 4.23 (s, 3H), 1.36 (t, *J* = 7.2 Hz, 3H). $^{13}C\{^1H\}$ NMR (126 MHz, $CDCl_3$, 6:1 mixture of *Z*:*E* isomers; only signals corresponding to the major isomer are listed) δ: 175.3 (q, *J* = 37.9 Hz), 158.7, 145.8, 115.7 (q, *J* = 290.0 Hz), 66.0, 63.1, 14.1. FTIR (neat), cm^{-1}: 3427, 3126, 2998, 2948, 1728, 1373, 1286, 1185, 1086, 1033, 897, 858. HRMS (APCI): Calcd for $[C_7H_8F_3NO_4+H]^+$: 228.0478, found: 228.0478.

Synthesis of ethyl (*R*)-4,4,4-trifluoro-3-hydroxy-2-(methoxyimino)butanoate *Z*-4f

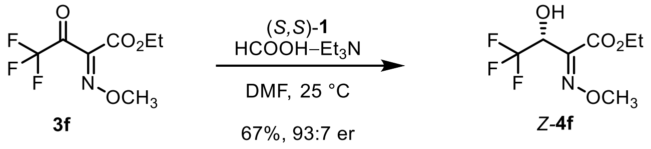

A solution of (*S,S*)-**1** (16.3 mg, 25.6 μmol, 0.02 equiv) in anhydrous *N,N*-dimethylformamide (1.0 mL) was evacuated and backfilled with argon (4 cycles). Then, α-methoxyimino ester **3f** (290 mg, 1.28 mmol, 1 equiv, 6:1 ratio of *Z:E* isomers) was added as a solution in anhydrous *N,N*-dimethylformamide (0.5 mL), and the mixture was stirred for 5 min in order to obtain a clear solution. Then, argon was bubbled through the solution for 10 min (outlet

needle), and a double-layered balloon filled with argon was attached. A mixture of formic acid and triethylamine (5:2 by volume, 0.64 mL) was added, followed by stirring for 40 h at 25 °C (TLC: 30% ethyl acetate in hexane). Then, ice-cold water (25 mL) was added, and the resulting mixture was extracted with ethyl acetate (3 × 30 mL). The combined organic phases were washed with brine (20 mL), dried over anhydrous sodium sulfate, the dried solution was filtered, and the filtrate was concentrated in vacuo. The obtained residue was purified by FCC (25% ethyl acetate in hexane) to provide alcohol *Z*-**4f** as a colorless oil (195 mg, 67%, *Z* isomer). The enantiomeric purity of *Z*-**4f** was determined by HPLC (93:7 er).

Alcohol *Z*-**4f**: TLC (30% ethyl acetate in hexane): R_f= 0.6. ^{1}H NMR (500 MHz, $CDCl_3$) δ: 4.86 (dq, *J* = 8.4, 6.4 Hz, 1H), 4.35 (q, *J* = 7.1, 2H), 4.03 (s, 3H), 3.60 (d, *J* = 8.4 Hz, 1H), 1.34 (t, *J* = 7.1 Hz, 3H). ^{13}C{^{1}H} NMR (126 MHz, $CDCl_3$) δ: 160.2, 142.7, 123.2 (q, *J* = 283.1 Hz), 70.2 (q, *J* = 33.4 Hz), 64.2, 62.7, 14.1. FTIR (neat), cm^{-1}: 3491, 2989, 2947, 1727, 1372, 1309, 1263, 1176, 1134, 1094, 1038, 886,863, 693. HRMS (APCI): Calcd for $[C_7H_{10}F_3NO_4+Cl]^-$: 264.0256, found: 264.0253.

Synthesis of ethyl (*R*)-2-diazo-4,4,4-trifluoro-3-hydroxybutanoate 21

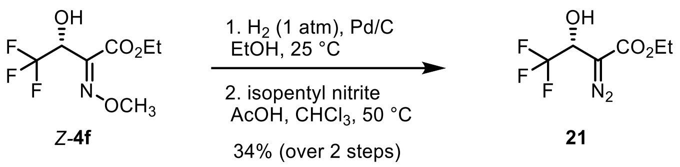

Palladium on charcoal (10% Pd basis; 35 mg) was added to a stirred solution of alcohol *Z*-**4f** (174 mg, 0.76 mmol, 1 equiv) in ethanol (3.5 mL) at 25 °C. After stirring of the suspension for 10 min, the reaction flask was evacuated, and a double layered balloon filled with hydrogen gas was attached. The mixture was vigorously stirred for 3 days at 25 °C (TLC: 30% ethyl acetate in hexane; ninhydrin). Filtration through a syringe PTFE filter and concentration of the filtrate in vacuo provided a crude amino alcohol (149 mg, not shown), which was used in the next step without further purification.

To a solution of the crude amino alcohol (149 mg, 0.74 mmol, 1 equiv [assumed]) in chloroform (4.0 mL) was added isopentyl nitrite (104 mg, 0.89 mmol, 1.2 equiv) and acetic acid (9 μL, 0.150 mmol, 0.2 equiv) at 25 °C, and the resulting mixture was stirred at 50 °C for 1.5 h (TLC: 30% ethyl acetate in hexane; ninhydrin). Then, a saturated aqueous solution of sodium hydrogen carbonate (20 mL) was added, and the resulting mixture was extracted with dichloromethane (3 × 25 mL), the organic phase was dried over anhydrous sodium sulfate, the dried solution was filtered, and the filtrate was concentrated in vacuo. The obtained residue was purified by FCC (gradient elution with 8-20% ethyl acetate in hexane) to provide diazo ester **21** as a pale-yellow oil (54 mg, 34%).

Diazo ester **21**: TLC (30% ethyl acetate in hexane; ninhydrin): R_f = 0.60. ^{1}H NMR (500 MHz, $CDCl_3$) δ: 4.97 (d, *J* = 6.1 Hz, 1H), 4.28 (q, *J* = 7.2 Hz, 2H), 1.31 (t, *J* = 7.2 Hz, 3H). ^{13}C{^{1}H} NMR (126 MHz, $CDCl_3$) δ: 165.3, 124.2 (q, *J* = 282.7 Hz), 66.4 (q, *J* = 34.5 Hz), 61.9, 14.5. FTIR (neat), cm^{-1}: 3493,

2988, 2939, 2110, 1669, 1377, 1300, 1266, 1174, 1147, 1109, 1068, 1013, 879, 827, 744, 682. HRMS (APCI): Calcd for $[C_6H_7F_3N_2O_3+Cl]^-$: 247.0103, found: 247.0105.

Synthesis of ethyl (*R*)-4,4,4-trifluoro-3-hydroxybutanoate *ent*-6f

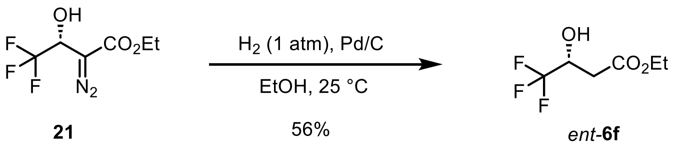

Palladium on charcoal (10% Pd basis; 18 mg) was added to a stirred solution of diazo ester **21** (39 mg, 0.18 mmol, 1 equiv) in ethyl acetate (1.5 mL) at 25 °C. After stirring of the suspension for 10 min, the reaction flask was evacuated, and a double layered balloon filled with hydrogen gas was attached. The mixture was vigorously stirred for 1 h at 25 °C (TLC: 20% ethyl acetate in hexane; $KMnO_4$). Filtration through a syringe PTFE filter and concentration of the filtrate in vacuo. The obtained residue was purified by FCC (25% ethyl acetate in hexane) to provide alcohol *ent*-**6f** as a colorless oil (19 mg, 56%). NMR data of *ent*-**6f** matched those obtained for the product of transfer hydrogenation of β-keto ester (see below). $[\alpha]_D^{23}$ = +9 (*c* 0.9, $CHCl_3$).

Synthesis of ethyl (*S*)-4,4,4-trifluoro-3-hydroxybutanoate 6f

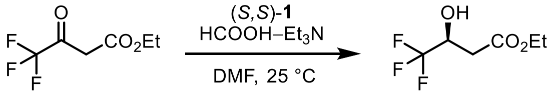

Ethyl (*S*)-4,4,4-trifluoro-3-hydroxybutanoate was prepared by using the literature procedure.[2] A solution of (*S,S*)-**1** (3.5 mg, 5.4 μmol, 0.02 equiv) in anhydrous *N,N*-dimethylformamide (1.5 mL) was evacuated and backfilled with argon (4 cycles). Then, ethyl 4,4,4-trifluoro-3-oxobutanoate **5f** (0.33 mL, 2.72 mmol, 1 equiv) was added as a solution in anhydrous *N,N*-dimethylformamide (0.5 mL), and the mixture was stirred for 5 min in order to obtain a clear solution. Then, argon was bubbled through the solution for 10 min (outlet needle), and a double-layered balloon filled with argon was attached. A mixture of formic acid and triethylamine (5:2 by volume, 1.4 mL) was added, followed by stirring for 16 h at 25 °C (TLC: 25% ethyl acetate in hexane). Then, cold water (20 mL) was added, and the resulting mixture was extracted with ethyl acetate (3 × 30 mL). The organic layer was washed with brine (30 mL), dried over anhydrous sodium sulfate, the dried solution was filtered, and the filtrate was concentrated in vacuo. The obtained residue was purified by FCC (gradient elution with 10–20% ethyl acetate in hexane) to provide alcohol **6f** as a yellow oil (497 mg, 98%). The enantiomeric purity of **6f** was determined by GC analysis (3:97 er).

Alcohol **6f**: Spectroscopic data for **6f** were in accordance with those reported in the literature.[2] $[\alpha]_D^{23}$ = −15 (*c* 0.9, $CHCl_3$). TLC (20% ethyl acetate in hexane): R_f = 0.45. ^1H NMR (300 MHz, $CDCl_3$) δ: 4.46 (m, 1H), 4.24 (q, *J* = 7.1 Hz, 2H), 3.62 (d, *J* = 5.6 Hz, 1H), 2.71 (m, 2H), 1.29 (t, *J* = 7.2 Hz, 3H).

Enantioselective transfer hydrogenation of isopropyl substrate 3g

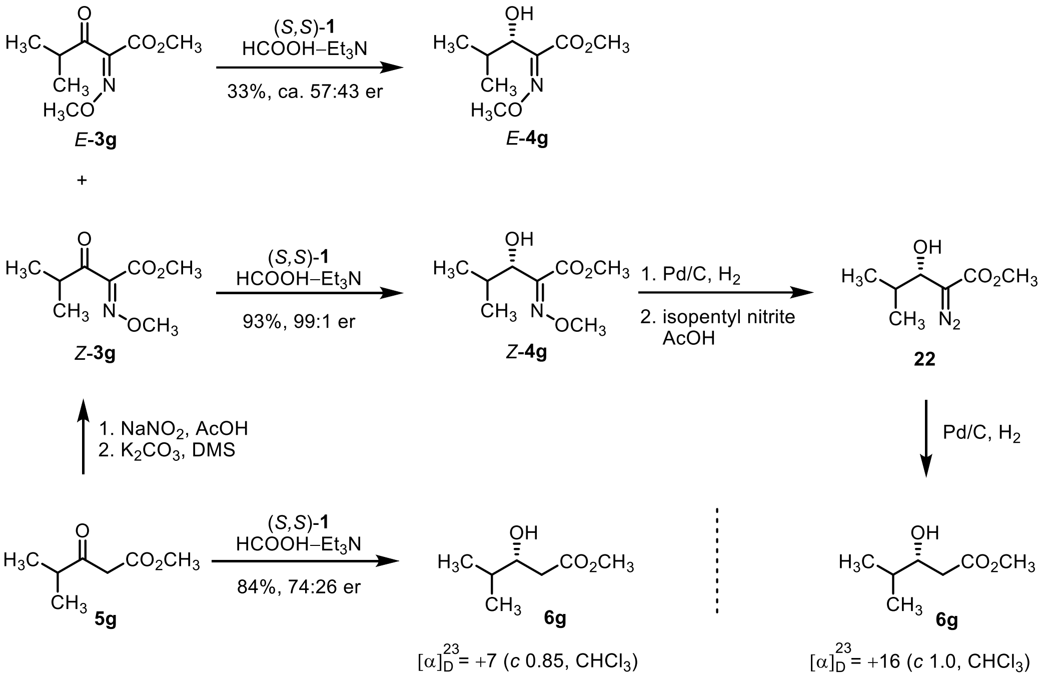

Synthesis of methyl 2-(methoxyimino)-4-methyl-3-oxopentanoate 3g

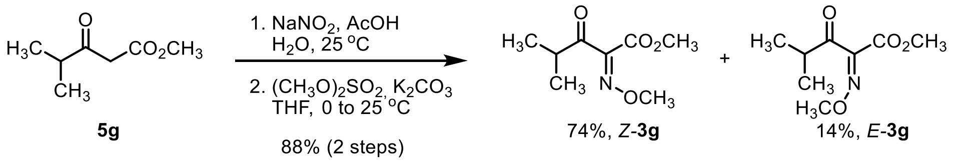

A solution of sodium nitrite (1.15 g, 16.6 mmol, 1.2 equiv) in water (2.5 mL) was added dropwise to a solution of methyl 4-methyl-3-oxopentanoate **5g** (2.00 g, 13.9 mmol, 1 equiv) in acetic acid (3.5 mL) over 40 min at 25 °C. The resulting mixture was stirred at this temperature for 1.5 h (TLC: 30% ethyl acetate in hexane; UV, PMA). Then, the mixture was poured into brine (80 mL) and extracted with ether (3 × 40 mL). The organic extracts were combined and washed with a saturated aqueous solution of sodium hydrogen carbonate (120 mL) to reach pH ~ 7, and the aqueous phase was extracted again with ether (3 × 40 mL). All organic extracts were combined, dried over anhydrous sodium sulfate, and filtered. The filtrate was concentrated under reduced pressure to yield the crude α-hydroxyimino ester (2.3 g, not shown), which was used in the next step without further purification.

Potassium carbonate (2.24 g, 16.2 mmol, 1.2 equiv) was added to a stirred solution of crude α-hydroxyimino ester (2.3 g, 13.5 mmol, 1 equiv) in anhydrous tetrahydrofuran (35 mL) at 0 °C. After 30 min of stirring at 0 °C, dimethyl sulfate (1.28 mL, 13.5 mmol, 1.0 equiv) was added at 0 °C, and the resulting solution was allowed to warm to room temperature and stirred at this temperature for 18 h (TLC: 20% ethyl acetate in hexane). The reaction mixture was filtered, ice-cold brine (80 mL) was added, and the resulting mixture was extracted with dichloromethane (3 × 80 mL). The combined organic phases were dried over anhydrous sodium sulfate, the dried solution was filtered, and the filtrate was concentrated in vacuo. The obtained residue was purified by FCC (gradient elution with 8–12% ethyl acetate in hexane) to provide separately (*Z*)-

α-methoxyimino ester *Z*-**3g** as a colorless oil (1.88 g, 74%) and (*E*)-α-methoxyimino ester *E*-**3g** as a colorless oil (348 mg, 14%).

α-Methoxyimino ester *Z*-**3g**: TLC (20% ethyl acetate in hexane): R_f = 0.65. ^{1}H NMR (300 MHz, $CDCl_3$) δ: 4.08 (s, 3H), 3.85 (s, 3H), 3.40 (hept, *J* = 6.9 Hz, 1H), 1.14 (d, *J* = 7.0 Hz, 6H). ^{13}C{^{1}H} NMR (126 MHz, $CDCl_3$) δ: 199.1, 161.9, 148.7, 64.4, 52.6, 35.5, 18.5. FTIR (neat), cm^{-1}: 2957, 2944, 1750, 1708, 1596, 1461, 1439, 1386, 1318, 1281, 1209, 1154, 1119, 1048, 997, 969, 939, 892, 868, 791. HRMS (APCI): Calcd for $[C_8H_{13}NO_4+H]^+$: 188.0917, found: 188.0915.

α-Methoxyimino ester *E*-**3g**: TLC (20% ethyl acetate in hexane): R_f = 0.5. ^{1}H NMR (500 MHz, $CDCl_3$) δ: 4.05 (s, 3H), 3.86 (s, 3H), 2.80 (hept, *J* = 7.0 Hz, 1H), 1.15 (d, *J* = 7.0 Hz, 6H). ^{13}C{^{1}H} NMR (126 MHz, $CDCl_3$) δ: 203.1, 161.0, 150.0, 64.3, 53.1, 40.7, 16.9. FTIR (neat), cm^{-1}: 2958, 2945, 1745, 1709, 1591, 1462, 1431, 1385, 1314, 1285, 1205, 1153, 1115, 1049, 991, 968, 934, 891, 864, 790. HRMS (APCI): Calcd for $[C_8H_{13}NO_4+H]^+$: 188.0917, found: 188.0916.

Synthesis of methyl (*S, Z*)-3-hydroxy-2-(methoxyimino)-4-methylpentanoate *Z*-4g

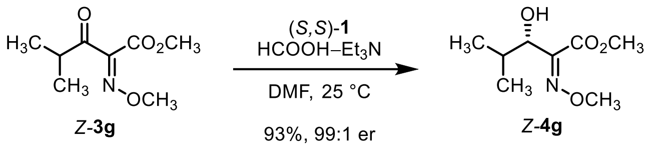

A solution of (*S,S*)-**1** (25.4 mg, 39.9 μmol, 0.02 equiv) in anhydrous *N*,*N*-dimethylformamide (2.0 mL) was evacuated and backfilled with argon (4 cycles). Then, α-methoxyimino ester *Z*-**3g** (374 mg, 2.03 mmol, 1 equiv, *Z* isomer) was added as a solution in anhydrous *N*,*N*-dimethylformamide (0.5 mL), and the mixture was stirred for 5 min in order to obtain a clear solution. Then, argon was bubbled through the solution for 10 min (outlet needle), and a double-layered balloon filled with argon was attached. A mixture of formic acid and triethylamine (5:2 by volume, 1.0 mL) was added, followed by stirring for 16 h at 25 °C (TLC: 25% ethyl acetate in hexane). Then, ice-cold water (30 mL) was added, and the resulting mixture was extracted with ethyl acetate (3 × 30 mL). The combined organic phases were washed with brine (25 mL), dried over anhydrous sodium sulfate, the dried solution was filtered, and the filtrate was concentrated in vacuo. The obtained residue was purified by FCC (gradient elution with 12–16% ethyl acetate in hexane) to provide alcohol *Z*-**4g** as a pale-yellow oil (352 mg, 93%, *Z* isomer). The enantiomeric purity of *Z*-**4g** was determined by HPLC (99:1 er).

Alcohol *Z*-**4g**: TLC (20% ethyl acetate in hexane): R_f = 0.35. ^{1}H NMR (300 MHz, $CDCl_3$) δ: 4.16 (t, *J* = 5.6 Hz, 1H), 3.91 (s, 3H), 3.84 (s, 3H), 2.52 (d, *J* = 5.8 Hz, 1H), 2.02–1.75 (m, *J* = 6.7 Hz, 1H), 0.97 (t, *J* = 7.0 Hz, 6H). ^{13}C{^{1}H} NMR (126 MHz, $CDCl_3$) δ: 163.1, 152.1, 75.8, 63.0, 52.5, 32.0, 19.0, 16.9. FTIR (neat), cm^{-1}: 3524, 2961, 2874, 1719, 1601, 1439, 1335, 1274, 1198, 1150, 1122, 1034, 975, 958, 934, 865, 817, 782. HRMS (APCI): Calcd for $[C_8H_{15}NO_4+H]^+$: 190.1074, found: 190.1076.

Synthesis of methyl (*S, E*)-3-hydroxy-2-(methoxyimino)-4-methylpentanoate *E*-4g

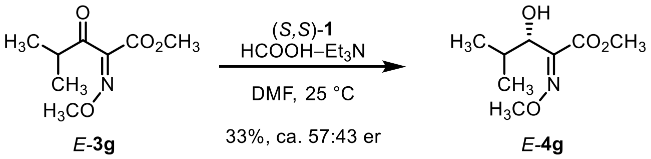

A solution of (*S,S*)-**1** (4.1 mg, 6.4 μmol, 0.02 equiv) in anhydrous *N,N*-dimethylformamide (0.6 mL) was evacuated and backfilled with argon (4 cycles). Then, α-methoxyimino ester *E*-**3g** (60 mg, 0.32 mmol, 1 equiv, *E* isomer) was added as a solution in anhydrous *N,N*-dimethylformamide (0.3 mL), and the mixture was stirred for 5 min in order to obtain a clear solution. Then, argon was bubbled through the solution for 10 min (outlet needle), and a double-layered balloon filled with argon was attached. A mixture of formic acid and triethylamine (5:2 by volume, 0.23 mL) was added, followed by stirring for 10 days at 25 °C (TLC: 25% ethyl acetate in hexane). Then, ice-cold water (20 mL) was added, and the resulting mixture was extracted with ethyl acetate (3 × 20 mL). The combined organic phases were washed with brine (20 mL), dried over anhydrous sodium sulfate, the dried solution was filtered, and the filtrate was concentrated in vacuo. The obtained residue was purified by FCC (gradient elution with 10–15% ethyl acetate in hexane) to provide separately alcohol *E*-**4g** as a pale-yellow oil (20 mg, 33%, *E* isomer) and recovered starting material *E*-**3g** (24 mg, 40%). The enantiomeric purity of *E*-**4g** was determined by HPLC analysis (ca. 57:43 er).

Alcohol *E*-**4g**: TLC (20% ethyl acetate in hexane): R_f = 0.35. ^1H NMR (500 MHz, $CDCl_3$) δ: 4.59 (dd, *J* = 11.4, 8.5 Hz, 1H), 4.04 (s, 3H), 3.85 (s, 3H), 3.32 (d, *J* = 11.5 Hz, 1H), 2.01 (dp, *J* = 8.4, 6.8 Hz, 1H), 1.04 (d, *J* = 6.7 Hz, 3H), 0.84 (d, *J* = 6.9 Hz, 3H). $^{13}C\{^1H\}$ NMR (126 MHz, $CDCl_3$) δ: 164.1, 152.2, 72.6, 63.7, 52.9, 33.1, 18.8, 18.6. FTIR (neat), cm^{-1}: 3523, 2965, 2878, 1716, 1604, 1431, 1339, 1271, 1194, 1154, 1121, 1054, 978, 951, 937, 861, 819, 783. HRMS (APCI): Calcd for $[C_8H_{15}NO_4+H]^+$: 190.1074, found: 190.1071.

Synthesis of methyl (*S*)-2-diazo-3-hydroxy-4-methylpentanoate 22

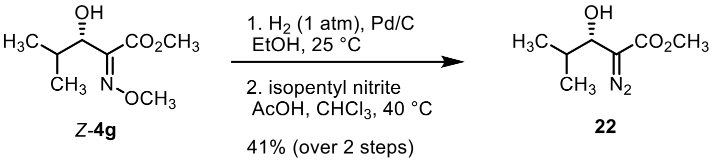

Palladium on charcoal (10% Pd basis; 80 mg) was added to a stirred solution of alcohol *Z*-**4g** (399 mg, 2.11 mmol, 1 equiv) in anhydrous ethanol (10 mL) at 25 °C. After stirring of the suspension for 10 min, the reaction flask was evacuated, and a double layered balloon filled with hydrogen gas was attached. The mixture was vigorously stirred for 4 days at 25 °C (TLC: 35% ethyl acetate in hexane; ninhydrin). Filtration through a syringe PTFE filter and concentration of the filtrate in vacuo provided a crude amino alcohol (34 mg, not shown), which was used in the next step without further purification.

To a solution of the crude amino alcohol (34 mg, 1.95 mmol, 1 equiv [assumed]) in chloroform (9.0 mL) was added isopentyl nitrite (0.31 mL, 2.34 mmol, 1.2 equiv) and acetic acid (22 μL, 0.39 mmol, 0.2 equiv) at 25 °C, and the resulting mixture was stirred at 40 °C for 3 h (TLC: 25% ethyl acetate in hexane; ninhydrin). Then, a saturated aqueous solution of sodium hydrogen carbonate (20 mL) was added, and the resulting mixture was extracted with dichloromethane (3

× 20 mL), the organic phase was dried over anhydrous sodium sulfate, the dried solution was filtered, and the filtrate was concentrated in vacuo. The obtained residue was purified by FCC (gradient elution with 25–30% diethyl ether in hexane) to provide diazo ester **22** as a pale-yellow oil (150 mg, 41% ca. 85% purity).

<u>Diazo ester **22**</u>: TLC (30% diethyl ether in hexane; ninhydrin): $R_f = 0.3$. ^1H NMR (500 MHz, $CDCl_3$) δ: 4.27 (dd, *J* = 8.6, 2.3 Hz, 1H), 3.78 (s, 3H), 2.57 (br s, 1H), 1.88 (m, 1H), 1.06 (d, *J* = 6.6 Hz, 3H), 0.94 (d, *J* = 6.8 Hz, 3H). $^{13}C\{^1H\}$ NMR (126 MHz, $CDCl_3$) δ: 167.2, 72.4, 52.1, 33.0, 18.9, 18.8. FTIR (neat), cm^{-1}: 3460, 2960, 2875, 2093, 1738, 1669, 1466, 1437, 1361, 1336, 1290, 1191, 1134, 1117, 1092, 1033, 968.

Synthesis of methyl (*R*)-3-hydroxy-4-methylpentanoate 6g

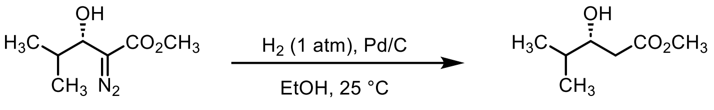

22 40% **6g**

Palladium on charcoal (10% Pd basis; 24 mg) was added to a stirred solution of diazo ester **22** (120 mg, 0.70 mmol, 1 equiv) in anhydrous ethanol (4.5 mL) at 25 °C. After stirring of the suspension for 10 min, the reaction flask was evacuated, and a double layered balloon filled with hydrogen gas was attached. The mixture was vigorously stirred for 1.5 h at 25 °C (TLC: 20% ethyl acetate in hexane; PMA, ninhydrin). Filtration through a syringe PTFE filter and concentration of the filtrate in vacuo. The obtained residue was purified by FCC (gradient elution with 25–30% ethyl acetate in hexane) to provide alcohol **6g** as a pale-yellow oil (41 mg, 40%). NMR data of **6g** matched those obtained for the product of transfer hydrogenation of β-keto ester (see below). $[\alpha]_D^{23} = +16$ (*c* 1.0, $CHCl_3$).

Synthesis of methyl (*R*)-3-hydroxy-4-methylpentanoate 5g

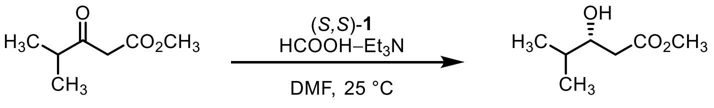

5g 84%, 74:26 er **6g**

A solution of (*S,S*)-**1** (17.6 mg, 27.7 μmol, 0.02 equiv) in anhydrous *N*,*N*-dimethylformamide (1.2 mL) was evacuated and backfilled with argon (4 cycles). Then, methyl 4-methyl-3-oxopentanoate **5g** (200 mg, 1.39 mmol, 1 equiv) was added as a solution in anhydrous *N*,*N*-dimethylformamide (0.4 mL), and the mixture was stirred for 5 min in order to obtain a clear solution. Then, argon was bubbled through the solution for 10 min (outlet needle), and a double-layered balloon filled with argon was attached. A mixture of formic acid and triethylamine (5:2 by volume, 0.7 mL) was added, followed by stirring for 7 days at 25 °C (TLC: 25% ethyl acetate in hexane). Then, ice-cold water (20 mL) was added, and the resulting mixture was extracted with ethyl acetate (3 × 20 mL). The combined organic phases were washed with brine (20 mL), dried over anhydrous sodium sulfate, the dried solution was filtered, and the filtrate was concentrated in vacuo. The obtained residue was purified by FCC (gradient elution with 10–15% ethyl acetate

in hexane) to provide alcohol **6g** as a pale-yellow oil (170 mg, 84%). The enantiomeric purity of **6g** was determined by GC (74:26 er).

Alcohol **6g**: $[\alpha]_D^{23}$ = +7 (*c* 0.85, $CHCl_3$). TLC (20% ethyl acetate in hexane; PMA): R_f = 0.3. ^{1}H NMR (300 MHz, $CDCl_3$) δ: 3.78 (dt, *J* = 6.2, 3.0 Hz, 1H), 3.75 (s, 3H), 2.85 (br s, 1H), 2.58–2.34 (m, 2H), 1.79–1.58 (m, 1H), 0.93 (dd, *J* = 8.4, 6.8 Hz, 6H). ^{13}C{^{1}H} NMR (126 MHz, $CDCl_3$) δ: 174.0, 72.9, 51.9, 38.5, 33.3, 18.5, 17.9. FTIR (neat), cm^{-1}: 3468, 2957, 2855, 1720, 1438, 1367, 1327, 1276, 1164, 1104, 1051, 997, 876. HRMS (APCI): Calcd for $[C_7H_{14}O_3+H]^+$: 147.1016, found: 147.1015.

Enantioselective transfer hydrogenation of cyclohexyl substrate 3h

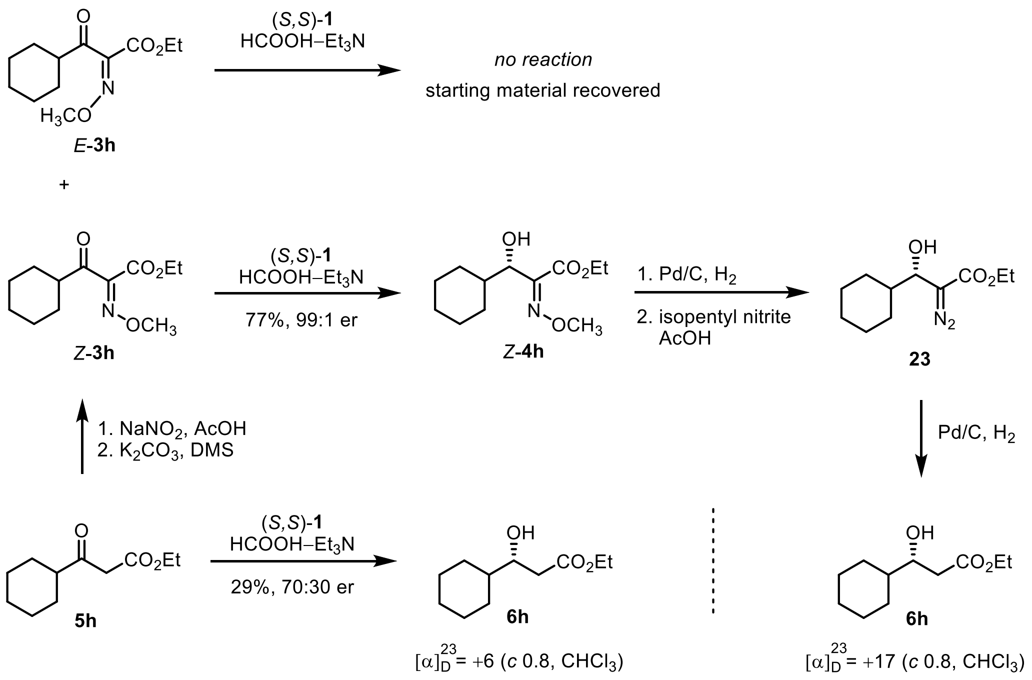

Synthesis of ethyl 3-cyclohexyl-3-oxopropanoate 5h

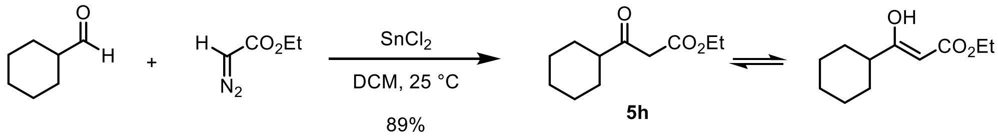

Anhydrous tin(II) chloride (116 mg, 0.61 mmol, 0.1 equiv) was added to dichloromethane (7.0 mL) under argon atmosphere at 25 °C. Ethyl diazoacetate (767 mg, 6.73 mmol, 1.1 equiv) was added and the resulting suspension was stirred for 5 min. Then, cyclohexane carbaldehyde (685 mg, 6.11 mmol, 1 equiv) was added portionwise over 30 min. The resulting mixture was stirred for 2 h at 25 °C. Then, brine (60 mL) was added, and the phases were separated. The water phase was extracted with ether (3 × 50 mL). Combined organic phases were dried over sodium sulfate, filtered, and the solvents were evaporated in vacuo. The residue was purified by FCC (elution with 15% diethyl ether in hexane) to produce β-keto ester **5h**[5] as a yellow oil (1.08 g, 89%, 4:1 mixture of keto and enol form).

Ethyl 3-cyclohexyl-3-oxopropanoate **5h**: TLC (40% diethyl ether in hexane; $KMnO_4$): R_f = 0.6. ^1H NMR (500 MHz, $CDCl_3$, 4:1 mixture of keto and enol form) δ: 12.13 (s, 1H), 4.95 (s, 1H), 4.18 (qd, *J* = 7.2, 3.7 Hz, 2H), 3.46 (s, 2H), 2.45 (tt, *J* = 11.1, 3.4 Hz, 1H), 2.09 (tt, *J* = 11.4, 3.4 Hz, 1H), 1.87 (m, 2H), 1.78 (m, 2H), 1.67 (m, 1H), 1.29 (m, 8H). $^{13}C\{^1H\}$ NMR (126 MHz, $CDCl_3$, 4:1 mixture of keto and enol form) δ: 206.0, 182.9, 173.3, 167.6, 87.1, 61.4, 60.0, 51.0, 47.5, 43.7, 30.1, 28.3, 25.9, 25.6, 14.4, 14.3. FTIR (neat), cm^{-1}: 2982, 2930, 2855, 1742, 1708, 1644, 1622, 1449, 1411, 1268, 1318, 1301, 1237, 1219, 1184, 1154, 1096, 1031, 1003. HRMS (APCI): Calcd for $[C_{11}H_{18}O_3+H]^+$: 199.1329, found: 199.1329.

Synthesis of ethyl 3-cyclohexyl-2-(methoxyimino)-3-oxopropanoate 3h

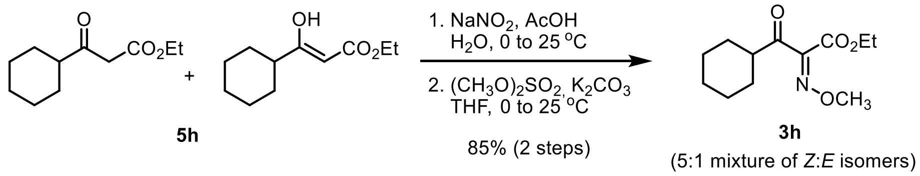

A solution of sodium nitrite (414 mg, 6.00 mmol, 1.3 equiv) in water (1.2 mL) was added dropwise to a solution of ethyl 3-cyclohexyl-3-oxopropanoate **5h**[3](914 mg, 4.62 mmol, 1 equiv) in acetic acid (3.0 mL) over 30 min at 0 °C. The resulting mixture was stirred at this temperature for 10 min, then for 1 h at 25 °C (TLC: 30% ethyl acetate in hexane; UV). The reaction mixture was poured into brine (40 mL) and extracted with ether (3 × 35 mL). The organic extracts were combined and washed with a saturated aqueous solution of sodium hydrogen carbonate (100 mL) to reach pH ~ 7, and the aqueous phase was extracted again with ether (3 × 35 mL). All organic extracts were combined, dried over anhydrous sodium sulfate, and filtered. The filtrate was concentrated under reduced pressure to yield the crude α-hydroxyimino ester (939 mg, not shown), which was used in the next step without further purification.

Potassium carbonate (685 mg, 4.97 mmol, 1.2 equiv) was added to a stirred solution of crude α-hydroxyimino ester (939 mg, 4.14 mmol, 1 equiv) in anhydrous tetrahydrofuran (18 mL) at 0 °C. After 5 min of stirring at 0 °C, dimethyl sulfate (0.43 mL, 4.97 mmol, 1.1 equiv) was added at 0 °C, and the resulting solution was allowed to warm to room temperature and stirred at this temperature for 48 h (TLC: 30% ethyl acetate in hexane, UV, $KmnO_4$). The reaction mixture was

filtered, ice-cold brine (40 mL) was added, and the resulting mixture was extracted with dichloromethane (3 × 40 mL). The combined organic phases were dried over anhydrous sodium sulfate, the dried solution was filtered, and the filtrate was concentrated in vacuo. The obtained residue was purified by FCC (15% ethyl acetate in hexane) to provide α-methoxyimino ester **3h** as a colorless oil (793 mg, 80%, 5:1 mixture of *Z*:*E* isomers).

α-Methoxyimino ester **3h**: TLC (30% ethyl acetate in hexane): R_f= 0.75. ^{1}H NMR (500 MHz, $CDCl_3$, 5:1 mixture of *Z*:*E* isomers; only signals corresponding to the major isomer are listed) δ: 4.33 (q, *J* = 7.1 Hz, 2H), 4.09 (s, 3H), 3.17 (tt, *J* = 11.4, 3.4 Hz, 1H), 1.83 (m, 4H), 1.69 (dddt, *J* = 12.8, 5.0, 3.4, 1.6 Hz, 1H), 1.35 (m, 8H). ^{13}C{^{1}H} NMR (126 MHz, $CDCl_3$, 5:1 mixture of *Z*:*E* isomers; only signals corresponding to the major isomer are listed) δ: 198.5, 161.6, 149.0, 64.4, 62.5, 45.2, 28.8, 25.9, 25.7, 14.2. FTIR (neat), cm^{-1}: 2984, 2933, 2856, 1742, 1686, 1449, 1375, 1319, 1282, 1243, 1204, 1135, 1045, 1024, 975, 9189, 859, 673. HRMS (APCI): Calcd for $[C_{12}H_{19}NO_4+H]^+$: 242.1387, found: 242.1389.

Synthesis of ethyl (*S, Z*)-3-cyclohexyl-3-hydroxy-2-(methoxyimino)propanoate *Z*-4h

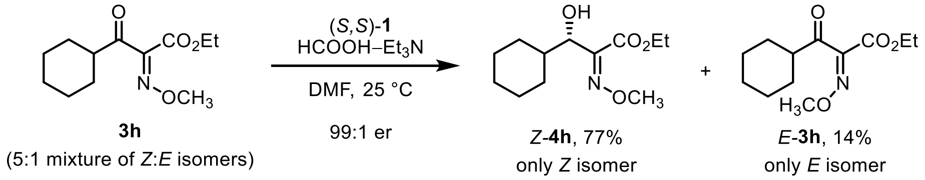

A solution of (*S,S*)-**1** (19.3 mg, 30.3 μmol, 0.02 equiv) in anhydrous *N,N*-dimethylformamide (1.0 mL) was evacuated and backfilled with argon (4 cycles). Then, α-methoxyimino ester **3h** (365 mg, 1.51 mmol, 1 equiv, 5:1 ratio of *Z*:*E* isomers) was added as a solution in anhydrous *N,N*-dimethylformamide (0.8 mL), and the mixture was stirred for 5 min in order to obtain a clear solution. Then, argon was bubbled through the solution for 15 min (outlet needle), and a double-layered balloon filled with argon was attached. A mixture of formic acid and triethylamine (5:2 by volume, 0.76 mL) was added, followed by stirring for 24 h at 25 °C (TLC: 30% ethyl acetate in hexane, UV, PMA). Then, ice-cold water (30 mL) was added, and the resulting mixture was extracted with ethyl acetate (3 × 30 mL). The combined organic phases were washed with brine (25 mL), dried over anhydrous sodium sulfate, the dried solution was filtered, and the filtrate was concentrated in vacuo. The obtained residue was purified by FCC (15% ethyl acetate in hexane) to provide alcohol *Z*-**4h** as a colorless oil (282 mg, 77%, *Z* isomer) and recovered unreacted (*E*)-α-methoxyimino ester *E*-**3h** as a colorless oil (51 mg, 14%). The enantiomeric purity of *Z*-**4h** was determined by HPLC (99:1 er).

Alcohol *Z*-**4h**: TLC (30% ethyl acetate in hexane): R_f= 0.60. ^{1}H NMR (500 MHz, $CDCl_3$) δ: 4.33 (q, *J* = 7.1 Hz, 2H), 4.16 (t, *J* = 6.0 Hz, 1H), 3.90 (s, 3H), 2.40 (d, *J* = 5.7 Hz, 1H), 1.87 (ddq, *J* = 12.3, 3.5, 2.0 Hz, 1H), 1.76 (m, 2H), 1.67 (m, 2H), 1.56 (tdt, *J* = 11.3, 6.5, 3.3 Hz, 1H), 1.33 (t, *J* = 7.1 Hz, 3H), 1.27 (m, 6H). ^{13}C{^{1}H} NMR (126 MHz, $CDCl_3$) δ: 162.6, 152.2, 75.3, 62.9, 61.9, 41.7, 29.3, 27.4, 26.4, 26.2, 26.0, 14.3. FTIR (neat), cm^{-1}: 3469, 2924, 2853, 1730, 1449, 1369, 1304, 1275, 1197, 1156, 1096, 1084, 1031, 963, 890, 860, 616. HRMS (APCI): Calcd for $[C_{12}H_{21}NO_4+H]^+$: 244.1543, found: 244.1541.

α-Methoxyimino ester *E*-**3h**: TLC (30% ethyl acetate in hexane): R_f = 0.75. ^{1}H NMR (300 MHz, $CDCl_3$) δ: 4.33 (q, *J* = 7.1 Hz, 2H), 4.05 (s, 3H), 2.54 (tt, *J* = 11.1, 3.5 Hz, 1H), 2.01–1.89 (m, 2H), 1.87–1.75 (m, 2H), 1.72–1.63 (m, 1H), 1.41–1.21 (m, 8H). $^{13}C\{^1H\}$ NMR (126 MHz, $CDCl_3$) δ: 202.7, 160.6, 150.4, 64.2, 62.5, 50.1, 27.3, 25.9, 25.6, 14.2.

Synthesis of ethyl (*S*)-3-cyclohexyl-2-diazo-3-hydroxypropanoate 23

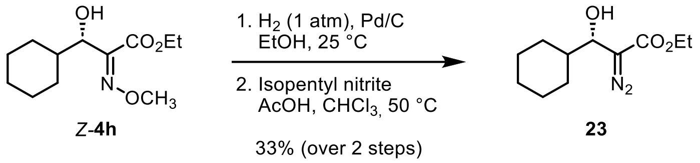

Palladium on charcoal (10% Pd basis; 48 mg) was added to a stirred solution of alcohol *Z*-**4h** (240 mg, 0.99 mmol, 1 equiv) in ethanol (6.0 mL) at 25 °C. After stirring of the suspension for 10 min, the reaction flask was evacuated, and a double layered balloon filled with hydrogen gas was attached. The mixture was vigorously stirred for 5 days at 25 °C (TLC: 30% ethyl acetate in hexane; ninhydrin). Filtration through a syringe PTFE filter and concentration of the filtrate in vacuo provided a crude amino alcohol (125 mg, not shown), which was used in the next step without further purification.

To a solution of the crude amino alcohol (125 mg, 0.58 mmol, 1 equiv [assumed]) in chloroform (4.0 mL) was added isopentyl nitrite (82 mg, 0.70 mmol, 1.2 equiv) and acetic acid (7 μL, 0.116 mmol, 0.2 equiv) at 25 °C, and the resulting mixture was stirred for 1 h at 40 °C (TLC: 30% ethyl acetate in hexane; ninhydrin). Then, a saturated aqueous solution of sodium hydrogen carbonate (30 mL) was added, and the resulting mixture was extracted with dichloromethane (3 × 30 mL). The organic phase was dried over anhydrous sodium sulfate, the dried solution was filtered, and the filtrate was concentrated in vacuo. The obtained residue was purified by FCC (20% ethyl acetate in hexane) to provide diazo ester **23** as a pale-yellow oil (43 mg, 33%).

Diazo ester **23**: TLC (30% ethyl acetate in hexane): R_f = 0.65. ^{1}H NMR (500 MHz, $CDCl_3$) δ: 4.32 (dd, *J* = 8.4, 3.1 Hz, 1H), 4.26 (qd, *J* = 7.1, 0.5 Hz, 2H), 2.43 (br s, 1H), 2.05 (dtd, *J* = 10.8, 3.3, 1.7 Hz, 1H), 1.85–1.73 (m, 2H), 1.73–1.63 (m, 2H), 1.63–1.54 (m, 1H), 1.31 (t, *J* = 7.1 Hz, 3H), 1.29–0.99 (m, 5H). $^{13}C\{^1H\}$ NMR (126 MHz, $CDCl_3$) δ: 166.8, 71.7, 62.9, 61.0, 42.3, 29.3, 26.4, 26.0, 25.8, 14.6. FTIR (neat), cm^{-1}: 3637, 3195, 2926, 2854, 2093, 1733, 1692, 1672, 1450, 1371, 1303, 1200, 1156, 1105, 1037, 892.

Synthesis of ethyl (*R*)-3-cyclohexyl-3-hydroxypropanoate 6h

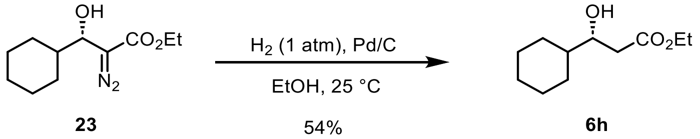

Palladium on charcoal (10% Pd basis; 8 mg) was added to a stirred solution of diazo ester **23** (40 mg, 0.23 mmol, 1 equiv) in ethanol (3 mL) at 25 °C. After stirring of the suspension for 10 min, the reaction flask was evacuated, and a double layered balloon filled with hydrogen gas was attached. The mixture was vigorously stirred for 45 min at 25 °C (TLC: 20% ethyl acetate in

hexane; $KMnO_4$). Filtration through a syringe PTFE filter, concentration of the filtrate in vacuo and subsequent purification by FCC (6% ethyl acetate in hexane) provided alcohol **6h** as a colorless oil (19 mg, 54%). NMR data of **6h** matched those obtained for the product of transfer hydrogenation of β-keto ester (see below). $[\alpha]_D^{23}$ = +17 (*c* 0.8, $CHCl_3$).

Synthesis of ethyl (*R*)-3-cyclohexyl-3-hydroxypropanoate 6h

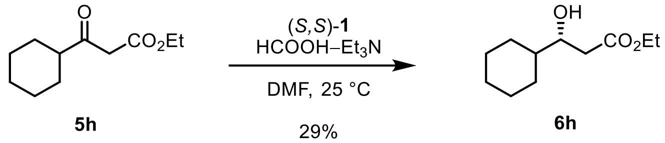

5h 29% **6h**

A solution of (*S,S*)-**1** (7.4 mg, 10.8 μmol, 0.02 equiv) in anhydrous *N,N*-dimethylformamide (1.0 mL) was evacuated and backfilled with argon (4 cycles). Then, keto ester **5h** (107 mg, 0.54 mmol, 1 equiv) was added as a solution in anhydrous *N,N*-dimethylformamide (0.5 mL), and the mixture was stirred for 5 min in order to obtain a clear solution. Argon was bubbled through the solution for 10 min (outlet needle), and a double-layered balloon filled with argon was attached. A mixture of formic acid and triethylamine (5:2 by volume, 0.27 mL) was added, followed by stirring for 6 days at 25 °C (TLC: 20% ethyl acetate in hexane, PMA). Then, cold water (20 mL) was added, and the resulting mixture was extracted with ethyl acetate (3 × 25 mL). The organic layer was washed with brine (20 mL), dried over anhydrous sodium sulfate, the dried solution was filtered, and the filtrate was concentrated in vacuo. The residue containing the product and unreacted starting material was purified by FCC (40% diethyl ether in hexane) to provide alcohol **6h** as a colorless oil (31 mg, 29%) and recovered starting material (45 mg, 42%). The enantiomer ratio of **6h** was determined by HPLC after its conversion to the 4-nitrobenzoyl ester (see below).

Alcohol **6h**: $[\alpha]_D^{23}$ = +6 (*c* 0.8, $CHCl_3$). TLC (20% ethyl acetate in hexane): R_f = 0.65. ^1H NMR (500 MHz, $CDCl_3$) δ: 4.17 (q, *J* = 7.2 Hz, 2H), 3.77 (ddd, *J* = 9.2, 6.1, 2.9 Hz, 1H), 2.84 (br s, 1H), 2.51 (dd, *J* = 16.3, 2.8 Hz, 1H), 2.41 (dd, *J* = 16.3, 9.5 Hz, 1H), 1.86 (dtt, *J* = 11.9, 3.5, 1.7 Hz, 1H), 1.76 (m, 2H), 1.66 (dtd, *J* = 10.7, 3.3, 1.8 Hz, 2H), 1.37 (m, 1H), 1.27 (m, 8H). $^{13}C\{^1H\}$ NMR (126 MHz, $CDCl_3$) δ: 173.7, 72.3, 60.8, 43.2, 38.8, 29.0, 28.4, 26.6, 26.3, 26.2, 14.3. FTIR (neat), cm^{-1}: 3472, 2981, 2924, 2853, 1718, 1449, 1402, 1372, 1309, 1279, 1260, 1164, 1129, 1098, 1026, 893. HRMS (APCI): Calcd for $[C_{12}H_{20}O_3+H]^+$: 201.1485, found: 201.1485.

Conversion of alcohol 6h to 4-nitrobenzoate 24

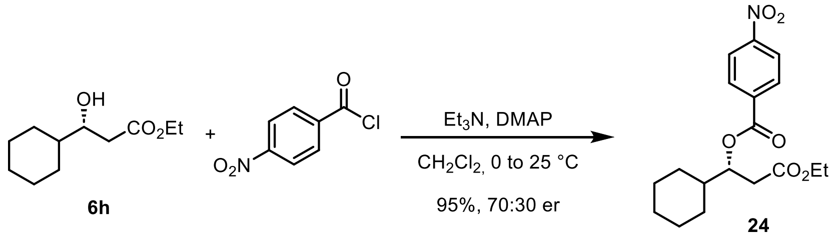

6h **24**

*Note: 4-Nitrobenzoyl derivative of alcohol **6h** was prepared for the purpose of enantiomer ratio determination using HPLC analysis.*

Triethylamine (58 µL, 0.42 mmol, 2.0 equiv) and 4-dimethylaminopyridine (2.6 mg, 21.3 µmol, 0.1 equiv) were added to a stirred solution of alcohol **6h** (42 mg, 0.21 mmol, 1 equiv) in anhydrous dichloromethane (1.2 mL) at 0 °C. After 5 min of stirring at 0 °C, 4-nitrobenzoyl chloride (78 mg, 0.42 mmol, 2.0 equiv) was slowly added as a solution in anhydrous dichloromethane (0.3 mL) at 0 °C. The resulting mixture was allowed to warm to 25 °C and stirred at this temperature for 16 h (TLC: 20% ethyl acetate in hexane). Then, water (10 mL) and dichloromethane (8.0 mL) were added. The mixture was extracted with dichloromethane (3 × 10 mL), the combined organic phases were washed with brine (10 mL), dried over anhydrous magnesium sulfate, and concentrated in vacuo. The obtained residue was purified by FCC (gradient elution with 10–12% ethyl acetate in hexane) to provide 4-nitrobenzoyl ester **24** as a colorless oil (69 mg, 95%). The enantiomeric purity of **24** was determined by HPLC (70:30 er).

4-Nitrobenzoyl ester **24**: TLC (20% ethyl acetate in hexane): R_f = 0.5. ^{1}H NMR (500 MHz, $CDCl_3$) δ: 8.32–8.25 (m, 2H), 8.23–8.17 (m, 2H), 5.42 (dt, *J* = 7.6, 5.5 Hz, 1H), 4.09 (qq, *J* = 6.9, 3.7 Hz, 2H), 2.95–2.52 (m, 2H), 1.96–1.64 (m, 6H), 1.41–1.03 (m, 8H). ^{13}C{^{1}H} NMR (126 MHz, $CDCl_3$) δ: 170.7, 164.2, 150.7, 136.0, 130.9, 123.7, 76.1, 60.9, 41.7, 37.0, 28.9, 28.3, 26.3, 26.1, 26.0, 14.3. FTIR (neat), cm^{-1}: 2928, 2854, 1723, 1607, 1526, 1449, 1347, 1316, 1269, 1189, 1172, 1114, 1014, 998, 872, 835, 782.

Enantioselective transfer hydrogenation of difluoroethyl substrate 3i

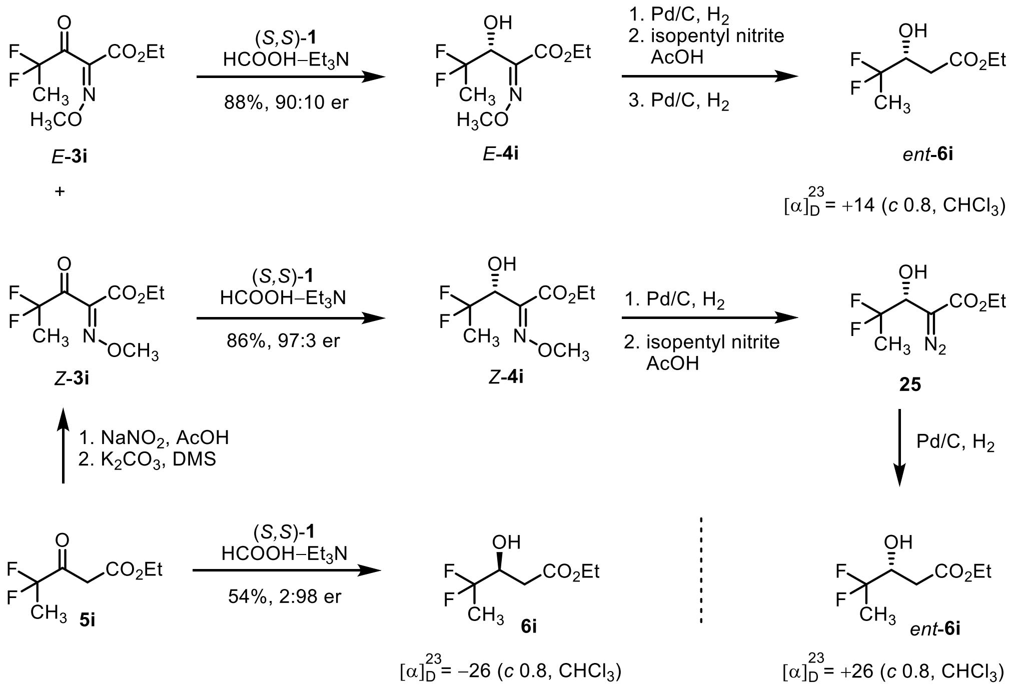

Synthesis of ethyl 4,4-difluoro-2-(methoxyimino)-3-oxopentanoate 3i

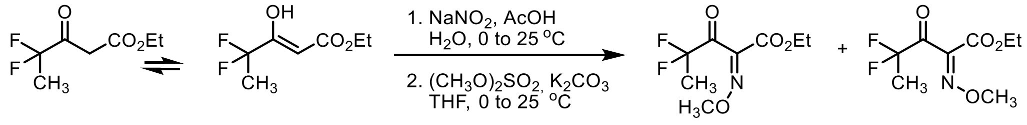

5i E-3i, 19% Z-3i, 59%

A solution of sodium nitrite (953 mg, 13.81 mmol, 1.3 equiv) in water (1.5 mL) was added dropwise to a solution of β-keto ester **5i**[3] (1.91 g, 10.6 mmol, 1 equiv) in acetic acid (5.0 mL) over 30 min at 0 °C. The resulting mixture was stirred at this temperature for 10 min, then for 45 min at 25 °C (TLC: 40% diethyl ether in hexane; UV, $KMnO_4$). The reaction mixture was poured into brine (25 mL) and extracted with ether (3 × 30 mL). The organic extracts were combined and washed with a saturated aqueous solution of sodium hydrogen carbonate (100 mL) to reach pH ~ 7, and the aqueous phase was extracted again with ether (3 × 30 mL). All organic extracts were combined, dried over anhydrous sodium sulfate, and filtered. The filtrate was concentrated under reduced pressure to yield the crude α-hydroxyimino ester (1.66 g, not shown), which was used in the next step without further purification.

Potassium carbonate (1.43 g, 10.3 mmol, 1.3 equiv) was added to a stirred solution of crude α-hydroxyimino ester (1.66 g, 8.0 mmol, 1 equiv) in tetrahydrofuran (25 mL) at 0 °C. After 5 min of stirring at 0 °C, dimethyl sulfate (0.68 mL, 7.2 mmol, 0.9 equiv) was added at 0 °C, and the resulting solution was allowed to warm to room temperature and stirred at this temperature for 18 h (TLC: 30% ethyl acetate in hexane, UV, $KMnO_4$). The reaction mixture was filtered, ice-cold brine (30 mL) was added, and the resulting mixture was extracted with dichloromethane (3 × 30 mL). The combined organic phases were dried over anhydrous sodium sulfate, the dried solution was filtered, and the filtrate was concentrated in vacuo. The obtained residue was purified by FCC (5% ethyl acetate in hexane) to provide separately *Z*-**3i** (1.04 g, 59%) and *E*-**3i** (0.34 g, 19%), both as yellow oils.

α-Methoxyimino ester *Z*-**3i**: TLC (20% ethyl acetate in hexane): R_f = 0.5, ^{1}H NMR (500 MHz, $CDCl_3$) δ: 4.36 (q, *J* = 7.1 Hz, 2H), 4.17 (s, 3H), 1.88 (t, *J* = 19.1 Hz, 3H), 1.35 (t, *J* = 7.1 Hz, 3H). $^{13}C\{^1H\}$ NMR (126 MHz, $CDCl_3$) δ: 183.8 (t, *J* = 32 Hz), 159.7, 146.8, 117.5 (t, *J* = 251 Hz), 65.3, 62.7, 21.8 (t, *J* = 25 Hz), 14.2. FTIR (neat), cm^{-1}: 2988, 2949, 1745, 1716, 1385, 1372, 1253, 1198, 1165, 1038, 942, 892, 858. HRMS (APCI): Calcd for $[C_8H_{11}F_2NO_4+H]^+$: 224.0729, found: 224.0731.

α-Methoxyimino ester *E*-**3i**: TLC (20% ethyl acetate in hexane): R_f = 0.7, ^{1}H NMR (500 MHz, $CDCl_3$) δ: 4.36 (q, *J* = 7.1 Hz, 2H), 4.11 (s, 3H), 1.77 (t, *J* = 19.0 Hz, 3H), 1.34 (t, *J* = 7.1 Hz, 3H). $^{13}C\{^1H\}$ NMR (126 MHz, $CDCl_3$) δ: 191.3 (t, *J* = 37 Hz), 159.4, 146.7, 116.9 (t, *J* = 250 Hz), 64.8, 63.0, 20.2 (t, *J* = 25 Hz), 14.1. FTIR (neat), cm^{-1}: 2985, 2923, 2852, 1750, 1715, 1376, 1330, 1256, 1197, 1163, 1055, 1017, 990, 944, 892. HRMS (APCI): Calcd for $[C_8H_{11}F_2NO_4+H]^+$: 224.0729, found: 224.0730.

Synthesis ethyl (*R, Z*)-4,4-difluoro-3-hydroxy-2-(methoxyimino)pentanoate *Z*-4i

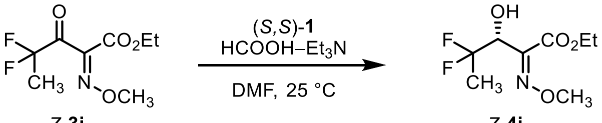

Z-3i 86%, 97:3 er Z-4i

A solution of (*S,S*)-**1** (6.3 mg, 9.9 µmol, 0.02 equiv) in anhydrous *N,N*-dimethylformamide (0.7 mL) was evacuated and backfilled with argon (4 cycles). Then, keto ester *Z*-**3i** (110 mg, 0.49

mmol, 1 equiv, tentatively *Z* isomer) was added as a solution in anhydrous *N*,*N*-dimethylformamide (0.4 mL), and the mixture was stirred for 5 min in order to obtain a clear solution. Then, argon was bubbled through the solution for 10 min (outlet needle), and a double-layered balloon filled with argon was attached. A mixture of formic acid and triethylamine (5:2 by volume, 0.25 mL) was added, followed by stirring for 16 h at 25 °C (TLC: 20% ethyl acetate in hexane, PMA). Then, cold water (10 mL) was added, and the resulting mixture was extracted with ethyl acetate (3 × 15 mL). The organic layer was washed with brine (10 mL), dried over anhydrous sodium sulfate, the dried solution was filtered, and the filtrate was concentrated in vacuo. The obtained residue was purified by FCC (gradient elution with 15–20% ethyl acetate in hexane) to provide alcohol *Z*-**4i** as a colorless oil (97 mg, 86%, tentatively *Z* isomer). The enantiomeric purity of *Z*-**4i** was determined by HPLC (97:3 er).

Alcohol *Z*-**4i**: $[\alpha]_D^{23}$ = −54.0 (*c* 0.8, $CHCl_3$). TLC (20% ethyl acetate in hexane): R_f= 0.4. ^{1}H NMR (500 MHz, $CDCl_3$) δ: 4.6 (ddd, *J* = 13.3, 7.1, 6.0 Hz, 1H), 4.33 (q, *J* = 7.1 Hz, 2H), 3.99 (s, 3H), 3.35 (d, *J* = 7.1 Hz, 1H), 1.69 (t, *J* = 18.9 Hz, 4H), 1.33 (t, *J* = 7.1 Hz, 3H). ^{13}C{^{1}H} NMR (126 MHz, $CDCl_3$) δ: 161.3, 146.1 (d, *J* = 3.8 Hz), 128.1–119.2 (m), 73.0 (dd, *J* = 31.5, 29.2 Hz), 63.7, 62.4, 19.8 (t, *J* = 25.7 Hz), 14.1. FTIR (neat), cm^{-1}: 3475, 2986, 2945, 2908, 1731, 1623, 1457, 1392, 1371, 1304, 1236, 1198, 1131, 1086, 1033, 929, 883, 860. HRMS (APCI): Calcd for $[C_8H_{13}F_2NO_4+H]^+$: 226.0885, found: 226.0887.

Synthesis ethyl (*R*, *E*)-4,4-difluoro-3-hydroxy-2-(methoxyimino)pentanoate *E*-4i

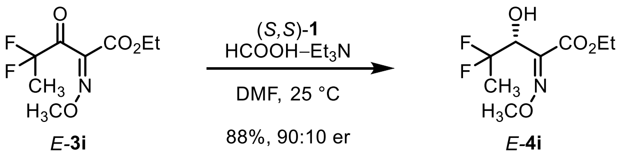

A solution of (*S,S*)-**1** (6.3 mg, 9.9 μmol, 0.02 equiv) in anhydrous *N*,*N*-dimethylformamide (0.7 mL) was evacuated and backfilled with argon (4 cycles). Then, keto ester *E*-**3i** (110 mg, 0.49 mmol, 1 equiv, tentatively *E* isomer) was added as a solution in anhydrous *N*,*N*-dimethylformamide (0.4 mL), and the mixture was stirred for 5 min in order to obtain a clear solution. Then, argon was bubbled through the solution for 10 min (outlet needle), and a double-layered balloon filled with argon was attached. A mixture of formic acid and triethylamine (5:2 by volume, 0.25 mL) was added, followed by stirring for 16 h at 25 °C (TLC: 20% ethyl acetate in hexane, PMA). Ice-cold water (10 mL) was added, and the resulting mixture was extracted with ethyl acetate (3 × 15 mL). The organic layer was washed with brine (10 mL), dried over anhydrous sodium sulfate, the dried solution was filtered, and the filtrate was concentrated in vacuo. The obtained residue was purified by FCC (gradient elution with 15–20% ethyl acetate in hexane) to provide alcohol *E*-**4i** as a colorless oil (95 mg, 88%, tentatively *E* isomer). The enantiomeric purity of *E*-**4i** was determined by HPLC (90:10 er).

Alcohol *E*-**4i**: $[\alpha]_D^{23}$ = +33 (*c* 1.0, $CHCl_3$). TLC (20% ethyl acetate in hexane): R_f= 0.4. ^{1}H NMR (500 MHz, $CDCl_3$) δ: 5.16 (ddd, *J* = 17.5, 11.6, 6.7 Hz, 1H), 4.91 (dd, *J* = 11.6, 0.9 Hz, 1H), 4.36 (qd, *J* = 7.1,

1.8 Hz, 2H), 4.11 (s, 3H), 1.68 (t, J = 18.8 Hz, 3H), 1.37 (t, J = 7.1 Hz, 3H). $^{13}C\{^1H\}$ NMR (126 MHz, $CDCl_3$) δ: 163.8, 145.7, 122.5 (dd, J = 246.8, 244.9 Hz), 69.3 (dd, J = 32.5, 27.4 Hz), 64.3, 62.6, 20.8 (t, J = 26.1 Hz), 14.1. FTIR (neat), cm^{-1}: 3466, 2986, 2947, 2912, 1732, 1447, 1389, 1376, 1330, 1266, 1235, 1200, 1133, 1089, 1070, 1039, 1015, 967, 933, 858, 833 HRMS (APCI): Calcd for $[C_8H_{13}F_2NO_4+H]^+$: 226.0885, found: 226.0887.

Synthesis of ethyl (*R*)-2-diazo-4,4-difluoro-3-hydroxypentanoate 25

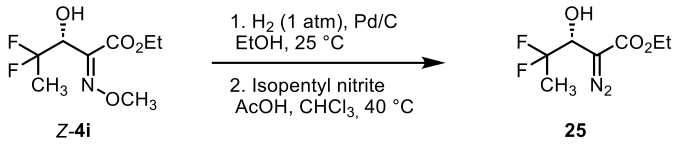

Palladium on charcoal (10% Pd basis; 80 mg) was added to a stirred solution of alcohol *Z*-**4i** (400 mg, 1.78 mmol, 1 equiv) in ethanol (4.0 mL) at 25 °C. After stirring of the suspension for 10 min, the reaction flask was evacuated, and a double layered balloon filled with hydrogen gas was attached. The mixture was vigorously stirred for 4 days at 25 °C (TLC: 30% ethyl acetate in hexane; ninhydrin). Filtration through a syringe PTFE filter and concentration of the filtrate in vacuo provided a crude amino alcohol (198 mg, not shown), which was used in the next step without further purification.

To a solution of the crude amino alcohol (198 mg, 0.10 mmol, 1 equiv [assumed]) in chloroform (4.5 mL) was added isopentyl nitrite (141 mg, 1.21 mmol, 1.2 equiv) and acetic acid (12 μL, 0.20 mmol, 0.2 equiv) at 25 °C, and the resulting mixture was warmed to 40 °C and stirred at this temperature for 1 h (TLC: 40% diethyl ether in hexane; ninhydrin). Then, a saturated aqueous solution of sodium hydrogen carbonate (20 mL) was added, and the resulting mixture was extracted with dichloromethane (3 × 25 mL). The organic phase was dried over anhydrous sodium sulfate, the dried solution was filtered, and the filtrate was concentrated in vacuo. The obtained residue was purified by FCC (gradient elution with 7–30% diethyl ether in hexane) to provide diazo ester **25** as a pale-yellow oil (104 mg, 50%). NMR data of **25** matched that of the same diazo ester prepared from the *E*-**4i**; denotated for clarity as diazo ester **25** (see below).

Synthesis of ethyl (*R*)-2-diazo-4,4-difluoro-3-hydroxypentanoate 25

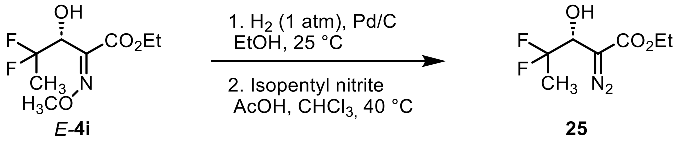

Palladium on charcoal (10% Pd basis; 53 mg) was added to a stirred solution of alcohol *E*-**4i** (264 mg, 1.17 mmol, 1 equiv) in ethanol (3.5 mL) at 25 °C. After stirring the suspension for 10 min, the reaction flask was evacuated, and a double layered balloon filled with hydrogen gas was attached. The mixture was vigorously stirred for 3 days at 25 °C (TLC: 30% ethyl acetate in hexane; ninhydrin). Filtration through a syringe PTFE filter and concentration of the filtrate in vacuo provided a crude amino alcohol (123 mg, not shown), which was used in the next step without further purification.

To a solution of the crude amino alcohol (123 mg, 0.62 mmol, 1 equiv [assumed]) in chloroform (4.0 mL) was added isopentyl nitrite (88 mg, 0.75 mmol, 1.2 equiv) and acetic acid (7 µL, 0.124 mmol, 0.2 equiv) at 25 °C, and the resulting mixture was warmed to 40 °C and stirred at this temperature for 1 h (TLC: 40% diethyl ether in hexane; ninhydrin). Then, a saturated aqueous solution of sodium hydrogen carbonate (30 mL) was added, and the resulting mixture was extracted with dichloromethane (3 × 30 mL), the organic phase was dried over anhydrous sodium sulfate, the dried solution was filtered, and the filtrate was concentrated in vacuo. The obtained residue was purified by FCC (gradient elution with 5–20% diethyl ether in hexane) to provide diazo ester **25** as a pale-yellow oil (80 mg, 61%).

Diazo ester **25**: TLC (40% diethyl ether in hexane): R_f = 0.4. ^{1}H NMR (500 MHz, $CDCl_3$) δ: 4.70 (td, *J* = 10.8, 5.4 Hz, 1H), 4.26 (qd, *J* = 7.1, 1.7 Hz, 2H), 3.23 (br s, 1H), 1.71 (t, *J* = 18.8 Hz, 3H), 1.29 (t, *J* = 7.1 Hz, 3H). $^{13}C\{^1H\}$ NMR (126 MHz, $CDCl_3$) δ: 166.2, 123.2 (t, *J* = 243.9 Hz), 69.2 (t, *J* = 29.7 Hz), 61.5, 20.4 (t, *J* = 26.1 Hz), 14.6. FTIR (neat), cm^{-1}: 3431, 2986, 2933, 2105, 1669, 1391, 1375, 1351, 1297, 1267, 1235, 1139, 1108, 1058, 1017, 928, 870, 810, 742, 605, 535. HRMS (APCI): Calcd for $[C_7H_{10}F_2N_2O_3+Cl]^-$: 243.0353, found: 243.0355.

Synthesis of ethyl (*R*)-4,4-difluoro-3-hydroxypentanoate *ent*-6i

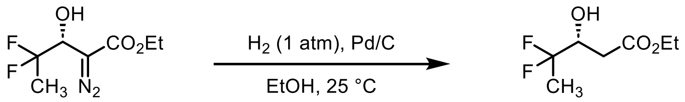

25 *ent*-**6i**

Palladium on charcoal (10% Pd basis; 17.2 mg) was added to a stirred solution of diazo ester **25** (86 mg, 0.41 mmol, 1 equiv, originating from *Z*-**4i**) in ethanol (3 mL) at 25 °C. After stirring of the suspension for 10 min, the reaction flask was evacuated, and a double layered balloon filled with hydrogen gas was attached. The mixture was vigorously stirred for 1 h at 25 °C (TLC: 30% ethyl acetate in hexane; $KMnO_4$). Filtration through a syringe PTFE filter and concentration of the filtrate in vacuo and subsequent purification by FCC (gradient elution with 7–10% ethyl acetate in hexane) provided alcohol *ent*-**6i** as a colorless oil (33 mg, 51%). NMR data of *ent*-**6i** matched those obtained for the product of transfer hydrogenation of β-keto ester (see below). $[\alpha]_D^{23}$ = +26 (*c* 0.8, $CHCl_3$).

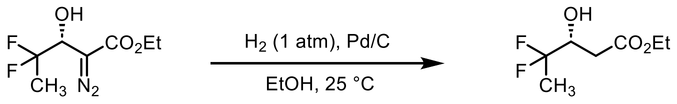

25 **6i**

Palladium on charcoal (10% Pd basis; 12.0 mg) was added to a stirred solution of diazo ester **25** (60 mg, 0.29 mmol, 1 equiv, originating from *E*-**4i**) in ethanol (3 mL) at 25 °C. After stirring of the suspension for 10 min, the reaction flask was evacuated, and a double layered balloon filled with hydrogen gas was attached. The mixture was vigorously stirred for 1 h at 25 °C (TLC: 30% ethyl acetate in hexane; $KMnO_4$). Filtration through a syringe PTFE filter, concentration of the filtrate in vacuo, and subsequent purification by FCC (7% ethyl acetate in hexane) provided

alcohol *ent*-**6i** as a colorless oil (25 mg, 48%). NMR data of *ent*-**6i** matched those obtained for the product of transfer hydrogenation of β-keto ester (see below). $[\alpha]_D^{23}$ = +14 (*c* 0.8, $CHCl_3$).

Synthesis of ethyl (*S*)-4,4-difluoro-3-hydroxypentanoate 6i

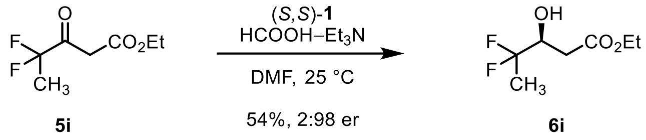

A solution of (*S,S*)-**1** (10.3 mg, 16.2 μmol, 0.02 equiv) in anhydrous *N,N*-dimethylformamide (1 mL) was evacuated and backfilled with argon (4 cycles). Then, β-keto ester **5i** (146 mg, 0.81 mmol, 1 equiv) was added as a solution in *N,N*-dimethylformamide (0.5 mL), and the mixture was stirred for 5 min in order to obtain a clear solution. Then, argon was bubbled through the solution for 10 min (outlet needle), and a double-layered balloon filled with argon was attached. A mixture of formic acid and triethylamine (5:2 by volume, 0.41 mL) was added, followed by stirring for 23 h at 25 °C (TLC: 30% ethyl acetate in hexane, $KMnO_4$). Then, cold water (25 mL) was added, and the resulting mixture was extracted with ethyl acetate (3 × 25 mL). The organic layer was washed with brine (20 mL), dried over anhydrous sodium sulfate, the dried solution was filtered, and the filtrate was concentrated in vacuo. The obtained residue (containing starting material and product) was purified by FCC (elution with 20% ethyl acetate in hexane) to provide alcohol **6i** as a colorless oil (80 mg, 54%). The enantiomeric purity of **6i** was determined by GC (2:98 er).

<u>Alcohol **6i**:</u> $[\alpha]_D^{23}$ = –26 (*c* 0.8, $CHCl_3$), TLC (30% ethyl acetate in hexane): R_f= 0.55. ^{1}H NMR (500 MHz, $CDCl_3$) δ 4.25 – 4.10 (m, 3H), 3.28 (br s, 1H), 2.73–2.52 (m, 2H), 1.66 (t, *J* = 19.0 Hz, 3H), 1.28 (t, *J* = 7.1 Hz, 3H). $^{13}C\{^1H\}$ NMR (126 MHz, $CDCl_3$) δ: 172.2, 123.0 (dd, *J* = 243.9, 240.2 Hz), 70.4 (dd, *J* = 32.2, 28.5 Hz), 61.3, 34.9, 19.74 (t, *J* = 26.4 Hz), 14.2. FTIR (neat), cm^{-1}: 3447, 2986, 2929, 1718, 1392, 1303, 1247, 1182, 1128, 1095, 1022, 919, 864. HRMS (APCI): Calcd for $[C_7H_{12}F_2O_3+H]^+$: 183.0827, found: 183.0830.

<u>Enantioselective transfer hydrogenation of *tert*-butyl substrate 3j</u>

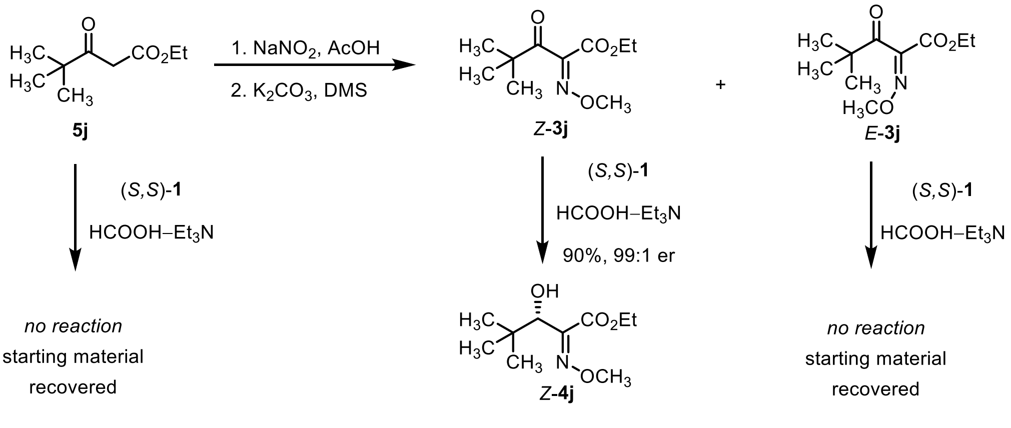

Synthesis of ethyl 2-(methoxyimino)-4,4-dimethyl-3-oxopentanoate 3j

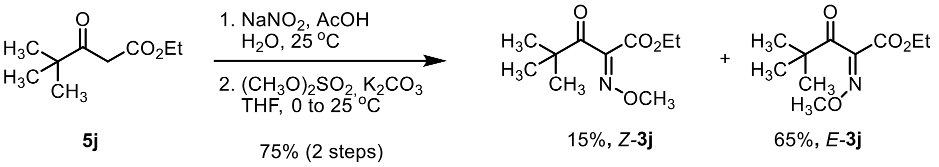

A solution of sodium nitrite (482 mg, 6.97 mmol, 1.2 equiv) in water (1.5 mL) was added dropwise to a solution of ethyl 4,4-dimethyl-3-oxopentanoate **5j** (1.00 g, 5.81 mmol, 1 equiv) in acetic acid (2.5 mL) over 30 min at 25 °C. The resulting mixture was stirred at this temperature for 2.5 h (TLC: 30% ethyl acetate in hexane; UV, PMA). Then, the mixture was poured into brine (80 mL) and extracted with ether (3 × 30 mL). The organic extracts were combined and washed with a saturated aqueous solution of sodium hydrogen carbonate (100 mL) to reach pH ~ 7, and the aqueous phase was extracted again with ether (3 × 30 mL). All organic extracts were combined, dried over anhydrous sodium sulfate, and filtered. The filtrate was concentrated under reduced pressure to yield the crude α-hydroxyimino ester (1.14 g, not shown), which was used in the next step without further purification.

Potassium carbonate (941 mg, 6.81 mmol, 1.2 equiv) was added to a stirred solution of crude α-hydroxyimino ester (1.14 g, 5.67 mmol, 1 equiv [assumed]) in anhydrous tetrahydrofuran (28 mL) at 0 °C. After 20 min of stirring at 0 °C, dimethyl sulfate (0.54 mL, 5.67 mmol, 1.0 equiv) was added at 0 °C, and the resulting solution was allowed to warm to room temperature and stirred at this temperature for 40 h (TLC: 20% ethyl acetate in hexane). The reaction mixture was filtered, ice-cold brine (80 mL) was added, and the resulting mixture was extracted with dichloromethane (3 × 80 mL). The combined organic phases were dried over anhydrous sodium sulfate, the dried solution was filtered, and the filtrate was concentrated in vacuo. The obtained residue was purified by FCC (gradient elution with 4–10% ethyl acetate in hexane) to provide (*Z*)-α-methoxyimino ester *Z*-**3j** as a colorless oil (187 mg, 15%) and (*E*)-α-methoxyimino ester *E*-**3j** as a colorless oil (749 mg, 65%).

α-Methoxyimino ester *Z*-**3j**: TLC (20% ethyl acetate in hexane): R_f = 0.65. ^{1}H NMR (300 MHz, $CDCl_3$) δ: 4.33 (q, *J* = 7.1 Hz, 2H), 4.07 (s, 3H), 1.33 (m, 12H). ^{13}C{^{1}H} NMR (126 MHz, $CDCl_3$) δ: 200.2, 161.5, 148.7, 64.2, 62.1, 44.8, 27.2, 14.2. FTIR (neat), cm^{-1}: 2978, 2941, 2907, 1741, 1721, 1695, 1592, 1471, 1464, 1391, 1371, 1318, 1274, 1254, 1194, 1151, 1051, 1028, 965, 921, 864, 824, 792,731. HRMS (APCI): Calcd for $[C_{10}H_{17}NO_4+H]^+$: 216.1230, found: 216.1232.

α-Methoxyimino ester *E*-**3j**: TLC (20% ethyl acetate in hexane): R_f = 0.55. ^{1}H NMR (500 MHz, $CDCl_3$) δ: 4.33 (q, *J* = 7.1 Hz, 2H), 4.04 (s, 3H), 1.33 (t, *J* = 7.1 Hz, 3H), 1.18 (s, 9H).^{13}C{^{1}H} NMR (126 MHz, $CDCl_3$) δ: 207.7, 160.4, 150.8, 64.0, 62.5, 43.5, 26.3, 14.2. FTIR (neat), cm^{-1}: 2976, 2942, 2908, 1748, 1720, 1685, 1597, 1479, 1463, 1394, 1370, 1313, 1276, 1252, 1199, 1150, 1055, 1029, 964, 920, 863, 825, 791, 732. HRMS (APCI): Calcd for $[C_{10}H_{17}NO_4+H]^+$: 216.1230, found: 216.1233.

Synthesis of ethyl (*S, Z*)-3-hydroxy-2-(methoxyimino)-4,4-dimethylpentanoate *Z*-4j

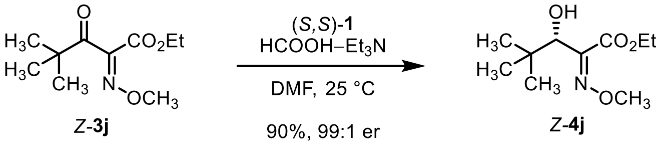

A solution of (*S,S*)-**1** (23.5 mg, 23.7 µmol, 0.02 equiv) in anhydrous *N,N*-dimethylformamide (1.5 mL) was evacuated and backfilled with argon (4 cycles). Then, β-keto ester *Z*-**3j** (400 mg, 1.86 mmol, 1 equiv, *Z* isomer) was added as a solution in anhydrous *N,N*-dimethylformamide (0.6 mL), and the mixture was stirred for 5 min in order to obtain a clear solution. Then, argon was bubbled through the solution for 10 min (outlet needle), and a double-layered balloon filled with argon was attached. A mixture of formic acid and triethylamine (5:2 by volume, 0.25 mL) was added, followed by stirring for 16 h at 25 °C (TLC: 20% ethyl acetate in hexane, PMA). Then, cold water (10 mL) was added, and the resulting mixture was extracted with ethyl acetate (3 × 15 mL). The organic layer was washed with brine (10 mL), dried over anhydrous sodium sulfate, the dried solution was filtered, and the filtrate was concentrated in vacuo. The obtained residue (containing starting material and product) was purified by FCC (gradient elution with 15–20% ethyl acetate in hexane) to provide alcohol *Z*-**4j** as a colorless oil (365 mg, 90%, *Z* isomer). The enantiomeric purity of *Z*-**4j** was determined by HPLC (99:1 er).

<u>Alcohol *Z*-**4j**</u>: $[\alpha]_D^{23}$ = −22 (*c* 1.0, $CHCl_3$). TLC (20% ethyl acetate in hexane): R_f = 0.5. ^{1}H NMR (500 MHz, $CDCl_3$) δ: 4.30 (q, *J* = 7.2 Hz, 2H), 4.14 (d, *J* = 6.1 Hz, 1H), 3.91 (s, 3H), 2.76 (d, *J* = 6.2 Hz, 1H), 1.33 (t, *J* = 7.2 Hz, 3H), 0.96 (s, 9H). ^{13}C{^{1}H} NMR (126 MHz, $CDCl_3$) δ: 163.1, 151.6, 78.7, 63.1, 61.9, 36.1, 25.8, 14.1. FTIR (neat), cm^{-1}: 3526, 2958, 2942, 2906, 2874, 1728, 1617, 1464, 1367, 1288, 1196, 1151, 1036, 1015, 926, 883, 734, 610. HRMS (APCI): Calcd for $[C_{10}H_{19}NO_4+H]^+$: 218.1387, found: 218.1387.

Enantioselective transfer hydrogenation of phenyl substrate 3k

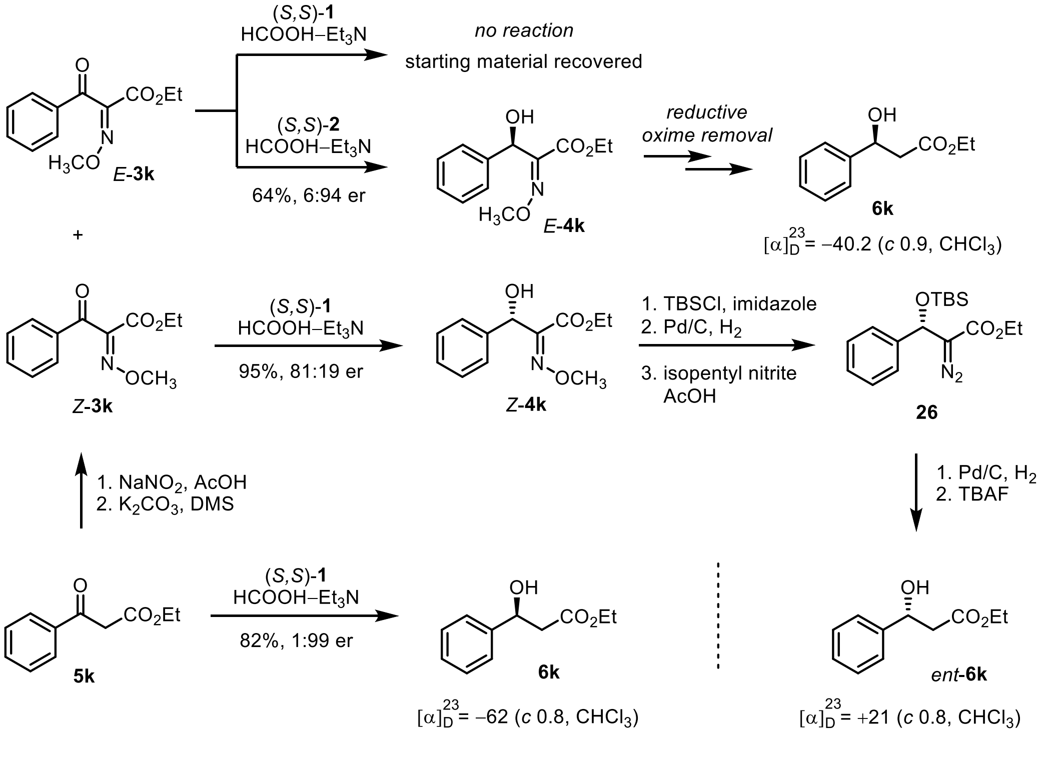

Synthesis of ethyl 2-(methoxyimino)-3-oxo-3-phenylpropanoate 3k

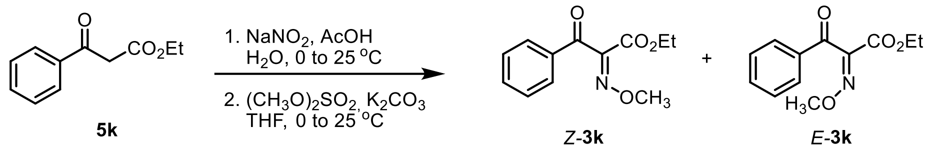

A solution of sodium nitrite (713 mg, 10.34 mmol, 1.3 equiv) in water (1.7 mL) was added dropwise to a solution of β-keto ester **5k** (1.52 g, 8.0 mmol, 1 equiv) in acetic acid (3.0 mL) over 30 min at 0 °C. The resulting mixture was stirred at this temperature for 10 min and then for 40 min at 25 °C (TLC: 30% ethyl acetate in hexane; UV). The reaction mixture was poured into brine (25 mL) and extracted with ether (3 × 20 mL). The organic extracts were combined and washed with a saturated aqueous solution of sodium hydrogen carbonate (100 mL) to reach pH ~ 7, and the aqueous phase was extracted again with ether (3 × 20 mL). All organic extracts were combined, dried over anhydrous sodium sulfate, and filtered. The filtrate was concentrated under reduced pressure to yield the crude α-hydroxyimino ester (**7**, 1.67 g, not shown, mixture of *E*-*Z* isomers*), which was used in the next step without further purification.

Potassium carbonate (1.36 g, 9.8 mmol, 1.3 equiv) was added to a stirred solution of crude α-hydroxyimino ester (see above, 1.67 g, 7.6 mmol, 1.0 equiv, mixture of *E*-*Z* isomers) in anhydrous tetrahydrofuran (20.0 mL) at 0 °C. After 5 min of stirring at 0 °C, dimethyl sulfate (0.86 mL, 9.1 mmol, 1.2 equiv) was added at 0 °C, and the resulting solution was allowed to warm to room temperature and stirred at this temperature for 17 h (TLC: 30% ethyl acetate in hexane, UV). The reaction mixture was filtered, ice-cold brine (30 mL) was added, and the resulting mixture was extracted with dichloromethane (3 × 30 mL). The combined organic phases were dried over anhydrous sodium sulfate, the dried solution was filtered, and the filtrate was concentrated in vacuo. The obtained residue was purified by FCC (10% ethyl acetate in hexane) to provide (*Z*)-α-methoxyimino ester *Z*-**3k** as a yellow oil (229 mg, 13%) and (*E*)-α-methoxyimino ester *E*-**3k** as a yellow oil (914 mg, 52%).

<u>α-Methoxyimino ester *Z*-**3k**:</u> TLC (30% ethyl acetate in hexane): R_f = 0.70. ^{1}H NMR (500 MHz, $CDCl_3$) δ: 8.05 (dd, *J* = 8.5, 1.3 Hz, 2H), 7.60 (t, *J* = 7.4 Hz, 1H), 7.47 (t, *J* = 7.9 Hz, 2H), 4.40 (q, *J* = 7.2 Hz, 2H), 4.13 (s, 3H), 1.37 (t, *J* = 7.2 Hz, 3H). ^{13}C{^{1}H} NMR (126 MHz, $CDCl_3$) δ: 186.7, 161.2, 149.8, 135.6, 133.7, 130.6, 128.4, 64.6, 62.3, 14.2. FTIR (neat), cm^{-1}: 2985, 2942, 1743, 1656, 1598, 1448, 1368, 1328, 1246, 1137, 1035, 955, 893, 859, 731, 690, 658. HRMS (APCI): Calcd for $[C_{12}H_{13}NO_4+H]^+$: 236.0917, found: 236.0917.

<u>α-Methoxyimino ester *E*-**3k**:</u> TLC (30% ethyl acetate in hexane): R_f = 0.60. ^{1}H NMR (500 MHz, $CDCl_3$) δ: 7.85 (d, *J* = 7.0 Hz, 2H), 7.63 (m, 1H), 7.51 (m, 2H), 4.33 (q, *J* = 7.1 Hz, 2H), 4.02 (s, 3H), 1.28 (t, *J* = 7.1 Hz, 3H). ^{13}C{^{1}H} NMR (126 MHz, $CDCl_3$) δ: 189.9, 160.5, 149.2, 134.7, 134.4, 129.2, 129.2, 64.4, 62.6, 14.2 FTIR (neat), cm^{-1}: 2985, 2942, 1743, 1656, 1596, 1448, 1368, 1328, 1246,

*In another experiment, the *E* and *Z* isomers of α-hydroxyimino ester **7** were separated (gradient elution with 5–20% ethyl acetate in hexane) and methylated separately. Crystals of the *E* oxime (*E*-**7**, prior to methylation) suitable for X-ray analysis were obtained by slow evaporation from a 1:1 mixture of tetrahydrofuran and methanol.

1137, 1035, 955, 893, 859, 731, 690, 658. HRMS (APCI): Calcd for $[C_{12}H_{13}NO_4+H]^+$: 236.0917, found: 236.0917.

α-Hydroxyimino ester *E*-**7**: TLC (30% ethyl acetate in hexane): R_f= 0.30. ^{1}H NMR (500 MHz, $CDCl_3$) δ: 9.16 (s, 1H), 7.98–7.78 (m, 2H), 7.71–7.61 (m, 1H), 7.52 (t, *J* = 7.8 Hz, 2H), 4.30 (q, *J* = 7.2 Hz, 2H), 1.25 (t, *J* = 7.1 Hz, 3H). ^{13}C{^{1}H} NMR (126 MHz, $CDCl_3$) δ: 189.8, 160.7, 150.1, 134.9, 134.3, 129.3, 129.2, 32.8, 14.1.

Synthesis of ethyl (*S, Z*)-3-hydroxy-2-(methoxyimino)-3-phenylpropanoate *Z*-4k

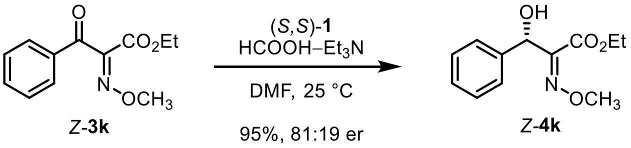

A solution of (*S,S*)-**1** (11.5 mg, 18.1 μmol, 0.02 equiv) in anhydrous *N,N*-dimethylformamide (1.0 mL) was evacuated and backfilled with argon (4 cycles). Then, α-methoxyimino ester *Z*-**3k** (212 mg, 0.91 mmol, 1 equiv, *Z* isomer) was added as a solution in anhydrous *N,N*-dimethylformamide (1.0 mL), and the mixture was stirred for 5 min in order to obtain a clear solution. Then, argon was bubbled through the solution for 15 min (outlet needle), and a double-layered balloon filled with argon was attached. A mixture of formic acid and triethylamine (5:2 by volume, 0.46 mL) was added, followed by stirring for 20 h at 25 °C (TLC: 30% ethyl acetate in hexane, UV, PMA). Then, ice-cold water (30 mL) was added, and the resulting mixture was extracted with ethyl acetate (3 × 20 mL). The combined organic phases were washed with brine (20 mL), dried over anhydrous sodium sulfate, the dried solution was filtered, and the filtrate was concentrated in vacuo. The obtained residue was purified by FCC (50% ethyl acetate in hexane) to provide alcohol *Z*-**4k** as a colorless oil (202 mg, 95%, *Z* isomer). The enantiomeric purity of *Z*-**4k** was determined by HPLC (81:19 er). For the analytical data of *Z*-**4k**, see below.

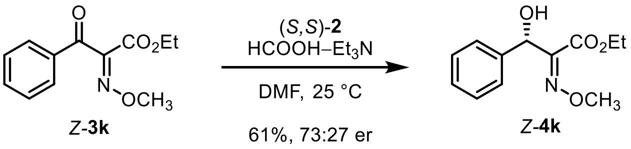

A solution of Wills catalyst (*S,S*)-**2** (2.9 mg, 4.7 μmol, 0.02 equiv) in anhydrous *N,N*-dimethylformamide (1.0 mL) was evacuated and backfilled with argon (4 cycles). Then, α-methoxyimino ester *Z*-**3k** (55 mg, 0.23 mmol, 1 equiv, *Z* isomer) was added as a solution in anhydrous *N,N*-dimethylformamide (0.5 mL), and the mixture was stirred for 5 min in order to obtain a clear solution. Then, argon was bubbled through the solution for 15 min (outlet needle), and a double-layered balloon filled with argon was attached. A mixture of formic acid and triethylamine (5:2 by volume, 0.11 mL) was added, followed by stirring for 1 h at 25 °C (TLC: 20% ethyl acetate in hexane, UV, PMA). Then, ice-cold water (25 mL) was added, and the resulting mixture was extracted with ethyl acetate (3 × 25 mL). The combined organic phases were washed with brine (25 mL), dried over anhydrous sodium sulfate, the dried solution was filtered, and the filtrate was concentrated in vacuo. The obtained residue was purified by FCC (15% ethyl acetate

in hexane) to provide alcohol *Z*-**4k** as a colorless oil (34 mg, 61%, *Z* isomer). The enantiomeric purity of *Z*-**4k** was determined by HPLC (73:27 er).

<u>Alcohol *Z*-**4k**</u>: $[\alpha]_D^{23}$ = +27 (*c* 0.86, $CHCl_3$). TLC (30% ethyl acetate in hexane): R_f = 0.53. ^{1}H NMR (500 MHz, $CDCl_3$) δ: 7.36 (m, 5H), 5.52 (d, *J* = 5.0 Hz, 1H), 4.14 (q, *J* = 7.1 Hz, 2H), 3.98 (s, 3H), 3.26 (d, *J* = 5.0 Hz, 1H), 1.10 (t, *J* = 7.2 Hz, 3H). $^{13}C\{^1H\}$ NMR (126 MHz, $CDCl_3$) δ: 161.6, 151.1, 138.6, 128.7, 128.7, 126.9, 73.1, 63.3, 61.8, 14.0. FTIR (neat), cm^{-1}: 3474, 2982, 2940, 2903, 1731, 1454, 1305, 1196, 1151, 1196, 1151, 1035, 701. HRMS (APCI): Calcd for $[C_{12}H_{15}NO_4+H]^+$: 238.1074, found: 238.1075.

Synthesis of ethyl (*R, E*)-3-hydroxy-2-(methoxyimino)-3-phenylpropanoate *E*-4k

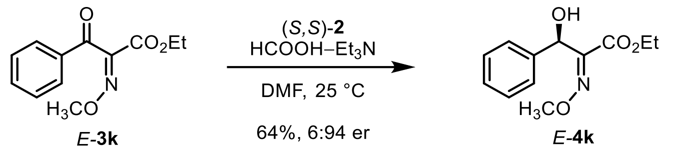

A solution of Wills catalyst (*S,S*)-**2** (6.1 mg, 9.8 μmol, 0.02 equiv) in *N,N*-dimethylformamide (1.0 mL) was evacuated and backfilled with argon (4 cycles). Then, α-methoxyimino ester *E*-**3k** (115 mg, 0.49 mmol, 1 equiv, *E* isomer) was added as a solution in *N,N*-dimethylformamide (0.5 mL), and the mixture was stirred for 5 min in order to obtain a clear solution. Then, argon was bubbled through the solution for 15 min (outlet needle), and a double-layered balloon filled with argon was attached. A mixture of formic acid and triethylamine (5:2 by volume, 0.25 mL) was added, followed by stirring for 6 days at 25 °C (TLC: 30% ethyl acetate in hexane, UV, PMA). Then, ice-cold water (25 mL) was added, and the resulting mixture was extracted with ethyl acetate (3 × 30 mL). The combined organic phases were washed with brine (25 mL), dried over anhydrous sodium sulfate, the dried solution was filtered, and the filtrate was concentrated in vacuo. The obtained residue was purified by FCC (gradient elution with 7–10% ethyl acetate in hexane) to provide alcohol *E*-**4k** as a colorless oil (74 mg, 64%, *E* isomer). The enantiomeric purity of *E*-**4k** was determined by HPLC (6:94 er).

<u>Alcohol *E*-**4k**</u>: TLC (20% ethyl acetate in hexane): R_f = 0.48. ^{1}H NMR (500 MHz, $CDCl_3$) δ: 7.42–7.32 (m, 4H), 7.30–7.26 (m, 1H), 6.12 (d, *J* = 11.5 Hz, 1H), 4.36–4.26 (m, 2H), 4.22 (d, *J* = 11.6 Hz, 1H), 4.10 (s, 3H), 1.32 (t, *J* = 7.1 Hz, 3H). $^{13}C\{^1H\}$ NMR (126 MHz, $CDCl_3$) δ: 163.4, 151.4, 140.0, 128.7, 127.9, 125.7, 68.4, 63.9, 62.4, 14.2. FTIR (neat), cm^{-1}: 3474, 2982, 2940, 2903, 1731, 1454, 1305, 1196, 1151, 1196, 1151, 1035, 701. HRMS (APCI): Calcd for $[C_{12}H_{15}NO_4+H]^+$: 238.1074, found: 238.1075.

Synthesis of ethyl (*S, Z*)-3-((*tert*-butyldimethylsilyl)oxy)-2-(methoxyimino)-3-phenylpropanoate 27

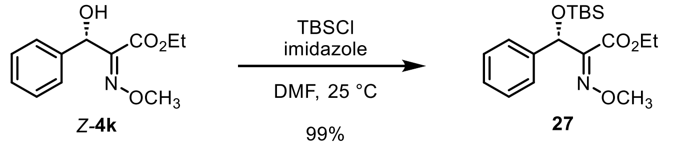

Imidazole (145 mg, 2.13 mmol, 2.5 equiv) was added to a solution of alcohol *Z*-**4k** (201 mg, 0.85 mmol, 1 equiv) in *N*,*N*-dimethylformamide (0.7 mL) at 25 °C under argon atmosphere. After 5 min, *tert*-butyldimethylsilyl chloride (167 mg, 1.11 mmol, 1.3 equiv) was added, and the resulting mixture was stirred for 18 h at 25 °C. The reaction was quenched by the addition of ice-cold brine (25 mL). The resulting mixture was then extracted with ethyl acetate (3 × 25 mL). The combined organic phases were dried over anhydrous sodium sulfate, the dried solution was filtered, and the filtrate was concentrated in vacuo. The obtained residue was purified by FCC (8% ethyl acetate in hexane) to provide silylated alcohol **27** as a grey oil (299 mg, 99%).

Silylated alcohol **27**: TLC (10% ethyl acetate in hexane): R_f= 0.6. ^{1}H NMR (500 MHz, $CDCl_3$) δ: 7.33 (m, 5H), 5.49 (s, 1H), 4.22 (dq, *J* = 10.7, 7.2 Hz, 1H), 4.12 (dq, *J* = 10.8, 7.2 Hz, 1H), 3.88 (s, 3H), 1.18 (t, *J* = 7.2 Hz, 3H), 0.92 (s, 9H), 0.14 (s, 3H), 0.03 (s, 3H). ^{13}C{^{1}H} NMR (126 MHz, $CDCl_3$) δ: 161.9, 153.7, 140.0, 128.2, 127.9, 126.3, 73.6, 62.7, 61.3, 25.8, 18.4, 14.2, −4.8, −4.9. FTIR (neat), cm^{-1}: 2956, 2932, 2898, 2858, 1739, 1464, 1365, 1299, 1257, 1195, 1155, 1095, 1068, 1039, 920, 869, 837, 779, 743, 705, 672, 594.

Synthesis of ethyl (*S*)-3-((*tert*-butyldimethylsilyl)oxy)-2-diazo-3-phenylpropanoate 26

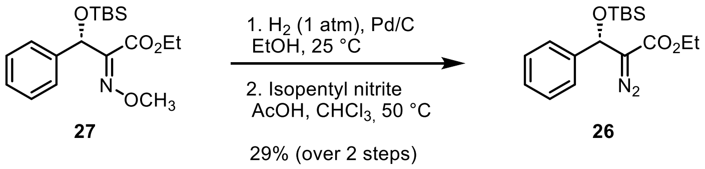

Palladium on charcoal (20% Pd basis; 48 mg) was added to a stirred solution of protected alcohol **27** (335 mg, 0.96 mmol, 1 equiv) in ethanol (4.5 mL) at 25 °C. After stirring of the suspension for 10 min, the reaction flask was evacuated, and a double layered balloon filled with hydrogen gas was attached. The mixture was vigorously stirred for 5 days at 25 °C (TLC: 30% ethyl acetate in hexane; ninhydrin). Filtration through a syringe PTFE filter and concentration of the filtrate in vacuo provided a crude amino alcohol (275 mg, not shown), which was used in the next step without further purification.

To a solution of the crude amino alcohol (275 mg, 0.85 mmol, 1 equiv [assumed]) in chloroform (4.5 mL) was added isopentyl nitrite (120 mg, 1.02 mmol, 1.2 equiv) and acetic acid (10 μL, 0.173 mmol, 0.2 equiv) at 25 °C, and the resulting mixture was stirred at 50 °C for 3 h (TLC: 10% ethyl acetate in hexane; ninhydrin). Then, a saturated aqueous solution of sodium hydrogen carbonate (30 mL) was added, and the resulting mixture was extracted with dichloromethane (3 × 30 mL), the organic phase was dried over anhydrous sodium sulfate, the dried solution was filtered, and the filtrate was concentrated in vacuo. The obtained residue was purified by FCC (gradient elution with 1–2% ethyl acetate in hexane) to provide diazo ester **26** as a pale-yellow oil (91 mg, 29% over two steps).

Diazo ester **26**: TLC (5% ethyl acetate in hexane; ninhydrin): R_f = 0.6. ^{1}H NMR (500 MHz, $CDCl_3$) δ: 7.34 (m, 5H), 5.82 (s, 1H), 4.27 (m, 2H), 1.29 (t, *J* = 7.2 Hz, 3H), 0.92 (s, 9H), 0.12 (s, 3H), 0.06 (s, 3H). ^{13}C{^{1}H} NMR (126 MHz, $CDCl_3$) δ: 141.7, 128.6, 127.9, 125.5, 68.9, 61.0, 25.9, 18.4, 14.7, −4.9, −5.1. FTIR (neat), cm^{-1}: 2956, 2931, 2896, 2858, 2096, 1693, 1372, 1292, 1253, 1109, 1080, 1069, 854, 779, 719.

Synthesis of ethyl (*R*)-3-((*tert*-butyldimethylsilyl)oxy)-3-phenylpropanoate 28

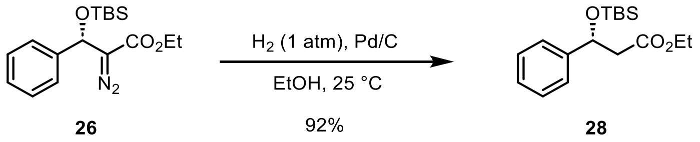

Palladium on charcoal (10% Pd basis; 10.2 mg) was added to a stirred solution of diazo ester **26** (52 mg, 0.16 mmol, 1 equiv) in ethanol (2 mL) at 25 °C. After stirring of the suspension for 10 min, the reaction flask was evacuated, and a double layered balloon filled with hydrogen gas was attached. The mixture was vigorously stirred for 1.0 h at 25 °C (TLC: 10% ethyl acetate in hexane; $KMnO_4$). Filtration through a syringe PTFE filter, concentration of the filtrate in vacuo, and subsequent purification by FCC (5% ethyl acetate in hexane) provided the protected β-hydroxy ester **28** as a colorless oil (44 mg, 92%).

β-Hydroxy ester **28**: TLC (5% ethyl acetate in hexane; PMA, $KMnO_4$): R_f = 0.45. ^{1}H NMR (500 MHz, $CDCl_3$) δ: 7.32 (m, 5H), 5.15 (dd, *J* = 9.2, 4.1 Hz, 1H), 4.13 (m, 2H), 2.72 (dd, *J* = 14.6, 9.2 Hz, 1H), 2.54 (dd, *J* = 14.5, 4.1 Hz, 1H), 1.25 (t, *J* = 7.2 Hz, 3H), 0.85 (s, 9H), 0.02 (s, 3H), −0.17 (s, 3H). ^{13}C{^{1}H} NMR (126 MHz, $CDCl_3$) δ: 171.3, 144.3, 128.4, 127.6, 126.0, 72.4, 60.6, 46.7, 25.8, 18.2, 14.3, −4.5, −5.1. FTIR (neat), cm^{-1}: 2956, 2929, 2897, 2857, 1737, 1694, 1472, 1371, 1296, 1254, 1198, 1163, 1092, 1050, 1027, 955, 835, 812, 778, 669.

Synthesis of ethyl (*R*)-3-hydroxy-3-phenylpropanoate *ent*-6k

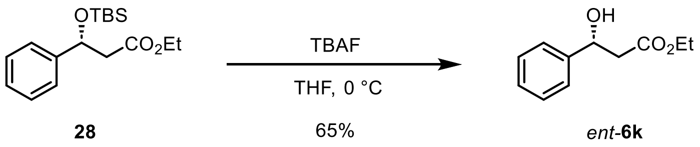

A solution of protected β-hydroxy ester **28** (41 mg, 0.13 mmol, 1 equiv) in tetrahydrofuran (4 mL) was cooled down to 0 °C and stirred at this temperature for 5 min. Then, a solution of tetrabutylammonium fluoride in tetrahydrofuran (1M, 0.16 mL, 0.16 mmol, 1.2 equiv) was added. The resulting mixture was stirred for 45 min at 0 °C, and then was concentrated in vacuo. The residue was purified by FCC (gradient elution with 15–20% ethyl acetate in hexane) to provide β-hydroxy ester *ent*-**6k** as a colorless oil (17 mg, 65 %). NMR data of *ent*-**6k** matched those obtained for the product of transfer hydrogenation of β-keto ester (see below). $[\alpha]_D^{23}$ = +21 (*c* 0.8, $CHCl_3$)

Synthesis of ethyl (*S*)-3-hydroxy-3-phenylpropanoate 6k

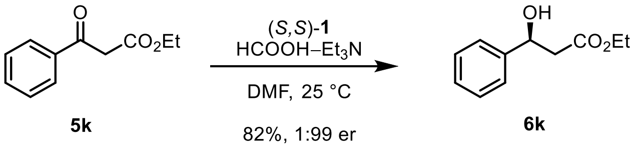

5k 6k

A solution of (*S,S*)-**1** (18.1 mg, 28.5 μmol, 0.02 equiv) in *N,N*-dimethylformamide (1 mL) was evacuated and backfilled with argon (4 cycles). Then, β-keto ester **5k** (271 mg, 1.42 mmol, 1.0 equiv) was added as a solution in *N,N*-dimethylformamide (0.5 mL), and the mixture was stirred for 5 min in order to obtain a clear solution. Then, argon was bubbled through the solution for 10 min (outlet needle), and a double-layered balloon filled with argon was attached. A mixture of formic acid and triethylamine (5:2 by volume, 0.71 mL) was added, followed by stirring for 16 h at 25 °C (TLC: 25% ethyl acetate in hexane). Then, ice-cold water (25 mL) was added, and the resulting mixture was extracted with ethyl acetate (3 × 20 mL). The organic layer was washed with brine (25 mL), dried over anhydrous sodium sulfate, the dried solution was filtered, and the filtrate was concentrated in vacuo. The obtained residue was purified by FCC (gradient elution with 10–20% ethyl acetate in hexane) to provide alcohol **6k** as a colorless oil (225 mg, 82%). The enantiomeric purity of **6k** was determined by HPLC (1:99 er).

Alcohol **6k**: $[\alpha]_D^{23}$ = –62 (*c* 0.8, $CHCl_3$) TLC (30% ethyl acetate in hexane; PMA): R_f = 0.5. ^1H NMR (500 MHz, $CDCl_3$) δ: 7.35 (m, 5H), 5.14 (dt, *J* = 8.9, 3.5 Hz, 1H), 4.19 (q, *J* = 7.2 Hz, 2H), 3.26 (d, *J* = 3.4 Hz, 1H), 2.74 (m, 2H), 1.26 (t, *J* = 7.2 Hz, 3H). $^{13}C\{^1H\}$ NMR (126 MHz, $CDCl_3$) δ: 172.5, 142.7, 128.7, 127.9, 125.8, 70.5, 61.0, 43.5, 14.3. FTIR (neat), cm^{-1}: 3466, 2982, 1728, 1495, 1454, 1372, 1297, 1267, 1195, 1160, 1082, 1038, 761, 700, 609, 541. HRMS (APCI): Calcd for $[C_{11}H_{14}O_3+H]^+$: 195.1016, found: 195.1019.

Enantioselective transfer hydrogenation of *ortho*-fluorophenyl substrate 3l

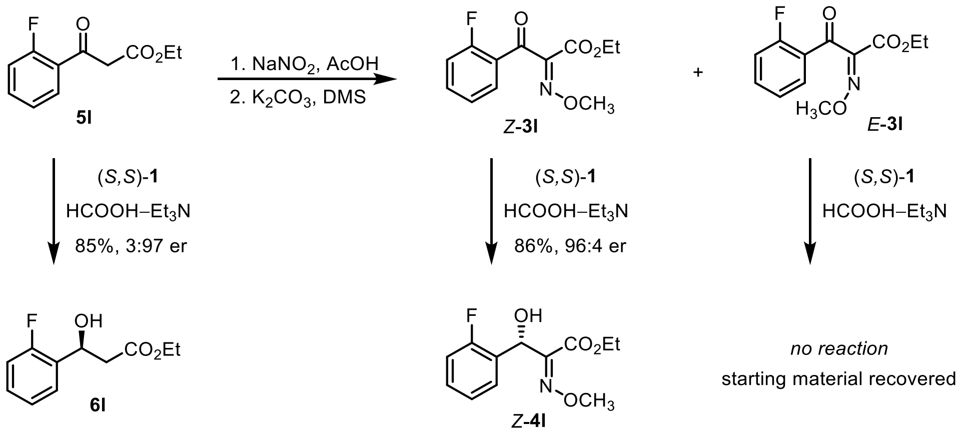

Synthesis of ethyl 3-(2-fluorophenyl)-2-(methoxyimino)-3-oxopropanoate 3l

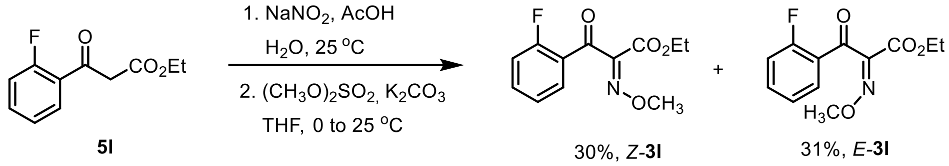

A solution of sodium nitrite (787 mg, 11.41 mmol, 1.2 equiv) in water (3.0 mL) was added dropwise to a solution of ethyl 3-(2-fluorophenyl)-3-oxopropanoate **5l** (2.00 g, 9.51 mmol, 1 equiv) in acetic acid (4.0 mL) over 40 min at 25 °C. The resulting mixture was stirred at this temperature for 2.0 h (TLC: 30% ethyl acetate in hexane; UV, PMA). Then, the mixture was poured into brine (80 mL) and extracted with ether (3 × 40 mL). The organic extracts were combined and washed with a saturated aqueous solution of sodium hydrogen carbonate (120 mL) to reach pH ~ 7, and the aqueous phase was extracted again with ether (3 × 40 mL). All organic extracts were combined, dried over anhydrous sodium sulfate, and filtered. The filtrate was concentrated under reduced pressure to yield the crude α-hydroxyimino ester (2.15 g, not shown), which was used in the next step without further purification.

Potassium carbonate (1.61 g, 11.7 mmol, 1.3 equiv) was added to a stirred solution of the crude α-hydroxyimino ester (2.15 g, 9.0 mmol, 1 equiv) in anhydrous tetrahydrofuran (30 mL) at 0 °C. After 30 min of stirring at 0 °C, dimethyl sulfate (0.85 mL, 9.0 mmol, 1.0 equiv) was added at 0 °C, and the resulting solution was allowed to warm to room temperature and stirred at this temperature for 20 h (TLC: 20% ethyl acetate in hexane). The reaction mixture was filtered, ice-cold brine (80 mL) was added, and the resulting mixture was extracted with dichloromethane (3 × 80 mL). The combined organic phases were dried over anhydrous sodium sulfate, the dried solution was filtered, and the filtrate was concentrated in vacuo. The obtained residue was purified by FCC (gradient elution with 8–12% ethyl acetate in hexane) to provide (*Z*)-α-methoxyimino ester *Z*-**3l** as a pale-yellow oil (723 mg, 30%) and (*E*)-α-methoxyimino ester *E*-**3l** as a pale-yellow oil (751 mg, 31%).

α-Methoxyimino ester *Z*-**3l**: TLC (20% ethyl acetate in hexane): R_f = 0.55. ^{1}H NMR (500 MHz, $CDCl_3$) δ: 7.61 (ddd, *J* = 7.7, 6.8, 1.8 Hz, 1H), 7.53 (dddd, *J* = 8.3, 7.1, 5.1, 1.8 Hz, 1H), 7.24 (dd, *J* = 7.6, 1.0 Hz, 1H), 7.13 (ddd, *J* = 10.1, 8.3, 1.1 Hz, 1H), 4.40 (q, *J* = 7.1 Hz, 2H), 4.06 (s, 3H), 1.37 (t, *J* = 7.1 Hz, 3H). ^{13}C{^{1}H} NMR (126 MHz, $CDCl_3$) δ: 186.2, 160.8 (m, 2C), 149.7, 134.2 (d, *J* = 8.7 Hz), 131.1 (d, *J* = 2.3 Hz), 125.3 (d, *J* = 13.3 Hz), 124.1 (d, *J* = 3.7 Hz), 116.4 (d, *J* = 21.7 Hz), 64.7, 62.4, 14.2. FTIR (neat), cm^{-1}: 2987, 2944, 1741, 1670, 1611, 1485, 1452, 1368, 1327, 1274, 1241, 1160, 1143, 1102, 1028, 960, 895, 858, 820, 755. HRMS (APCI): Calcd for $[C_{12}H_{12}FNO_4+H]^+$: 254.0823, found: 254.0824.

α-Methoxyimino ester *E*-**3l**: TLC (20% ethyl acetate in hexane): R_f = 0.55. ^{1}H NMR (500 MHz, $CDCl_3$) δ: 7.96 (td, *J* = 7.6, 1.8 Hz, 1H), 7.61 (dddd, *J* = 8.4, 7.2, 5.1, 1.8 Hz, 1H), 7.29 (ddd, *J* = 8.3, 7.4, 1.1 Hz, 1H), 7.13 (ddd, *J* = 11.1, 8.3, 1.1 Hz, 1H), 4.34 (q, *J* = 7.1 Hz, 2H), 4.01 (s, 3H), 1.29 (t, *J* = 7.1 Hz, 3H). ^{13}C{^{1}H} NMR (126 MHz, $CDCl_3$) δ: 185.8, 162.1 (m, 2C), 150.0, 136.5 (d, *J* = 9.3 Hz), 130.6, 124.9 (d, *J* = 3.4 Hz), 123.4 (d, *J* = 9.4 Hz), 116.9 (d, *J* = 22.4 Hz), 64.4, 62.5, 14.2. FTIR (neat), cm^{-1}: 2985, 2943, 1748, 1714, 1676, 1608, 1481, 1455, 1373, 1323, 1268, 1233, 1209, 1157, 1129, 1101, 1044, 1013, 943, 829, 761, 735. HRMS (APCI): Calcd for $[C_{12}H_{12}FNO_4+H]^+$: 254.0823, found: 254.0821.

Synthesis of ethyl (*S*, *Z*)-3-(2-fluorophenyl)-3-hydroxy-2-(methoxyimino)propanoate *Z*-4l

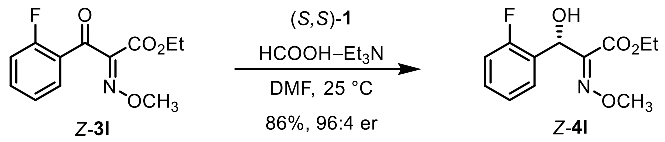

A solution of (*S,S*)-**1** (10.0 mg, 15.8 μmol, 0.02 equiv) in *N,N*-dimethylformamide (0.7 mL) was evacuated and backfilled with argon (4 cycles). Then, α-methoxyimino ester *Z*-**3l** (200 mg, 0.79 mmol, 1 equiv, *Z* isomer) was added as a solution in *N,N*-dimethylformamide (0.3 mL), and the mixture was stirred for 5 min in order to obtain a clear solution. Then, argon was bubbled through the solution for 10 min (outlet needle), and a double-layered balloon filled with argon was attached. A mixture of formic acid and triethylamine (5:2 by volume, 0.4 mL) was added, followed by stirring for 18 h at 25 °C (TLC: 20% ethyl acetate in hexane, UV). Then, ice-cold water (25 mL) was added, and the resulting mixture was extracted with ethyl acetate (3 × 25 mL). The combined organic phases were washed with brine (20 mL), dried over anhydrous sodium sulfate, the dried solution was filtered, and the filtrate was concentrated in vacuo. The obtained residue was purified by FCC (gradient elution with 15–20% ethyl acetate in hexane) to provide alcohol *Z*-**4l** as a pale-yellow oil (173 mg, 86%, *Z* isomer). The enantiomeric purity of *Z*-**4l** was determined by HPLC (96:4 er).

Alcohol *Z*-**4l**: $[\alpha]_D^{23}$ = +38 (*c* 0.8, $CHCl_3$). TLC (20% ethyl acetate in hexane): R_f= 0.5. ^1H NMR (500 MHz, $CDCl_3$) δ: 7.45 (td, *J* = 7.5, 1.8 Hz, 1H), 7.30 (dddd, *J* = 8.3, 7.2, 5.3, 1.8 Hz, 1H), 7.16 (td, *J* = 7.6, 1.2 Hz, 1H), 7.04 (ddd, *J* = 10.5, 8.2, 1.2 Hz, 1H), 5.82 (d, *J* = 2.8 Hz, 1H), 4.18 (q, *J* = 7.1 Hz, 2H), 3.96 (s, 3H), 3.31 (d, *J* = 5.5 Hz, 1H), 1.15 (t, *J* = 7.1 Hz, 3H). $^{13}C\{^1H\}$ NMR (126 MHz, $CDCl_3$) δ: 161.4, 160.5 (d, *J* = 248.3 Hz), 150.2, 130.4 (d, *J* = 8.4 Hz), 128.7 (d, *J* = 3.6 Hz), 125.9 (d, *J* = 12.9 Hz), 124.5 (d, *J* = 3.5 Hz), 115.6 (d, *J* = 21.5 Hz), 67.4 (d, *J* = 3.8 Hz), 63.4, 61.9, 14.0. FTIR (neat), cm^{-1}: 3468, 2983, 2941, 1726, 1616, 1588, 1489, 1457, 1369, 1303, 1270, 1226, 1197, 1176, 1147, 1102, 1028, 915, 882, 859, 838. HRMS (APCI): Calcd for $[C_{12}H_{14}FNO_4+H]^+$: 256.0980, found: 256.0978.

Synthesis of ethyl (*S*)-3-(2-fluorophenyl)-3-hydroxypropanoate 6l

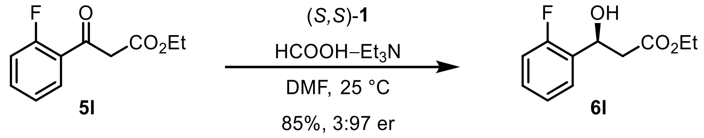

A solution of (*S,S*)-**1** (7.3 mg, 11.5 μmol, 0.02 equiv) in *N,N*-dimethylformamide (0.7 mL) was evacuated and backfilled with argon (4 cycles). Then, ethyl 3-(2-fluorophenyl)-3-oxopropanoate **5l** (120 mg, 0.57 mmol, 1 equiv) was added as a solution in *N,N*-dimethylformamide (0.3 mL), and the mixture was stirred for 5 min in order to obtain a clear solution. Then, argon was bubbled through the solution for 10 min (outlet needle), and a double-layered balloon filled with argon was attached. A mixture of formic acid and triethylamine (5:2 by volume, 0.3 mL) was added, followed by stirring for 18 h at 25 °C (TLC: 20% ethyl acetate in hexane). Then, ice-cold water (20 mL) was added, and the resulting mixture was extracted with ethyl acetate (3 × 20 mL). The combined organic phases were washed with brine (15 mL), dried

over anhydrous sodium sulfate, the dried solution was filtered, and the filtrate was concentrated in vacuo. The obtained residue was purified by FCC (gradient elution with 15–20% ethyl acetate in hexane) to provide alcohol **6l** as a pale-yellow oil (103 mg, 85%). The enantiomeric purity of **6l** was determined by HPLC (3:97 er).

<u>Alcohol **6l**</u>: $[\alpha]_D^{23}$ = –54 (*c* 1.2, $CHCl_3$). TLC (20% ethyl acetate in hexane; ninhydrin): R_f = 0.35. ^1H NMR (500 MHz, $CDCl_3$) δ: 7.54 (td, *J* = 7.6, 1.8 Hz, 1H), 7.33–7.23 (m, 1H), 7.20–7.14 (m, 1H), 7.02 (ddt, *J* = 10.5, 8.2, 1.1 Hz, 1H), 5.41 (dt, *J* = 9.2, 3.7 Hz, 1H), 4.19 (q, *J* = 7.2 Hz, 2H), 3.52–3.45 (m, 1H), 2.80 (ddd, *J* = 16.5, 3.4, 1.2 Hz, 1H), 2.72 (dd, *J* = 16.5, 9.2 Hz, 1H), 1.26 (t, *J* = 7.2 Hz, 3H). $^{13}C\{^1H\}$ NMR (126 MHz, $CDCl_3$) δ: 172.5, 159.6 (d, *J* = 245.7 Hz), 129.6 (dd, *J* = 13.0, 1.7 Hz), 129.3 (d, *J* = 8.2 Hz), 127.4 (d, *J* = 4.2 Hz), 124.5 (d, *J* = 3.4 Hz), 115.4 (d, *J* = 21.4 Hz), 64.8 (d, *J* = 3.0 Hz), 61.1, 42.0, 14.3. FTIR (neat), cm^{-1}: 3461, 2983, 2937, 1715, 1617, 1586, 1488, 1455, 1404, 1372, 1279, 1219, 1179, 1102, 1067, 1030, 942, 872, 809, 756. HRMS (APCI): Calcd for $[C_{11}H_{13}FO_3+H]^+$: 213.0921, found: 213.0922.

<u>**Enantioselective transfer hydrogenation of *ortho*-chlorophenyl substrate 3m**</u>

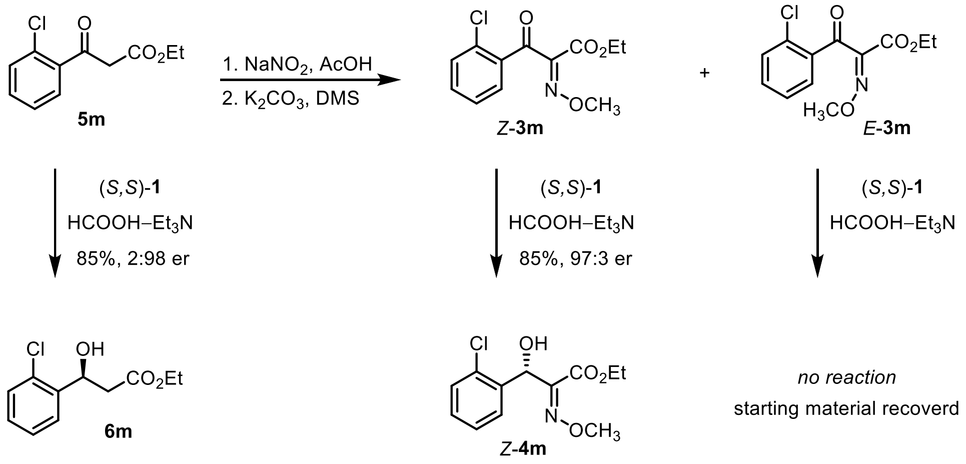

Synthesis of ethyl 3-(2-chlorophenyl)-2-(methoxyimino)-3-oxopropanoate 3m

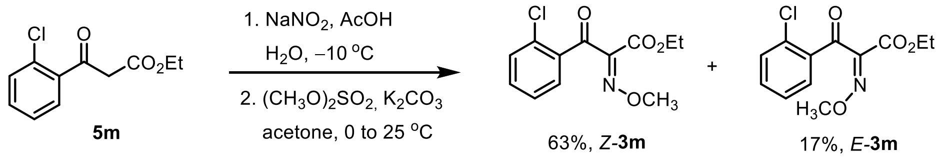

A solution of sodium nitrite (0.80 g, 11,6 mmol, 1.3 equiv) in water (2.0 mL) was added dropwise to a solution of methyl 4-methyl-3-oxopentanoate **5m** (2.03 g, 8.9 mmol, 1 equiv) in acetic acid (3.5 mL) over 15 min at 0 °C. The resulting mixture was stirred for 10 min at 0 °C, then 45 min at 25 °C (TLC: 30% ethyl acetate in hexane; UV). The mixture was poured into brine (40 mL) and extracted with dichloromethane (3 × 30 mL). The organic extracts were combined and washed with a saturated aqueous solution of sodium hydrogen carbonate (100 mL) to reach pH ~

7, and the aqueous phase was extracted again with dichloromethane (3 × 30 mL). All organic extracts were combined, dried over anhydrous sodium sulfate, and filtered. The filtrate was concentrated under reduced pressure to yield the crude α-hydroxyimino ester (2.3 g, not shown), which was used in the next step without further purification.

Potassium carbonate (1.64 g, 11.9 mmol, 1.3 equiv) was added to a stirred solution of crude α-hydroxyimino ester (2.3 g, 9.1 mmol, 1 equiv [assumed]) in anhydrous tetrahydrofuran (20 mL) at 0 °C. After 5 min of stirring at 0 °C, dimethyl sulfate (1.04 mL, 10.96 mmol, 1.2 equiv) was added at 0 °C, and the resulting solution was allowed to warm to room temperature and stirred at this temperature for 18 h (TLC: 30% ethyl acetate in hexane). The reaction mixture was filtered, ice-cold brine (40 mL) was added, and the resulting mixture was extracted with dichloromethane (3 × 30 mL). The combined organic phases were dried over anhydrous sodium sulfate, the dried solution was filtered, and the filtrate was concentrated in vacuo. The obtained residue was purified by FCC (gradient elution with 8–20% ethyl acetate in hexane) to provide separately the *Z* isomer *Z*-**3m** (1.56 g, 63%) and the *E* isomer *E*-**3m** (0.42 g, 17%) of α-methoxyimino ester, each as a yellow oil.

α-Methoxyimino ester *Z*-**3m**: TLC (30% ethyl acetate in hexane): R_f = 0.69. ^{1}H NMR (500 MHz, $CDCl_3$) δ: 7.46–7.40 (m, 3H), 7.37–7.29 (m, 1H), 4.41 (q, *J* = 7.1 Hz, 2H), 4.02 (s, 3H), 1.38 (t, *J* = 7.1 Hz, 3H). $^{13}C\{^1H\}$ NMR (126 MHz, $CDCl_3$) δ: 188.5, 160.8, 149.9, 136.6, 132.2, 132.0, 130.2, 130.0, 126.5, 64.8, 62.5, 14.2. FTIR (neat), cm^{-1}: 2986, 2944, 2361, 1742, 1676, 1591, 1471, 1437, 1389, 1323, 1268, 1238, 1150, 1059, 1029, 961, 895, 858, 755, 738, 676, 645, 475. HRMS (APCI): Calcd for $[C_{12}H_{12}ClNO_4+H]^+$: 270.0528, found: 270.0528.

α-Methoxyimino ester *E*-**3m**: TLC (30% ethyl acetate in hexane): R_f = 0.57. ^{1}H NMR (500 MHz, $CDCl_3$) δ: 7.86 (dd, *J* = 7.8, 1.7 Hz, 1H), 7.52–7.37 (m, 3H), 4.35 (q, *J* = 7.1 Hz, 2H), 4.01 (s, 3H), 1.30 (t, *J* = 7.1 Hz, 3H). $^{13}C\{^1H\}$ NMR (126 MHz, $CDCl_3$) δ: 188.0, 160.4, 149.7, 134.5, 134.3, 133.7, 131.9, 131.4, 127.4, 64.5, 62.6, 14.2. FTIR (neat), cm^{-1}: 2983, 2942, 2361, 1747, 1717, 1672, 1587, 1465, 1437, 1373, 1320, 1265, 1233, 1122, 1062, 1040, 1015, 938, 751.

Synthesis of ethyl (*S*, *Z*)-3-(2-chlorophenyl)-3-hydroxy-2-(methoxyimino)propanoate *Z*-4m

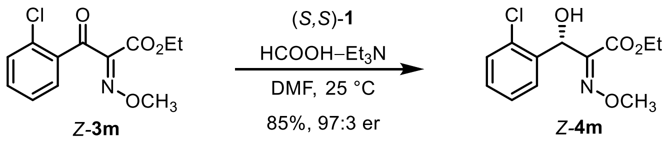

A solution of (*S,S*)-**1** (12.8 mg, 20.1 μmol, 0.02 equiv) in *N*,*N*-dimethylformamide (0.8 mL) was evacuated and backfilled with argon (4 cycles). Then, α-methoxyimino ester *Z*-**3m** (269 mg, 1.00 mmol, 1equiv, *Z* isomer) was added as a solution in anhydrous *N*,*N*-dimethylformamide (0.4 mL), and the mixture was stirred for 5 min in order to obtain a clear solution. Then, argon was bubbled through the solution for 10 min (outlet needle), and a double-layered balloon filled with argon was attached. A mixture of formic acid and triethylamine (5:2 by volume, 0.5 mL) was added, followed by stirring for 16 h at 25 °C (TLC: 20% ethyl acetate in hexane). Then, ice-cold water (25 mL) was added, and the resulting mixture was extracted with ethyl acetate (3 × 25 mL).

The combined organic phases were washed with brine (20 mL), dried over anhydrous sodium sulfate, the dried solution was filtered, and the filtrate was concentrated in vacuo. The obtained residue was purified by FCC (gradient elution with 15–20% ethyl acetate in hexane) to provide alcohol *Z*-**4m** as a pale-yellow oil (211 mg, 85%, *Z* isomer). The enantiomeric purity of *Z*-**4m** was determined by HPLC (97:3 er).

Alcohol *Z*-**4m**: $[\alpha]_D^{23}$ = +64 (*c* 0.9, $CHCl_3$). TLC (20% ethyl acetate in hexane): R_f= 0.5. ^{1}H NMR (500 MHz, $CDCl_3$) δ: 7.51 (dd, *J* = 7.6, 1.8 Hz, 1H), 7.37–7.33 (m, 1H), 7.32–7.23 (m, 3H), 5.97 (d, *J* = 4.8 Hz, 1H), 4.17 (qd, *J* = 7.1, 0.9 Hz, 2H), 3.96 (s, 3H), 3.32 (d, *J* = 5.2 Hz, 1H), 1.14 (t, *J* = 7.1 Hz, 3H). $^{13}C\{^1H\}$ NMR (126 MHz, $CDCl_3$) δ: 161.5, 150.3, 136.1, 133.4, 129.8, 129.7, 128.9, 127.2, 69.7, 63.4, 61.9, 14.0. FTIR (neat), cm^{-1}: 3468, 2982, 2940, 2902, 1725, 1627, 1471, 1441, 1369, 1300, 1267, 1194, 1145, 1062, 1027, 914, 882, 859. HRMS (APCI): Calcd for $[C_{12}H_{14}ClNO_4+H]^+$: 272.0684, found: 272.0669.

Synthesis of ethyl (*S*)-3-(2-chlorophenyl)-3-hydroxypropanoate 6m

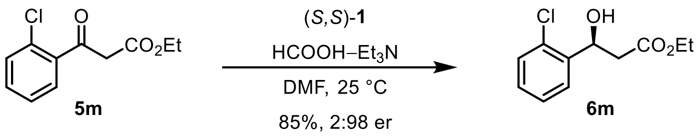

A solution of (*S,S*)-**1** (8.5 mg, 13.3 μmol, 0.02 equiv) in *N,N*-dimethylformamide (0.8 mL) was evacuated and backfilled with argon (4 cycles). Then, ethyl 3-(2-chlorophenyl)-3-oxopropanoate **5m** (150 mg, 0.66 mmol, 1 equiv) was added as a solution in *N,N*-dimethylformamide (0.3 mL), and the mixture was stirred for 5 min in order to obtain a clear solution. Then, argon was bubbled through the solution for 10 min (outlet needle), and a double-layered balloon filled with argon was attached. A mixture of formic acid and triethylamine (5:2 by volume, 0.33 mL) was added, followed by stirring for 40 h at 25 °C (TLC: 20% ethyl acetate in hexane). Then, ice-cold water (25 mL) was added, and the resulting mixture was extracted with ethyl acetate (3 × 25 mL). The combined organic phases were washed with brine (20 mL), dried over anhydrous sodium sulfate, the dried solution was filtered, and the filtrate was concentrated in vacuo. The obtained residue was purified by FCC (gradient elution with 15–20% ethyl acetate in hexane) to provide alcohol **6m** as a pale-yellow oil (128 mg, 85%). The enantiomeric purity of **6m** was determined by HPLC (2:98 er).

Alcohol **6m**: $[\alpha]_D^{23}$ = –79 (*c* 0.9, $CHCl_3$). TLC (20% ethyl acetate in hexane; ninhydrin): R_f = 0.5. ^{1}H NMR (500 MHz, $CDCl_3$) δ: 7.63 (dd, *J* = 7.7, 1.7 Hz, 1H), 7.38–7.28 (m, 2H), 7.22 (td, *J* = 7.6, 1.7 Hz, 1H), 5.49 (dt, *J* = 9.7, 3.1 Hz, 1H), 4.20 (q, *J* = 7.2 Hz, 2H), 3.56 (d, *J* = 3.7 Hz, 1H), 2.85 (dd, *J* = 16.6, 2.8 Hz, 1H), 2.59 (dd, *J* = 16.6, 9.6 Hz, 1H), 1.27 (t, *J* = 7.2 Hz, 3H). $^{13}C\{^1H\}$ NMR (126 MHz, $CDCl_3$) δ: 172.6, 140.1, 131.6, 129.5, 128.9, 127.3, 127.2, 67.2, 61.1, 41.6, 14.3. FTIR (neat), cm^{-1}: 3467, 2982, 2934, 1714, 1573, 1472, 1439, 1398, 1371, 1351, 1292, 1262, 1189, 1158, 1125, 1074, 1029, 945, 891, 869, 754.

Enantioselective transfer hydrogenation of biphenyl substrate 3n

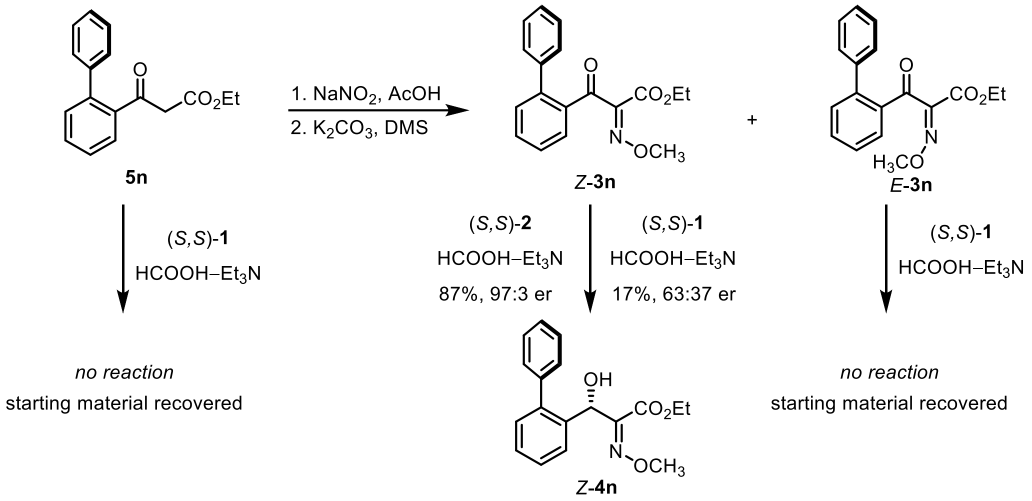

Synthesis of ethyl 3-([1,1'-biphenyl]-2-yl)-2-(methoxyimino)-3-oxopropanoate 3n

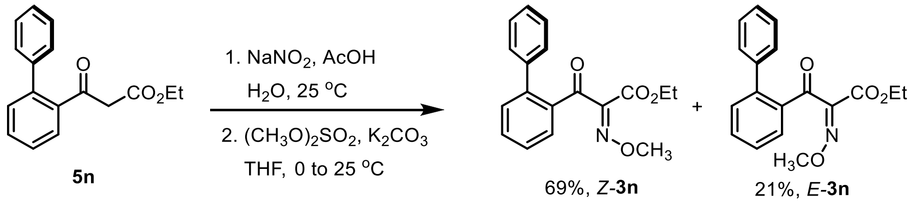

A solution of sodium nitrite (247 mg, 3.58 mmol, 1.2 equiv) in water (1.5 mL) was added dropwise to a solution of ethyl 3-([1,1'-biphenyl]-2-yl)-3-oxopropanoate **5n**[6] (800 mg, 2.99 mmol, 1 equiv) in acetic acid (2.5 mL) over 40 min at 25 °C. The resulting mixture was stirred at this temperature for 2.0 h (TLC: 30% ethyl acetate in hexane; UV, PMA). Then, the mixture was poured into brine (50 mL) and extracted with ether (3 × 30 mL). The organic extracts were combined and washed with a saturated aqueous solution of sodium hydrogen carbonate (80 mL) to reach pH ~ 7, and the aqueous phase was extracted again with ether (3 × 30 mL). All organic extracts were combined, dried over anhydrous sodium sulfate, and filtered. The filtrate was concentrated under reduced pressure to yield the crude α-hydroxyimino ester (975 mg, not shown), which was used in the next step without further purification.

Potassium carbonate (590 mg, 4.27 mmol, 1.3 equiv) was added to a stirred solution of crude α-hydroxyimino ester (975 mg, 3.28 mmol, 1 equiv [assumed]) in anhydrous tetrahydrofuran (25 mL) at 0 °C. After 30 min of stirring at 0 °C, dimethyl sulfate (0.31 mL, 3.28 mmol, 1.0 equiv) was added at 0 °C, and the resulting solution was allowed to warm to room temperature and stirred at this temperature for 2 days (TLC: 20% ethyl acetate in hexane, UV). The reaction mixture was filtered, ice-cold brine (80 mL) was added, and the resulting mixture was extracted with dichloromethane (3 × 60 mL). The combined organic phases were dried over anhydrous sodium sulfate, the dried solution was filtered, and the filtrate was concentrated in vacuo. The obtained residue was purified by FCC (gradient elution with 8–12% ethyl acetate in hexane) to provide (*Z*)-α-methoxyimino ester *Z*-**3n** as a pale-yellow oil (643 mg, 69%) and (*E*)-α-methoxyimino ester *E*-**3n** as a pale-yellow oil (199 mg, 21%).

α-Methoxyimino ester *Z*-**3n**: TLC (20% ethyl acetate in hexane): R_f = 0.55. ^{1}H NMR (500 MHz, $CDCl_3$) δ: 7.60–7.55 (m, 2H), 7.50–7.38 (m, 4H), 7.37–7.29 (m, 3H), 4.29 (q, *J* = 7.1 Hz, 2H), 3.76 (s, 3H), 1.32 (t, *J* = 7.2 Hz, 3H). $^{13}C\{^1H\}$ NMR (126 MHz, $CDCl_3$) δ: 191.7, 160.9, 149.6, 142.2, 140.7, 136.9, 131.4, 130.0, 129.5, 129.2, 128.7, 127.6, 127.2, 64.2, 62.0, 14.2. FTIR (neat), cm^{-1}: 2985, 2941, 1740, 1664, 1595, 1473, 1450, 1367, 1321, 1260, 1233, 1146, 1107, 1027, 959, 893, 858, 778, 744. HRMS (APCI): Calcd for $[C_{18}H_{17}NO_4+H]^+$: 312.1230, found: 312.1230.

α-Methoxyimino ester *E*-**3n**: TLC (20% ethyl acetate in hexane): R_f= 0.5. ^{1}H NMR (500 MHz, $CDCl_3$) δ: 7.89 (dd, *J* = 7.9, 1.4 Hz, 1H), 7.59 (app td, *J* = 7.5, 1.4 Hz, 1H), 7.49 (app td, *J* = 7.6, 1.3 Hz, 1H), 7.42–7.27 (m, 6H), 4.21 (q, *J* = 7.1 Hz, 2H), 3.90 (s, 3H), 1.25 (t, *J* = 7.1 Hz, 3H). $^{13}C\{^1H\}$ NMR (126 MHz, $CDCl_3$) δ: 189.7, 160.5, 148.7, 143.5, 140.1, 134.9, 133.0, 131.4, 130.3, 129.3, 128.2, 128.1, 127.8, 64.2, 62.1, 14.1. FTIR (neat), cm^{-1}: 2984, 2940, 1744, 1713, 1664, 1593, 1473, 1447, 1371, 1316, 1260, 1229, 1129, 1104, 1055, 1039, 1008, 925, 819, 773. HRMS (APCI): Calcd for $[C_{18}H_{17}NO_4+H]^+$: 312.1230, found: 312.1232.

Synthesis of ethyl (*S, Z*)-3-([1,1'-biphenyl]-2-yl)-3-hydroxy-2-(methoxyimino)propanoate *Z*-4n

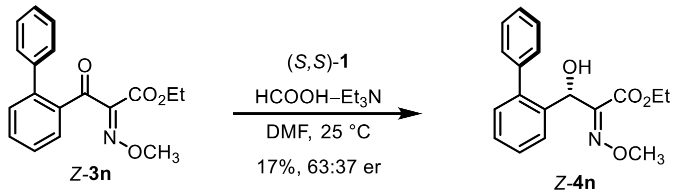

A solution of (*S,S*)-**1** (8.2 mg, 12.9 μmol, 0.02 equiv) in *N,N*-dimethylformamide (0.7 mL) was evacuated and backfilled with argon (4 cycles). Then, α-methoxyimino ester *Z*-**3n** (200 mg, 0.64 mmol, 1 equiv, *Z* isomer) was added as a solution in *N,N*-dimethylformamide (0.3 mL), and the mixture was stirred for 5 min in order to obtain a clear solution. Then, argon was bubbled through the solution for 10 min (outlet needle), and a double-layered balloon filled with argon was attached. A mixture of formic acid and triethylamine (5:2 by volume, 0.35 mL) was added, followed by stirring for 6 days at 25 °C (TLC: 20% ethyl acetate in hexane). Then, ice-cold water (25 mL) was added, and the resulting mixture was extracted with ethyl acetate (3 × 25 mL). The combined organic phases were washed with brine (20 mL), dried over anhydrous sodium sulfate, the dried solution was filtered, and the filtrate was concentrated in vacuo. The obtained residue was purified by FCC (gradient elution with 15–20% ethyl acetate in hexane) to provide alcohol *Z*-**4n** as a pale-yellow oil (34 mg, 17%, *Z* isomer) and recovered starting material *Z*-**3n** (58 mg, 29%). The enantiomeric purity of *Z*-**4n** was determined by HPLC (63:37 er). $[\alpha]_D^{23}$ = +20 (*c* 0.4, $CHCl_3$).

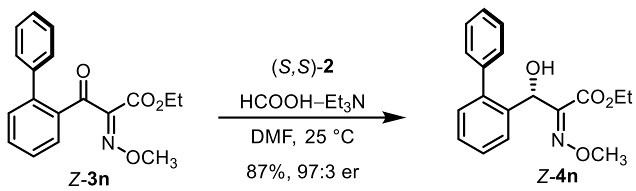

A solution of (*S,S*)-**2** (4.8 mg, 7.7 μmol, 0.02 equiv) in *N,N*-dimethylformamide (0.6 mL) was evacuated and backfilled with argon (4 cycles). Then, α-methoxyimino ester *Z*-**3n** (120 mg,

0.39 mmol, 1 equiv, *Z* isomer) was added as a solution in anhydrous *N*,*N*-dimethylformamide (0.25 mL), and the mixture was stirred for 5 min in order to obtain a clear solution. Then, argon was bubbled through the solution for 10 min (outlet needle), and a double-layered balloon filled with argon was attached. A mixture of formic acid and triethylamine (5:2 by volume, 0.21 mL) was added, followed by stirring for 40 h at 25 °C (TLC: 20% ethyl acetate in hexane). Then, ice-cold water (20 mL) was added, and the resulting mixture was extracted with ethyl acetate (3 × 25 mL). The combined organic phases were washed with brine (20 mL), dried over anhydrous sodium sulfate, the dried solution was filtered, and the filtrate was concentrated in vacuo. The obtained residue was purified by FCC (gradient elution with 15–20% ethyl acetate in hexane) to provide alcohol *Z*-**4n** as a pale-yellow oil (106 mg, 87%, *Z* isomer). The enantiomeric purity of *Z*-**4n** was determined by HPLC (97:3 er). $[\alpha]_D^{23}$ = +69 (*c* 0.8, $CHCl_3$). Single crystals of *Z*-**4n** for X-ray analysis were obtained by allowing the product *Z*-**4n** stand neat at 4 °C.

Alcohol *Z*-**4n**: TLC (20% ethyl acetate in hexane): R_f= 0.45. ^{1}H NMR (500 MHz, $CDCl_3$) δ: 7.50–7.42 (m, 1H), 7.38–7.25 (m, 7H), 7.21–7.14 (m, 1H), 5.54 (s, 1H), 4.07–3.95 (m, 2H), 3.82 (s, 3H), 3.06 (br s, 1H), 0.97 (t, *J* = 7.1 Hz, 3H). $^{13}C\{^1H\}$ NMR (126 MHz, $CDCl_3$) δ: 161.5, 151.3, 142.4, 140.3, 135.8, 130.4, 129.6, 128.6, 128.3, 128.0, 128.0, 127.5, 69.6, 63.2, 61.6, 13.9. FTIR (neat), cm^{-1}: 3493, 2981, 2938, 1728, 1477, 1439, 1368, 1299, 1196, 1145, 1109, 1027, 914, 882, 859, 747. HRMS (APCI): Calcd for $[C_{18}H_{19}NO_4+H]^+$: 314.1387, found: 314.1390.

Enantioselective transfer hydrogenation of furan substrate 4o

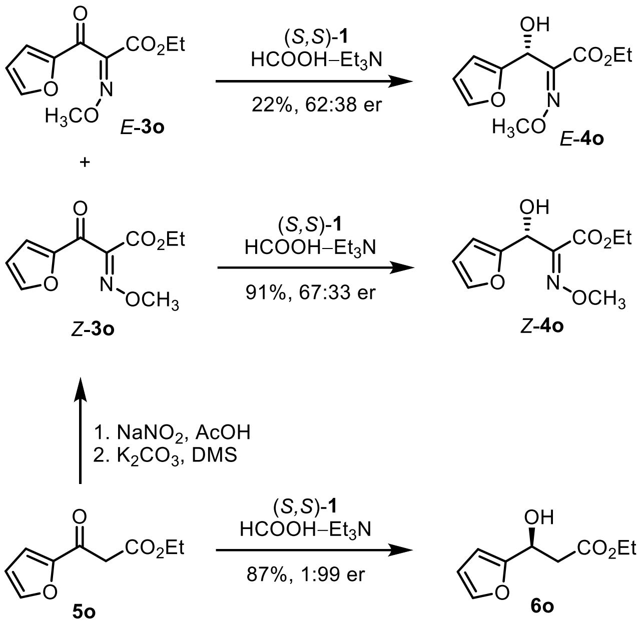

Synthesis of ethyl 3-(furan-2-yl)-2-(methoxyimino)-3-oxopropanoate 3o

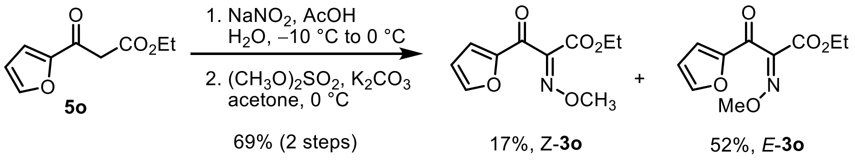

A solution of sodium nitrite (1.14 g, 16.4 mmol, 1.2 equiv) in water (2.0 mL) was added dropwise to a solution of ethyl 3-(furan-2-yl)-3-oxopropanoate **5o** (2.5 g, 13.7 mmol, 1 equiv) in acetic acid (5.0 mL) over 40 min at –10 °C. The resulting mixture was stirred at 0 °C for 2 h (TLC: 30% ethyl acetate in cyclohexane; UV, $KMnO_4$). Then, the mixture was poured into brine (50 mL) and extracted with ethyl acetate (3 × 30 mL). The organic extracts were combined and washed with a saturated aqueous solution of sodium hydrogen carbonate (100 mL) to reach pH ~ 7, and the aqueous phase was extracted again with ethyl acetate (2 × 30 mL). All organic extracts were combined, dried over anhydrous sodium sulfate, and filtered. The filtrate was concentrated under reduced pressure to yield the crude α-hydroxyimino ester (2.89 g, not shown), which was used in the next step without further purification.

Potassium carbonate (2.45 g, 17.8 mmol, 1.3 equiv) was added to a stirred solution of crude α-hydroxyimino ester (2.89 g, 13.6 mmol, 1 equiv) in acetone (30 mL) at 0 °C. After 5 min of stirring at 0 °C, dimethyl sulfate (970 mg, 7.69 mmol, 1 equiv) was added at 0 °C, and the resulting solution was stirred at this temperature for 6 h (TLC: 30% ethyl acetate in cyclohexane; UV, $KMnO_4$). The reaction mixture was filtered, ice-cold brine (50 mL) was added, and the resulting mixture was extracted with ethyl acetate (2 × 50 mL). The combined organic phases were dried over anhydrous sodium sulfate, the dried solution was filtered, and the filtrate was concentrated in vacuo. The obtained residue was purified by FCC (gradient elution with 10–20% ethyl acetate in cyclohexane) to provide separately (*Z*)-α-methoxyimino ester *Z*-**3o** as a white solid (510 mg, 17%) and (*E*)-α-methoxyimino ester *E*-**3o** as a white solid (1.6 g, 52%).

<u>α-Methoxyimino ester *Z*-**3o**</u>: TLC (30% ethyl acetate in cyclohexane): R_f= 0.42. ^{1}H NMR (500 MHz, $CDCl_3$) δ: 7.72 (dd, *J* = 1.7, 0.8 Hz, 1H), 7.60 (dd, *J* = 3.7, 0.8 Hz, 1H), 6.57 (dd, *J* = 3.6, 1.7 Hz, 1H),

4.39 (q, J = 7.1 Hz, 2H), 4.15 (s, 3H), 1.36 (t, J = 7.1 Hz, 3H). $^{13}C\{^{1}H\}$ NMR (126 MHz, $CDCl_3$) δ: 172.7, 160.6, 149.6, 149.3, 148.5, 123.1, 112.6, 64.5, 62.4, 14.2. FTIR (neat), cm^{-1}: 3145, 2986, 2944, 1738, 1643, 1461, 1394, 1336, 1259, 1196, 1144, 1023, 884, 847, 765, 681, 592. HRMS (APCI): Calcd for $[C_{10}H_{11}NO_5+H]^+$: 226.0710, found: 226.0711.

α-Methoxyimino ester *E*-**3o**: TLC (30% ethyl acetate in cyclohexane): R_f = 0.37. ^{1}H NMR (500 MHz, $CDCl_3$) δ: 7.66 (dd, J = 1.7, 0.8 Hz, 1H), 7.23 (dd, J = 3.7, 0.8 Hz, 1H), 6.60 (dd, J = 3.7, 1.7 Hz, 1H), 4.34 (q, J = 7.1 Hz, 2H), 4.05 (s, 3H), 1.30 (t, J = 7.1 Hz, 3H). $^{13}C\{^{1}H\}$ NMR (126 MHz, $CDCl_3$) δ: 176.6, 160.3, 151.1, 148.3, 147.9, 120.4, 113.1, 64.5, 62.6, 14.2. FTIR (neat), cm^{-1}: 3133, 2986, 2944, 1745, 1715, 1663, 1566, 1461, 1395, 1322, 1264, 1135, 1047, 1016, 962, 881, 770. HRMS (APCI): Calcd for $[C_{10}H_{11}NO_5+H]^+$: 226.0710, found: 226.0708.

Synthesis of ethyl (*R*,*Z*)-3-(furan-2-yl)-3-hydroxy-2-(methoxyimino)propanoate *Z*-4o

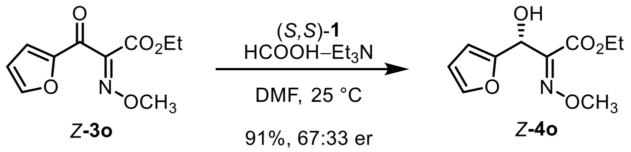

A solution of (*S,S*)-**1** (25.4 mg, 39.9 μmol, 0.02 equiv) in anhydrous *N,N*-dimethylformamide (2.0 mL) was evacuated and backfilled with argon (4 cycles). Then, α-methoxyimino ester *Z*-**3o** (450 mg, 1.99 mmol, 1 equiv, *Z* isomer) was added as a solution in anhydrous *N,N*-dimethylformamide (0.7 mL), and the mixture was stirred for 5 min in order to obtain a clear solution. Then, argon was bubbled through the solution for 10 min (outlet needle), and a double-layered balloon filled with argon was attached. A mixture of formic acid and triethylamine (5:2 by volume, 1.0 mL) was added, followed by stirring for 4 days at 25 °C (TLC: 30% ethyl acetate in cyclohexane; UV, $KMnO_4$). Then, ice-cold water (30 mL) was added, and the resulting mixture was extracted with ethyl acetate (3 × 30 mL). The combined organic phases were washed with brine (10 mL), dried over anhydrous sodium sulfate, the dried solution was filtered, and the filtrate was concentrated in vacuo. The obtained residue was purified by FCC (gradient elution with 10–20% ethyl acetate in cyclohexane) to provide alcohol *Z*-**4o** as a colorless oil (411 mg, 91%, *Z* isomer). The enantiomeric purity of *Z*-**4o** was determined by HPLC (67:33 er).

Alcohol *Z*-**4o**: TLC (30% ethyl acetate in cyclohexane): R_f = 0.35. ^{1}H NMR (500 MHz, $CDCl_3$) δ: 7.40 (dd, J = 1.9, 0.8 Hz, 1H), 6.37 (dt, J = 3.3, 0.8 Hz, 1H), 6.34 (dd, J = 3.3, 1.8 Hz, 1H), 5.54 (d, J = 6.6 Hz, 1H), 4.24 (q, J = 7.1 Hz, 2H), 3.98 (s, 3H), 3.27 (d, J = 6.7 Hz, 1H), 1.22 (t, J = 7.1 Hz, 3H). $^{13}C\{^{1}H\}$ NMR (126 MHz, $CDCl_3$) δ: 161.2, 151.5, 148.4, 143.3, 110.6, 108.6, 67.1, 63.5, 62.0, 14.1. FTIR (neat), cm^{-1}: 3458, 2983, 2942, 1726, 1628, 1464, 1369, 1304, 1197, 1143, 1028, 948, 905, 883, 859, 741, 598, 556. HRMS (APCI): Calcd for $[C_{10}H_{13}NO_5+Na]^+$: 250.0686, found: 250.0684.

Synthesis of ethyl (*R*,*E*)-3-(furan-2-yl)-3-hydroxy-2-(methoxyimino)propanoate *E*-4o

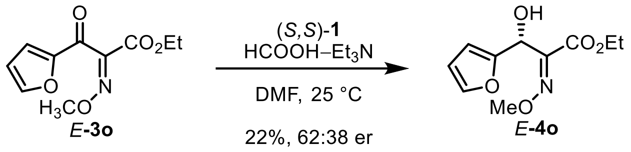

A solution of (*S,S*)-**1** (16.9 mg, 26.6 μmol, 0.02 equiv) in anhydrous *N,N*-dimethylformamide (1.3 mL) was evacuated and backfilled with argon (4 cycles). Then, α-methoxyimino ester *E*-**3o** (300 mg, 1.33 mmol, 1 equiv, *E* isomer) was added as a solution in anhydrous *N,N*-dimethylformamide (0.7 mL), and the mixture was stirred for 5 min in order to obtain a clear solution. Then, argon was bubbled through the solution for 10 min (outlet needle), and a double-layered balloon filled with argon was attached. A mixture of formic acid and triethylamine (5:2 by volume, 0.66 mL) was added, followed by stirring for 8 days at 25 °C (TLC: 20% ethyl acetate in cyclohexane; UV, $KMnO_4$). Then, ice-cold water (30 mL) was added, and the resulting mixture was extracted with ethyl acetate (3 × 20 mL). The combined organic phases were washed with brine (10 mL), dried over anhydrous sodium sulfate, the dried solution was filtered, and the filtrate was concentrated in vacuo. The obtained residue was purified by FCC (gradient elution with 10–40% ethyl acetate in cyclohexane) to provide alcohol *E*-**4o** as a colorless oil (67 mg, 22%, *Z* isomer) and recovered unreacted (*E*)-α-methoxyimino ester *E*-**3o** as an off-white solid (179 mg, 60%). The enantiomeric purity of *E*-**4o** was determined by HPLC (62:38 er).

Alcohol *E*-**4o**: TLC (30% ethyl acetate in cyclohexane): R_f = 0.38. ^{1}H NMR (500 MHz, $CDCl_3$) δ: 7.37 (dd, *J* = 1.8, 0.9 Hz, 1H), 6.33 (dd, *J* = 3.3, 1.8 Hz, 1H), 6.28 (dt, *J* = 3.3, 0.9 Hz, 1H), 6.04 (dd, *J* = 11.8, 0.8 Hz, 1H), 4.41 – 4.31 (m, 2H), 4.23 (d, *J* = 11.7 Hz, 1H), 4.08 (s, 3H), 1.36 (t, *J* = 7.1 Hz, 3H). $^{13}C\{^1H\}$ NMR (126 MHz, $CDCl_3$) δ: 163.2, 152.4, 149.1, 142.7, 110.6, 107.6, 64.1, 63.0, 62.5, 14.2. FTIR (neat), cm^{-1}: 3504, 2984, 2943, 1701, 1601, 1375, 1330, 1255, 1139, 1008, 933, 856, 741, 598. HRMS (APCI): Calcd for $[C_{10}H_{13}NO_5+Na]^+$: 250.0686, found: 250.0683.

Synthesis of ethyl (*S*)-3-(furan-2-yl)-3-hydroxypropanoate 6o

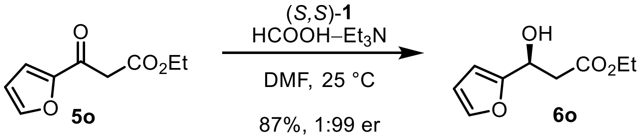

A solution of (*S,S*)-**1** (13.9 mg, 21.9 μmol, 0.02 equiv) in anhydrous *N,N*-dimethylformamide (1.0 mL) was evacuated and backfilled with argon (4 cycles). Then, ethyl 3-(furan-2-yl)-3-oxopropanoate **5o** (200 mg, 1.09 mmol, 1 equiv) was added as a solution in anhydrous *N,N*-dimethylformamide (0.7 mL), and the mixture was stirred for 5 min in order to obtain a clear solution. Then, argon was bubbled through the solution for 10 min (outlet needle), and a double-layered balloon filled with argon was attached. A mixture of formic acid and triethylamine (5:2 by volume, 0.55 mL) was added, followed by stirring for 3 days at 25 °C (TLC: 30% ethyl acetate in cyclohexane; $KMnO_4$). Then, ice-cold water (30 mL) was added, and the resulting mixture was extracted with ethyl acetate (3 × 20 mL). The combined organic phases were washed with brine (10 mL), dried over anhydrous sodium sulfate, the dried solution was filtered, and the filtrate was concentrated in vacuo. The obtained residue was purified by FCC

(gradient elution with 10–30% ethyl acetate in cyclohexane) to provide alcohol **6o** as a colorless oil (174 mg, 87%). The enantiomeric purity of **6o** was determined by HPLC (1:99 er).

<u>Alcohol **6o**</u>: $[\alpha]_D^{25}$ = –23 (*c* 1.22, $CHCl_3$), [lit.[2f] $[\alpha]_D^{25}$ = –17.5 (*c* 1.0, $CHCl_3$), 77% ee]. TLC (30% ethyl acetate in cyclohexane): R_f = 0.4. ^{1}H NMR (500 MHz, $CDCl_3$) δ: 7.37 (dd, *J* = 1.8, 0.8 Hz, 1H), 6.33 (dd, *J* = 3.2, 1.8 Hz, 1H), 6.28 (dt, *J* = 3.2, 0.8 Hz, 1H), 5.16 – 5.12 (m, 1H), 4.19 (q, *J* = 7.2 Hz, 2H), 3.21 (d, *J* = 5.1 Hz, 1H), 2.90 (dd, *J* = 16.5, 8.5 Hz, 1H), 2.83 (dd, *J* = 16.5, 4.1 Hz, 1H), 1.27 (t, *J* = 7.2 Hz, 3H). $^{13}C\{^1H\}$ NMR (126 MHz, $CDCl_3$) δ 172.1, 154.9, 142.4, 110.4, 106.4, 64.4, 61.1, 39.9, 14.3. FTIR (neat), cm^{-1}: 3437, 2983, 2937, 2907, 1718, 1505, 1372, 1282, 1194, 1159, 1065, 1009, 926, 884, 813, 737, 598. HRMS (APCI): Calcd for $[C_9H_{12}O_4+Na]^+$: 207.0628, found: 207.0628.

<u>Enantioselective transfer hydrogenation of amide substrate *Z*-4p</u>

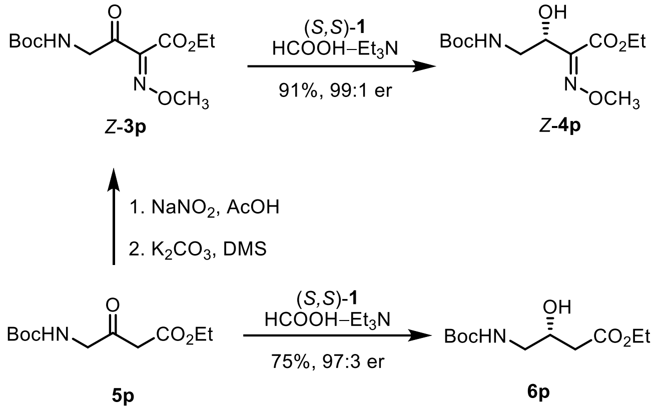

Synthesis of ethyl (*Z*)-4-((*tert*-butoxycarbonyl)amino)-2-(methoxyimino)-3-oxobutanoate 3p

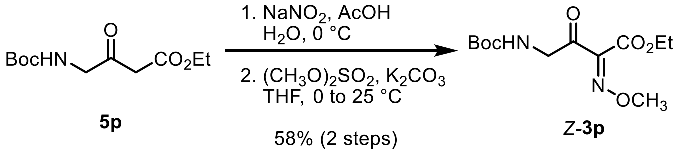

A solution of sodium nitrite (673 mg, 9.75 mmol, 1.2 equiv) in water (2.0 mL) was added dropwise to a solution of ethyl 4-((*tert*-butoxycarbonyl)amino)-3-oxobutanoate[12] **5p** (1.99 g, 8.12 mmol, 1 equiv) in acetic acid (3.5 mL) over 40 min at 0 °C. The resulting mixture was stirred at this temperature for 2 h (TLC: 30% ethyl acetate in cyclohexane; UV, $KMnO_4$). Then, the mixture was poured into brine (50 mL) and extracted with ethyl acetate (3 × 30 mL). The organic extracts were combined and washed with a saturated aqueous solution of sodium hydrogen carbonate (80 mL) to reach pH ~ 7, and the aqueous phase was extracted again with ethyl acetate (3 × 20 mL). All organic extracts were combined, dried over anhydrous sodium sulfate, and filtered. The filtrate was concentrated under reduced pressure to yield the crude α-hydroxyimino ester (2.11 g, not shown), which was used in the next step without further purification.

Potassium carbonate (1.38 g, 10.0 mmol, 1.3 equiv) was added to a stirred solution of crude α-hydroxyimino ester (2.11 g, 7.69 mmol, 1 equiv) in anhydrous tetrahydrofuran (20 mL) at 0 °C. After 5 min of stirring at 0 °C, dimethyl sulfate (970 mg, 7.69 mmol, 1 equiv) was added at 0 °C, and the resulting solution was allowed to warm to room temperature and stirred at this temperature for 23 h (TLC: 20% ethyl acetate in cyclohexane; $KMnO_4$). The reaction mixture was

filtered, ice-cold brine, 50 mL) was added, and the resulting mixture was extracted with ethyl acetate (2 × 30 mL). The combined organic phases were dried over anhydrous sodium sulfate, the dried solution was filtered, and the filtrate was concentrated in vacuo. The obtained residue was purified by FCC (gradient elution with 10–25% ethyl acetate in cyclohexane) to provide α-methoxyimino ester *Z*-**3p** as a white solid (1.35 g, 58%).

α-Methoxyimino ester *Z*-**3p**: TLC (20% ethyl acetate in cyclohexane): R_f = 0.2. ^{1}H NMR (500 MHz, $CDCl_3$) δ: 5.14 (brs, 1H), 4.44 (d, *J* = 5.2 Hz, 2H), 4.35 (q, *J* = 7.2 Hz, 2H), 4.10 (s, 3H), 1.45 (s, 9H), 1.33 (t, *J* = 7.2 Hz, 3H). $^{13}C\{^1H\}$ NMR (126 MHz, $CDCl_3$) δ: 190.2, 160.3, 155.7, 148.1, 80.2, 64.9, 62.5, 46.7, 28.4, 14.2. FTIR (neat), cm^{-1}: 3442, 2988, 2952, 2917, 1741, 1716, 1594, 1510, 1393, 1366, 1283, 1222, 1152, 1010, 932, 909, 881, 709, 504.

Synthesis of ethyl (*S*,*Z*)-4-((*tert*-butoxycarbonyl)amino)-3-hydroxy-2-(methoxyimino)-butanoate *Z*-4p

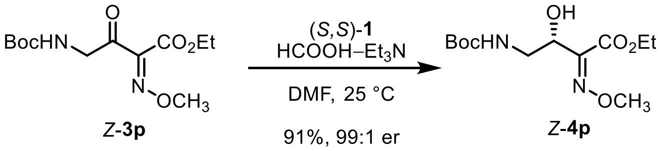

A solution of (*S,S*)-**1** (18.9 mg, 29.8 μmol, 0.02 equiv) in anhydrous *N*,*N*-dimethylformamide (1.5 mL) was evacuated and backfilled with argon (4 cycles). Then, α-methoxyimino ester *Z*-**3p** (430 mg, 1.49 mmol, 1 equiv, *Z* isomer) was added as a solution in anhydrous *N*,*N*-dimethylformamide (0.7 mL), and the mixture was stirred for 5 min in order to obtain a clear solution. Then, argon was bubbled through the solution for 10 min (outlet needle), and a double-layered balloon filled with argon was attached. A mixture of formic acid and triethylamine (5:2 by volume, 0.74 mL) was added, followed by stirring for 15 h at 25 °C (TLC: 30% ethyl acetate in cyclohexane; $KMnO_4$). Then, ice-cold water (30 mL) was added, and the resulting mixture was extracted with ethyl acetate (3 × 20 mL). The combined organic phases were washed with brine (20 mL), dried over anhydrous sodium sulfate, the dried solution was filtered, and the filtrate was concentrated in vacuo. The obtained residue was purified by FCC (gradient elution with 10–25% ethyl acetate in cyclohexane) to provide alcohol *Z*-**4p** as a colorless oil (394 mg, 91%, *Z* isomer). The enantiomeric purity of *Z*-**4p** was determined by HPLC (99:1 er).

Alcohol *Z*-**4p**: TLC (30% ethyl acetate in cyclohexane): R_f = 0.2. ^{1}H NMR (500 MHz, $CDCl_3$) δ: 4.99 (s, 1H), 4.55 – 4.52 (m, 1H), 4.32 (q, *J* = 7.2 Hz, 2H), 3.91 (s, 3H), 3.55 – 3.37 (m, 3H), 1.44 (s, 9H), 1.33 (t, *J* = 7.1 Hz, 3H). $^{13}C\{^1H\}$ NMR (126 MHz, $CDCl_3$) δ: 162.1, 157.0, 150.7, 80.1, 70.4, 63.1, 62.0, 44.5, 28.5, 14.2. FTIR (neat), cm^{-1}: 3399, 2979, 2940, 1691, 1510, 1367, 1250, 1156, 1034, 886, 857, 735, 532. HRMS (APCI): Calcd for $[C_{12}H_{22}N_2O_6+H]^+$: 291.1551, found: 291.1548.

Synthesis of ethyl (3*S*)-2-amino-4-((*tert*-butoxycarbonyl)amino)-3-hydroxybutanoate 4p-amine

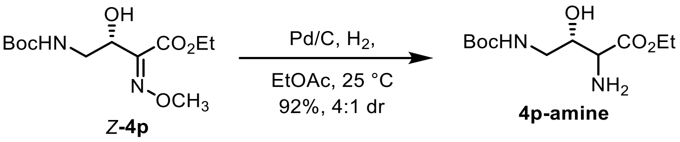

Palladium on charcoal (10% Pd basis; 12.5 mg) was added to a stirred solution of alcohol *Z*-**4p** (300 mg, 1.03 mmol, 1 equiv) in ethyl acetate (5.0 mL) at 25 °C. After stirring the suspension for 10 min, the reaction flask was evacuated, and a double-layered balloon filled with hydrogen gas was attached. The mixture was vigorously stirred for 6 days at 25 °C (TLC: 7% methanol in dichloromethane; ninhydrin). Filtration through a syringe equipped with a PTFE filter and concentration of the filtrate in vacuo provided the crude amino alcohol **4p-amine** as a 4:1 mixture of diastereomers (off-white solid, 250 mg, 92%). By slow evaporation of a solution of crude **4p-amine** (4:1 dr) in a 10:1 mixture of acetonitrile and methanol, respectively, we obtained single crystals of the *S,S*-diastereomer (presumed major diastereomer) for X-ray crystallographic analysis.

4p-amine (crude): TLC (7% methanol in dichloromethane): R_f= 0.3. ^{1}H NMR (500 MHz, $CDCl_3$, 4:1 mixture of two diastereomers; only signals corresponding to the major diastereomer are listed) δ: 5.03 (s, 1H), 4.24 – 4.19 (m, 2H), 3.87 – 3.84 (m, 1H), 3.53 (d, *J* = 5.9 Hz, 1H), 3.32 – 3.23 (m, 2H), 2.54 – 2.32 (m, 3H), 1.43 (s, 9H), 1.29 (t, *J* = 7.1 Hz, 3H). $^{13}C\{^1H\}$ NMR (126 MHz, $CDCl_3$) δ: 173.8, 156.9, 79.8, 72.4, 61.6, 57.0, 43.0, 28.5, 14.3.

Synthesis of ethyl (*R*)-4-((*tert*-butoxycarbonyl)amino)-3-hydroxybutanoate 6p

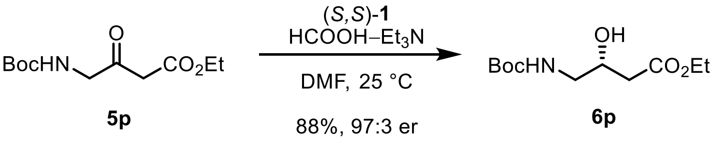

A solution of (*S,S*)-**1** (5.18 mg, 8.15 μmol, 0.02 equiv) in anhydrous *N,N*-dimethylformamide (0.7 mL) was evacuated and backfilled with argon (4 cycles). Then, ethyl 4-((*tert*-butoxycarbonyl)amino)-3-oxobutanoate **5p** (100 mg, 0.41 mmol, 1 equiv) was added as a solution in anhydrous *N,N*-dimethylformamide (0.5 mL), and the mixture was stirred for 5 min in order to obtain a clear solution. Then, argon was bubbled through the solution for 10 min (outlet needle), and a double-layered balloon filled with argon was attached. A mixture of formic acid and triethylamine (5:2 by volume, 0.20 mL) was added, followed by stirring for 16 h at 25 °C (TLC: 30% ethyl acetate in cyclohexane; $KMnO_4$). Then, ice-cold water (20 mL) was added, and the resulting mixture was extracted with ethyl acetate (3 × 15 mL). The combined organic phases were washed with brine (10 mL), dried over anhydrous sodium sulfate, the dried solution was filtered, and the filtrate was concentrated in vacuo. The obtained residue was purified by FCC (elution with 30% ethyl acetate in cyclohexane) to provide alcohol **6p** as a colorless oil (89 mg, 88%). The enantiomeric purity of **6p** was determined by HPLC (97:3 er).

Alcohol **6p**: $[\alpha]_D^{25}$ = +5.8 (*c* 1.0, $CHCl_3$), [lit.[2g] $[\alpha]_D^{20}$ = +6.6 (*c* 1.06, $CHCl_3$)]. TLC (30% ethyl acetate in cyclohexane): R_f = 0.14. ^1H NMR (500 MHz, $CDCl_3$) δ: 4.99 (s, 1H), 4.17 (q, *J* = 7.1 Hz, 2H), 4.12 – 4.07 (m, 1H), 3.48 (d, *J* = 3.9 Hz, 1H), 3.35 – 3.30 (m, 1H), 3.12 (ddd, *J* = 14.0, 6.9, 5.6 Hz, 1H), 2.52 – 2.43 (m, 2H), 1.44 (s, 9H), 1.27 (t, *J* = 7.1 Hz, 3H). $^{13}C\{^1H\}$ NMR (126 MHz, $CDCl_3$) δ: 172.7, 156.7, 79.8, 67.9, 61.0, 45.6, 38.8, 28.5, 14.3. FTIR (neat), cm^{-1}: 3376, 2978, 2933, 1689, 1518, 1367, 1273, 1249, 1162, 1096, 1028.

Enantioselective transfer hydrogenation of pyrazole substrate *Z*-4q

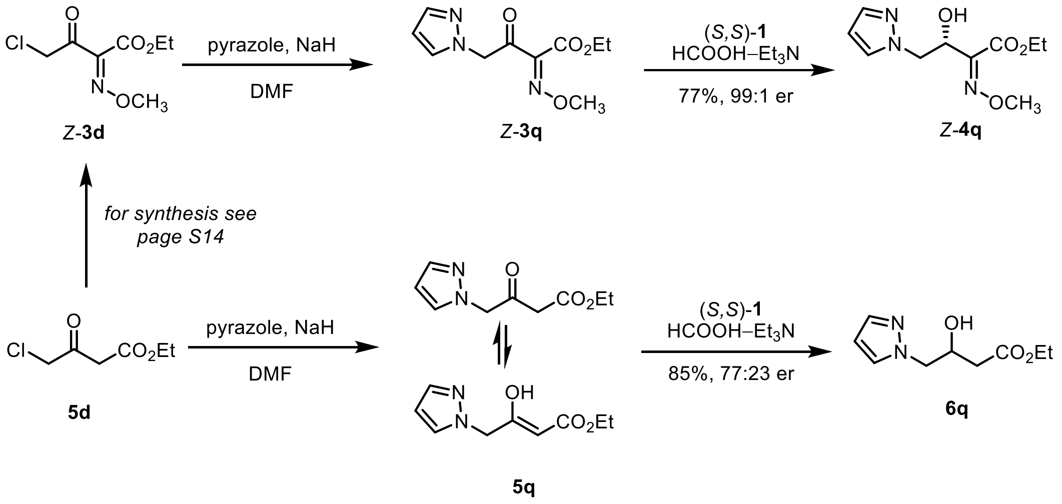

Synthesis of ethyl (*Z*)-2-(methoxyimino)-3-oxo-4-(1*H*-pyrazol-1-yl)butanoate *Z*-3q

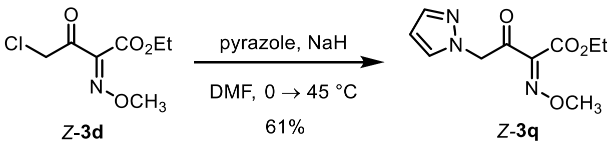

To a solution of the ethyl (*Z*)-4-chloro-2-(methoxyimino)-3-oxobutanoate *Z*-**3d** (1.3 g, 6.26 mmol, 1 equiv) in anhydrous *N*,*N*-dimethylformamide (10 mL) was added sodium hydride (60% dispersion in mineral oil, 375 mg, 9.39 mmol, 1.5 equiv) at 0 °C, and the resulting mixture was stirred for 30 min. Then, pyrazole (639 mg, 9.39 mmol, 1.5 equiv) was added as a solution in anhydrous *N*,*N*-dimethylformamide (4 mL) at 0 °C and the resulting mixture was heated to 45 °C and stirred for 7 h (TLC: 30% ethyl acetate in cyclohexane; UV, $KMnO_4$). Then, the reaction mixture cooled to room temperature and a saturated aqueous solution of ammonium chloride (50 mL) was added carefully. The resulting mixture was extracted with ethyl acetate (3 × 50 mL), the combined organic phases were dried over anhydrous sodium sulfate, the dried solution was filtered, and the filtrate was concentrated in vacuo. The obtained residue was purified by FCC (gradient elution with 10–30% ethyl acetate in cyclohexane) to provide ethyl (*Z*)-2-(methoxyimino)-3-oxo-4-(1*H*-pyrazol-1-yl)butanoate *Z*-**3q** as a pale-yellow oil (915 mg, 61%).

α-Methoxyimino ester *Z*-**3q**: TLC (30% ethyl acetate in cyclohexane): R_f = 0.2. ^1H NMR (500 MHz, $CDCl_3$) δ: 7.56 (d, *J* = 1.4 Hz, 1H), 7.44 (d, *J* = 2.3 Hz, 1H), 6.34 (t, *J* = 2.0 Hz, 1H), 5.41 (s, 2H), 4.34 (q, *J* = 7.1 Hz, 2H), 4.16 (s, 3H), 1.31 (t, *J* = 7.1 Hz, 3H). $^{13}C\{^1H\}$ NMR (126 MHz, $CDCl_3$) δ: 187.5,

159.9, 148.3, 140.3, 131.1, 106.7, 65.1, 62.6, 56.4, 14.1. FTIR (neat), cm^{-1}: 2986, 2945, 1738, 1711, 1597, 1448, 1372, 1302, 1214, 1033, 753, 631. HRMS (APCI): Calcd for $[C_{10}H_{13}N_3O_4+H]^+$: 240.0979, found: 240.0979.

Synthesis of ethyl (*S*,*Z*)-3-hydroxy-2-(methoxyimino)-4-(1*H*-pyrazol-1-yl)butanoate *Z*-4q

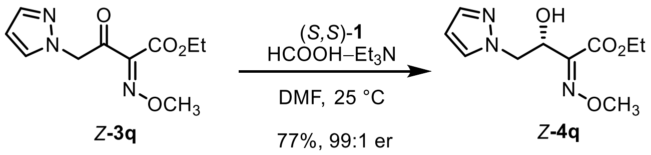

A solution of (*S,S*)-**1** (21.3 mg, 33.4 μmol, 0.02 equiv) in anhydrous *N,N*-dimethylformamide (1.5 mL) was evacuated and backfilled with argon (4 cycles). Then, α-methoxyimino ester *Z*-**3q** (400 mg, 1.67 mmol, 1 equiv, *Z* isomer) was added as a solution in anhydrous *N,N*-dimethylformamide (0.7 mL), and the mixture was stirred for 5 min in order to obtain a clear solution. Then, argon was bubbled through the solution for 10 min (outlet needle), and a double-layered balloon filled with argon was attached. A mixture of formic acid and triethylamine (5:2 by volume, 0.83 mL) was added, followed by stirring for 17 h at 25 °C (TLC: 30% ethyl acetate in cyclohexane; UV, $KMnO_4$). Then, ice-cold water (30 mL) was added, and the resulting mixture was extracted with ethyl acetate (3 × 20 mL). The combined organic phases were washed with brine (10 mL), dried over anhydrous sodium sulfate, the dried solution was filtered, and the filtrate was concentrated in vacuo. The obtained residue was purified by FCC (gradient elution with 10–30% ethyl acetate in cyclohexane) to provide alcohol *Z*-**4q** as a pale-yellow solid (311 mg, 77%, *Z* isomer). The enantiomeric purity of *Z*-**4q** was determined by HPLC (99:1 er).

Alcohol *Z*-**4q**: TLC (30% ethyl acetate in cyclohexane): R_f= 0.2. ^{1}H NMR (500 MHz, $CDCl_3$) δ: 7.53 (d, *J* = 1.8 Hz, 1H), 7.45 (d, *J* = 2.3 Hz, 1H), 6.26 (t, *J* = 2.1 Hz, 1H), 4.85 (m, 1H), 4.48 (dd, *J* = 14.0, 3.4 Hz, 1H), 4.40 (dd, *J* = 14.0, 7.4 Hz, 1H), 4.34 – 4.30 (m, 3H), 3.88 (s, 3H), 1.33 (t, *J* = 7.1 Hz, 3H). $^{13}C\{^1H\}$ NMR (126 MHz, $CDCl_3$) δ: 161.9, 149.9, 140.2, 131.0, 105.7, 70.0, 63.1, 62.1, 54.6, 14.3. FTIR (neat), cm^{-1}: 3184, 2983, 2941, 1724, 1445, 1398, 1304, 1199, 1033, 885, 756, 619. HRMS (APCI): Calcd for $[C_{10}H_{15}N_3O_4+H]^+$: 242.1135, found: 242.1133.

Synthesis of ethyl 3-oxo-4-(1*H*-pyrazol-1-yl)butanoate 5q

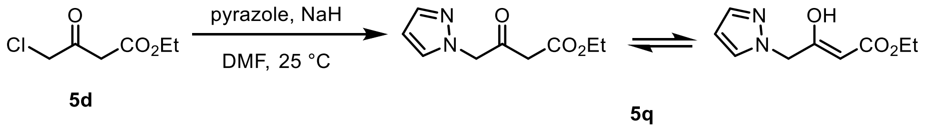

To a solution of the ethyl 4-chloro-3-oxobutanoate **5d** (1.0 g, 6.07 mmol, 1 equiv) in anhydrous *N,N*-dimethylformamide (10 mL) was added sodium hydride (60% dispersion in mineral oil, 380 mg, 9.11 mmol, 1.5 equiv) at 0 °C under argon atmosphere, and the resulting mixture was stirred for 30 min. Then, pyrazole (620 mg, 9.11 mmol, 1.5 equiv) was added as a solution in anhydrous *N,N*-dimethylformamide (4 mL), and the resulting mixture was stirred at 25 °C for 47 h (TLC: 40% ethyl acetate in cyclohexane; $KMnO_4$). Then, a saturated aqueous solution of ammonium chloride (50 mL) was added carefully, and the resulting mixture was extracted with

ethyl acetate (3 × 30 mL). The combined organic phases were dried over anhydrous sodium sulfate, the dried solution was filtered, and the filtrate was concentrated in vacuo. The obtained residue was purified by FCC (gradient elution with 20–30% ethyl acetate in cyclohexane) to provide ethyl 3-oxo-4-(1*H*-pyrazol-1-yl)butanoate **5q** as a brown solid (714 mg, 60%).

Ethyl 3-oxo-4-(1*H*-pyrazol-1-yl)butanoate **5q**: TLC (40% ethyl acetate in cyclohexane): R_f= 0.2. ^{1}H NMR (500 MHz, $CDCl_3$, 5:1 mixture of keto and enol form) δ: 12.06 (s, 1H, enol), 7.57 (d, *J* = 1.9 Hz, 1H, keto), 7.55 (d, *J* = 1.9 Hz, 1H, enol), 7.46 (d, *J* = 2.3 Hz, 1H, enol), 7.43 (d, *J* = 2.3 Hz, 1H, keto), 6.33 (t, *J* = 2.1 Hz, 1H, keto), 6.30 (t, *J* = 2.1 Hz, 1H, enol), 5.03 (s, 2H, keto), 4.83 (d, *J* = 1.0 Hz, 2H, enol), 4.74 (t, *J* = 1.0 Hz, 1H, enol), 4.16 (q, *J* = 7.2 Hz, 2H, enol), 4.15 (q, *J* = 7.2 Hz, 2H, keto), 3.36 (s, 2H, keto), 1.25 (t, *J* = 7.1 Hz, 3H, keto), 1.25 (t, *J* = 7.1 Hz, 3H, enol). ^{13}C{^{1}H} NMR (126 MHz, $CDCl_3$, 5:1 mixture of keto and enol form) δ 197.7 (keto), 172.4 (enol), 171.7 (enol), 166.7 (keto), 140.8 (keto), 140.4 (enol), 131.0 (keto), 130.5 (enol), 106.9 (keto), 106.5 (enol), 90.2 (enol), 61.8 (keto), 60.6 (enol), 60.4(keto), 53.9 (enol), 46.1 (keto), 14.2 (enol), 14.1 (keto). FTIR (neat), cm^{-1}: 3122, 2983, 2936, 1720, 1518, 1395, 1368, 1317, 1260, 1185, 1090, 1026, 966, 753, 618, 578. HRMS (APCI): Calcd for $[C_9H_{12}N_2O_3+H]^+$: 197.0921, found: 197.0923.

Synthesis of ethyl 3-hydroxy-4-(1*H*-pyrazol-1-yl)butanoate 6q

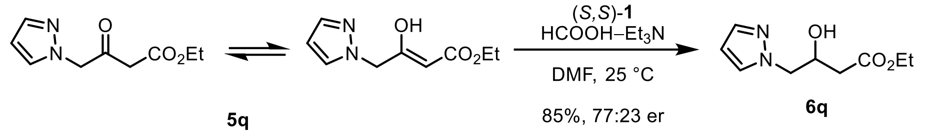

5q **6q**

A solution of (*S,S*)-**1** (13.0 mg, 20.4 μmol, 0.02 equiv) in anhydrous *N,N*-dimethylformamide (1.5 mL) was evacuated and backfilled with argon (4 cycles). Then, ethyl 3-oxo-4-(1*H*-pyrazol-1-yl)butanoate **5q** (200 mg, 1.02 mmol, 1 equiv) was added as a solution in anhydrous *N,N*-dimethylformamide (0.7 mL), and the mixture was stirred for 5 min in order to obtain a clear solution. Then, argon was bubbled through the solution for 10 min (outlet needle), and a double-layered balloon filled with argon was attached. A mixture of formic acid and triethylamine (5:2 by volume, 0.50 mL) was added, followed by stirring for 16 h at 25 °C (TLC: 40% ethyl acetate in cyclohexane; $KMnO_4$). Then, ice-cold water (20 mL) was added, and the resulting mixture was extracted with ethyl acetate (3 × 20 mL). The combined organic phases were washed with brine (20 mL), dried over anhydrous sodium sulfate, the dried solution was filtered, and the filtrate was concentrated in vacuo. The obtained residue was purified by FCC (gradient elution with 20–50% ethyl acetate in cyclohexane) to provide alcohol **6q** as a pale-yellow oil (171 mg, 85%). The enantiomeric purity of **6q** was determined by HPLC (77:23 er).

Alcohol **6q**: $[\alpha]_D^{25}$ = –0.8 (*c* 1.15, $CHCl_3$). TLC (40% ethyl acetate in cyclohexane): R_f= 0.15. ^{1}H NMR (500 MHz, $CDCl_3$) δ: 7.51 (d, *J* = 1.2 Hz, 1H), 7.44 (d, *J* = 2.2 Hz, 1H), 6.26 (t, *J* = 2.1 Hz, 1H), 4.39 (m, 1H), 4.28 (dd, *J* = 13.9, 3.7 Hz, 1H), 4.18 (dd, *J* = 13.9, 6.6 Hz, 1H), 4.15 (q, *J* = 7.1 Hz, 2H), 4.03 (d, *J* = 4.2 Hz, 1H), 2.45 – 2.43 (m, 2H), 1.25 (t, *J* = 7.1 Hz, 3H). ^{13}C{^{1}H} NMR (126 MHz, $CDCl_3$) δ 171.8, 139.9, 130.6, 105.7, 67.7, 61.0, 56.3, 38.7, 14.2. FTIR (neat), cm^{-1}: 3198, 3123, 2980, 2914, 1725, 1517, 1401, 1355, 1273, 1224, 1190, 1153, 1092, 1064, 1020, 977, 884, 758, 618, 540. HRMS (APCI): Calcd for $[C_9H_{14}N_2O_3+H]^+$: 199.1077, found: 199.1079.

Enantioselective transfer hydrogenation of thiophenol substrate *Z*-4r

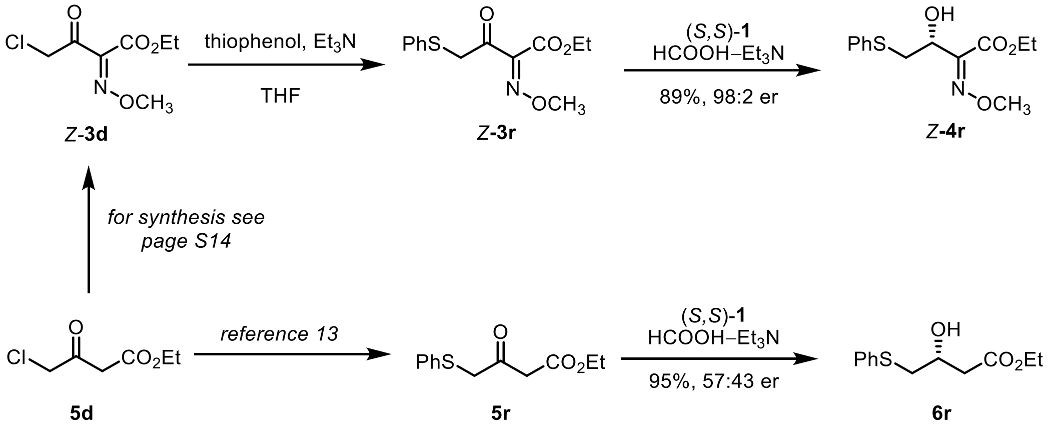

Synthesis of ethyl (*Z*)-2-(methoxyimino)-3-oxo-4-(phenylthio)butanoate *Z*-3r

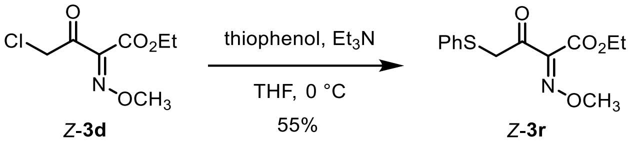

To a solution of the ethyl (*Z*)-4-chloro-2-(methoxyimino)-3-oxobutanoate *Z*-**3d** (1 g, 4.82 mmol, 1 equiv) and triethylamine (731 mg, 7.22 mmol, 1.5 equiv) in anhydrous tetrahydrofuran (5.0 mL) was added thiophenol (583 mg, 5.29 mmol, 1.1 equiv) at 0 °C, and the resulting mixture was stirred for 2 h at this temperature (TLC: 20% ethyl acetate in cyclohexane; UV, $KMnO_4$). Then, water was added and the resulting mixture was extracted with ethyl acetate (3 × 30 mL), the combined organic phases were washed with brine (20 mL), dried over anhydrous sodium sulfate, the dried solution was filtered, and the filtrate was concentrated in vacuo. The obtained residue was purified by FCC (gradient elution with 10–20% ethyl acetate in cyclohexane) to provide ethyl (*Z*)-2-(methoxyimino)-3-oxo-4-(phenylthio)butanoate *Z*-**3r** as a pale-yellow oil (800 mg, 55%).

<u>α-Methoxyimino ester *Z*-**3r**:</u> TLC (20% ethyl acetate in cyclohexane): R_f= 0.55. ^{1}H NMR (500 MHz, $CDCl_3$) δ: 7.44 – 7.41 (m, 2H), 7.31 – 7.28 (m, 2H), 7.26 – 7.23 (m, 1H), 4.34 (q, *J* = 7.1 Hz, 2H), 4.04 (s, 3H), 4.03 (s, 2H), 1.33 (t, *J* = 7.2 Hz, 3H). $^{13}C\{^1H\}$ NMR (126 MHz, $CDCl_3$) δ 188.8, 160.7, 148.8, 134.5, 131.5, 129.2, 127.6, 64.7, 62.4, 39.9, 14.2. FTIR (neat), cm^{-1}: 2986, 2942, 2905, 1738, 1688, 1597, 1439, 1368, 1323, 1243, 1202, 1036, 920, 740, 690. HRMS (APCI): Calcd for $[C_{13}H_{15}NO_4S+H]^+$: 282.0795, found: 282.0797.

Synthesis of ethyl (*R*)-4-chloro-3-hydroxy-2-(methoxyimino)butanoate *Z*-4r

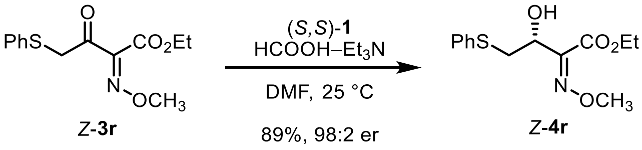

A solution of (*S,S*)-**1** (10.2 mg, 15.9 μmol, 0.02 equiv) in anhydrous *N*,*N*-dimethylformamide (1.5 mL) was evacuated and backfilled with argon (4 cycles). Then, ethyl (*Z*)-2-(methoxyimino)-3-oxo-4-(phenylthio)butanoate *Z*-**3r** (225 mg, 0.80 mmol, 1 equiv, *Z* isomer) was added as a solution in anhydrous *N*,*N*-dimethylformamide (0.8 mL), and the mixture was stirred for 5 min in order to obtain a clear solution. Then, argon was bubbled through the solution for 10 min (outlet needle), and a double-layered balloon filled with argon was attached. A mixture of formic acid and triethylamine (5:2 by volume, 0.4 mL) was added, followed by stirring for 18 h at 25 °C (TLC: 30% ethyl acetate in cyclohexane; UV, $KMnO_4$). Then, ice-cold water (25 mL) was added, and the resulting mixture was extracted with ethyl acetate (3 × 25 mL). The combined organic phases were washed with brine (10 mL), dried over anhydrous sodium sulfate, the dried solution was filtered, and the filtrate was concentrated in vacuo. The obtained residue was purified by FCC (elution with 20% ethyl acetate in cyclohexane) to provide alcohol *Z*-**4r** as a colorless oil (202 mg, 89%, *Z* isomer). The enantiomeric purity of *Z*-**4r** was determined by HPLC (98:2 er).

<u>Alcohol *Z*-**4r**:</u> TLC (30% ethyl acetate in cyclohexane): R_f = 0.4. ^{1}H NMR (500 MHz, $CDCl_3$) δ 7.43 – 7.40 (m, 2H), 7.32 – 7.28 (m, 2H), 7.25 – 7.21 (m, 1H), 4.51 (dt, *J* = 8.4, 4.6 Hz, 1H), 4.32 (qd, *J* = 7.2, 1.0 Hz, 2H), 3.91 (s, 3H), 3.35 (dd, *J* = 14.1, 4.3 Hz, 1H), 3.23 (dd, *J* = 14.1, 8.3 Hz, 1H), 2.97 (d, *J* = 4.8 Hz, 1H), 1.32 (t, *J* = 7.1 Hz, 3H). ^{13}C{^{1}H} NMR (126 MHz, $CDCl_3$) δ: 162.0, 150.7, 134.7, 130.7, 129.3, 127.1, 68.9, 63.1, 62.0, 39.8, 14.2. FTIR (neat), cm^{-1}: 3477, 2981, 1725, 1439, 1301, 1199, 1033, 741, 692. HRMS (APCI): Calcd for $[C_{13}H_{17}NO_4S+Na]^+$: 306.0770, found: 306.0770.

Synthesis of ethyl (*R*)-3-hydroxy-4-(phenylthio)butanoate 6r

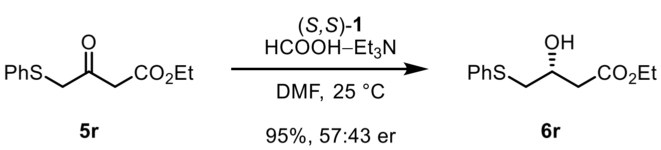

A solution of (*S,S*)-**1** (10.7 mg, 16.8 μmol, 0.02 equiv) in anhydrous *N*,*N*-dimethylformamide (1.5 mL) was evacuated and backfilled with argon (4 cycles). Then, ethyl 3-oxo-4-(phenylthio)butanoate[13] **5r** (200 mg, 0.84 mmol, 1 equiv) was added as a solution in anhydrous *N*,*N*-dimethylformamide (0.7 mL), and the mixture was stirred for 5 min in order to obtain a clear solution. Then, argon was bubbled through the solution for 10 min (outlet needle), and a double-layered balloon filled with argon was attached. A mixture of formic acid and triethylamine (5:2 by volume, 0.42 mL) was added, followed by stirring for 20 h at 25 °C (TLC: 30% ethyl acetate in cyclohexane; $KMnO_4$). Then, ice-cold water (20 mL) was added, and the resulting mixture was extracted with ethyl acetate (3 × 15 mL). The combined organic phases were washed with brine (10 mL), dried over anhydrous sodium sulfate, the dried solution was filtered, and the filtrate was concentrated in vacuo. The obtained residue was purified by FCC (elution with 20% ethyl acetate in cyclohexane) to provide alcohol **6r** as a colorless oil (191 mg, 95%). The enantiomeric purity of **6r** was determined by HPLC (57:43 er).

<u>Alcohol **6r**</u>: $[\alpha]_D^{25}$ = +1.8 (*c* 0.79, $CHCl_3$), [lit.[2h] $[\alpha]_D$ = +5.8 (*c* 10.0, $CHCl_3$, 58% ee)]. TLC (30% ethyl acetate in cyclohexane): R_f = 0.6. ^{1}H NMR (500 MHz, $CDCl_3$) δ: 7.41 – 7.37 (m, 2H), 7.31 – 7.27 (m, 2H), 7.22 – 7.19 (m, 1H), 4.18 – 4.10 (m, 3H), 3.19 (d, *J* = 4.0 Hz, 1H), 3.10 (dd, *J* = 13.8, 5.7 Hz, 1H), 3.05 (dd, *J* = 13.8, 6.9 Hz, 1H), 2.66 (dd, *J* = 16.4, 4.2 Hz, 1H), 2.56 (dd, *J* = 16.4, 8.0 Hz, 1H), 1.26 (t,

J = 7.1 Hz, 3H). $^{13}C\{^1H\}$ NMR (126 MHz, $CDCl_3$) δ: 172.2, 135.4, 129.9, 129.2, 126.7, 66.8, 61.0, 40.4, 40.1, 14.3. FTIR (neat), cm^{-1}:3450, 2981, 2927, 1722, 1583, 1480, 1439, 1372, 1299, 1185, 1145, 1023, 738, 690. HRMS (APCI): Calcd for $[C_{12}H_{16}O_3S+Na]^+$: 263.0712, found: 263.0711.

Enantioselective transfer hydrogenation of ethyl substrate *E*-8

Synthesis of 4-(methoxyimino)hexan-3-one *E*-8

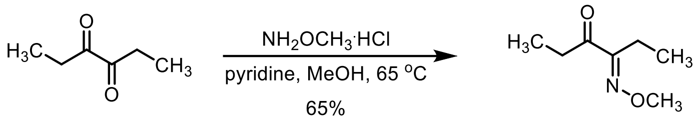

To a solution of hexane-3,4-dione **29** (1.46 mL, 12.0 mmol, 1 equiv) in anhydrous methanol (18 mL) was added methoxyamine hydrochloride (1.00 g, 12.0 mmol, 1.0 equiv) and pyridine (0.97 mL, 12.0 mmol, 1.0 mL). The reaction mixture was refluxed for 5 h. Then, the solvent was removed in vacuo and the obtained residue was purified by FCC (gradient elution with 10–15% dichloromethane in hexane) to provide α-methoxyimino ester *E*-**8** as a pale-yellow oil (1.12 g, 65%).

Methoxyimino ketone *E*-**8**: TLC (20% dichloromethane in hexane): R_f = 0.5. ^1H NMR (500 MHz, $CDCl_3$) δ: 4.02 (s, 3H), 2.79 (q, *J* = 7.4 Hz, 2H), 2.47 (q, *J* = 7.6 Hz, 2H), 1.09 (t, *J* = 7.4 Hz, 3H), 0.98 (t, *J* = 7.6 Hz, 3H). $^{13}C\{^1H\}$ NMR (126 MHz, $CDCl_3$) δ: 199.5, 159.6, 63.1, 30.7, 16.7, 10.7, 8.3. FTIR (neat), cm^{-1}: 2977, 2941, 2881, 1693, 1602, 1460, 1411, 1373, 1232, 1102, 1077, 1038, 984, 942, 882, 821, 754. HRMS (APCI): Calcd for $[C_7H_{13}NO_2+H]^+$: 144.1019, found: 144.1022.

Synthesis of (*S*)-4-hydroxyhexan-3-one *O*-methyl oxime 9

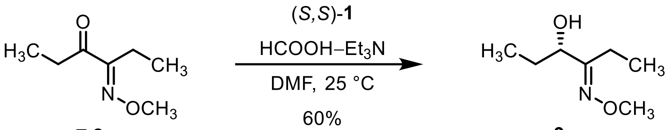

A solution of (*S,S*)-**1** (17.8 mg, 27.9 μmol, 0.02 equiv) in *N,N*-dimethylformamide (1.2 mL) was evacuated and backfilled with argon (4 cycles). Then, α-methoxyimino ester *E*-**8** (200 mg, 1.40 mmol, 1 equiv, *Z* isomer) was added as a solution in *N,N*-dimethylformamide (0.4 mL), and the mixture was stirred for 5 min in order to obtain a clear solution. Then, argon was bubbled through the solution for 10 min (outlet needle), and a double-layered balloon filled with argon was attached. A mixture of formic acid and triethylamine (5:2 by volume, 0.7 mL) was added, followed by stirring for 2 days at 25 °C (TLC: 20% ethyl acetate in hexane). Then, ice-cold water (25 mL) was added, and the resulting mixture was extracted with diethyl ether (3 × 25 mL). The combined organic phases were washed with brine (20 mL), dried over anhydrous sodium sulfate, the dried solution was filtered, and the filtrate was concentrated in vacuo. The obtained residue was purified by FCC (gradient elution with 15–20% ethyl acetate in hexane) to provide alcohol **9** as a colorless oil (122 mg, 60%, 8:1 mixture of *E*:*Z* isomers; this was the only case, for which apparent *Z*↔*E* isomerization was recorded).

Alcohol **9**: $[\alpha]_D^{23}$ = +16 (*c* 0.9, $CHCl_3$). TLC (20% ethyl acetate in hexane, PMA): R_f = 0.35. ^1H NMR (500 MHz, $CDCl_3$, 8:1 mixture of *Z*:*E* isomers; only signals corresponding to the major isomer are listed) δ: 4.14 (p, *J* = 3.5 Hz, 1H), 3.85 (s, 3H), 2.90 (s, 1H), 2.45–2.29 (m, 1H), 2.17 (dq, *J* = 13.2, 7.6 Hz, 1H), 1.81–1.70 (m, 1H), 1.54 (dp, *J* = 14.4, 7.3 Hz, 1H), 1.08 (t, *J* = 7.6 Hz, 3H), 0.94 (t, *J* = 7.5 Hz, 3H). $^{13}C\{^1H\}$ NMR (126 MHz, $CDCl_3$, 8:1 mixture of *Z*:*E* isomers; only signals corresponding to the major isomer are listed) δ: 162.1, 73.2, 61.8, 27.7, 19.5, 10.7, 9.3. FTIR (neat), cm^{-1}: 3398, 2966, 2938, 2879, 1601, 1462, 1377, 1328, 1097, 1048, 972, 922, 865. HRMS (APCI): Calcd for $[C_7H_{15}NO_2+H]^+$: 146.1176, found: 146.1173.

Conversion of alcohol 9 to 4-nitrobenzoate 30

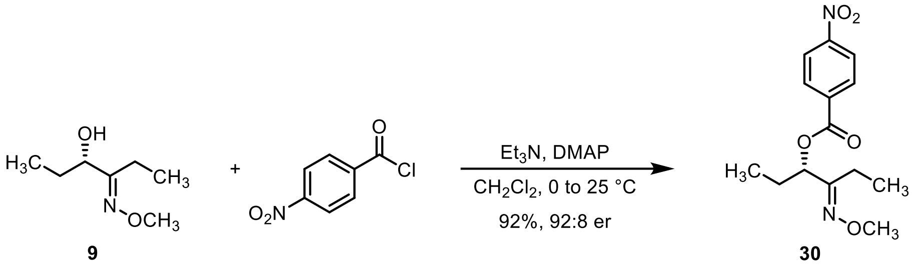

*Note: 4-Nitrobenzoyl derivative of alcohol **9** (product **30**) was prepared for the purpose of enantiomer ratio determination using HPLC analysis.*

Triethylamine (116 µL, 0.83 mmol, 2.0 equiv) and 4-dimethylaminopyridine (5.0 mg, 41.3 mmol, 0.1 equiv) were added to a stirred solution of alcohol **9** (60 mg, 0.41 mmol, 1 equiv) in dichloromethane (1.5 mL) at 0 °C. After 5 min of stirring at 0 °C, 4-nitrobenzoyl chloride (153 mg, 0.83 mmol, 2.0 equiv) was slowly added as a solution in dichloromethane (0.5 mL) at 0 °C. The resulting mixture was allowed to warm to 25 °C and stirred at this temperature for 16 h (TLC: 20% ethyl acetate in hexane). Then, water (10 mL) and dichloromethane (8.0 mL) were added. The mixture was extracted with dichloromethane (3 × 10 mL), the combined organic phases were washed with brine (10 mL), dried over anhydrous magnesium sulfate, and concentrated in vacuo. The obtained residue was purified by FCC (gradient elution with 10–12% ethyl acetate in hexane) to provide 4-nitrobenzoyl ester **30** as a colorless oil (112 mg, 92%). The enantiomeric purity of **30** was determined by HPLC (92:8 er).

4-Nitrobenzoyl ester **30**: $[\alpha]_D^{23}$ = +32 (*c* 0.9, $CHCl_3$). TLC (20% ethyl acetate in hexane): R_f = 0.55. ^1H NMR (500 MHz, $CDCl_3$) δ: 8.32–8.25 (m, 2H), 8.25–8.15 (m, 2H), 5.50 (dd, *J* = 7.6, 6.4 Hz, 1H), 3.86 (s, 3H), 2.46–2.25 (m, 2H), 2.11–1.83 (m, 2H), 1.11 (t, *J* = 7.6 Hz, 3H), 1.00 (t, *J* = 7.4 Hz, 3H). $^{13}C\{^1H\}$ NMR (126 MHz, $CDCl_3$) δ: 164.0, 158.5, 150.8, 135.8, 130.9, 123.7, 77.8, 62.0, 25.5, 19.5, 10.9, 10.0. FTIR (neat), cm^{-1}: 2972, 2938, 2879, 1724, 1607, 1526, 1462, 1409, 1343, 1318, 1300, 1263, 1100, 1046, 1014, 928, 863. HRMS (APCI): Calcd for $[C_{14}H_{18}N_2O_5+H]^+$: 295.1288, found: 295.1291.

Enantioselective transfer hydrogenation of 4,5-dihydroisoxazole substrate 10

Synthesis of ethyl 4,5-dihydroisoxazole-3-carboxylate 32

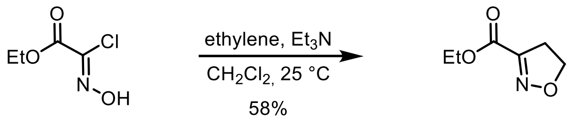

31 **32**

A mixture of ethyl 2-chloro-2-(hydroxyimino)acetate **31** (2.75 g, 18.1 mmol, 1 equiv) in dichloromethane (60 mL) was saturated with ethylene (balloon) by bubbling for 10 min. Then, triethylamine (2.53 mL, 18.1 mmol, 1.0 equiv) was added dropwise over 2 h by syringe pump at 23 °C. The reaction mixture was stirred for 20 h under ethylene atmosphere (balloon) and then was quenched by addition of 0.5 M aqueous solution of hydrochloric acid (25 mL). The resulting mixture was extracted with dichloromethane (3 × 40 mL). Combined organic fractions were washed with brine (40 mL), dried over anhydrous sodium sulphate, the dried solution was filtered, and the filtrate was concentrated in vacuo. The obtained residue was purified by FCC (5–20% gradient of ethyl acetate in cyclohexane) to provide 4,5-dihydroisoxazolyl ester **32**[7] as a colorless oil (1.51 g, 58%).

Ester **32**: TLC: (30% ethyl acetate in hexane) R_f= 0.45. ^{1}H NMR (500 MHz, $CDCl_3$) δ: 4.52 (t, *J* = 10.7 Hz, 2H), 4.35 (q, *J* = 7.1 Hz, 2H), 3.20 (t, *J* = 10.7 Hz, 2H), 1.36 (t, *J* = 7.1 Hz, 3H). ^{13}C{^{1}H} NMR (126 MHz, $CDCl_3$) δ: 160.8, 152.0, 71.4, 62.2, 33.9, 14.2. FTIR (neat), cm^{-1}: 3453, 2984, 2941, 2903, 1715, 1588, 1381, 1334, 1256, 1173, 1120, 1018, 919, 860, 521, 750. HRMS (APCI): Calcd for $[C_6H_9NO_3+H]^+$: 144.0655, found: 144.0654.

Synthesis of ethyl 4,5-dihydroisoxazole-3-carboxylic acid 33

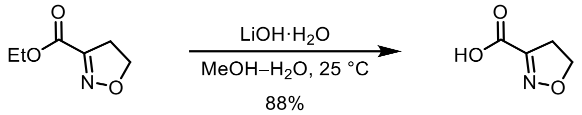

32 **33**

Lithium hydroxide monohydrate (247 mg, 5.88 mmol, 1.1 equiv) was added to a solution of ester **32** (764 mg, 5.34 mmol, 1 equiv) in methanol⍰water (3:1, 18 mL). The resulting mixture was stirred for 19 h at 25 °C. Then, methanol was evaporated in vacuo, and the remaining aqueous residue was acidified to pH = 1 using 1M aqueous solution of hydrochloric acid. The acidified aqueous solution was extracted with dichloromethane (15 × 25 mL; sodium chloride was added to keep the aqueous phase saturated and facilitate the extraction of acid **33**). Combined organic fractions were dried over anhydrous sodium sulfate, filtered, and concentrated in vacuo to provide carboxylic acid **33**[8] as a white solid (537 mg, 88%).

Carboxylic acid **33**: ^{1}H NMR (500 MHz, $CDCl_3$) δ: 10.29 (s, 1H), 4.62 (t, *J* = 10.8 Hz, 2H), 3.23 (t, *J* = 10.8 Hz, 2H). ^{13}C{^{1}H} NMR (126 MHz, $CDCl_3$) δ: 163.8, 151.4, 72.3, 33.0. FTIR (neat), cm^{-1}: 3000, 2970, 2890, 2666, 2581, 1691, 1583.3, 1460, 1430, 1325, 1276, 114, 921, 846, 742. HRMS (APCI): Calcd for $[C_4H_5NO_3+Cl]^-$: 149.9963, found: 149.9965.

Synthesis of *N*-methoxy-*N*-methyl-4,5-dihydroisoxazole-3-carboxamide 34

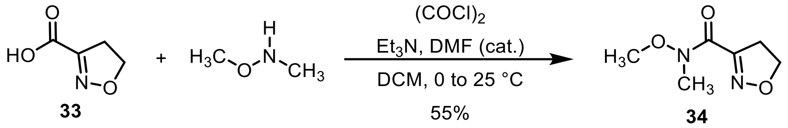

Oxalyl chloride (0.48 mL, 5.57 mmol, 1.3 equiv) was added dropwise to a cold solution (0 °C) of 4,5-dihydroisoxazole-3-carboxylic acid **33** (493 mg, 4.29 mmol, 1 equiv) in dichloromethane (20 mL) and then 4 drops of anhydrous *N,N*-dimethylformamide were added. The solution was stirred for 1.5 h at 0 °C. Then, *N,O*-dimethylhydroxylamine (543 mg, 5.57 mmol, 1.3 equiv) was added, followed by dropwise addition (over 30 min) of triethylamine (2.9 mL, 15.00 mmol, 3.5 equiv) at 0 °C. Then, the reaction mixture was allowed to warm to 23 °C and stirred for 19 h. Subsequently, 1M aqueous solution of hydrochloric acid (15 mL) and water (20 mL) were sequentially added. The aqueous phase was extracted with dichloromethane (4 × 25 mL). Combined organic phases were washed with brine (20 mL), dried over anhydrous sodium sulfate, filtered, and concentrated in vacuo. The residue was purified by FCC (gradient elution with 30–40% ethyl acetate in cyclohexane) to provide Weinreb amide **34** as a yellow oil (370 mg, 55 %).

Weinreb amide **34**: TLC (50% ethyl acetate in hexane): R_f= 0.35. ^{1}H NMR (500 MHz, $CDCl_3$) δ: 4.41 (t, *J* = 10.5 Hz, 2H), 3.77 (s, 3H), 3.34–3.24 (m, 5H). ^{13}C{^{1}H} NMR (126 MHz, $CDCl_3$) δ: 152.8, 69.5, 62.2, 36.3 (some signals could not be observed at this stage). FTIR (neat), cm^{-1}: 3567, 2977, 2939, 2898, 1642, 1589, 1436, 1391, 1301, 1207, 1172, 980, 905, 869, 835, 718, 662., 558. HRMS (APCI): Calcd for $[C_6H_{10}N_2O_3+H]^+$: 159.0764, found: 159.0764.

Synthesis of 1-(4,5-dihydroisoxazol-3-yl)propan-1-one 10

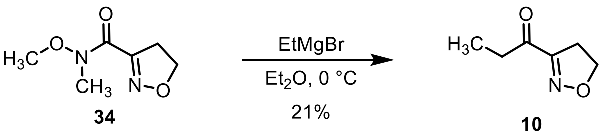

A solution of ethylmagnesium bromide in diethyl ether (3M, 0.82 mL, 2.47 mmol, 1.1 equiv) was added dropwise over 30 min (by syringe) to a solution of Weinreb amide **34** (354 mg, 2.24 mmol, 1 equiv) in diethyl ether (9 mL) at 0 °C. The resulting mixture was stirred for 20 min at 0 °C. Then, a saturated aqueous solution of ammonium chloride (20 mL) was added. The mixture was extracted with dichloromethane (3 × 20 mL). Combined organic fractions were washed with brine (20 mL), dried over anhydrous sodium sulfate, filtered, and concentrated in vacuo. The resulting residue was purified by FCC (10–15% gradient of diethyl ether in pentane) to provide ketone **10** as a colorless oil (60 mg, 21%). The product was relatively volatile.

Ketone **10**: TLC (60% diethyl ether in pentane): R_f= 0.60. ^{1}H NMR (500 MHz, $CDCl_3$) δ: 4.49 (t, *J* = 10.7 Hz, 2H), 3.12 (t, *J* = 10.7 Hz, 2H), 2.92 (q, *J* = 7.4 Hz, 2H), 1.15 (t, *J* = 7.4 Hz, 3H). ^{13}C{^{1}H} NMR (126 MHz, $CDCl_3$) δ: 196.2, 157.9, 71.6, 32.8, 32.6, 8.0. FTIR (neat), cm^{-1}): 2923, 2853, 1755, 1691, 1665, 1578, 1461, 1380, 1221, 323, 907, 846.

Synthesis of (*S*)-1-(4,5-dihydroisoxazol-3-yl)propan-1-ol 11

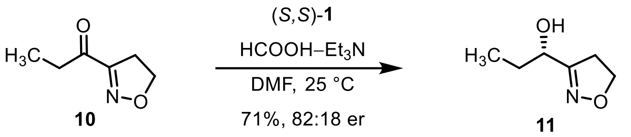

A solution of (*S*,*S*)-**1** (2.5 mg, 3.9 μmol, 0.02 equiv) in *N*,*N*-dimethylformamide (0.5 mL) was evacuated and backfilled with argon (4 cycles). Then, 4,5-dihydroisoxazole ketone **10** (25 mg, 0.20 mmol, 1 equiv) was added as a solution in *N*,*N*-dimethylformamide (0.5 mL), and the mixture was stirred for 5 min in order to obtain a clear solution. Then, argon was bubbled through the solution for 10 min (outlet needle), and a double-layered balloon filled with argon was attached. A mixture of formic acid and triethylamine (5:2 by volume, 0.1 mL) was added, followed by stirring for 19 h at 25 °C (TLC: 70% diethyl ether in pentane). Then, ice-cold water (15 mL) was added, and the resulting mixture was extracted with diethyl ether (3 × 25 mL) and ethyl acetate (2 × 15 mL). The combined organic phases were washed with brine (15 mL), dried over anhydrous sodium sulfate, the dried solution was filtered, and the filtrate was concentrated in vacuo. The obtained residue was purified by FCC (50% diethyl ether in pentane) to provide alcohol **11** as a yellow oil (18 mg, 71%). The enantiomeric purity of **11** was determined by GC (82:18 er).

<u>Alcohol **11**:</u> $[\alpha]_D^{23}$ = –7.9 (c 1.3, $CHCl_3$). TLC (70% diethyl ether in pentane): R_f = 0.15. ^{1}H NMR (500 MHz, $CDCl_3$) δ: 4.49 (t, *J* = 6.5 Hz, 1H), 4.34 (ddd, *J* = 10.6, 9.3, 2.1 Hz, 2H), 3.09–2.89 (m, 2H), 2.31 (br s, 1H), 1.80–1.67 (m, 2H), 0.99 (t, *J* = 7.4 Hz, 3H). $^{13}C\{^1H\}$ NMR (126 MHz, $CDCl_3$) δ: 161.3, 69.3, 68.9, 34.1, 28.0, 9.5. FTIR (neat), cm^{-1}:): 3392, 2967, 2934, 2880, 1459, 1436, 1320, 1099, 1042, 1018, 976, 863, 818. HRMS (APCI): Calcd for $[C_6H_{11}NO_2+H]^+$: 130.0863, found: 130.0864.

<u>Enantioselective transfer hydrogenation of isoxazole substrate 12</u>

Synthesis of *N*-methoxy-*N*-methylisoxazole-3-carboxamide 35

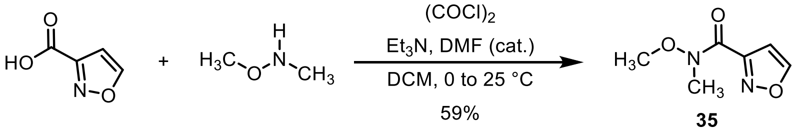

Oxalyl chloride (0.91 mL, 10.54 mmol, 1.3 equiv) was added dropwise to an ice-cold solution of isoxazole-3-carboxylic acid (916 mg, 8.11 mmol, 1 equiv) in dichloromethane (35 mL), and then 4 drops of dimethyl formamide were added. The resulting mixture was stirred for 1 h at 0 °C. *N*,*O*-Dimethylhydroxylamine (1.03 g, 10.5 mmol, 1.3 equiv) was added and then triethylamine (3.95 mL, 28.4 mmol, 3.5 equiv) was added dropwise over 30 min (by hand) at 0 °C. The mixture was allowed to warm to 25 °C and stirred at this temperature for 20 h. Subsequently, 1M hydrochloric acid (15 mL) was added to quench the reaction. Then, water (50 mL) was added, the organic phase was separated, and the aqueous phase was extracted with dichloromethane (3 × 40 mL). Organic phases were combined, washed sequentially by water (30 mL) and brine (20 mL), and dried over anhydrous sodium sulfate. The dried solution was filtered

and concentrated in vacuo. Obtained residue was purified by FCC (40% ethyl acetate in cyclohexane) to provide Weinreb amide **35** as a yellow oil (926 mg, 73 %).

Weinreb amide **35**: TLC (50% ethyl acetate in hexane): R_f= 0.45. ^{1}H NMR (500 MHz, $CDCl_3$) δ: 8.47 (d, *J* = 1.7 Hz, 1H), 6.71 (s, 1H), 3.79 (s, 3H), 3.40 (s, 3H). $^{13}C\{^1H\}$ NMR (126 MHz, $CDCl_3$) δ: 158.6 (two carbons), 157.0, 105.7, 62.5, 33.1. FTIR (neat), cm^{-1}: 3126, 2981, 2940, 1650, 1551, 1488, 1458, 1420, 1396, 1373, 1182, 1116, 1088, 1001, 965, 896, 857, 757, 595. HRMS (APCI): Calcd for $[C_6H_8N_2O_3+H]^+$: 157.0608, found: 157.0607.

Synthesis of 1-(isoxazol-3-yl)propan-1-one 12

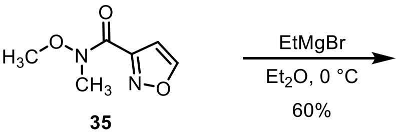

35 **12**

A solution of ethylmagnesium bromide in diethyl ether (3*M*, 0.58 mL, 1.74 mmol, 1.1 equiv) was added dropwise over 30 min to a solution of Weinreb amide **35** (247 mg, 1.58 mmol, 1 equiv) in diethyl ether (7 mL) at 0 °C. The resulting mixture was stirred for 20 min at 0 °C and subsequently quenched by aaddition of a saturated solution of ammonium chloride (5 mL), ethyl acetate (5 mL) and water (20 mL). The mixture was extracted with ethyl acetate (3 × 30 mL). Combined organic phases were washed with brine (30 mL), dried over anhydrous sodium sulfate, filtered, and concentrated in vacuo. The resulting residue was purified by FCC (30% ethyl acetate in cyclohexane) to provide ketone **12** as a colorless oil (119 mg, 60%). The product was relatively volatile.

Ketone **12**: TLC (30% ethyl acetate in hexane): R_f= 0.75. ^{1}H NMR (500 MHz, $CDCl_3$) δ: 8.47 (d, *J* = 1.7 Hz, 1H), 6.74 (d, *J* = 1.7 Hz, 1H), 3.10 (q, *J* = 7.3 Hz, 2H), 1.23 (t, *J* = 7.3 Hz, 3H). $^{13}C\{^1H\}$ NMR (126 MHz, $CDCl_3$) δ: 195.0, 160.9, 159.7, 103.4, 33.7, 7.6. FTIR (neat), cm^{-1}: 3161, 3132, 2982, 2942, 2920, 2361, 2330, 1704, 1422, 1347, 1270, 1114, 1045, 928, 915, 896, 785. HRMS (APCI): Calcd for $[C_6H_7NO_2+H]^+$: 126.0550, found: 126.0555.

Synthesis of (*S*)-1-(isoxazol-3-yl)propan-1-ol 13

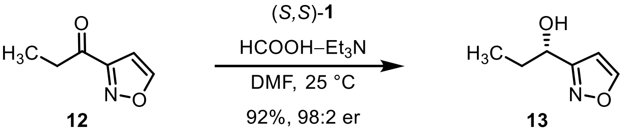

12 **13**

A solution of (*S,S*)-**1** (24.8 mg, 39.0 μmol, 0.02 equiv) in *N,N*-dimethylformamide (1.0 mL) was evacuated and backfilled with argon (4 cycles). Then, isoxazole ketone **12** (244 mg, 1.95 mmol, 1 equiv) was added as a solution in *N,N*-dimethylformamide (0.7 mL), and the mixture was stirred for 5 min in order to obtain a clear solution. Then, argon was bubbled through the solution for 10 min (outlet needle), and a double-layered balloon filled with argon was attached. A mixture of formic acid and triethylamine (5:2 by volume, 0.98 mL) was added, followed by stirring for 6 h

at 25 °C (TLC: 60% ethyl acetate in hexane, UV). Then, ice-cold water (25 mL) was added, and the resulting mixture was extracted with diethyl ether (3 × 20 mL) and ethyl acetate (2 × 15 mL). The combined organic phases were washed with brine (15 mL), dried over anhydrous sodium sulfate, the dried solution was filtered, and the filtrate was concentrated in vacuo. The obtained residue was purified by FCC (50% diethyl ether in cyclohexane) to provide alcohol **13** as a colorless oil (225 mg, 92%). The enantiomeric purity of **13** was determined by HPLC (98:2 er).

Alcohol **13**: $[\alpha]_D^{23} = -41$ (*c* 0.8, $CHCl_3$). TLC (60% ethyl acetate in hexane): $R_f = 0.6$. ^1H NMR (500 MHz, $CDCl_3$) δ: 8.35 (d, *J* = 1.4 Hz, 1H), 6.36 (d, *J* = 1.6 Hz, 1H), 4.84 (t, *J* = 6.5 Hz, 1H), 2.45 (br s, 1H), 1.90–1.82 (m, 2H), 0.98 (t, *J* = 7.4 Hz, 3H). $^{13}C\{^1H\}$ NMR (126 MHz, $CDCl_3$) δ: 165.8, 158.7, 102.3, 68.4, 30.0, 9.5. FTIR (neat), cm^{-1}: 3365, 2970, 2937, 2880, 1562, 1458, 1422, 1367, 1120, 1091, 1042, 1022, 993, 974, 865, 784, 592. HRMS (APCI): Calcd for $[C_6H_9NO_2+H]^+$: 128.0706, found: 128.0705.

Enantioselective transfer hydrogenation of phenyl substrate *Z*-3k using alternative diamine ligands:

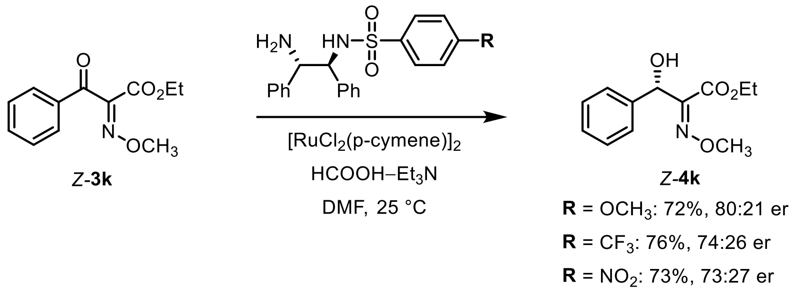

In situ catalyst preparation. A 5-mL round-bottom flask equipped with a magnetic stirring-bar was charged successively with $[RuCl_2(P\text{-cymene})]_2$ (21 mg, 34.0 μmol, 0.01 equiv), a diamine ligand (0.02 equiv) and anhydrous *N*,*N*-dimethylformamide (2 mL). The mixture was degassed by purging with argon and was then heated for 25 min at 80 °C. The resulting tinted color solution was allowed to cool down to 23 °C and used directly as a catalyst in the transfer hydrogenation.

Transfer hydrogenation. A solution of the above prepared (*S,S*) catalyst (0.2 mL, 6.8 μmol, 0.02 equiv) in *N,N*-dimethylformamide was added to α-methoxyimino ester *Z*-**3k** (80 mg, 0.34 mmol, 1 equiv) in anhydrous *N*,*N*-dimethylformamide (0.5 mL), and the mixture was stirred for 5 min in order to obtain a clear solution. Then, argon was bubbled through the solution for 15 min (outlet needle), and a double-layered balloon filled with argon was attached. A freshly prepared mixture of formic acid and triethylamine (5:2 by volume, 0.2 mL) was added, followed by stirring for 20 h at 25 °C (TLC: 30% ethyl acetate in hexane, UV, PMA). Then, ice-cold water (10 mL) was added, and the resulting mixture was extracted with ethyl acetate (3 × 12 mL). The combined organic phases were washed with brine (10 mL), dried over anhydrous sodium sulfate, the dried solution was filtered, and the filtrate was concentrated in vacuo. The obtained residue was purified by FCC (gradient elution with 15–20% ethyl acetate in hexane) to provide alcohol *Z*-**4k** as a colorless oil. The enantiomeric purity of *Z*-**4k** was determined by HPLC.

X-Ray Crystallography

Diffraction data were collected on Rigaku Synergy rotating anode X-ray source diffractometers with hybrid pixel array detectors and kappa goniometers using either Mo *Kα* (*E*-**7**, *Z*-**3a**) or Cu *Kα* (*Z*-**4n**, **4p-amine**) radiation. *CrysAlisPro* was used for data collection and data reduction.[9a] The structures were solved by intrinsic phasing and refined by full matrix least-squares methods on F^2 using *SHELXT* and *SHELXL*.[9b, 9c] All non-hydrogen atoms were refined anisotropically and the hydrogen atoms were refined as riding on their carrier atoms. Crystal data and refinement parameters are gathered in Table S1. The supplementary crystallographic data for this paper can be obtained free of charge from The Cambridge Crystallographic Data Centre via www.ccdc.cam.ac.uk/data_request/cif.

Table S1. Crystallographic information for *Z*-**3a**, *E*-**7**, *Z*-**4n**, and **4p-amine**.

	Z-**3a**	*E*-**7**	*Z*-**4n**	**4p-amine** *S,S* diastereomer
CCDC No	2286956	2286955	2286957	2366712
Crystal data				
Chemical formula	$C_8H_{13}NO_4$	$C_{11}H_{11}NO_4$	$C_{18}H_{19}NO_4$	$C_{11}H_{22}N_2O_5$
M_r	187.19	221.21	313.34	262.30
Crystal system, space group	Triclinic, $P\bar{1}$	Orthorhombic, $Pna2_1$	Orthorhombic, $P2_12_12_1$	Orthorhombic, P2_1 2_1 2_1
Temperature (K)	120	120	120	120
a (Å)	7.3114 (3)	18.4611 (6)	7.3082 (1)	5.0433 (1)
b (Å)	8.0769 (3)	4.7375 (1)	10.6552 (1)	11.6613 (1)
c (Å)	9.4621 (3)	24.3036 (7)	20.6354 (2)	23.9884 (3)
α (°)	105.433 (3)	90	90	90
β (°)	94.838 (3)	90	90	90
γ (°)	112.780 (4)	90	90	90
V (Å^3)	485.45 (3)	2125.58 (10)	1606.89 (3)	1410.79 (4)
Z	2	8	4	4
μ (mm^{-1})	0.10	0.11	0.75	0.81
Crystal size (mm)	0.25 × 0.10 × 0.05	0.25 × 0.20 × 0.15	0.30 × 0.25 × 0.25	0.30 × 0.25 × 0.25
Data collection				
Absorption correction	Multi-scan	Multi-scan	Multi-scan	Multi-scan
T_{min}/T_{max}	0.762	0.118	0.840	0.761
Measured, unique, observed [$I > 2\sigma(I)$] data	12489, 1774, 1588	13446, 3716, 3558	36220, 2945, 2925	8054, 2548, 2535
R_{int}	0.047	0.025	0.032	0.018
$(\sin \theta/\lambda)_{max}$ (Å^{-1})	0.602	0.602	0.602	0.602
Refinement				
$R[F^2 > 2\sigma(F^2)]$, $wR(F^2)$, S	0.030, 0.079, 1.09	0.063, 0.150, 1.06	0.023, 0.059, 1.08	0.044, 0.120, 1.08
Reflections, parameters, restraints	1774, 121, 0	3716, 295, 217	2945, 212, 0	2548, 175, 2
$\Delta\rho_{max}$, $\Delta\rho_{min}$ (e Å^{-3})	0.20, −0.19	0.99, −0.22	0.13, −0.15	0.77, −0.22

Figure S1. Molecular structure of *Z*-**3a**. Thermal ellipsoids are drawn at the 50% probability level.

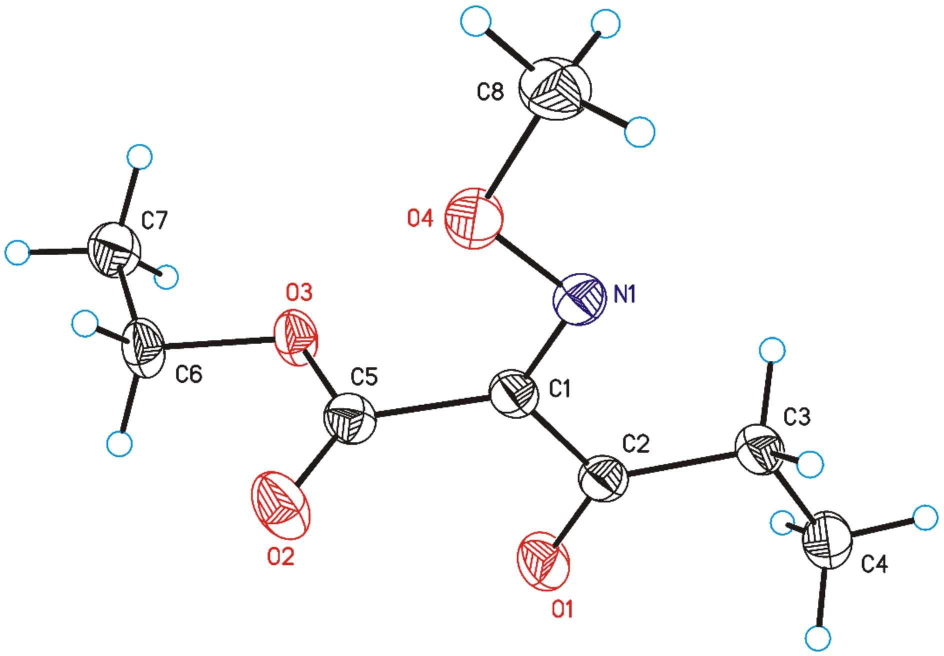

Figure S2. Molecular structure of *E*-**7**.[9d] Thermal ellipsoids are drawn at the 50% probability level.

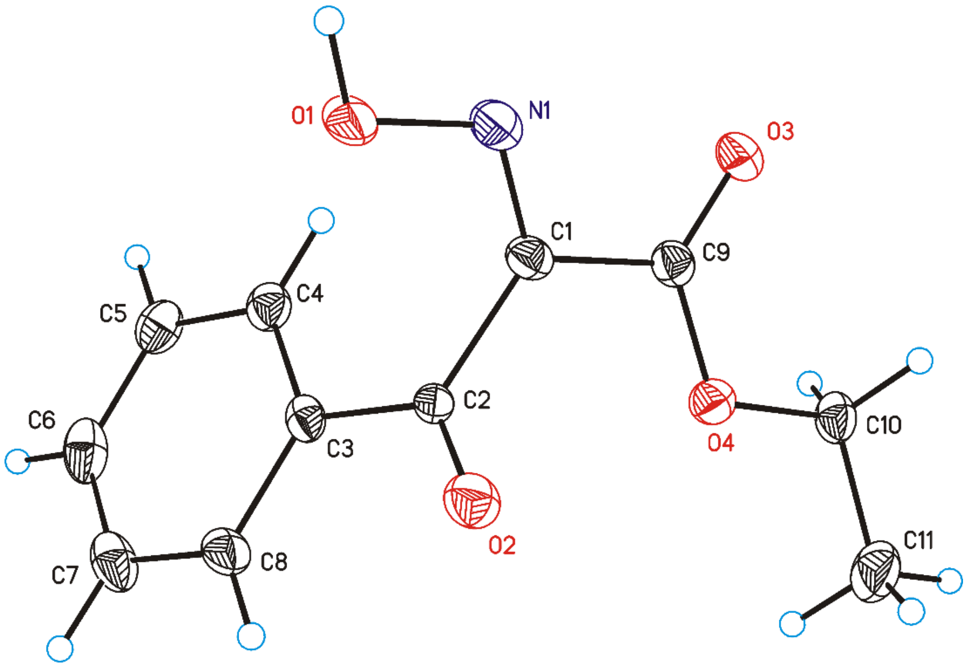

Figure S3a. Molecular structure of *Z*-**4n**. Thermal ellipsoids are drawn at the 50% probability level.

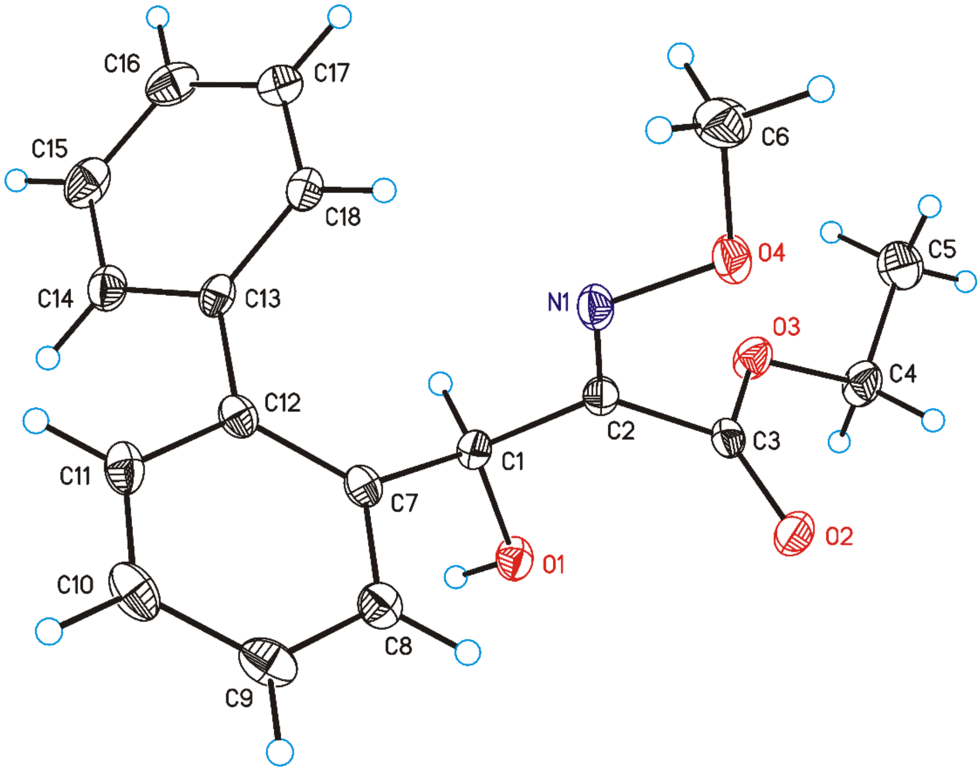

Figure S3b. Molecular structure of **4p-amine** (*S,S* diastereomer). Thermal ellipsoids are drawn at the 50% probability level.

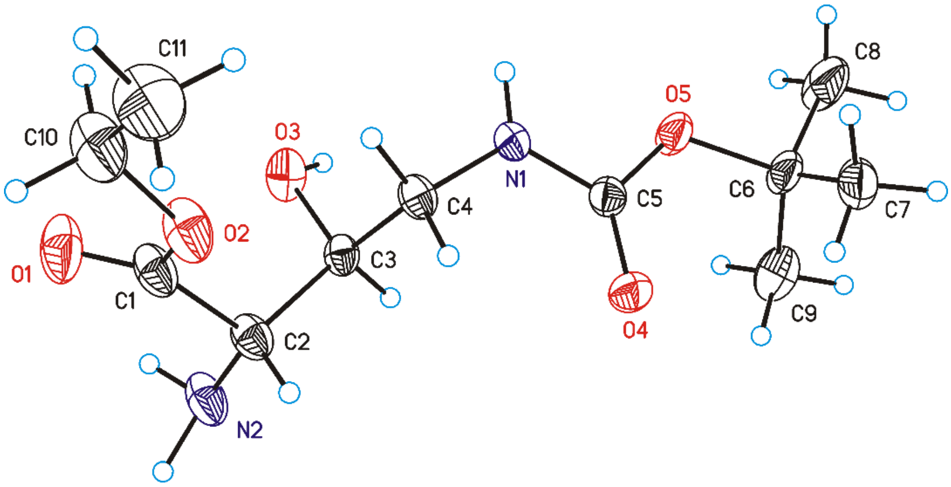

Computational Analysis

The popular hybrid exchange–correlation functional B3LYP[11a,b] (with the global 20% orbital exchange fraction) parameterized via the D3 dispersion model[11c] was used to model Z ↔ E ground and excited state isomerization of the substrate as well as stereoselectivity determining transition states leading to four stereoisomers of product **4a** under LACV3P**++//LACVP**+ level coupled with a polarizable continuum model in dimethylformamide. To include the conformational thermostatistics in these calculations, conformational sampling based on the Monte Carlo method with the OPLS4[11d] force field was used. Each minimum or transition state was limited to ten lowest-energy conformers, each of which was subsequently refined by DFT and Boltzmann averaged to obtain the final properties. The procedure is fully automated as implemented in Reaction Workflow (RXNWF) module of the Schrödinger Materials Science Suite.[10c]

DFT calculations were performed with the Jaguar code[10a] (selected jaguar_keywords 'iuhf=2 nops=1 maxit=150 maxitg=100 iaccg=2 nofail=0 isymm=8 isolv=7 solvent=dimethylformamide'). Pseudospectral method was used to model thermal and photoexcited *Z*-to-*E* isomerization pathway for *Z*-**3a** (selected -jaguar_keywords 'iuhf=2 nops=0 iacc=3 maxit=150 maxitg=100 iaccg=2 nofail=1 isymm=8 isolv=7 solvent=dimethylformamide ts_vet_dist_fac0=0.7' for ground state and 'iuhf=2 nops=0 iacc=3 maxit=150 maxitg=100 iaccg=2 nofail=1 isymm=8 isolv=7 solvent=dimethylformamide' for triplet state). Conformational analysis was performed with MacroModel code[10b] with -dedup_geom_eps 0.25 flag. Reaction Workflow (RXNWF) module of Schrödinger Materials Science Suite[10c] available via graphics user interface (GUI) was used to automate the calculations. For example, the input to model thermal and photoexcited *Z*-to-*E* isomerization pathway for *Z*-**3a** is Cartesian coordinates for three and five DFT pre-optimized stationary points (minima and transition states), whereas the output is thirty and fifty DFT optimized stationary points (ten lowest energy conformers per stationary point were considered) and Boltzmann-averaged properties, respectively. Likewise, the input for Figure 1 is Cartesian coordinates for four DFT pre-optimized transition states, and the output is 40 DFT optimized transition states and Boltzmann-averaged properties. Because two transition states (out of 40) failed, specifically the highest-energy conformers for the transition states leading to *Z*-(*S*)-**4a** and *Z*-(*R*)-**4a** from *Z*-**3a**, the results are reported for the conformer space of 38.

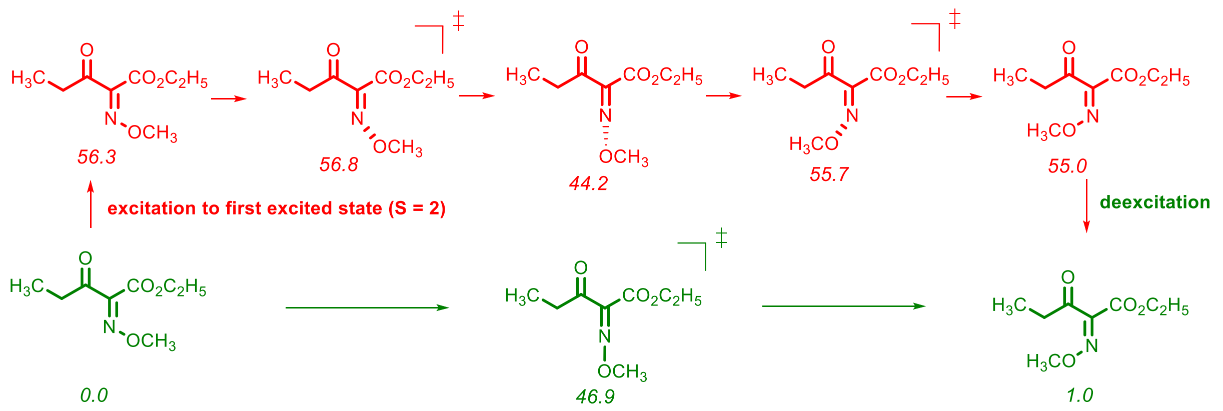

Scheme S1. Thermal (green) and photoexcited (red) *Z*-to-*E* isomerization pathway for *Z*-**3a**. Calculated Relative Ensemble Free Energies are presented in kcal/mol.

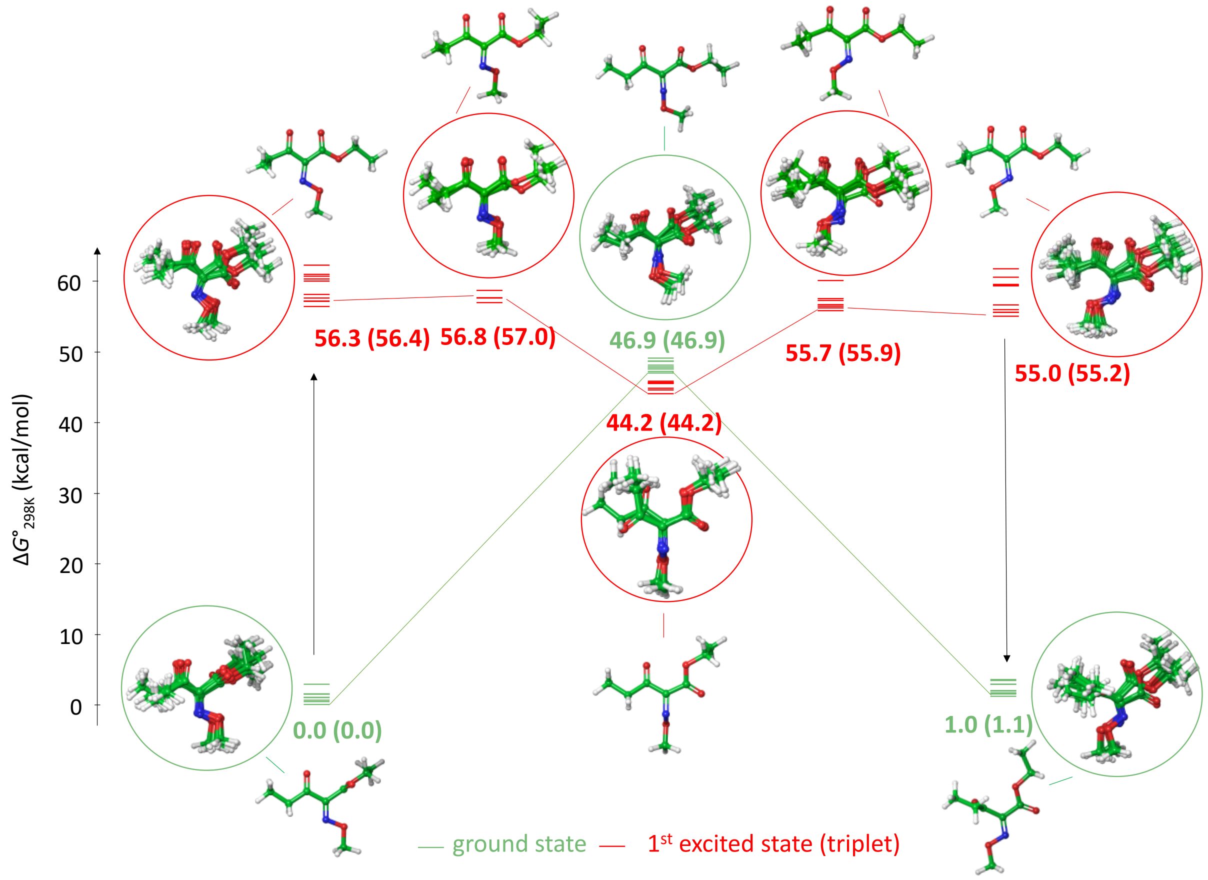

Figure S4. Computed Relative Ensemble Free Energy Profile for Thermal (green) and photoexcited (red) *Z*-to-*E* isomerization pathway for *Z*-**3a**. Boltzmann-averaged relative free energies values are presented in kcal/mol. Lowest-isomer relative free energies values are presented in kcal/mol in brackets. Theory: B3LYP-D3 with 6-311G**++//6-31G**+ in PCM (dimethylformamide).

Computation data

```
Catalyst Transition States Conformers and Final Free Energies
99
iTS1-major-Z_0-5
G = -2623.2962105
N          -1.29530        0.84360       -0.69530
H          -1.96540        1.01310       -1.44190
H          -1.74310        1.12410        0.19600
C           0.96480        1.24090        0.14790
H           0.61180        1.61360        1.11710
C          -0.07080        1.65230       -0.91990
H           0.31620        1.36340       -1.89940
C          -1.03620       -1.82030       -2.69820
C           0.10730       -2.45460       -2.17300
C          -2.27470       -1.99140       -1.98530
C          -1.18690       -3.44710       -0.30540
C          -2.35420       -2.78820       -0.82690
C          -1.02290       -0.96800       -3.95290
H          -1.63540       -0.08200       -3.74030
```

N	1.00030	-0.24430	0.15550
S	1.95680	-0.90270	1.29980
O	2.06180	-0.04420	2.51570
O	1.54750	-2.31340	1.51970
C	2.29900	1.90050	-0.17060
C	2.80720	2.90850	0.65480
C	3.01760	1.53580	-1.31750
C	4.01260	3.54690	0.34090
H	2.25910	3.19340	1.54910
C	4.22010	2.16770	-1.63530
H	2.64580	0.73360	-1.94690
C	4.72180	3.17880	-0.80630
H	4.39690	4.32610	0.99340
H	4.77120	1.86540	-2.52140
H	5.66080	3.66850	-1.04920
C	-0.34970	3.14270	-0.92430
C	0.02350	3.92190	-2.02690
C	-0.97360	3.76240	0.16970
C	-0.21580	5.30020	-2.03970
H	0.50870	3.44890	-2.87690
C	-1.21730	5.13820	0.15700
H	-1.27310	3.17080	1.03050
C	-0.83780	5.91190	-0.94650
H	0.08150	5.89220	-2.90080
H	-1.70260	5.60660	1.00880
H	-1.02670	6.98170	-0.95390
C	0.03090	-3.25090	-0.98480
Ru	-0.81170	-1.20290	-0.49060
H	-1.37520	-0.87420	1.09930
C	3.61610	-0.98810	0.59970
C	3.81420	-1.74970	-0.55590
C	4.67020	-0.28200	1.17560
C	5.07950	-1.79710	-1.13650
C	5.93450	-0.33820	0.58240
C	6.15990	-1.09030	-0.57910
H	-3.15790	-1.45600	-2.31880
H	1.07570	-2.29850	-2.63210
H	4.49850	0.32080	2.05990
H	6.75280	0.22500	1.02350
H	5.22960	-2.38160	-2.04070
H	2.98580	-2.28450	-1.00760
C	7.53140	-1.15760	-1.20780
H	8.11850	-0.26310	-0.97980
H	7.46600	-1.26240	-2.29530
H	8.08840	-2.02370	-0.82850
O	-5.37610	0.59380	1.70260
O	-2.05920	1.04230	1.97300
O	-4.18600	0.15180	-0.17640
O	-5.01090	-2.45150	1.82280
N	-3.74350	-2.03390	2.22980
C	-3.46480	-0.84890	1.83790
C	-2.10320	-0.22960	2.13910
C	-1.31230	-0.85680	3.29080
H	-1.29060	-1.94420	3.17920
H	-0.28730	-0.48970	3.20810
C	-1.91160	-0.46350	4.65030
H	-1.31560	-0.89290	5.46230
H	-1.92030	0.62440	4.77140

H	-2.93880	-0.82840	4.75450
C	-4.45600	0.04440	1.12380
C	-5.05210	1.02040	-0.97980
H	-4.88060	0.67450	-2.00010
H	-6.08930	0.82510	-0.69860
C	-4.68250	2.48500	-0.80630
H	-3.64230	2.67060	-1.08710
H	-5.32430	3.09200	-1.45330
H	-4.82840	2.80670	0.22760
C	-5.25620	-3.78650	2.29780
H	-6.25310	-4.04050	1.93420
H	-4.51660	-4.48040	1.88650
H	-5.23550	-3.81500	3.39160
C	-1.70700	-1.74080	-5.10100
H	-2.72440	-2.04270	-4.83200
H	-1.13580	-2.64270	-5.34960
H	-1.76230	-1.11210	-5.99580
C	0.37350	-0.49260	-4.36980
H	0.28950	0.21770	-5.19780
H	0.99520	-1.32780	-4.71140
H	0.89500	0.00440	-3.54690
C	-1.26810	-4.28430	0.94020
H	-0.29540	-4.33240	1.43430
H	-1.58240	-5.30330	0.68370
H	-2.00050	-3.87010	1.63730
H	-3.29630	-2.86700	-0.29680
H	0.94070	-3.65990	-0.56290

99

iTS1-major-Z_30

G = -2623.2956240

N	-1.27770	0.75290	-0.77300
H	-1.96770	0.92010	-1.50340
H	-1.71050	1.02720	0.12720
C	0.96060	1.19050	0.08900
H	0.57520	1.55800	1.04790
C	-0.06380	1.57560	-1.00070
H	0.33990	1.28070	-1.97190
C	-0.86050	-1.90120	-2.79000
C	0.24500	-2.54520	-2.19970
C	-2.13710	-2.05810	-2.14360
C	-1.15980	-3.52590	-0.41030
C	-2.29120	-2.85770	-0.99490
C	-0.76950	-1.04750	-4.04030
H	-1.37920	-0.15290	-3.85630
N	1.02970	-0.29390	0.10200
S	1.94160	-0.92480	1.29860
O	1.94800	-0.07070	2.52270
O	1.56860	-2.34950	1.49150
C	2.28680	1.87870	-0.19760
C	2.74100	2.91260	0.62750
C	3.05190	1.51570	-1.31430
C	3.93820	3.57880	0.34180
H	2.15660	3.19650	1.49880
C	4.24660	2.17540	-1.60390
H	2.72210	0.69320	-1.94070
C	4.69380	3.21270	-0.77610
H	4.28030	4.37810	0.99370
H	4.83420	1.87460	-2.46690

H	5.62670	3.72420	-0.99680
C	-0.36080	3.06220	-1.02260
C	0.05310	3.84360	-2.10900
C	-1.03490	3.67770	0.04310
C	-0.19460	5.22000	-2.13350
H	0.57770	3.37320	-2.93680
C	-1.28780	5.05150	0.01830
H	-1.36560	3.08630	0.89220
C	-0.86690	5.82760	-1.06830
H	0.13500	5.81350	-2.98170
H	-1.81350	5.51660	0.84770
H	-1.06310	6.89600	-1.08420
C	0.09660	-3.34460	-1.02040
Ru	-0.75550	-1.28760	-0.57280
H	-1.34580	-0.99520	1.01550
C	3.64000	-0.94920	0.69410
C	4.63000	-0.19730	1.32250
C	3.93460	-1.71350	-0.43930
C	5.92880	-0.21040	0.80560
C	5.23280	-1.71740	-0.94330
C	6.25110	-0.96450	-0.33100
H	-2.99360	-1.50530	-2.51490
H	1.24000	-2.39470	-2.60050
H	3.15520	-2.28420	-0.93280
H	5.45910	-2.30440	-1.82990
H	6.69760	0.38740	1.28840
H	4.38330	0.40630	2.18820
C	7.65790	-0.98660	-0.87970
H	7.65650	-1.01400	-1.97410
H	8.19470	-1.87830	-0.53240
H	8.22680	-0.11040	-0.55550
O	-4.18040	-0.09360	-0.52410
O	-2.01100	0.91450	1.90230
O	-5.07010	0.78920	1.36510
O	-5.12200	-2.40520	1.52550
N	-3.86440	-2.06410	2.03200
C	-3.47260	-0.91850	1.62220
C	-2.11170	-0.35880	2.02920
C	-1.41730	-1.04620	3.20810
H	-1.44250	-2.13110	3.07440
H	-0.37270	-0.72820	3.18810
C	-2.06900	-0.64860	4.54200
H	-1.53990	-1.12280	5.37530
H	-2.02910	0.43590	4.68500
H	-3.11720	-0.96190	4.58340
C	-4.28070	-0.04260	0.68880
C	-5.80080	1.79870	0.59250
H	-6.21620	1.32120	-0.29770
H	-6.61170	2.09660	1.25810
C	-4.88920	2.96500	0.24520
H	-4.06490	2.64340	-0.39630
H	-5.46660	3.72820	-0.28690
H	-4.47240	3.41400	1.15140
C	-5.50390	-3.70310	2.01100
H	-6.48010	-3.89980	1.56480
H	-4.78240	-4.45980	1.68740
H	-5.58310	-3.69740	3.10260
C	-1.40270	-1.80820	-5.22510

H	-2.43650	-2.09740	-5.01050
H	-0.83120	-2.71710	-5.44610
H	-1.40300	-1.17710	-6.12000
C	0.65360	-0.59220	-4.38260
H	0.62430	0.11620	-5.21600
H	1.28150	-1.43690	-4.68750
H	1.13670	-0.09890	-3.53450
C	-1.31760	-4.35860	0.83110
H	-1.59030	-5.38520	0.55730
H	-2.10930	-3.95810	1.46890
H	-0.38390	-4.38590	1.39720
H	-3.26120	-2.92190	-0.51550
H	0.97820	-3.76140	-0.54950

99

iTS1-major-Z_30-3

G = -2623.2945124

N	-1.21070	0.85940	-0.77830
H	-1.88500	1.04300	-1.51930
H	-1.65500	1.14100	0.11380
C	1.04020	1.22940	0.09600
H	0.66860	1.60360	1.05780
C	0.02690	1.65100	-0.99090
H	0.42450	1.35420	-1.96400
C	-0.89200	-1.79590	-2.80090
C	0.21700	-2.45550	-2.23550
C	-2.15950	-1.94400	-2.13440
C	-1.16640	-3.43120	-0.42520
C	-2.30060	-2.74830	-0.98740
C	-0.81330	-0.93410	-4.04670
H	-1.40680	-0.03270	-3.84470
N	1.06680	-0.25610	0.10200
S	1.97340	-0.92190	1.28330
O	2.02970	-0.07050	2.50770
O	1.55290	-2.33310	1.47960
C	2.38560	1.87990	-0.19140
C	2.87910	2.88750	0.64350
C	3.13040	1.50740	-1.31880
C	4.09520	3.51830	0.35760
H	2.31040	3.17850	1.52280
C	4.34360	2.13220	-1.60900
H	2.76930	0.70530	-1.95450
C	4.83040	3.14300	-0.77070
H	4.46770	4.29730	1.01720
H	4.91450	1.82460	-2.48070
H	5.77760	3.62700	-0.99190
C	-0.22330	3.14640	-0.99900
C	0.20270	3.92240	-2.08440
C	-0.85810	3.77680	0.08250
C	0.00540	5.30720	-2.09180
H	0.69820	3.44170	-2.92410
C	-1.06010	5.15950	0.07500
H	-1.19430	3.18910	0.93240
C	-0.62690	5.92980	-1.01090
H	0.34430	5.89640	-2.93930
H	-1.55280	5.63610	0.91800
H	-0.78220	7.00500	-1.01410
C	0.08060	-3.25640	-1.05570
Ru	-0.74210	-1.19520	-0.58090

H	-1.32920	-0.87850	1.00140
C	3.65990	-1.00680	0.65050
C	4.68840	-0.29560	1.26400
C	3.90570	-1.77640	-0.49110
C	5.97670	-0.35540	0.72430
C	5.19380	-1.82680	-1.01810
C	6.25040	-1.11600	-0.42060
H	-3.01720	-1.38240	-2.48950
H	1.20590	-2.31470	-2.65420
H	3.09660	-2.31500	-0.97290
H	5.38200	-2.41770	-1.91090
H	6.77570	0.21110	1.19550
H	4.48010	0.31350	2.13590
C	7.64550	-1.18910	-0.99410
H	8.15100	-2.10630	-0.66660
H	8.25540	-0.34090	-0.67000
H	7.62430	-1.20300	-2.08860
O	-4.14780	0.12260	-0.47010
O	-2.00210	1.03910	1.88490
O	-5.19960	0.74190	1.44280
O	-5.06740	-2.32930	1.54830
N	-3.81130	-1.96670	2.04180
C	-3.44790	-0.80950	1.63730
C	-2.08980	-0.23360	2.02260
C	-1.37200	-0.90680	3.19560
H	-1.38480	-1.99240	3.06610
H	-0.33170	-0.57610	3.16180
C	-2.01320	-0.51270	4.53570
H	-1.46800	-0.97650	5.36430
H	-1.98670	0.57280	4.67450
H	-3.05650	-0.84020	4.59060
C	-4.29870	0.06020	0.73660
C	-6.10180	1.63480	0.71060
H	-6.41750	2.35730	1.46420
H	-5.52730	2.14510	-0.06580
C	-7.27700	0.86040	0.13780
H	-7.81990	0.33820	0.93130
H	-7.96480	1.55860	-0.35080
H	-6.94050	0.13010	-0.60280
C	-5.42000	-3.63620	2.03180
H	-6.39850	-3.84730	1.59750
H	-4.69000	-4.37860	1.69460
H	-5.48490	-3.63740	3.12440
C	-1.48000	-1.67710	-5.22440
H	-2.51370	-1.95270	-4.99230
H	-0.92620	-2.59240	-5.46270
H	-1.48810	-1.03940	-6.11460
C	0.60920	-0.49580	-4.41330
H	0.57340	0.22070	-5.23950
H	1.21860	-1.34640	-4.73840
H	1.11630	-0.01750	-3.57050
C	-1.31090	-4.27090	0.81310
H	-1.61450	-5.28870	0.53910
H	-2.07590	-3.85710	1.47460
H	-0.36360	-4.32310	1.35410
H	-3.26360	-2.80960	-0.49370
H	0.96600	-3.68390	-0.60150

99

iTS1-major-Z_0
G = -2623.2945112

N	-1.21500	0.87490	-0.75270
H	-1.90010	1.06000	-1.48340
H	-1.64650	1.15350	0.14730
C	1.04780	1.24770	0.09120
H	0.69250	1.62440	1.05820
C	0.01840	1.66900	-0.98050
H	0.40330	1.37460	-1.95940
C	-0.93040	-1.77830	-2.78060
C	0.19350	-2.43170	-2.23700
C	-2.18460	-1.93590	-2.09220
C	-1.15000	-3.41720	-0.40200
C	-2.29840	-2.74050	-0.94250
C	-0.87990	-0.91540	-4.02700
H	-1.47620	-0.01830	-3.81410
N	1.07170	-0.23740	0.10060
S	1.99880	-0.90260	1.26500
O	2.08010	-0.04780	2.48550
O	1.57860	-2.31210	1.47270
C	2.39000	1.89490	-0.21860
C	3.11630	1.51850	-1.35670
C	2.89870	2.90300	0.60640
C	4.32630	2.13960	-1.66700
H	2.74370	0.71600	-1.98520
C	4.11180	3.53050	0.30030
H	2.34460	3.19710	1.49410
C	4.82840	3.15120	-0.83870
H	4.88300	1.82870	-2.54660
H	4.49630	4.31010	0.95240
H	5.77330	3.63250	-1.07560
C	-0.23370	3.16430	-0.98320
C	0.17660	3.94200	-2.07360
C	-0.85520	3.79240	0.10760
C	-0.02300	5.32670	-2.07740
H	0.66160	3.46270	-2.92020
C	-1.05950	5.17490	0.10360
H	-1.17910	3.20330	0.96130
C	-0.64210	5.94680	-0.98750
H	0.30370	5.91720	-2.92890
H	-1.54180	5.65010	0.95340
H	-0.79940	7.02180	-0.98750
C	0.08390	-3.23430	-1.05570
Ru	-0.74160	-1.17920	-0.56520
H	-1.31580	-0.86590	1.02500
C	3.67270	-0.99360	0.60130
C	4.71470	-0.28460	1.19490
C	3.89440	-1.76200	-0.54560
C	5.99120	-0.34470	0.62870
C	5.17160	-1.81320	-1.09920
C	6.24050	-1.10360	-0.52330
H	-3.05240	-1.37990	-2.43110
H	1.17340	-2.28450	-2.67430
H	3.07530	-2.29940	-1.01120
H	5.34040	-2.40290	-1.99670
H	6.80030	0.22100	1.08360
H	4.52490	0.32340	2.07180
C	7.62470	-1.17520	-1.12320

H 7.58160 -1.26760 -2.21300
H 8.16670 -2.04990 -0.74210
H 8.21380 -0.28780 -0.87320
O -4.15130 0.11980 -0.40230
O -1.97710 1.04870 1.91490
O -5.17950 0.73460 1.52500
O -5.03170 -2.33450 1.63450
N -3.76840 -1.96660 2.10580
C -3.41720 -0.80800 1.69450
C -2.05480 -0.22550 2.05510
C -1.31520 -0.89240 3.21830
H -1.32370 -1.97840 3.09090
H -0.27740 -0.55610 3.16740
C -1.93710 -0.49940 4.56750
H -1.37580 -0.95820 5.38820
H -1.91500 0.58640 4.70370
H -2.97730 -0.83320 4.63980
C -4.28540 0.05700 0.80630
C -6.09490 1.62480 0.80620
H -6.39870 2.34880 1.56320
H -5.53450 2.13390 0.01890
C -7.27860 0.84740 0.25530
H -7.80770 0.32690 1.05920
H -7.97520 1.54320 -0.22410
H -6.95360 0.11500 -0.48850
C -5.36940 -3.64350 2.12300
H -6.35440 -3.85890 1.70590
H -4.64200 -4.38200 1.77210
H -5.41480 -3.64610 3.21650
C -1.56230 -1.66270 -5.19290
H -2.58980 -1.94550 -4.94260
H -1.00640 -2.57400 -5.44150
H -1.59050 -1.02470 -6.08260
C 0.53260 -0.46670 -4.41870
H 0.47670 0.25110 -5.24280
H 1.14180 -1.31250 -4.75660
H 1.05180 0.01360 -3.58450
C -1.26700 -4.25760 0.83870
H -0.31010 -4.30270 1.36320
H -1.56820 -5.27750 0.57020
H -2.02330 -3.84890 1.51330
H -3.25120 -2.80690 -0.43000
H 0.97990 -3.65760 -0.61850
99
iTS1-major-Z_30-5
G = -2623.2935486
N -1.42020 0.72320 -0.70810
H -2.18020 0.91260 -1.35920
H -1.75830 0.94560 0.24570
C 0.88370 1.15940 -0.03860
H 0.57970 1.46970 0.96820
C -0.24270 1.57390 -1.01030
H 0.07470 1.32640 -2.02540
C 0.01940 -2.17680 -2.61070
C 0.20230 -3.09750 -1.52210
C -1.29570 -1.72070 -2.85130
C -2.21560 -3.12590 -1.03580
C -2.40260 -2.18690 -2.07240

C	1.23770	-1.70010	-3.37950
H	1.94960	-1.35340	-2.62130
N	0.98160	-0.32190	-0.10740
S	1.98950	-0.98510	0.99610
O	2.00800	-0.22570	2.28130
O	1.69990	-2.43930	1.08790
C	2.16040	1.89750	-0.41340
C	2.65660	2.91230	0.41100
C	2.82830	1.60850	-1.61080
C	3.80110	3.63070	0.04690
H	2.14680	3.14060	1.34350
C	3.97080	2.31950	-1.97830
H	2.46310	0.80760	-2.24410
C	4.46150	3.33620	-1.14940
H	4.17760	4.41370	0.69960
H	4.48410	2.07550	-2.90430
H	5.35440	3.88730	-1.43110
C	-0.55480	3.05610	-0.94070
C	-0.25340	3.88650	-2.02780
C	-1.12370	3.62040	0.21170
C	-0.50950	5.26050	-1.96850
H	0.19160	3.45710	-2.92180
C	-1.38460	4.99200	0.27120
H	-1.36170	2.99050	1.06430
C	-1.07670	5.81690	-0.81750
H	-0.26750	5.89240	-2.81850
H	-1.82570	5.41750	1.16840
H	-1.27810	6.88330	-0.76870
C	-0.87940	-3.57450	-0.76170
Ru	-0.85410	-1.31130	-0.67300
H	-1.38180	-1.13660	0.98130
C	3.66600	-0.86930	0.33750
C	4.02930	-1.68320	-0.73890
C	4.57440	0.04000	0.87760
C	5.30350	-1.56290	-1.29070
C	5.84950	0.14660	0.31860
C	6.23050	-0.63860	-0.77890
H	-1.48100	-0.96660	-3.60720
H	1.20380	-3.39130	-1.23430
H	4.28080	0.66810	1.70990
H	6.55260	0.86300	0.73530
H	5.58170	-2.19550	-2.12980
H	3.33070	-2.40810	-1.13960
C	7.58850	-0.47340	-1.41770
H	8.32590	-0.10270	-0.69950
H	7.53740	0.25060	-2.24090
H	7.95220	-1.41760	-1.83470
O	-4.27480	-0.07790	-0.17840
O	-1.82020	0.76150	2.02450
O	-4.98240	0.79260	1.78690
O	-5.21340	-2.23820	2.15010
N	-3.86150	-2.02770	2.43200
C	-3.41370	-0.96090	1.88750
C	-1.97190	-0.51350	2.10660
C	-1.18280	-1.28730	3.16830
H	-1.31060	-2.36250	3.01180
H	-0.13000	-1.04530	3.01740
C	-1.61160	-0.88790	4.58910

H	-1.00990	-1.42800	5.32790
H	-1.46560	0.18500	4.75030
H	-2.66490	-1.12230	4.77190
C	-4.26910	-0.04560	1.03960
C	-5.81770	1.76390	1.07910
H	-5.17040	2.34660	0.41770
H	-6.53750	1.21110	0.46910
C	-6.49130	2.62410	2.12840
H	-5.74930	3.15270	2.73430
H	-7.12560	3.36590	1.63300
H	-7.11930	2.01710	2.78750
C	-5.65700	-3.45360	2.77480
H	-6.71040	-3.54590	2.50570
H	-5.09450	-4.31010	2.39010
H	-5.55040	-3.38840	3.86220
C	0.96020	-0.53610	-4.33680
H	0.50180	0.31680	-3.82820
H	0.29820	-0.84060	-5.15540
H	1.90060	-0.19320	-4.77920
C	1.88510	-2.87890	-4.13540
H	2.80400	-2.54640	-4.62950
H	1.20410	-3.26620	-4.90210
H	2.14380	-3.70180	-3.46170
C	-3.37930	-3.66640	-0.25710
H	-3.72900	-4.59000	-0.73460
H	-4.20660	-2.95550	-0.23250
H	-3.09220	-3.90350	0.76910
H	-3.39150	-1.77990	-2.25100
H	-0.69810	-4.24600	0.07000

99
iTS1-major-Z_30-4
G = -2623.2906463

N	-1.02390	0.86790	-1.03740
H	-1.87310	1.04790	-1.57030
H	-1.24440	1.06820	-0.04430
C	1.36970	1.28320	-0.79180
H	1.23040	1.37580	0.29560
C	0.06710	1.77190	-1.47480
H	0.18720	1.62310	-2.55070
C	-1.09680	-1.67950	-3.24170
C	0.05060	-2.43710	-2.92550
C	-2.20260	-1.76570	-2.32470
C	-1.00740	-3.39820	-0.90960
C	-2.17090	-2.60680	-1.19330
C	-1.20070	-0.77220	-4.45150
H	-1.74880	0.12270	-4.12800
N	1.48280	-0.14870	-1.17360
S	2.86580	-0.95010	-0.84890
O	2.81920	-2.25030	-1.56240
O	4.06440	-0.11340	-1.12010
C	2.50100	2.20700	-1.21600
C	3.09880	3.05290	-0.27760
C	2.89290	2.29730	-2.55800
C	4.08320	3.96930	-0.66660
H	2.79250	2.99880	0.76410
C	3.87200	3.20860	-2.95260
H	2.44390	1.63620	-3.29310
C	4.47350	4.04980	-2.00610

H	4.54030	4.61790	0.07580
H	4.16900	3.26430	-3.99650
H	5.23660	4.76000	-2.31250
C	-0.25340	3.23230	-1.21520
C	-0.29670	4.13430	-2.28640
C	-0.49550	3.70630	0.08410
C	-0.57210	5.48860	-2.06950
H	-0.10490	3.77690	-3.29480
C	-0.77300	5.05840	0.30240
H	-0.46840	3.01960	0.92430
C	-0.81080	5.95460	-0.77260
H	-0.59930	6.17560	-2.91070
H	-0.95720	5.41290	1.31290
H	-1.02460	7.00570	-0.60050
C	0.09260	-3.29430	-1.78080
Ru	-0.44120	-1.16570	-1.11700
H	-0.55660	-0.92950	0.61390
C	2.96670	-1.34110	0.90780
C	3.39250	-0.35280	1.80130
C	2.62310	-2.61500	1.36620
C	3.47000	-0.65030	3.16330
C	2.71180	-2.89820	2.72970
C	3.13220	-1.92400	3.64890
H	-3.06880	-1.12910	-2.47520
H	0.95480	-2.33340	-3.51060
H	2.29330	-3.37110	0.66330
H	2.43740	-3.88790	3.08560
H	3.79210	0.12070	3.85810
H	3.66860	0.63170	1.43820
C	3.23020	-2.24530	5.12100
H	3.16660	-1.33990	5.73150
H	4.18620	-2.73310	5.34870
H	2.43260	-2.92810	5.43040
O	-3.75730	0.05130	-0.09520
O	-1.10730	0.91820	1.71910
O	-4.27560	0.59520	2.04050
O	-4.12870	-2.47150	2.01150
N	-2.79010	-2.11440	2.18570
C	-2.53370	-0.94870	1.72710
C	-1.12870	-0.36290	1.80890
C	-0.16370	-1.05150	2.77040
H	-0.17340	-2.13060	2.60340
H	0.83180	-0.68570	2.52240
C	-0.48940	-0.72520	4.23510
H	-0.45010	0.35460	4.41060
H	-1.48530	-1.08350	4.51450
H	0.24290	-1.20480	4.89260
C	-3.58650	-0.05450	1.10620
C	-5.30940	1.52320	1.57880
H	-4.83420	2.25850	0.92360
H	-6.03980	0.95370	0.99750
C	-5.92100	2.15840	2.81010
H	-5.16680	2.70780	3.38150
H	-6.70180	2.86110	2.50270
H	-6.37310	1.40080	3.45730
C	-4.36320	-3.77150	2.58040
H	-5.41670	-3.98110	2.38920
H	-3.73480	-4.52200	2.09160

H	-4.17110	-3.75890	3.65770
C	-2.04030	-1.46630	-5.54450
H	-3.02920	-1.74990	-5.16940
H	-1.53480	-2.37240	-5.89820
H	-2.17760	-0.79450	-6.39850
C	0.15860	-0.32120	-5.00020
H	0.78670	0.10160	-4.21070
H	0.01180	0.44150	-5.77110
H	0.70250	-1.15440	-5.45920
C	-0.96800	-4.29960	0.29180
H	-1.53910	-5.21150	0.07900
H	-1.42100	-3.81730	1.16000
H	0.05710	-4.58620	0.53670
H	-3.01400	-2.61980	-0.51330
H	1.01210	-3.81680	-1.55410

99
iTS1-major-Z_0-4
G = -2623.2884879

N	-1.01910	0.74330	-0.67210
H	-1.90690	1.10160	-1.01920
H	-1.04080	0.84810	0.35680
C	1.42520	0.88830	-0.66780
H	1.55230	1.31700	0.33370
C	0.12420	1.49170	-1.25100
H	0.10670	1.30110	-2.32630
C	-1.25690	-1.47840	-3.28270
C	-0.13800	-2.29270	-3.00320
C	-2.42430	-1.66680	-2.46620
C	-1.34120	-3.51180	-1.22080
C	-2.47540	-2.66620	-1.47170
C	-1.19580	-0.47070	-4.41570
H	-0.16750	-0.08980	-4.44760
N	1.21440	-0.57280	-0.55600
S	2.43220	-1.49710	-0.03060
O	1.86240	-2.74730	0.54360
O	3.50040	-1.73840	-1.04600
C	2.61710	1.32140	-1.50710
C	2.75590	0.88900	-2.83290
C	3.57790	2.18310	-0.96860
C	3.83340	1.31530	-3.60980
H	2.02680	0.20040	-3.24870
C	4.66150	2.61410	-1.74270
H	3.48320	2.51250	0.06300
C	4.79080	2.18200	-3.06560
H	3.93070	0.97090	-4.63580
H	5.40210	3.28180	-1.31110
H	5.63160	2.51350	-3.66870
C	0.03340	2.98750	-1.01830
C	-0.13110	3.50520	0.27620
C	0.12970	3.87280	-2.09910
C	-0.19890	4.88520	0.48290
H	-0.20740	2.83260	1.12680
C	0.06750	5.25560	-1.89420
H	0.25770	3.47860	-3.10390
C	-0.09750	5.76480	-0.60220
H	-0.32820	5.27370	1.48920
H	0.14590	5.93100	-2.74140
H	-0.14820	6.83790	-0.44070

C	-0.17560	-3.30000	-1.98660
Ru	-0.73270	-1.32560	-1.03340
H	-0.97580	-1.38640	0.65230
C	3.22070	-0.57420	1.30480
C	2.42910	-0.00530	2.30870
C	4.60800	-0.43370	1.32320
C	3.04670	0.69410	3.34450
C	5.21140	0.26690	2.37070
C	4.44370	0.83750	3.39690
H	-3.27990	-1.01230	-2.58440
H	0.79000	-2.12330	-3.54030
H	5.20430	-0.85520	0.52170
H	6.29240	0.37830	2.38310
H	2.43190	1.14710	4.11820
H	1.34600	-0.07080	2.26630
C	5.09530	1.57630	4.54050
H	4.50010	2.44380	4.84310
H	6.09980	1.91900	4.27600
H	5.18720	0.92320	5.41730
O	-3.80660	0.50040	0.26850
O	-1.00690	0.28210	2.11860
O	-4.18250	0.69000	2.49660
O	-4.79000	-2.25650	1.68700
N	-3.41400	-2.27900	1.91650
C	-2.87810	-1.12520	1.78220
C	-1.37630	-0.93400	1.96650
C	-0.65220	-2.04810	2.72970
H	-1.05590	-3.01830	2.43170
H	0.39660	-2.03330	2.43110
C	-0.79030	-1.84490	4.24600
H	-0.26860	-2.64400	4.78280
H	-0.35520	-0.88720	4.54850
H	-1.84080	-1.85710	4.55690
C	-3.67260	0.10770	1.41400
C	-4.98000	1.90460	2.30290
H	-4.91740	2.41000	3.26730
H	-4.49620	2.51640	1.53820
C	-6.41260	1.55540	1.93610
H	-6.86500	0.92130	2.70450
H	-6.99880	2.47700	1.85840
H	-6.45520	1.03550	0.97540
C	-5.32670	-3.58210	1.83550
H	-6.39320	-3.48370	1.62730
H	-4.86270	-4.26400	1.11600
H	-5.17380	-3.94630	2.85620
C	-2.14460	0.72450	-4.25260
H	-2.00690	1.23600	-3.29640
H	-3.19370	0.41830	-4.32620
H	-1.95780	1.45120	-5.04900
C	-1.46780	-1.19810	-5.75070
H	-2.48020	-1.61850	-5.76040
H	-0.75610	-2.01410	-5.91100
H	-1.38130	-0.49570	-6.58670
C	-1.40100	-4.56990	-0.15490
H	-1.79450	-5.49960	-0.58370
H	-2.06470	-4.26550	0.65780
H	-0.40750	-4.77250	0.25130
H	-3.36290	-2.76450	-0.85670

H	0.72180	-3.86380	-1.76060

99
iTS1-major-Z_0-3
G = -2623.2856606

N	-1.26460	0.73690	-0.83140
H	-2.04520	1.01710	-1.42250
H	-1.51700	0.98140	0.14210
C	1.13370	0.88660	-0.38680
H	0.94480	1.13980	0.66490
C	-0.03550	1.47510	-1.21650
H	0.15220	1.24680	-2.26840
C	-1.45750	-1.74790	-3.06600
C	-0.42830	-2.63120	-2.67910
C	-2.63330	-1.71950	-2.23540
C	-1.75770	-3.52060	-0.79060
C	-2.79180	-2.58490	-1.13120
C	-1.36800	-0.81940	-4.26210
H	-1.80380	0.13980	-3.95220
N	1.04230	-0.59350	-0.51310
S	2.34480	-1.54900	-0.53910
O	1.95940	-2.88680	-0.00690
O	3.03750	-1.61670	-1.86170
C	2.42070	1.58260	-0.80440
C	2.94830	1.44420	-2.09500
C	3.04790	2.45780	0.09100
C	4.08850	2.15460	-2.47700
H	2.48480	0.75820	-2.79390
C	4.18580	3.17470	-0.28720
H	2.64880	2.56910	1.09520
C	4.71240	3.02350	-1.57400
H	4.49030	2.02940	-3.47880
H	4.66380	3.84260	0.42420
H	5.60070	3.57450	-1.87020
C	-0.18820	2.97580	-1.05780
C	0.03950	3.81920	-2.15330
C	-0.54010	3.54340	0.17740
C	-0.07680	5.20700	-2.02180
H	0.31650	3.38810	-3.11190
C	-0.65980	4.92940	0.30960
H	-0.72240	2.90420	1.03590
C	-0.42730	5.76610	-0.78850
H	0.10480	5.84780	-2.88010
H	-0.93420	5.35600	1.27050
H	-0.51990	6.84350	-0.68380
C	-0.57940	-3.51380	-1.56020
Ru	-0.93830	-1.35340	-0.86450
H	-1.14700	-1.12830	0.82740
C	3.52320	-0.84640	0.62730
C	4.82600	-0.55120	0.22930
C	3.10250	-0.61140	1.93830
C	5.71380	-0.00710	1.15970
C	3.99710	-0.06210	2.85460
C	5.31560	0.25010	2.48040
H	-3.40490	-0.98210	-2.43420
H	0.52220	-2.62290	-3.19860
H	2.08450	-0.84000	2.23040
H	3.66600	0.13470	3.87110
H	6.72590	0.23730	0.84810

H	5.12740	-0.71690	-0.79850
C	6.28300	0.82980	3.48450
H	7.08100	1.39270	2.99130
H	6.75490	0.03140	4.07080
H	5.77330	1.49410	4.18930
O	-4.16350	0.00310	0.08000
O	-1.49210	0.82970	1.91340
O	-4.56170	0.87040	2.13590
O	-4.77770	-2.25550	2.37980
N	-3.41040	-2.02090	2.54360
C	-3.04330	-0.92070	2.00580
C	-1.60420	-0.43380	2.07520
C	-0.64120	-1.11220	3.05600
H	0.23660	-0.45960	3.07150
H	-1.11810	-1.01780	4.04160
C	-0.22730	-2.56370	2.80980
H	0.49690	-2.87050	3.57250
H	-1.08290	-3.23820	2.86370
H	0.24250	-2.67400	1.82840
C	-3.98880	0.02070	1.28450
C	-5.38140	1.94170	1.56270
H	-6.01520	1.51480	0.78210
H	-6.00250	2.26690	2.39800
C	-4.50060	3.06390	1.03610
H	-3.86350	2.71410	0.21980
H	-5.13490	3.87360	0.66040
H	-3.86390	3.46190	1.83150
C	-5.13520	-3.50170	3.00100
H	-6.20680	-3.61060	2.82710
H	-4.59280	-4.33120	2.53680
H	-4.92930	-3.46860	4.07540
C	-2.23640	-1.37640	-5.41000
H	-3.27430	-1.52180	-5.09290
H	-1.84460	-2.34070	-5.75370
H	-2.23150	-0.68250	-6.25740
C	0.06680	-0.55830	-4.73590
H	0.06820	0.22520	-5.49960
H	0.51130	-1.45570	-5.18070
H	0.70930	-0.23530	-3.91220
C	-1.93970	-4.47260	0.35740
H	-0.97800	-4.80760	0.75150
H	-2.49640	-5.35160	0.01030
H	-2.51100	-4.00920	1.16380
H	-3.67940	-2.50730	-0.51520
H	0.25390	-4.13980	-1.26950

99
iTS1-major-Z_30-2
G = -2623.2844776

N	-1.22180	0.70710	-0.70490
H	-2.04510	1.00630	-1.22460
H	-1.41420	0.90190	0.29430
C	1.19790	0.89610	-0.37180
H	1.11450	1.22710	0.67200
C	-0.02230	1.46240	-1.13980
H	0.12300	1.24010	-2.19950
C	-1.13410	-1.69580	-3.11790
C	-0.16770	-2.59840	-2.61140
C	-2.39960	-1.66940	-2.43960

C	-1.73380	-3.51900	-0.94100
C	-2.70430	-2.56260	-1.38460
C	-0.79120	-0.80910	-4.30390
H	0.24360	-0.47390	-4.15630
N	1.07250	-0.58470	-0.39770
S	2.36270	-1.55460	-0.31590
O	1.94490	-2.84280	0.30610
O	3.09000	-1.73610	-1.60890
C	2.45540	1.52170	-0.95840
C	2.85670	1.24730	-2.27260
C	3.18320	2.45140	-0.20670
C	3.97350	1.88190	-2.82040
H	2.30800	0.51700	-2.85750
C	4.29830	3.09370	-0.75180
H	2.88250	2.66360	0.81560
C	4.69890	2.80820	-2.06100
H	4.27860	1.65360	-3.83800
H	4.85670	3.80760	-0.15260
H	5.56910	3.30070	-2.48620
C	-0.18590	2.95990	-0.96670
C	-0.52380	3.50990	0.27990
C	0.01180	3.81590	-2.05790
C	-0.65920	4.89270	0.42880
H	-0.68680	2.85990	1.13500
C	-0.12000	5.20060	-1.91010
H	0.27570	3.39700	-3.02550
C	-0.45600	5.74260	-0.66510
H	-0.92400	5.30610	1.39800
H	0.03820	5.85240	-2.76480
H	-0.56090	6.81760	-0.54800
C	-0.46320	-3.51450	-1.55430
Ru	-0.87200	-1.36980	-0.85780
H	-1.14580	-1.26690	0.83820
C	3.51300	-0.75930	0.81800
C	4.84030	-0.55110	0.44410
C	3.05060	-0.35960	2.07420
C	5.71150	0.06610	1.34350
C	3.93000	0.26150	2.95960
C	5.27280	0.48400	2.60970
H	-3.14080	-0.92510	-2.70680
H	0.84240	-2.57180	-3.00860
H	2.01250	-0.51360	2.34660
H	3.56720	0.58510	3.93170
H	6.74350	0.23970	1.04990
H	5.17520	-0.84470	-0.54430
C	6.22540	1.13710	3.58230
H	5.70290	1.83310	4.24550
H	7.01730	1.68180	3.05970
H	6.70780	0.38080	4.21420
O	-4.08340	0.03350	-0.03440
O	-1.46010	0.64720	2.02890
O	-4.53910	0.84770	2.03120
O	-4.93430	-2.26930	2.09580
N	-3.57230	-2.11830	2.36630
C	-3.11150	-1.01600	1.90980
C	-1.65780	-0.61350	2.10690
C	-0.80340	-1.40430	3.10410
H	0.12040	-0.82650	3.19750

H -1.32870 -1.31150 4.06520
C -0.48310 -2.87170 2.81850
H 0.16290 -3.26290 3.61240
H -1.38700 -3.48070 2.78550
H 0.04740 -2.97070 1.86810
C -3.96720 0.00020 1.17720
C -5.29020 1.97080 1.46260
H -5.89240 1.60050 0.62990
H -5.94630 2.28110 2.27660
C -4.34490 3.08290 1.03700
H -3.67740 2.74960 0.23850
H -4.92980 3.93210 0.66850
H -3.73790 3.42020 1.88200
C -5.40270 -3.51680 2.63460
H -6.46290 -3.55790 2.37990
H -4.87280 -4.35680 2.17490
H -5.27560 -3.53800 3.72150
C -1.68190 0.43260 -4.44570
H -1.69600 1.04030 -3.53690
H -2.71480 0.15930 -4.68820
H -1.30730 1.06100 -5.25940
C -0.83170 -1.65130 -5.59740
H -0.53340 -1.04010 -6.45600
H -1.84530 -2.02680 -5.78010
H -0.15480 -2.50920 -5.53440
C -2.07610 -4.49940 0.14460
H -2.62660 -5.33970 -0.29570
H -2.71270 -4.03920 0.90240
H -1.17780 -4.89030 0.62620
H -3.66200 -2.48900 -0.88360
H 0.32000 -4.15980 -1.17780
99
iTS1-major-Z_0-2
N -1.21880 0.82650 -0.80780
H -1.89040 1.00080 -1.55340
H -1.67050 1.10620 0.08190
C 1.02570 1.22440 0.07480
H 0.65020 1.60240 1.03350
C 0.01150 1.63070 -1.01710
H 0.41630 1.33490 -1.98740
C -0.87620 -1.83820 -2.81240
C 0.24420 -2.47750 -2.24530
C -2.14050 -2.00220 -2.14390
C -1.12120 -3.46250 -0.42610
C -2.26640 -2.80020 -0.99070
C -0.81220 -0.98350 -4.06400
H -1.42240 -0.09170 -3.86860
N 1.06360 -0.26050 0.09160
S 1.97860 -0.91150 1.27390
O 2.03350 -0.05050 2.49140
O 1.56790 -2.32390 1.48150
C 2.36680 1.88280 -0.21560
C 2.85150 2.90040 0.61220
C 3.11610 1.50710 -1.33900
C 4.06330 3.53820 0.32310
H 2.27940 3.19380 1.48850
C 4.32500 2.13860 -1.63220
H 2.76210 0.69730 -1.96900

C	4.80280	3.15980	-0.80130
H	4.42890	4.32510	0.97720
H	4.89970	1.82830	-2.50040
H	5.74660	3.64930	-1.02510
C	-0.25460	3.12350	-1.03220
C	0.16580	3.89900	-2.12040
C	-0.89930	3.75190	0.04490
C	-0.04680	5.28170	-2.13560
H	0.66890	3.41950	-2.95630
C	-1.11670	5.13230	0.02950
H	-1.23120	3.16450	0.89670
C	-0.68920	5.90200	-1.05930
H	0.28790	5.87040	-2.98510
H	-1.61700	5.60780	0.86860
H	-0.85670	6.97530	-1.06810
C	0.12200	-3.27280	-1.06020
Ru	-0.73030	-1.22210	-0.59770
H	-1.33270	-0.90590	0.98110
C	3.66370	-0.99150	0.63700
C	3.91080	-1.76690	-0.49960
C	4.69010	-0.26860	1.24190
C	5.19750	-1.81150	-1.03190
C	5.97580	-0.32230	0.69730
C	6.25070	-1.08860	-0.44420
H	-3.00750	-1.45660	-2.50130
H	1.22980	-2.32410	-2.66710
H	4.48060	0.34470	2.11060
H	6.77260	0.25380	1.16070
H	5.38590	-2.40720	-1.92140
H	3.10400	-2.31500	-0.97430
C	7.64610	-1.15250	-1.01840
H	8.20160	-0.23150	-0.81720
H	7.62480	-1.31530	-2.10040
H	8.21020	-1.98140	-0.57280
O	-4.16810	0.03060	-0.50080
O	-2.02260	1.01170	1.84390
O	-5.19430	0.69560	1.40570
O	-5.04990	-2.39390	1.55450
N	-3.79440	-2.01400	2.03750
C	-3.44410	-0.85800	1.61890
C	-2.09150	-0.26190	1.99390
C	-1.36460	-0.91200	3.17440
H	-1.36230	-1.99910	3.05700
H	-0.32900	-0.56750	3.13670
C	-2.01060	-0.51180	4.51020
H	-1.45830	-0.95830	5.34370
H	-1.99950	0.57540	4.63660
H	-3.04900	-0.85360	4.56970
C	-4.30310	-0.00580	0.70920
C	-6.06810	1.59580	0.65110
H	-5.43490	2.29050	0.09240
H	-6.64200	0.99390	-0.05900
C	-6.95440	2.30410	1.65460
H	-6.35560	2.88510	2.36250
H	-7.62350	2.98880	1.12400
H	-7.56470	1.58710	2.21200
C	-5.38710	-3.69850	2.05540
H	-6.36490	-3.92530	1.62760

H	-4.65050	-4.43720	1.72450
H	-5.44770	-3.68690	3.14820
C	-1.46440	-1.74680	-5.23680
H	-2.49290	-2.04000	-5.00320
H	-0.89340	-2.65330	-5.46860
H	-1.48370	-1.11550	-6.13130
C	0.60220	-0.52160	-4.43280
H	0.55340	0.18920	-5.26330
H	1.22710	-1.36280	-4.75290
H	1.10030	-0.02910	-3.59290
C	-1.25120	-4.29480	0.81870
H	-0.30330	-4.32540	1.36030
H	-1.53670	-5.32000	0.55300
H	-2.02350	-3.88900	1.47680
H	-3.22700	-2.87160	-0.49360
H	1.01440	-3.68440	-0.60490

99
iTS-2-major-E_0-3
G = -2623.2901922

N	-1.30300	0.63400	-0.50380
H	-1.99850	0.84790	-1.21510
H	-1.73570	0.86500	0.40960
C	0.89490	1.05240	0.44640
H	0.48380	1.43920	1.38710
C	-0.07880	1.45100	-0.68410
H	0.36490	1.15850	-1.64010
C	-0.77510	-1.89150	-2.65770
C	0.37530	-2.45220	-2.06200
C	-2.04810	-2.21320	-2.07370
C	-0.97200	-3.69110	-0.39490
C	-2.14360	-3.08640	-0.96820
C	-0.62780	-1.03070	-3.89880
H	0.35190	-0.54170	-3.83190
N	0.92710	-0.43220	0.47600
S	1.71550	-1.07620	1.74940
O	1.57820	-0.24890	2.98430
O	1.34320	-2.50930	1.86610
C	2.24680	1.70550	0.20270
C	3.05020	1.29650	-0.87050
C	2.68660	2.75060	1.02130
C	4.27190	1.92220	-1.12100
H	2.72440	0.46790	-1.49230
C	3.90950	3.38420	0.77240
H	2.07070	3.06930	1.85840
C	4.70530	2.97140	-0.30010
H	4.89100	1.58710	-1.94860
H	4.24050	4.19290	1.41830
H	5.65880	3.45580	-0.49150
C	-0.37030	2.93830	-0.70750
C	0.08220	3.72650	-1.77290
C	-1.08510	3.54640	0.33520
C	-0.16750	5.10240	-1.79770
H	0.63660	3.26180	-2.58430
C	-1.34040	4.91930	0.30990
H	-1.44860	2.94930	1.16700
C	-0.88050	5.70260	-0.75520
H	0.19160	5.70150	-2.62980
H	-1.89970	5.37740	1.12090

H	-1.07870	6.77060	-0.77280
C	0.27510	-3.33760	-0.94380
Ru	-0.78430	-1.41020	-0.39780
H	-1.54620	-1.24940	1.13480
C	3.47160	-1.06360	1.33410
C	3.91780	-1.86380	0.27870
C	4.36010	-0.24630	2.03050
C	5.26390	-1.83580	-0.08040
C	5.70660	-0.22720	1.65920
C	6.17860	-1.01090	0.59670
H	-2.95310	-1.75240	-2.45150
H	1.35760	-2.17320	-2.42990
H	3.99920	0.37720	2.84030
H	6.39660	0.41680	2.19840
H	5.60890	-2.45780	-0.90240
H	3.22270	-2.49490	-0.26410
C	7.62350	-0.94080	0.16400
H	8.28260	-0.71140	1.00680
H	7.76010	-0.14970	-0.58440
H	7.95190	-1.88110	-0.28900
O	-4.25170	0.32620	-0.39850
O	-2.17770	0.67060	2.10470
O	-5.19240	0.87390	1.58780
O	-3.80790	-3.14140	2.18560
N	-4.42750	-2.06920	1.56330
C	-3.74010	-0.98860	1.55130
C	-2.37440	-0.59640	2.13500
C	-1.82480	-1.38020	3.33030
H	-1.73110	-2.43850	3.08710
H	-0.82420	-0.98010	3.51260
C	-2.71910	-1.19100	4.56540
H	-2.28820	-1.71480	5.42500
H	-2.81510	-0.13150	4.82450
H	-3.72110	-1.59720	4.39120
C	-4.41660	0.13730	0.79500
C	-5.81450	2.06550	1.00330
H	-6.21870	1.80030	0.02370
H	-6.63440	2.28860	1.68690
C	-4.81490	3.20740	0.91870
H	-3.99000	2.96030	0.24670
H	-5.31950	4.10030	0.53510
H	-4.40510	3.43830	1.90640
C	-4.68590	-4.27990	2.17200
H	-4.92420	-4.56800	1.14320
H	-4.12650	-5.07660	2.66460
H	-5.60300	-4.06310	2.72820
C	-1.69220	0.06110	-4.06310
H	-1.69570	0.76250	-3.22560
H	-2.69770	-0.35980	-4.16470
H	-1.48470	0.63740	-4.97010
C	-0.61400	-1.95770	-5.13450
H	-0.44570	-1.37050	-6.04340
H	-1.57370	-2.47770	-5.23610
H	0.17720	-2.71040	-5.05850
C	-1.07340	-4.64020	0.76600
H	-1.26720	-5.65530	0.39830
H	-1.89260	-4.35520	1.42780
H	-0.14260	-4.64640	1.33670

H	-3.11420	-3.27630	-0.52310
H	1.17780	-3.69940	-0.46690

99
iTS-2-major-E_0-7
G = -2623.2887478

N	-1.24360	0.72740	-0.47180
H	-1.93710	0.95590	-1.18010
H	-1.67230	0.96490	0.44030
C	0.97680	1.08680	0.45360
H	0.58720	1.47470	1.40320
C	-0.00320	1.51640	-0.66020
H	0.41940	1.22030	-1.62450
C	-0.82250	-1.79900	-2.64430
C	0.32870	-2.38850	-2.07950
C	-2.08860	-2.09610	-2.03270
C	-1.00720	-3.60490	-0.38510
C	-2.17810	-2.97220	-0.92860
C	-0.68050	-0.92510	-3.87730
H	0.28840	-0.41690	-3.79510
N	0.97780	-0.39800	0.46950
S	1.78230	-1.07110	1.71680
O	1.69850	-0.24980	2.96060
O	1.37800	-2.49530	1.83320
C	2.33950	1.71200	0.19660
C	2.82670	2.72130	1.03270
C	3.10690	1.31150	-0.90590
C	4.06070	3.32800	0.77210
H	2.23990	3.03190	1.89340
C	4.33840	1.91200	-1.16950
H	2.74460	0.50970	-1.54260
C	4.81930	2.92540	-0.33060
H	4.42950	4.10800	1.43260
H	4.92760	1.58510	-2.02180
H	5.78090	3.38960	-0.53150
C	-0.25260	3.01150	-0.66840
C	0.23060	3.79710	-1.72220
C	-0.94760	3.63130	0.38100
C	0.03130	5.18130	-1.72930
H	0.77110	3.32370	-2.53790
C	-1.15210	5.01320	0.37360
H	-1.33080	3.03720	1.20660
C	-0.66130	5.79340	-0.68020
H	0.41450	5.77790	-2.55250
H	-1.69290	5.48180	1.19120
H	-0.81960	6.86810	-0.68330
C	0.23420	-3.27550	-0.96190
Ru	-0.77140	-1.32940	-0.38120
H	-1.50080	-1.15240	1.15490
C	3.52810	-1.10220	1.25950
C	4.45300	-0.30290	1.92960
C	3.92850	-1.91510	0.19510
C	5.79000	-0.31880	1.52500
C	5.26610	-1.92300	-0.19610
C	6.21810	-1.12390	0.45990
H	-2.99140	-1.61280	-2.38620
H	1.30800	-2.13020	-2.46980
H	3.20340	-2.52520	-0.33210
H	5.57390	-2.55120	-1.02800

H	6.50660	0.31740	2.03800
H	4.12600	0.33810	2.73990
C	7.66860	-1.14750	0.04020
H	8.15180	-0.18320	0.22460
H	7.77250	-1.39030	-1.02170
H	8.22290	-1.90640	0.60660
O	-4.15930	0.47120	-0.28950
O	-2.13890	0.78230	2.14350
O	-5.31260	0.82420	1.62810
O	-3.74680	-3.03190	2.33200
N	-4.38190	-1.97570	1.70200
C	-3.69660	-0.89490	1.64010
C	-2.32640	-0.48220	2.19000
C	-1.72850	-1.25590	3.36700
H	-1.63800	-2.31510	3.12720
H	-0.72430	-0.84930	3.51050
C	-2.57770	-1.06310	4.63350
H	-3.58350	-1.47510	4.49940
H	-2.11200	-1.58020	5.47900
H	-2.66850	-0.00270	4.89010
C	-4.40480	0.20500	0.87440
C	-6.06850	1.91910	1.01460
H	-6.38720	2.52190	1.86600
H	-5.38730	2.50110	0.38980
C	-7.25040	1.38190	0.22510
H	-7.90480	0.78420	0.86680
H	-7.82950	2.22170	-0.17320
H	-6.91320	0.76570	-0.61270
C	-4.62360	-4.17130	2.36380
H	-4.88230	-4.48310	1.34690
H	-4.05270	-4.95550	2.86310
H	-5.52900	-3.94160	2.93380
C	-1.76630	0.14510	-4.04140
H	-1.80990	0.82420	-3.18660
H	-2.75800	-0.29830	-4.17870
H	-1.55110	0.74910	-4.92820
C	-0.63440	-1.83660	-5.12340
H	-0.47380	-1.23470	-6.02410
H	-1.58010	-2.37880	-5.23840
H	0.17460	-2.57030	-5.05020
C	-1.10240	-4.55860	0.77270
H	-1.89340	-4.25430	1.45980
H	-0.15640	-4.59480	1.31650
H	-1.33590	-5.56550	0.40550
H	-3.14270	-3.14430	-0.46330
H	1.13940	-3.65860	-0.50680

99
iTS-2-major-E_0-6
G = -2623.2864021

N	-0.99190	0.86010	-0.70550
H	-1.78040	1.14650	-1.28130
H	-1.25730	1.05690	0.28220
C	1.42070	1.03820	-0.33050
H	1.25330	1.16390	0.74960
C	0.20650	1.65290	-1.06790
H	0.37550	1.50230	-2.13710
C	-1.94460	-1.47940	-2.65880
C	-0.65660	-1.99050	-2.96920

C	-2.61520	-1.99600	-1.50640
C	-0.71270	-3.51540	-1.00460
C	-1.99780	-2.97310	-0.69260
C	-2.65040	-0.43630	-3.50730
H	-3.19590	0.22110	-2.81880
N	1.40980	-0.40750	-0.68630
S	2.68890	-1.31310	-0.21570
O	2.49910	-2.69020	-0.73150
O	3.97470	-0.66190	-0.58510
C	2.64820	1.84470	-0.73410
C	3.24700	2.71370	0.18280
C	3.11980	1.82060	-2.05380
C	4.31190	3.53740	-0.20300
H	2.87260	2.76030	1.20230
C	4.17840	2.64020	-2.44510
H	2.66490	1.14290	-2.77010
C	4.78160	3.50240	-1.51860
H	4.76740	4.20550	0.52280
H	4.53580	2.60840	-3.47090
H	5.60650	4.14070	-1.82270
C	0.01690	3.13600	-0.80890
C	-0.28200	3.61860	0.47500
C	0.15440	4.04800	-1.86350
C	-0.43650	4.98990	0.69540
H	-0.39530	2.92370	1.30130
C	0.00320	5.42120	-1.64410
H	0.39020	3.68240	-2.85960
C	-0.29250	5.89600	-0.36230
H	-0.66680	5.35120	1.69380
H	0.11620	6.11600	-2.47160
H	-0.41090	6.96180	-0.18840
C	-0.05700	-2.98310	-2.13990
Ru	-0.61740	-1.21420	-0.81400
H	-0.78640	-1.01710	0.92360
C	2.75720	-1.46020	1.58130
C	2.39070	-2.65720	2.19910
C	3.19790	-0.37220	2.34200
C	2.46280	-2.75960	3.58940
C	3.24910	-0.48510	3.73190
C	2.88260	-1.67750	4.37790
H	-3.57560	-1.58770	-1.21520
H	-0.09600	-1.59870	-3.80850
H	3.50990	0.54610	1.85770
H	3.57920	0.36570	4.32230
H	2.17000	-3.69010	4.06890
H	2.04860	-3.49270	1.60010
C	2.95950	-1.79870	5.88100
H	2.20180	-2.49000	6.26220
H	2.81850	-0.82810	6.36590
H	3.94040	-2.18230	6.18870
O	-4.71370	0.42270	2.42210
O	-1.31520	0.87110	1.99280
O	-3.72880	0.45800	0.38140
O	-2.34620	-3.08390	2.60180
N	-3.28160	-2.11550	2.27910
C	-2.78510	-0.96430	2.00640
C	-1.35300	-0.41250	2.08630
C	-0.40290	-1.05690	3.09920

H	-0.25580	-2.11080	2.87840
H	0.55120	-0.54440	2.97080
C	-0.90600	-0.87780	4.53800
H	-0.17590	-1.29750	5.23770
H	-1.04180	0.18110	4.78100
H	-1.85940	-1.39380	4.69360
C	-3.85140	0.04980	1.64560
C	-4.67270	1.45760	-0.11680
H	-4.94910	2.12060	0.70520
H	-4.09990	2.01940	-0.85670
C	-5.88030	0.77520	-0.73600
H	-6.42500	0.19340	0.01310
H	-6.55610	1.53320	-1.14560
H	-5.57420	0.10980	-1.54890
C	-3.01360	-4.31360	2.93660
H	-3.64830	-4.17720	3.81730
H	-3.60990	-4.67020	2.09070
H	-2.21150	-5.02030	3.15450
C	-3.70450	-1.14550	-4.38580
H	-4.41350	-1.71540	-3.77710
H	-3.21990	-1.83400	-5.08710
H	-4.26640	-0.40470	-4.96390
C	-1.71520	0.42750	-4.36250
H	-2.29030	1.22490	-4.84260
H	-1.23990	-0.16090	-5.15460
H	-0.92590	0.89180	-3.76490
C	-0.10050	-4.59520	-0.15380
H	-0.45020	-5.57930	-0.48870
H	-0.39030	-4.47320	0.89350
H	0.98760	-4.56800	-0.22800
H	-2.50130	-3.30460	0.20590
H	0.95690	-3.29580	-2.35160

99
iTS-2-major-E_330
G = -2623.2854668

N	-1.11590	1.01220	-0.72570
H	-1.88010	1.29210	-1.33780
H	-1.45540	1.15460	0.25360
C	1.28210	1.24660	-0.25280
H	1.07640	1.28490	0.82560
C	0.06800	1.86930	-0.98830
H	0.27420	1.79400	-2.05960
C	-1.09470	-1.39710	-3.11040
C	-0.05620	-2.26080	-2.68910
C	-2.30250	-1.42250	-2.33660
C	-1.43580	-3.23500	-0.89580
C	-2.47670	-2.31920	-1.25830
C	-0.90390	-0.52510	-4.33890
H	0.15720	-0.24800	-4.36690
N	1.33060	-0.16140	-0.72170
S	2.64720	-1.07930	-0.41460
O	2.58250	-2.27110	-1.29730
O	3.90100	-0.28570	-0.49390
C	2.49790	2.11510	-0.53600
C	3.09750	2.83210	0.50280
C	2.97400	2.28350	-1.84280
C	4.16480	3.70100	0.24680
H	2.73020	2.70980	1.51870

C	4.03660	3.14820	-2.10470
H	2.52330	1.71980	-2.65470
C	4.63770	3.86200	-1.05860
H	4.62270	4.24990	1.06530
H	4.39880	3.26690	-3.12240
H	5.46500	4.53640	-1.26180
C	-0.17960	3.32470	-0.63890
C	-0.49220	3.71690	0.67230
C	-0.07990	4.30390	-1.63620
C	-0.69630	5.06580	0.97570
H	-0.58530	2.96740	1.45210
C	-0.28260	5.65470	-1.33380
H	0.16670	4.00920	-2.65290
C	-0.59040	6.03940	-0.02490
H	-0.93640	5.35740	1.99460
H	-0.19890	6.40220	-2.11770
H	-0.74740	7.08750	0.21400
C	-0.22210	-3.17930	-1.60950
Ru	-0.64880	-1.03640	-0.89450
H	-0.86490	-0.87880	0.86170
C	2.61460	-1.71880	1.27130
C	3.10370	-0.92760	2.31460
C	2.09850	-2.99120	1.52700
C	3.05860	-1.41530	3.62130
C	2.06570	-3.46740	2.83820
C	2.53510	-2.68670	3.90590
H	-3.09590	-0.71820	-2.55450
H	0.91500	-2.19840	-3.16530
H	1.73480	-3.60190	0.71010
H	1.65760	-4.45530	3.03370
H	3.42400	-0.79260	4.43370
H	3.50910	0.05770	2.10880
C	2.48700	-3.20770	5.32220
H	2.40190	-2.38950	6.04350
H	3.40020	-3.76650	5.56250
H	1.63980	-3.88560	5.46510
O	-4.73000	-0.27090	2.88230
O	-1.73470	0.95000	1.87540
O	-4.11840	-0.35650	0.70890
O	-1.48010	-2.96020	3.13110
N	-2.68630	-2.36340	2.81910
C	-2.58350	-1.19390	2.30260
C	-1.38180	-0.27180	2.08190
C	-0.14190	-0.47820	2.95270
H	0.27100	-1.47190	2.81590
H	0.59200	0.24820	2.59960
C	-0.44750	-0.22890	4.43610
H	0.47170	-0.32870	5.02260
H	-0.85130	0.77600	4.59600
H	-1.16920	-0.95840	4.81780
C	-3.92580	-0.55920	2.01110
C	-5.34500	0.34140	0.33180
H	-6.19660	-0.24120	0.69350
H	-5.34670	1.31270	0.83480
C	-5.34680	0.47920	-1.17620
H	-5.34250	-0.50200	-1.66000
H	-6.25220	1.00980	-1.48700
H	-4.48010	1.05140	-1.51950

C	-1.71320	-4.21120	3.79650
H	-2.25940	-4.05230	4.73140
H	-2.26510	-4.89770	3.14690
H	-0.71970	-4.60950	4.00580
C	-1.73220	0.76690	-4.34300
H	-1.51750	1.39360	-3.47380
H	-2.80780	0.56170	-4.36380
H	-1.49300	1.35160	-5.23650
C	-1.21050	-1.36800	-5.59640
H	-1.02320	-0.77940	-6.50080
H	-2.26160	-1.68000	-5.60280
H	-0.58660	-2.26670	-5.63490
C	-1.64600	-4.24140	0.19910
H	-1.92790	-5.19950	-0.25410
H	-2.44920	-3.93490	0.86870
H	-0.73720	-4.39480	0.78400
H	-3.39170	-2.28540	-0.67980
H	0.61160	-3.80480	-1.32020

99

iTS-2-major-E_0-4

G = -2623.2853943

N	-1.02390	0.76260	-0.65360
H	-1.85860	1.02180	-1.17190
H	-1.22210	0.96560	0.34900
C	1.39490	1.00560	-0.41760
H	1.28600	1.19400	0.66070
C	0.13140	1.56610	-1.11310
H	0.23570	1.38460	-2.18680
C	-1.71330	-1.61620	-2.80800
C	-0.44290	-2.23480	-2.87060
C	-2.56780	-1.96850	-1.71100
C	-0.88520	-3.58420	-0.83970
C	-2.15410	-2.91830	-0.74820
C	-2.13820	-0.68680	-3.93330
H	-1.22940	-0.19530	-4.30100
N	1.39140	-0.45860	-0.68510
S	2.68400	-1.31640	-0.16820
O	2.52790	-2.71930	-0.62290
O	3.95700	-0.65130	-0.55810
C	2.58760	1.79740	-0.93640
C	2.99510	1.68180	-2.27220
C	3.21900	2.73470	-0.11350
C	4.02390	2.48000	-2.77260
H	2.51410	0.94920	-2.91380
C	4.25410	3.53720	-0.60900
H	2.89560	2.85030	0.91790
C	4.66020	3.41170	-1.94040
H	4.33220	2.37730	-3.80960
H	4.73660	4.25930	0.04400
H	5.46190	4.03350	-2.32900
C	-0.08470	3.05050	-0.88040
C	-0.29600	3.55780	0.41140
C	-0.08640	3.93440	-1.96680
C	-0.50150	4.92530	0.60860
H	-0.31200	2.88430	1.26300
C	-0.29110	5.30450	-1.77150
H	0.07810	3.54960	-2.97000
C	-0.49890	5.80360	-0.48170

H	-0.66600	5.30490	1.61320
H	-0.28880	5.97780	-2.62410
H	-0.65950	6.86690	-0.32690
C	-0.04430	-3.20840	-1.90510
Ru	-0.62680	-1.29580	-0.79060
H	-0.80340	-1.11470	0.94860
C	2.74510	-1.38890	1.63380
C	3.12230	-0.25220	2.35640
C	2.44360	-2.58240	2.29380
C	3.17190	-0.31260	3.75020
C	2.51590	-2.63160	3.68640
C	2.87000	-1.50000	4.43700
H	-3.52110	-1.47270	-1.57470
H	0.25250	-1.94860	-3.65270
H	2.15210	-3.45650	1.72280
H	2.27380	-3.55940	4.19830
H	3.45190	0.57560	4.31050
H	3.39160	0.66340	1.84180
C	2.94290	-1.56610	5.94370
H	3.91130	-1.96730	6.26760
H	2.16530	-2.22160	6.34810
H	2.82810	-0.57500	6.39210
O	-4.59870	0.57170	2.51890
O	-1.29100	0.78860	2.03720
O	-3.80060	0.41960	0.40370
O	-2.48560	-3.13000	2.59210
N	-3.37480	-2.11610	2.27810
C	-2.82560	-0.98480	2.03020
C	-1.37640	-0.49190	2.12940
C	-0.45890	-1.17860	3.14240
H	-0.33120	-2.23180	2.90210
H	0.50600	-0.68280	3.03830
C	-0.97900	-1.01450	4.57730
H	-0.26610	-1.45840	5.27970
H	-1.09830	0.04270	4.83580
H	-1.94340	-1.51530	4.71160
C	-3.83860	0.09150	1.69670
C	-4.69120	1.49130	-0.04940
H	-4.78740	1.30770	-1.11990
H	-5.66300	1.36000	0.43080
C	-4.09100	2.85740	0.24020
H	-3.11910	2.97590	-0.24600
H	-4.76370	3.63160	-0.14310
H	-3.96420	3.00950	1.31510
C	-3.21150	-4.33220	2.90360
H	-2.44580	-5.07690	3.12490
H	-3.85180	-4.17690	3.77700
H	-3.81030	-4.65190	2.04490
C	-3.14850	0.40270	-3.55160
H	-2.74870	1.10110	-2.81250
H	-4.07990	-0.02020	-3.16220
H	-3.40090	0.98950	-4.44010
C	-2.70390	-1.56140	-5.07640
H	-2.95860	-0.93370	-5.93660
H	-3.61280	-2.07980	-4.74950
H	-1.97770	-2.31320	-5.40070
C	-0.46720	-4.61110	0.17790
H	0.61980	-4.71750	0.19050

H	-0.90820	-5.58490	-0.06720
H	-0.80830	-4.32550	1.17520
H	-2.80520	-3.13270	0.08960
H	0.95680	-3.61310	-1.95530

99
iTS-2-major-E_0-5
G = -2623.2853616

N	-1.04620	0.78480	-0.79530
H	-1.85500	1.00190	-1.37470
H	-1.32770	0.97050	0.18650
C	1.33910	1.11650	-0.39630
H	1.13680	1.18790	0.68300
C	0.09620	1.66150	-1.14480
H	0.27820	1.53100	-2.21420
C	-1.15090	-1.67780	-3.03790
C	-0.01910	-2.46260	-2.73560
C	-2.27530	-1.78670	-2.14460
C	-1.12610	-3.50090	-0.78390
C	-2.26960	-2.67030	-1.04560
C	-1.22290	-0.72190	-4.21270
H	-1.75570	0.17200	-3.86230
N	1.43130	-0.30920	-0.80680
S	2.77040	-1.15590	-0.41860
O	2.72080	-2.44870	-1.14550
O	4.00780	-0.35630	-0.62100
C	2.52090	2.01410	-0.72850
C	2.99660	2.12250	-2.04190
C	3.08370	2.82100	0.26420
C	4.02250	3.01390	-2.35470
H	2.57520	1.49100	-2.81840
C	4.11480	3.71730	-0.04260
H	2.71290	2.75300	1.28390
C	4.58780	3.81640	-1.35380
H	4.38400	3.08460	-3.37710
H	4.54330	4.33590	0.74140
H	5.38690	4.51140	-1.59660
C	-0.19310	3.12720	-0.87730
C	-0.14160	4.04710	-1.93300
C	-0.49980	3.58890	0.41260
C	-0.38720	5.40610	-1.70980
H	0.10110	3.69960	-2.93390
C	-0.74770	4.94580	0.63720
H	-0.54670	2.88880	1.24050
C	-0.69100	5.85950	-0.42210
H	-0.34050	6.10670	-2.53890
H	-0.98280	5.29030	1.64060
H	-0.88200	6.91420	-0.24490
C	-0.01320	-3.37440	-1.63160
Ru	-0.53310	-1.26280	-0.87970
H	-0.77390	-1.07650	0.84360
C	2.76850	-1.56170	1.33740
C	2.34270	-2.82020	1.76700
C	3.19260	-0.59890	2.25920
C	2.34680	-3.11430	3.13120
C	3.18460	-0.90660	3.62070
C	2.76220	-2.16540	4.07830
H	-3.12950	-1.13140	-2.28210
H	0.89490	-2.34960	-3.30390

H	3.53210	0.37340	1.91760
H	3.50550	-0.15530	4.33760
H	2.00790	-4.09170	3.46480
H	2.01580	-3.55630	1.04170
C	2.76950	-2.49610	5.55170
H	1.97050	-3.20000	5.80390
H	2.64340	-1.59660	6.16150
H	3.72070	-2.96050	5.84030
O	-3.77060	0.11460	0.04130
O	-1.36110	0.81610	1.91880
O	-4.62160	0.48510	2.10250
O	-2.54150	-2.97770	3.02030
N	-3.37840	-2.06800	2.39910
C	-2.80500	-1.01580	1.94220
C	-1.37670	-0.46150	2.03920
C	-0.39870	-1.09000	3.02660
H	-0.32510	-2.16340	2.86440
H	0.57250	-0.64700	2.80390
C	-0.79000	-0.78530	4.47960
H	-0.04970	-1.21940	5.15950
H	-0.82570	0.29430	4.65710
H	-1.76760	-1.21190	4.72570
C	-3.77350	-0.08140	1.24420
C	-5.59860	1.41960	1.54480
H	-5.05080	2.21440	1.03060
H	-6.20450	0.88030	0.81120
C	-6.42610	1.94540	2.69980
H	-5.79550	2.46660	3.42650
H	-7.17000	2.65180	2.31810
H	-6.95150	1.13070	3.20730
C	-3.31330	-4.09160	3.50240
H	-4.05150	-3.75610	4.23710
H	-3.80830	-4.60250	2.67070
H	-2.58760	-4.75640	3.97330
C	-2.06720	-1.35690	-5.33780
H	-3.06600	-1.63090	-4.98250
H	-1.57880	-2.26090	-5.71970
H	-2.18070	-0.65140	-6.16780
C	0.15010	-0.28060	-4.73460
H	0.02540	0.51440	-5.47620
H	0.67800	-1.10750	-5.22270
H	0.78370	0.09780	-3.92710
C	-1.12310	-4.46470	0.36900
H	-1.72350	-5.34470	0.10780
H	-1.55570	-4.01280	1.26190
H	-0.10920	-4.80000	0.59890
H	-3.11800	-2.69250	-0.37160
H	0.89360	-3.92130	-1.41270

99
iTS-2-major-E_330-3
G = -2623.2853256

N	-1.14100	0.68710	-0.36620
H	-1.96450	1.09870	-0.79950
H	-1.26850	0.75350	0.65710
C	1.30500	0.73070	-0.14070
H	1.36010	1.06530	0.90110
C	0.07940	1.43010	-0.77510
H	0.14530	1.33350	-1.85980

C	-1.58310	-1.32510	-3.02860
C	-0.36740	-2.03430	-2.95150
C	-2.65120	-1.73330	-2.15370
C	-1.27720	-3.57730	-1.22720
C	-2.50760	-2.83730	-1.28820
C	-1.82300	-0.16300	-3.97710
H	-2.30210	0.63160	-3.38980
N	1.04410	-0.72600	-0.17200
S	2.20580	-1.74800	0.29530
O	1.56710	-2.99960	0.78810
O	3.26320	-1.98680	-0.73320
C	2.57200	1.20370	-0.83840
C	2.84440	0.83300	-2.16180
C	3.46240	2.05310	-0.17310
C	3.98280	1.30980	-2.81320
H	2.17340	0.14910	-2.67110
C	4.60570	2.53460	-0.82070
H	3.26450	2.33320	0.85840
C	4.86780	2.16540	-2.14350
H	4.18380	1.01220	-3.83880
H	5.29010	3.19170	-0.29120
H	5.75560	2.53590	-2.64840
C	0.00890	2.90290	-0.41870
C	0.06600	3.87270	-1.42720
C	-0.12440	3.31410	0.91700
C	-0.00470	5.23400	-1.11070
H	0.16880	3.56160	-2.46370
C	-0.19660	4.67160	1.23560
H	-0.18650	2.57440	1.71100
C	-0.13560	5.63620	0.22220
H	0.04210	5.97590	-1.90280
H	-0.30450	4.97690	2.27250
H	-0.19200	6.69220	0.47080
C	-0.21410	-3.13020	-2.03930
Ru	-0.89610	-1.37240	-0.80960
H	-1.25610	-1.52740	0.84640
C	3.03850	-0.95280	1.68570
C	2.27620	-0.40040	2.72060
C	4.43240	-0.90010	1.72060
C	2.92550	0.19800	3.79980
C	5.06800	-0.30110	2.81080
C	4.32810	0.25900	3.86340
H	-3.57650	-1.16620	-2.14140
H	0.48830	-1.72490	-3.53830
H	5.00880	-1.31440	0.90080
H	6.15390	-0.26350	2.83840
H	2.33280	0.63250	4.60080
H	1.19150	-0.40620	2.67280
C	5.01660	0.94630	5.01830
H	4.46760	0.79870	5.95350
H	5.07560	2.02760	4.84120
H	6.03780	0.57770	5.15200
O	-4.37740	0.72960	2.89350
O	-1.26120	0.10510	2.39400
O	-3.95550	0.70850	0.66620
O	-3.47360	-3.37230	1.85880
N	-4.03650	-2.10820	1.81180
C	-3.20550	-1.13970	1.93160

C	-1.69240	-1.07860	2.18390
C	-1.05190	-2.24180	2.94860
H	-1.26870	-3.18920	2.45870
H	0.02690	-2.08880	2.90810
C	-1.54440	-2.26460	4.40370
H	-1.32130	-1.32010	4.91030
H	-2.62580	-2.43390	4.45190
H	-1.05240	-3.07320	4.95390
C	-3.90270	0.20630	1.90210
C	-4.65940	1.98240	0.47780
H	-4.93750	1.96470	-0.57690
H	-5.56190	1.96630	1.09270
C	-3.76570	3.16490	0.80940
H	-2.87750	3.18500	0.17370
H	-4.32580	4.09130	0.64410
H	-3.44640	3.13520	1.85400
C	-4.51170	-4.36580	1.78760
H	-5.07840	-4.26330	0.85670
H	-3.98980	-5.32360	1.80600
H	-5.18020	-4.28270	2.64960
C	-2.82230	-0.60250	-5.07030
H	-3.76120	-0.96510	-4.64040
H	-2.39250	-1.40450	-5.68130
H	-3.05240	0.24270	-5.72720
C	-0.55210	0.41060	-4.61360
H	-0.80570	1.29650	-5.20360
H	-0.08140	-0.31380	-5.28760
H	0.18660	0.70760	-3.86500
C	-1.11850	-4.75390	-0.30490
H	-0.08140	-4.83930	0.02600
H	-1.40100	-5.67680	-0.82570
H	-1.76080	-4.64700	0.57130
H	-3.31930	-3.10200	-0.62200
H	0.75620	-3.60570	-1.94780

99
iTS-2-major-E_0
G = -2623.2851559

N	-1.10990	0.57700	-0.44570
H	-1.97450	0.95230	-0.83070
H	-1.19670	0.64360	0.58210
C	1.32240	0.74580	-0.24050
H	1.37270	1.16250	0.77280
C	0.06540	1.35410	-0.91090
H	0.13250	1.19890	-1.99010
C	-1.22940	-1.51630	-3.13050
C	-0.02410	-2.20980	-2.89330
C	-2.38170	-1.90660	-2.36440
C	-1.09480	-3.69970	-1.22960
C	-2.31810	-2.97630	-1.44550
C	-1.27380	-0.43010	-4.19030
H	-0.27660	0.02660	-4.21980
N	1.10730	-0.71740	-0.15760
S	2.28070	-1.64510	0.45430
O	1.67250	-2.90770	0.95650
O	3.43490	-1.86310	-0.46970
C	2.57380	1.19170	-0.98130
C	3.50660	2.01840	-0.34680
C	2.80060	0.80300	-2.30880

C	4.64940	2.45600	-1.02650
H	3.34370	2.31380	0.68650
C	3.93790	1.23590	-2.99160
H	2.09150	0.14530	-2.80180
C	4.86740	2.06630	-2.35090
H	5.36690	3.09610	-0.52030
H	4.10260	0.92550	-4.01980
H	5.75410	2.40320	-2.88070
C	-0.06450	2.84040	-0.63750
C	-0.33850	3.31140	0.65620
C	0.09680	3.76340	-1.67830
C	-0.45240	4.68160	0.90150
H	-0.46940	2.60980	1.47590
C	-0.00980	5.13700	-1.43410
H	0.30880	3.40580	-2.68260
C	-0.28600	5.59910	-0.14330
H	-0.67190	5.03250	1.90580
H	0.11960	5.84220	-2.25030
H	-0.37350	6.66480	0.04810
C	0.04530	-3.27860	-1.94390
Ru	-0.76800	-1.47720	-0.85780
H	-1.19670	-1.62960	0.78000
C	2.94460	-0.73930	1.86660
C	2.06330	-0.19790	2.80910
C	4.32330	-0.58050	2.00370
C	2.58130	0.49240	3.90380
C	4.82620	0.11040	3.10940
C	3.96680	0.65340	4.07600
H	-3.31280	-1.36140	-2.46470
H	0.88080	-1.90010	-3.40570
H	4.99120	-0.97930	1.24850
H	5.90070	0.23620	3.21440
H	1.89730	0.92470	4.63000
H	0.98860	-0.27700	2.67300
C	4.50900	1.38150	5.28210
H	3.88180	2.24010	5.54240
H	5.52960	1.73580	5.11140
H	4.52950	0.71710	6.15510
O	-3.94380	0.67810	0.38730
O	-1.31140	0.03260	2.30940
O	-4.27760	0.70220	2.62700
O	-3.52190	-3.42670	1.68580
N	-4.05980	-2.15750	1.55590
C	-3.22450	-1.19880	1.71630
C	-1.73780	-1.14990	2.09200
C	-1.15820	-2.30300	2.91450
H	-1.33250	-3.25580	2.41810
H	-0.07950	-2.14350	2.95940
C	-1.76710	-2.31580	4.32560
H	-1.31860	-3.11790	4.92050
H	-1.58870	-1.36650	4.84090
H	-2.84810	-2.48920	4.28680
C	-3.85000	0.16250	1.48910
C	-4.81760	2.06360	2.56770
H	-5.48120	2.13530	1.70300
H	-5.40330	2.14730	3.48380
C	-3.69460	3.08610	2.51250
H	-3.10710	2.97750	1.59810

H	-4.12400	4.09330	2.53080
H	-3.02860	2.97750	3.37370
C	-4.56260	-4.41060	1.55150
H	-5.04850	-4.32380	0.57450
H	-4.05630	-5.37330	1.63510
H	-5.29920	-4.29980	2.35280
C	-2.29790	0.68230	-3.92620
H	-2.14290	1.16070	-2.95630
H	-3.32560	0.30590	-3.96170
H	-2.20380	1.45460	-4.69570
C	-1.53130	-1.08880	-5.56390
H	-2.51400	-1.57440	-5.57960
H	-0.77210	-1.84360	-5.79180
H	-1.51210	-0.33050	-6.35380
C	-1.02700	-4.84240	-0.25530
H	-1.74660	-4.70520	0.55350
H	-0.02470	-4.92230	0.17120
H	-1.26460	-5.78190	-0.76870
H	-3.19420	-3.22870	-0.86020
H	1.00320	-3.74200	-1.73600

99
iTS-2-major-E_0-2
G = -2623.2820697

N	-1.03570	0.62350	-0.53120
H	-1.90620	0.93260	-0.95910
H	-1.16250	0.74270	0.49350
C	1.40170	0.88190	-0.37030
H	1.42290	1.23390	0.67140
C	0.10270	1.43600	-1.01300
H	0.16750	1.27520	-2.09250
C	-1.34270	-1.54220	-3.03770
C	-0.08700	-2.18070	-2.91990
C	-2.38810	-1.96850	-2.14920
C	-0.90350	-3.68080	-1.12460
C	-2.16990	-3.00720	-1.21740
C	-1.53820	-0.47660	-4.10360
H	-0.60430	0.09740	-4.15290
N	1.31070	-0.59780	-0.40360
S	2.54820	-1.45130	0.21780
O	2.16400	-2.88430	0.23250
O	3.83290	-1.13060	-0.46690
C	2.57390	1.50360	-1.12120
C	3.27600	2.58060	-0.57100
C	2.89030	1.07700	-2.41820
C	4.29250	3.21500	-1.29590
H	3.02060	2.93790	0.42350
C	3.89990	1.70680	-3.14640
H	2.34990	0.23790	-2.84590
C	4.60830	2.77870	-2.58530
H	4.83090	4.04870	-0.85350
H	4.13780	1.36320	-4.14950
H	5.39630	3.26830	-3.15080
C	-0.09490	2.91710	-0.74770
C	0.00050	3.83790	-1.79840
C	-0.37160	3.38410	0.54660
C	-0.17170	5.20620	-1.56360
H	0.21620	3.48280	-2.80280
C	-0.54870	4.74940	0.78300

H	-0.45680	2.68270	1.37250
C	-0.44710	5.66510	-0.27130
H	-0.09260	5.90970	-2.38760
H	-0.76790	5.09770	1.78830
H	-0.58420	6.72690	-0.08670
C	0.12710	-3.23360	-1.97540
Ru	-0.64170	-1.42130	-0.84310
H	-0.99650	-1.45920	0.85290
C	2.83070	-0.98500	1.94170
C	2.48940	-1.87590	2.96220
C	3.40520	0.25310	2.24790
C	2.70210	-1.51120	4.29300
C	3.59800	0.61010	3.58360
C	3.24780	-0.26180	4.62700
H	-3.34590	-1.46290	-2.14840
H	0.74550	-1.83590	-3.52480
H	3.72120	0.92380	1.45780
H	4.03500	1.57790	3.81590
H	2.43110	-2.20700	5.08310
H	2.06350	-2.84170	2.71510
C	3.48820	0.11590	6.06940
H	4.45100	-0.27970	6.41620
H	2.71220	-0.29570	6.72220
H	3.51150	1.20180	6.19900
O	-3.92710	0.41260	0.09880
O	-1.35360	0.32860	2.16080
O	-4.37750	0.69740	2.30030
O	-3.12450	-3.40840	1.98920
N	-3.81180	-2.24300	1.68150
C	-3.08970	-1.18500	1.69110
C	-1.62140	-0.92290	2.05780
C	-0.93780	-1.87480	3.04110
H	-0.90590	-2.89120	2.64840
H	0.08360	-1.50630	3.13150
C	-1.62770	-1.84700	4.41320
H	-1.07610	-2.47520	5.12050
H	-1.66140	-0.82930	4.81580
H	-2.65170	-2.22930	4.34950
C	-3.84230	0.05820	1.26300
C	-5.01660	1.99040	2.03740
H	-5.63230	1.89690	1.14000
H	-5.65920	2.14130	2.90550
C	-3.97110	3.08570	1.90310
H	-3.31600	2.90400	1.04780
H	-4.47350	4.04770	1.75730
H	-3.35740	3.14850	2.80650
C	-4.04050	-4.51590	2.02720
H	-4.78040	-4.37450	2.82090
H	-4.53960	-4.63570	1.06030
H	-3.42160	-5.38880	2.24050
C	-2.69090	0.50020	-3.83730
H	-2.59310	1.01120	-2.87640
H	-3.66110	-0.00750	-3.85460
H	-2.70570	1.26640	-4.61830
C	-1.73490	-1.17630	-5.46700
H	-1.82610	-0.42880	-6.26210
H	-2.64880	-1.78150	-5.45960
H	-0.89140	-1.83160	-5.70520

C	-0.68660	-4.78970	-0.13110
H	-1.28250	-4.62160	0.76880
H	0.36780	-4.85250	0.14630
H	-0.99160	-5.74820	-0.56820
H	-2.96420	-3.27640	-0.53220
H	1.12170	-3.64580	-1.86150

99
iTS-2-major-E_330-2
G = -2623.2784721

N	-1.27950	0.69760	-0.57300
H	-2.01790	1.04970	-1.17910
H	-1.59630	0.83900	0.40340
C	1.06680	0.76660	0.07520
H	0.78530	0.91990	1.12520
C	-0.02030	1.44910	-0.79100
H	0.25180	1.31610	-1.84090
C	-1.25290	-1.55360	-3.06030
C	-0.30860	-2.51540	-2.63730
C	-2.51720	-1.54480	-2.37190
C	-1.88700	-3.51020	-1.01460
C	-2.83640	-2.49620	-1.37790
C	-0.98740	-0.53250	-4.15120
H	-1.41810	0.41780	-3.80960
N	0.97060	-0.69600	-0.19690
S	2.27300	-1.65070	-0.24240
O	1.84550	-3.04370	0.06950
O	3.07880	-1.54860	-1.49650
C	2.39210	1.48040	-0.14980
C	3.02110	1.49880	-1.40180
C	2.95570	2.21930	0.89810
C	4.19750	2.22610	-1.59650
H	2.60720	0.92300	-2.22060
C	4.12990	2.95230	0.70830
H	2.47830	2.20930	1.87370
C	4.75760	2.95580	-0.54150
H	4.67690	2.22270	-2.57170
H	4.55640	3.51160	1.53640
H	5.67420	3.51940	-0.69210
C	-0.17320	2.93060	-0.50380
C	0.15810	3.87040	-1.48880
C	-0.62760	3.38440	0.74470
C	0.04270	5.24120	-1.23520
H	0.51520	3.52800	-2.45660
C	-0.74610	4.75340	0.99880
H	-0.89250	2.67010	1.51800
C	-0.41040	5.68660	0.01100
H	0.30480	5.95740	-2.00900
H	-1.10150	5.09100	1.96850
H	-0.50280	6.75050	0.21060
C	-0.62560	-3.49290	-1.63920
Ru	-0.98520	-1.38420	-0.79240
H	-1.38490	-1.34360	0.90060
C	3.34310	-1.11480	1.10430
C	2.79710	-0.98390	2.38370
C	4.69070	-0.84560	0.87070
C	3.61280	-0.57150	3.43530
C	5.49720	-0.43610	1.93480
C	4.97460	-0.29050	3.22880

H	-3.23130	-0.75610	-2.58790
H	0.69840	-2.50230	-3.03670
H	5.09090	-0.92600	-0.13360
H	6.54460	-0.21100	1.75140
H	3.18470	-0.45580	4.42780
H	1.74430	-1.17870	2.54840
C	5.85380	0.13940	4.37890
H	6.21440	-0.73440	4.93600
H	5.30460	0.77110	5.08400
H	6.73000	0.69180	4.02720
O	-4.12910	0.12480	-0.00270
O	-1.73770	0.51270	2.12890
O	-4.81630	0.75600	2.05980
O	-3.48100	-3.12070	2.94890
N	-4.13470	-2.08800	2.29420
C	-3.38370	-1.11740	1.92830
C	-1.90530	-0.76200	2.14760
C	-1.05010	-1.55630	3.15150
H	-0.24730	-0.86110	3.41710
H	-1.67040	-1.68190	4.04780
C	-0.45980	-2.90170	2.72910
H	-1.24150	-3.62860	2.51140
H	0.16860	-2.79570	1.84140
H	0.15780	-3.29880	3.54270
C	-4.14430	-0.01930	1.20710
C	-5.48550	1.93520	1.50580
H	-5.99900	1.64480	0.58650
H	-6.22210	2.19600	2.26680
C	-4.48590	3.05700	1.27220
H	-3.73830	2.76960	0.52880
H	-5.01580	3.94330	0.90790
H	-3.97220	3.31790	2.20210
C	-4.43060	-4.13280	3.32260
H	-4.91960	-4.54720	2.43510
H	-3.84150	-4.90450	3.82060
H	-5.17740	-3.72490	4.01090
C	-1.73650	-0.94950	-5.43460
H	-2.80960	-1.06750	-5.25200
H	-1.34660	-1.90110	-5.81400
H	-1.60310	-0.18980	-6.21220
C	0.50250	-0.30590	-4.43420
H	0.62610	0.54120	-5.11570
H	0.95790	-1.18250	-4.90840
H	1.05710	-0.09310	-3.51650
C	-2.24070	-4.55420	0.00680
H	-2.80620	-5.35580	-0.48380
H	-2.86340	-4.13750	0.79960
H	-1.34510	-4.99110	0.45350
H	-3.78410	-2.42890	-0.85710
H	0.14320	-4.18290	-1.31770

99

iTS-4-minor-Z_330-3

G = -2623.2839199

N	-1.84310	-0.22540	-1.63360
H	-2.50610	-0.65960	-2.27320
H	-2.35930	0.53960	-1.16410
C	0.43690	0.71330	-1.44890
H	0.13820	1.67500	-1.01040

C	-0.68870	0.27610	-2.42030
H	-0.32560	-0.58060	-2.99370
C	-2.91030	-3.10290	-0.07700
C	-2.03150	-3.32690	-1.18140
C	-2.30710	-3.02590	1.20680
C	-0.08250	-3.57940	0.30320
C	-0.91780	-3.26180	1.40470
C	-4.41570	-2.99430	-0.25810
H	-4.83910	-2.73790	0.71990
N	0.50610	-0.30040	-0.36700
S	1.85570	-0.46480	0.51510
O	1.46780	-0.96620	1.86450
O	2.92460	-1.27020	-0.14430
C	1.70110	0.93800	-2.26850
C	2.32570	-0.12250	-2.93860
C	2.21750	2.22850	-2.41700
C	3.44440	0.10530	-3.74160
H	1.94740	-1.13190	-2.81350
C	3.33510	2.46470	-3.22260
H	1.75370	3.05220	-1.88610
C	3.95310	1.40200	-3.88870
H	3.92140	-0.72740	-4.25150
H	3.72510	3.47400	-3.32190
H	4.82420	1.57970	-4.51330
C	-1.07950	1.39010	-3.37410
C	-0.69910	1.33190	-4.72080
C	-1.79560	2.50690	-2.91740
C	-1.02220	2.37150	-5.59870
H	-0.13850	0.47360	-5.08160
C	-2.12460	3.54410	-3.79380
H	-2.09300	2.57540	-1.87450
C	-1.73720	3.48150	-5.13690
H	-0.71690	2.31330	-6.63970
H	-2.67550	4.40460	-3.42430
H	-1.99020	4.29030	-5.81660
C	-0.64860	-3.59450	-1.00300
Ru	-1.23160	-1.54890	-0.10870
H	-1.88170	-0.26520	0.84070
C	2.56620	1.17650	0.73780
C	1.75790	2.21170	1.20310
C	3.92080	1.39230	0.47770
C	2.31140	3.47170	1.41920
C	4.46330	2.65960	0.69620
C	3.67080	3.71810	1.17000
H	-2.92330	-2.78110	2.06500
H	-2.42870	-3.30490	-2.19170
H	4.53240	0.58500	0.09160
H	5.51540	2.83120	0.48360
H	1.67340	4.27430	1.77910
H	0.70560	2.04660	1.37550
C	4.26610	5.08240	1.42290
H	3.53180	5.87450	1.24680
H	5.13460	5.26400	0.78280
H	4.59980	5.17010	2.46450
O	-2.88090	3.99420	1.81800
O	-3.40200	1.26710	0.06480
O	-1.12880	3.53970	0.45980
O	-0.54260	2.47220	3.42840

N	-1.26660	1.42650	2.86490
C	-1.95180	1.79530	1.84930
C	-2.90320	0.82700	1.15150
C	-3.77690	-0.00630	2.09280
H	-4.25510	-0.77960	1.49080
H	-3.15220	-0.49150	2.84710
C	-4.84360	0.87410	2.76350
H	-5.47760	0.26290	3.41360
H	-4.38700	1.65840	3.37660
H	-5.48040	1.35380	2.01370
C	-2.03720	3.23180	1.37890
C	-1.16180	4.91810	-0.04410
H	-2.13360	5.07230	-0.52140
H	-1.08070	5.58950	0.81440
C	-0.01430	5.08830	-1.01550
H	-0.11820	4.41090	-1.86540
H	-0.02160	6.11580	-1.39290
H	0.94510	4.90560	-0.52580
C	0.35390	1.96140	4.43170
H	0.85730	2.84020	4.83680
H	-0.20710	1.44770	5.21810
H	1.08420	1.28560	3.97730
C	-4.98190	-4.37030	-0.66360
H	-4.70530	-5.14340	0.06100
H	-4.60230	-4.67230	-1.64650
H	-6.07480	-4.32720	-0.72080
C	-4.83650	-1.90200	-1.25410
H	-4.48290	-0.91800	-0.93250
H	-5.92850	-1.86550	-1.32730
H	-4.45030	-2.10640	-2.25940
C	1.36020	-3.93940	0.51090
H	1.97750	-3.61730	-0.32830
H	1.43050	-5.03080	0.60030
H	1.75210	-3.48390	1.42040
H	-0.49310	-3.18690	2.39840
H	-0.01750	-3.78190	-1.86460

99
iTS-4-minor-Z_0-7
G = -2623.2830264

N	-1.80680	-0.37680	-1.70220
H	-2.39740	-0.87170	-2.36840
H	-2.40170	0.36060	-1.28100
C	0.38070	0.70430	-1.38600
H	-0.05670	1.57880	-0.89070
C	-0.64730	0.20090	-2.42940
H	-0.18860	-0.62730	-2.97680
C	-2.88250	-3.16830	-0.00450
C	-2.01380	-3.43530	-1.10490
C	-2.26770	-3.04170	1.27080
C	-0.05350	-3.64620	0.37170
C	-0.87840	-3.28090	1.46650
C	-4.38840	-3.06240	-0.18260
H	-4.80660	-2.75910	0.78400
N	0.53240	-0.37580	-0.37950
S	1.89870	-0.55750	0.47200
O	1.54390	-1.10710	1.81120
O	2.95220	-1.33620	-0.24410
C	1.62690	1.16290	-2.12950

C	2.42700	0.25780	-2.83880
C	1.94120	2.52590	-2.17710
C	3.52520	0.70810	-3.57490
H	2.20330	-0.80270	-2.79520
C	3.03460	2.98330	-2.91600
H	1.33710	3.23140	-1.61610
C	3.83240	2.07350	-3.61740
H	4.14170	-0.00550	-4.11480
H	3.26800	4.04430	-2.93490
H	4.68760	2.42320	-4.18920
C	-1.05590	1.28710	-3.40670
C	-1.85350	2.36450	-2.99200
C	-0.60020	1.24940	-4.73100
C	-2.18400	3.38630	-3.88640
H	-2.21530	2.40970	-1.96890
C	-0.92660	2.27270	-5.62660
H	0.02400	0.42250	-5.05890
C	-1.71930	3.34580	-5.20580
H	-2.79900	4.21650	-3.55010
H	-0.56200	2.23170	-6.64920
H	-1.97360	4.14250	-5.89920
C	-0.62950	-3.69910	-0.92830
Ru	-1.19680	-1.62100	-0.10740
H	-1.84490	-0.27980	0.78550
C	2.60470	1.07460	0.75240
C	3.92360	1.34830	0.38980
C	1.82050	2.04170	1.37800
C	4.45420	2.61080	0.66090
C	2.36390	3.29390	1.65150
C	3.68690	3.60170	1.29460
H	-2.87520	-2.76010	2.12380
H	-2.41850	-3.45000	-2.11230
H	0.79430	1.82580	1.63340
H	1.74740	4.04110	2.14240
H	5.47660	2.83230	0.36550
H	4.51330	0.59270	-0.11640
C	4.26960	4.96000	1.60290
H	3.50770	5.74280	1.53260
H	5.08740	5.20830	0.91990
H	4.67180	4.98520	2.62350
O	-2.79840	4.04040	1.52880
O	-3.35290	1.20970	-0.06320
O	-1.05370	3.40120	0.22700
O	-0.65390	2.59540	3.36260
N	-1.35200	1.51620	2.82760
C	-1.95160	1.81540	1.73750
C	-2.86910	0.80740	1.04710
C	-3.76100	0.00700	2.00100
H	-4.21730	-0.79500	1.42100
H	-3.15370	-0.44130	2.79130
C	-4.85340	0.90410	2.60470
H	-5.50000	0.31480	3.26260
H	-4.42110	1.71810	3.19630
H	-5.47340	1.34500	1.81790
C	-1.97950	3.21630	1.16160
C	-1.02740	4.69140	-0.47050
H	-0.67680	4.43800	-1.47100
H	-2.04960	5.06860	-0.53440

C	-0.09720	5.66380	0.23090
H	0.92460	5.27760	0.25070
H	-0.09880	6.61430	-0.31290
H	-0.42590	5.85110	1.25710
C	0.09490	2.17160	4.51580
H	-0.57670	1.77280	5.28190
H	0.83930	1.42180	4.23150
H	0.59120	3.07270	4.87870
C	-4.96210	-4.45390	-0.51940
H	-4.68510	-5.19280	0.23990
H	-4.58850	-4.80390	-1.48870
H	-6.05500	-4.40880	-0.57340
C	-4.80950	-2.01780	-1.22870
H	-4.43170	-2.27400	-2.22510
H	-4.44680	-1.02130	-0.96100
H	-5.90170	-1.97760	-1.29580
C	1.38710	-4.01810	0.57360
H	1.45160	-5.11200	0.63200
H	1.78050	-3.58820	1.49410
H	2.00630	-3.67720	-0.25740
H	-0.44460	-3.17000	2.45270
H	-0.00680	-3.92110	-1.78790

99
iTS-4-minor-Z_0-6
G = -2623.2829865

N	-1.80480	-0.35860	-1.71290
H	-2.39840	-0.84410	-2.38320
H	-2.39680	0.37510	-1.28190
C	0.38770	0.71280	-1.39320
H	-0.04390	1.58240	-0.88400
C	-0.64730	0.22630	-2.43790
H	-0.19300	-0.59510	-2.99890
C	-2.87550	-3.17610	-0.06540
C	-1.99750	-3.42960	-1.16250
C	-2.27130	-3.06490	1.21570
C	-0.04900	-3.65460	0.32810
C	-0.88300	-3.30410	1.42000
C	-4.38040	-3.06920	-0.25260
H	-4.80550	-2.78230	0.71600
N	0.54260	-0.38360	-0.40590
S	1.90420	-0.56500	0.45390
O	1.54350	-1.11640	1.79120
O	2.96030	-1.34290	-0.25850
C	1.63230	1.17520	-2.13730
C	2.43020	0.27270	-2.85210
C	1.94810	2.53810	-2.17810
C	3.52790	0.72530	-3.58730
H	2.20490	-0.78770	-2.81320
C	3.04140	2.99780	-2.91590
H	1.34570	3.24150	-1.61240
C	3.83710	2.09050	-3.62300
H	4.14280	0.01380	-4.13180
H	3.27630	4.05860	-2.92950
H	4.69220	2.44200	-4.19380
C	-1.05910	1.32660	-3.39800
C	-1.85580	2.39750	-2.96540
C	-0.60830	1.30860	-4.72430
C	-2.19030	3.43130	-3.84410

H	-2.21410	2.42850	-1.94050
C	-0.93870	2.34400	-5.60440
H	0.01530	0.48690	-5.06630
C	-1.73080	3.41030	-5.16570
H	-2.80490	4.25600	-3.49380
H	-0.57750	2.31750	-6.62870
H	-1.98870	4.21630	-5.84690
C	-0.61490	-3.69440	-0.97740
Ru	-1.19070	-1.62540	-0.13730
H	-1.83520	-0.30000	0.77700
C	2.61150	1.06530	0.74260
C	3.93040	1.33860	0.37910
C	1.83110	2.02950	1.37780
C	4.46520	2.59750	0.65950
C	2.37900	3.27780	1.66030
C	3.70220	3.58480	1.30350
H	-2.88620	-2.79570	2.06750
H	-2.39440	-3.43400	-2.17310
H	0.80510	1.81470	1.63490
H	1.76610	4.02230	2.15960
H	5.48770	2.81810	0.36410
H	4.51710	0.58550	-0.13400
C	4.28920	4.93870	1.62280
H	3.53250	5.72600	1.54740
H	5.11550	5.18480	0.94950
H	4.68030	4.95820	2.64790
O	-2.79310	4.01050	1.57420
O	-3.34830	1.20340	-0.04990
O	-1.04780	3.39190	0.26400
O	-0.63700	2.54150	3.38480
N	-1.33770	1.47040	2.83700
C	-1.94180	1.78510	1.75410
C	-2.86270	0.78790	1.05380
C	-3.75140	-0.02760	1.99780
H	-4.21000	-0.81960	1.40570
H	-3.14190	-0.48880	2.77890
C	-4.84120	0.86050	2.61930
H	-5.48640	0.26090	3.26940
H	-4.40650	1.66420	3.22300
H	-5.46330	1.31480	1.84180
C	-1.97240	3.19310	1.19680
C	-1.02830	4.69010	-0.41870
H	-0.66250	4.45170	-1.41740
H	-2.05440	5.05520	-0.49100
C	-0.11920	5.66710	0.30400
H	0.90750	5.29460	0.33040
H	-0.12770	6.62340	-0.22950
H	-0.46180	5.83850	1.32830
C	0.12020	2.09990	4.52570
H	0.61750	2.99570	4.90010
H	-0.54590	1.68720	5.28910
H	0.86350	1.35620	4.22330
C	-4.94930	-4.45560	-0.61670
H	-4.67680	-5.20660	0.13210
H	-4.56790	-4.78870	-1.58890
H	-6.04180	-4.41120	-0.67820
C	-4.79580	-2.00770	-1.28360
H	-4.40850	-2.24510	-2.28120

H	-4.43900	-1.01500	-0.99450
H	-5.88750	-1.96970	-1.35960
C	1.39030	-4.02700	0.53800
H	1.45520	-5.12110	0.59000
H	1.77660	-3.60270	1.46420
H	2.01570	-3.68020	-0.28590
H	-0.45810	-3.20560	2.41140
H	0.01520	-3.90530	-1.83440

99

iTS-4-minor-Z_0

G = -2623.2827022

N	-1.83530	-0.27660	-1.72620
H	-2.49760	-0.74230	-2.34430
H	-2.34230	0.51400	-1.28430
C	0.40770	0.73270	-1.56850
H	0.03960	1.67090	-1.13620
C	-0.68360	0.19850	-2.53290
H	-0.27600	-0.68500	-3.03270
C	-2.83110	-3.10930	-0.02020
C	-1.98360	-3.36020	-1.14130
C	-2.18990	-2.96870	1.24160
C	0.01840	-3.50190	0.28690
C	-0.79060	-3.17090	1.40450
C	-4.34360	-3.05470	-0.15260
H	-4.73590	-2.66380	0.79310
N	0.54310	-0.27280	-0.48710
S	1.86140	-0.32520	0.46190
O	1.46210	-0.93730	1.75780
O	3.02310	-0.98060	-0.20900
C	1.63710	1.04460	-2.40810
C	2.41630	0.02430	-2.96710
C	1.94430	2.37510	-2.71290
C	3.49670	0.33100	-3.79730
H	2.19110	-1.00880	-2.72630
C	3.01970	2.68900	-3.54760
H	1.34200	3.17310	-2.29040
C	3.80390	1.66570	-4.08960
H	4.10000	-0.47020	-4.21550
H	3.24740	3.72830	-3.76790
H	4.64580	1.90400	-4.73370
C	-1.09090	1.21980	-3.57820
C	-0.67580	1.06710	-4.90750
C	-1.85440	2.34340	-3.22880
C	-1.00780	2.02320	-5.87240
H	-0.07900	0.20240	-5.18550
C	-2.18990	3.29920	-4.19180
H	-2.18680	2.47720	-2.20340
C	-1.76560	3.14400	-5.51630
H	-0.67500	1.89280	-6.89840
H	-2.77760	4.16710	-3.90580
H	-2.02460	3.88860	-6.26370
C	-0.58880	-3.57920	-0.99840
Ru	-1.18870	-1.52270	-0.15590
H	-1.81400	-0.19540	0.75530
C	2.45130	1.33600	0.85870
C	1.93700	1.97830	1.98620
C	3.46280	1.93240	0.10360
C	2.42810	3.23000	2.34950

C	3.93880	3.19260	0.47230
C	3.43140	3.86160	1.59640
H	-2.78630	-2.70090	2.10750
H	-2.41160	-3.38690	-2.13850
H	3.87330	1.42100	-0.75840
H	4.71620	3.66030	-0.12620
H	2.01320	3.72880	3.22160
H	1.16190	1.50710	2.57360
C	3.95410	5.21890	2.00130
H	4.49810	5.69900	1.18300
H	4.63910	5.13270	2.85380
H	3.13620	5.87930	2.30810
O	-2.03550	4.10570	1.98740
O	-3.04700	1.53390	-0.05220
O	-0.47100	3.14630	0.65810
O	-0.70790	2.08400	3.86090
N	-1.49230	1.22630	3.09730
C	-1.81860	1.72020	1.96220
C	-2.75220	0.94230	1.04050
C	-3.84130	0.14100	1.76080
H	-4.28520	-0.52960	1.02530
H	-3.39900	-0.46420	2.55520
C	-4.91520	1.07870	2.33480
H	-5.70120	0.49410	2.82370
H	-4.49160	1.76320	3.07740
H	-5.37480	1.67610	1.54120
C	-1.46060	3.12830	1.53950
C	0.05990	4.44640	0.24400
H	0.08650	5.09810	1.11970
H	1.08130	4.21600	-0.05860
C	-0.75520	5.04250	-0.88940
H	-1.78850	5.22020	-0.58010
H	-0.31030	5.99940	-1.18220
H	-0.75600	4.38310	-1.76000
C	-0.32230	1.43250	5.08450
H	-1.20640	1.17150	5.67400
H	0.27010	0.53760	4.87000
H	0.28450	2.16590	5.61740
C	-4.88630	-4.48860	-0.32530
H	-4.56640	-5.13580	0.49800
H	-4.52810	-4.92740	-1.26390
H	-5.98120	-4.47860	-0.35000
C	-4.83430	-2.13930	-1.28510
H	-4.49040	-2.48750	-2.26520
H	-4.49060	-1.11060	-1.14390
H	-5.92880	-2.12910	-1.30770
C	1.48240	-3.79880	0.44080
H	1.61940	-4.88680	0.41130
H	1.86640	-3.41510	1.38520
H	2.06320	-3.35600	-0.37050
H	-0.33440	-3.04160	2.37800
H	0.02020	-3.77820	-1.87340

99
iTS-4-minor-Z_0-2
G = -2623.2819357

N	-1.94920	-0.12450	-1.51850
H	-2.61650	-0.54500	-2.16370
H	-2.46900	0.62810	-1.03130

C	0.33250	0.82680	-1.34690
H	0.05920	1.80690	-0.93530
C	-0.80320	0.38950	-2.30940
H	-0.44000	-0.46350	-2.88860
C	-2.68660	-3.10760	-0.77800
C	-1.39620	-3.31960	-1.37980
C	-2.75810	-3.05470	0.63450
C	-0.32420	-3.45130	0.84820
C	-1.58340	-3.24060	1.43500
C	-3.90680	-2.97180	-1.67210
H	-3.55870	-2.54910	-2.62420
N	0.37380	-0.15560	-0.23680
S	1.70850	-0.37680	0.64610
O	1.30620	-0.69400	2.04480
O	2.66530	-1.37120	0.06940
C	1.59660	0.99150	-2.17790
C	2.22890	-0.11970	-2.74900
C	2.10150	2.26860	-2.43900
C	3.35280	0.04540	-3.56000
H	1.85460	-1.11530	-2.53510
C	3.22200	2.44110	-3.25480
H	1.62630	3.13240	-1.98780
C	3.85330	1.32760	-3.81750
H	3.84050	-0.82500	-3.99060
H	3.60560	3.44020	-3.44230
H	4.72930	1.45530	-4.44750
C	-1.20540	1.50300	-3.25850
C	-0.80670	1.46250	-4.60050
C	-1.94850	2.60220	-2.80300
C	-1.13870	2.50190	-5.47520
H	-0.22440	0.61800	-4.95980
C	-2.28730	3.63880	-3.67650
H	-2.25860	2.65780	-1.76300
C	-1.88170	3.59390	-5.01490
H	-0.81820	2.45760	-6.51230
H	-2.85970	4.48550	-3.30800
H	-2.14220	4.40240	-5.69200
C	-0.24270	-3.45020	-0.58840
Ru	-1.31040	-1.48600	-0.04760
H	-2.00540	-0.28790	0.96950
C	2.58780	1.19440	0.69410
C	1.91950	2.33720	1.13640
C	3.93550	1.24830	0.33750
C	2.61500	3.54110	1.22500
C	4.61790	2.46280	0.42700
C	3.97070	3.62760	0.86740
H	-3.70410	-2.87170	1.12820
H	-1.30770	-3.31130	-2.46200
H	4.43510	0.35490	-0.01940
H	5.66650	2.50570	0.14420
H	2.09540	4.42800	1.57830
H	0.87130	2.30000	1.40050
C	4.69730	4.94980	0.92450
H	5.77950	4.80970	1.00080
H	4.36290	5.55070	1.77610
H	4.50160	5.53390	0.01640
O	-2.97480	4.00980	1.90990
O	-3.49500	1.31370	0.19300

O	-1.20060	3.57410	0.57420
O	-0.46180	2.48590	3.37380
N	-1.24460	1.44480	2.88770
C	-2.02270	1.81670	1.94390
C	-3.01640	0.86850	1.28550
C	-3.95310	0.05190	2.18290
H	-4.72110	0.77360	2.49690
H	-4.45050	-0.65810	1.51770
C	-3.37710	-0.65400	3.40990
H	-4.13620	-1.31230	3.84490
H	-2.50720	-1.25830	3.14540
H	-3.06290	0.05880	4.17600
C	-2.12140	3.25340	1.47800
C	-1.23300	4.95610	0.08410
H	-2.21220	5.12480	-0.37260
H	-1.12890	5.61900	0.94700
C	-0.10400	5.12520	-0.90900
H	-0.23880	4.46490	-1.76790
H	-0.10110	6.15920	-1.26860
H	0.86280	4.91690	-0.44530
C	0.50490	1.96550	4.30740
H	1.09930	2.82850	4.61100
H	-0.00220	1.52990	5.17380
H	1.13400	1.21970	3.81420
C	-4.99440	-2.04570	-1.10970
H	-4.59260	-1.06470	-0.83770
H	-5.46790	-2.47700	-0.22110
H	-5.77770	-1.90130	-1.86020
C	-4.46790	-4.37660	-1.97420
H	-4.83160	-4.84900	-1.05450
H	-3.70320	-5.02550	-2.41380
H	-5.30390	-4.30570	-2.67800
C	0.89240	-3.73990	1.68190
H	1.00860	-4.82810	1.76470
H	0.79240	-3.32160	2.68470
H	1.79090	-3.32750	1.22240
H	-1.66420	-3.20220	2.51540
H	0.72940	-3.53470	-1.06230

99
iTS-4-minor-Z_0-5.
G = -2623.2818212

N	-1.92690	-0.15470	-1.54290
H	-2.58220	-0.58220	-2.19580
H	-2.46350	0.58040	-1.04820
C	0.33990	0.83410	-1.35660
H	0.05190	1.80270	-0.92780
C	-0.79080	0.39530	-2.32450
H	-0.41420	-0.43940	-2.92130
C	-2.65060	-3.14930	-0.79730
C	-1.37740	-3.33840	-1.44150
C	-2.67570	-3.11210	0.61700
C	-0.23430	-3.49370	0.74790
C	-1.47520	-3.30030	1.37820
C	-3.90180	-3.02230	-1.64920
H	-3.58700	-2.61700	-2.62000
N	0.40090	-0.16610	-0.26270
S	1.74260	-0.37680	0.61320
O	1.34690	-0.73130	2.00510

O	2.71990	-1.33790	0.01450
C	1.59880	1.03360	-2.18820
C	2.08200	2.32270	-2.42950
C	2.24730	-0.05710	-2.78060
C	3.19650	2.52740	-3.24600
H	1.59410	3.17080	-1.96250
C	3.36550	0.14010	-3.59240
H	1.89000	-1.06220	-2.58280
C	3.84410	1.43440	-3.82970
H	3.56250	3.53600	-3.41760
H	3.86560	-0.71490	-4.03930
H	4.71540	1.58730	-4.46050
C	-1.21460	1.52080	-3.25020
C	-0.81250	1.51810	-4.59190
C	-1.98130	2.59430	-2.77250
C	-1.16410	2.56930	-5.44430
H	-0.21200	0.69410	-4.96820
C	-2.34020	3.64220	-3.62430
H	-2.29470	2.62100	-1.73220
C	-1.93090	3.63510	-4.96230
H	-0.84060	2.55450	-6.48130
H	-2.93150	4.46830	-3.23920
H	-2.20720	4.45250	-5.62230
C	-0.19760	-3.47270	-0.69020
Ru	-1.26550	-1.52330	-0.08870
H	-1.97310	-0.34880	0.94590
C	2.58960	1.21130	0.69200
C	1.89820	2.33160	1.15440
C	3.93320	1.30260	0.32600
C	2.56530	3.55090	1.25440
C	4.58660	2.53160	0.42690
C	3.91600	3.67400	0.89110
H	-3.60690	-2.94300	1.14280
H	-1.32300	-3.31340	-2.52570
H	4.44920	0.42810	-0.05310
H	5.62930	2.60530	0.12900
H	2.02440	4.42180	1.61520
H	0.85170	2.26780	1.41850
C	4.63020	4.99840	1.01570
H	5.05240	5.11630	2.02160
H	3.94570	5.83570	0.84810
H	5.45540	5.07550	0.30160
O	-2.97450	3.92490	1.98900
O	-3.47270	1.25990	0.19880
O	-1.21120	3.53270	0.62480
O	-0.45490	2.38630	3.41300
N	-1.22400	1.35010	2.89600
C	-2.00620	1.73860	1.96200
C	-2.99380	0.79850	1.28360
C	-3.92660	-0.03840	2.16630
H	-4.69270	0.67720	2.49840
H	-4.42750	-0.73320	1.48830
C	-3.34840	-0.77150	3.37630
H	-4.11020	-1.43230	3.80260
H	-2.48470	-1.37720	3.09520
H	-3.02460	-0.07610	4.15410
C	-2.11980	3.18530	1.53120
C	-1.25940	4.92390	0.16160

H	-2.24810	5.09560	-0.27280
H	-1.14150	5.57080	1.03480
C	-0.15100	5.11680	-0.85010
H	-0.29930	4.47090	-1.71770
H	-0.16050	6.15700	-1.19100
H	0.82560	4.90600	-0.40920
C	0.51850	1.85110	4.33140
H	1.16070	1.13260	3.81500
H	1.09750	2.71300	4.66620
H	0.01650	1.37770	5.18090
C	-4.96550	-2.08200	-1.06510
H	-4.55280	-1.09560	-0.83250
H	-5.40450	-2.49210	-0.14920
H	-5.77800	-1.95380	-1.78710
C	-4.47870	-4.43000	-1.90480
H	-4.81030	-4.88350	-0.96370
H	-3.73270	-5.09030	-2.35920
H	-5.33950	-4.36890	-2.57900
C	1.01070	-3.78340	1.53810
H	1.14140	-4.87150	1.59530
H	0.93720	-3.38550	2.55140
H	1.89010	-3.35160	1.05930
H	-1.52110	-3.27690	2.46110
H	0.75850	-3.54650	-1.19700

99

iTS-4-minor-Z_0-4

G = -2623.2809318

N	-1.93490	-0.15240	-1.58790
H	-2.57520	-0.58460	-2.25230
H	-2.47840	0.58920	-1.11040
C	0.31170	0.84370	-1.33670
H	-0.02770	1.78150	-0.88120
C	-0.77750	0.39220	-2.34300
H	-0.37480	-0.44600	-2.91800
C	-2.73990	-3.10770	-0.78010
C	-1.47150	-3.34360	-1.41740
C	-2.76580	-3.04300	0.63350
C	-0.33680	-3.49230	0.77630
C	-1.57250	-3.25190	1.40030
C	-3.98660	-2.96420	-1.63610
H	-3.66070	-2.58610	-2.61420
N	0.38410	-0.19170	-0.27930
S	1.71560	-0.43980	0.59890
O	1.31070	-0.77480	1.99380
O	2.65420	-1.43920	0.00020
C	1.57690	1.13370	-2.13100
C	2.30030	0.10230	-2.74340
C	1.98640	2.45740	-2.32350
C	3.42130	0.39060	-3.52470
H	1.99840	-0.92780	-2.58550
C	3.10410	2.75190	-3.10770
H	1.43590	3.26130	-1.84400
C	3.82750	1.71740	-3.71030
H	3.97920	-0.41910	-3.98730
H	3.41380	3.78490	-3.24060
H	4.70150	1.94170	-4.31570
C	-1.17040	1.50990	-3.29030
C	-0.76040	1.47630	-4.62910

C	-1.90510	2.61360	-2.83180
C	-1.07040	2.52870	-5.49650
H	-0.18430	0.62870	-4.99080
C	-2.21870	3.66500	-3.69670
H	-2.23220	2.65620	-1.79720
C	-1.80010	3.62770	-5.03160
H	-0.74160	2.48990	-6.53120
H	-2.78490	4.51510	-3.32610
H	-2.04140	4.44720	-5.70280
C	-0.29700	-3.49770	-0.66160
Ru	-1.31290	-1.50580	-0.09900
H	-1.99680	-0.29630	0.91140
C	2.62510	1.11260	0.67440
C	1.99360	2.25430	1.16810
C	3.96360	1.15010	0.28140
C	2.71730	3.44120	1.26820
C	4.67290	2.34720	0.38360
C	4.06230	3.51230	0.87250
H	-3.69290	-2.83940	1.15420
H	-1.41510	-3.34170	-2.50190
H	4.43400	0.25740	-0.11400
H	5.71370	2.37680	0.07150
H	2.22880	4.32720	1.66290
H	0.95190	2.23250	1.45910
C	4.81970	4.81670	0.93720
H	5.88790	4.65080	1.10650
H	4.43630	5.46210	1.73320
H	4.71820	5.36480	-0.00810
O	-2.94090	4.02770	1.65920
O	-3.48530	1.30120	0.12370
O	-1.09830	3.48090	0.45560
O	-0.48360	2.53150	3.31460
N	-1.26600	1.48050	2.84730
C	-2.01500	1.82680	1.87000
C	-3.00910	0.87090	1.22240
C	-3.94470	0.06460	2.12970
H	-4.70050	0.79580	2.45140
H	-4.45810	-0.63760	1.46930
C	-3.36720	-0.65070	3.35070
H	-4.12870	-1.30670	3.78500
H	-2.50250	-1.25920	3.07860
H	-3.04480	0.05540	4.11910
C	-2.07340	3.23950	1.32700
C	-1.08240	4.79060	-0.20190
H	-0.49650	4.61390	-1.10390
H	-2.10770	5.04200	-0.48300
C	-0.45800	5.85220	0.68570
H	0.56770	5.58400	0.94910
H	-0.43540	6.80220	0.14150
H	-1.03770	5.99090	1.60150
C	0.43120	2.04610	4.31600
H	-0.12070	1.63480	5.16660
H	1.09280	1.28920	3.88510
H	1.00540	2.92100	4.62380
C	-5.02520	-1.98490	-1.07140
H	-4.58620	-1.00600	-0.85610
H	-5.47660	-2.36650	-0.14920
H	-5.83300	-1.84840	-1.79730

C	-4.60030	-4.36080	-1.86540
H	-4.94440	-4.78730	-0.91610
H	-3.87170	-5.04920	-2.30640
H	-5.45890	-4.29000	-2.54160
C	0.89640	-3.81010	1.57380
H	0.99210	-4.90110	1.64490
H	0.83290	-3.39770	2.58190
H	1.79010	-3.41250	1.09220
H	-1.61990	-3.20880	2.48250
H	0.65720	-3.61040	-1.16470

99
iTS-4-minor-Z_0-3
G = -2623.2797830

N	-1.81240	-0.12510	-1.65420
H	-2.51850	-0.52220	-2.27010
H	-2.28580	0.63940	-1.13930
C	0.51610	0.75290	-1.59310
H	0.34260	1.76910	-1.21840
C	-0.68860	0.35670	-2.49250
H	-0.38190	-0.50500	-3.09110
C	-2.69980	-3.21420	-0.41950
C	-1.67990	-3.31040	-1.42290
C	-2.25800	-3.11450	0.92230
C	0.10030	-3.43950	0.27650
C	-0.87830	-3.23590	1.27480
C	-4.17000	-3.27780	-0.82890
H	-4.23250	-4.14900	-1.49610
N	0.53980	-0.18330	-0.44500
S	1.82480	-0.18970	0.54710
O	1.38430	-0.73160	1.86030
O	3.02260	-0.86150	-0.03800
C	1.74490	0.76510	-2.49360
C	2.30500	-0.43450	-2.94900
C	2.26910	1.97510	-2.95520
C	3.37850	-0.42390	-3.83950
H	1.90880	-1.37610	-2.58320
C	3.34340	1.99350	-3.84990
H	1.84140	2.91080	-2.61110
C	3.90300	0.79280	-4.29440
H	3.80880	-1.36250	-4.17820
H	3.74150	2.94360	-4.19550
H	4.74000	0.80210	-4.98710
C	-1.10170	1.48360	-3.42210
C	-0.75940	1.43880	-4.77920
C	-1.80540	2.59540	-2.93480
C	-1.10550	2.48740	-5.63760
H	-0.21030	0.58340	-5.16390
C	-2.15730	3.64170	-3.79130
H	-2.07710	2.65130	-1.88390
C	-1.80620	3.59300	-5.14520
H	-0.82870	2.43940	-6.68710
H	-2.70000	4.49740	-3.39930
H	-2.07770	4.40850	-5.80940
C	-0.31230	-3.44930	-1.09260
Ru	-1.14740	-1.50400	-0.21170
H	-1.82480	-0.28940	0.80810
C	2.36760	1.50890	0.84090
C	1.76120	2.24790	1.85750

C	3.42720	2.04870	0.10910
C	2.21320	3.53560	2.13670
C	3.86510	3.34430	0.39300
C	3.26830	4.10790	1.40790
H	-2.97820	-2.95900	1.71550
H	-1.96870	-3.29640	-2.47030
H	3.90260	1.46500	-0.66940
H	4.68170	3.76630	-0.18700
H	1.72930	4.10720	2.92460
H	0.95090	1.82330	2.43140
C	3.74790	5.50440	1.72160
H	4.34420	5.91640	0.90260
H	4.37170	5.50660	2.62400
H	2.90410	6.17670	1.90870
O	-2.40350	3.95210	2.19170
O	-3.25250	1.36540	0.11520
O	-0.87280	3.23950	0.68820
O	-0.76350	2.04090	3.90660
N	-1.49800	1.12870	3.15920
C	-1.93140	1.61440	2.05670
C	-2.84700	0.77760	1.17130
C	-3.81270	-0.14220	1.92280
H	-4.23660	-0.82320	1.18490
H	-3.27010	-0.73390	2.66390
C	-4.93030	0.67060	2.59490
H	-5.62920	-0.00220	3.10220
H	-4.52620	1.36360	3.34040
H	-5.48920	1.25150	1.85470
C	-1.76060	3.06370	1.65790
C	-0.61210	4.62860	0.30090
H	-1.56200	5.08200	0.00610
H	-0.23190	5.14860	1.18440
C	0.39460	4.61750	-0.82740
H	-0.02150	4.15050	-1.72210
H	0.66130	5.65060	-1.07270
H	1.30320	4.08960	-0.53240
C	-0.21740	1.39300	5.07030
H	-1.02010	0.98430	5.69120
H	0.47700	0.60110	4.77390
H	0.31490	2.17840	5.60850
C	-4.61970	-2.05160	-1.64380
H	-4.03690	-1.94690	-2.56470
H	-4.51950	-1.13230	-1.05820
H	-5.66920	-2.15950	-1.93630
C	-5.12640	-3.54440	0.34300
H	-5.18600	-2.69410	1.02920
H	-4.82300	-4.42570	0.91760
H	-6.13420	-3.72290	-0.04430
C	1.54010	-3.67640	0.63140
H	1.72120	-4.75850	0.63180
H	1.77380	-3.27640	1.61820
H	2.21030	-3.21250	-0.09370
H	-0.58110	-3.16330	2.31420
H	0.42850	-3.54110	-1.87930

99
iTS-4-minor-Z_330
G = -2623.2772781

N	-1.85890	-0.34390	-1.80050

H	-2.41330	-0.85760	-2.48360
H	-2.45020	0.42710	-1.44300
C	0.25680	0.81100	-1.36900
H	-0.26970	1.67490	-0.94540
C	-0.65170	0.20820	-2.46780
H	-0.12840	-0.64380	-2.91030
C	-2.99750	-3.07370	0.12830
C	-2.25660	-3.32830	-1.05790
C	-2.24560	-2.98700	1.33930
C	-0.15160	-3.59680	0.18740
C	-0.85210	-3.22120	1.37440
C	-4.50970	-2.95200	0.14310
H	-4.77440	-2.38190	1.04080
N	0.36790	-0.19940	-0.27940
S	1.77070	-0.51210	0.46390
O	1.45310	-1.07660	1.80800
O	2.72470	-1.34530	-0.33110
C	1.52440	1.33340	-2.03280
C	1.78980	2.70790	-2.01180
C	2.38520	0.49130	-2.74970
C	2.89610	3.23660	-2.68050
H	1.13130	3.36560	-1.45510
C	3.49470	1.01340	-3.41840
H	2.20300	-0.57730	-2.76570
C	3.75510	2.38840	-3.38640
H	3.08940	4.30510	-2.64300
H	4.15690	0.34700	-3.96440
H	4.62000	2.79330	-3.90470
C	-0.99320	1.20830	-3.55610
C	-1.74350	2.35940	-3.26850
C	-0.52210	1.01370	-4.86120
C	-2.00950	3.29890	-4.26800
H	-2.11550	2.52570	-2.26220
C	-0.78690	1.95320	-5.86320
H	0.06650	0.12950	-5.09150
C	-1.53080	3.10060	-5.56830
H	-2.58490	4.18910	-4.02910
H	-0.41070	1.78990	-6.86920
H	-1.73460	3.83390	-6.34350
C	-0.86110	-3.62780	-1.03330
Ru	-1.33990	-1.53470	-0.13060
H	-2.09950	-0.23450	0.69660
C	2.61890	1.05570	0.74500
C	1.93010	2.10570	1.34650
C	3.96230	1.19400	0.39270
C	2.59200	3.30530	1.60070
C	4.61440	2.40000	0.65360
C	3.94380	3.47340	1.26160
H	-2.75910	-2.73130	2.25870
H	-2.76390	-3.31910	-2.01700
H	4.47850	0.37860	-0.10020
H	5.65700	2.51250	0.36710
H	2.04510	4.12450	2.06020
H	0.88320	2.00800	1.58580
C	4.66320	4.76770	1.55740
H	3.96740	5.61140	1.59190
H	5.42900	4.97900	0.80460
H	5.16670	4.71570	2.53100

O	-2.69490	4.12320	1.78140
O	-3.12770	1.57990	-0.31240
O	-0.77620	3.61120	0.68240
O	-0.54980	2.18530	3.40450
N	-1.33670	1.29050	2.68410
C	-1.95100	1.84660	1.71010
C	-2.94380	1.08290	0.84260
C	-4.16670	0.47890	1.54380
H	-4.87330	1.31850	1.60640
H	-4.60070	-0.23030	0.83730
C	-3.98870	-0.14220	2.92840
H	-4.90400	-0.66860	3.21790
H	-3.16070	-0.85180	2.94660
H	-3.78080	0.61960	3.68440
C	-1.85540	3.32550	1.40180
C	-0.53960	5.00950	0.31670
H	-0.85340	5.64300	1.14890
H	0.54440	5.06570	0.20840
C	-1.26180	5.35420	-0.97400
H	-2.34380	5.24700	-0.85750
H	-1.04210	6.39280	-1.24270
H	-0.93050	4.70790	-1.79070
C	0.24810	1.46160	4.36120
H	0.83550	2.22270	4.87660
H	-0.39740	0.93420	5.07010
H	0.90650	0.75680	3.84510
C	-5.12840	-4.35830	0.28740
H	-4.75180	-4.86800	1.18050
H	-4.88850	-4.97580	-0.58600
H	-6.21850	-4.28630	0.36690
C	-5.08460	-2.21950	-1.07810
H	-4.61600	-1.24020	-1.21480
H	-6.16090	-2.06890	-0.94770
H	-4.94370	-2.79690	-1.99810
C	1.29010	-4.00900	0.25280
H	1.80450	-3.82540	-0.69110
H	1.32270	-5.08510	0.46670
H	1.81710	-3.47810	1.04490
H	-0.30740	-3.11690	2.30440
H	-0.34230	-3.85660	-1.95780

99

iTS-4-minor-Z_330-2

N	-1.89220	-0.28640	-1.66470
H	-2.51150	-0.75240	-2.32470
H	-2.45160	0.46880	-1.22960
C	0.33000	0.72700	-1.38550
H	-0.06800	1.63050	-0.90830
C	-0.72430	0.24540	-2.41090
H	-0.29970	-0.60270	-2.95520
C	-3.00250	-3.11140	-0.13320
C	-2.07370	-3.35880	-1.19170
C	-2.46340	-3.03160	1.17500
C	-0.20440	-3.62460	0.39370
C	-1.08760	-3.28280	1.44630
C	-4.49800	-2.98630	-0.37670
H	-4.95530	-2.71460	0.58220
N	0.44250	-0.33420	-0.35260
S	1.80900	-0.56620	0.48210

O	1.44590	-1.05750	1.84260
O	2.81200	-1.42210	-0.21890
C	1.58760	1.11820	-2.14890
C	2.34770	0.16500	-2.83890
C	1.95250	2.46720	-2.23660
C	3.45840	0.55290	-3.59180
H	2.08370	-0.88450	-2.76620
C	3.05920	2.86180	-2.99210
H	1.37690	3.21110	-1.69480
C	3.81870	1.90350	-3.67160
H	4.04370	-0.19820	-4.11540
H	3.33220	3.91230	-3.04170
H	4.68420	2.20470	-4.25540
C	-1.10940	1.33420	-3.39520
C	-0.65620	1.27770	-4.71970
C	-1.88110	2.43240	-2.98680
C	-0.95940	2.30300	-5.62110
H	-0.05220	0.43410	-5.04300
C	-2.18840	3.45670	-3.88640
H	-2.24050	2.49300	-1.96370
C	-1.72570	3.39740	-5.20590
H	-0.59690	2.24730	-6.64370
H	-2.78300	4.30320	-3.55440
H	-1.96180	4.19560	-5.90390
C	-0.70630	-3.63910	-0.94080
Ru	-1.30350	-1.57290	-0.09710
H	-1.91960	-0.26560	0.84970
C	2.60500	1.03590	0.69540
C	3.95000	1.19950	0.36170
C	1.87270	2.08860	1.24050
C	4.56010	2.43600	0.58120
C	2.49530	3.31390	1.46330
C	3.84510	3.51220	1.13080
H	-3.12360	-2.78540	1.99760
H	-2.42000	-3.34420	-2.22080
H	0.82640	1.96200	1.47400
H	1.92280	4.12570	1.90220
H	5.60620	2.56700	0.31600
H	4.50230	0.37670	-0.07720
C	4.49670	4.86010	1.32670
H	4.32410	5.49930	0.45150
H	5.57880	4.76660	1.45770
H	4.08340	5.37950	2.19670
O	-2.89970	4.09630	1.45390
O	-3.42040	1.27140	-0.00440
O	-1.09960	3.47570	0.21870
O	-0.55870	2.74100	3.21500
N	-1.27430	1.63270	2.76900
C	-1.98290	1.89920	1.73700
C	-2.93870	0.88950	1.11110
C	-3.86560	0.10240	2.04770
H	-4.67410	0.80890	2.28390
H	-4.31230	-0.67760	1.42710
C	-3.29590	-0.47640	3.34500
H	-4.01570	-1.17830	3.77960
H	-2.35630	-1.00720	3.17100
H	-3.09750	0.30550	4.08110
C	-2.05040	3.28580	1.12830

C	-1.07300	4.75820	-0.49060
H	-0.63290	4.51000	-1.45630
H	-2.10260	5.08940	-0.64030
C	-0.24470	5.77850	0.26870
H	0.78610	5.43350	0.37980
H	-0.23600	6.71970	-0.29090
H	-0.66640	5.96830	1.25960
C	0.26560	2.37050	4.33490
H	0.79000	3.28480	4.61590
H	-0.35480	2.01660	5.16360
H	0.98490	1.60140	4.03910
C	-5.06230	-4.35970	-0.79260
H	-4.82400	-5.12910	-0.05070
H	-4.64580	-4.67430	-1.75650
H	-6.15130	-4.30570	-0.89520
C	-4.87160	-1.89850	-1.39530
H	-4.44940	-2.11180	-2.38420
H	-4.52600	-0.91500	-1.06450
H	-5.95970	-1.85560	-1.51070
C	1.21590	-4.02220	0.67490
H	1.88360	-3.71720	-0.13170
H	1.25120	-5.11510	0.76700
H	1.57290	-3.57590	1.60310
H	-0.71600	-3.21220	2.46160
H	-0.03590	-3.84420	-1.76810

99
iTS-3-minor-E_0
G = -2623.2829855

N	2.08430	-0.27800	1.92420
H	2.85700	-0.66570	2.46350
H	2.48530	0.46280	1.31950
C	-0.19860	0.64950	2.07180
H	0.03220	1.51180	1.43670
C	1.08380	0.29010	2.86410
H	0.82760	-0.51400	3.55990
C	2.88160	-3.19150	0.19620
C	2.38010	-3.38530	1.51660
C	1.92120	-3.21570	-0.85690
C	0.09390	-3.78550	0.69700
C	0.55270	-3.51310	-0.62190
C	4.36300	-3.01320	-0.08560
H	4.45110	-2.64870	-1.11500
N	-0.50120	-0.51250	1.19930
S	-2.00030	-0.75210	0.62180
O	-1.88910	-1.49940	-0.65970
O	-2.91390	-1.37460	1.62530
C	-1.24510	1.08100	3.08970
C	-1.55480	2.43920	3.21880
C	-1.83210	0.16480	3.97100
C	-2.44440	2.88080	4.20080
H	-1.10080	3.15630	2.54210
C	-2.72970	0.59830	4.94920
H	-1.60570	-0.89090	3.87100
C	-3.03960	1.95870	5.06740
H	-2.67730	3.93880	4.28300
H	-3.18760	-0.12400	5.61960
H	-3.73810	2.29540	5.82850
C	1.62810	1.47550	3.63920

C	1.57950	1.48650	5.03870
C	2.14770	2.59150	2.96470
C	2.03500	2.59600	5.75860
H	1.17070	0.62910	5.56720
C	2.60530	3.69950	3.68220
H	2.19300	2.59350	1.87930
C	2.54810	3.70680	5.08100
H	1.98750	2.59240	6.84400
H	3.00270	4.55900	3.14910
H	2.90170	4.57030	5.63740
C	1.01940	-3.71110	1.77130
Ru	1.21270	-1.70690	0.63500
H	1.60690	-0.47710	-0.50170
C	-2.77670	0.82820	0.21200
C	-2.47230	1.43500	-1.00910
C	-3.72360	1.39220	1.06840
C	-3.11410	2.61890	-1.36530
C	-4.35430	2.58300	0.70070
C	-4.05750	3.21800	-0.51410
H	2.24090	-2.99000	-1.86850
H	3.05400	-3.28880	2.36220
H	-3.95720	0.91170	2.01100
H	-5.08320	3.02600	1.37420
H	-2.86920	3.09140	-2.31310
H	-1.74730	0.98770	-1.67700
C	-4.69600	4.53350	-0.88640
H	-5.58260	4.73730	-0.27940
H	-4.98470	4.55160	-1.94240
H	-3.98670	5.35640	-0.73040
O	0.99390	3.64660	-2.37100
O	2.91670	1.37620	-0.11890
O	0.59250	3.05250	-0.22470
O	0.61750	-0.50650	-2.88290
N	0.36610	0.81550	-2.60090
C	1.17260	1.33540	-1.74490
C	2.39360	0.71120	-1.06940
C	3.34600	-0.05490	-2.00030
H	4.13820	-0.44570	-1.36040
H	2.84250	-0.88760	-2.48640
C	3.94600	0.88670	-3.05830
H	4.67660	0.34470	-3.66760
H	3.17320	1.27670	-3.72970
H	4.45430	1.73660	-2.59100
C	0.90490	2.80240	-1.49320
C	0.46680	4.45020	0.19120
H	0.72100	4.42480	1.25170
H	1.21540	5.03630	-0.34640
C	-0.93900	4.97480	-0.03590
H	-1.67370	4.36760	0.49800
H	-0.99910	6.00300	0.33640
H	-1.19440	4.97240	-1.09820
C	-0.45560	-1.05700	-3.66980
H	-0.60750	-0.46270	-4.57550
H	-0.12540	-2.06440	-3.92850
H	-1.36760	-1.10400	-3.06960
C	5.06100	-4.38720	-0.01350
H	4.59580	-5.10520	-0.69710
H	5.00420	-4.79660	1.00200

H	6.11810	-4.29040	-0.28330
C	5.04870	-1.99580	0.84000
H	5.02860	-2.32180	1.88580
H	4.56990	-1.01450	0.77610
H	6.09910	-1.88080	0.55360
C	-1.33290	-4.17680	0.95750
H	-1.98270	-3.85910	0.14310
H	-1.70250	-3.73520	1.88500
H	-1.38100	-5.26880	1.05070
H	-0.15360	-3.49400	-1.44190
H	0.68280	-3.86920	2.79010

99
iTS-3-minor-E_0-3
G = -2623.2819846

N	2.04640	-0.36300	1.98920
H	2.75110	-0.79810	2.58250
H	2.53000	0.35530	1.42270
C	-0.15560	0.70200	1.92190
H	0.22990	1.48550	1.25990
C	1.00970	0.27100	2.84540
H	0.64150	-0.51130	3.51460
C	2.86800	-3.17590	0.07240
C	2.42940	-3.40240	1.40700
C	1.86070	-3.20030	-0.93970
C	0.12710	-3.87870	0.68340
C	0.51460	-3.53410	-0.64690
C	4.33090	-2.95710	-0.26860
H	4.36500	-2.55900	-1.28850
N	-0.49250	-0.45490	1.04530
S	-2.01540	-0.95320	0.81070
O	-2.03950	-1.72980	-0.46050
O	-2.61610	-1.66850	1.97670
C	-1.24480	1.34100	2.77350
C	-1.85730	0.65380	3.83060
C	-1.57650	2.68490	2.55810
C	-2.79310	1.29390	4.64550
H	-1.62140	-0.38980	4.00410
C	-2.51000	3.33000	3.37350
H	-1.10830	3.22030	1.73780
C	-3.12340	2.63510	4.42050
H	-3.26470	0.74570	5.45670
H	-2.76140	4.37060	3.18690
H	-3.85280	3.13210	5.05430
C	1.56160	1.42170	3.66620
C	2.17530	2.52050	3.04330
C	1.42920	1.41840	5.06100
C	2.64140	3.59530	3.80460
H	2.28990	2.53730	1.96360
C	1.89490	2.49380	5.82480
H	0.94800	0.57600	5.55090
C	2.50180	3.58680	5.19740
H	3.10980	4.44130	3.30950
H	1.78190	2.47790	6.90520
H	2.86260	4.42450	5.78760
C	1.09100	-3.78680	1.71390
Ru	1.20000	-1.73470	0.61280
H	1.64820	-0.49620	-0.46790
C	-3.05930	0.49090	0.52250

C	-4.22050	0.66800	1.27600
C	-2.72110	1.38610	-0.49280
C	-5.05020	1.75680	1.00430
C	-3.55860	2.47110	-0.74970
C	-4.73500	2.67440	-0.01010
H	2.12910	-2.94050	-1.95840
H	3.13720	-3.30210	2.22360
H	-1.80400	1.26330	-1.05550
H	-3.28430	3.17870	-1.52820
H	-5.94780	1.90250	1.60010
H	-4.45420	-0.02370	2.07670
C	-5.64650	3.84080	-0.30990
H	-6.39640	3.56220	-1.06110
H	-5.08600	4.69260	-0.70720
H	-6.18510	4.16660	0.58530
O	-0.06830	3.29010	-0.52960
O	2.98640	1.32210	-0.00410
O	1.97350	3.64750	-1.43920
O	0.41350	-0.30490	-2.66260
N	0.21610	0.98740	-2.24770
C	1.15600	1.44660	-1.49980
C	2.42010	0.76320	-0.99390
C	3.28730	0.06100	-2.04050
H	4.11420	-0.40110	-1.50000
H	2.72330	-0.71610	-2.55490
C	3.83150	1.07840	-3.05900
H	4.39910	1.87150	-2.56220
H	4.49470	0.57420	-3.76920
H	3.02000	1.54470	-3.62810
C	0.93640	2.88400	-1.08640
C	1.94000	5.05570	-1.04780
H	2.67020	5.52330	-1.70950
H	0.94570	5.45370	-1.26440
C	2.31260	5.22320	0.41640
H	3.29040	4.77680	0.62030
H	2.36160	6.29050	0.65630
H	1.56880	4.75660	1.06600
C	-0.74740	-0.78020	-3.37280
H	-0.97890	-0.11440	-4.20930
H	-0.46380	-1.76710	-3.74190
H	-1.59130	-0.86640	-2.68520
C	5.06010	-4.31660	-0.27370
H	4.58130	-5.02170	-0.96130
H	5.05600	-4.76080	0.72860
H	6.10220	-4.18660	-0.58500
C	5.03530	-1.95460	0.65920
H	5.09150	-2.32740	1.68780
H	4.51670	-0.99180	0.67040
H	6.06140	-1.78720	0.31560
C	-1.25560	-4.38360	0.98280
H	-1.60160	-4.04080	1.95930
H	-1.22210	-5.48050	0.98870
H	-1.96880	-4.05290	0.22940
H	-0.23050	-3.51760	-1.43200
H	0.80910	-3.98940	2.74140

99
iTS-3-minor-E_330-2
G = -2623.2817366

N	2.11330	-0.33590	1.91380
H	2.83360	-0.76700	2.49090
H	2.57630	0.39920	1.34590
C	-0.10970	0.68600	1.89250
H	0.24940	1.48500	1.23470
C	1.07790	0.26800	2.79280
H	0.73560	-0.52860	3.45900
C	2.96290	-3.09130	-0.08450
C	2.56980	-3.34570	1.25790
C	1.92710	-3.12490	-1.06810
C	0.25720	-3.86530	0.59250
C	0.59910	-3.49710	-0.74340
C	4.40890	-2.83060	-0.46450
H	4.40160	-2.39980	-1.47130
N	-0.43560	-0.46630	1.00400
S	-1.95170	-1.00410	0.80030
O	-1.98300	-1.77160	-0.47590
O	-2.50560	-1.74270	1.97450
C	-1.19900	1.29250	2.76760
C	-1.56520	2.63000	2.56880
C	-1.77670	0.58360	3.83000
C	-2.50000	3.24670	3.40440
H	-1.12300	3.18270	1.74580
C	-2.71340	1.19550	4.66530
H	-1.51340	-0.45520	3.99200
C	-3.07920	2.52990	4.45610
H	-2.77840	4.28250	3.23010
H	-3.15790	0.63030	5.48010
H	-3.80950	3.00490	5.10550
C	1.61810	1.42110	3.61810
C	2.19510	2.54120	2.99820
C	1.50810	1.39910	5.01470
C	2.64620	3.61890	3.76430
H	2.29260	2.57110	1.91720
C	1.95910	2.47740	5.78320
H	1.05500	0.54000	5.50230
C	2.52850	3.59200	5.15880
H	3.08550	4.48180	3.27180
H	1.86350	2.44700	6.86500
H	2.87700	4.43250	5.75250
C	1.24710	-3.75680	1.59740
Ru	1.27460	-1.69960	0.52770
H	1.69630	-0.40640	-0.54520
C	-3.03710	0.41530	0.54670
C	-2.74520	1.32450	-0.47050
C	-4.18320	0.55940	1.32970
C	-3.61450	2.39050	-0.69950
C	-5.04440	1.63030	1.08650
C	-4.77620	2.56160	0.07100
H	2.15980	-2.84350	-2.08980
H	3.29760	-3.23950	2.05580
H	-4.38050	-0.14260	2.13110
H	-5.92970	1.75130	1.70570
H	-3.37710	3.10890	-1.48020
H	-1.83960	1.22710	-1.05640
C	-5.72160	3.70850	-0.19730
H	-6.48610	3.41670	-0.92850
H	-5.19150	4.57440	-0.60570

H	-6.24190	4.01810	0.71440
O	-0.12540	3.31990	-0.49680
O	2.97880	1.39440	-0.04910
O	1.89950	3.73190	-1.41970
O	0.39190	-0.22410	-2.70210
N	0.17500	1.05790	-2.25790
C	1.11790	1.51600	-1.51360
C	2.39480	0.82980	-1.03600
C	3.26480	0.19350	-2.12800
H	4.12060	-0.25120	-1.61830
H	2.71840	-0.59240	-2.64880
C	3.74960	1.25030	-3.13520
H	4.29900	2.05400	-2.63490
H	4.41520	0.78560	-3.87010
H	2.91020	1.69800	-3.67810
C	0.88090	2.94210	-1.07140
C	1.84290	5.13210	-1.00190
H	2.55260	5.62720	-1.66590
H	0.83730	5.51320	-1.19500
C	2.23530	5.28130	0.45910
H	3.22510	4.85170	0.63980
H	2.26640	6.34480	0.71770
H	1.51150	4.78810	1.11160
C	-0.77480	-0.71340	-3.39210
H	-1.59240	-0.85370	-2.68170
H	-1.05680	-0.02630	-4.19530
H	-0.47090	-1.67590	-3.80700
C	5.16400	-4.17390	-0.53730
H	4.67750	-4.86590	-1.23290
H	5.20060	-4.65040	0.44930
H	6.19260	-4.01180	-0.87690
C	5.12160	-1.84360	0.47330
H	4.57980	-0.89570	0.53770
H	6.12970	-1.63740	0.09930
H	5.22480	-2.25100	1.48490
C	-1.10180	-4.41040	0.92540
H	-1.42590	-4.09270	1.91780
H	-1.03850	-5.50600	0.91260
H	-1.84650	-4.08810	0.19940
H	-0.16810	-3.48830	-1.50690
H	0.99810	-3.97550	2.63010

99
iTS-3-minor-E_60-2
G = -2623.2813324

N	2.10970	-0.16180	1.68970
H	3.00360	-0.52930	2.00870
H	2.32200	0.64050	1.05850
C	-0.09540	0.62490	2.39630
H	-0.04410	1.43230	1.65210
C	1.34440	0.31550	2.86770
H	1.28630	-0.52680	3.56280
C	2.51440	-3.20800	0.94070
C	1.25010	-3.60050	1.47990
C	2.58270	-2.86580	-0.43810
C	0.15430	-3.31850	-0.73300
C	1.41010	-2.88700	-1.23930
C	3.73200	-3.18970	1.85060
H	3.41280	-2.73170	2.79630

N	-0.56230	-0.62860	1.74130
S	-2.15850	-0.76700	1.42120
O	-2.37890	-2.07710	0.76310
O	-2.98480	-0.52260	2.63340
C	-0.87980	1.13260	3.59860
C	-1.31930	2.45820	3.62370
C	-1.07890	0.33090	4.73130
C	-1.96140	2.97940	4.75340
H	-1.15390	3.09190	2.75800
C	-1.71590	0.84290	5.86090
H	-0.75050	-0.70400	4.71980
C	-2.16260	2.17180	5.87560
H	-2.29890	4.01220	4.75410
H	-1.86740	0.20870	6.73020
H	-2.65940	2.57120	6.75540
C	2.02020	1.48410	3.56010
C	2.37350	1.38480	4.91150
C	2.27360	2.68210	2.87480
C	2.96350	2.46610	5.57450
H	2.17520	0.46260	5.45140
C	2.86310	3.76300	3.53440
H	2.01150	2.76620	1.82550
C	3.20770	3.65950	4.88720
H	3.22870	2.37680	6.62430
H	3.04930	4.68700	2.99370
H	3.66340	4.50150	5.40080
C	0.09970	-3.63920	0.65210
Ru	1.00910	-1.54920	0.52480
H	1.05220	-0.17100	-0.57250
C	-2.69280	0.48220	0.23670
C	-3.27580	1.66580	0.69450
C	-2.54440	0.24100	-1.13060
C	-3.71120	2.61420	-0.23140
C	-2.98760	1.19800	-2.04220
C	-3.56580	2.40160	-1.61020
H	3.51210	-2.53050	-0.87780
H	1.16290	-3.83230	2.53570
H	-2.09320	-0.68280	-1.47540
H	-2.86480	1.01380	-3.10500
H	-4.16200	3.53710	0.12450
H	-3.39450	1.83550	1.75860
C	-3.97450	3.45890	-2.60720
H	-4.36000	3.01240	-3.52920
H	-3.10860	4.07580	-2.87970
H	-4.73840	4.12510	-2.19580
O	0.15030	3.76620	-2.52660
O	2.20050	1.74110	-0.24760
O	-0.37510	2.96840	-0.47530
O	0.41430	-0.35090	-3.29950
N	0.02400	0.93980	-3.00960
C	0.61800	1.46570	-2.00030
C	1.79010	0.94630	-1.16700
C	2.90060	0.21740	-1.94880
H	3.62300	-0.11030	-1.19620
H	2.52220	-0.65670	-2.47140
C	3.57360	1.17270	-2.94670
H	4.41040	0.66990	-3.44250
H	2.87010	1.49600	-3.72190

```
H          3.95990        2.06380       -2.44170
C          0.11450        2.86120       -1.70660
C         -0.83820        4.28710       -0.05150
H         -1.38080        4.74830       -0.87910
H         -1.53980        4.06860        0.75180
C          0.31970        5.14730        0.42560
H          1.04230        5.31610       -0.37720
H         -0.06720        6.11810        0.75320
H          0.82900        4.67790        1.27050
C         -0.30370       -0.82420       -4.45350
H         -0.09690       -0.18870       -5.31940
H          0.07210       -1.83330       -4.62900
H         -1.37830       -0.85140       -4.24940
C          4.92720       -2.39800        1.29940
H          4.65840       -1.38330        0.98880
H          5.37000       -2.90200        0.43350
H          5.70060       -2.32180        2.06920
C          4.14840       -4.64480        2.15840
H          4.45200       -5.15870        1.23940
H          3.33030       -5.21040        2.61450
H          4.99680       -4.65060        2.85040
C         -1.03470       -3.46010       -1.64360
H         -1.96410       -3.29820       -1.09960
H         -1.04790       -4.46920       -2.07400
H         -0.97850       -2.74680       -2.46900
H          1.46980       -2.55730       -2.27050
H         -0.86150       -3.86690        1.09490
99
iTS-3-minor-E_0-4
G = -2623.2781298
N          1.92850       -0.32290        1.99750
H          2.43500       -0.84960        2.70720
H          2.61420        0.29200        1.51770
C         -0.02170        1.06360        1.53780
H          0.59660        1.68440        0.87670
C          0.88600        0.50940        2.65540
H          0.28700       -0.17470        3.26280
C          2.58750       -3.28590        0.17730
C          1.96420       -3.38810        1.45000
C          1.71640       -3.19070       -0.95170
C         -0.28400       -3.49760        0.45710
C          0.31140       -3.28170       -0.82330
C          4.09370       -3.33570        0.00420
H          4.31840       -2.88700       -0.96980
N         -0.49190       -0.13750        0.78970
S         -1.66900        0.12560       -0.30740
O         -1.56300        1.43850       -0.99950
O         -1.77540       -1.06700       -1.18660
C         -1.10050        1.96180        2.13100
C         -1.26030        3.26220        1.63890
C         -1.91800        1.53610        3.18670
C         -2.21520        4.12450        2.18640
H         -0.63970        3.59190        0.81310
C         -2.87480        2.39060        3.73670
H         -1.82330        0.52680        3.57310
C         -3.02670        3.69010        3.23900
H         -2.32550        5.12990        1.78910
H         -3.50630        2.04040        4.54850
```

H	-3.77310	4.35460	3.66580
C	1.47810	1.57890	3.55440
C	1.21480	1.55680	4.93050
C	2.27720	2.60730	3.03210
C	1.73510	2.54340	5.77440
H	0.58980	0.76900	5.34330
C	2.79710	3.59420	3.87300
H	2.49540	2.63400	1.96950
C	2.52810	3.56690	5.24620
H	1.51860	2.51320	6.83870
H	3.40840	4.38880	3.45410
H	2.93220	4.33690	5.89750
C	0.55220	-3.53430	1.59500
Ru	1.11770	-1.56970	0.49070
H	1.89130	-0.39880	-0.53510
C	-3.23180	0.17820	0.60350
C	-4.18700	1.14280	0.28390
C	-3.49130	-0.77620	1.58950
C	-5.41230	1.14440	0.95670
C	-4.71880	-0.76820	2.24870
C	-5.70040	0.19000	1.94320
H	2.14550	-3.03960	-1.93560
H	2.57510	-3.37610	2.34660
H	-2.72560	-1.48950	1.86650
H	-4.91070	-1.50440	3.02530
H	-6.14950	1.90640	0.71620
H	-3.96640	1.89500	-0.46540
C	-7.03520	0.18200	2.64920
H	-6.94070	-0.18510	3.67590
H	-7.74290	-0.47670	2.13000
H	-7.47780	1.18230	2.67770
O	3.37910	3.66090	-1.47190
O	3.35620	1.19440	0.27890
O	1.19720	3.80740	-0.88230
O	0.85350	0.18620	-2.75140
N	1.07920	1.49680	-2.44240
C	2.01200	1.69290	-1.58210
C	2.94890	0.74190	-0.83970
C	3.98140	-0.03860	-1.66390
H	4.78120	0.70280	-1.80330
H	4.39040	-0.80030	-0.99950
C	3.61250	-0.62570	-3.02370
H	4.49300	-1.11480	-3.45340
H	2.81000	-1.35890	-2.94860
H	3.28230	0.15110	-3.71920
C	2.28180	3.15340	-1.30500
C	1.32860	5.23890	-0.60920
H	1.94200	5.68800	-1.39350
H	0.30570	5.61020	-0.69010
C	1.90670	5.47570	0.77610
H	2.92210	5.07780	0.84920
H	1.94010	6.55220	0.97370
H	1.28780	5.00100	1.54160
C	-0.18360	0.08560	-3.74250
H	-1.10220	0.53700	-3.36410
H	0.13840	0.56020	-4.67500
H	-0.32690	-0.98500	-3.89230
C	4.54820	-4.80940	-0.04990

H	4.03120	-5.35550	-0.84600
H	4.33870	-5.31200	0.90170
H	5.62590	-4.86700	-0.23740
C	4.86560	-2.55940	1.08190
H	4.76850	-3.03080	2.06580
H	4.51170	-1.52700	1.16070
H	5.93140	-2.53900	0.83300
C	-1.76380	-3.71920	0.57670
H	-2.09760	-3.65260	1.61440
H	-1.99490	-4.72760	0.21170
H	-2.31200	-3.00190	-0.03460
H	-0.32160	-3.17260	-1.69450
H	0.12090	-3.64530	2.58390

99
iTS-3-minor-E_60-3
G = -2623.2769312

N	2.27210	-0.14170	1.54690
H	3.08390	-0.49670	2.04760
H	2.62610	0.58670	0.90350
C	-0.00860	0.73670	1.80660
H	0.17830	1.57050	1.12150
C	1.32020	0.42770	2.53410
H	1.13210	-0.36290	3.26570
C	2.50170	-3.31040	1.40860
C	1.11280	-3.59400	1.50990
C	3.05700	-3.09900	0.10750
C	0.84020	-3.46840	-0.95960
C	2.23170	-3.15330	-1.04270
C	3.34000	-3.25760	2.67920
H	2.79030	-2.63120	3.39400
N	-0.34580	-0.44390	0.96010
S	-1.85680	-1.00520	0.84370
O	-1.98040	-1.75030	-0.44060
O	-2.32900	-1.78920	2.02650
C	-1.01260	1.21950	2.84610
C	-1.40580	0.41470	3.92420
C	-1.48490	2.53510	2.77900
C	-2.26570	0.91260	4.90540
H	-1.06040	-0.61100	3.98460
C	-2.34130	3.04050	3.75950
H	-1.19480	3.16170	1.94270
C	-2.73660	2.22820	4.82710
H	-2.56810	0.27410	5.73100
H	-2.70460	4.06160	3.68440
H	-3.40690	2.61520	5.58960
C	1.87970	1.64810	3.24040
C	2.30850	2.76600	2.50710
C	1.93620	1.69020	4.63950
C	2.77780	3.90600	3.16440
H	2.27500	2.74750	1.42190
C	2.40450	2.83110	5.29970
H	1.59990	0.83190	5.21510
C	2.82490	3.94340	4.56310
H	3.10220	4.76590	2.58490
H	2.43890	2.85070	6.38540
H	3.18700	4.83130	5.07360
C	0.30250	-3.64300	0.34030
Ru	1.35900	-1.62700	0.34680

H	1.58270	-0.44510	-0.85890
C	-2.94310	0.42690	0.70220
C	-4.08680	0.51750	1.49490
C	-2.64150	1.41620	-0.23550
C	-4.93520	1.61610	1.34330
C	-3.50080	2.50400	-0.37840
C	-4.65860	2.62460	0.40750
H	4.10300	-2.84780	-0.01210
H	0.65550	-3.71970	2.48500
H	-1.73560	1.35960	-0.82710
H	-3.26080	3.27700	-1.10330
H	-5.81790	1.69610	1.97260
H	-4.29220	-0.24650	2.23590
C	-5.58810	3.80230	0.23620
H	-6.33980	3.59220	-0.53520
H	-5.04140	4.69770	-0.07500
H	-6.12340	4.02640	1.16390
O	1.83720	3.86470	-2.16580
O	3.03490	1.41900	-0.56840
O	0.44240	3.35040	-0.45000
O	-0.07690	-0.06320	-2.80100
N	0.00890	1.25010	-2.42360
C	1.12080	1.59330	-1.88310
C	2.39460	0.84790	-1.50210
C	3.23450	0.08040	-2.52830
H	3.93750	0.84600	-2.88660
H	3.83060	-0.63500	-1.95360
C	2.57640	-0.58900	-3.73140
H	3.35400	-1.02970	-4.36390
H	1.88380	-1.37490	-3.43190
H	2.01510	0.13090	-4.33360
C	1.17790	3.06440	-1.52410
C	0.41520	4.73010	0.03870
H	0.47330	4.63060	1.12390
H	1.30400	5.24960	-0.32260
C	-0.86950	5.40550	-0.40560
H	-1.74180	4.86080	-0.03400
H	-0.89970	6.42460	-0.00620
H	-0.92520	5.45820	-1.49710
C	-1.37820	-0.33510	-3.35590
H	-1.60950	0.38290	-4.14760
H	-1.30300	-1.34170	-3.76790
H	-2.13450	-0.31590	-2.56860
C	4.74600	-2.66790	2.49510
H	4.73950	-1.68150	2.02070
H	5.37340	-3.32450	1.88220
H	5.22830	-2.56470	3.47150
C	3.43880	-4.67670	3.28040
H	3.95600	-5.35070	2.58790
H	2.45110	-5.09580	3.49320
H	4.00650	-4.64700	4.21620
C	-0.00830	-3.62720	-2.19130
H	-1.02770	-3.29170	-1.99630
H	-0.03570	-4.68220	-2.49050
H	0.39770	-3.05310	-3.02740
H	2.67400	-2.95180	-2.01180
H	-0.76580	-3.79010	0.44500

99

iTS-3-minor-E_330
G = -2623.2761901

N	2.15180	-0.31700	1.93650
H	2.86700	-0.74380	2.52330
H	2.61800	0.42430	1.38380
C	-0.09530	0.67040	1.90870
H	0.24100	1.48990	1.26540
C	1.09810	0.26890	2.80680
H	0.76910	-0.53560	3.47030
C	2.99680	-3.14120	-0.07520
C	2.66230	-3.31490	1.29400
C	1.91830	-3.22910	-1.01170
C	0.31750	-3.83850	0.76590
C	0.60510	-3.55010	-0.60680
C	4.42240	-2.93120	-0.54790
H	4.36650	-2.40210	-1.50520
N	-0.38970	-0.48030	1.00750
S	-1.90820	-0.95230	0.71420
O	-1.90470	-1.74270	-0.54860
O	-2.57840	-1.64030	1.85760
C	-1.20370	1.22380	2.79780
C	-1.75980	0.47520	3.84410
C	-1.62060	2.54910	2.62590
C	-2.71790	1.03800	4.69010
H	-1.46140	-0.55720	3.98630
C	-2.57550	3.11980	3.47020
H	-1.20800	3.12980	1.80940
C	-3.12940	2.36350	4.50760
H	-3.14320	0.44190	5.49300
H	-2.89050	4.14750	3.31160
H	-3.87550	2.79990	5.16590
C	1.61160	1.43070	3.63780
C	1.55670	1.37410	5.03640
C	2.11090	2.59040	3.02240
C	1.98670	2.45580	5.81240
H	1.16340	0.48410	5.52090
C	2.54080	3.67140	3.79570
H	2.16410	2.64770	1.93960
C	2.47840	3.60930	5.19300
H	1.93430	2.39800	6.89610
H	2.92060	4.56450	3.30690
H	2.81020	4.45230	5.79260
C	1.35280	-3.70260	1.71350
Ru	1.35420	-1.69330	0.53790
H	1.81750	-0.45480	-0.56180
C	-2.89430	0.52600	0.39070
C	-2.43060	1.47210	-0.52340
C	-4.12160	0.69660	1.03180
C	-3.20560	2.59760	-0.79530
C	-4.88700	1.83010	0.75090
C	-4.44380	2.79790	-0.16360
H	2.11150	-3.03160	-2.06010
H	3.42470	-3.17350	2.05270
H	-4.45670	-0.03360	1.75930
H	-5.83590	1.97020	1.26240
H	-2.83490	3.33810	-1.49910
H	-1.46610	1.35210	-0.99920
C	-5.27770	4.01820	-0.47370

H	-5.95430	4.26030	0.35120
H	-5.89220	3.85050	-1.36720
H	-4.64550	4.88960	-0.67170
O	1.95230	3.90190	-1.89940
O	3.03890	1.45560	0.04830
O	0.56170	3.35840	-0.19230
O	0.41310	-0.12370	-2.63040
N	0.37200	1.20610	-2.29350
C	1.35360	1.60630	-1.57290
C	2.58320	0.90890	-1.00410
C	3.62620	0.34090	-1.97260
H	4.20840	1.23430	-2.24130
H	4.29180	-0.27690	-1.36810
C	3.20680	-0.37660	-3.25220
H	2.60710	-1.26220	-3.04460
H	2.61780	0.27460	-3.90340
H	4.10460	-0.68360	-3.79890
C	1.31910	3.08530	-1.25160
C	0.51360	4.74040	0.29120
H	0.47040	4.63820	1.37670
H	1.44290	5.24040	0.01330
C	-0.70920	5.44620	-0.26690
H	-1.62520	4.92160	0.01800
H	-0.75240	6.46470	0.13250
H	-0.66210	5.50280	-1.35850
C	-0.76250	-0.48320	-3.37840
H	-1.64780	-0.42820	-2.74110
H	-0.86530	0.15870	-4.25850
H	-0.59330	-1.51730	-3.68260
C	5.07370	-4.30430	-0.81590
H	4.49220	-4.88570	-1.53920
H	5.14450	-4.88420	0.11180
H	6.08500	-4.17220	-1.21530
C	5.27680	-2.09580	0.41710
H	5.48460	-2.63990	1.34470
H	4.78340	-1.15420	0.67710
H	6.24000	-1.86250	-0.04760
C	-1.03920	-4.33230	1.17910
H	-1.28660	-4.02020	2.19500
H	-1.02520	-5.42930	1.14670
H	-1.81410	-3.97090	0.50470
H	-0.19750	-3.56730	-1.33280
H	1.14930	-3.86240	2.76690

99
iTS-3-minor-E_0-2
G = -2623.2753524

N	2.05970	-0.34830	2.06030
H	2.72540	-0.80720	2.67980
H	2.56470	0.39940	1.55420
C	-0.09110	0.76930	1.85700
H	0.40680	1.51790	1.23200
C	0.96680	0.26230	2.86500
H	0.52380	-0.54680	3.45240
C	2.92830	-3.08400	-0.10190
C	2.63140	-3.29990	1.26840
C	1.82710	-3.15650	-1.01820
C	0.27390	-3.81760	0.78980
C	0.52890	-3.49170	-0.58460

C	4.33860	-2.84160	-0.60410
H	4.25660	-2.21900	-1.50010
N	-0.41810	-0.35390	0.92480
S	-1.92860	-0.92480	0.79790
O	-1.97460	-1.79590	-0.41010
O	-2.46380	-1.56120	2.03960
C	-1.21110	1.49290	2.59510
C	-1.53420	2.80360	2.21970
C	-1.87070	0.92630	3.69530
C	-2.50320	3.53120	2.91570
H	-1.03350	3.24910	1.36560
C	-2.83930	1.64960	4.39410
H	-1.64550	-0.08980	3.99650
C	-3.16070	2.95530	4.00710
H	-2.74560	4.54310	2.60280
H	-3.34420	1.19280	5.24110
H	-3.91640	3.51640	4.54990
C	1.48190	1.34080	3.80010
C	1.36600	1.17980	5.18710
C	2.06290	2.51750	3.29890
C	1.81630	2.17370	6.06270
H	0.91090	0.27610	5.58470
C	2.51080	3.51220	4.17150
H	2.16360	2.65670	2.22750
C	2.38880	3.34490	5.55630
H	1.71640	2.03410	7.13560
H	2.95200	4.42010	3.76910
H	2.73630	4.12040	6.23320
C	1.33140	-3.69880	1.71120
Ru	1.30640	-1.66130	0.57860
H	1.83260	-0.41100	-0.45840
C	-3.03480	0.45420	0.42410
C	-2.73140	1.30370	-0.64090
C	-4.21220	0.61600	1.15460
C	-3.62170	2.32410	-0.97250
C	-5.09460	1.64140	0.80890
C	-4.81680	2.50940	-0.25820
H	1.98870	-2.92010	-2.06400
H	3.41180	-3.17430	2.01090
H	-4.42010	-0.03640	1.99430
H	-6.00510	1.77630	1.38730
H	-3.37410	2.99790	-1.78900
H	-1.79860	1.20010	-1.18170
C	-5.78420	3.60540	-0.63860
H	-6.36690	3.94090	0.22490
H	-6.49420	3.24950	-1.39590
H	-5.26130	4.46900	-1.06110
O	-0.20680	3.43420	-0.90510
O	2.80930	1.55810	0.25010
O	1.98980	3.78500	-1.32000
O	0.54810	-0.29350	-2.53180
N	0.31520	1.01160	-2.22290
C	1.21570	1.56810	-1.48930
C	2.48280	1.00880	-0.84560
C	3.63760	0.56210	-1.74910
H	4.16860	1.50710	-1.93310
H	4.30330	-0.03780	-1.12580
C	3.36350	-0.11570	-3.08940

H	4.31450	-0.29010	-3.60340
H	2.85520	-1.07220	-2.96870
H	2.73940	0.50990	-3.73420
C	0.90180	3.01730	-1.19460
C	1.87500	5.18520	-0.92050
H	2.71720	5.66750	-1.41850
H	0.94000	5.58880	-1.31590
C	1.95870	5.31960	0.59240
H	2.89430	4.89500	0.96790
H	1.92440	6.37970	0.86420
H	1.12220	4.81070	1.07810
C	-0.56250	-0.84320	-3.26740
H	-0.77560	-0.22930	-4.14720
H	-0.22950	-1.83690	-3.57090
H	-1.43440	-0.92590	-2.61530
C	4.96040	-4.18720	-1.03280
H	4.34560	-4.68620	-1.78940
H	5.05640	-4.85910	-0.17180
H	5.95850	-4.02570	-1.45440
C	5.23940	-2.11690	0.40620
H	5.48770	-2.76010	1.25760
H	4.76150	-1.21080	0.79200
H	6.18070	-1.83200	-0.07420
C	-1.06810	-4.33200	1.22740
H	-1.30680	-4.00750	2.24190
H	-1.03610	-5.42900	1.21170
H	-1.85780	-3.99210	0.55930
H	-0.29350	-3.48940	-1.28770
H	1.15610	-3.88530	2.76550

99
iTS-3-minor-E_60
G = -2623.2746004

N	2.31100	-0.15550	1.47300
H	3.14180	-0.49390	1.95440
H	2.63480	0.58630	0.82520
C	0.03610	0.72840	1.73110
H	0.23200	1.56190	1.04540
C	1.35730	0.40100	2.46590
H	1.14600	-0.40120	3.17580
C	2.47530	-3.42230	1.34480
C	1.08310	-3.67920	1.33370
C	3.10160	-3.09940	0.09700
C	0.96520	-3.41830	-1.13500
C	2.35830	-3.08830	-1.10990
C	3.26100	-3.56840	2.64840
H	3.13960	-4.63020	2.90750
N	-0.30600	-0.45230	0.88680
S	-1.81320	-1.02970	0.78110
O	-1.94150	-1.76380	-0.50970
O	-2.25860	-1.83250	1.96210
C	-0.96730	1.21890	2.76690
C	-1.36100	0.42100	3.85000
C	-1.43810	2.53420	2.69070
C	-2.21700	0.92760	4.83000
H	-1.01820	-0.60530	3.91640
C	-2.29070	3.04800	3.67000
H	-1.14900	3.15230	1.84800
C	-2.68410	2.24430	4.74460

H	-2.51910	0.29530	5.66040
H	-2.65270	4.06920	3.58870
H	-3.35090	2.63810	5.50670
C	1.93000	1.58950	3.21450
C	2.37960	2.72700	2.52660
C	1.98900	1.57370	4.61450
C	2.87410	3.82830	3.22980
H	2.33970	2.75560	1.44200
C	2.48250	2.67550	5.32020
H	1.63710	0.69890	5.15490
C	2.92600	3.80750	4.62820
H	3.21120	4.70590	2.68520
H	2.51890	2.64990	6.40580
H	3.30810	4.66610	5.17320
C	0.34700	-3.65490	0.11270
Ru	1.39760	-1.63730	0.27500
H	1.60100	-0.43210	-0.92650
C	-2.92000	0.38860	0.66780
C	-4.03620	0.47300	1.49960
C	-2.65430	1.37950	-0.27840
C	-4.89270	1.56880	1.37940
C	-3.51660	2.46950	-0.38320
C	-4.64800	2.58350	0.44110
H	4.15130	-2.83800	0.05980
H	0.55870	-3.88630	2.25920
H	-1.77130	1.32660	-0.90280
H	-3.29720	3.25040	-1.10690
H	-5.75440	1.64410	2.03780
H	-4.21200	-0.29210	2.24690
C	-5.58470	3.76070	0.31000
H	-6.36640	3.55190	-0.43120
H	-5.05200	4.65780	-0.02010
H	-6.08290	3.98150	1.25890
O	1.72130	3.86010	-2.10840
O	2.90060	1.50620	-0.57370
O	-0.04590	3.34140	-0.78820
O	-0.06140	-0.14840	-2.91150
N	-0.05770	1.15700	-2.51120
C	1.01720	1.57090	-1.94320
C	2.32400	0.90360	-1.53120
C	3.24590	0.22170	-2.54840
H	3.90270	1.04330	-2.86750
H	3.87430	-0.46660	-1.97470
C	2.66680	-0.45850	-3.78520
H	3.48800	-0.82130	-4.41200
H	2.03030	-1.30320	-3.52490
H	2.06560	0.23530	-4.37980
C	0.95430	3.04770	-1.61890
C	-0.29100	4.75220	-0.48920
H	-0.19910	5.32100	-1.41750
H	-1.33110	4.76360	-0.15930
C	0.65030	5.26570	0.58720
H	1.69020	5.21680	0.25480
H	0.55050	4.68480	1.50670
H	0.40410	6.30940	0.80890
C	-1.35030	-0.47330	-3.47320
H	-2.09120	-0.55180	-2.67480
H	-1.63720	0.27940	-4.21220

H	-1.21750	-1.44160	-3.95470
C	2.68030	-2.75290	3.82020
H	1.59470	-2.84550	3.90430
H	2.92330	-1.69150	3.72120
H	3.12190	-3.09830	4.76010
C	4.76710	-3.30480	2.50830
H	4.97270	-2.26700	2.22130
H	5.23140	-3.96300	1.76750
H	5.25810	-3.48500	3.46950
C	0.19020	-3.52150	-2.41850
H	0.22020	-4.55390	-2.78750
H	0.61130	-2.87580	-3.19110
H	-0.85110	-3.23940	-2.25340
H	2.86470	-2.83230	-2.03360
H	-0.72470	-3.80810	0.14250

99
iTS-3-minor-E_60-4
G = -2623.2677527

N	2.13280	-0.11430	1.60100
H	3.06640	-0.41140	1.87540
H	2.25290	0.73350	1.00690
C	-0.10200	0.50360	2.39260
H	-0.11630	1.39250	1.74480
C	1.36080	0.24530	2.81620
H	1.35880	-0.63810	3.46050
C	2.66930	-3.19280	0.83130
C	1.41300	-3.60640	1.37090
C	2.71010	-2.82260	-0.54540
C	0.29410	-3.33990	-0.83400
C	1.53450	-2.87590	-1.34250
C	3.93880	-3.22420	1.68150
H	4.26890	-4.27250	1.61690
N	-0.50630	-0.69280	1.61280
S	-2.08500	-0.82820	1.22730
O	-2.23650	-2.00270	0.33640
O	-2.94980	-0.83250	2.43960
C	-0.89360	0.81910	3.65520
C	-1.06720	-0.14240	4.66000
C	-1.36310	2.11710	3.87210
C	-1.70710	0.18650	5.85490
H	-0.71620	-1.15660	4.49490
C	-2.00800	2.45400	5.06820
H	-1.21980	2.87210	3.10540
C	-2.18200	1.48900	6.06370
H	-1.83860	-0.57010	6.62370
H	-2.36920	3.46770	5.21930
H	-2.68070	1.74630	6.99410
C	1.97760	1.40710	3.57090
C	2.11370	2.67050	2.97410
C	2.39810	1.23020	4.89460
C	2.65500	3.73730	3.69500
H	1.79640	2.82070	1.94740
C	2.93990	2.29740	5.61860
H	2.29100	0.25620	5.36500
C	3.06780	3.55480	5.02010
H	2.75070	4.71180	3.22390
H	3.25810	2.14690	6.64650
H	3.48600	4.38600	5.58100

C	0.25580	-3.64760	0.55190
Ru	1.11700	-1.53920	0.41390
H	1.12780	-0.19080	-0.70110
C	-2.63940	0.60070	0.27410
C	-2.48980	0.59400	-1.11430
C	-3.23560	1.68610	0.92040
C	-2.95360	1.67800	-1.85740
C	-3.69190	2.76600	0.16200
C	-3.56290	2.77810	-1.23470
H	3.62710	-2.47140	-0.99800
H	1.31880	-3.86220	2.41780
H	-3.35030	1.68080	1.99800
H	-4.15130	3.61230	0.66600
H	-2.82160	1.67470	-2.93540
H	-2.01700	-0.24940	-1.60380
C	-4.07740	3.93670	-2.05550
H	-3.40230	4.16200	-2.88740
H	-4.18980	4.83870	-1.44680
H	-5.05870	3.70160	-2.48620
O	0.27900	3.78700	-2.96920
O	2.02080	1.85900	-0.19780
O	-0.24140	3.33230	-0.81880
O	0.27510	-0.40400	-3.11410
N	0.00090	0.93880	-3.02750
C	0.64860	1.56620	-2.11310
C	1.79940	1.12090	-1.21250
C	3.09710	0.61090	-1.89090
H	3.67380	1.54010	-1.98930
H	3.61180	0.01460	-1.13210
C	3.10800	-0.09960	-3.24300
H	4.14770	-0.20340	-3.57200
H	2.66700	-1.09360	-3.20110
H	2.57190	0.47260	-4.00570
C	0.21490	3.01540	-2.02360
C	-0.61640	4.72400	-0.58930
H	0.18580	5.36260	-0.96850
H	-1.52600	4.92650	-1.16090
C	-0.82660	4.89220	0.90130
H	0.08800	4.65830	1.45380
H	-1.62930	4.24010	1.25260
H	-1.10500	5.92970	1.11200
C	-0.35350	-0.94250	-4.29240
H	-1.43690	-0.79610	-4.24620
H	0.05770	-0.47100	-5.18970
H	-0.11600	-2.00630	-4.28760
C	3.70580	-2.92650	3.17460
H	2.99680	-3.61900	3.63440
H	3.33500	-1.91110	3.34010
H	4.65240	-3.02410	3.71420
C	5.08320	-2.36010	1.12190
H	4.79350	-1.30870	1.01750
H	5.42320	-2.70930	0.14320
H	5.94010	-2.40470	1.80010
C	-0.89730	-3.50950	-1.74040
H	-1.73750	-3.94820	-1.20280
H	-0.63180	-4.16200	-2.57970
H	-1.22380	-2.54940	-2.14260
H	1.58460	-2.55510	-2.37540

H	-0.69890	-3.89370	1.00150

26
start-3
G = -668.3319372

O	1.23380	6.22590	2.46610
O	2.63490	3.52930	1.16640
O	-0.32900	4.58580	2.39140
O	-0.69660	6.31940	-0.18390
N	0.30710	5.46520	-0.55840
C	0.93840	4.96780	0.44530
C	2.06190	4.01130	0.19340
C	2.43160	3.68600	-1.23540
H	2.66000	4.63280	-1.74100
H	1.53120	3.30450	-1.73280
C	3.59180	2.69950	-1.35270
H	3.80910	2.50670	-2.40720
H	3.35100	1.74730	-0.87090
H	4.49620	3.09570	-0.88160
C	0.63900	5.33890	1.88210
C	-0.72480	4.85810	3.77800
H	-1.03940	5.90340	3.83650
H	0.15500	4.71680	4.41110
C	-1.84390	3.89820	4.12030
H	-2.70370	4.04930	3.46080
H	-2.16380	4.07500	5.15190
H	-1.50870	2.86030	4.03510
C	-1.37360	6.84980	-1.34530
H	-2.14040	7.51220	-0.94320
H	-1.83160	6.03810	-1.91650
H	-0.67100	7.41170	-1.96600

26
start-2
G = -668.3312988

O	1.12960	5.87540	2.65990
O	2.70860	3.45600	1.05590
O	-0.33860	4.17590	2.31830
O	-0.73790	6.23070	-0.00470
N	0.31430	5.48220	-0.46230
C	0.94830	4.88490	0.48380
C	2.12440	4.02580	0.13880
C	2.53090	3.90000	-1.31150
H	2.71900	4.91250	-1.69060
H	1.65820	3.54130	-1.87120
C	3.74060	2.99230	-1.52610
H	3.98630	2.94780	-2.59100
H	3.53850	1.97550	-1.17700
H	4.61550	3.36590	-0.98610
C	0.59700	5.04500	1.94760
C	-0.80930	4.22880	3.70870
H	0.05180	4.40510	4.35640
H	-1.20010	3.22620	3.88390
C	-1.87900	5.29450	3.87110
H	-1.47390	6.29090	3.67610
H	-2.25610	5.26900	4.89880
H	-2.71560	5.11010	3.19080
C	-1.41720	6.88010	-1.10240
H	-1.82340	6.13220	-1.78820
H	-0.73100	7.55190	-1.62450

H -2.22330 7.44660 -0.63590
26
Start
G = -668.3309703

O	0.81370	6.19330	2.48490
O	2.47750	3.57270	1.29580
O	-0.62180	4.45850	2.18690
O	-0.75050	6.31480	-0.36420
N	0.31650	5.50080	-0.64040
C	0.83340	4.98120	0.41610
C	2.00650	4.06330	0.27370
C	2.54920	3.78480	-1.10890
H	2.80600	4.74980	-1.56370
H	1.72450	3.39130	-1.71620
C	3.74450	2.83360	-1.11090
H	4.08630	2.67000	-2.13690
H	3.47930	1.86480	-0.67760
H	4.57720	3.24440	-0.53240
C	0.34860	5.28870	1.81730
C	-1.19540	4.63210	3.52810
H	-2.18680	4.18670	3.44050
H	-1.29070	5.70210	3.72400
C	-0.34450	3.93000	4.57140
H	-0.24370	2.86630	4.33710
H	-0.82730	4.02500	5.54960
H	0.65100	4.37760	4.63230
C	-1.29500	6.87060	-1.58210
H	-2.12520	7.49750	-1.25630
H	-1.65430	6.06940	-2.23300
H	-0.53850	7.47330	-2.09130

26
start-8
G = -668.3303249

O	1.42470	5.90600	2.53230
O	2.35800	3.02700	1.22000
O	-0.41660	4.59070	2.39180
O	-0.42690	6.34740	-0.16840
N	0.43210	5.34490	-0.52700
C	0.95520	4.74810	0.48590
C	1.91850	3.63100	0.24420
C	2.34600	3.31560	-1.16910
H	1.48690	3.38290	-1.84230
H	2.71570	2.28670	-1.16840
C	3.45400	4.28170	-1.64100
H	3.77420	4.00640	-2.64980
H	4.32440	4.23170	-0.97930
H	3.09300	5.31360	-1.66440
C	0.68990	5.15330	1.91980
C	-0.79540	4.91630	3.77180
H	-0.90300	6.00190	3.84190
H	0.02010	4.59790	4.42650
C	-2.08980	4.18770	4.06320
H	-2.88240	4.51200	3.38250
H	-2.40220	4.40920	5.08850
H	-1.95990	3.10570	3.96730
C	-0.98430	6.98290	-1.34130
H	-1.55440	6.25660	-1.92600
H	-0.18610	7.42430	-1.94340

```
H          -1.64250         7.75970        -0.95180
26
start-9
G = -668.3302620
O          -0.92870         3.37100         1.59750
O           2.34180         2.98640         1.10260
O           0.18190         5.10560         2.54440
O          -0.77610         5.96430        -0.35060
N           0.40970         5.33070        -0.60330
C           0.75560         4.52480         0.33820
C           2.03890         3.77080         0.20600
C           2.93450         4.03240        -0.98110
H           2.33170         4.19310        -1.87870
H           3.54740         3.13770        -1.12070
C           3.83480         5.26120        -0.73030
H           4.50230         5.40670        -1.58430
H           4.44830         5.11980         0.16480
H           3.23660         6.16790        -0.60320
C          -0.09780         4.25960         1.55990
C          -0.58880         4.96220         3.78500
H          -0.42190         3.95200         4.16780
H          -1.64710         5.07600         3.53570
C          -0.10210         6.03080         4.74000
H           0.96260         5.90540         4.95760
H          -0.65900         5.95190         5.67890
H          -0.26470         7.02970         4.32450
C          -1.12330         6.84830        -1.44070
H          -2.07020         7.29940        -1.14390
H          -1.24520         6.27550        -2.36340
H          -0.35470         7.61610        -1.55990
26
start-5
G = -668.3295101
O           1.27790         5.53090         2.70080
O           2.40260         2.91090         1.03980
O          -0.49270         4.16930         2.28590
O          -0.48370         6.27460         0.01010
N           0.43820         5.37360        -0.44810
C           0.94840         4.66010         0.49350
C           1.97590         3.63310         0.14150
C           2.47890         3.55050        -1.27990
H           1.64470         3.66800        -1.97720
H           2.91040         2.55440        -1.40910
C           3.54280         4.63500        -1.55540
H           3.91730         4.52880        -2.57740
H           4.38890         4.53660        -0.86810
H           3.12050         5.63770        -1.44660
C           0.60390         4.84430         1.95570
C          -0.97110         4.27620         3.67060
H          -0.10500         4.26280         4.33530
H          -1.55360         3.36520         3.81020
C          -1.81480         5.52600         3.85010
H          -1.21930         6.42840         3.68930
H          -2.20840         5.54830         4.87160
H          -2.65870         5.52670         3.15400
C          -1.02500         7.05100        -1.08250
H          -1.53530         6.39490        -1.79230
H          -0.22680         7.61060        -1.57670
```

H -1.73560 7.73270 -0.61490
26
start-7
G = -668.3294554

O	1.01020	6.08190	2.64330
O	3.02970	3.83260	1.25380
O	-0.12880	4.13880	2.34690
O	-0.69720	6.05750	-0.10900
N	0.46340	5.43280	-0.47640
C	1.11270	4.95820	0.52810
C	2.40050	4.24190	0.28060
C	2.85230	4.00090	-1.13970
H	3.93470	3.84920	-1.10850
H	2.63470	4.87740	-1.75590
C	2.15610	2.75840	-1.73570
H	2.52870	2.58020	-2.74830
H	1.07320	2.90140	-1.78820
H	2.36160	1.86830	-1.13300
C	0.66370	5.13480	1.96270
C	-0.67090	4.18390	3.71140
H	0.11220	4.54670	4.38030
H	-0.88800	3.13900	3.93480
C	-1.91760	5.04980	3.76280
H	-1.68390	6.08940	3.51870
H	-2.33450	5.01690	4.77480
H	-2.67440	4.68100	3.06430
C	-1.38170	6.58270	-1.26890
H	-1.64280	5.76910	-1.95030
H	-0.75390	7.32270	-1.77160
H	-2.28200	7.05230	-0.87270

26
start-6
G = -668.3294376

O	-1.03180	3.80080	1.80110
O	2.20280	3.07480	1.30170
O	0.29120	5.51660	2.48450
O	-0.74630	6.03420	-0.49810
N	0.38550	5.28800	-0.68190
C	0.70810	4.60560	0.36000
C	1.93220	3.75030	0.31120
C	2.81230	3.78520	-0.91520
H	2.19660	3.84270	-1.81680
H	3.37610	2.84850	-0.92990
C	3.77800	4.98900	-0.86530
H	4.43080	4.97170	-1.74250
H	4.40550	4.94980	0.03040
H	3.22830	5.93450	-0.86480
C	-0.11760	4.58490	1.62870
C	-0.40730	5.61610	3.77310
H	-1.47540	5.47460	3.59580
H	-0.22140	6.64430	4.08460
C	0.14910	4.61020	4.76500
H	-0.02990	3.58590	4.42800
H	-0.34760	4.74880	5.73090
H	1.22400	4.75750	4.90420
C	-1.06500	6.77760	-1.69640
H	-0.25040	7.46480	-1.93890
H	-1.97050	7.33180	-1.44870

H	-1.25210	6.09040	-2.52540

26
start-4
G = -668.3294196

O	-0.45000	3.50070	1.95540
O	2.79910	4.09530	1.57620
O	-0.03210	5.67870	2.44850
O	-0.95310	5.54860	-0.54670
N	0.38210	5.26600	-0.64180
C	0.87800	4.81970	0.45830
C	2.32960	4.46790	0.50320
C	3.14810	4.53760	-0.76380
H	4.19500	4.62960	-0.46250
H	2.86670	5.42200	-1.34210
C	2.94810	3.26950	-1.62140
H	3.58060	3.32620	-2.51180
H	1.90790	3.17230	-1.94430
H	3.22450	2.37080	-1.06120
C	0.05450	4.57970	1.70570
C	-0.80260	5.60170	3.69700
H	-1.69520	5.00060	3.51230
H	-1.09060	6.63690	3.88230
C	0.04860	5.03670	4.82020
H	0.34250	4.00520	4.60920
H	-0.53150	5.04860	5.74880
H	0.94820	5.64150	4.96630
C	-1.45750	6.04800	-1.80620
H	-0.94390	6.97470	-2.07480
H	-2.51620	6.23670	-1.62810
H	-1.32950	5.29390	-2.58680

26
start-10
G = -668.3273349

O	1.34800	5.78880	2.43640
O	2.04130	3.98060	-1.55560
O	-0.24730	4.18690	2.21700
O	-0.89660	6.29810	0.10370
N	0.06820	5.57530	-0.54730
C	0.82900	4.92540	0.26110
C	1.94010	4.09590	-0.34250
C	2.88670	3.43670	0.63370
H	2.28190	2.83590	1.32830
H	3.32790	4.23210	1.25000
C	3.96660	2.58780	-0.03130
H	4.61160	2.14060	0.73010
H	4.58760	3.19460	-0.69660
H	3.52340	1.78300	-0.62510
C	0.67670	5.02840	1.76260
C	-0.52440	4.18810	3.65940
H	0.42400	4.29070	4.19090
H	-0.93970	3.19620	3.83940
C	-1.50540	5.29120	4.01550
H	-1.07910	6.27680	3.81170
H	-1.74030	5.22930	5.08310
H	-2.43490	5.18060	3.44940
C	-1.71250	7.02520	-0.84100
H	-2.22300	6.32900	-1.51140
H	-1.09580	7.72710	-1.40840

```
H          -2.43590        7.56340        -0.22820
26
prod-5
G = -668.3301977
O           2.79620        2.40000         0.25080
O           0.68360       -0.34060        -2.43090
O           3.12780        1.64280        -1.86000
O          -1.19110        1.55920        -0.52260
N           0.09120        1.90720        -0.17070
C           0.94690        1.58230        -1.07200
C           0.57500        0.87350        -2.36920
C           0.08620        1.75320        -3.48770
H           0.88680        2.47870        -3.69110
H          -0.74480        2.34620        -3.08130
C          -0.32080        0.99530        -4.74900
H          -0.66430        1.69840        -5.51290
H          -1.13180        0.29170        -4.53850
H           0.52170        0.42850        -5.15590
C           2.37790        1.92510        -0.79000
C           4.55920        1.92740        -1.77020
H           4.96330        1.36050        -0.92710
H           4.67820        2.99500        -1.56610
C           5.17870        1.51950        -3.09020
H           5.03060        0.45180        -3.27750
H           6.25410        1.71990        -3.05880
H           4.74440        2.08840        -3.91760
C          -2.12510        1.99010         0.49070
H          -2.10510        3.07920         0.58330
H          -1.88780        1.51690         1.44710
H          -3.09970        1.65730         0.13290
26
prod-3
G = -668.3297175
O           2.91000        1.63050        -3.06670
O           0.11480       -0.08280        -3.17960
O           3.29080        1.73520        -0.83620
O          -0.69650        1.29390        -0.27960
N           0.66050        1.49850        -0.38540
C           1.06960        1.39780        -1.59890
C           0.15650        1.06740        -2.77550
C          -0.63260        2.21150        -3.35010
H           0.10290        2.95510        -3.69080
H          -1.15900        2.68810        -2.51230
C          -1.58910        1.81180        -4.47130
H          -2.12210        2.69270        -4.83960
H          -2.32680        1.08610        -4.11580
H          -1.04790        1.36130        -5.30840
C           2.51650        1.59730        -1.90860
C           4.71760        1.95670        -1.07720
H           4.82330        2.86910        -1.67050
H           5.09820        1.11290        -1.65920
C           5.38620        2.06950         0.27670
H           4.97830        2.91020         0.84560
H           6.45850        2.23580         0.13410
H           5.25270        1.15120         0.85620
C          -1.12740        1.46960         1.08700
H          -0.93930        2.49600         1.41300
H          -0.61370        0.75750         1.73820
```

H	-2.19850	1.26660	1.07150

26
prod-6
G = -668.3293561

O	2.95730	2.19620	0.08680
O	0.24420	2.59330	-3.32950
O	3.09410	1.73190	-2.13260
O	-1.12030	1.71780	-0.50180
N	0.20890	1.86220	-0.18210
C	0.96530	1.79840	-1.21840
C	0.43680	1.60830	-2.63520
C	0.18540	0.18890	-3.06510
H	-0.48350	-0.25390	-2.31410
H	1.13930	-0.34640	-2.95490
C	-0.37790	0.05380	-4.47770
H	-0.53030	-1.00150	-4.72030
H	0.30650	0.48240	-5.21560
H	-1.33820	0.57040	-4.56720
C	2.43820	1.93900	-0.98420
C	4.55630	1.79070	-2.10960
H	4.85840	2.60360	-1.44610
H	4.82000	2.04060	-3.13780
C	5.13930	0.45490	-1.68150
H	4.85090	0.21240	-0.65520
H	6.23180	0.50780	-1.73080
H	4.80080	-0.34540	-2.34620
C	-1.93270	1.75320	0.69130
H	-1.80160	2.70800	1.20760
H	-1.67350	0.91960	1.34940
H	-2.95800	1.65140	0.33490

26
prod-4
G = -668.3292485

O	2.90400	2.52280	-0.04550
O	0.67300	-0.29080	-2.55640
O	3.05820	1.80850	-2.19610
O	-1.08410	1.46040	-0.41930
N	0.20700	1.87400	-0.19310
C	0.97900	1.61810	-1.18760
C	0.51200	0.91490	-2.45660
C	-0.13060	1.78750	-3.50050
H	0.60940	2.55540	-3.76850
H	-0.94340	2.33150	-2.99990
C	-0.62700	1.03050	-4.72990
H	-1.07930	1.72770	-5.44070
H	-1.37790	0.28440	-4.45350
H	0.19600	0.51250	-5.23100
C	2.41090	2.03650	-1.04710
C	4.48190	2.14190	-2.26010
H	4.64010	3.08250	-1.72820
H	4.66310	2.28900	-3.32530
C	5.32430	1.01330	-1.69070
H	5.11790	0.86820	-0.62700
H	6.38390	1.26340	-1.80670
H	5.12880	0.07810	-2.22350
C	-1.92760	1.80510	0.70090
H	-1.95780	2.89020	0.83060
H	-1.56440	1.31760	1.60950

H	-2.91490	1.42680	0.43470

26
prod
G = -668.3287782

O	3.00910	0.67080	-2.47840
O	0.26730	1.94970	-3.74780
O	3.17710	1.78020	-0.50520
O	-0.82040	2.38190	-0.66570
N	0.53390	2.18710	-0.51810
C	1.05130	1.60650	-1.54060
C	0.25480	1.20790	-2.77940
C	-0.49690	-0.09250	-2.70030
H	-1.10080	-0.05630	-1.78370
H	0.25500	-0.87520	-2.52100
C	-1.34670	-0.40060	-3.93100
H	-1.85800	-1.35860	-3.80180
H	-0.72860	-0.45850	-4.83160
H	-2.10220	0.37490	-4.08850
C	2.51420	1.30750	-1.55850
C	4.61940	1.53030	-0.44500
H	4.81140	0.51510	-0.79860
H	4.84540	1.59450	0.62000
C	5.38040	2.57000	-1.24960
H	5.13040	2.50410	-2.31180
H	6.45540	2.39570	-1.13550
H	5.15370	3.57850	-0.89140
C	-1.37300	2.99470	0.51910
H	-0.89990	3.96410	0.69680
H	-1.23990	2.33620	1.38150
H	-2.43310	3.12340	0.29940

26
prod-2
G = -668.3286709

O	3.04920	1.28900	-2.77390
O	0.02230	2.15820	-3.69580
O	3.19280	2.24870	-0.72170
O	-0.84190	2.05370	-0.50350
N	0.53310	2.10680	-0.48060
C	1.04850	1.75040	-1.60190
C	0.22460	1.33660	-2.81720
C	-0.28240	-0.07930	-2.82910
H	-0.79420	-0.24330	-1.87140
H	0.60610	-0.72700	-2.79710
C	-1.17920	-0.41300	-4.01910
H	-1.50220	-1.45620	-3.96270
H	-0.64940	-0.26650	-4.96470
H	-2.06930	0.22330	-4.02820
C	2.53350	1.73930	-1.76010
C	4.65690	2.24050	-0.77970
H	4.94360	3.03750	-0.09270
H	4.96560	2.50400	-1.79350
C	5.20740	0.89280	-0.34630
H	4.86660	0.64160	0.66240
H	6.30150	0.93750	-0.34110
H	4.89740	0.10240	-1.03510
C	-1.38060	2.41250	0.78710
H	-1.07350	3.42720	1.05410
H	-1.05110	1.69730	1.54540

H	-2.46260	2.36270	0.66280

26
prod-9
G = -668.3273378

O	2.73320	1.17280	-3.14690
O	0.04840	-0.52870	-2.82010
O	3.18660	1.81660	-1.02360
O	-0.74890	1.46400	-0.18900
N	0.59500	1.64750	-0.41440
C	0.94760	1.27990	-1.59400
C	-0.00580	0.67460	-2.62200
C	-0.95730	1.60730	-3.32260
H	-1.59800	2.05070	-2.55020
H	-1.58190	1.00620	-3.98920
C	-0.23360	2.73010	-4.09240
H	-0.97410	3.37510	-4.57300
H	0.42120	2.31930	-4.86590
H	0.36980	3.35110	-3.42320
C	2.37930	1.41320	-2.00110
C	4.59840	1.99410	-1.36590
H	4.66190	2.73480	-2.16780
H	4.97980	1.03940	-1.73820
C	5.30850	2.44580	-0.10720
H	4.89900	3.39380	0.25410
H	6.37120	2.58910	-0.32650
H	5.21680	1.69560	0.68380
C	-1.10810	1.93690	1.12710
H	-0.91790	3.01050	1.20670
H	-0.54950	1.38780	1.88970
H	-2.17530	1.73270	1.21610

26
prod-10
G = -668.3271659

O	2.80890	2.76510	-0.39730
O	0.77380	-0.62650	-2.16450
O	2.95900	1.58610	-2.33210
O	-1.12810	1.49540	-0.38870
N	0.14910	1.99520	-0.31360
C	0.90680	1.54820	-1.24990
C	0.45050	0.54300	-2.30280
C	-0.39350	1.05880	-3.43830
H	-1.35050	1.36730	-2.99670
H	-0.58410	0.22170	-4.11570
C	0.23540	2.25390	-4.17860
H	-0.43280	2.57670	-4.98130
H	1.19930	1.98290	-4.61800
H	0.39010	3.10530	-3.50850
C	2.32210	2.04240	-1.24820
C	4.36230	1.95850	-2.51280
H	4.48510	3.00030	-2.20940
H	4.51420	1.87070	-3.58920
C	5.27020	1.02350	-1.73230
H	5.09150	1.11090	-0.65730
H	6.31400	1.28690	-1.93250
H	5.11020	-0.01480	-2.03770
C	-1.94870	2.04810	0.66320
H	-2.02070	3.13340	0.55440
H	-1.53570	1.78410	1.64040

H -2.92700 1.58670 0.52640
26
prod-8
G = -668.3265474

O	2.88360	0.95290	-2.80980
O	0.09060	-0.58820	-2.71120
O	3.13840	1.68160	-0.67610
O	-0.87650	1.57520	-0.27780
N	0.49250	1.67670	-0.35760
C	0.95130	1.23000	-1.47150
C	0.08440	0.62470	-2.57340
C	-0.72990	1.56930	-3.41710
H	-1.43570	2.07120	-2.74340
H	-1.29990	0.96900	-4.13160
C	0.13130	2.62780	-4.13450
H	-0.51640	3.28210	-4.72400
H	0.85360	2.15830	-4.80800
H	0.67900	3.25130	-3.42110
C	2.42410	1.27020	-1.72190
C	4.58680	1.80730	-0.85570
H	4.93960	0.95570	-1.44130
H	4.97630	1.73760	0.16060
C	4.93590	3.13420	-1.50740
H	4.52130	3.19610	-2.51710
H	6.02500	3.22600	-1.57450
H	4.55420	3.96940	-0.91290
C	-1.35140	2.12820	0.96850
H	-1.10790	3.19230	1.02590
H	-0.91510	1.58560	1.81130
H	-2.43220	1.98660	0.94340

26
prod-7
G = -668.3262587

O	2.84730	0.98370	-2.84170
O	0.16980	2.49820	-3.68770
O	3.10990	1.58910	-0.67080
O	-0.87380	2.19970	-0.47120
N	0.48220	1.97240	-0.45770
C	0.93760	1.65940	-1.61750
C	0.08240	1.58630	-2.88080
C	-0.82430	0.39550	-3.04440
H	-1.35560	0.51180	-3.99320
H	-1.55730	0.44090	-2.22890
C	-0.07310	-0.94870	-2.98130
H	-0.78660	-1.76820	-3.10230
H	0.43170	-1.08230	-2.01950
H	0.67400	-1.02120	-3.77650
C	2.39730	1.38040	-1.77610
C	4.55110	1.33630	-0.73910
H	4.71810	0.43440	-1.33180
H	4.82660	1.14740	0.29900
C	5.28240	2.53920	-1.31000
H	4.98310	2.72510	-2.34490
H	6.36010	2.34650	-1.29060
H	5.08020	3.43370	-0.71360
C	-1.34650	2.50860	0.85770
H	-0.85080	3.40760	1.23370
H	-1.17100	1.66200	1.52660

```
H         -2.41600        2.68490        0.74030
26
ts-6
G = -668.2571046
O         -0.77550       -1.31740       -2.00370
O         -2.28350       -1.61280        0.55090
O          1.24950       -0.71060       -1.19810
O          0.49390        2.15250        0.87640
N         -0.06650        1.08100        0.41100
C         -0.64980        0.04780        0.00660
C         -1.94380       -0.43960        0.64740
C         -2.71730        0.58580        1.44920
H         -2.92200        1.43460        0.78260
H         -2.04110        0.98460        2.21670
C         -3.99990        0.04090        2.07250
H         -4.51200        0.83010        2.63090
H         -3.78440       -0.78190        2.76070
H         -4.68300       -0.33500        1.30450
C         -0.08590       -0.76110       -1.16340
C          1.91270       -1.31350       -2.34900
H          1.51640       -0.84560       -3.25480
H          1.66530       -2.37890       -2.36960
C          3.39930       -1.07200       -2.18380
H          3.61790       -0.00030       -2.14900
H          3.93420       -1.50700       -3.03390
H          3.77130       -1.53750       -1.26610
C          1.79990        2.45080        0.24810
H          1.64310        2.62390       -0.81740
H          2.47210        1.60970        0.42090
H          2.14290        3.35260        0.75520
26
ts-3
G = -668.2567269
O          0.91580        0.74360       -2.04810
O         -2.13970       -1.20930       -0.52510
O          0.69170       -1.32880       -1.17360
O          0.00060        2.62890        1.13890
N         -0.22020        1.52080        0.50310
C         -0.47540        0.44230       -0.08120
C         -1.69850       -0.39180        0.27450
C         -2.34560       -0.09960        1.61060
H         -2.58560        0.97130        1.63530
H         -1.57500       -0.23500        2.38180
C         -3.58040       -0.95030        1.89740
H         -3.99180       -0.69730        2.87900
H         -3.33490       -2.01670        1.89220
H         -4.35780       -0.78160        1.14600
C          0.43040       -0.02100       -1.22970
C          1.60210       -1.87010       -2.18090
H          2.55080       -1.33090       -2.10750
H          1.16820       -1.68030       -3.16680
C          1.75930       -3.34940       -1.89710
H          2.18200       -3.51300       -0.90120
H          2.43650       -3.78870       -2.63630
H          0.79610       -3.86430       -1.96120
C          1.15640        3.38980        0.61670
H          0.96360        3.65100       -0.42470
H          2.05280        2.77610        0.71730
```

H	1.20420	4.27550	1.25030

26
ts-4
G = -668.2562627

O	-0.47640	-1.50130	-1.83810
O	-2.13610	-1.68150	0.67030
O	1.44430	-0.84130	-0.82780
O	0.32700	2.33090	0.63680
N	-0.11210	1.15270	0.32160
C	-0.58530	0.02500	0.04740
C	-1.87480	-0.48390	0.67680
C	-2.74760	0.55520	1.34710
H	-2.97430	1.32470	0.59670
H	-2.13410	1.06650	2.10050
C	-4.02100	-0.01600	1.96590
H	-4.60700	0.78420	2.42750
H	-3.78640	-0.75740	2.73560
H	-4.64270	-0.50420	1.20900
C	0.11370	-0.88380	-0.96620
C	2.26190	-1.48500	-1.85320
H	1.77390	-2.41140	-2.16400
H	3.19330	-1.72030	-1.33630
C	2.48710	-0.53840	-3.02070
H	1.54260	-0.29830	-3.51690
H	3.15060	-1.01290	-3.75120
H	2.95450	0.39030	-2.67980
C	1.64270	2.64580	0.03800
H	1.54940	2.61120	-1.04840
H	2.37390	1.92160	0.39900
H	1.86590	3.65290	0.39040

26
ts-5
G = -668.2560857

O	0.87780	-1.85040	-0.07360
O	-2.03950	-1.66800	0.46460
O	1.10030	-0.21480	-1.62770
O	-0.31750	2.67630	0.02380
N	-0.39270	1.38280	0.03360
C	-0.51510	0.13580	0.07470
C	-1.70970	-0.52410	0.75410
C	-2.47950	0.33960	1.73150
H	-2.80120	1.24150	1.19460
H	-1.76460	0.69780	2.48480
C	-3.66640	-0.37010	2.37850
H	-4.17420	0.30400	3.07450
H	-3.34180	-1.25600	2.93310
H	-4.39000	-0.69380	1.62440
C	0.54100	-0.77840	-0.55000
C	2.21270	-0.91140	-2.26670
H	2.82460	-1.37850	-1.49160
H	2.78110	-0.11230	-2.74570
C	1.70510	-1.92550	-3.27790
H	1.12460	-2.71060	-2.78600
H	2.55740	-2.39010	-3.78480
H	1.07820	-1.43910	-4.03140
C	0.82430	3.19340	-0.76270
H	0.71940	2.85780	-1.79490
H	1.74970	2.82870	-0.31530

H	0.73870	4.27710	-0.68070

26
ts
G = -668.2557338

O	1.16220	0.48740	-1.85060
O	-2.02920	-1.33470	-0.43730
O	0.85460	-1.48000	-0.77010
O	-0.11790	2.71710	0.95660
N	-0.25280	1.53280	0.44660
C	-0.42850	0.38400	-0.02110
C	-1.66980	-0.43280	0.31040
C	-2.43840	-0.01860	1.54630
H	-2.69260	1.04380	1.43800
H	-1.73900	-0.05800	2.39260
C	-3.68400	-0.86150	1.80930
H	-4.18610	-0.51780	2.71830
H	-3.42620	-1.91740	1.93730
H	-4.39140	-0.78800	0.97780
C	0.59680	-0.19040	-1.00790
C	1.84920	-2.14490	-1.61210
H	2.18390	-2.98570	-1.00300
H	2.68290	-1.45800	-1.77460
C	1.22920	-2.60640	-2.92040
H	0.38560	-3.27740	-2.73360
H	1.98050	-3.14830	-3.50440
H	0.88180	-1.75420	-3.51050
C	1.06650	3.44530	0.45320
H	0.97270	3.56360	-0.62710
H	1.96210	2.88190	0.71890
H	1.03050	4.40600	0.96690

26
ts-2
G = -668.2556331

O	1.91340	-0.33730	-0.36540
O	-1.55020	-1.74670	0.78780
O	0.25270	-1.40610	-1.47530
O	-0.17430	2.74250	0.51520
N	-0.26180	1.46590	0.30730
C	-0.39260	0.23250	0.13040
C	-1.60300	-0.52980	0.65040
C	-2.80800	0.30060	1.03440
H	-3.10350	0.87530	0.14580
H	-2.47480	1.05340	1.76050
C	-3.97240	-0.52030	1.58320
H	-4.81220	0.13590	1.83010
H	-3.68040	-1.05940	2.48950
H	-4.31560	-1.25740	0.85070
C	0.72870	-0.54680	-0.56960
C	1.21720	-2.15670	-2.27990
H	0.66310	-3.04380	-2.58990
H	2.04960	-2.45240	-1.63760
C	1.68150	-1.33520	-3.47090
H	0.82970	-1.03060	-4.08650
H	2.35470	-1.94020	-4.08730
H	2.22110	-0.44250	-3.14340
C	1.10460	3.32900	0.05950
H	1.19500	3.17620	-1.01690
H	1.92200	2.85280	0.60260

```
H          1.02030         4.38700         0.30820
26
ts-9
G = -668.2553540
O          1.15600         0.71850        -1.82180
O         -2.25270        -1.09530        -1.01490
O          0.63420        -1.36770        -1.12440
O         -0.30430         2.56890         1.16450
N         -0.44160         1.48840         0.46320
C         -0.61820         0.43360        -0.18940
C         -1.92260        -0.34720        -0.10090
C         -2.78420        -0.10880         1.11890
H         -3.75670        -0.57070         0.93000
H         -2.93050         0.97000         1.24440
C         -2.14030        -0.68980         2.39400
H         -2.80040        -0.52460         3.25030
H         -1.17980        -0.21160         2.60680
H         -1.97480        -1.76730         2.29380
C          0.47090        -0.04340        -1.15850
C          1.68640        -1.93230        -1.96720
H          2.63780        -1.47910        -1.67440
H          1.47350        -1.65810        -3.00430
C          1.67580        -3.43190        -1.75570
H          1.87620        -3.68010        -0.70900
H          2.45420        -3.88930        -2.37450
H          0.71040        -3.86000        -2.04170
C          0.95150         3.30020         0.88770
H          0.95890         3.59880        -0.16140
H          1.79390         2.64950         1.12610
H          0.91190         4.16440         1.55100
26
ts-7
G = -668.2543053
O          1.33910         0.44620        -1.60960
O         -2.16750        -1.23690        -0.94170
O          0.71440        -1.54580        -0.72970
O         -0.37420         2.63990         0.97750
N         -0.45850         1.48330         0.39950
C         -0.58770         0.35900        -0.13790
C         -1.90550        -0.40220        -0.08220
C         -2.86270        -0.03700         1.03010
H         -3.82150        -0.51320         0.80950
H         -3.00640         1.04940         1.03190
C         -2.33160        -0.48530         2.40680
H         -3.05680        -0.22990         3.18460
H         -1.38580         0.00890         2.64720
H         -2.17140        -1.56800         2.43190
C          0.58090        -0.23240        -0.93620
C          1.81730        -2.23460        -1.40000
H          1.97510        -3.12510        -0.79010
H          2.70420        -1.59850        -1.35310
C          1.44550        -2.58770        -2.83050
H          0.54350        -3.20630        -2.85450
H          2.26520        -3.15190        -3.28760
H          1.27400        -1.68550        -3.42370
C          0.91110         3.32980         0.73300
H          1.02140         3.49510        -0.33950
H          1.71890         2.71350         1.12980
```

H	0.81950	4.26990	1.27720

26
ts-8
G = -668.2541902

O	0.99270	0.47290	-1.96160
O	-2.49680	-1.00230	-0.96710
O	0.35740	-1.55760	-1.18230
O	-0.07410	2.41700	1.14880
N	-0.36260	1.36960	0.44300
C	-0.68550	0.35250	-0.21330
C	-2.06040	-0.28470	-0.07330
C	-2.85010	0.05160	1.17170
H	-3.87600	-0.29080	1.01340
H	-2.86250	1.13950	1.30200
C	-2.24080	-0.60760	2.42570
H	-2.84870	-0.36750	3.30260
H	-1.22350	-0.24910	2.60750
H	-2.20940	-1.69680	2.32000
C	0.29860	-0.22390	-1.23940
C	1.31560	-2.23890	-2.05340
H	1.32010	-1.73740	-3.02370
H	0.90000	-3.24150	-2.16350
C	2.69670	-2.26600	-1.42050
H	3.09210	-1.25380	-1.30120
H	3.37860	-2.83050	-2.06510
H	2.66400	-2.75330	-0.44130
C	1.24290	3.00640	0.82350
H	1.23960	3.31590	-0.22240
H	2.01630	2.26270	1.01990
H	1.32920	3.86110	1.49480

26
ts-10
G = -668.2537242

O	1.82370	-0.05070	-0.59530
O	-1.60200	-1.86540	0.15720
O	0.26630	-1.03290	-1.91430
O	-0.44950	2.62660	0.85530
N	-0.46390	1.42430	0.37350
C	-0.52410	0.25240	-0.06680
C	-1.70910	-0.64640	0.24620
C	-2.95760	0.01840	0.78520
H	-3.80340	-0.62250	0.51960
H	-3.09280	0.99710	0.31500
C	-2.88410	0.18250	2.31870
H	-3.81460	0.62150	2.69030
H	-2.05740	0.83890	2.60580
H	-2.74370	-0.78690	2.80700
C	0.66180	-0.31200	-0.86110
C	1.30040	-1.58310	-2.79120
H	2.07700	-0.82710	-2.92880
H	0.77880	-1.74480	-3.73550
C	1.86070	-2.87780	-2.22650
H	2.36440	-2.70260	-1.27230
H	2.58890	-3.29280	-2.93130
H	1.06390	-3.61280	-2.07900
C	0.82130	3.34500	0.61510
H	0.97480	3.43290	-0.46130
H	1.63110	2.79220	1.09280

H 0.67120 4.31950 1.07990
26
start-3
G = -668.3319371

O	1.23380	6.22590	2.46610
O	2.63490	3.52930	1.16640
O	-0.32900	4.58580	2.39140
O	-0.69660	6.31940	-0.18390
N	0.30710	5.46520	-0.55840
C	0.93840	4.96780	0.44530
C	2.06190	4.01130	0.19340
C	2.43160	3.68600	-1.23540
H	2.66000	4.63280	-1.74100
H	1.53120	3.30450	-1.73280
C	3.59180	2.69950	-1.35270
H	3.80910	2.50670	-2.40720
H	3.35100	1.74730	-0.87090
H	4.49620	3.09570	-0.88160
C	0.63900	5.33890	1.88210
C	-0.72480	4.85810	3.77800
H	-1.03940	5.90340	3.83650
H	0.15500	4.71680	4.41110
C	-1.84390	3.89820	4.12030
H	-2.70370	4.04930	3.46080
H	-2.16380	4.07500	5.15190
H	-1.50870	2.86030	4.03510
C	-1.37360	6.84980	-1.34530
H	-2.14040	7.51220	-0.94320
H	-1.83160	6.03810	-1.91650
H	-0.67100	7.41170	-1.96600

26
start-2
G = -668.3312988

O	1.12960	5.87540	2.65990
O	2.70860	3.45600	1.05590
O	-0.33860	4.17590	2.31830
O	-0.73790	6.23070	-0.00470
N	0.31430	5.48220	-0.46230
C	0.94830	4.88490	0.48380
C	2.12440	4.02580	0.13880
C	2.53090	3.90000	-1.31150
H	2.71900	4.91250	-1.69060
H	1.65820	3.54130	-1.87120
C	3.74060	2.99230	-1.52610
H	3.98630	2.94780	-2.59100
H	3.53850	1.97550	-1.17700
H	4.61550	3.36590	-0.98610
C	0.59700	5.04500	1.94760
C	-0.80930	4.22880	3.70870
H	0.05180	4.40510	4.35640
H	-1.20010	3.22620	3.88390
C	-1.87900	5.29450	3.87110
H	-1.47390	6.29090	3.67610
H	-2.25610	5.26900	4.89880
H	-2.71560	5.11010	3.19080
C	-1.41720	6.88010	-1.10240
H	-1.82340	6.13210	-1.78820
H	-0.73100	7.55190	-1.62450

H	-2.22330	7.44660	-0.63590

26
start
G = -668.3309704

O	0.81370	6.19330	2.48490
O	2.47750	3.57270	1.29580
O	-0.62180	4.45850	2.18690
O	-0.75050	6.31480	-0.36420
N	0.31650	5.50080	-0.64040
C	0.83340	4.98120	0.41610
C	2.00650	4.06330	0.27370
C	2.54920	3.78480	-1.10900
H	2.80600	4.74980	-1.56370
H	1.72450	3.39130	-1.71620
C	3.74450	2.83360	-1.11090
H	4.08630	2.67000	-2.13690
H	3.47930	1.86480	-0.67760
H	4.57720	3.24440	-0.53240
C	0.34860	5.28870	1.81730
C	-1.19540	4.63210	3.52810
H	-2.18680	4.18670	3.44050
H	-1.29070	5.70210	3.72400
C	-0.34450	3.93000	4.57140
H	-0.24370	2.86630	4.33710
H	-0.82730	4.02510	5.54960
H	0.65100	4.37760	4.63230
C	-1.29500	6.87060	-1.58210
H	-2.12520	7.49750	-1.25630
H	-1.65430	6.06940	-2.23300
H	-0.53850	7.47330	-2.09130

26
start-8
G = -668.3303246

O	1.42490	5.90560	2.53250
O	2.35830	3.02710	1.21980
O	-0.41670	4.59080	2.39180
O	-0.42700	6.34720	-0.16820
N	0.43210	5.34490	-0.52690
C	0.95530	4.74800	0.48590
C	1.91870	3.63100	0.24410
C	2.34610	3.31580	-1.16930
H	1.48700	3.38310	-1.84240
H	2.71570	2.28680	-1.16860
C	3.45410	4.28180	-1.64120
H	3.77430	4.00660	-2.65000
H	4.32460	4.23180	-0.97960
H	3.09320	5.31380	-1.66450
C	0.69000	5.15320	1.91990
C	-0.79550	4.91640	3.77170
H	-0.90270	6.00200	3.84190
H	0.01980	4.59770	4.42650
C	-2.09020	4.18820	4.06300
H	-2.88260	4.51280	3.38220
H	-2.40260	4.40980	5.08820
H	-1.96070	3.10620	3.96700
C	-0.98470	6.98270	-1.34090
H	-1.55470	6.25630	-1.92560
H	-0.18660	7.42420	-1.94320

H	-1.64290	7.75940	-0.95140

26
start-9
G = -668.3302622

O	-0.92870	3.37100	1.59750
O	2.34180	2.98640	1.10260
O	0.18190	5.10560	2.54440
O	-0.77610	5.96430	-0.35060
N	0.40970	5.33070	-0.60330
C	0.75560	4.52480	0.33820
C	2.03890	3.77080	0.20600
C	2.93450	4.03240	-0.98110
H	2.33170	4.19310	-1.87870
H	3.54740	3.13770	-1.12070
C	3.83480	5.26120	-0.73030
H	4.50230	5.40670	-1.58430
H	4.44830	5.11980	0.16480
H	3.23660	6.16790	-0.60320
C	-0.09780	4.25960	1.55990
C	-0.58880	4.96220	3.78500
H	-0.42190	3.95200	4.16780
H	-1.64710	5.07600	3.53570
C	-0.10210	6.03080	4.74000
H	0.96260	5.90540	4.95760
H	-0.65900	5.95190	5.67890
H	-0.26470	7.02970	4.32450
C	-1.12330	6.84830	-1.44070
H	-2.07020	7.29940	-1.14390
H	-1.24520	6.27550	-2.36340
H	-0.35470	7.61610	-1.55990

26
start-5
G = -668.3295102

O	1.27790	5.53090	2.70080
O	2.40260	2.91090	1.03980
O	-0.49270	4.16930	2.28590
O	-0.48370	6.27460	0.01010
N	0.43820	5.37360	-0.44810
C	0.94840	4.66010	0.49350
C	1.97590	3.63310	0.14150
C	2.47890	3.55050	-1.27990
H	1.64470	3.66800	-1.97720
H	2.91040	2.55440	-1.40910
C	3.54280	4.63500	-1.55540
H	3.91730	4.52880	-2.57740
H	4.38890	4.53660	-0.86810
H	3.12050	5.63770	-1.44660
C	0.60390	4.84430	1.95570
C	-0.97110	4.27620	3.67060
H	-0.10500	4.26280	4.33530
H	-1.55360	3.36520	3.81020
C	-1.81480	5.52600	3.85010
H	-1.21930	6.42840	3.68930
H	-2.20840	5.54830	4.87160
H	-2.65870	5.52670	3.15400
C	-1.02500	7.05100	-1.08250
H	-1.53530	6.39490	-1.79230
H	-0.22680	7.61060	-1.57670

H -1.73560 7.73270 -0.61490
26
start-7
G = -668.3294552
O 1.01020 6.08190 2.64330
O 3.02970 3.83260 1.25380
O -0.12880 4.13880 2.34690
O -0.69720 6.05750 -0.10900
N 0.46340 5.43280 -0.47640
C 1.11270 4.95820 0.52810
C 2.40050 4.24190 0.28060
C 2.85230 4.00090 -1.13970
H 3.93470 3.84920 -1.10850
H 2.63470 4.87740 -1.75590
C 2.15610 2.75840 -1.73570
H 2.52870 2.58020 -2.74830
H 1.07320 2.90140 -1.78820
H 2.36160 1.86830 -1.13300
C 0.66370 5.13480 1.96270
C -0.67090 4.18390 3.71140
H 0.11220 4.54670 4.38030
H -0.88800 3.13900 3.93480
C -1.91760 5.04980 3.76280
H -1.68390 6.08940 3.51870
H -2.33450 5.01690 4.77480
H -2.67440 4.68100 3.06430
C -1.38170 6.58270 -1.26890
H -1.64280 5.76910 -1.95030
H -0.75390 7.32270 -1.77160
H -2.28200 7.05230 -0.87270
26
start-6
G = -668.3294378
O -1.03180 3.80080 1.80110
O 2.20280 3.07480 1.30170
O 0.29120 5.51660 2.48450
O -0.74630 6.03420 -0.49810
N 0.38550 5.28800 -0.68190
C 0.70810 4.60560 0.36000
C 1.93220 3.75030 0.31120
C 2.81230 3.78520 -0.91520
H 2.19660 3.84270 -1.81680
H 3.37610 2.84850 -0.92990
C 3.77800 4.98900 -0.86530
H 4.43080 4.97170 -1.74250
H 4.40550 4.94980 0.03040
H 3.22830 5.93450 -0.86480
C -0.11760 4.58490 1.62870
C -0.40730 5.61610 3.77310
H -1.47540 5.47460 3.59580
H -0.22140 6.64430 4.08460
C 0.14910 4.61020 4.76500
H -0.02990 3.58590 4.42800
H -0.34760 4.74880 5.73090
H 1.22400 4.75750 4.90420
C -1.06500 6.77760 -1.69640
H -0.25040 7.46480 -1.93890
H -1.97050 7.33180 -1.44870

H	-1.25210	6.09040	-2.52540

26
start-4
G = -668.3294196

O	-0.45000	3.50070	1.95540
O	2.79910	4.09530	1.57620
O	-0.03210	5.67870	2.44850
O	-0.95310	5.54860	-0.54670
N	0.38210	5.26600	-0.64180
C	0.87800	4.81970	0.45830
C	2.32960	4.46790	0.50320
C	3.14810	4.53760	-0.76380
H	4.19500	4.62960	-0.46250
H	2.86670	5.42200	-1.34210
C	2.94810	3.26950	-1.62140
H	3.58060	3.32620	-2.51180
H	1.90790	3.17230	-1.94430
H	3.22450	2.37080	-1.06120
C	0.05450	4.57970	1.70570
C	-0.80260	5.60170	3.69700
H	-1.69520	5.00060	3.51230
H	-1.09060	6.63690	3.88230
C	0.04860	5.03670	4.82020
H	0.34250	4.00520	4.60920
H	-0.53150	5.04860	5.74880
H	0.94820	5.64150	4.96630
C	-1.45750	6.04800	-1.80620
H	-0.94390	6.97470	-2.07480
H	-2.51620	6.23670	-1.62810
H	-1.32950	5.29390	-2.58680

26
start-10
G = -668.3273349

O	1.34800	5.78880	2.43640
O	2.04130	3.98060	-1.55560
O	-0.24730	4.18690	2.21700
O	-0.89660	6.29810	0.10370
N	0.06820	5.57530	-0.54730
C	0.82900	4.92540	0.26110
C	1.94010	4.09590	-0.34250
C	2.88670	3.43670	0.63370
H	2.28190	2.83590	1.32830
H	3.32790	4.23210	1.25000
C	3.96660	2.58780	-0.03130
H	4.61160	2.14060	0.73010
H	4.58760	3.19460	-0.69660
H	3.52340	1.78300	-0.62500
C	0.67670	5.02840	1.76260
C	-0.52440	4.18810	3.65940
H	0.42400	4.29070	4.19090
H	-0.93970	3.19620	3.83940
C	-1.50540	5.29120	4.01550
H	-1.07910	6.27680	3.81170
H	-1.74030	5.22930	5.08310
H	-2.43490	5.18060	3.44940
C	-1.71250	7.02520	-0.84100
H	-2.22300	6.32900	-1.51140
H	-1.09580	7.72710	-1.40840

H	-2.43590	7.56340	-0.22820

26
minima_3
G = -668.2614639

O	1.69050	5.92730	3.82520
O	2.62940	1.90660	3.45080
O	3.48200	4.53800	3.88370
O	-0.66030	4.28330	2.33560
N	0.59500	4.36060	1.81810
C	1.56960	3.86530	2.66000
C	1.89860	2.42110	2.60310
C	1.25440	1.61900	1.48700
H	1.49950	2.11290	0.53750
H	0.16570	1.71830	1.59120
C	1.66940	0.14990	1.47350
H	1.17650	-0.36910	0.64650
H	1.39130	-0.34770	2.40720
H	2.75160	0.04700	1.34770
C	2.24360	4.87620	3.52170
C	4.20810	5.46490	4.75280
H	4.01300	6.48550	4.41610
H	5.25450	5.21500	4.57370
C	3.81970	5.26100	6.20790
H	2.76500	5.49860	6.36940
H	4.42370	5.92350	6.83690
H	4.00420	4.22780	6.51610
C	-1.64610	4.76750	1.38770
H	-2.60230	4.65660	1.89810
H	-1.61360	4.15490	0.48390
H	-1.44760	5.81640	1.15710

26
minima_4-2
G = -668.2607014

O	1.86830	5.96630	3.61240
O	0.82730	1.89250	1.51950
O	3.37980	4.28910	3.81940
O	-0.78350	4.45280	2.51930
N	0.39390	4.51060	1.85020
C	1.44360	3.90520	2.51550
C	1.65520	2.45990	2.24160
C	2.82390	1.70800	2.84290
H	2.80290	1.85570	3.92890
H	3.74480	2.20210	2.51160
C	2.83130	0.22310	2.48550
H	3.69410	-0.26350	2.94980
H	2.89410	0.07540	1.40330
H	1.92360	-0.27500	2.83930
C	2.24310	4.82510	3.36610
C	4.21000	5.09900	4.71000
H	4.21780	6.12660	4.34050
H	5.20350	4.66290	4.59880
C	3.71000	5.00990	6.14220
H	2.70740	5.43560	6.23450
H	4.38650	5.57210	6.79440
H	3.68880	3.96940	6.47940
C	-1.84650	5.09290	1.76760
H	-2.73460	4.98070	2.38870
H	-1.97440	4.58370	0.80980

```
H          -1.60570         6.14810         1.62070
26
minima_4-3
G = -668.2604848
O           1.71500         5.87040         3.86870
O           0.96470         1.97420         1.34770
O           2.96210         4.04970         4.38630
O          -0.78940         4.47730         2.28270
N           0.48720         4.55770         1.83430
C           1.39640         3.87520         2.62190
C           1.66040         2.46100         2.24680
C           2.72750         1.64630         2.94700
H           2.50070         1.64080         4.01950
H           3.67500         2.19160         2.86480
C           2.85380         0.22560         2.40130
H           3.63940        -0.30890         2.94350
H           3.11120         0.23040         1.33780
H           1.91780        -0.32880         2.51650
C           2.03680         4.70280         3.67700
C           3.64470         4.77930         5.45390
H           3.99130         3.98930         6.12150
H           2.91330         5.40210         5.97320
C           4.79880         5.59660         4.89820
H           5.50500         4.95410         4.36410
H           5.32770         6.07980         5.72640
H           4.43760         6.37280         4.21850
C          -1.69690         5.20580         1.41610
H          -2.68030         5.07120         1.86530
H          -1.66560         4.77730         0.41190
H          -1.41780         6.26160         1.40000
26
minima_4-6
G = -668.2593510
O           2.04830         5.85350         3.39920
O           0.48100         1.70860         1.82520
O           2.75610         3.98320         4.46710
O          -0.82560         4.57090         2.27430
N           0.42050         4.38670         1.77370
C           1.27740         3.73610         2.64270
C           1.35520         2.25890         2.50720
C           2.47580         1.45210         3.12610
H           2.20050         0.40140         3.00260
H           2.54700         1.67160         4.19360
C           3.82970         1.73400         2.44470
H           3.78420         1.50510         1.37530
H           4.60490         1.10770         2.89620
H           4.12940         2.77820         2.56450
C           2.06680         4.63490         3.52560
C           3.60020         4.77080         5.36530
H           3.70040         4.13420         6.24510
H           3.06640         5.68490         5.63350
C           4.94490         5.06120         4.72000
H           5.45020         4.13110         4.44460
H           5.57610         5.60140         5.43310
H           4.82550         5.67960         3.82630
C          -1.67750         5.26010         1.32280
H          -2.63760         5.35810         1.82830
H          -1.77460         4.65770         0.41700
```

H	-1.25390	6.24120	1.09740

26
minima_4-7
G = -668.2592634

O	1.65020	5.69220	3.94970
O	1.10530	2.10460	0.96480
O	3.42760	4.31560	3.65630
O	-0.80030	4.24770	2.41280
N	0.38060	4.54240	1.81670
C	1.46790	3.91170	2.39170
C	1.80410	2.56040	1.87990
C	2.90170	1.72230	2.50020
H	3.82710	2.29950	2.55490
H	3.05540	0.87470	1.82720
C	2.51480	1.22010	3.90620
H	3.31490	0.58980	4.30520
H	1.59660	0.62560	3.86860
H	2.36250	2.04970	4.60140
C	2.18100	4.72800	3.41120
C	4.18910	5.01470	4.69180
H	3.99060	6.08510	4.60690
H	5.22680	4.81270	4.42420
C	3.84490	4.47610	6.07020
H	2.79530	4.66180	6.31270
H	4.46700	4.97960	6.81740
H	4.03860	3.40100	6.12520
C	-1.90360	4.91520	1.74740
H	-2.79080	4.60460	2.29820
H	-1.95530	4.58560	0.70740
H	-1.76680	5.99710	1.80880

26
minima_4-8
G = -668.2591776

O	1.79060	5.85010	3.69860
O	0.66270	1.77470	1.64950
O	3.38100	4.23340	3.63430
O	-0.86990	4.49250	2.45080
N	0.33240	4.44480	1.82760
C	1.31670	3.81690	2.56690
C	1.42700	2.34670	2.43900
C	2.41580	1.54110	3.25640
H	1.92630	0.58430	3.46210
H	2.63620	2.03210	4.20570
C	3.71980	1.30010	2.46950
H	4.39800	0.67770	3.06080
H	4.22430	2.24460	2.25010
H	3.51290	0.78370	1.52690
C	2.17520	4.73700	3.36140
C	4.28190	5.02220	4.47410
H	4.19480	6.07250	4.18840
H	5.27140	4.65070	4.20530
C	3.97300	4.79960	5.94510
H	2.97040	5.15830	6.19350
H	4.69820	5.35070	6.55270
H	4.04440	3.73760	6.19730
C	-1.86840	5.13720	1.61820
H	-2.78450	5.10640	2.20710
H	-1.98420	4.57730	0.68760

H -1.56800 6.16840 1.42030
26
minima_4-9
G = -668.2588957
O 1.58660 5.66080 4.04750
O 1.18150 2.18690 0.91030
O 3.01200 3.91310 4.26570
O -0.78350 4.32330 2.23540
N 0.47630 4.59290 1.81670
C 1.45180 3.87710 2.48660
C 1.83980 2.57750 1.88450
C 2.95100 1.71660 2.44430
H 3.79970 2.33340 2.74460
H 3.26280 1.05730 1.62940
C 2.46270 0.87490 3.64120
H 3.27590 0.23600 3.99830
H 1.62570 0.23310 3.34820
H 2.14230 1.51330 4.46860
C 2.02080 4.57900 3.66830
C 3.63000 4.51630 5.44560
H 4.05140 3.66200 5.97700
H 2.84700 4.97540 6.05280
C 4.70040 5.51610 5.04160
H 5.45880 5.03730 4.41520
H 5.18840 5.90320 5.94220
H 4.26510 6.35690 4.49530
C -1.76160 5.09390 1.49020
H -2.72430 4.79960 1.90710
H -1.70020 4.83430 0.43100
H -1.58050 6.16000 1.64360
26
reactant_is1-5
G = -668.2420309
O 2.57420 5.34860 3.52610
O 2.83060 3.25200 2.81370
O 0.92870 6.72690 2.85230
O -0.70020 5.69900 1.05250
N -0.01710 4.50030 1.08340
C 1.04600 4.51010 1.94120
C 1.81010 3.33140 2.03350
C 1.52860 2.08870 1.20530
H 0.44170 1.97090 1.15720
H 1.94880 1.22440 1.72650
C 2.12310 2.21020 -0.20850
H 1.89910 1.30100 -0.77550
H 3.20980 2.33050 -0.16870
H 1.69410 3.06400 -0.73940
C 1.56380 5.56350 2.82490
C 1.46090 7.75910 3.74650
H 2.49300 7.96570 3.45040
H 1.45700 7.36160 4.76500
C 0.56540 8.97070 3.59830
H 0.57300 9.33870 2.56800
H 0.92960 9.76820 4.25340
H -0.46390 8.73260 3.88260
C -1.79450 5.59660 0.12670
H -2.28560 6.57070 0.15130
H -2.49110 4.81360 0.44350

H	-1.42350	5.38540	-0.88140

26
reactant_is1-3
G = -668.2408090

O	2.16770	5.40960	3.75220
O	2.62040	3.34360	3.05630
O	0.57160	6.73090	2.86390
O	-0.73200	5.66410	0.83530
N	-0.00380	4.49800	0.95070
C	0.92450	4.54040	1.95190
C	1.72150	3.39490	2.13720
C	1.62070	2.15750	1.26030
H	0.55830	1.99000	1.05780
H	2.00580	1.30360	1.82370
C	2.40160	2.33580	-0.05330
H	2.30360	1.42930	-0.65900
H	3.46480	2.50460	0.14170
H	2.01090	3.18010	-0.62730
C	1.26060	5.59810	2.91550
C	0.92050	7.79000	3.81760
H	1.07630	7.33820	4.79970
H	0.02280	8.40870	3.83960
C	2.13380	8.57200	3.34530
H	3.02280	7.93690	3.31130
H	2.32260	9.39370	4.04390
H	1.95780	8.99580	2.35220
C	-1.67820	5.52700	-0.23780
H	-2.21400	6.47680	-0.27360
H	-2.37400	4.70750	-0.03100
H	-1.15840	5.34970	-1.18480

26
reactant_is1-2
G = -668.2407406

O	2.62140	5.36110	3.33470
O	2.93120	3.41050	2.35370
O	0.87450	6.72380	2.94640
O	-0.84480	5.81500	1.15850
N	-0.10100	4.66360	0.99120
C	1.02330	4.63930	1.76580
C	1.85740	3.50590	1.65610
C	1.53750	2.35180	0.71900
H	1.43870	2.78590	-0.28340
H	0.53730	1.99640	0.99510
C	2.55310	1.20820	0.72850
H	2.23200	0.43520	0.02480
H	2.63500	0.75440	1.72030
H	3.54640	1.55320	0.42730
C	1.55110	5.60400	2.73190
C	1.43530	7.67060	3.91520
H	2.43170	7.95950	3.56970
H	1.52800	7.15880	4.87690
C	0.48490	8.84690	3.97880
H	0.39580	9.33050	3.00150
H	0.87020	9.58040	4.69400
H	-0.50820	8.52940	4.31000
C	-2.00450	5.75680	0.31240
H	-2.53980	6.69060	0.49150
H	-2.63320	4.90250	0.58370

```
H          -1.70750        5.68670        -0.73900
26
reactant_is1-4
G = -668.2400141
O           2.54270        5.62300         3.34800
O           2.79810        3.45190         2.90720
O           0.90420        6.91080         2.48960
O          -0.73020        5.65770         0.84850
N          -0.04240        4.47510         1.02660
C           1.01880        4.59410         1.87780
C           1.78200        3.43600         2.11810
C           1.50550        2.10090         1.44620
H           0.41900        1.97560         1.40990
H           1.92620        1.30750         2.06960
C           2.10470        2.05160         0.03010
H           1.88460        1.07980        -0.42300
H           3.19100        2.17850         0.05870
H           1.67560        2.83380        -0.60150
C           1.53620        5.75170         2.62180
C           1.40780        8.05700         3.25470
H           1.00230        8.91320         2.71450
H           2.49800        8.06840         3.19110
C           0.91640        8.01060         4.69110
H          -0.17660        7.97720         4.72460
H           1.25140        8.91330         5.21270
H           1.31750        7.13990         5.21640
C          -1.82520        5.43610        -0.05530
H          -2.32030        6.40370        -0.15160
H          -2.51800        4.69600         0.35810
H          -1.45450        5.10200        -1.02970
26
reactant_is1
G = -668.2394007
O           2.57360        5.63970         3.17340
O           2.90620        3.58610         2.43890
O           0.81380        6.93040         2.60760
O          -0.88760        5.78800         0.93720
N          -0.13190        4.63190         0.92590
C           0.98860        4.71650         1.70130
C           1.83160        3.58460         1.73670
C           1.51950        2.31690         0.95480
H           1.41290        2.61980        -0.09400
H           0.52280        1.99220         1.27810
C           2.54430        1.19180         1.10410
H           2.22650        0.33350         0.50560
H           2.63380        0.86820         2.14490
H           3.53340        1.50340         0.75620
C           1.50440        5.79870         2.54210
C           1.32860        8.00150         3.46650
H           0.84030        8.89250         3.07030
H           2.40780        8.08130         3.31740
C           0.96630        7.75920         4.92200
H          -0.11710        7.66320         5.04050
H           1.30620        8.61130         5.51980
H           1.44930        6.85560         5.30280
C          -2.03630        5.61180         0.09190
H          -2.58240        6.55490         0.14650
H          -2.65990        4.79100         0.46070
```

H -1.72500 5.41320 -0.93880
26
reactant_is2-3
G = -668.2362720

O	-0.26460	6.81100	2.51560
O	2.38190	3.86140	3.31060
O	1.83360	6.26680	3.15380
O	-0.79240	5.39330	0.24340
N	-0.06230	4.34210	0.75630
C	0.70900	4.70990	1.81660
C	1.55880	3.71950	2.32330
C	1.63350	2.30040	1.73540
H	0.59950	2.02070	1.50810
H	2.01300	1.61990	2.50200
C	2.51790	2.26450	0.48270
H	2.53530	1.24220	0.08960
H	3.54570	2.55790	0.71540
H	2.12520	2.92950	-0.29020
C	0.67790	6.03520	2.50190
C	1.88910	7.43110	4.03100
H	1.07080	7.35450	4.75240
H	1.73310	8.32230	3.41610
C	3.24840	7.42500	4.69890
H	3.38640	6.51610	5.29230
H	3.32670	8.28950	5.36540
H	4.04890	7.48720	3.95570
C	-1.53450	4.94360	-0.90240
H	-2.08010	5.82040	-1.25450
H	-2.23500	4.15220	-0.61730
H	-0.85430	4.58270	-1.68060

26
reactant_is2
G = -668.2359271

O	-0.41590	6.30610	3.00820
O	3.07020	4.29750	2.57640
O	1.83900	6.36800	3.12910
O	-1.05480	5.22060	0.58740
N	0.02510	4.37230	0.71840
C	0.90930	4.79540	1.66270
C	2.10150	4.06580	1.75370
C	2.38280	2.81050	0.90790
H	3.46300	2.64960	0.86700
H	2.03230	3.05590	-0.10020
C	1.66520	1.57890	1.47300
H	1.87860	0.71820	0.82980
H	0.58410	1.73400	1.50020
H	2.01650	1.34550	2.48220
C	0.67470	5.89430	2.64610
C	1.77170	7.31460	4.23760
H	1.20020	6.85190	5.04710
H	1.23360	8.20110	3.89000
C	3.19550	7.63120	4.64550
H	3.71690	6.72730	4.97460
H	3.18240	8.34520	5.47500
H	3.74980	8.07690	3.81420
C	-1.90780	4.74440	-0.46650
H	-2.72950	5.46050	-0.51780
H	-2.29030	3.74700	-0.22730

H	-1.36510	4.72170	-1.41710

26
reactant_is2-4
G = -668.2352916

O	-0.11750	6.67090	2.84050
O	2.49560	3.61170	3.21240
O	1.98640	6.00990	3.35160
O	-0.78350	5.50130	0.47200
N	-0.04450	4.39510	0.83450
C	0.77850	4.63620	1.89260
C	1.63000	3.58460	2.25210
C	1.65560	2.23450	1.51810
H	0.61060	2.00350	1.28650
H	2.03380	1.46930	2.20100
C	2.51460	2.30750	0.24910
H	2.49800	1.33140	-0.24770
H	3.55390	2.55020	0.48900
H	2.12570	3.05850	-0.44250
C	0.80370	5.88030	2.71570
C	2.12810	7.08270	4.33220
H	2.88790	6.70920	5.02050
H	1.17940	7.19630	4.86140
C	2.56270	8.37210	3.65610
H	3.50190	8.22740	3.11380
H	2.71840	9.14270	4.41850
H	1.79810	8.72330	2.95840
C	-1.57890	5.18180	-0.68170
H	-2.13030	6.09440	-0.91320
H	-2.27430	4.36800	-0.45260
H	-0.93680	4.90300	-1.52340

26
reactant_is2-5
G = -668.2347924

O	0.02890	6.94310	2.44320
O	2.59630	3.91440	3.16250
O	2.16320	6.32040	2.87640
O	-0.83470	5.40050	0.36110
N	-0.10430	4.34830	0.87160
C	0.80640	4.74630	1.80260
C	1.65930	3.74720	2.28760
C	1.59460	2.28710	1.81050
H	0.52890	2.05570	1.71070
H	2.01550	1.64330	2.58710
C	2.34030	2.10350	0.48250
H	2.25940	1.05620	0.17160
H	3.40140	2.34680	0.58820
H	1.90800	2.73390	-0.29810
C	0.92330	6.11510	2.38410
C	2.39120	7.50820	3.69390
H	1.87690	8.35050	3.22500
H	3.46950	7.66110	3.63480
C	1.92680	7.28050	5.12250
H	0.84730	7.11100	5.16070
H	2.15900	8.16600	5.72340
H	2.43850	6.41890	5.56170
C	-1.72630	4.91190	-0.65470
H	-2.26340	5.79130	-1.01340
H	-2.42850	4.18740	-0.23020

H -1.16030 4.45380 -1.47220
26
reactant_is2-2
G = -668.2328228
O -0.06860 6.92570 2.58830
O 2.73760 4.05030 2.68920
O 2.11490 6.36650 2.81250
O -1.06740 5.55420 0.45050
N -0.19870 4.52010 0.73110
C 0.76770 4.87200 1.62330
C 1.73020 3.88700 1.89480
C 1.67210 2.49180 1.25160
H 1.83230 2.65670 0.17910
H 0.63710 2.15510 1.37400
C 2.65960 1.46190 1.80340
H 2.51120 0.51530 1.27650
H 2.50050 1.28580 2.87110
H 3.69670 1.77490 1.65550
C 0.85380 6.15740 2.36860
C 2.33350 7.41810 3.80120
H 1.75170 8.29310 3.50100
H 3.39750 7.64060 3.71310
C 1.96540 6.93830 5.19480
H 0.89980 6.70140 5.25900
H 2.18730 7.72860 5.91940
H 2.54480 6.04960 5.46190
C -2.01820 5.11890 -0.53520
H -2.66640 5.97900 -0.71010
H -2.60370 4.27560 -0.15500
H -1.50680 4.83540 -1.46080
26
pr1-5
G = -668.2440015
O 3.78330 3.83510 3.61650
O 3.26010 2.37390 1.98500
O 2.38320 5.47360 4.27260
O -0.36970 4.26740 1.59620
N 0.53470 4.73310 2.52790
C 1.68000 4.00600 2.53870
C 2.09280 2.89410 1.77450
C 1.29680 2.15250 0.70570
H 2.01080 1.59630 0.09190
H 0.80530 2.89280 0.07280
C 0.26750 1.19240 1.32520
H -0.25670 0.65990 0.52470
H -0.46810 1.74250 1.91630
H 0.75620 0.45190 1.96570
C 2.69770 4.42920 3.51590
C 3.37940 5.91550 5.25300
H 4.29440 6.17430 4.71330
H 3.58850 5.07880 5.92480
C 2.78560 7.10260 5.98060
H 2.56920 7.91910 5.28550
H 3.50400 7.46210 6.72400
H 1.86330 6.82250 6.49810
C -1.56860 5.05840 1.64380
H -1.34480 6.10390 1.41010
H -2.03430 4.98280 2.63140

H -2.22500 4.63310 0.88320
26
pr1-2
G = -668.2433549

O	3.65410	3.72930	3.65820
O	2.92990	2.21720	2.19380
O	2.44530	5.54010	4.23940
O	-0.52490	4.39710	1.76300
N	0.45160	4.84760	2.63100
C	1.52800	4.01960	2.66000
C	1.80510	2.81470	1.97490
C	0.88810	2.12050	0.97040
H	0.67380	2.84570	0.17940
H	-0.06340	1.93800	1.47920
C	1.45420	0.82420	0.38640
H	0.73010	0.40340	-0.31700
H	1.64380	0.08100	1.16630
H	2.38990	1.00060	-0.15190
C	2.62140	4.41680	3.55470
C	3.52390	5.95570	5.14140
H	4.43280	6.08220	4.54730
H	3.68370	5.15620	5.86980
C	3.07860	7.24690	5.79360
H	2.90860	8.02520	5.04380
H	3.86060	7.59050	6.47790
H	2.15840	7.09840	6.36650
C	-1.63840	5.30530	1.77190
H	-1.32150	6.29970	1.44190
H	-2.07620	5.36120	2.77360
H	-2.35890	4.88580	1.06810

26
pr1-3
G = -668.2433323

O	3.73320	4.11840	3.62300
O	3.26080	2.44390	2.19630
O	2.30270	5.82190	4.00870
O	-0.38070	4.21000	1.48380
N	0.49690	4.81420	2.36030
C	1.65110	4.11300	2.49250
C	2.09160	2.91260	1.89650
C	1.32680	2.01850	0.92620
H	2.06090	1.39880	0.40370
H	0.83280	2.65660	0.19210
C	0.30560	1.13020	1.65590
H	-0.19620	0.48430	0.92780
H	-0.44890	1.73990	2.15810
H	0.79770	0.49260	2.39660
C	2.64550	4.68160	3.41980
C	3.24720	6.42060	4.95790
H	2.94320	7.46760	4.98490
H	4.25450	6.34420	4.54310
C	3.13600	5.76450	6.32280
H	2.11320	5.83650	6.70410
H	3.80300	6.27950	7.02210
H	3.42810	4.71200	6.27860
C	-1.59090	4.98000	1.39650
H	-1.37610	5.98620	1.02340
H	-2.07720	5.03510	2.37550

H	-2.22440	4.44200	0.68980

26
pr1-4
G = -668.2427129

O	3.50290	3.84530	4.00610
O	3.20990	2.36860	2.33290
O	2.03210	5.50390	4.43190
O	-0.29840	4.29150	1.38710
N	0.46110	4.76070	2.43880
C	1.58500	4.02410	2.62750
C	2.09480	2.90030	1.94370
C	1.46150	2.15720	0.77200
H	2.25510	1.59260	0.27470
H	1.07560	2.89680	0.06900
C	0.34460	1.20730	1.23540
H	-0.05800	0.67340	0.36830
H	-0.46710	1.76540	1.70740
H	0.72710	0.46750	1.94530
C	2.45120	4.44940	3.74130
C	2.86700	5.97900	5.54030
H	3.23070	5.11360	6.09850
H	2.16560	6.54140	6.15750
C	4.00000	6.85310	5.03210
H	4.68610	6.28100	4.40210
H	4.56060	7.24450	5.88740
H	3.60850	7.69880	4.45900
C	-1.48110	5.09510	1.24320
H	-1.21190	6.13520	1.03460
H	-2.09240	5.03680	2.14920
H	-2.01930	4.66670	0.39640

26
pr1
G = -668.2415771

O	3.39130	3.78240	4.01780
O	2.87430	2.24610	2.48760
O	2.10620	5.59830	4.40350
O	-0.47970	4.42440	1.54670
N	0.36750	4.88580	2.53610
C	1.42650	4.05750	2.72890
C	1.79230	2.84170	2.10720
C	1.01910	2.13110	0.99810
H	0.92240	2.84090	0.17070
H	0.00470	1.96350	1.37260
C	1.65140	0.82060	0.52370
H	1.02900	0.38890	-0.26520
H	1.72520	0.09250	1.33670
H	2.65420	0.98160	0.11730
C	2.38420	4.46780	3.76360
C	3.04530	6.06050	5.43150
H	3.35570	5.20220	6.03140
H	2.43730	6.72950	6.04130
C	4.22550	6.78200	4.80480
H	4.81690	6.10410	4.18380
H	4.86910	7.17250	5.59990
H	3.88400	7.62200	4.19270
C	-1.57760	5.33720	1.38490
H	-1.21130	6.32400	1.08450
H	-2.14940	5.41380	2.31520

H -2.19650 4.90790 0.59570
26
pr2-3
G = -668.2373604

O	1.69390	6.46430	3.56450
O	2.72830	2.52930	3.39930
O	3.06330	4.76390	4.12690
O	-0.49600	3.88870	1.29410
N	0.21610	4.80850	2.02760
C	1.26720	4.26030	2.68600
C	1.70720	2.92410	2.69770
C	1.16170	1.72550	1.90260
H	0.07470	1.74110	1.99400
H	1.52620	0.81330	2.38450
C	1.61360	1.76670	0.43650
H	2.70510	1.75470	0.36050
H	1.23140	2.65890	-0.06340
H	1.22580	0.88150	-0.07930
C	2.00390	5.28390	3.48850
C	3.86660	5.65580	4.95740
H	3.21270	6.08710	5.72030
H	4.24460	6.46010	4.32030
C	4.98220	4.82610	5.55850
H	4.57870	4.01890	6.17700
H	5.60650	5.46690	6.18890
H	5.61120	4.39180	4.77570
C	-1.57560	4.54230	0.60500
H	-1.18300	5.28800	-0.09310
H	-2.25390	5.01410	1.32250
H	-2.09110	3.74960	0.06110

26
pr2-4
G = -668.2370725

O	1.66510	6.21410	3.96930
O	2.72410	2.34340	3.30120
O	3.02040	4.44560	4.34120
O	-0.45440	3.96880	1.31690
N	0.23400	4.78070	2.18750
C	1.27030	4.14920	2.79270
C	1.71710	2.82490	2.63390
C	1.19560	1.74340	1.67330
H	0.10660	1.74620	1.73920
H	1.55160	0.77540	2.03840
C	1.68190	1.97870	0.23670
H	2.77500	1.97780	0.18580
H	1.30800	2.92880	-0.14990
H	1.30990	1.16940	-0.40100
C	1.98110	5.05470	3.74600
C	3.83190	5.20720	5.28760
H	4.27760	4.44010	5.92220
H	3.16420	5.83220	5.88490
C	4.88550	6.02460	4.56050
H	5.52600	5.37790	3.95340
H	5.51190	6.53990	5.29630
H	4.42130	6.77470	3.91480
C	-1.51920	4.70930	0.69590
H	-1.11250	5.54420	0.11690
H	-2.21780	5.07770	1.45340

H -2.01710 3.99800 0.03550
26
pr2-5
G = -668.2370230

O	1.97260	6.36900	3.54660
O	2.87100	2.42060	3.14350
O	3.34210	4.60940	3.90680
O	-0.53030	3.93190	1.45680
N	0.28140	4.80470	2.14280
C	1.38400	4.20740	2.66010
C	1.78340	2.86070	2.58160
C	1.10710	1.70000	1.83320
H	0.03930	1.75120	2.05070
H	1.49110	0.76560	2.25330
C	1.38670	1.75650	0.32500
H	2.46060	1.70850	0.12160
H	0.98190	2.67170	-0.11140
H	0.90930	0.89660	-0.15720
C	2.23560	5.18380	3.40550
C	4.26220	5.42820	4.69280
H	4.34370	6.40620	4.21310
H	5.21350	4.90110	4.60910
C	3.79600	5.53380	6.13440
H	2.83270	6.04680	6.19690
H	4.53160	6.10630	6.70910
H	3.70210	4.54100	6.58440
C	-1.66300	4.63660	0.91980
H	-1.33150	5.40590	0.21570
H	-2.24760	5.08780	1.72730
H	-2.25340	3.87940	0.40220

26
pr2
G = -668.2353623

O	1.54190	6.41130	3.86920
O	2.86220	2.69540	2.94100
O	3.03430	4.73620	4.08790
O	-0.54920	4.13520	1.21100
N	0.11400	4.94790	2.10260
C	1.23090	4.37490	2.61710
C	1.77010	3.09790	2.36190
C	1.20060	2.02470	1.41430
H	1.13770	2.48720	0.42540
H	0.17510	1.83470	1.74210
C	2.00420	0.72210	1.35680
H	1.51220	0.03890	0.65890
H	2.05320	0.23260	2.33370
H	3.02570	0.89060	1.00400
C	1.92350	5.28740	3.57360
C	3.81580	5.52180	5.03810
H	3.16600	5.77580	5.88000
H	4.12460	6.44410	4.53840
C	4.99730	4.67250	5.45890
H	4.66250	3.74880	5.94010
H	5.60650	5.23450	6.17360
H	5.62120	4.41720	4.59730
C	-1.71630	4.81150	0.71230
H	-1.42910	5.72820	0.18810
H	-2.40110	5.04340	1.53380

H -2.18130 4.10920 0.01900
26
pr2-2
G = -668.2334971

O	1.85310	6.36210	3.79070
O	2.94630	2.61380	2.71810
O	3.31990	4.64390	3.81630
O	-0.59790	4.16010	1.39250
N	0.18900	4.95050	2.19990
C	1.34090	4.34280	2.58050
C	1.80670	3.04980	2.26780
C	1.09670	1.99650	1.39690
H	0.92700	2.46320	0.42270
H	0.11320	1.83430	1.84640
C	1.85000	0.67190	1.24240
H	2.00360	0.17800	2.20610
H	2.82530	0.81300	0.76790
H	1.25760	0.00520	0.60950
C	2.16760	5.23130	3.44840
C	4.22890	5.36480	4.70380
H	4.23700	6.41490	4.40310
H	5.20240	4.91880	4.49570
C	3.82030	5.18580	6.15550
H	2.83510	5.62160	6.34080
H	4.54910	5.68970	6.79900
H	3.79770	4.12510	6.42290
C	-1.79650	4.87030	1.03600
H	-1.54800	5.78150	0.48320
H	-2.37320	5.11660	1.93290
H	-2.35930	4.18450	0.40120

26
ts1-3
G = -668.2616985

O	2.08500	5.45260	4.25920
O	2.65200	2.90590	3.07050
O	1.30200	6.88970	2.69380
O	-0.79670	4.87210	1.23190
N	0.54690	4.97890	1.04660
C	1.29090	4.61660	2.14830
C	1.82910	3.23480	2.21560
C	1.31140	2.26300	1.16980
H	1.54210	2.68540	0.18250
H	0.21520	2.25580	1.23040
C	1.87930	0.85290	1.31170
H	1.47400	0.20740	0.52710
H	1.62080	0.41880	2.28210
H	2.97000	0.85660	1.22570
C	1.61970	5.66730	3.15020
C	1.52740	8.01020	3.60120
H	2.58900	8.02930	3.86420
H	0.94580	7.83570	4.51090
C	1.09380	9.26720	2.87520
H	1.67930	9.41350	1.96260
H	1.24910	10.13170	3.52810
H	0.03310	9.21950	2.61100
C	-1.54700	5.27220	0.05250
H	-2.18610	6.10790	0.34410
H	-2.14980	4.41500	-0.25290

```
H          -0.85720         5.56600        -0.73870
26
ts1mod_opt_str
G = -668.2411729
O          -0.04610         5.95140         3.60070
O           2.26800         3.46440         3.57100
O           1.86440         6.54150         2.52610
O          -0.45970         5.54650         0.57970
N           0.12130         4.33850         0.76740
C           0.88840         4.46290         2.00360
C           1.62770         3.34160         2.49530
C           1.60560         2.05260         1.69700
H           1.94560         2.27920         0.67710
H           0.55640         1.74550         1.58470
C           2.44000         0.93420         2.31700
H           2.38240         0.03540         1.69600
H           2.08110         0.68360         3.31990
H           3.49110         1.22670         2.40220
C           0.84820         5.73260         2.80130
C           1.94700         7.81160         3.26240
H           0.93940         8.22360         3.34860
H           2.54690         8.44500         2.60850
C           2.60250         7.60510         4.61630
H           1.99680         6.95250         5.25040
H           2.70520         8.57450         5.11500
H           3.59830         7.16730         4.50160
C          -1.24340         5.57600        -0.63620
H          -1.64950         6.58500        -0.69090
H          -2.04090         4.83240        -0.56470
H          -0.59110         5.36410        -1.48670
26
ts1-4
G = -668.2400816
O           2.64850         5.65380         3.54800
O           3.04600         3.36510         3.00470
O           1.10880         6.86550         2.41900
O          -0.72920         5.24650         1.21910
N           0.39740         4.51470         0.90860
C           1.32980         4.55610         1.91830
C           2.11780         3.40670         2.13840
C           1.90060         2.13330         1.32190
H           2.83020         1.55950         1.33160
H           1.67880         2.43610         0.29410
C           0.74080         1.30940         1.90490
H           0.59560         0.41270         1.29360
H          -0.18950         1.88390         1.89810
H           0.95490         0.99580         2.93080
C           1.74750         5.73130         2.70020
C           1.48330         8.06420         3.17190
H           1.17020         8.87930         2.51820
H           2.57000         8.08150         3.28350
C           0.77040         8.11120         4.51230
H          -0.31420         8.07100         4.37490
H           1.02090         9.04920         5.01890
H           1.08080         7.28020         5.15130
C          -1.65790         5.20650         0.12130
H          -2.52650         5.77370         0.45850
H          -1.93980         4.17210        -0.09850
```

H	-1.21880	5.67600	-0.76420

26
ts1-5
G = -668.2399538

O	2.10430	5.49010	4.01010
O	2.75250	3.28740	3.32800
O	0.74860	6.75310	2.71390
O	-0.68120	5.20630	0.98280
N	0.53610	4.57340	0.84520
C	1.22670	4.52990	2.03320
C	2.02300	3.39460	2.29440
C	2.05660	2.21610	1.32150
H	2.99070	1.67160	1.47820
H	2.04760	2.62660	0.30720
C	0.84300	1.29870	1.54470
H	0.88220	0.47260	0.82720
H	-0.09290	1.84270	1.39120
H	0.84570	0.87820	2.55460
C	1.39660	5.62550	3.00280
C	0.91040	7.88680	3.62760
H	0.84630	7.52040	4.65440
H	0.04210	8.51010	3.41050
C	2.21300	8.62150	3.36060
H	3.07560	7.98260	3.56760
H	2.26890	9.49900	4.01350
H	2.25970	8.96020	2.32140
C	-1.34510	5.27500	-0.29150
H	-2.30820	5.74540	-0.08890
H	-1.48990	4.26890	-0.69730
H	-0.76400	5.88560	-0.98930

26
ts1-2
G = -668.2383315

O	1.96370	5.41760	4.01510
O	2.28630	3.14780	3.41700
O	0.80250	6.82920	2.68510
O	-0.69690	5.51290	0.85490
N	0.38610	4.66070	0.81170
C	1.01690	4.55640	2.02640
C	1.62650	3.31750	2.34900
C	1.54330	2.14490	1.37070
H	1.92260	2.50710	0.40830
H	0.47060	1.96310	1.23030
C	2.27120	0.87750	1.81430
H	2.13380	0.10110	1.05620
H	1.87990	0.50250	2.76440
H	3.34400	1.05370	1.93180
C	1.30050	5.63140	2.98810
C	1.08890	7.93980	3.59670
H	0.96950	7.58960	4.62410
H	0.30420	8.66140	3.36650
C	2.47310	8.51120	3.34120
H	3.25050	7.77420	3.55930
H	2.62790	9.37850	3.99160
H	2.57060	8.83680	2.30140
C	-1.29360	5.61380	-0.45000
H	-2.14270	6.28750	-0.32610
H	-1.63380	4.63030	-0.78900

H	-0.57760	6.03390	-1.16290

26
ts2_xyz-9
G = -668.2429424

O	3.83200	3.60630	3.90640
O	2.81060	1.64760	2.86180
O	3.02680	5.65010	3.38520
O	-0.25700	4.17600	1.89880
N	1.02160	4.67820	1.98500
C	1.90580	3.77140	2.50420
C	1.93330	2.36680	2.28990
C	0.93340	1.60700	1.41820
H	1.49700	0.80610	0.93100
H	0.56700	2.28650	0.64720
C	-0.22760	1.01590	2.23190
H	-0.87490	0.43370	1.56810
H	-0.82110	1.80890	2.69210
H	0.14280	0.34920	3.01640
C	3.00210	4.31420	3.33200
C	4.09700	6.26550	4.16880
H	5.05380	5.98990	3.71670
H	4.05700	5.85860	5.18300
C	3.86040	7.76110	4.14070
H	3.88870	8.14290	3.11580
H	4.64580	8.25980	4.71730
H	2.89270	8.01190	4.58550
C	-1.11040	5.09360	1.18930
H	-0.76770	5.20460	0.15630
H	-1.12150	6.06410	1.69390
H	-2.10150	4.63950	1.21500

26
ts2_xyz-8
G = -668.2424393

O	3.45110	3.71250	4.46700
O	2.08900	1.73020	3.98370
O	3.17520	5.54310	3.17410
O	-0.20250	4.24620	1.72420
N	1.13670	4.52010	1.87610
C	1.74100	3.71060	2.79760
C	1.46460	2.33770	3.06370
C	0.43760	1.50270	2.28970
H	-0.54470	1.96620	2.38650
H	0.40400	0.52150	2.76790
C	0.83350	1.36390	0.81100
H	0.09670	0.73120	0.30560
H	1.81590	0.89350	0.70770
H	0.85580	2.33250	0.30740
C	2.85440	4.30420	3.56520
C	4.28200	6.19460	3.87220
H	5.18540	5.59940	3.71380
H	4.05250	6.20740	4.94150
C	4.40770	7.58950	3.29560
H	4.62270	7.55190	2.22350
H	5.22980	8.11190	3.79490
H	3.48840	8.16170	3.45260
C	-0.74420	4.99400	0.61870
H	-0.25650	4.69370	-0.31340
H	-0.61290	6.06650	0.78940

H -1.80400 4.73860 0.59330
26
ts2_xyz-3
G = -668.2418741

O	3.59020	3.60850	4.13890
O	2.32820	1.70150	3.37750
O	3.12380	5.64430	3.28190
O	-0.28900	4.41420	1.81720
N	1.04140	4.75800	1.92180
C	1.76230	3.83310	2.62440
C	1.58560	2.42010	2.64000
C	0.51160	1.69620	1.82450
H	0.40140	2.23980	0.88300
H	-0.43820	1.80470	2.35880
C	0.83310	0.21960	1.57830
H	0.03910	-0.22510	0.97140
H	0.90050	-0.33820	2.51620
H	1.77950	0.10360	1.04110
C	2.89590	4.33470	3.42020
C	4.24670	6.20820	4.03020
H	5.16440	5.72090	3.68990
H	4.09990	5.98160	5.09010
C	4.25440	7.69790	3.75730
H	4.38700	7.89940	2.69000
H	5.08470	8.15850	4.30180
H	3.32170	8.16140	4.09220
C	-0.96930	5.31350	0.92240
H	-0.55660	5.22500	-0.08700
H	-0.87900	6.34290	1.28180
H	-2.01250	4.99580	0.93610

26
ts2_xyz-4
G = -668.2415608

O	3.36320	4.00650	4.45690
O	2.25030	1.88330	3.94700
O	2.85430	5.81470	3.19630
O	-0.32860	4.13280	1.71740
N	0.96870	4.56210	1.87170
C	1.66620	3.82200	2.78630
C	1.55990	2.42140	3.03180
C	0.64510	1.47920	2.24080
H	-0.38690	1.81760	2.33910
H	0.72990	0.49370	2.70370
C	1.05920	1.41350	0.76180
H	0.40600	0.70490	0.24250
H	2.09180	1.06750	0.65560
H	0.96550	2.38590	0.27370
C	2.69770	4.53680	3.56550
C	3.88300	6.59950	3.87850
H	3.85390	6.36420	4.94490
H	3.55750	7.62930	3.72430
C	5.25470	6.34740	3.27580
H	5.56290	5.30920	3.42410
H	5.98710	6.99940	3.76340
H	5.25280	6.57010	2.20460
C	-0.95710	4.82330	0.62050
H	-0.43710	4.59540	-0.31470
H	-0.95560	5.90160	0.80460

H -1.97850 4.44270 0.59110
26
ts2_xyz-7
G = -668.2407260
O 3.66580 3.84560 4.06400
O 2.90480 1.83340 2.92140
O 2.68730 5.81940 3.54990
O -0.33760 4.07430 1.82820
N 0.87580 4.69790 2.00890
C 1.81420 3.86690 2.55870
C 1.99240 2.47810 2.31590
C 1.12290 1.64600 1.37320
H 1.78870 0.91530 0.90510
H 0.73560 2.30520 0.59470
C -0.01870 0.92600 2.10670
H -0.56770 0.30040 1.39550
H -0.71170 1.64670 2.54620
H 0.37050 0.27940 2.89880
C 2.80400 4.48930 3.46150
C 3.64820 6.53420 4.39030
H 3.81120 5.95830 5.30400
H 3.12880 7.46240 4.63300
C 4.94360 6.79420 3.64040
H 5.44590 5.85630 3.38920
H 5.61250 7.38710 4.27290
H 4.75170 7.35410 2.72020
C -1.23060 4.92240 1.08220
H -0.83480 5.09400 0.07680
H -1.37050 5.87350 1.60430
H -2.17120 4.37300 1.03280
26
ts2_xyz-2
G = -668.2401147
O 3.44170 3.88260 4.23000
O 2.41390 1.87190 3.40630
O 2.80250 5.87460 3.37010
O -0.40450 4.31730 1.75620
N 0.87570 4.79910 1.92730
C 1.66000 3.94250 2.64850
C 1.63080 2.51930 2.64390
C 0.67650 1.69580 1.77600
H 0.53810 2.24280 0.84040
H -0.29770 1.68420 2.27630
C 1.17300 0.27050 1.51710
H 0.45770 -0.24780 0.87210
H 1.26900 -0.29640 2.44700
H 2.14550 0.27410 1.01500
C 2.70700 4.54620 3.49120
C 3.84040 6.55940 4.14210
H 3.88190 6.11940 5.14100
H 3.46840 7.58270 4.21040
C 5.18230 6.49500 3.43310
H 5.53660 5.46360 3.35720
H 5.91770 7.07290 4.00260
H 5.10950 6.92270 2.42860
C -1.13740 5.15920 0.84730
H -0.67610 5.13530 -0.14450
H -1.17080 6.18480 1.22680

H	-2.14140	4.73470	0.81010

26
ts2mod_xyz
G = -668.2362331

O	2.51740	6.37200	3.11800
O	1.20150	2.38120	3.97200
O	3.48440	4.42110	3.82140
O	-0.40610	4.04230	1.47490
N	0.38140	5.01650	1.99280
C	1.47490	4.30210	2.66350
C	1.46650	2.80810	2.85450
C	1.82010	1.93650	1.67940
H	2.83630	2.22900	1.37570
H	1.17430	2.23260	0.84380
C	1.73230	0.43850	1.96540
H	2.01540	-0.12800	1.07370
H	0.71440	0.15270	2.24700
H	2.40150	0.15260	2.78210
C	2.51310	5.14090	3.21560
C	4.61390	5.14970	4.38280
H	5.00440	4.47630	5.14760
H	4.24360	6.06120	4.85810
C	5.65390	5.45070	3.31530
H	5.99760	4.52670	2.84050
H	6.51540	5.94360	3.77810
H	5.24620	6.11460	2.54820
C	-1.55190	4.59780	0.78950
H	-1.20520	5.22320	-0.03670
H	-2.14280	5.18380	1.49750
H	-2.11220	3.73840	0.42430

References

1. Jr, C. R. I.; Moran, S. J. P. Diastereoselective Reduction of *E* and *Z* α-Alkoxyimino-β-Ketoesters by Sodium Borohydride. *Tetrahedron*, **1999**, *55*, 14221–14232.
2. a) Šterk, D.; Stephan, M.; Mohar, B. Highly Enantioselective Transfer Hydrogenation of Fluoroalkyl Ketones. *Org. Lett.* **2006**, *8*, 5935–5938. b) Sun, Y.; Wan, X.; Guo, M.; Wang, D.; Dong, X.; Pana, Y.; Zhang, Z. Chiral Biphosphine Ligands for the Ru-Catalyzed Enantioselective Hydrogenation of β-Ketoesters. *Tetrahedron: Asymmetry*, **2004**, *15*, 2185–2188. c) Junge, K.; Hagemann, B.; Enthaler, S.; Oehme, G.; Michalik, M.; Monsees, A.; Riermeier, T.; Dingerdissen, U.; Beller, M. Enantioselective Hydrogenation of β-Ketoesters with Monodentate Ligands. *Angew. Chem. Int. Ed.* **2004**, *43*, 5066–5069. d) Wan, X.; Sun, Y.; Luo, Y.; Li, D.; Zhang, Z. Synthesis of a Bulky and Electron-Rich Derivative of SEGPhos and Its Application in Ru-Catalyzed Enantioselective Hydrogenation of β-Ketoesters. *J. Org. Chem.* **2005**, *70*, 1070–1072. e) Wu, J.; Chen, H.; Zhou, Z.-Y.; Yeung, H. C.; Chan C. S. A. Synthesis and Structural Characterization of a Highly Effective Chiral Dipyridylphosphine Ligand and Its Application in the Ru-Catalyzed Asymmetric Hydrogenation of β-Ketoesters. *Synlett* **2001**, *SI*, 1050–1054. f) Niu, Z.; Chen, J.; Chen, Z.; Ma, M.; Song, C.; Ma, Y. Application of Bidentate Oxazoline−Carbene Ligands with Planar and Central Chirality in Asymmetric β-Boration of α,β-Unsaturated Esters. *J. Org. Chem.* **2015**, *80*, 602−608. g) Nishiguchi, S.; Sydnes, M. O.; Taguchi, A.; Regnier, T.; Kajimoto, T.; Node, M.; Yamazaki, Y, Yakushiji, F.; Kiso Y.; Hayashi, Y. Total synthesis of (+)-negamycin and its 5-epi-derivative. *Tetrahedron* **2010**, *66*, 314–320. h) Baker-Glenn, C.; Ancliff, R.; Gouverneur, V. A biocatalytic route to enantioenriched, sulfanyl aldol products. *Tetrahedron* **2004**, *60*, 7607–7619. i) Kitamura, M.; Ohkuma, T.; Takaya, H.; Noyori, R. A Practical Asymmetric Synthesis of Carnitine. *Tetrahedron Lett.* **1988**, *29*, 1555-1556.
3. Tharra, P. R.; Mikhaylov, A.; Švejkar, J.; Gysin, M.; Hobbie, S. N.; Švenda, J. Short Synthesis of (+)-Actinobolin: Simple Entry to Complex Small-Molecule Inhibitors of Protein Synthesis. *Angew. Chem.Int. Ed.* **2022**, *61*, e2021165.
4. a) Shibata, Y.; Kagechika, K.; Yamaguchi, M.; Yoshikawa, K.; Chiba, K.; Takano, H.; Akiyama, C.; Ono, M.; Nishi, M.; Kubo, H.; Kobayashi, Y.; Usui, H. Synthesis and Structure-Activity Relationships of Novel Zwitterionic Compounds as Peroxisome Proliferator Activated Receptor α/γ Dual Agonists with Improved Physicochemical Properties. *Chem. Pharm. Bull.* **2013**, *61*, 1248–1263. b) Jiao, Y.-X.; Wu, L.-L.; Zhu, H.-M.; Qin, J.-K.; Pan, C.-X.; Mo, D.-L.; Su, G.-F. Tandem C–N Bond Formation through Condensation and Metal-Free N-Arylation: Protocol for Synthesizing Diverse Functionalized Quinoxalines. *J. Org. Chem.* **2017**, *82*, 4407–4414.
5. Holmquist, R. C.; Roskamp, J. E. A Selective Method for the Direct Conversion of Aldehydes into β-Keto Esters with Ethyl Diazoacetate Catalyzed by Tin(II) Chloride. *J. Org. Chem.* **1989**, *54*, 3258–3260.
6. Jiang, Y.-T.; Yu, Z.-Z.; Zhang, Y.-K.; Wang B. N-Bromosuccinimide-Induced C−H Bond Functionalization: An Intramolecular Cycloaromatization of Electron Withdrawing Group Substituted 1-Biphenyl-2-Ylethanone for the Synthesis of 10-Phenanthrenol. *Org. Lett.* **2018**, *20*, 3728−3731.
7. Becker, M. R.; Wearing, E. R.; Schindler, C. S. Synthesis of Azetidines via Visible-Light-Mediated Intermolecular [2+2] Photocycloadditions. *Nat. Chem.* **2020**, *12*, 898–905.
8. Blackmun D. E.; Chamness S. A.; Schindler C. S. Intramolecular, Visible-Light-Mediated Aza Paternò-Büchi Reactions of Unactivated Alkenes. *Org. Lett.* **2022**, *24*, 3053-3057.

9. a) Rigaku Oxford Diffraction, CrysAlisPro Software system, version 1.171.41.116a, Rigaku Corporation, Wroclaw, Poland, **2021**. b) Sheldrick, G. M. *Acta Cryst.* **2015**, *A71*, 3. c) Sheldrick, G. M. *Acta Cryst.* **2015**, *C71*, 3. d) Silva, M. R.; Beja, A. M.; Paixao, J. A.; Lopes, S. H.; Cabral, A. M. T. D. P. V.; Gonsalves, A. M. d'A. R.; Sobral, A. J. F. N. Crystal structure of 2-hydroxyimino-3-oxo-3-phenyI-propionic acid ethyl ester, $C_{11}H_{11}NO_4$. *Z. Kristallogr. New Cryst. Struct.* **2004**, *219*, 145-146.
10. a) Jaguar: A high-performance quantum chemistry software program with strengths in life and materials sciences - Bochevarov - 2013 - International Journal of Quantum Chemistry - Wiley Online Library; Schrödinger Release 2023-2: Jaguar, Schrödinger, LLC, New York, NY, 2023. b) Macromodel—an integrated software system for modeling organic and bioorganic molecules using molecular mechanics - Mohamadi - 1990 - Journal of Computational Chemistry - Wiley Online Library; Schrödinger Release 2023-2: MacroModel, Schrödinger, LLC, New York, NY, 2023. c) Schrödinger Release 2023-2: Materials Science Suite, Schrödinger, LLC, New York, NY, 2023.
11. a) Becke, A. D. Density-Functional Thermochemistry. III. The Role of Exact Exchange. *J. Chem. Phys*. **1993**, *98*, 5648–5652. b) Lee, C.; Yang, W.; Parr, R. G. Development of the Colle-Salvetti Correlation-Energy Formula into a Functional of the Electron Density. *Phys. Rev. B* **1988**, *37*, 785–789. c) Grimme, S.; Antony, J.; Ehrlich, S.; Krieg, H. A Consistent and Accurate Ab Initio Parametrization of Density Functional Dispersion Correction (DFT-D) for the 94 Elements H-Pu. *J. Chem. Phys.* **2010**, *132*, 154104. d) Lu, C.; Wu, C.; Ghoreishi, D.; Chen, W.; Wang, L.; Damm, W.; Ross, G. A.; Dahlgren, M. K.; Russell, E.; Von Bargen, C. D.; Abel, R.; Friesner, R. A.; Harder, E. D. OPLS4: Improving Force Field Accuracy on Challenging Regimes of Chemical Space. *J. Chem. Theory Comput.* **2021**, *17*, 4291–4300.
12. Chen. J.; Fu X.-G.; Zhou, L.; Zhang, J.-T.; Qi, X.-L.; Cao X.-P. A Convergent Route for the Total Synthesis of Malyngamides O, P, Q, and R. *J. Org. Chem.* **2009**, *74*, 4149–4157.
13. Bhatta, S.; Senapati, B. K.; Patra, S. K.; Nanda, S. A sequential Friedlander and anionic benzannulation strategy for the regiodefined assembly of unsymmetrical acridines *Org. Biomol. Chem*. **2023**, *21*, 8727–8738.

Copies of ^{1}H and ^{13}C{^{1}H} NMR spectra

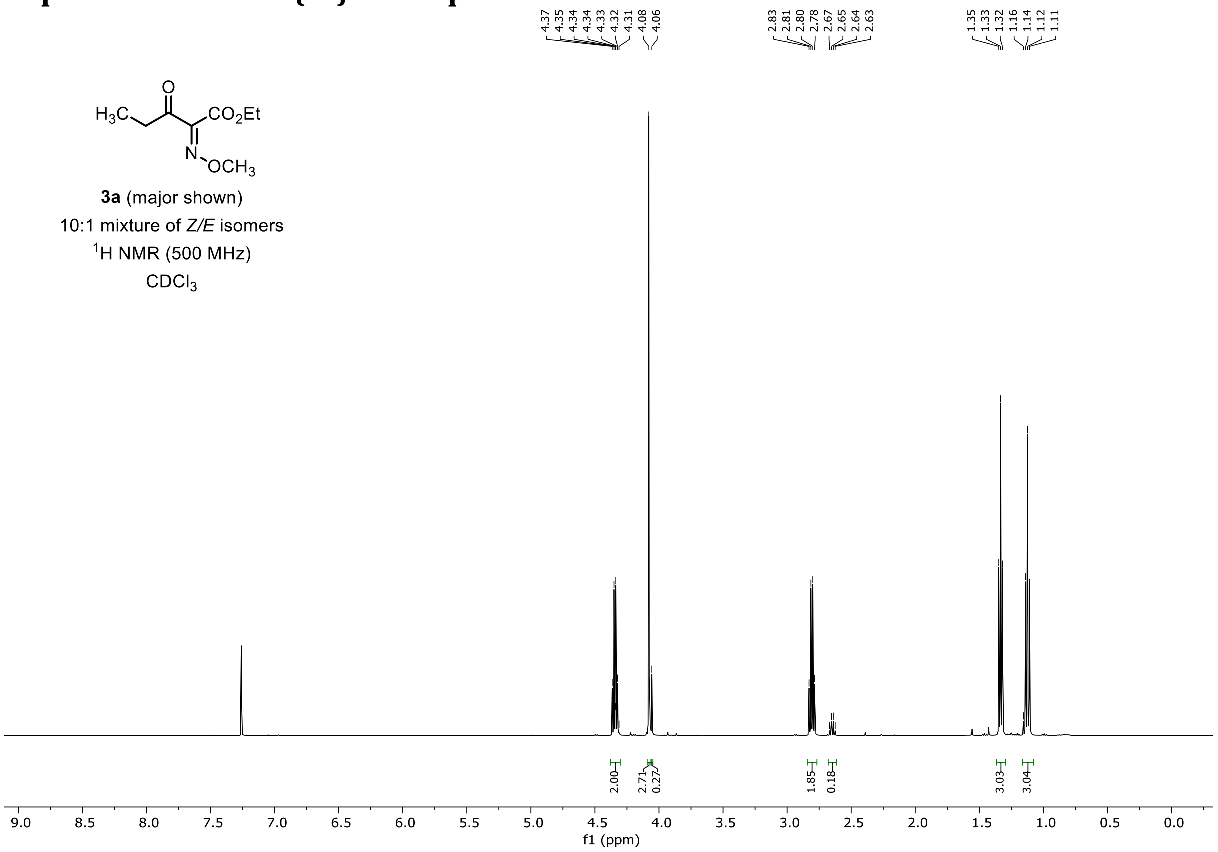

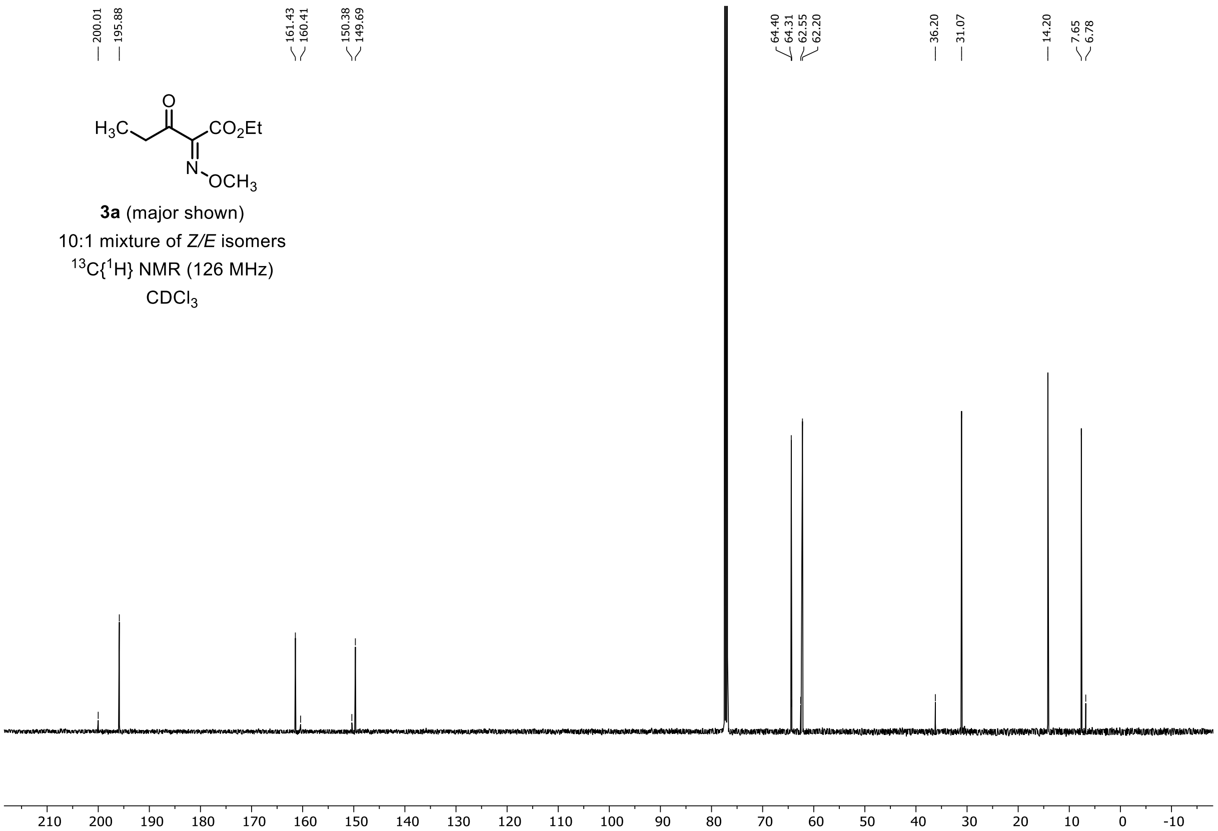

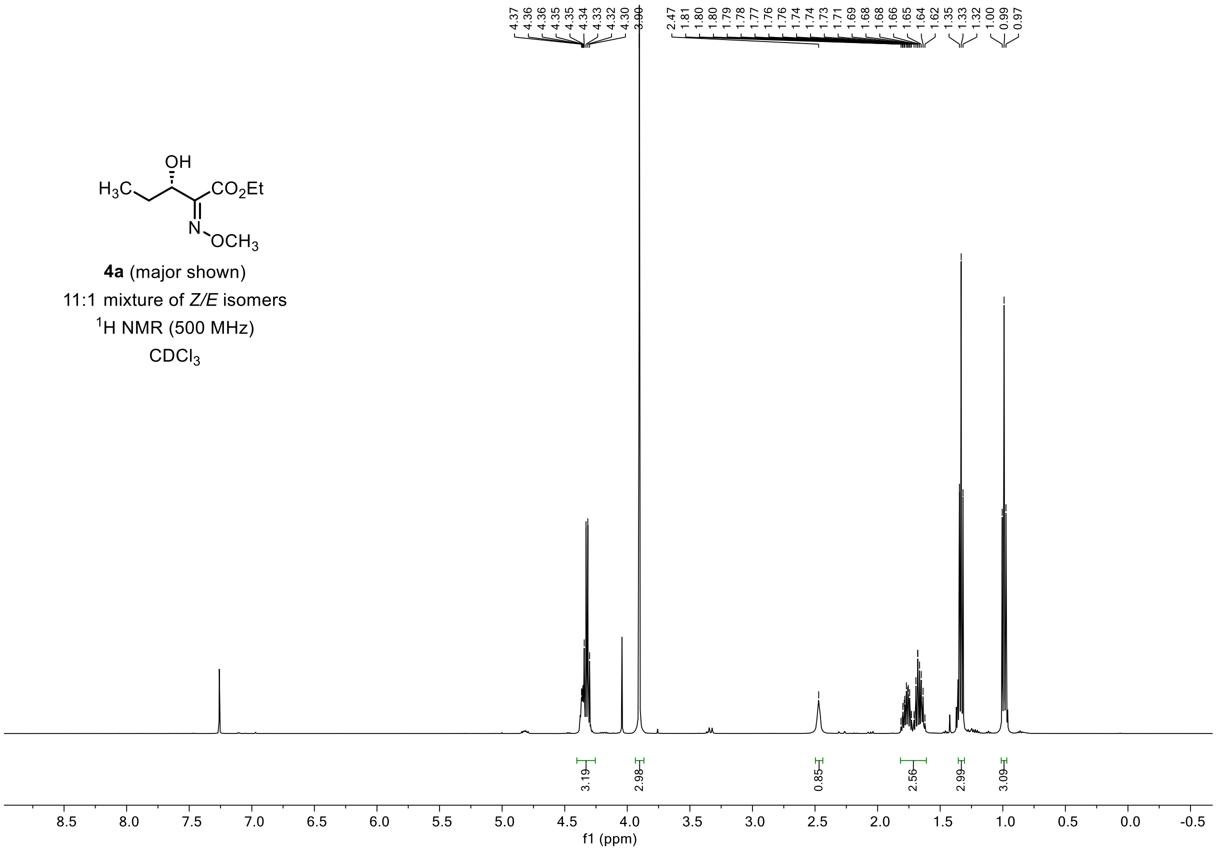

4a (major shown)
11:1 mixture of *Z/E* isomers
^{1}H NMR (500 MHz)
$CDCl_3$

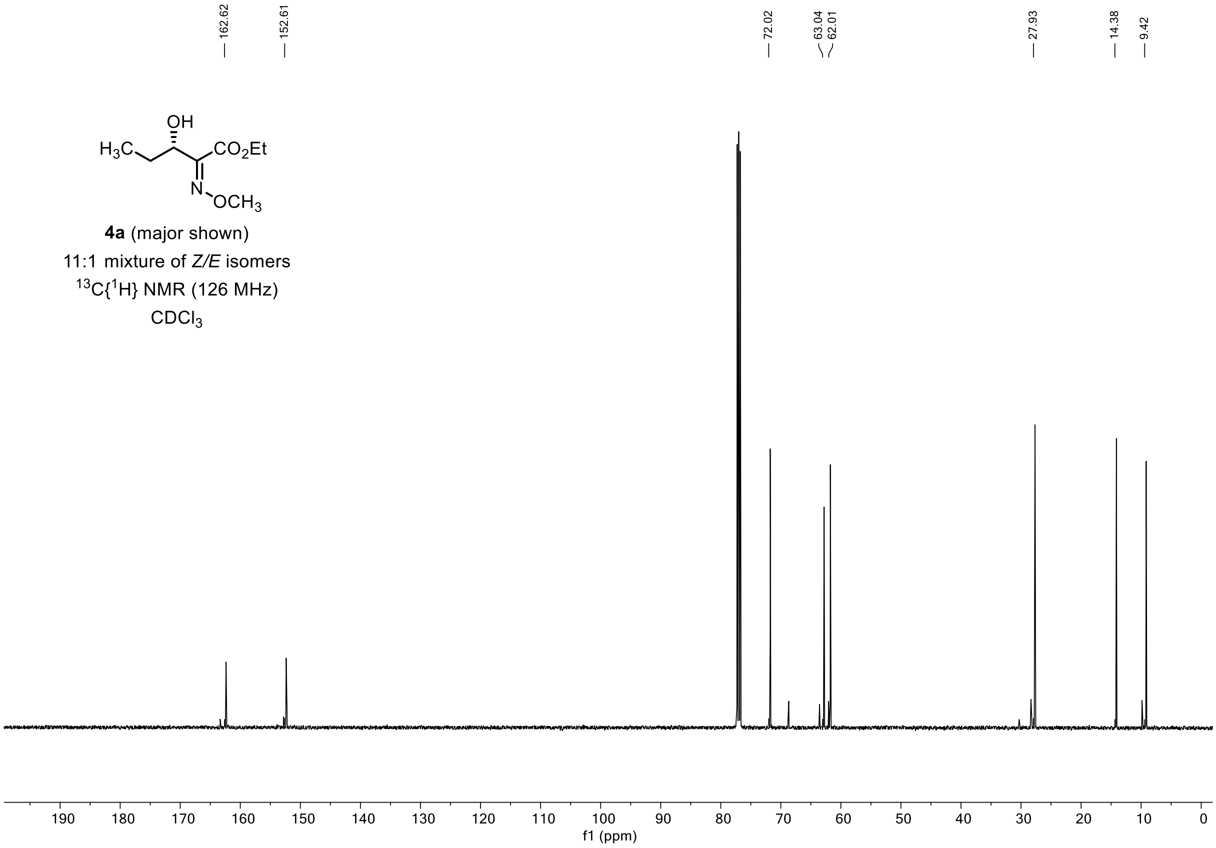

4a (major shown)
11:1 mixture of *Z/E* isomers
$^{13}C\{^{1}H\}$ NMR (126 MHz)
$CDCl_3$

14 (ca. 85% purity)
^{1}H NMR (500 MHz)
$CDCl_3$

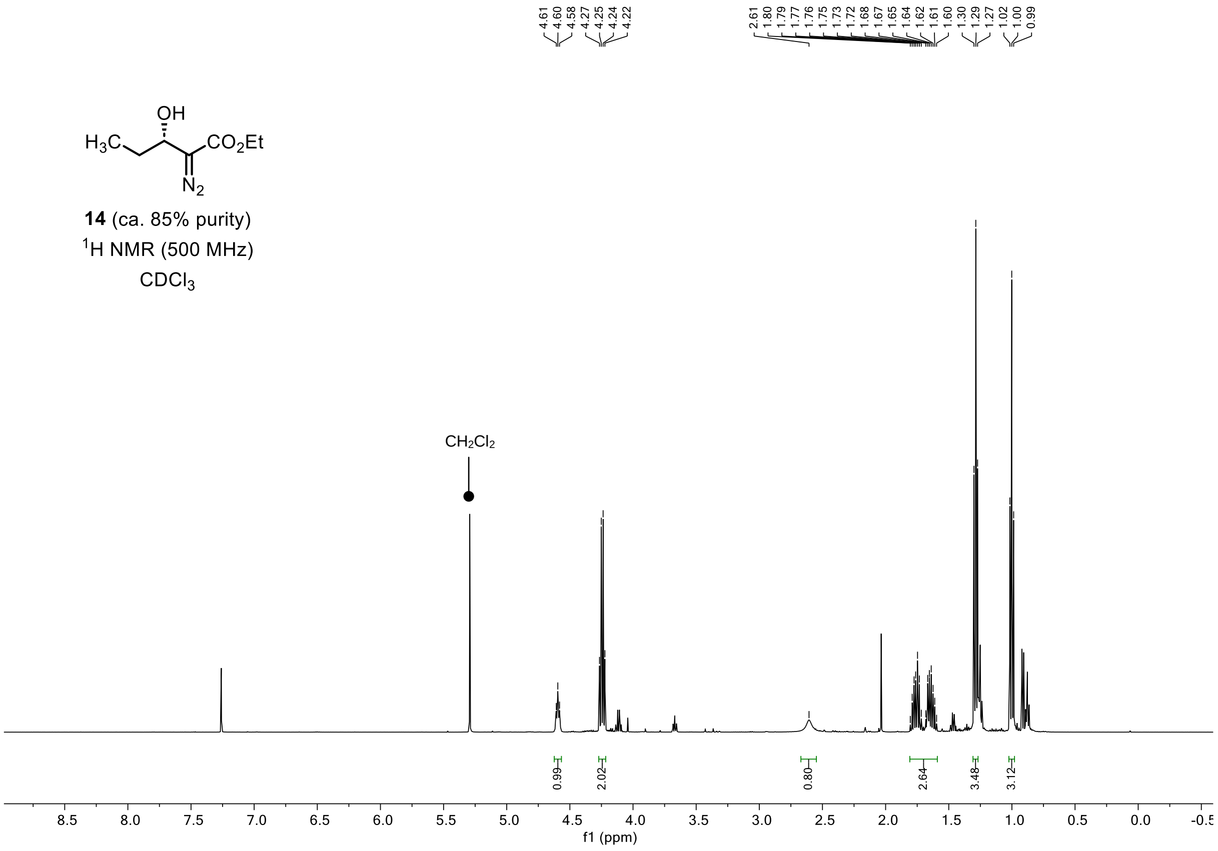

14 (ca. 85% purity)
$^{13}C\{^1H\}$ NMR (126 MHz)
$CDCl_3$

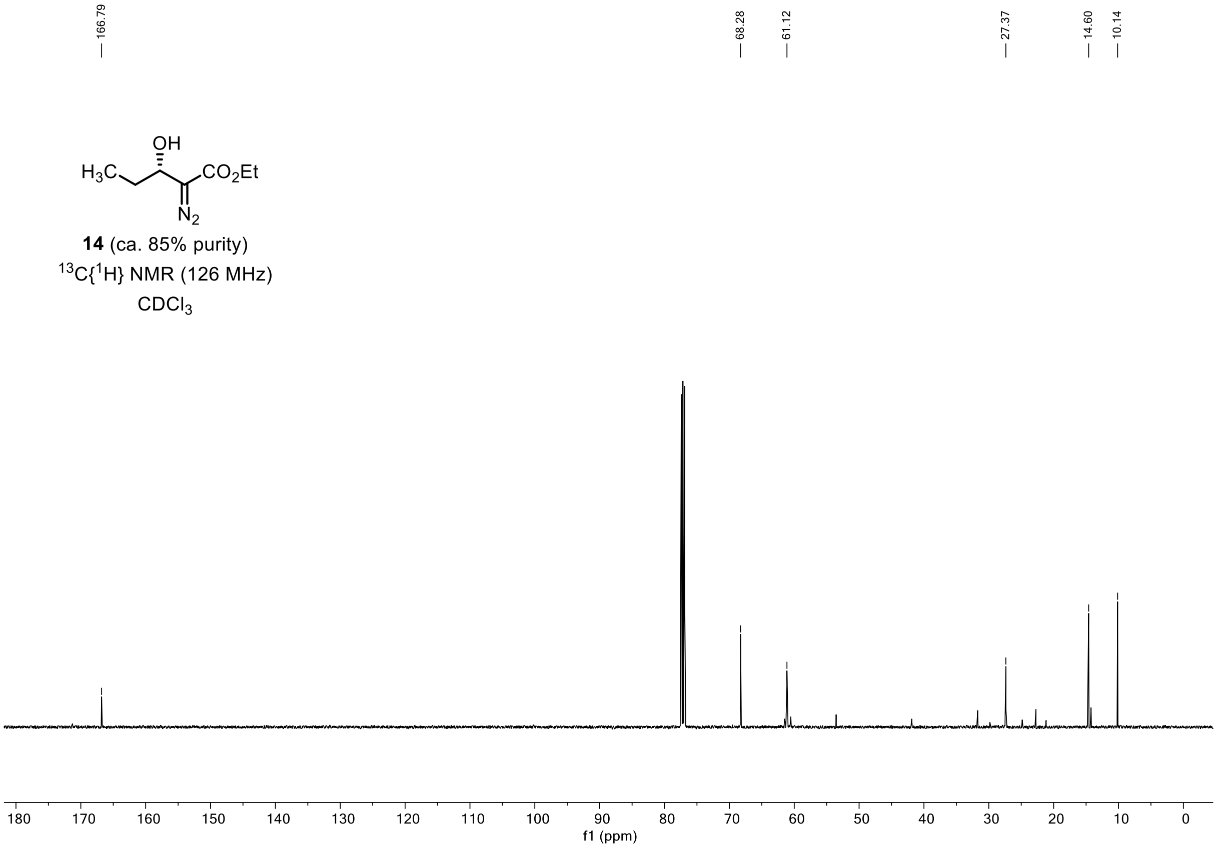

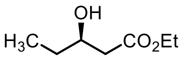

6a

^1H NMR (500 MHz)

$CDCl_3$

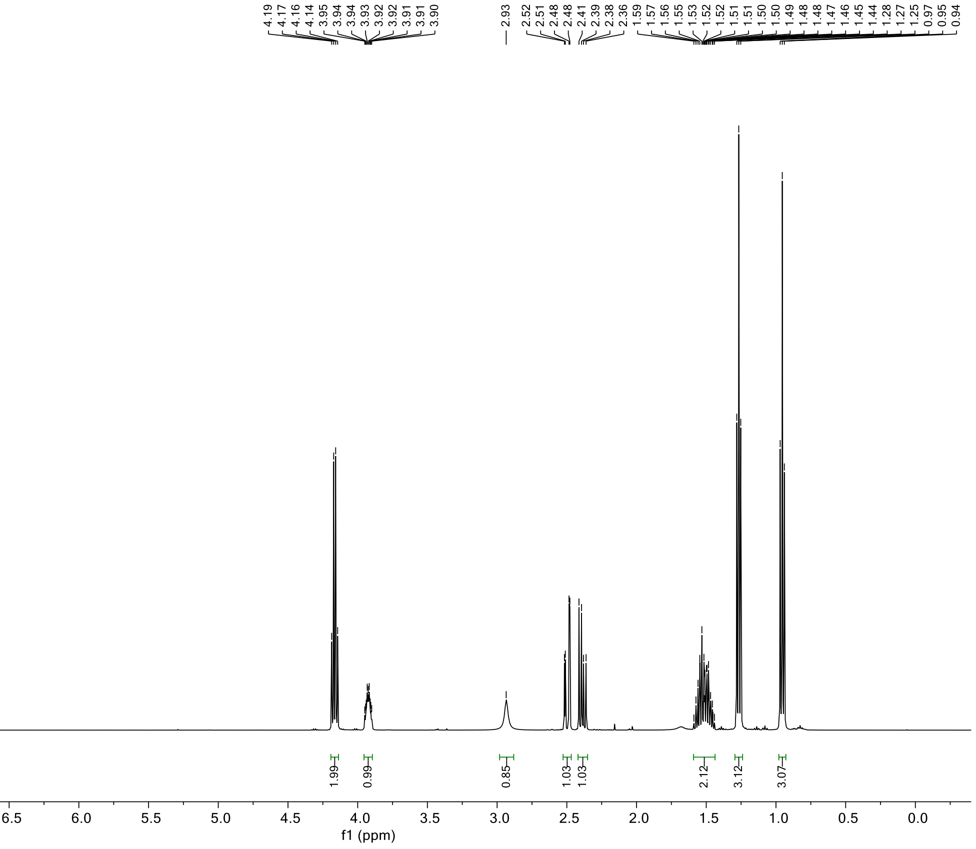

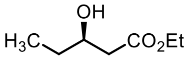

6a

$^{13}C\{^1H\}$ NMR (126 MHz)

$CDCl_3$

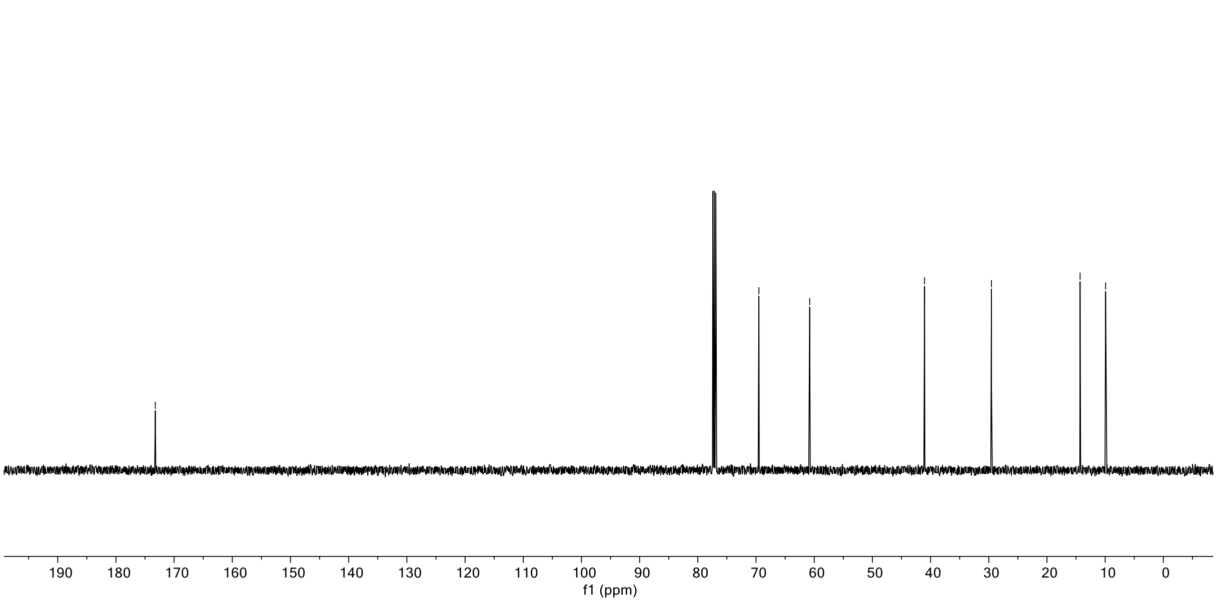

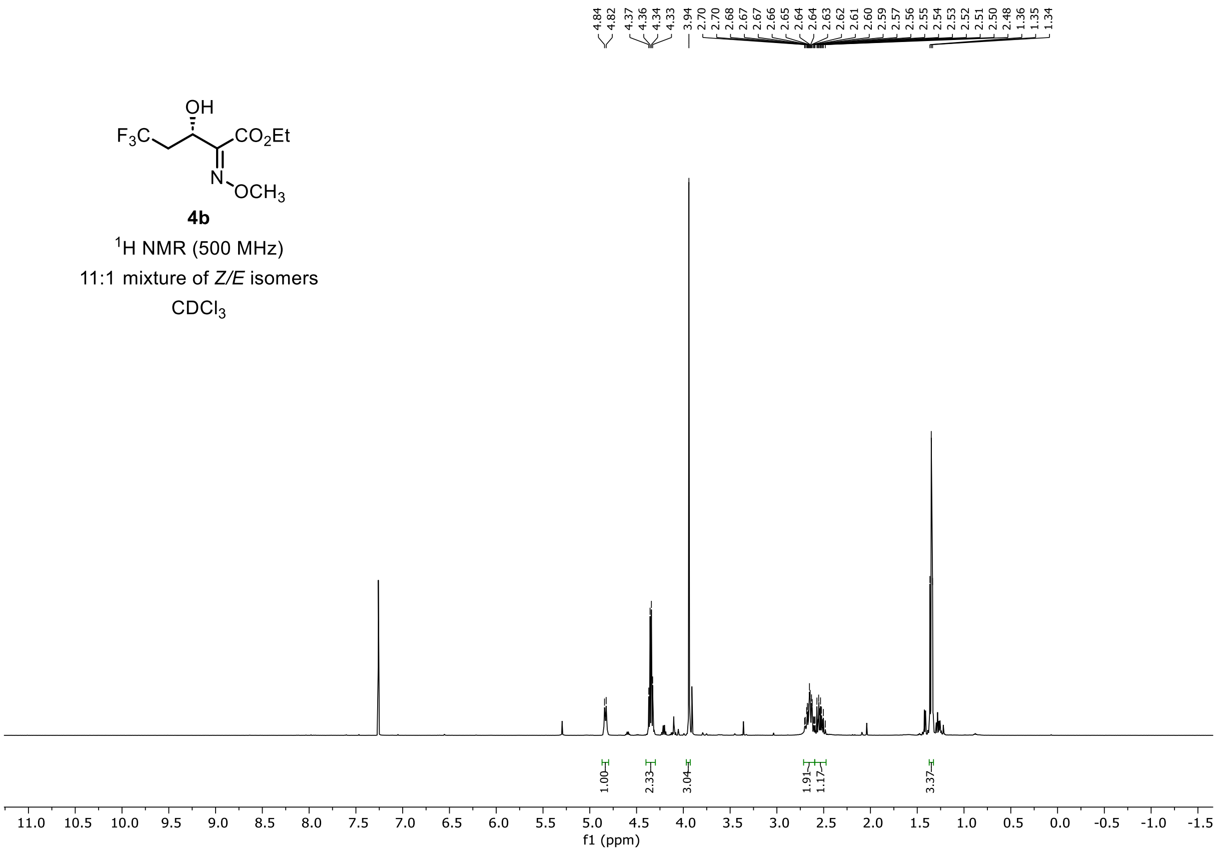
OH
F3C
CO2Et
N
OCH3
4b
1H NMR (500 MHz)
11:1 mixture of Z/E isomers
CDCl3
4.84 4.82 4.37 4.36 4.34 4.33 3.94 2.70 2.70 2.68 2.67 2.67 2.66 2.65 2.64 2.64 2.63 2.62 2.61 2.60 2.59 2.57 2.56 2.55 2.54 2.53 2.52 2.51 2.50 2.48 1.36 1.35 1.34
1.00 2.33 3.04 1.91 1.17 3.37
11.0 10.5 10.0 9.5 9.0 8.5 8.0 7.5 7.0 6.5 6.0 5.5 5.0 4.5 4.0 3.5 3.0 2.5 2.0 1.5 1.0 0.5 0.0 -0.5 -1.0 -1.5
f1 (ppm)

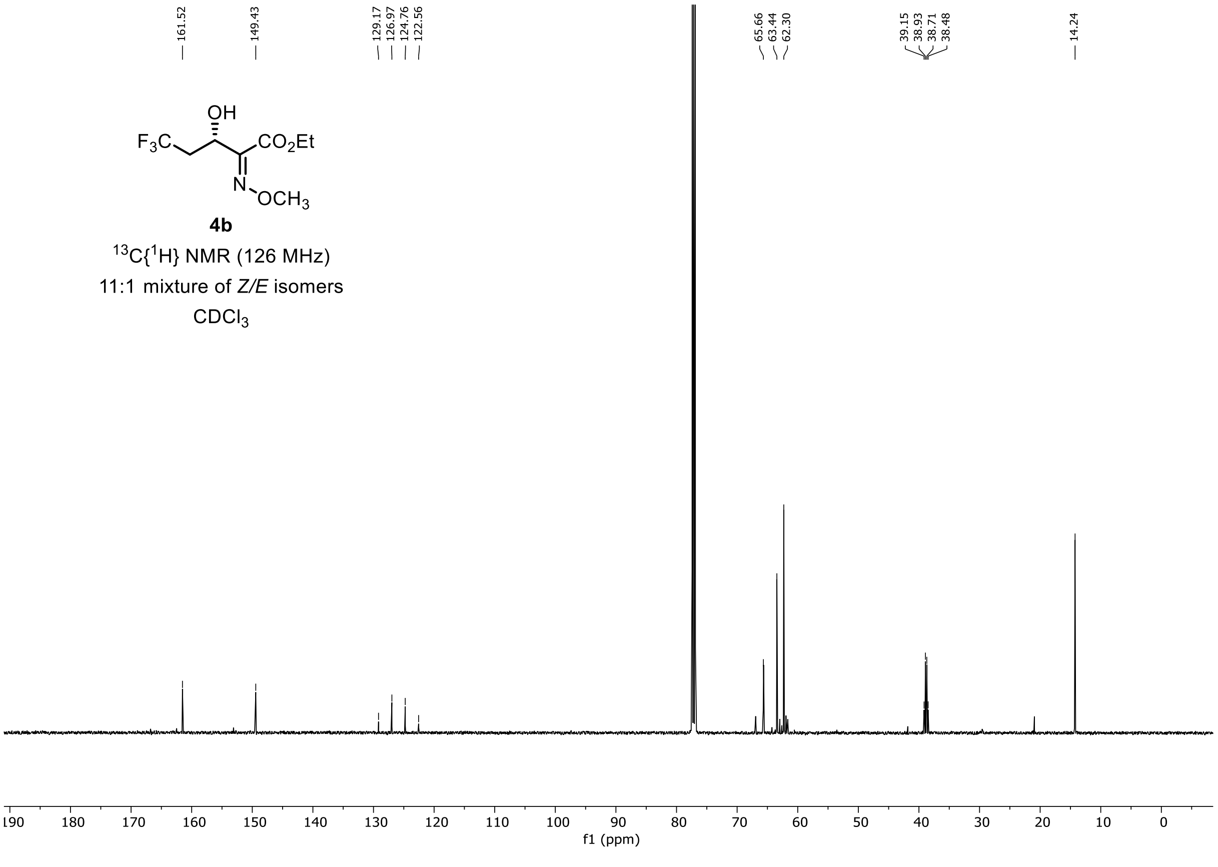
161.52 149.43 129.17 126.97 124.76 122.56 65.66 63.44 62.30 39.15 38.93 38.71 38.48 14.24
OH
F3C
CO2Et
N
OCH3
4b
13C{1H} NMR (126 MHz)
11:1 mixture of Z/E isomers
CDCl3
190 180 170 160 150 140 130 120 110 100 90 80 70 60 50 40 30 20 10 0
f1 (ppm)

15

^{1}H NMR (500 MHz)

$CDCl_3$

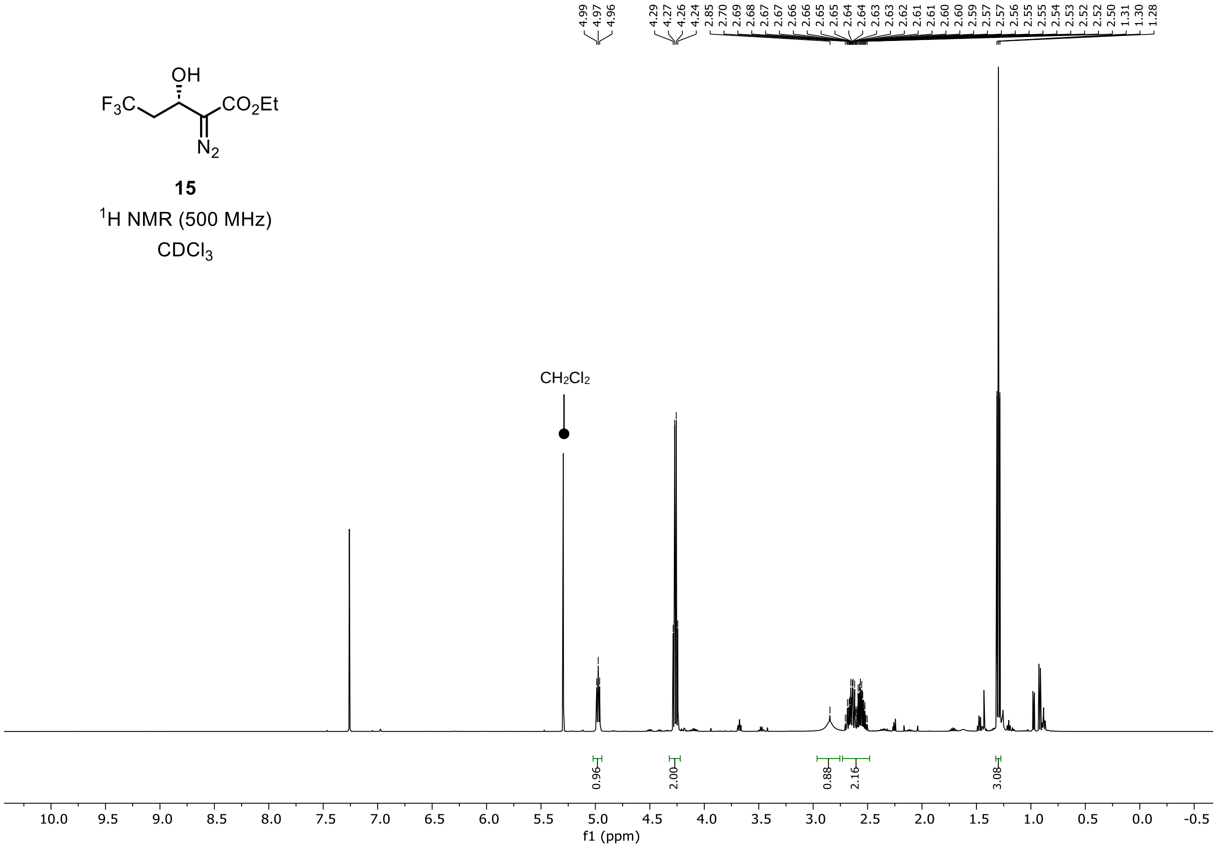

15

^{13}C{^{1}H} NMR (126 MHz)

$CDCl_3$

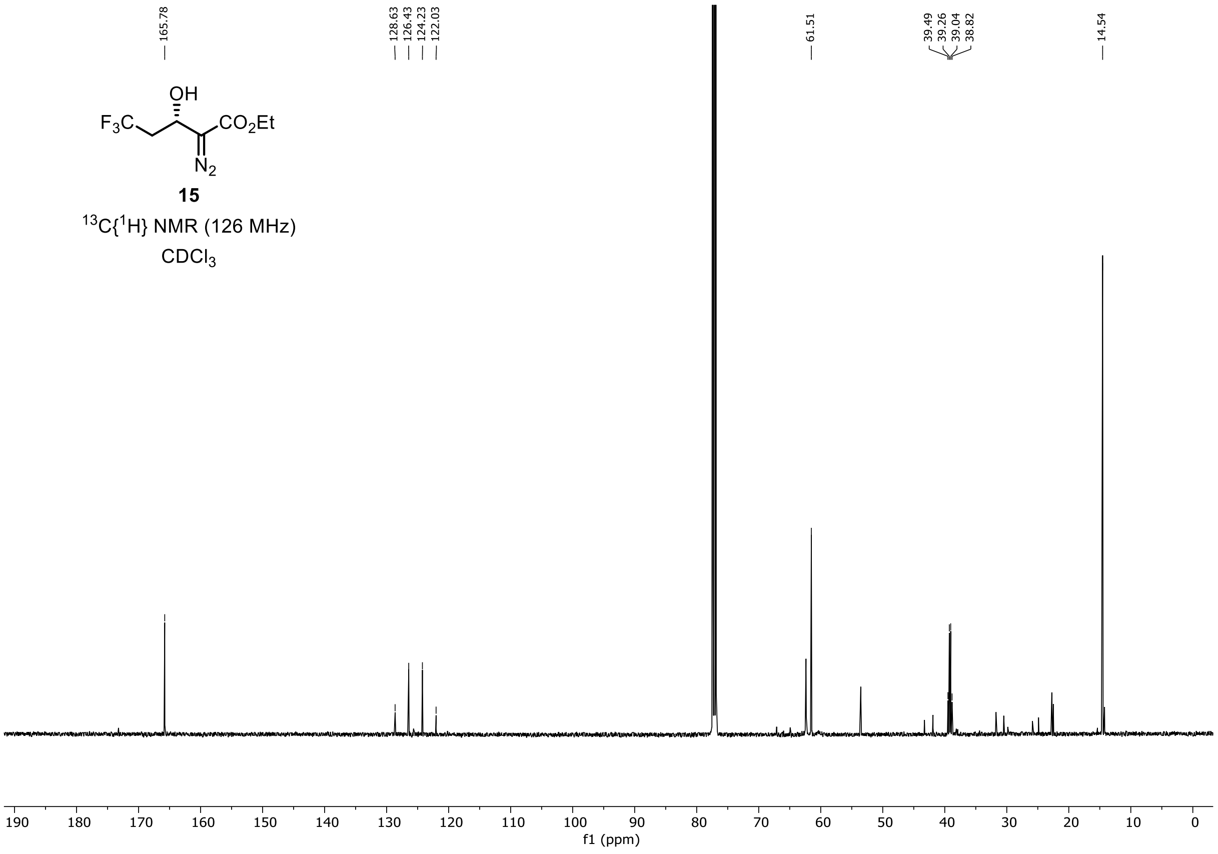

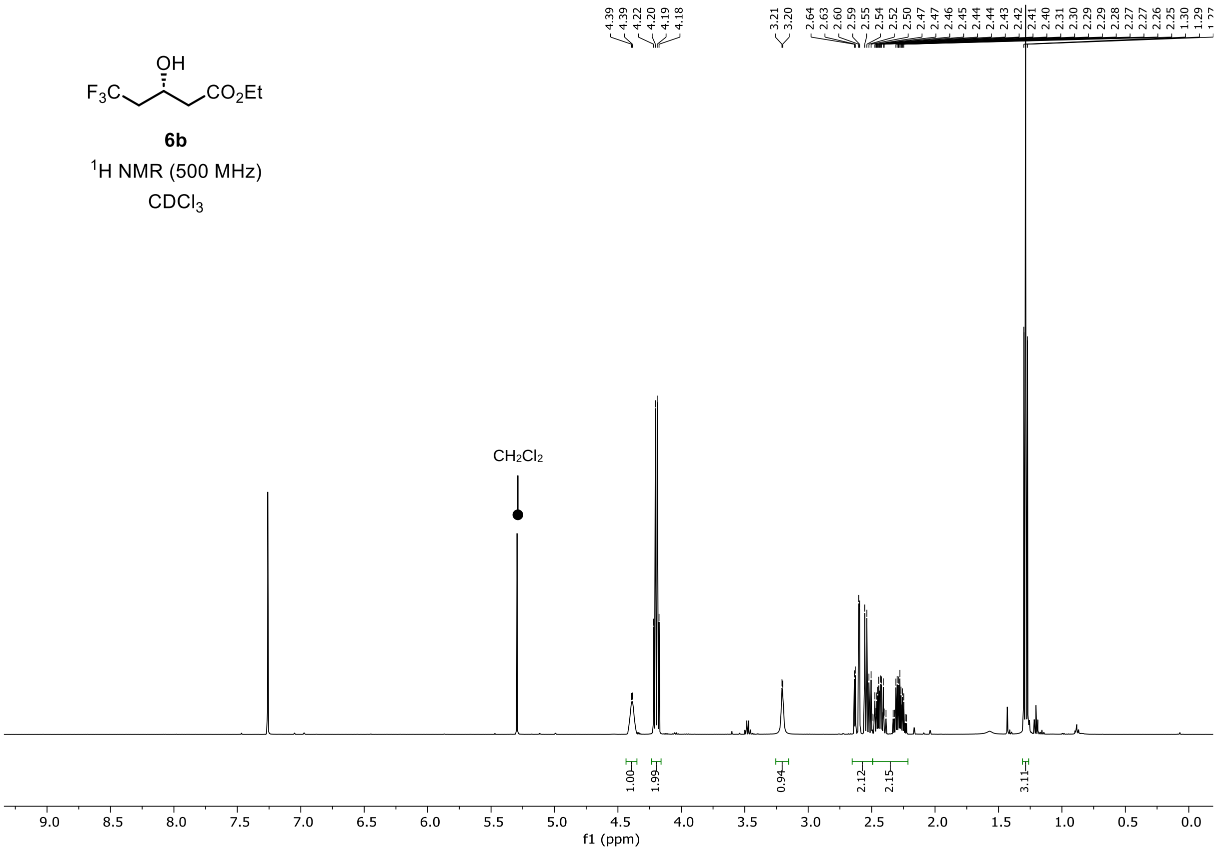
OH
F3C CO2Et
6b
1H NMR (500 MHz)
CDCl3
CH2Cl2
4.39 4.39 4.22 4.20 4.19 4.18
3.21 3.20
2.64 2.63 2.60 2.59 2.55 2.54 2.52 2.50 2.47 2.47 2.46 2.45 2.44 2.44 2.43 2.42 2.41 2.40 2.31 2.30 2.29 2.29 2.28 2.27 2.27 2.26 2.25 1.30 1.29
1.00 1.99 0.94 2.12 2.15 3.11
9.0 8.5 8.0 7.5 7.0 6.5 6.0 5.5 5.0 4.5 4.0 3.5 3.0 2.5 2.0 1.5 1.0 0.5 0.0
f1 (ppm)

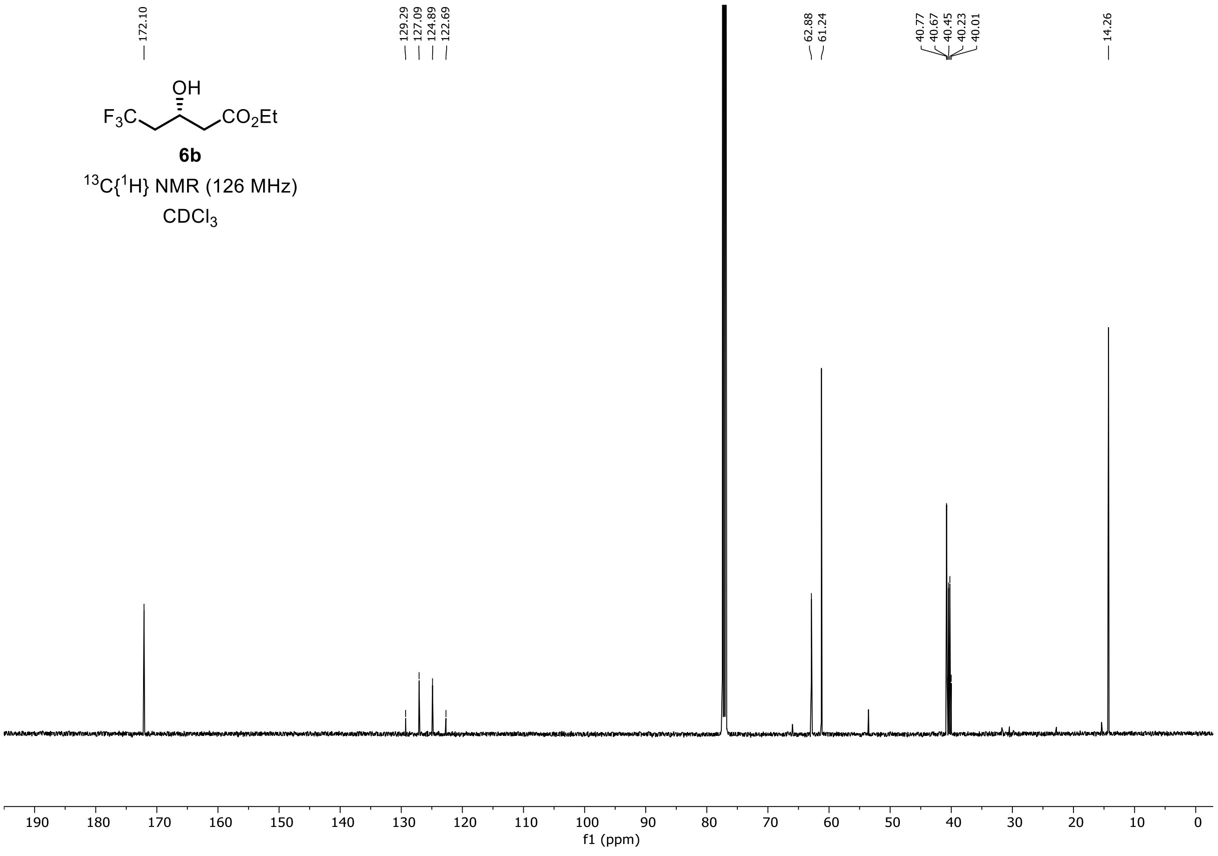
OH
F3C CO2Et
6b
13C{1H} NMR (126 MHz)
CDCl3
172.10
129.29 127.09 124.89 122.69
62.88 61.24
40.77 40.67 40.45 40.23 40.01
14.26
190 180 170 160 150 140 130 120 110 100 90 80 70 60 50 40 30 20 10 0
f1 (ppm)

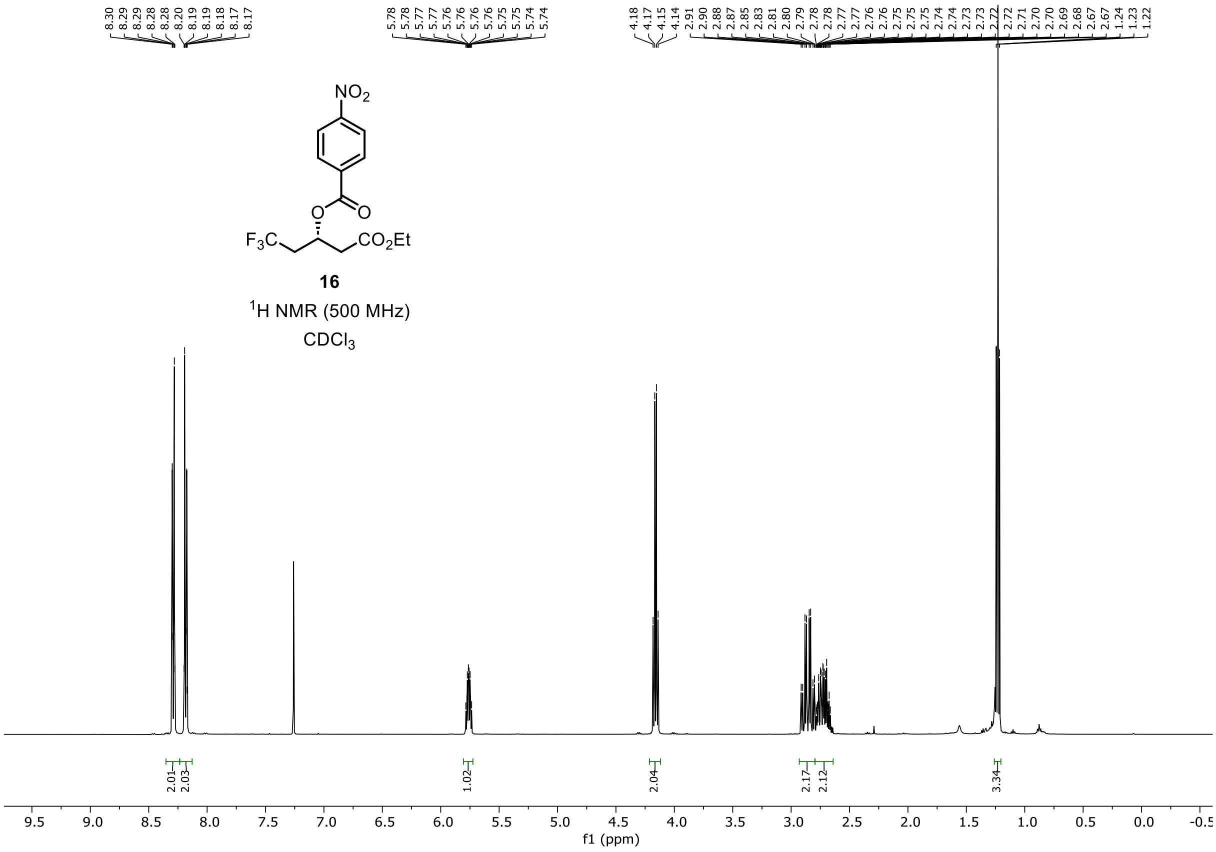
8.30 8.29 8.28 8.28 8.20 8.19 8.19 8.18 8.17 8.17
5.78 5.78 5.77 5.77 5.76 5.76 5.76 5.76 5.75 5.75 5.74 5.74
4.18 4.17 4.15 4.14 2.91 2.90 2.88 2.87 2.85 2.83 2.81 2.80 2.79 2.78 2.78 2.77 2.77 2.76 2.76 2.75 2.75 2.75 2.74 2.74 2.73 2.73 2.72 2.72 2.71 2.70 2.70 2.69 2.68 2.67 1.24 1.23 1.22
NO_2
O
O
F_3C
CO_2Et
16
^{1}H NMR (500 MHz)
$CDCl_3$
2.01
2.03
1.02
2.04
2.17
2.12
3.34
f1 (ppm)

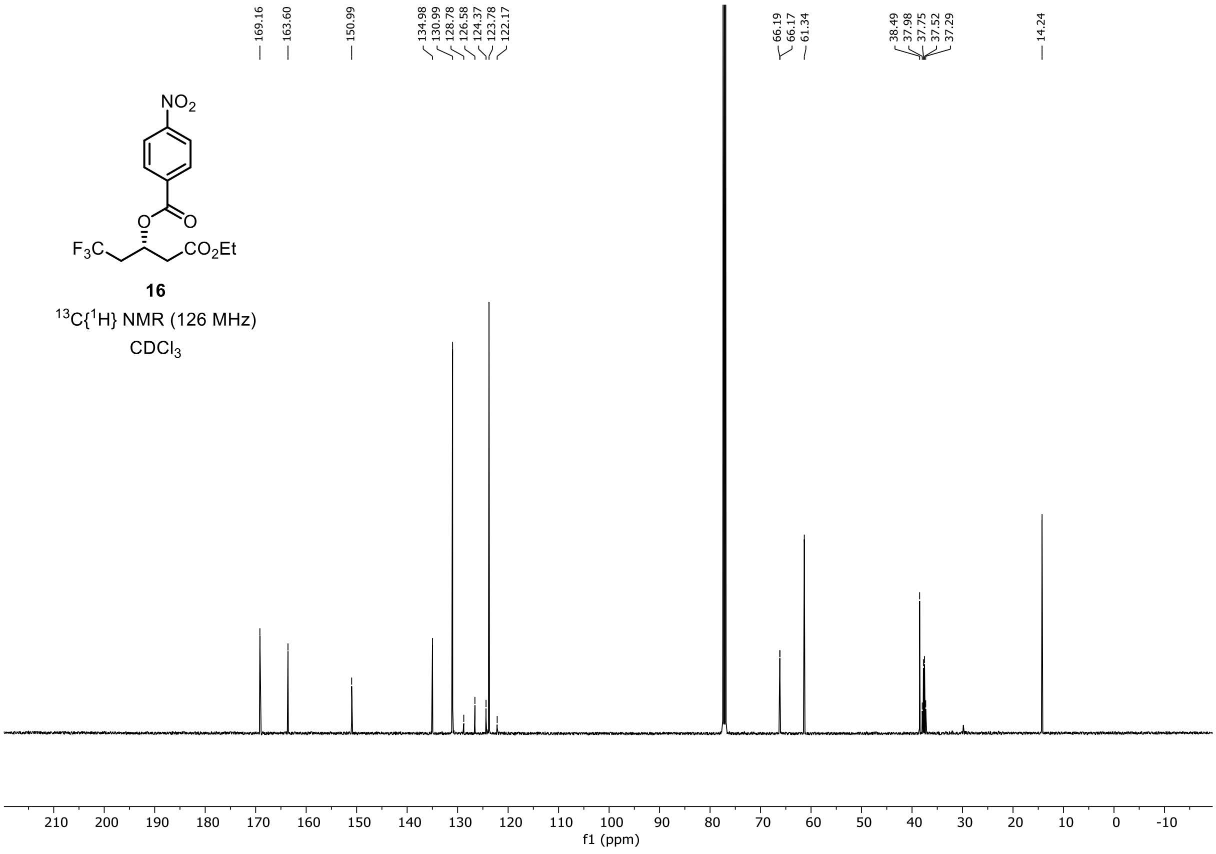
169.16
163.60
150.99
134.98 130.99 128.78 126.58 124.37 123.78 122.17
66.19 66.17 61.34
38.49 37.98 37.75 37.52 37.29
14.24
NO_2
O
O
F_3C
CO_2Et
16
$^{13}C\{^1H\}$ NMR (126 MHz)
$CDCl_3$
f1 (ppm)

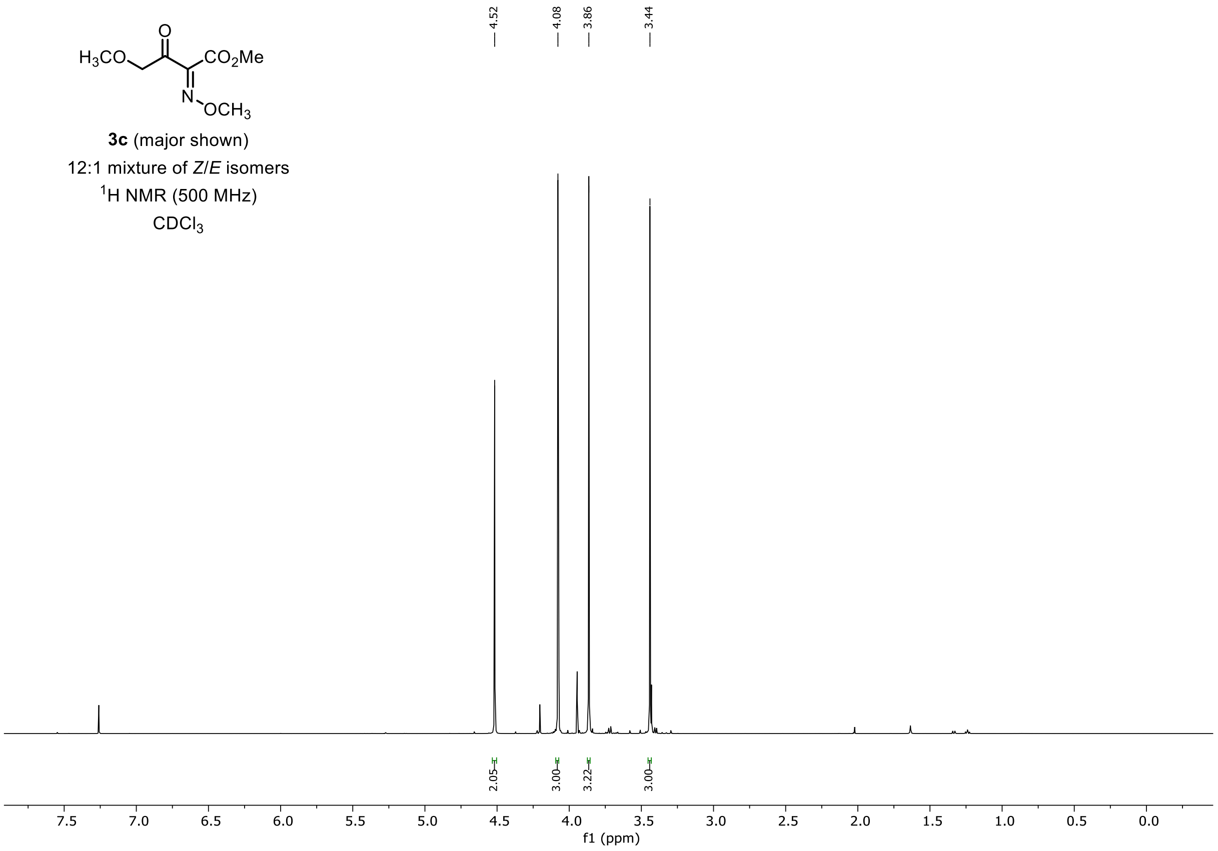
H3CO
O
CO2Me
N
OCH3
3c (major shown)
12:1 mixture of Z/E isomers
1H NMR (500 MHz)
CDCl3
4.52
4.08
3.86
3.44
2.05
3.00
3.22
3.00
7.5 7.0 6.5 6.0 5.5 5.0 4.5 4.0 3.5 3.0 2.5 2.0 1.5 1.0 0.5 0.0
f1 (ppm)

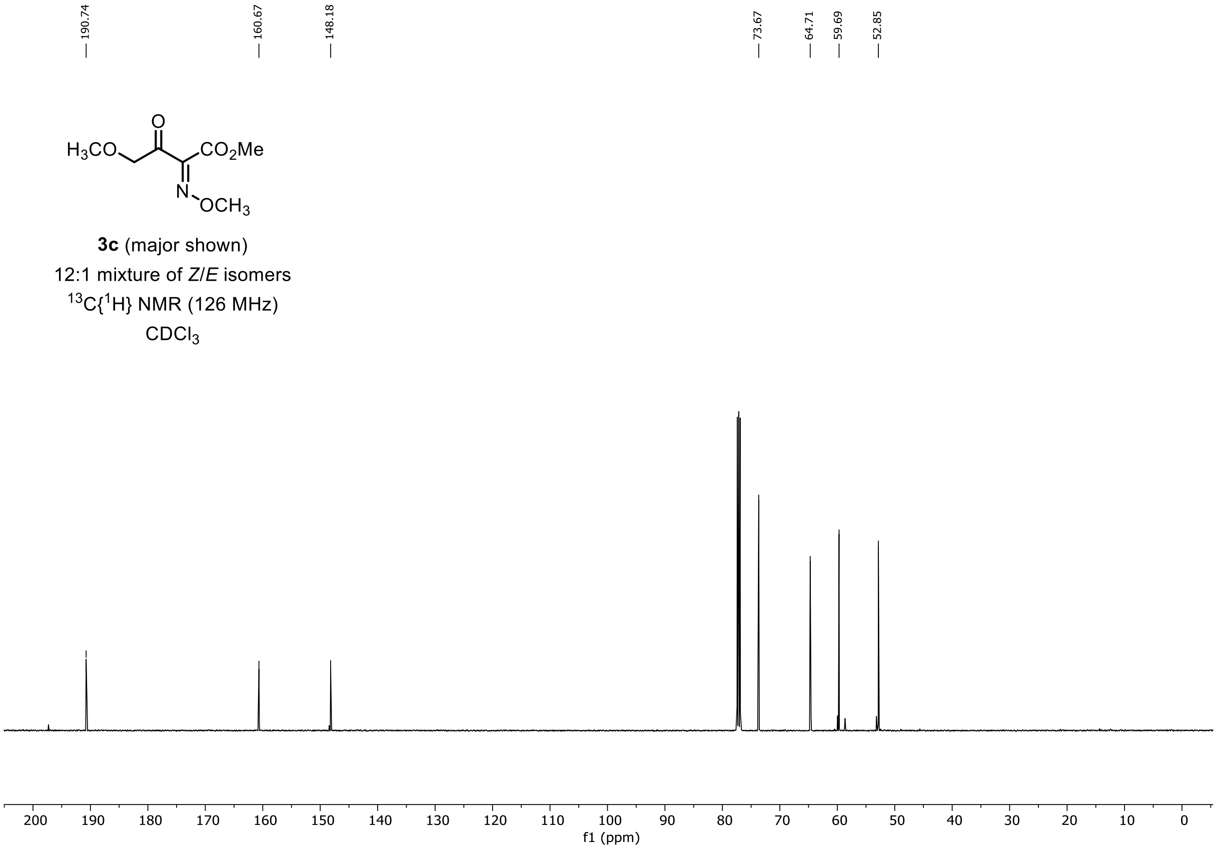
190.74
160.67
148.18
73.67
64.71
59.69
52.85
H3CO
O
CO2Me
N
OCH3
3c (major shown)
12:1 mixture of Z/E isomers
13C{1H} NMR (126 MHz)
CDCl3
200 190 180 170 160 150 140 130 120 110 100 90 80 70 60 50 40 30 20 10 0
f1 (ppm)

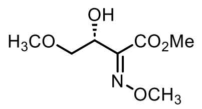

4c (major shown)
12:1 mixture of *Z*/*E* isomers
^{1}H NMR (500 MHz)
$CDCl_3$

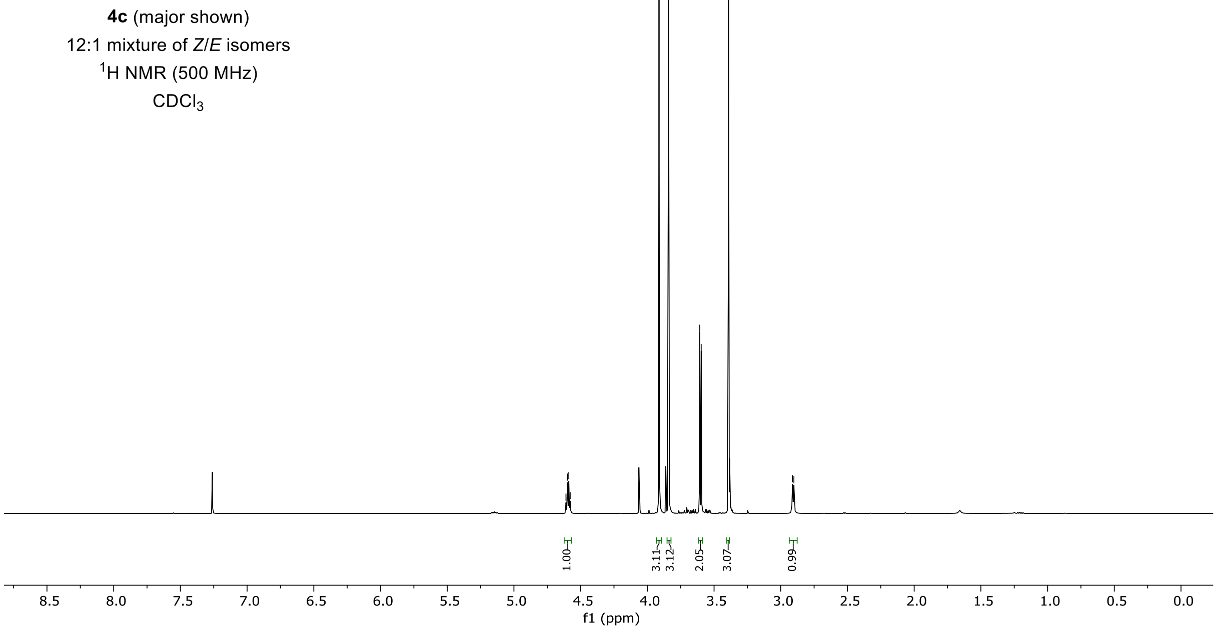

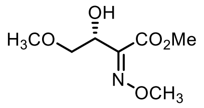

4c (major shown)
12:1 mixture of *Z*/*E* isomers
$^{13}C\{^1H\}$ NMR (126 MHz)
$CDCl_3$

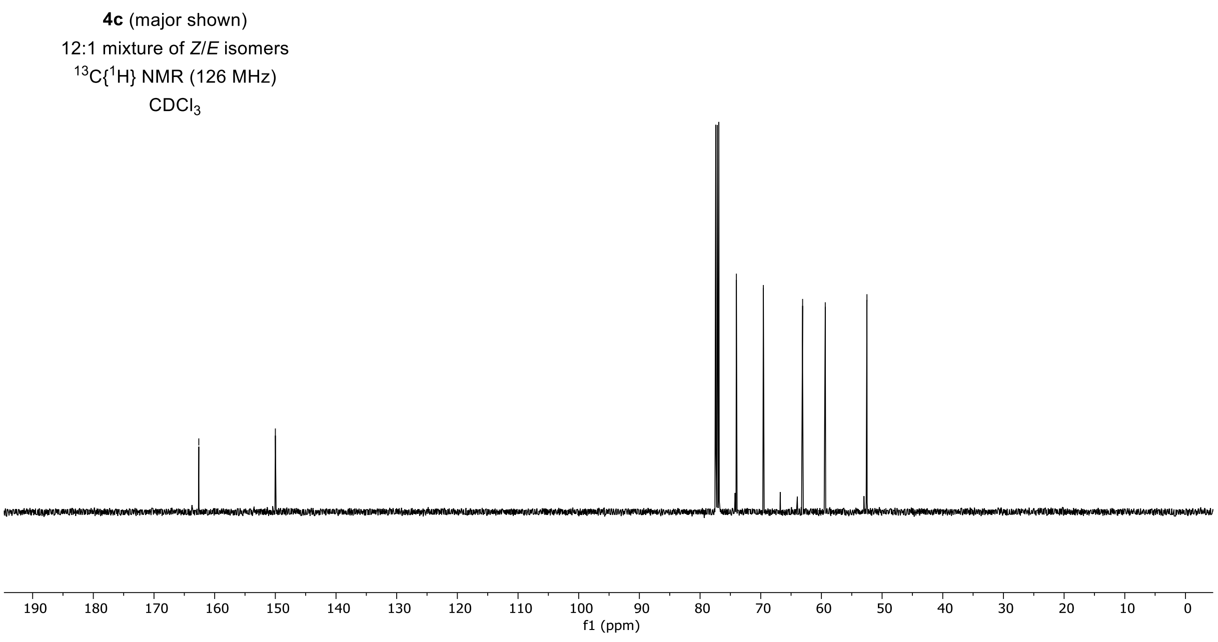

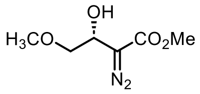

17

^1H NMR (500 MHz)

$CDCl_3$

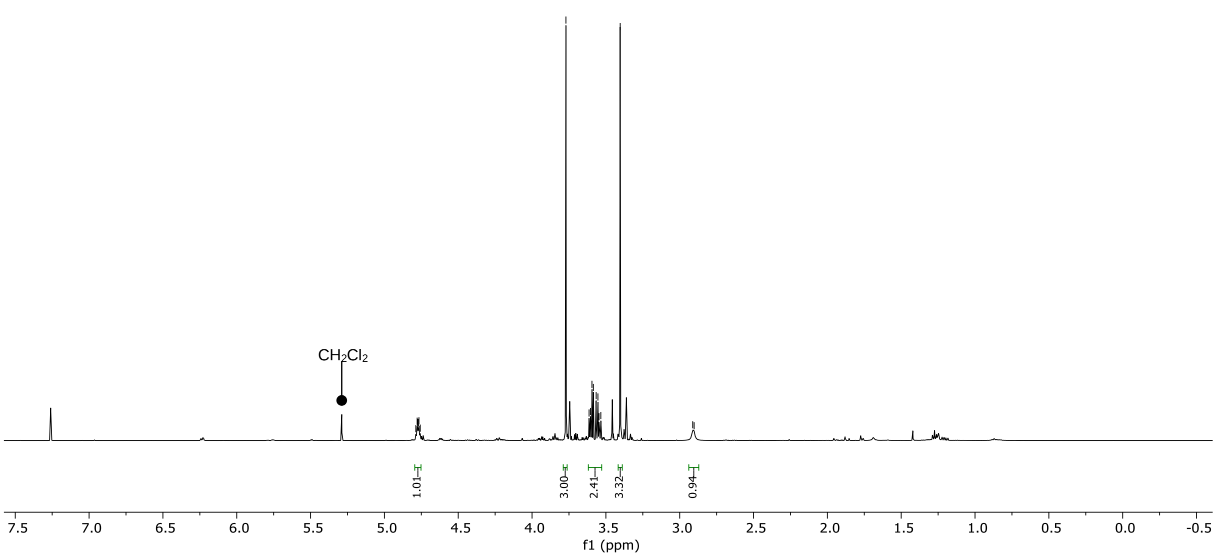

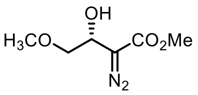

17

$^{13}C\{^1H\}$ NMR (126 MHz)

$CDCl_3$

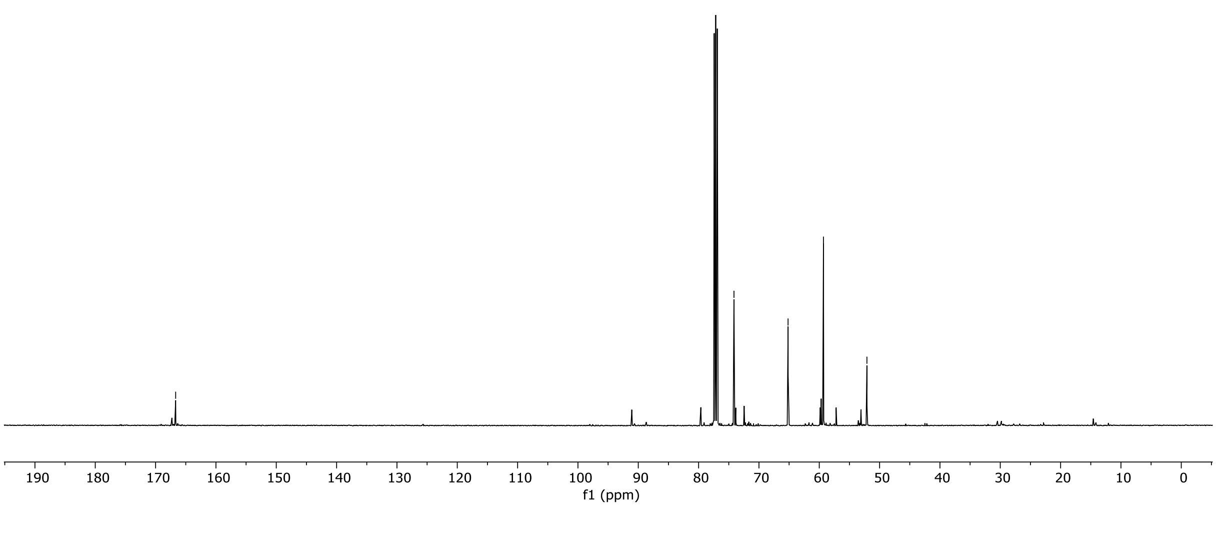

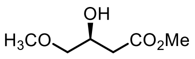

6c

^{1}H NMR (500 MHz)

$CDCl_3$

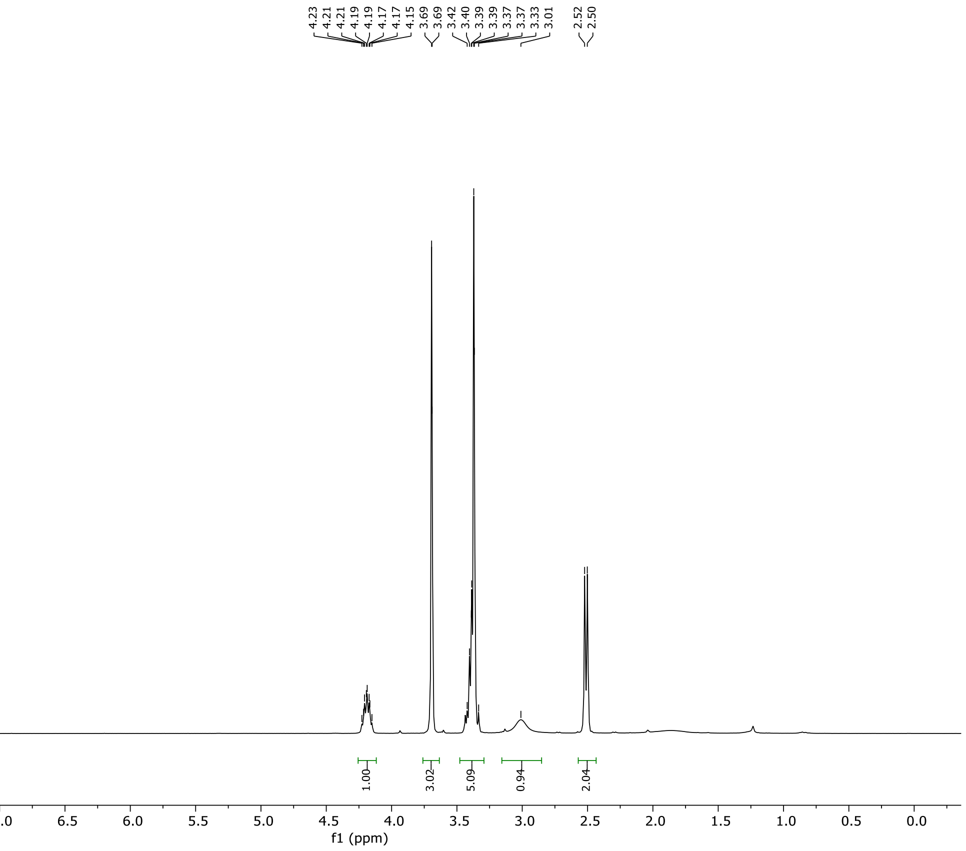

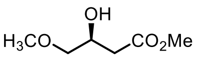

6c

$^{13}C\{^1H\}$ NMR (126 MHz)

$CDCl_3$

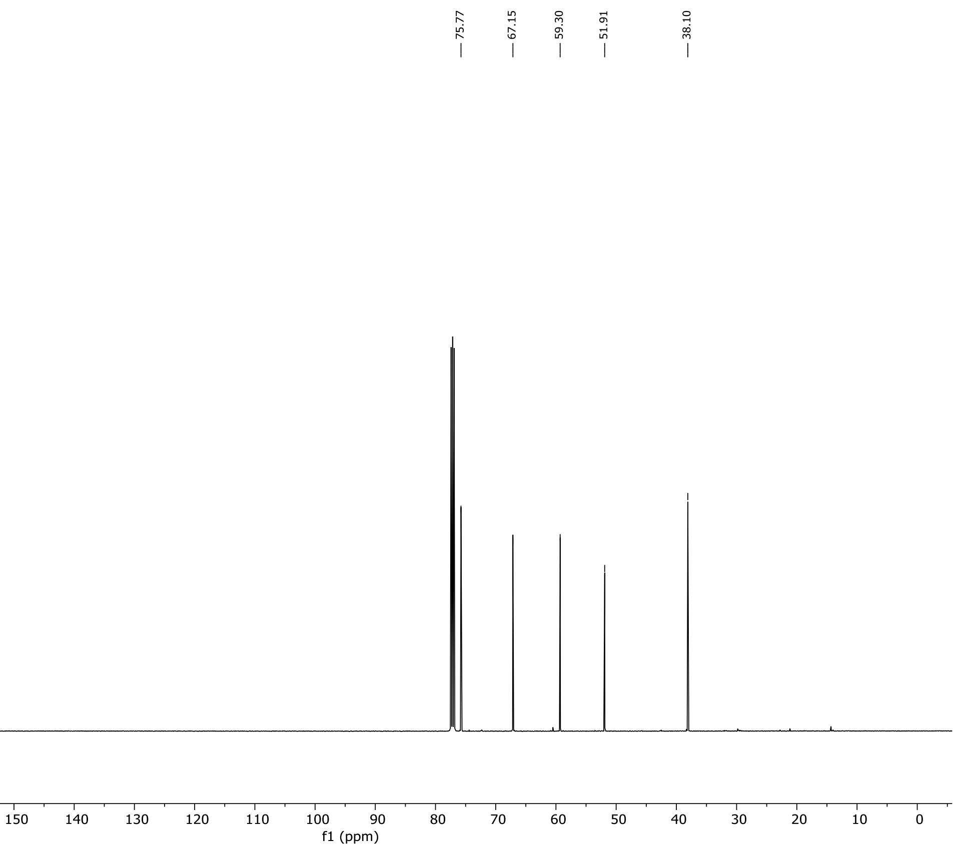

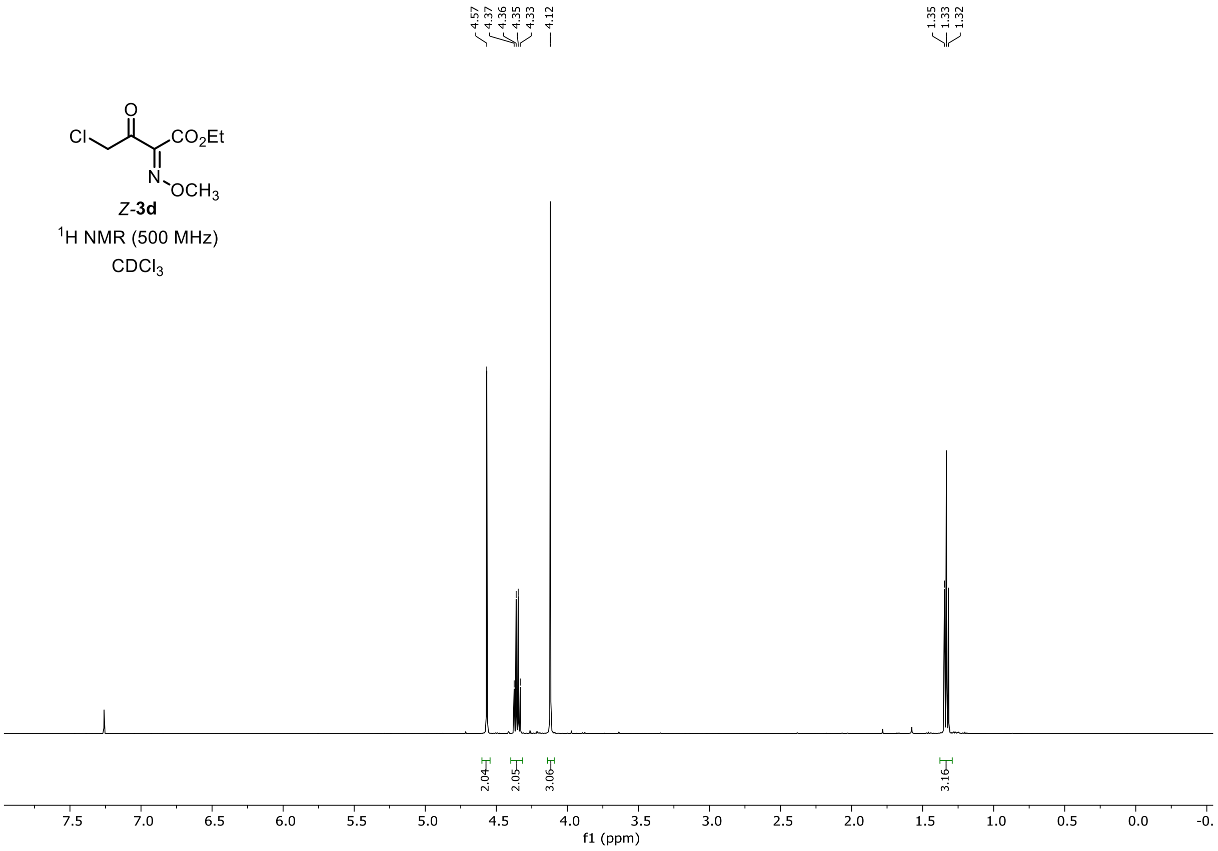
4.57
4.37
4.36
4.35
4.33
4.12
1.35
1.33
1.32
Cl
O
CO2Et
N
OCH3
Z-3d
1H NMR (500 MHz)
CDCl3
2.04
2.05
3.06
3.16
7.5 7.0 6.5 6.0 5.5 5.0 4.5 4.0 3.5 3.0 2.5 2.0 1.5 1.0 0.5 0.0 -0.
f1 (ppm)

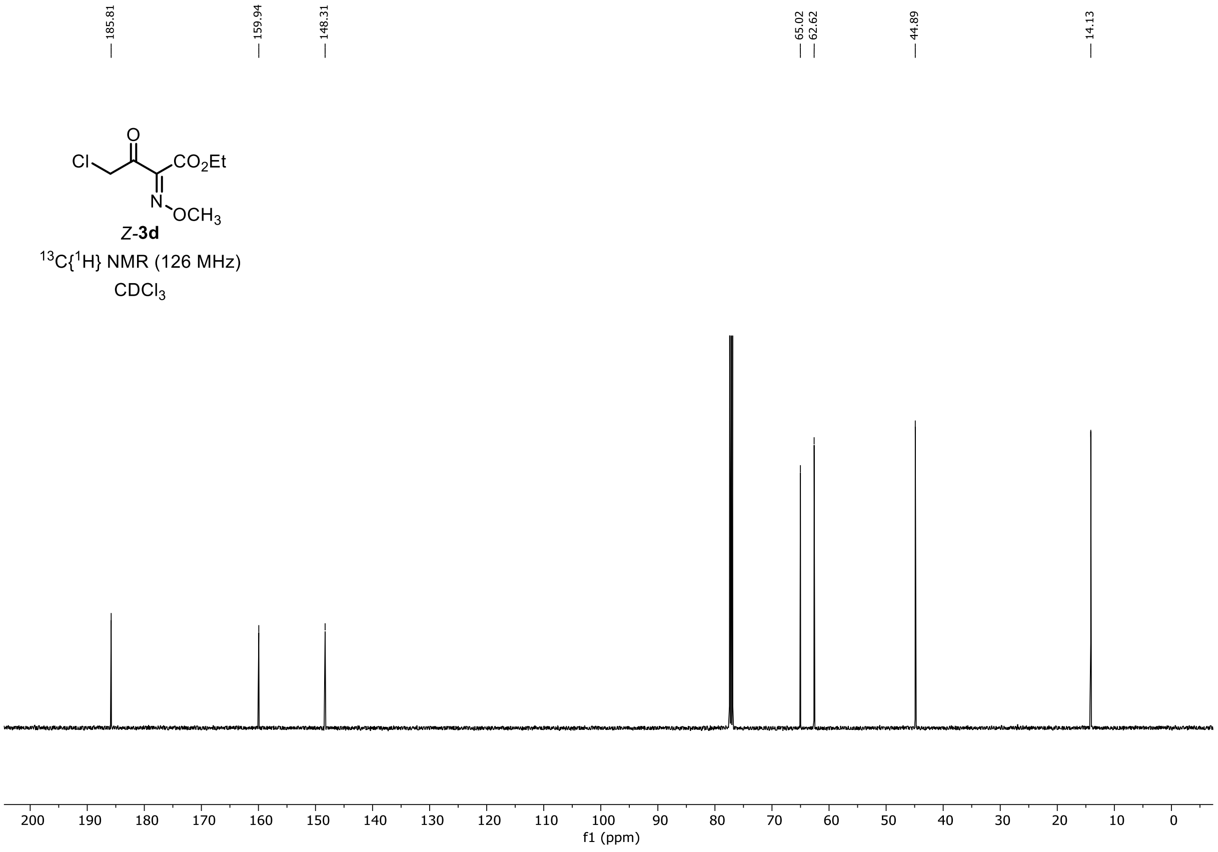
185.81
159.94
148.31
65.02
62.62
44.89
14.13
Cl
O
CO2Et
N
OCH3
Z-3d
13C{1H} NMR (126 MHz)
CDCl3
200 190 180 170 160 150 140 130 120 110 100 90 80 70 60 50 40 30 20 10 0
f1 (ppm)

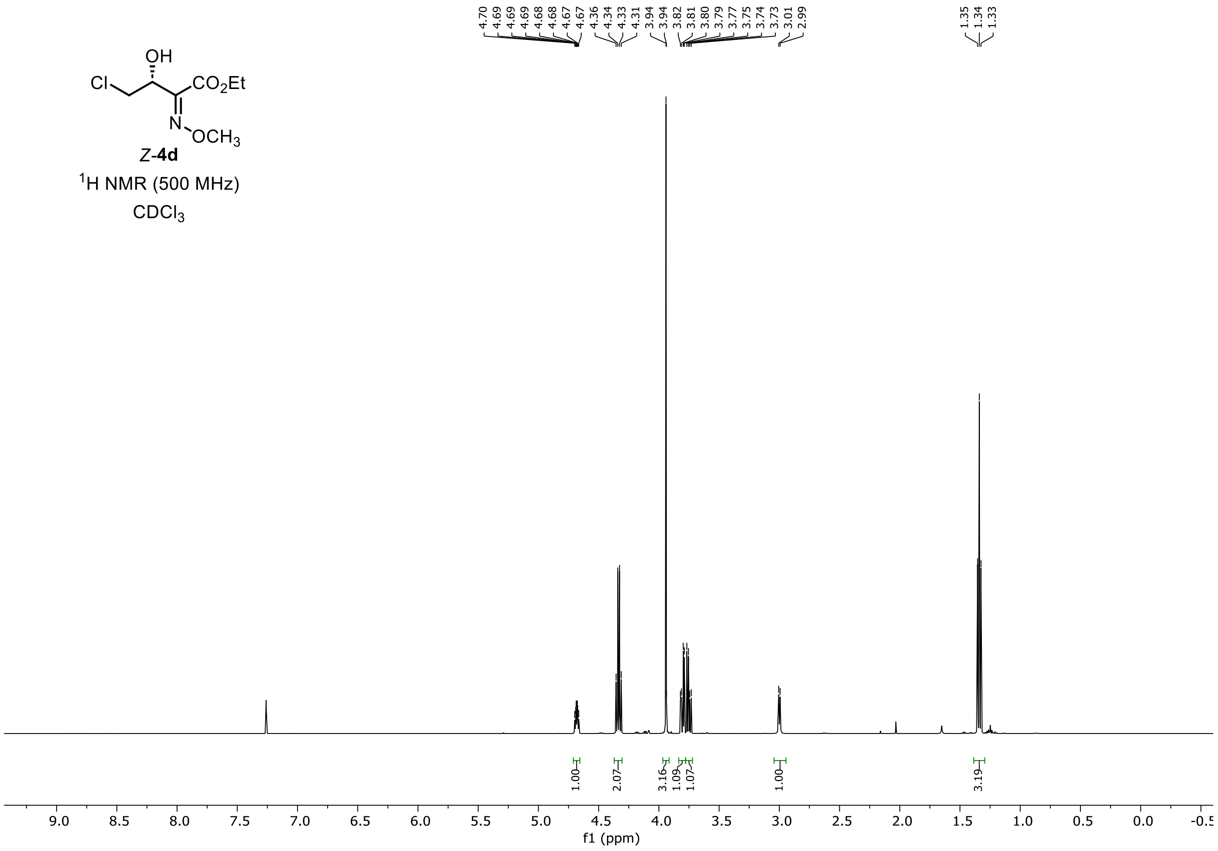
OH
Cl
CO2Et
N
OCH3
Z-4d
1H NMR (500 MHz)
CDCl3
f1 (ppm)

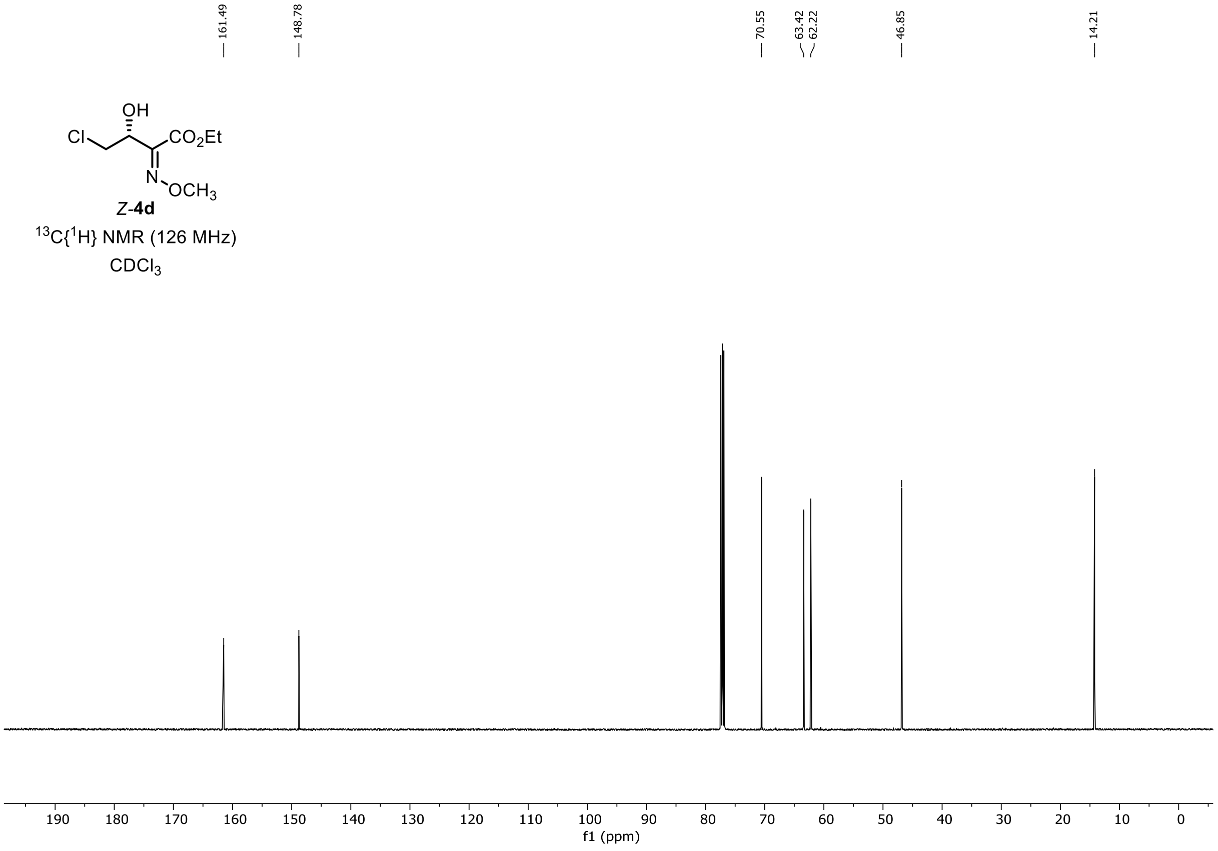
OH
Cl
CO2Et
N
OCH3
Z-4d
13C{1H} NMR (126 MHz)
CDCl3
161.49
148.78
70.55
63.42
62.22
46.85
14.21
f1 (ppm)

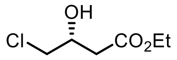

6d

^{1}H NMR (300 MHz)

$CDCl_3$

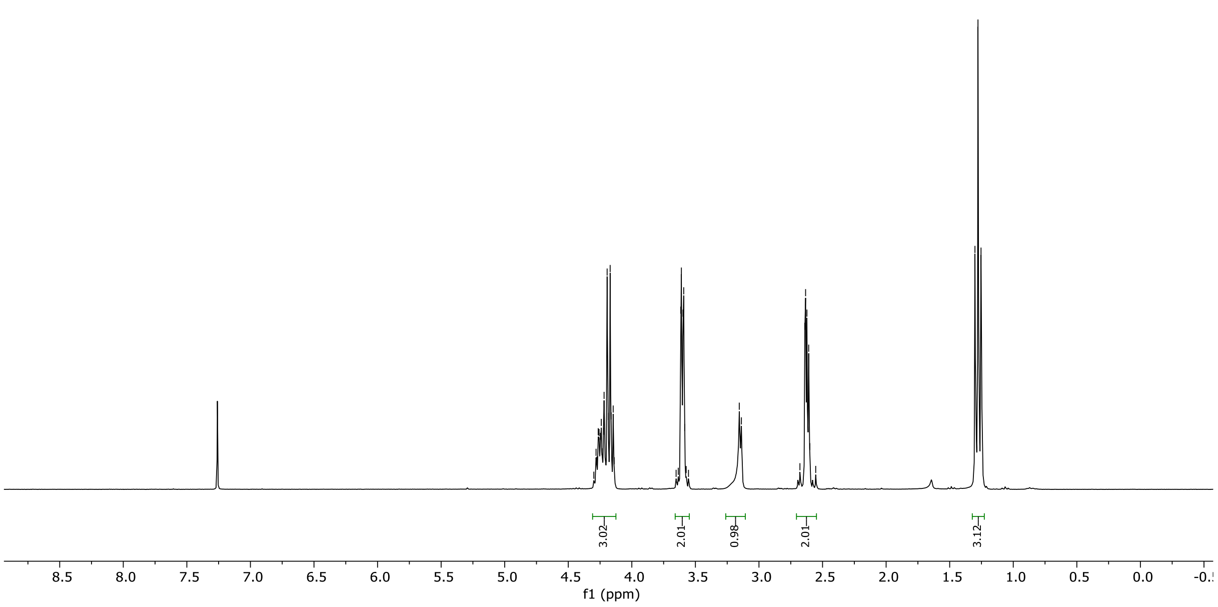

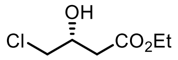

6d

$^{13}C\{^1H\}$ NMR (126 MHz)

$CDCl_3$

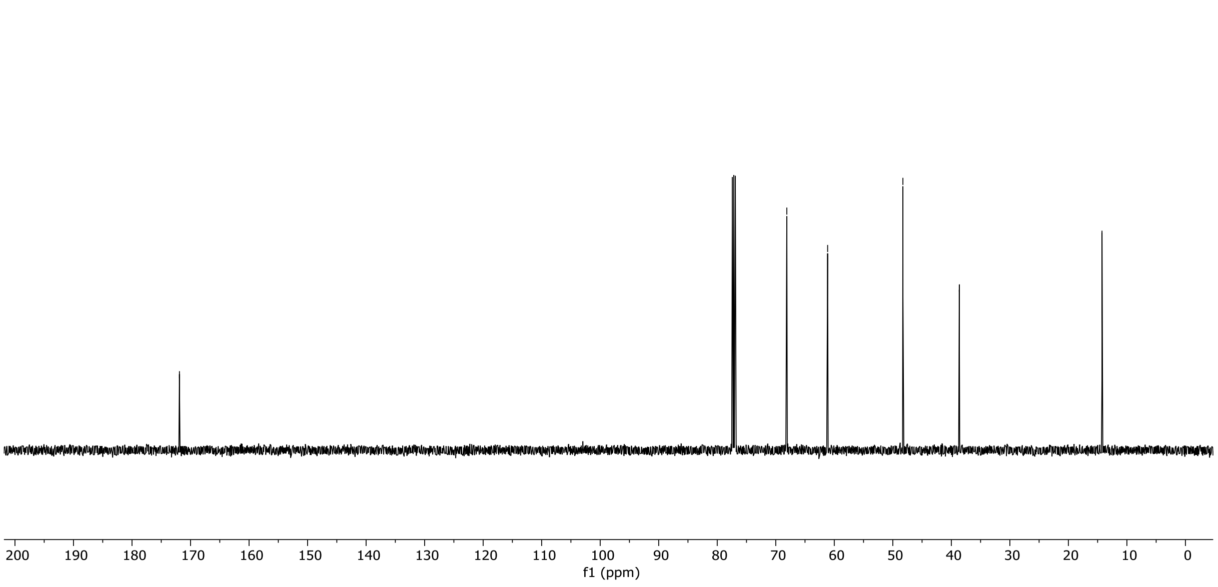

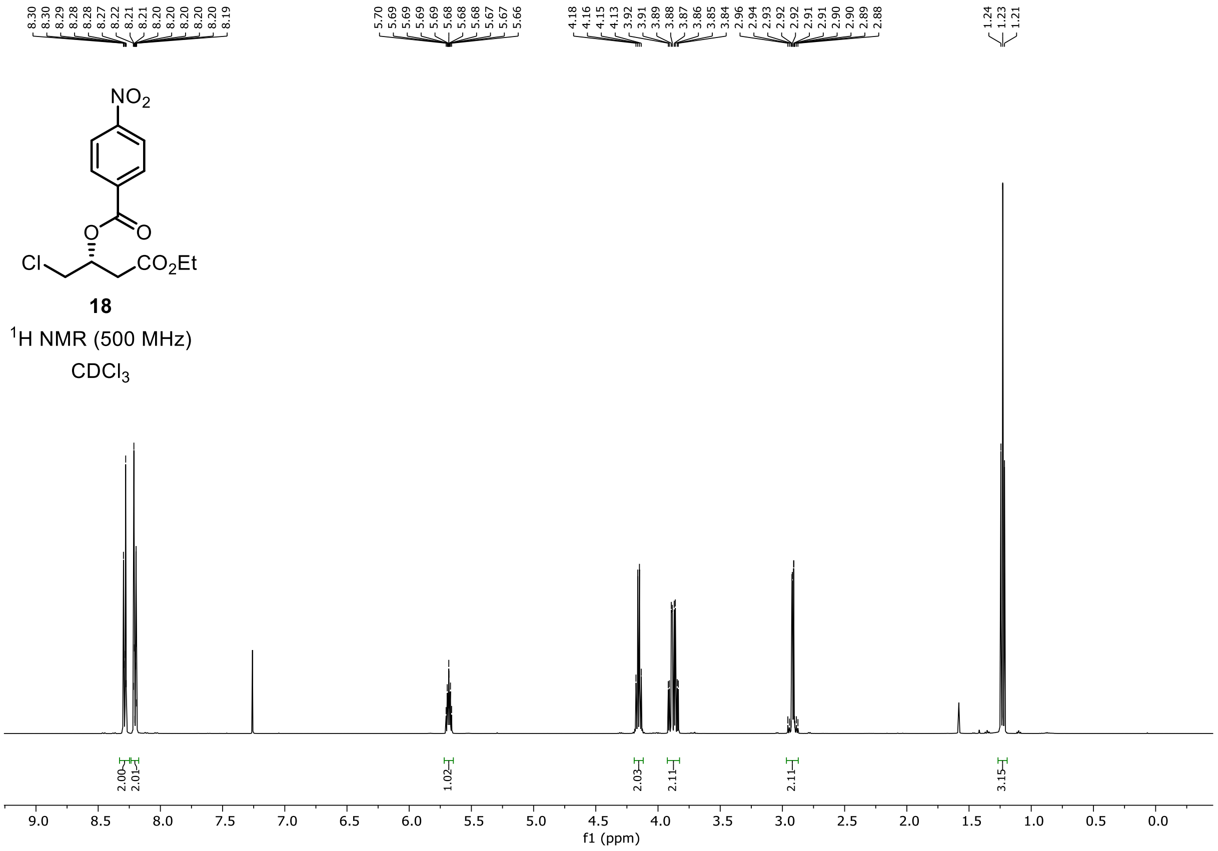
8.30 8.30 8.29 8.28 8.28 8.27 8.22 8.21 8.21 8.20 8.20 8.20 8.20 8.20 8.19
5.70 5.69 5.69 5.69 5.69 5.68 5.68 5.68 5.68 5.67 5.67 5.66
4.18 4.16 4.15 4.13 3.92 3.91 3.89 3.88 3.87 3.86 3.85 3.84 2.96 2.94 2.93 2.92 2.92 2.91 2.91 2.90 2.90 2.89 2.88
1.24 1.23 1.21
NO2
O
O
Cl
CO2Et
18
1H NMR (500 MHz)
CDCl3
2.00 2.01 1.02 2.03 2.11 2.11 3.15
f1 (ppm)

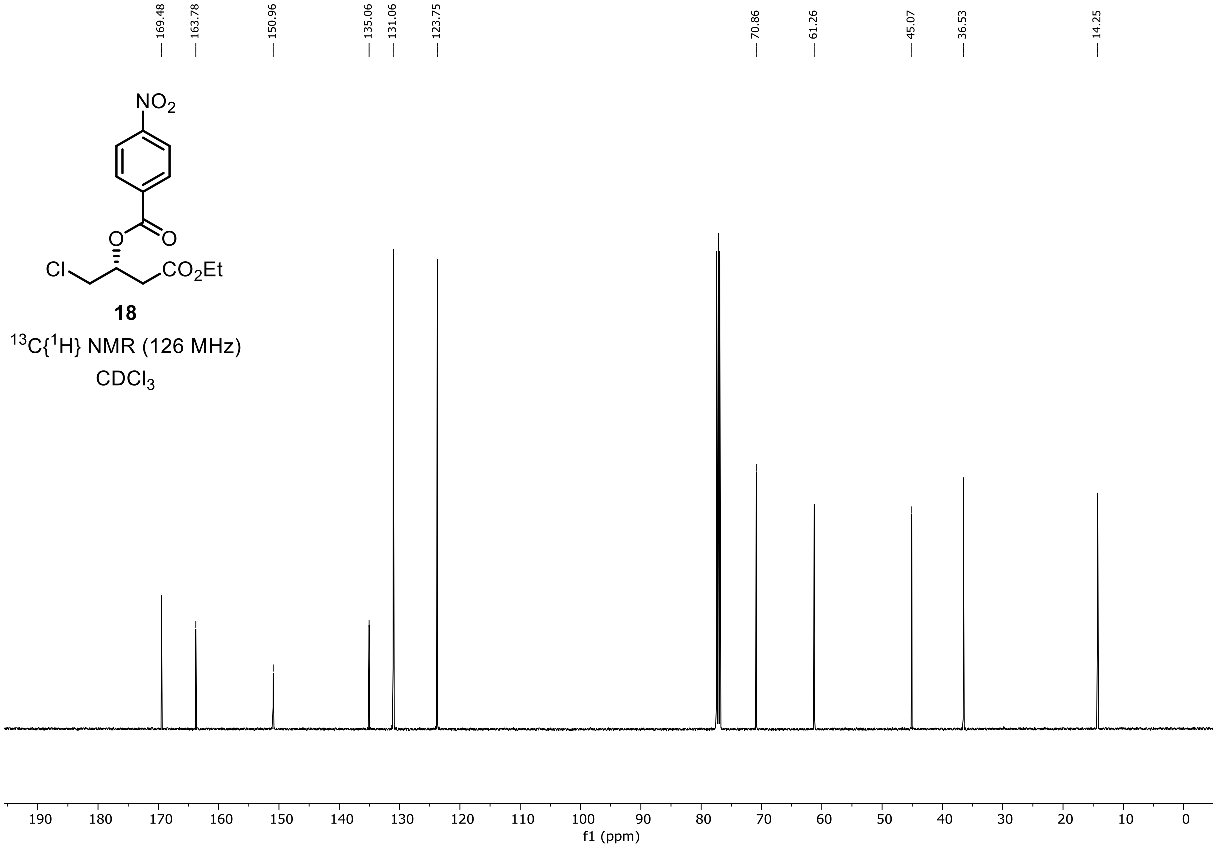
169.48 163.78 150.96 135.06 131.06 123.75 70.86 61.26 45.07 36.53 14.25
NO2
O
O
Cl
CO2Et
18
13C{1H} NMR (126 MHz)
CDCl3
f1 (ppm)

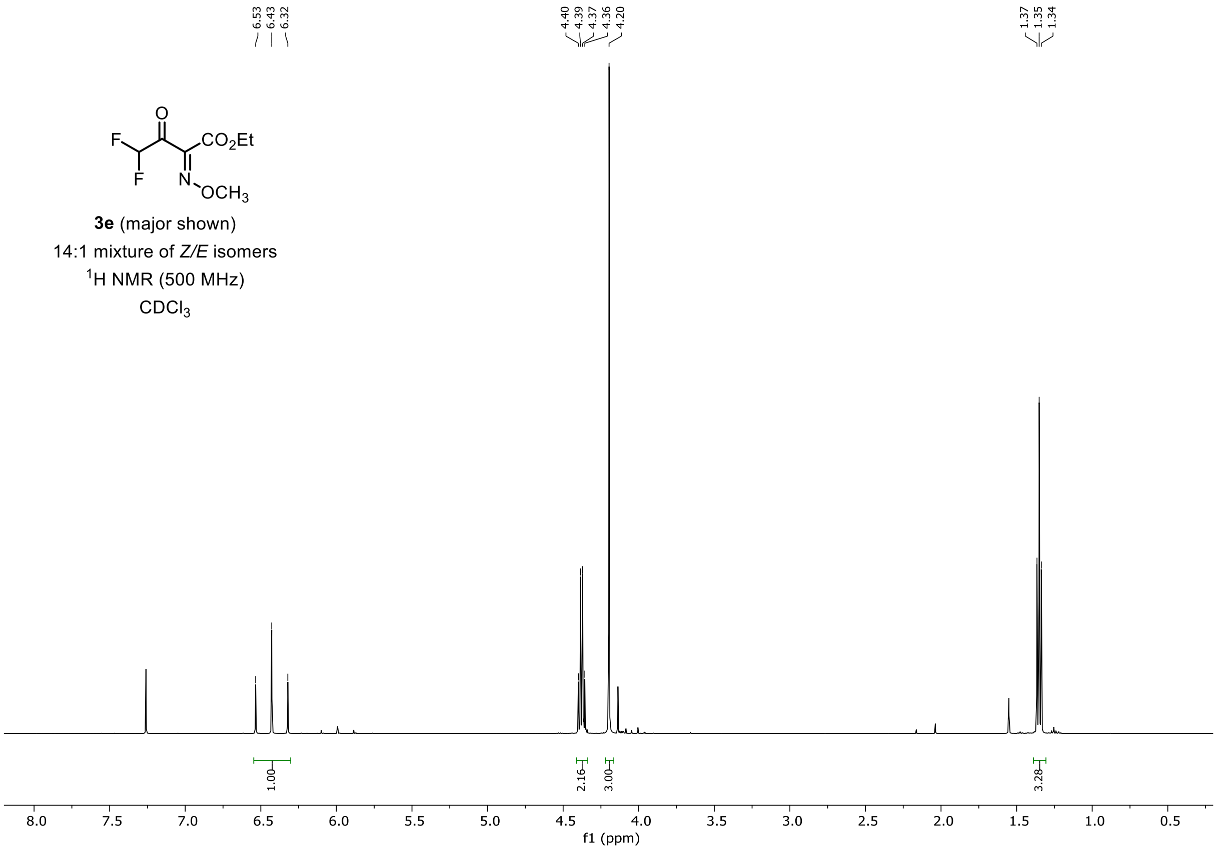
6.53
6.43
6.32
4.40
4.39
4.37
4.36
4.20
1.37
1.35
1.34
O
F
CO2Et
F
N
OCH3
3e (major shown)
14:1 mixture of Z/E isomers
1H NMR (500 MHz)
CDCl3
1.00
2.16
3.00
3.28
8.0 7.5 7.0 6.5 6.0 5.5 5.0 4.5 4.0 3.5 3.0 2.5 2.0 1.5 1.0 0.5
f1 (ppm)

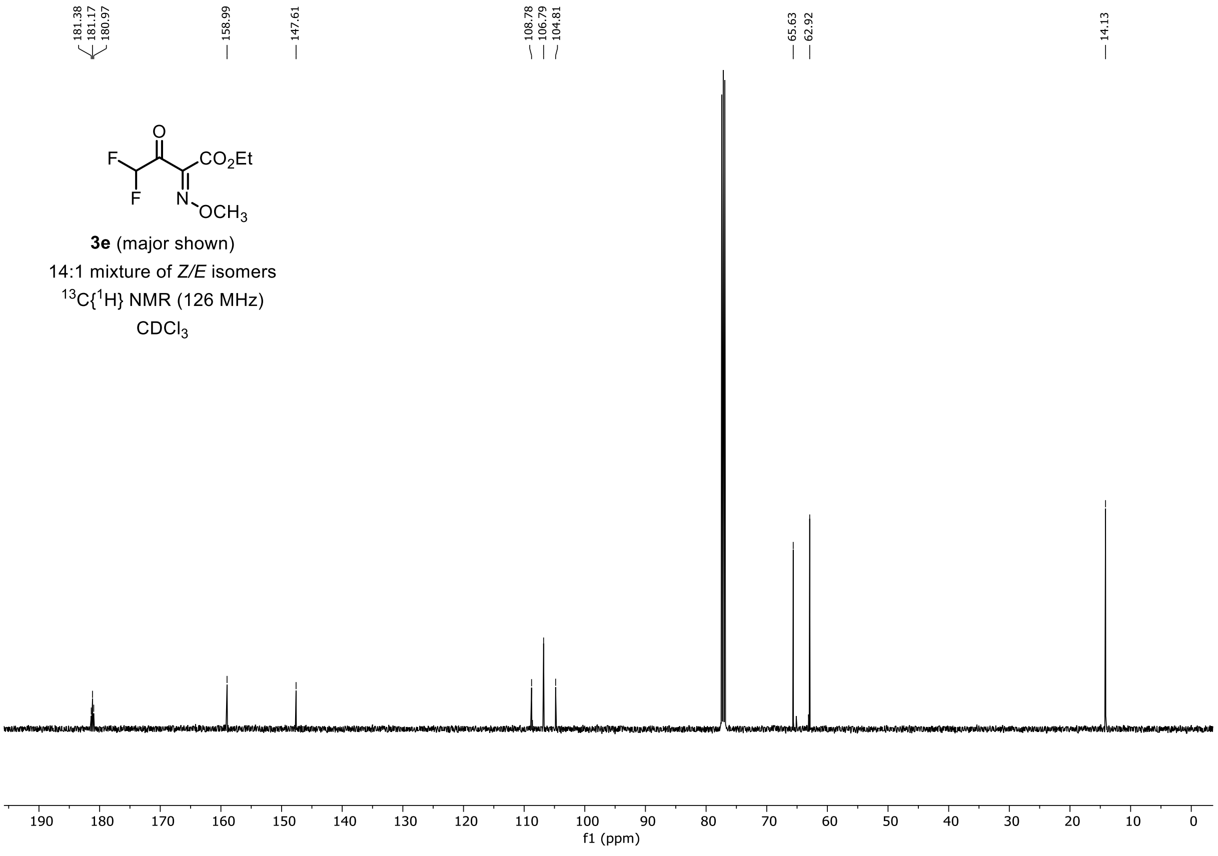
181.38
181.17
180.97
158.99
147.61
108.78
106.79
104.81
65.63
62.92
14.13
O
F
CO2Et
F
N
OCH3
3e (major shown)
14:1 mixture of Z/E isomers
13C{1H} NMR (126 MHz)
CDCl3
190 180 170 160 150 140 130 120 110 100 90 80 70 60 50 40 30 20 10 0
f1 (ppm)

4e (major shown)
21:1 mixture of *Z/E* isomers
^{1}H NMR (500 MHz)
$CDCl_3$

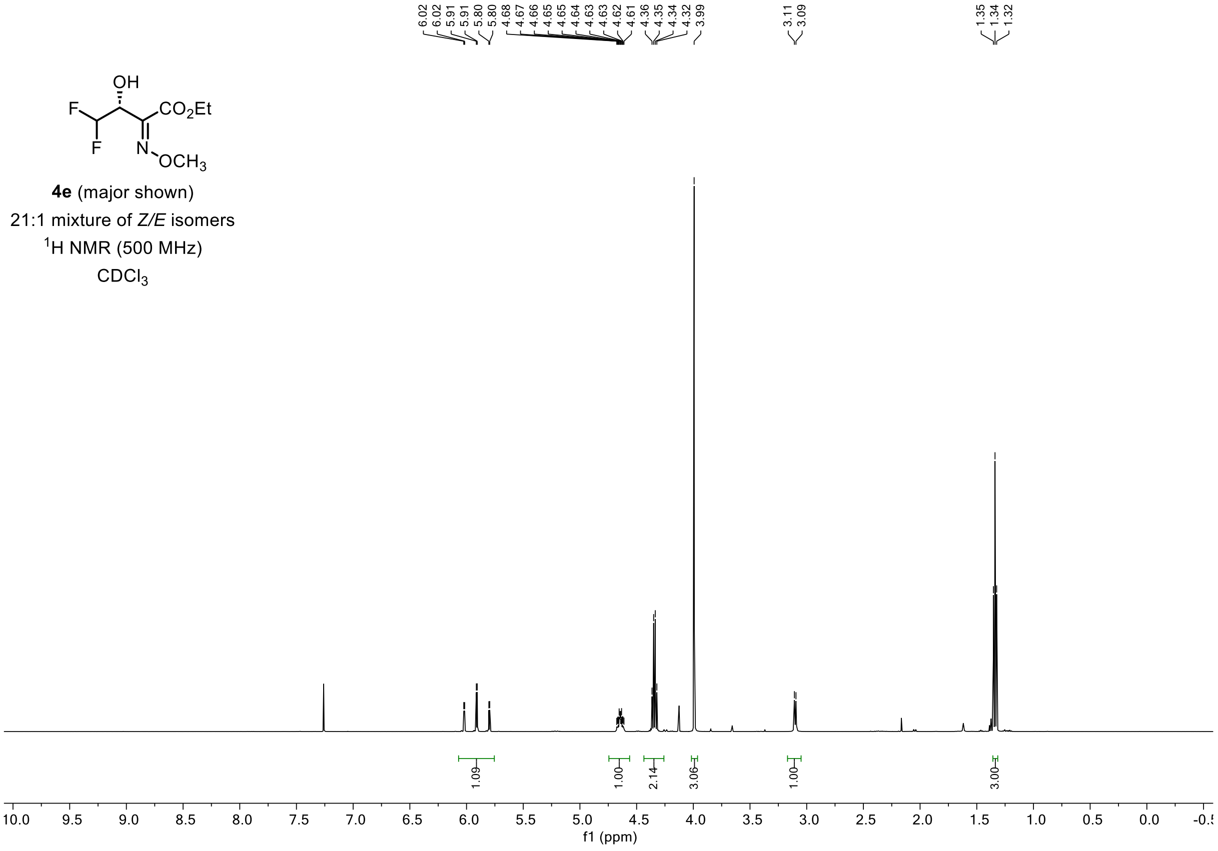

4e (major shown)
21:1 mixture of *Z/E* isomers
$^{13}C\{^{1}H\}$ NMR (126 MHz)
$CDCl_3$

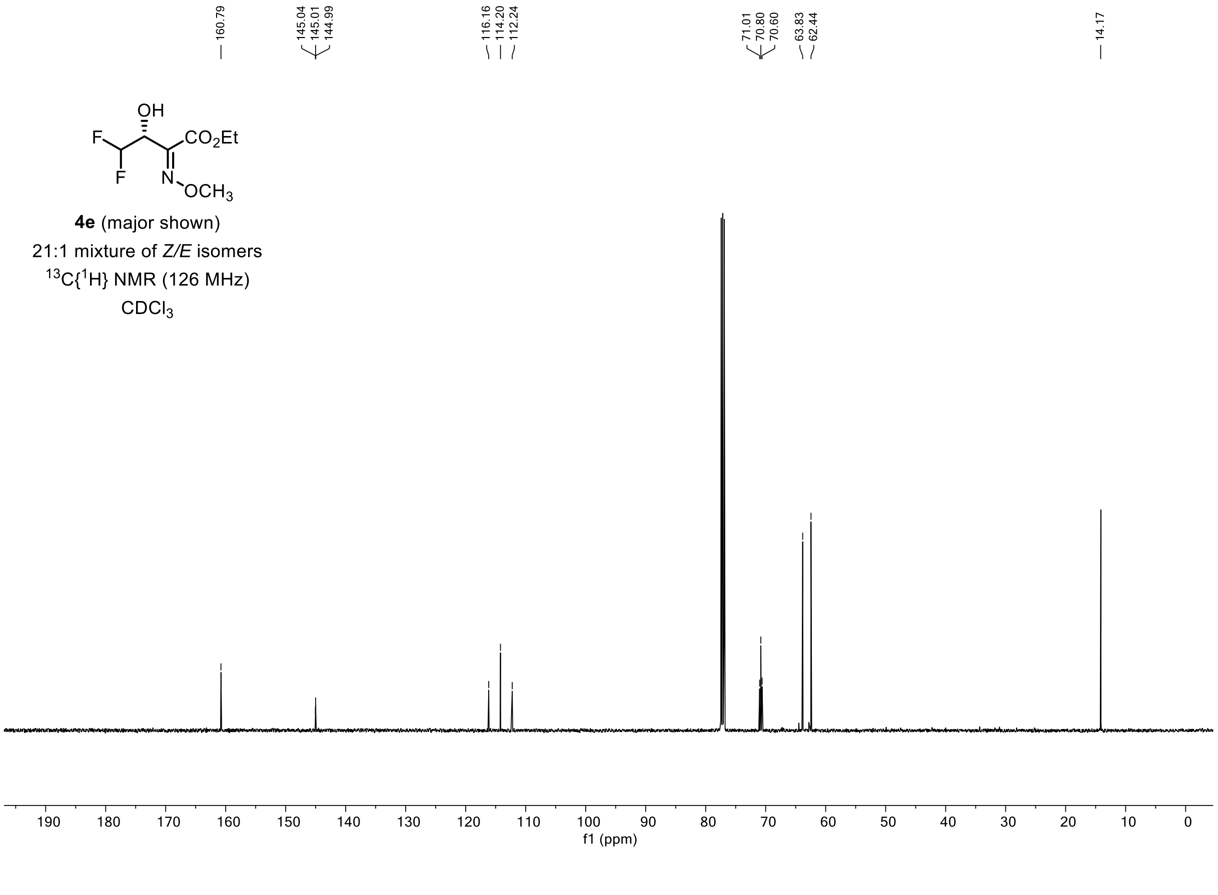

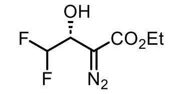

19 (ca. 85% purity)
^{1}H NMR (500 MHz)
$CDCl_3$

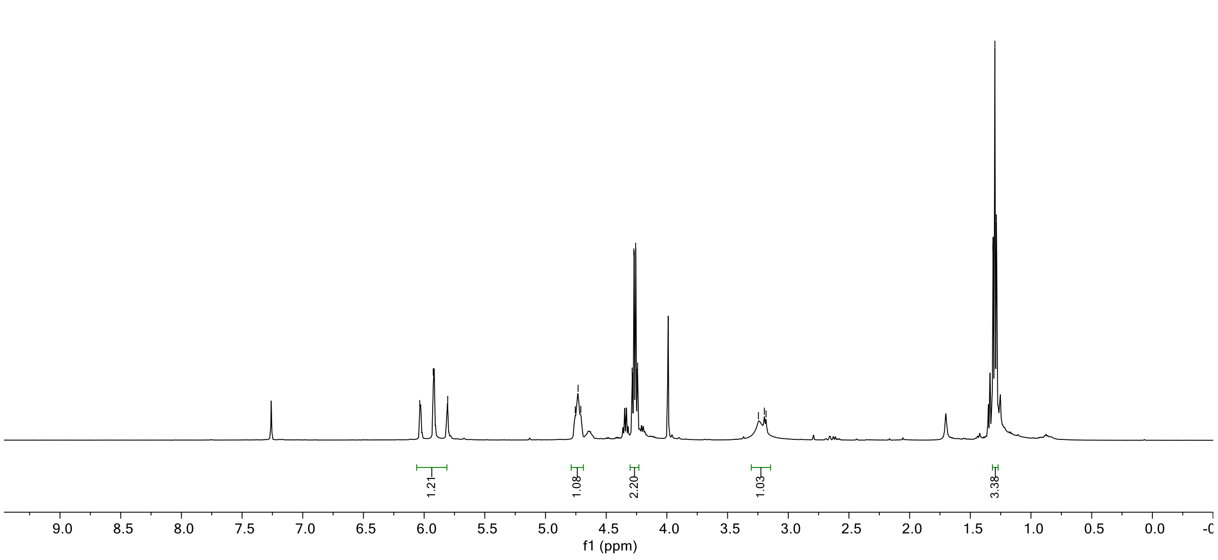

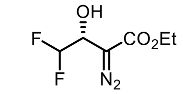

19 (ca. 85% purity)
$^{13}C\{^{1}H\}$ NMR (126 MHz)
$CDCl_3$

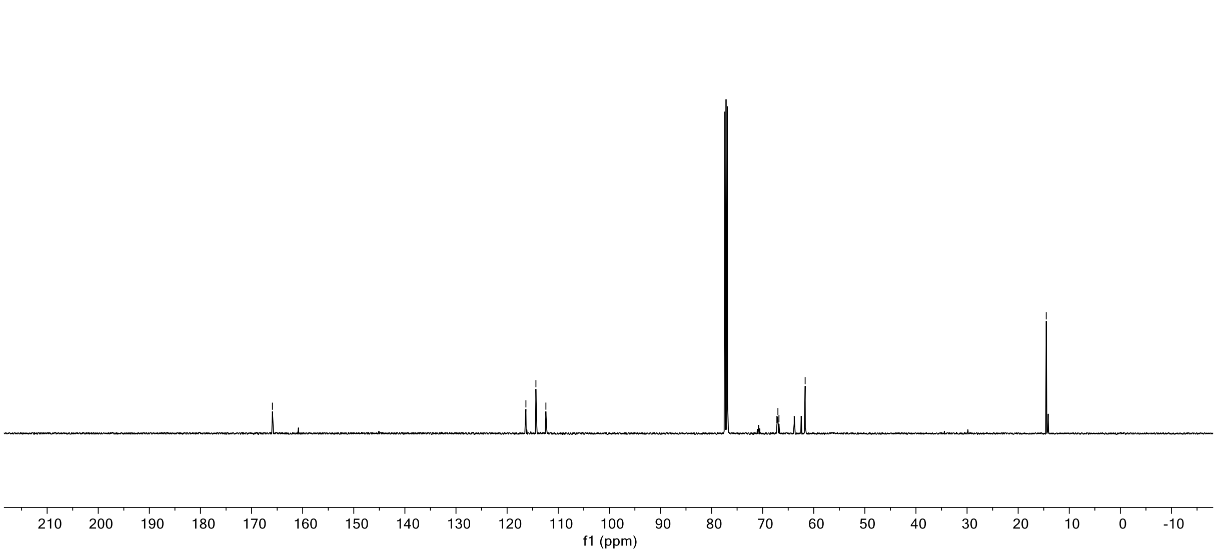

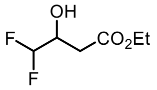

6e

^{1}H NMR (500 MHz)

$CDCl_3$

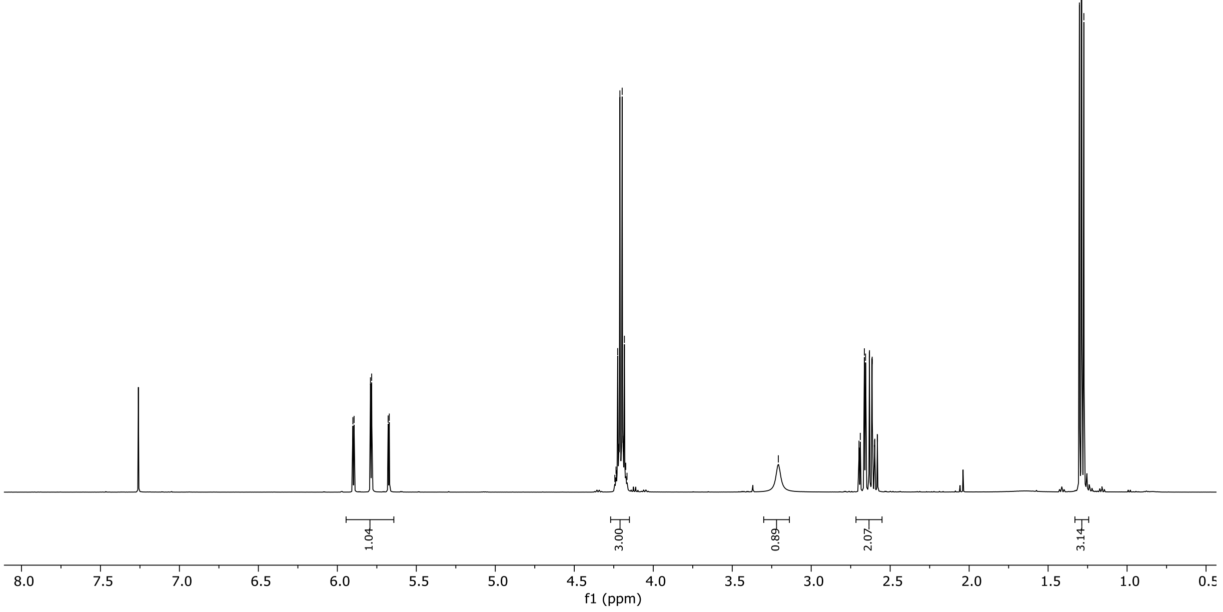

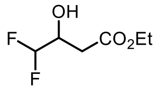

6e

$^{13}C\{^{1}H\}$ NMR (126 MHz)

$CDCl_3$

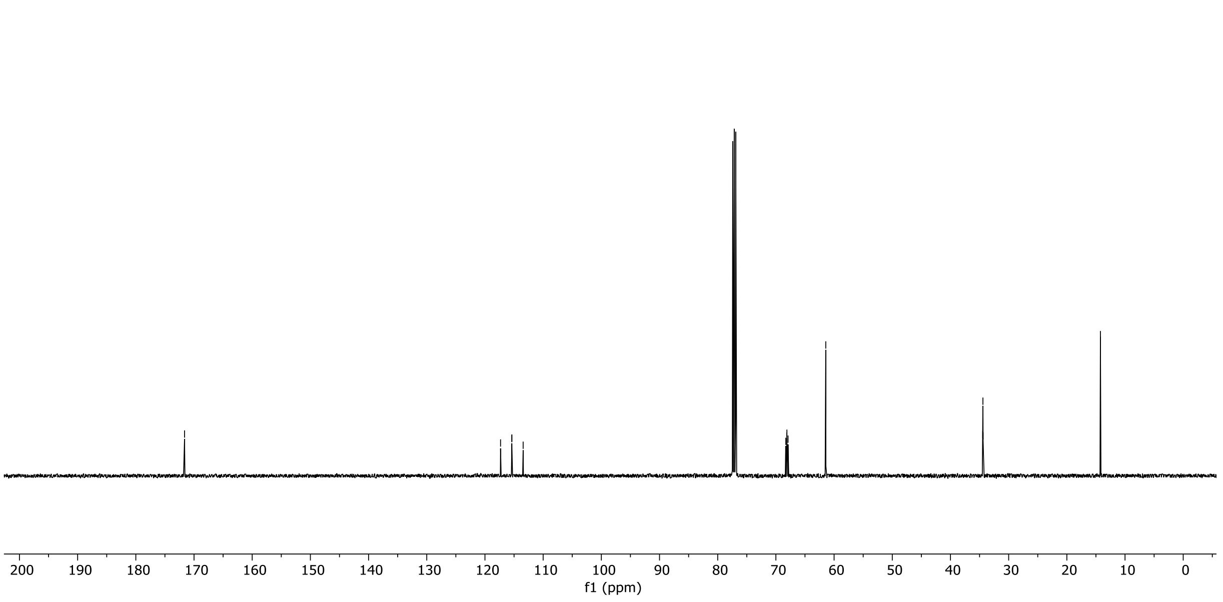

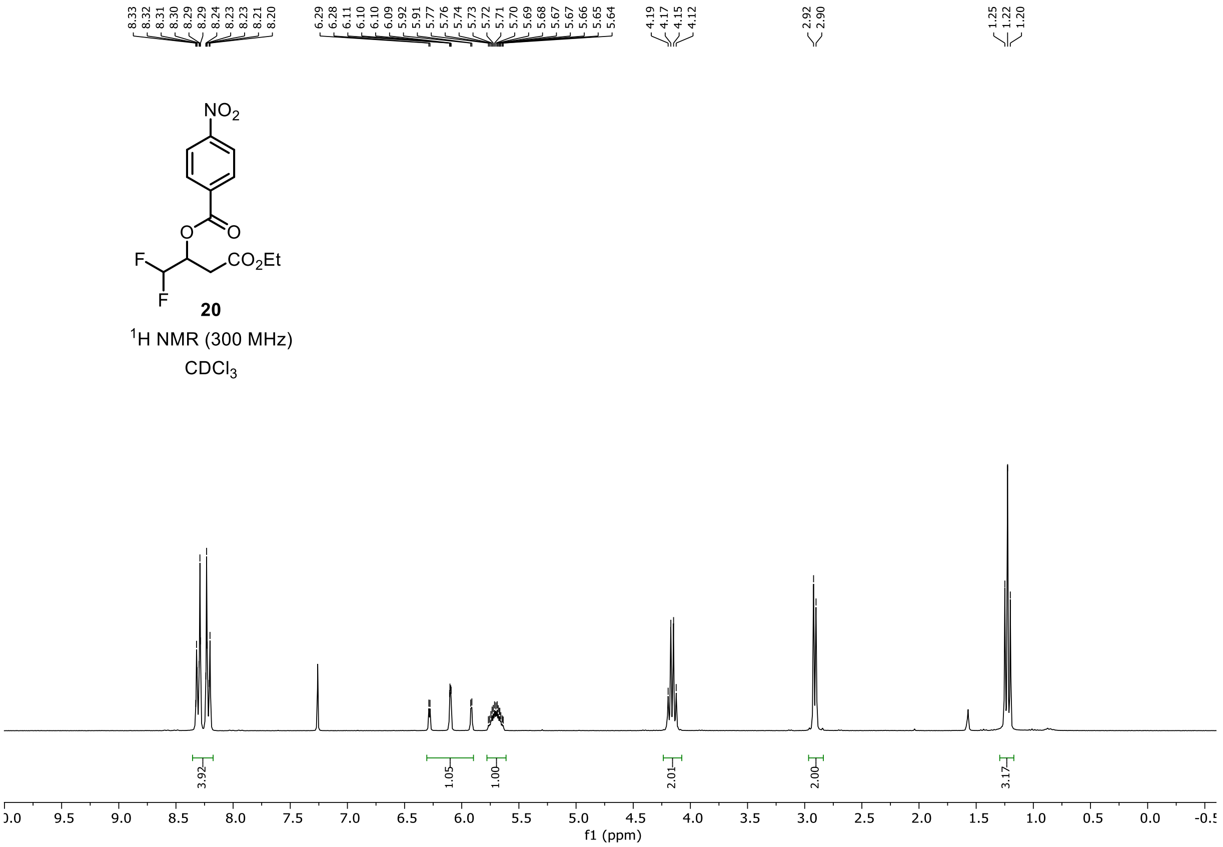
8.33 8.32 8.31 8.30 8.29 8.29 8.24 8.23 8.23 8.21 8.20
6.29 6.28 6.11 6.10 6.10 6.09 5.92 5.91 5.77 5.76 5.74 5.73 5.72 5.71 5.70 5.69 5.68 5.67 5.67 5.66 5.65 5.64
4.19 4.17 4.15 4.12
2.92 2.90
1.25 1.22 1.20
NO2
O
O
F
CO2Et
F
20
1H NMR (300 MHz)
CDCl3
3.92
1.05
1.00
2.01
2.00
3.17
f1 (ppm)

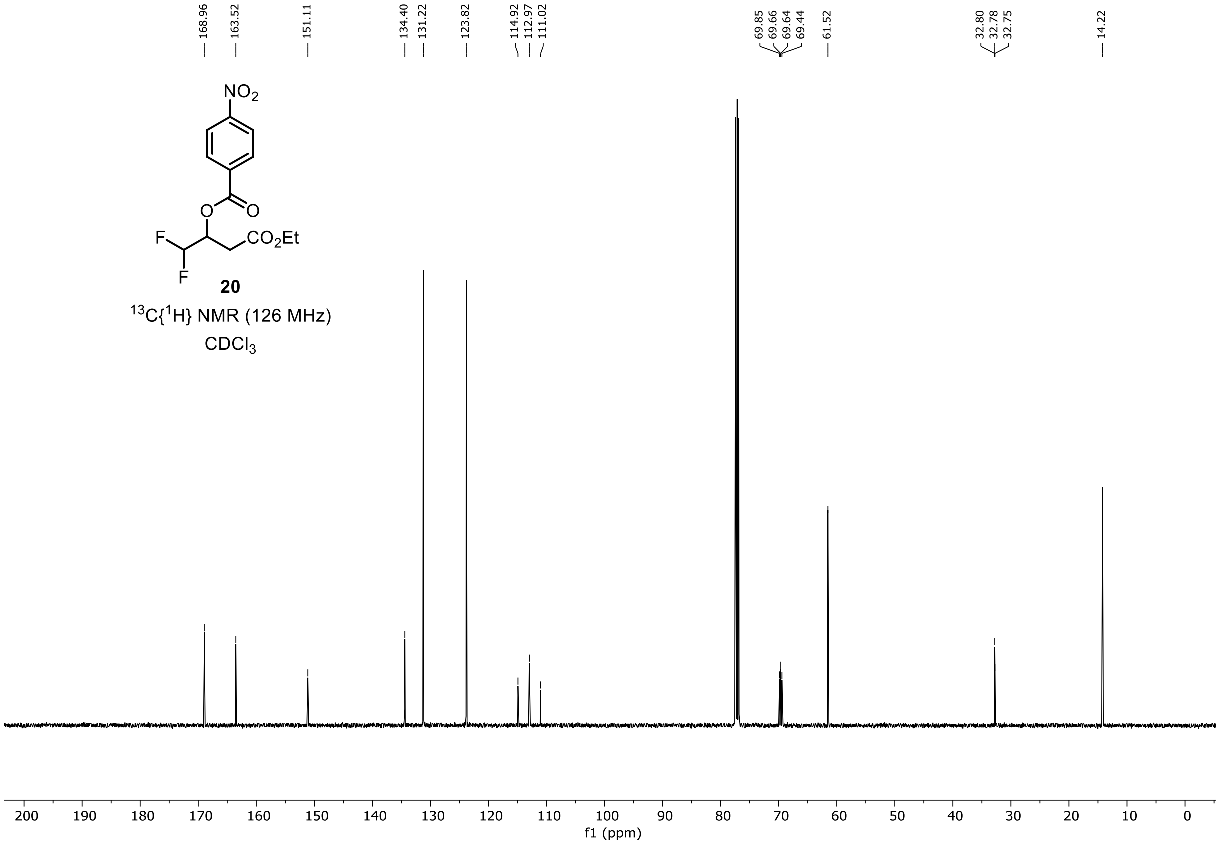
168.96
163.52
151.11
134.40
131.22
123.82
114.92 112.97 111.02
69.85 69.66 69.64 69.44
61.52
32.80 32.78 32.75
14.22
NO2
O
O
F
CO2Et
F
20
13C{1H} NMR (126 MHz)
CDCl3
f1 (ppm)

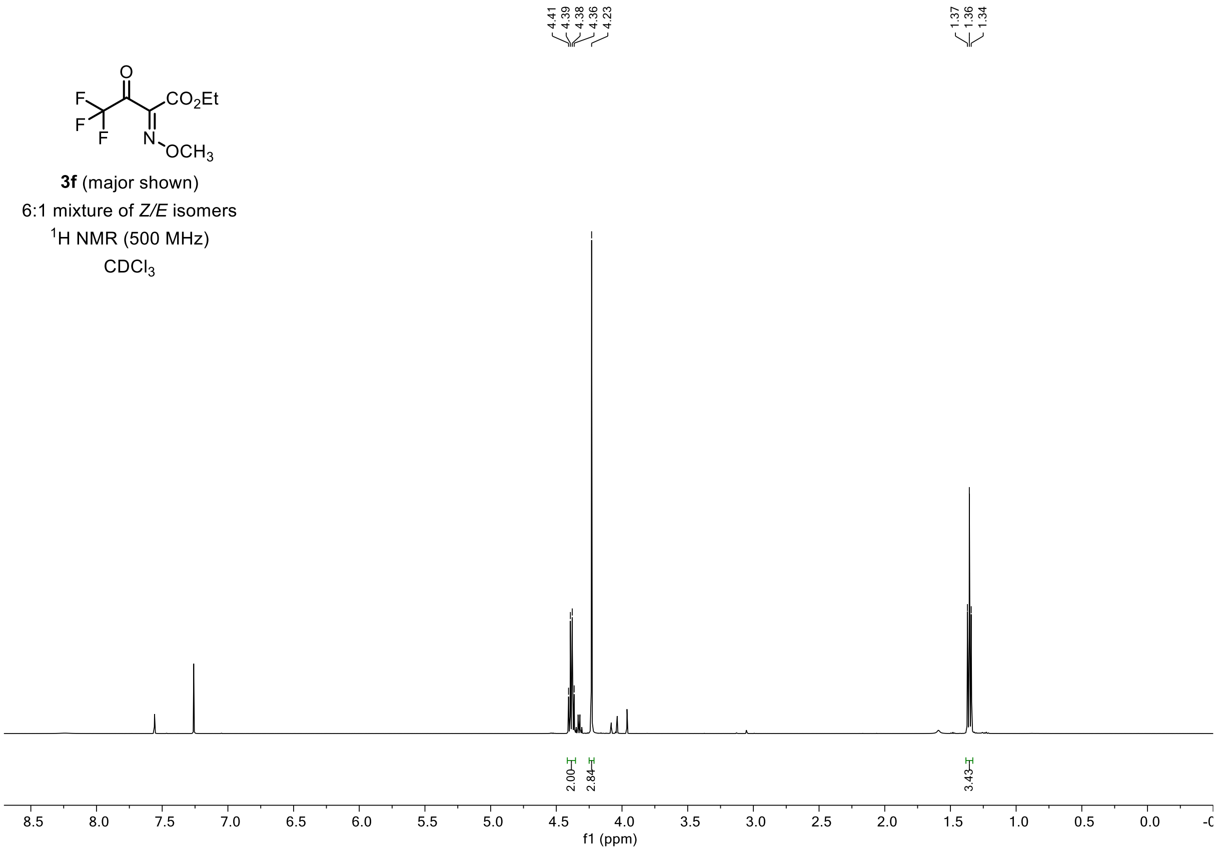
4.41
4.39
4.38
4.36
4.23
1.37
1.36
1.34
CO_2Et
F
O
N
OCH_3
3f (major shown)
6:1 mixture of *Z/E* isomers
^{1}H NMR (500 MHz)
$CDCl_3$
2.00
2.84
3.43
8.5 8.0 7.5 7.0 6.5 6.0 5.5 5.0 4.5 4.0 3.5 3.0 2.5 2.0 1.5 1.0 0.5 0.0
f1 (ppm)

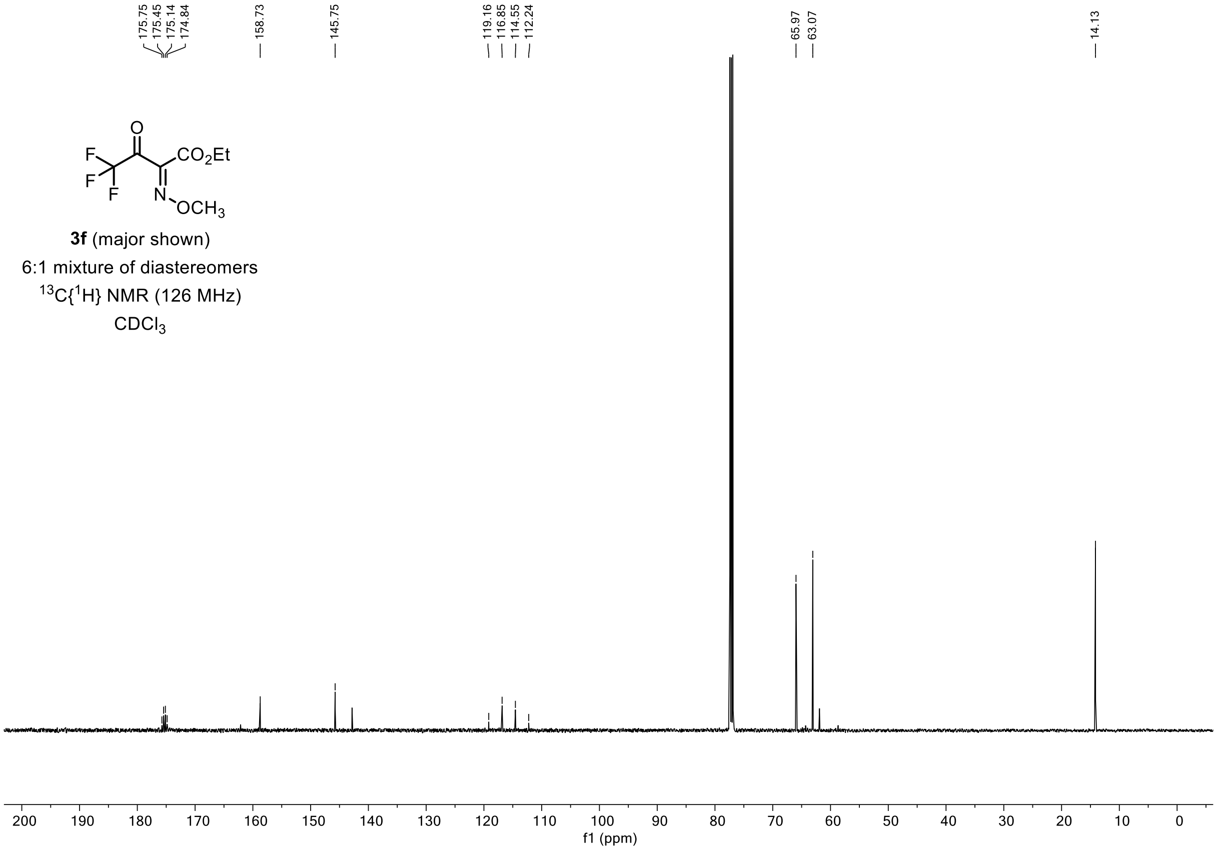
175.75
175.45
175.14
174.84
158.73
145.75
119.16
116.85
114.55
112.24
65.97
63.07
14.13
CO_2Et
F
O
N
OCH_3
3f (major shown)
6:1 mixture of diastereomers
$^{13}C\{^1H\}$ NMR (126 MHz)
$CDCl_3$
200 190 180 170 160 150 140 130 120 110 100 90 80 70 60 50 40 30 20 10 0
f1 (ppm)

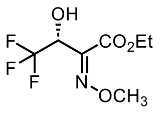

Z-**4f**
^{1}H NMR (500 MHz)
$CDCl_3$

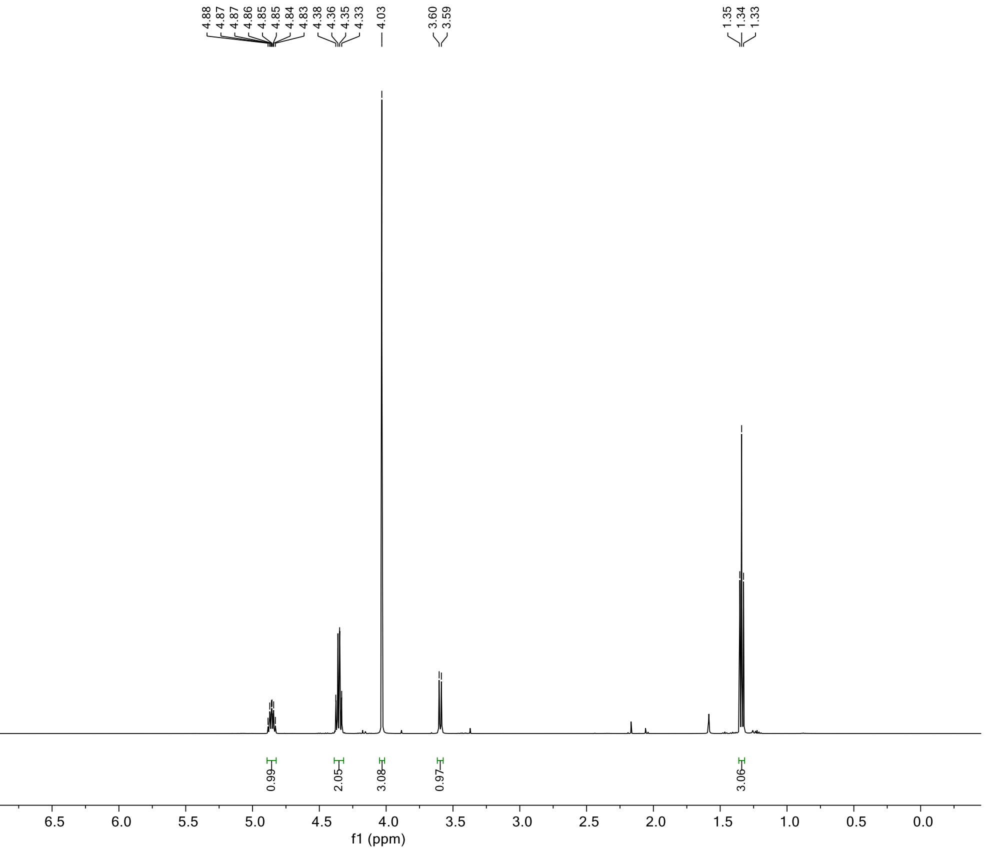

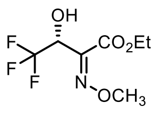

Z-**4f**
$^{13}C\{^1H\}$ NMR (126 MHz)
$CDCl_3$

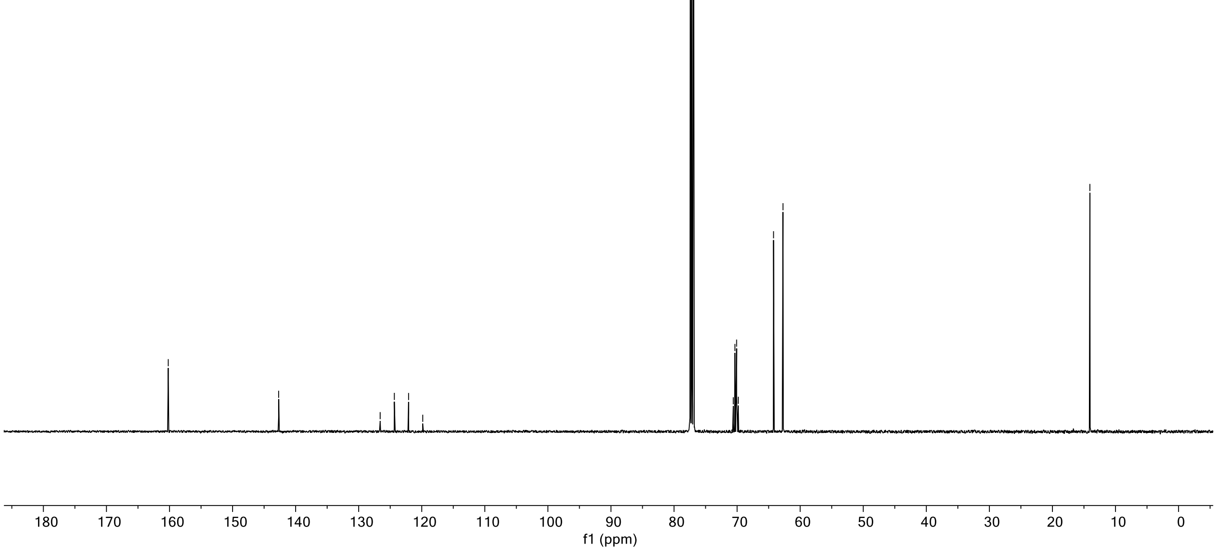

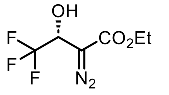

21

^{1}H NMR (500 MHz)

$CDCl_3$

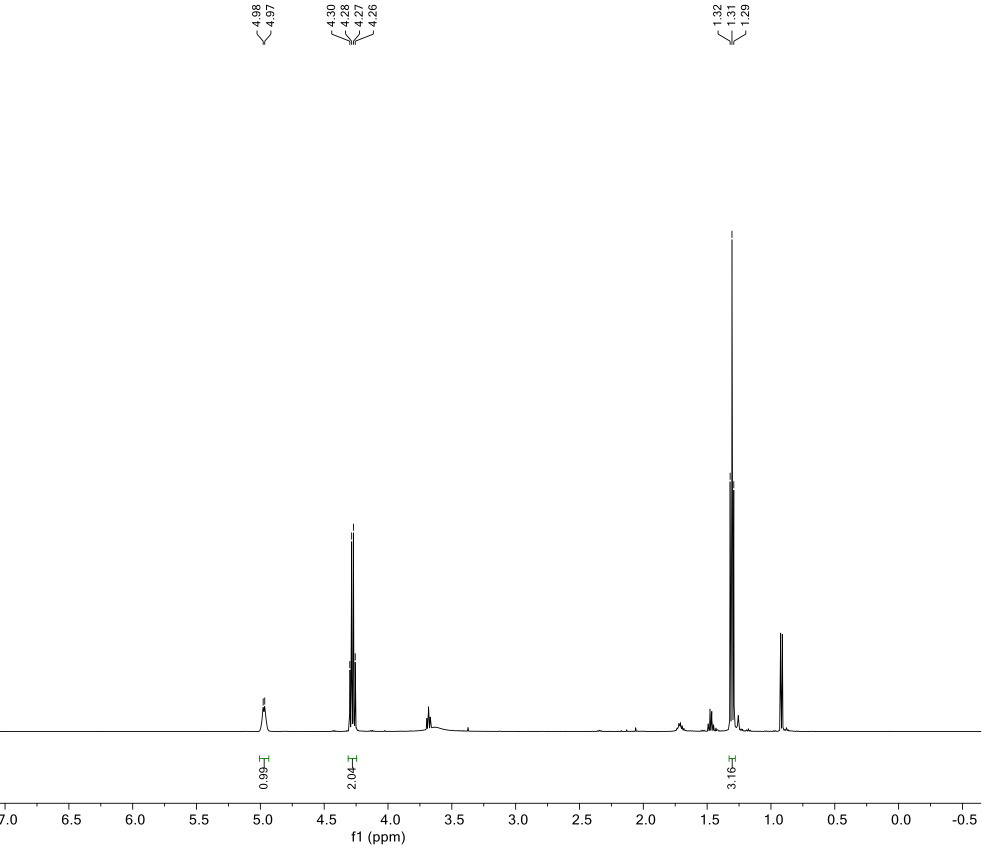

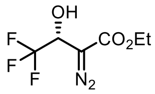

21

^{13}C{^{1}H} NMR (126 MHz)

$CDCl_3$

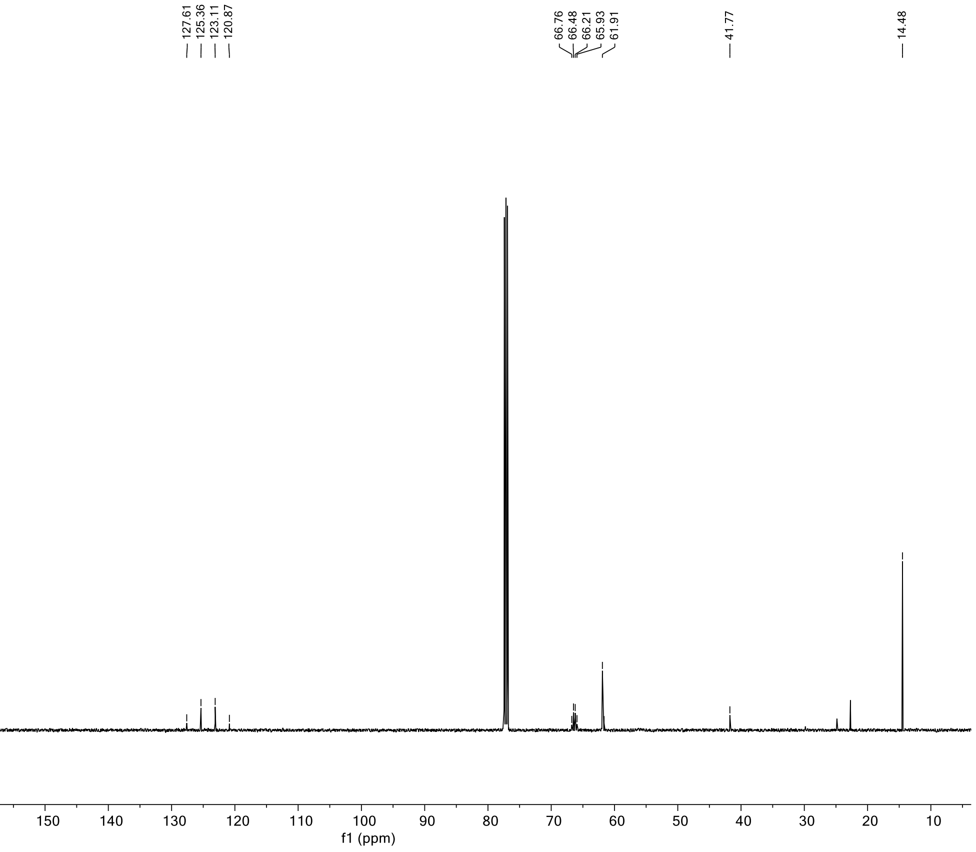

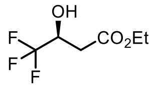

6f

^{1}H NMR (500 MHz)

$CDCl_3$

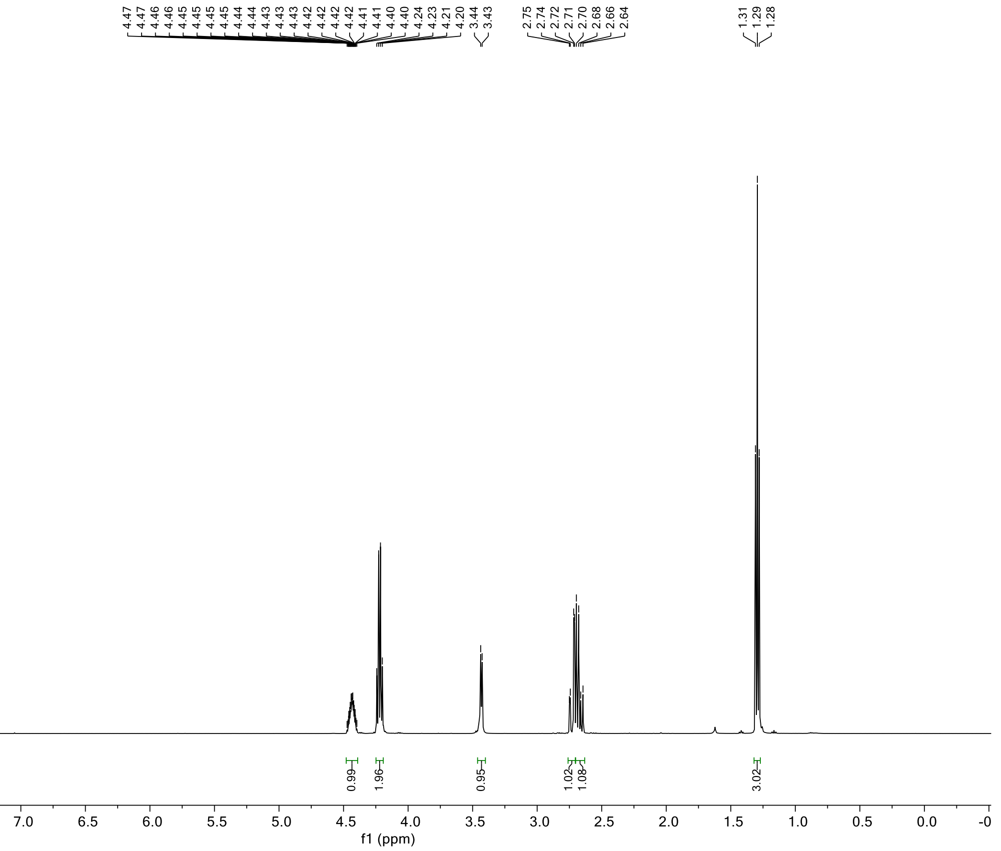

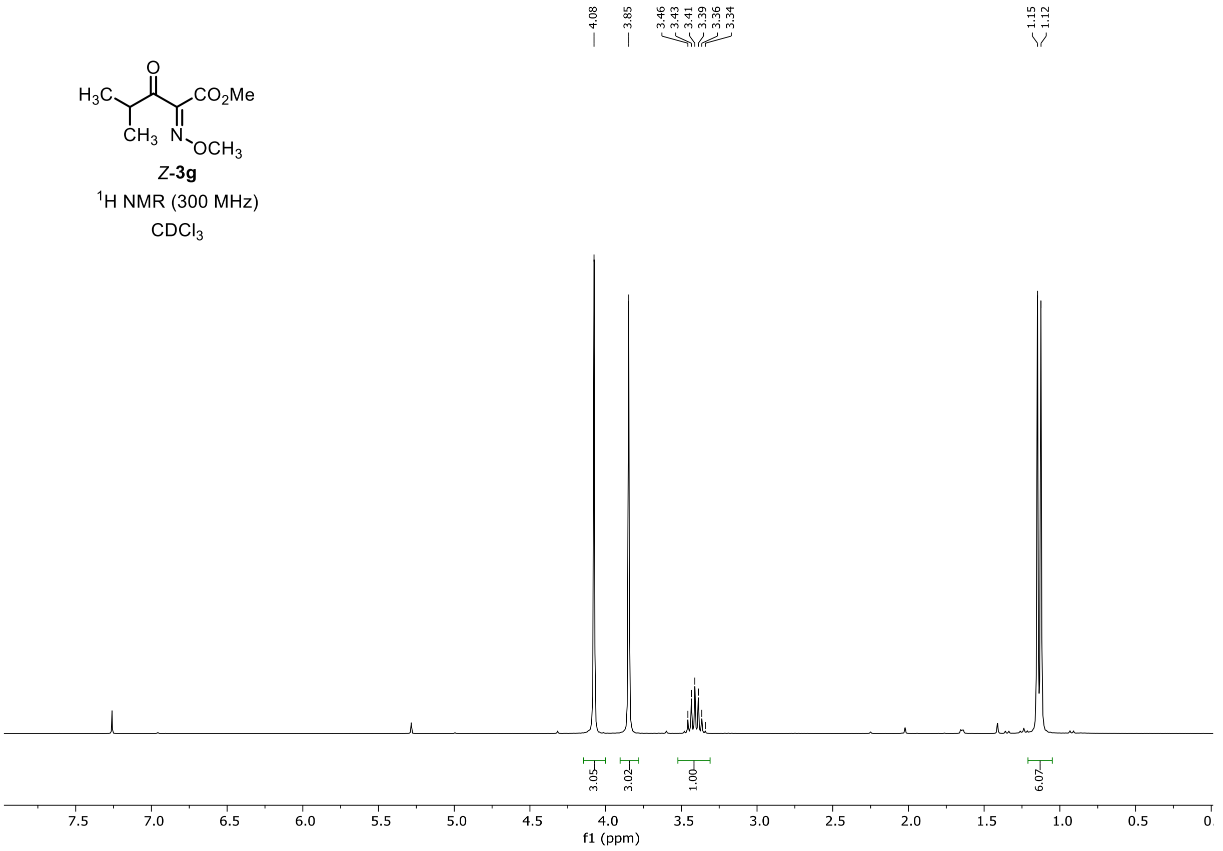

H3C
CO2Me
CH3
N
OCH3
Z-3g
1H NMR (300 MHz)
CDCl3
4.08
3.85
3.46
3.43
3.41
3.39
3.36
3.34
1.15
1.12
3.05
3.02
1.00
6.07
7.5
7.0
6.5
6.0
5.5
5.0
4.5
4.0
3.5
3.0
2.5
2.0
1.5
1.0
0.5
f1 (ppm)

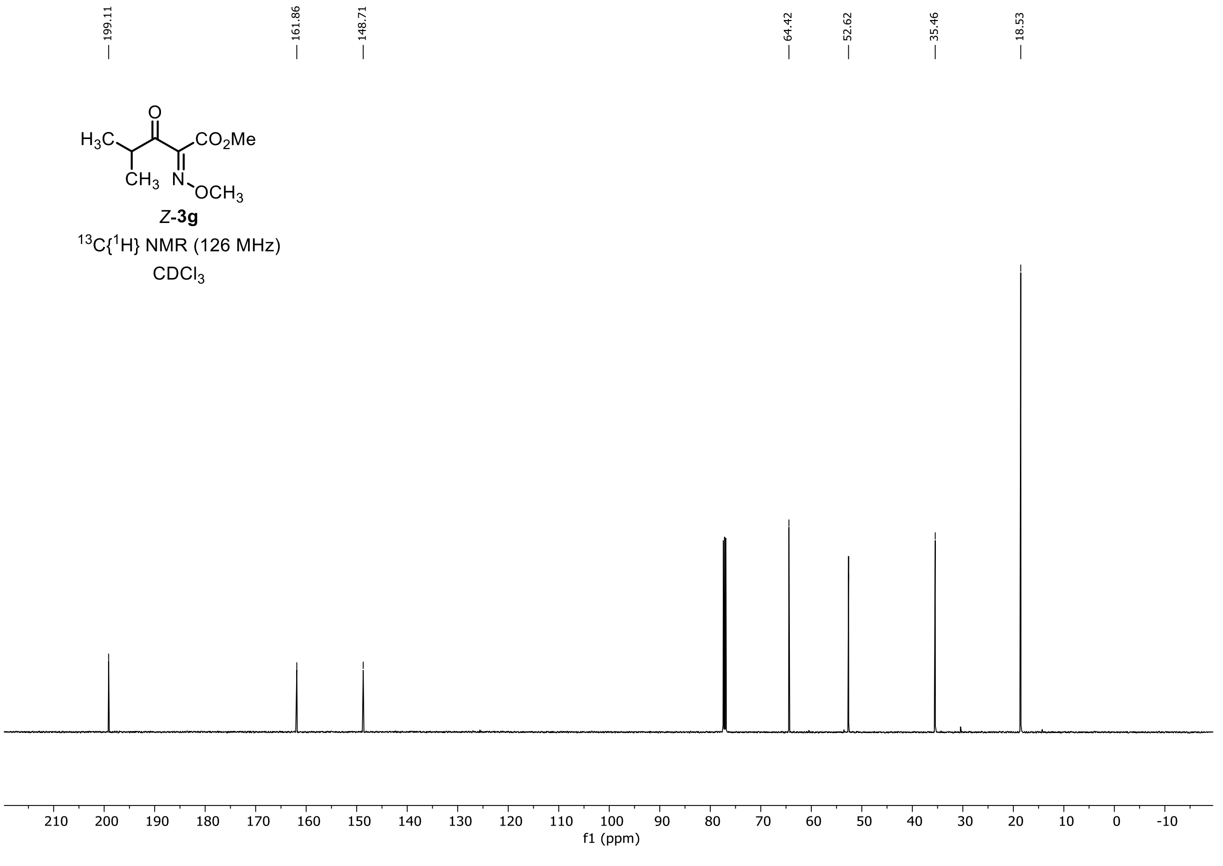

199.11
161.86
148.71
64.42
52.62
35.46
18.53
H3C
CO2Me
CH3
N
OCH3
Z-3g
13C{1H} NMR (126 MHz)
CDCl3
210
200
190
180
170
160
150
140
130
120
110
100
90
80
70
60
50
40
30
20
10
0
-10
f1 (ppm)

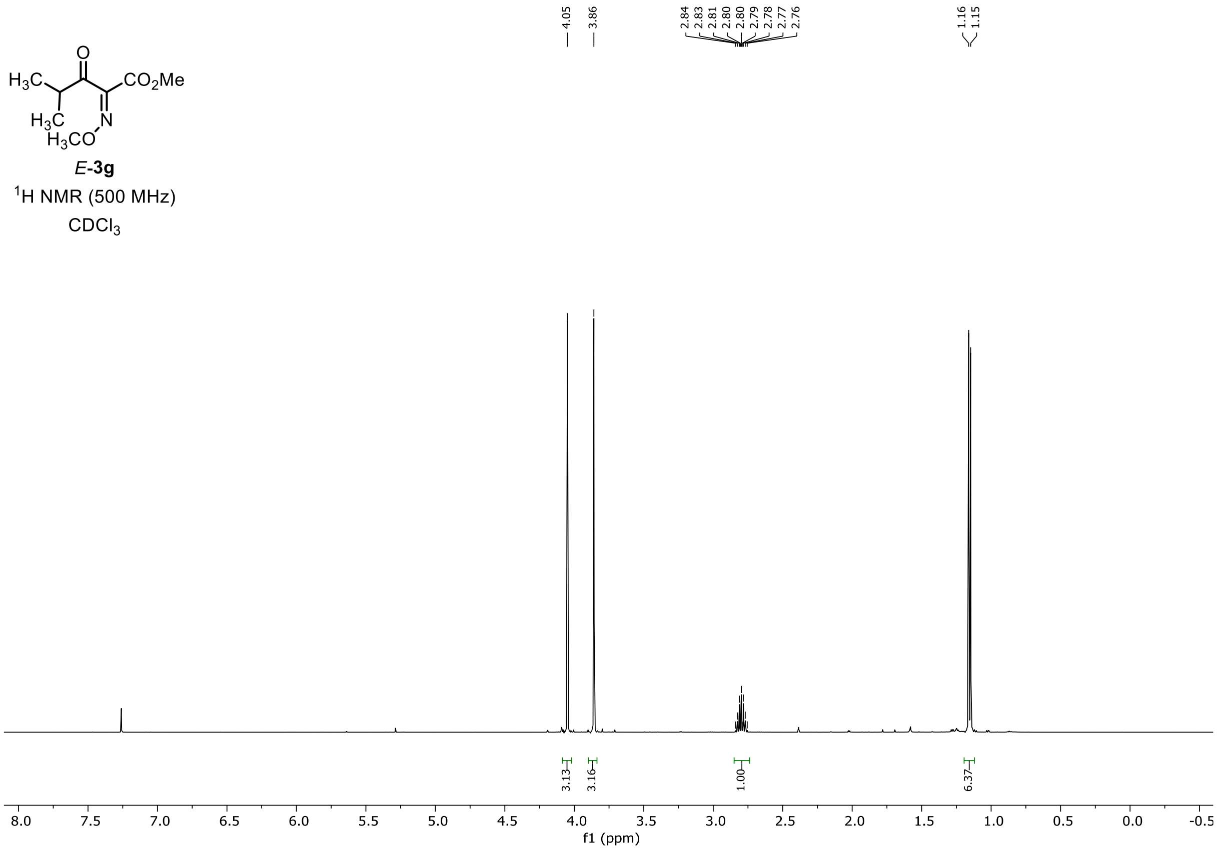
H3C
O
CO2Me
H3C
N
H3CO
E-3g
1H NMR (500 MHz)
CDCl3
4.05
3.86
2.84
2.83
2.81
2.80
2.80
2.79
2.78
2.77
2.76
1.16
1.15
3.13
3.16
1.00
6.37
8.0 7.5 7.0 6.5 6.0 5.5 5.0 4.5 4.0 3.5 3.0 2.5 2.0 1.5 1.0 0.5 0.0 -0.5
f1 (ppm)

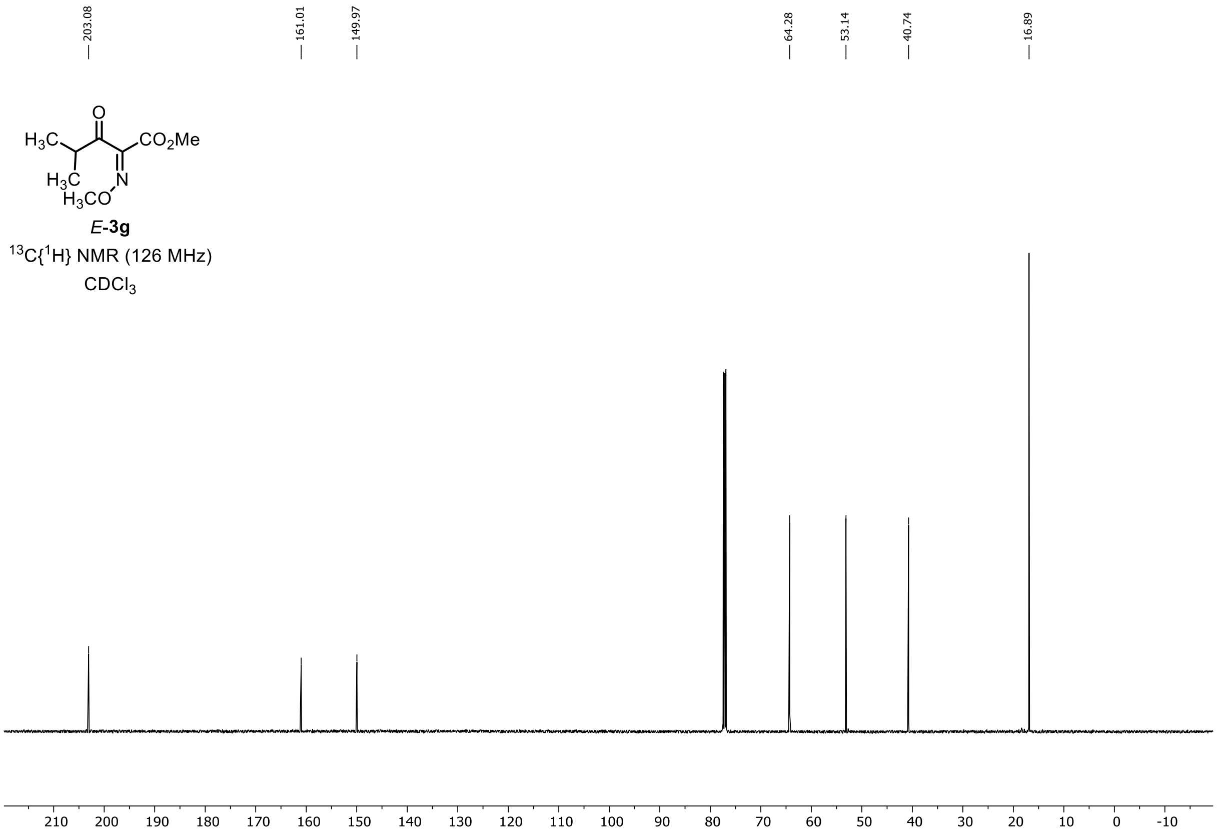
203.08
161.01
149.97
64.28
53.14
40.74
16.89
H3C
O
CO2Me
H3C
N
H3CO
E-3g
13C{1H} NMR (126 MHz)
CDCl3
210 200 190 180 170 160 150 140 130 120 110 100 90 80 70 60 50 40 30 20 10 0 -10
f1 (ppm)

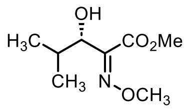

Z-**4g**

^{1}H NMR (300 MHz)

$CDCl_3$

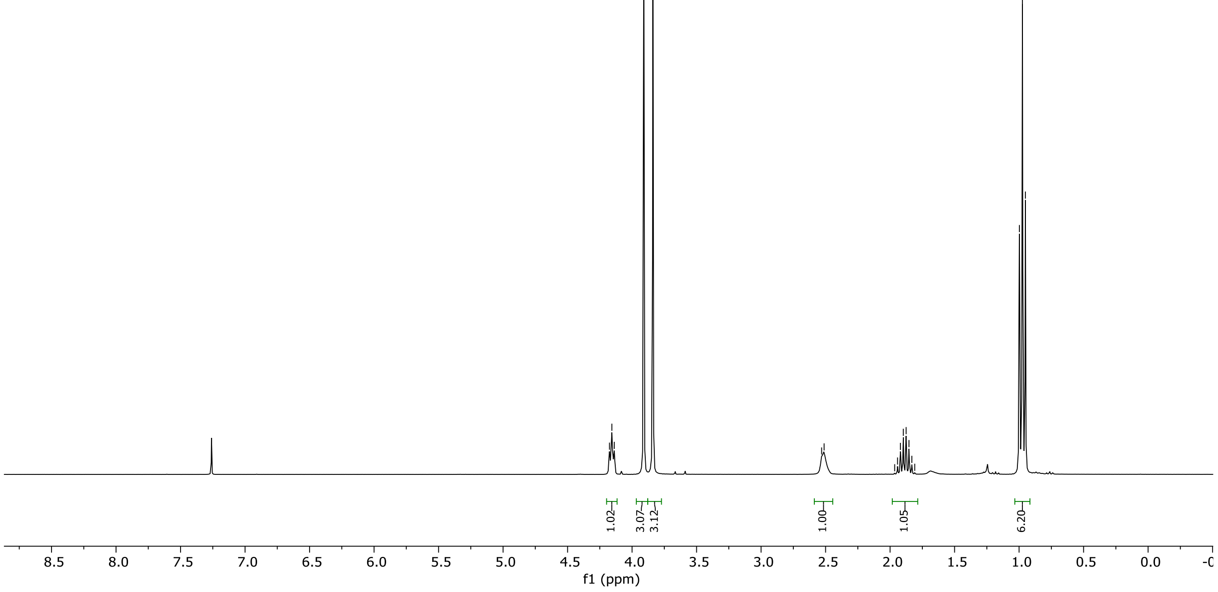

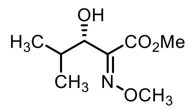

Z-**4g**

$^{13}C\{^{1}H\}$ NMR (126 MHz)

$CDCl_3$

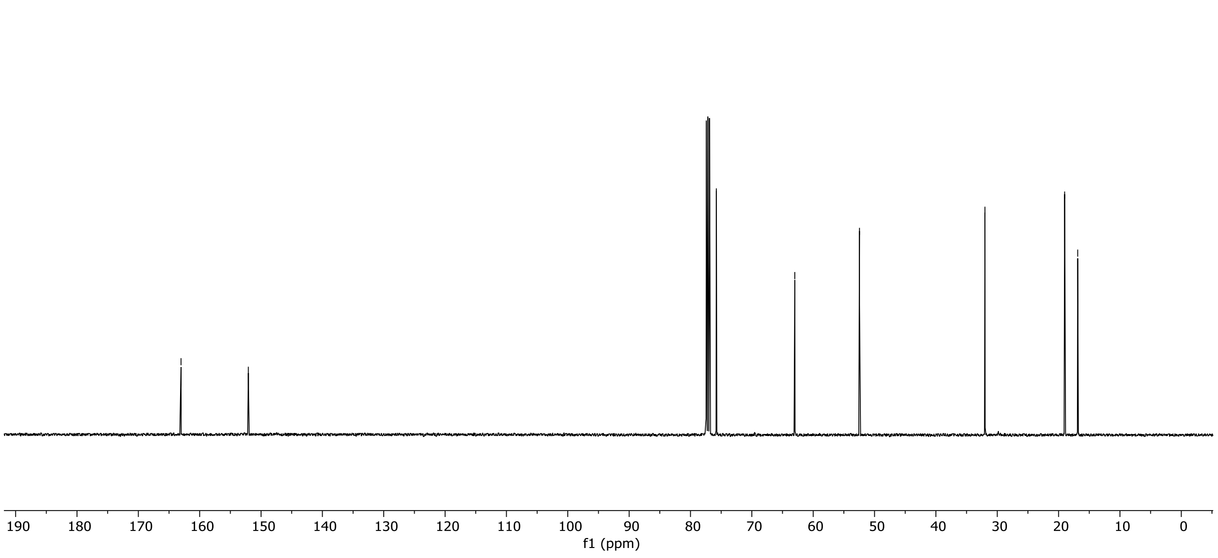

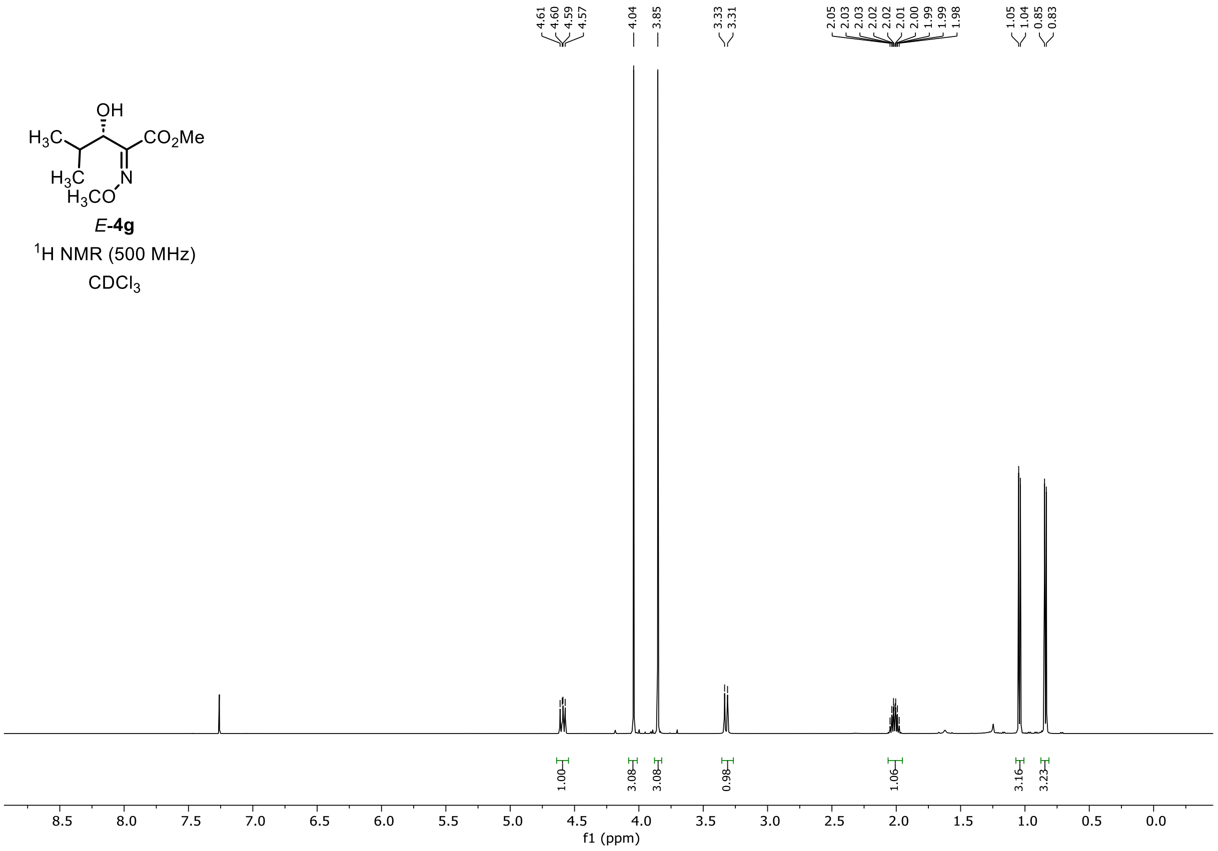
4.61 4.60 4.59 4.57
4.04
3.85
3.33 3.31
2.05 2.03 2.03 2.02 2.02 2.01 2.00 1.99 1.99 1.98
1.05 1.04 0.85 0.83
OH
H_3C
CO_2Me
H_3C
N
H_3CO
E-**4g**
^1H NMR (500 MHz)
$CDCl_3$
1.00
3.08
3.08
0.98
1.06
3.16
3.23
8.5 8.0 7.5 7.0 6.5 6.0 5.5 5.0 4.5 4.0 3.5 3.0 2.5 2.0 1.5 1.0 0.5 0.0
f1 (ppm)

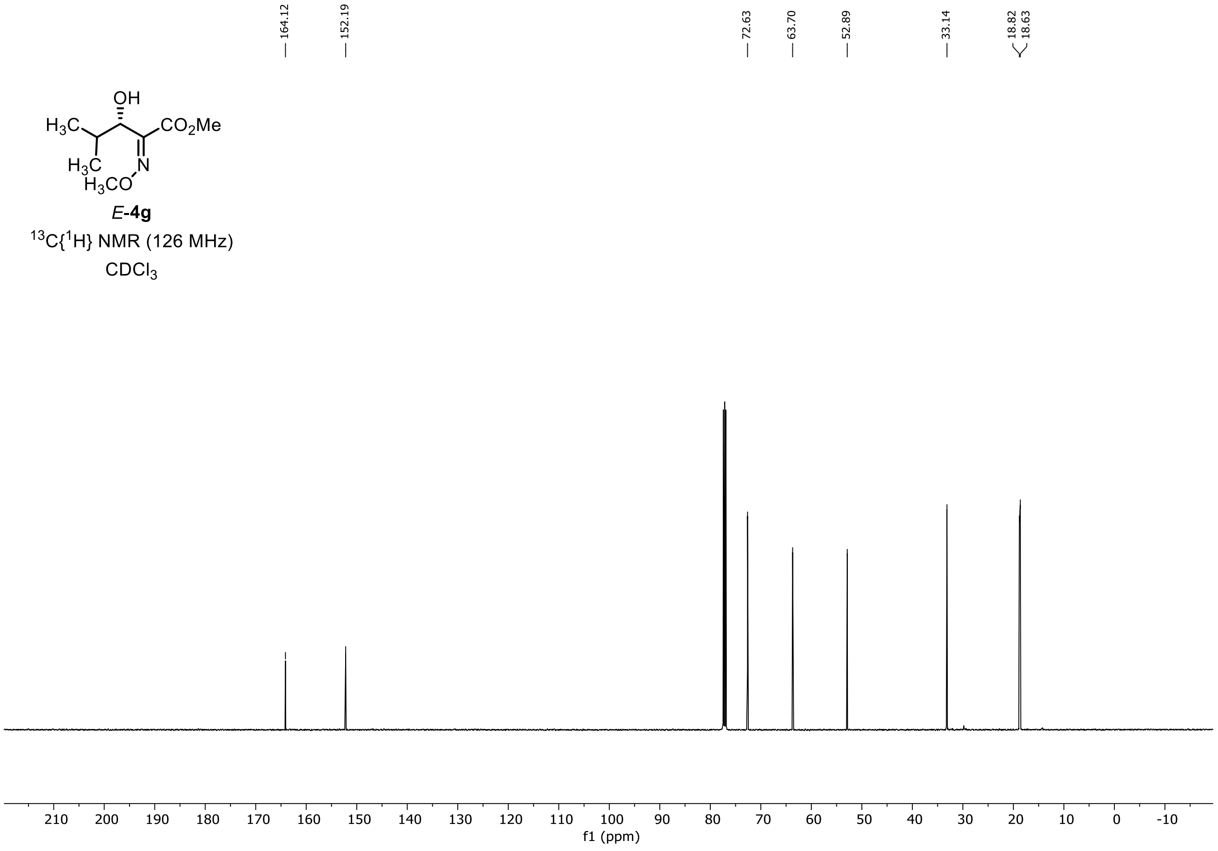
164.12
152.19
72.63
63.70
52.89
33.14
18.82 18.63
OH
H_3C
CO_2Me
H_3C
N
H_3CO
E-**4g**
$^{13}C\{^1H\}$ NMR (126 MHz)
$CDCl_3$
210 200 190 180 170 160 150 140 130 120 110 100 90 80 70 60 50 40 30 20 10 0 -10
f1 (ppm)

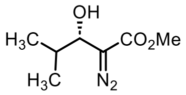

22 (ca. 85% purity)
^{1}H NMR (500 MHz)
$CDCl_3$

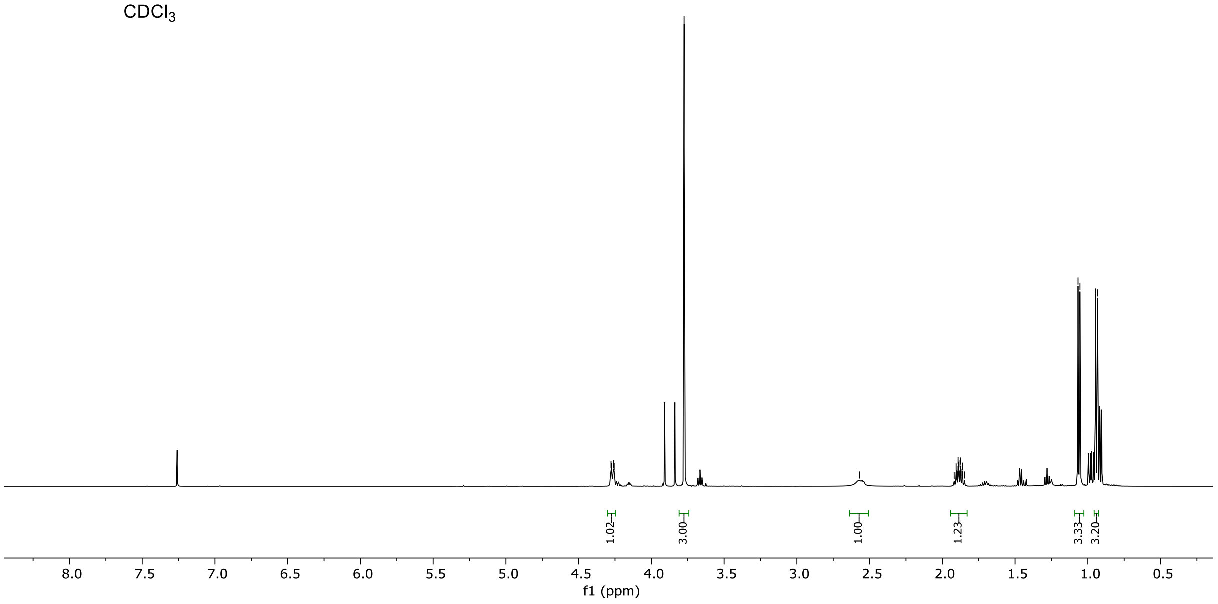

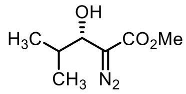

22 (ca. 85% purity)
$^{13}C\{^1H\}$ NMR (126 MHz)
$CDCl_3$

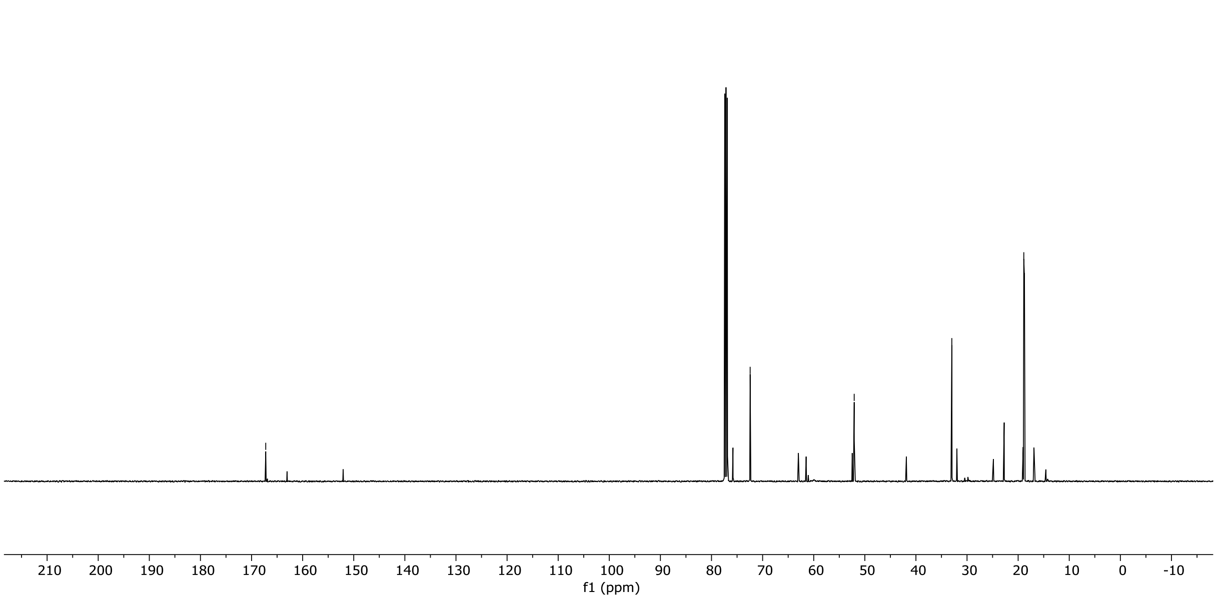

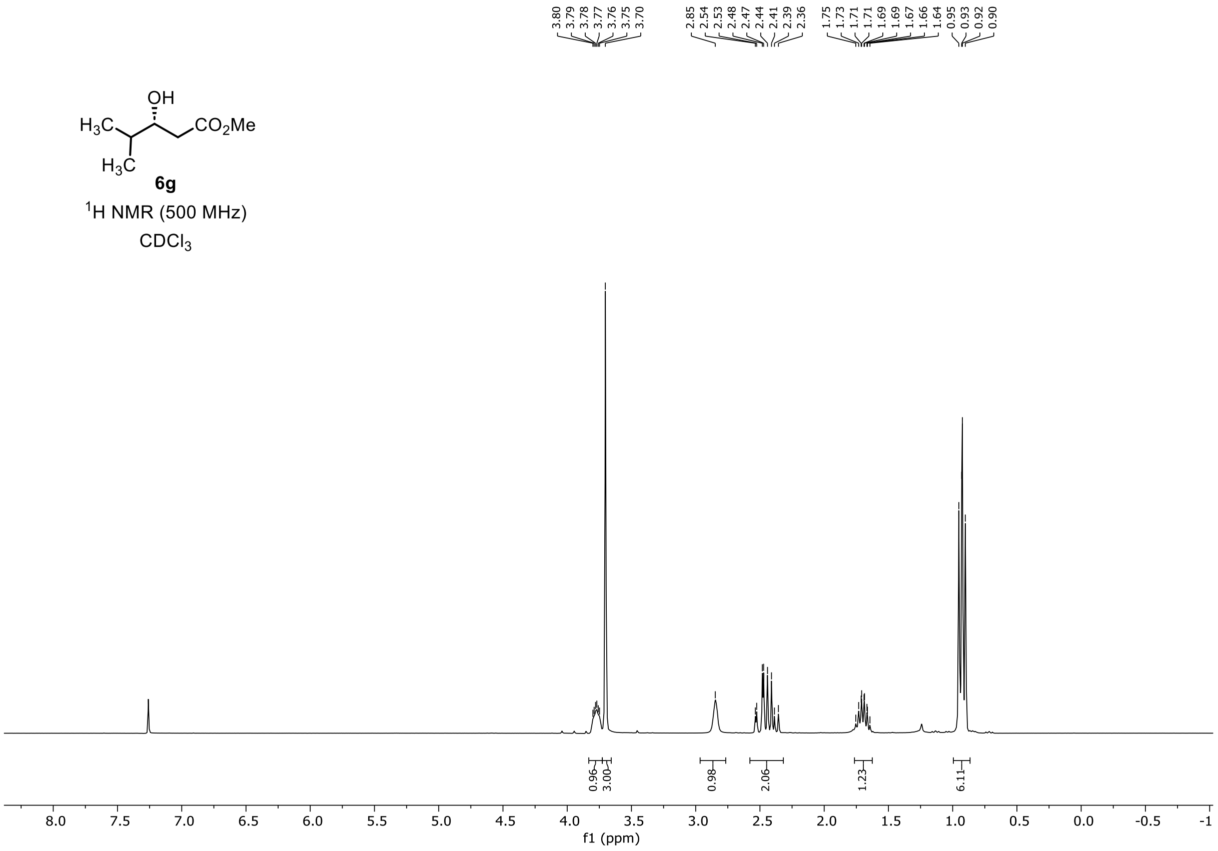
OH
H3C
CO2Me
H3C
6g
1H NMR (500 MHz)
CDCl3
3.80 3.79 3.78 3.77 3.76 3.75 3.70
2.85 2.54 2.53 2.48 2.47 2.44 2.41 2.39 2.36
1.75 1.73 1.71 1.71 1.69 1.69 1.67 1.66 1.64
0.95 0.93 0.92 0.90
0.96 3.00 0.98 2.06 1.23 6.11
f1 (ppm)

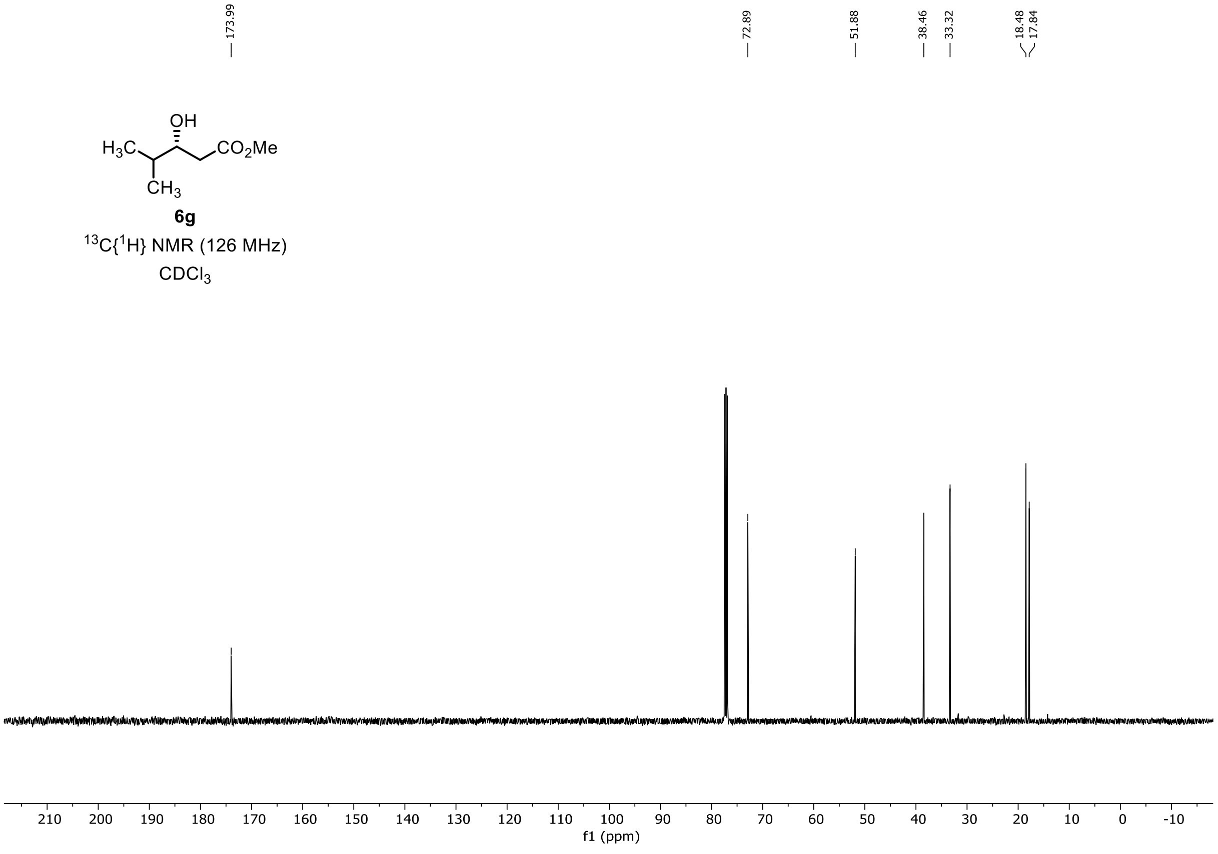
173.99
72.89
51.88
38.46
33.32
18.48
17.84
OH
H3C
CO2Me
CH3
6g
13C{1H} NMR (126 MHz)
CDCl3
f1 (ppm)

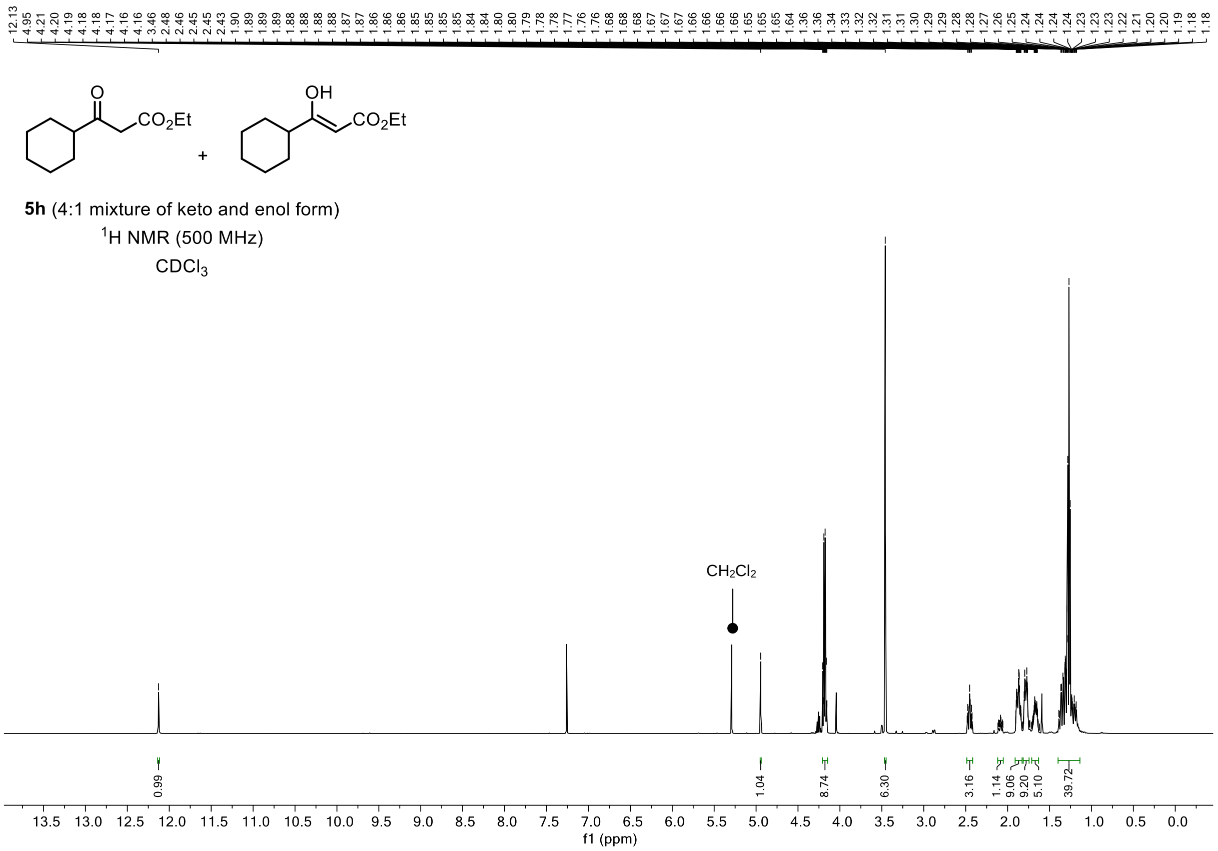
12.13 4.95 4.21 4.20 4.19 4.18 4.18 4.17 4.16 4.16 3.46 2.48 2.46 2.45 2.45 2.43 1.90 1.89 1.89 1.88 1.88 1.88 1.88 1.87 1.87 1.86 1.86 1.86 1.85 1.85 1.85 1.85 1.84 1.84 1.80 1.80 1.79 1.78 1.78 1.77 1.76 1.76 1.68 1.68 1.68 1.68 1.67 1.67 1.67 1.66 1.66 1.66 1.66 1.65 1.65 1.65 1.64 1.36 1.36 1.34 1.33 1.32 1.32 1.31 1.31 1.30 1.29 1.29 1.28 1.28 1.27 1.26 1.25 1.24 1.24 1.24 1.24 1.23 1.23 1.23 1.22 1.21 1.20 1.20 1.19 1.18 1.18
O
CO2Et
OH
CO2Et
+
5h (4:1 mixture of keto and enol form)
1H NMR (500 MHz)
CDCl3
CH2Cl2
0.99
1.04
8.74
6.30
3.16
1.14
9.06
9.20
5.10
39.72
13.5 13.0 12.5 12.0 11.5 11.0 10.5 10.0 9.5 9.0 8.5 8.0 7.5 7.0 6.5 6.0 5.5 5.0 4.5 4.0 3.5 3.0 2.5 2.0 1.5 1.0 0.5 0.0
f1 (ppm)

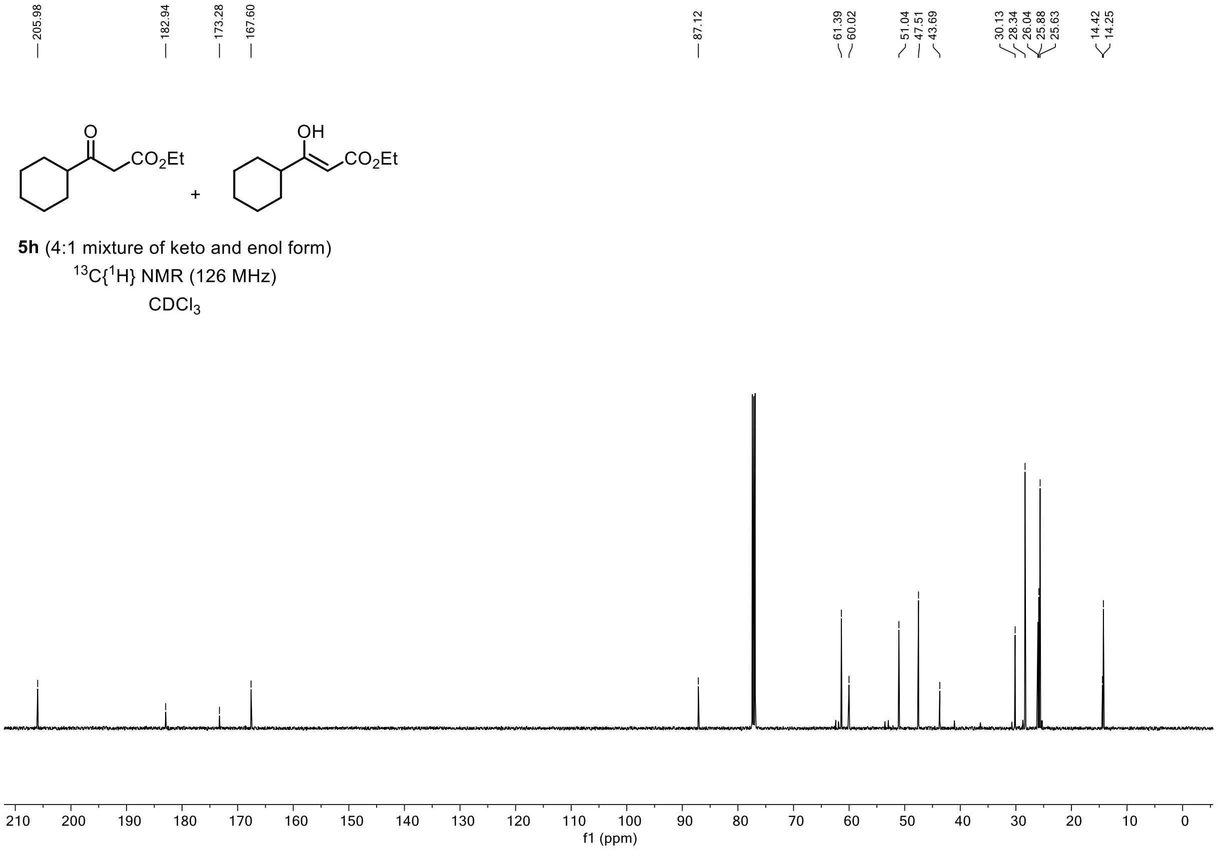
205.98
182.94
173.28
167.60
87.12
61.39
60.02
51.04
47.51
43.69
30.13
28.34
26.04
25.88
25.63
14.42
14.25
O
CO2Et
OH
CO2Et
+
5h (4:1 mixture of keto and enol form)
13C{1H} NMR (126 MHz)
CDCl3
210 200 190 180 170 160 150 140 130 120 110 100 90 80 70 60 50 40 30 20 10 0
f1 (ppm)

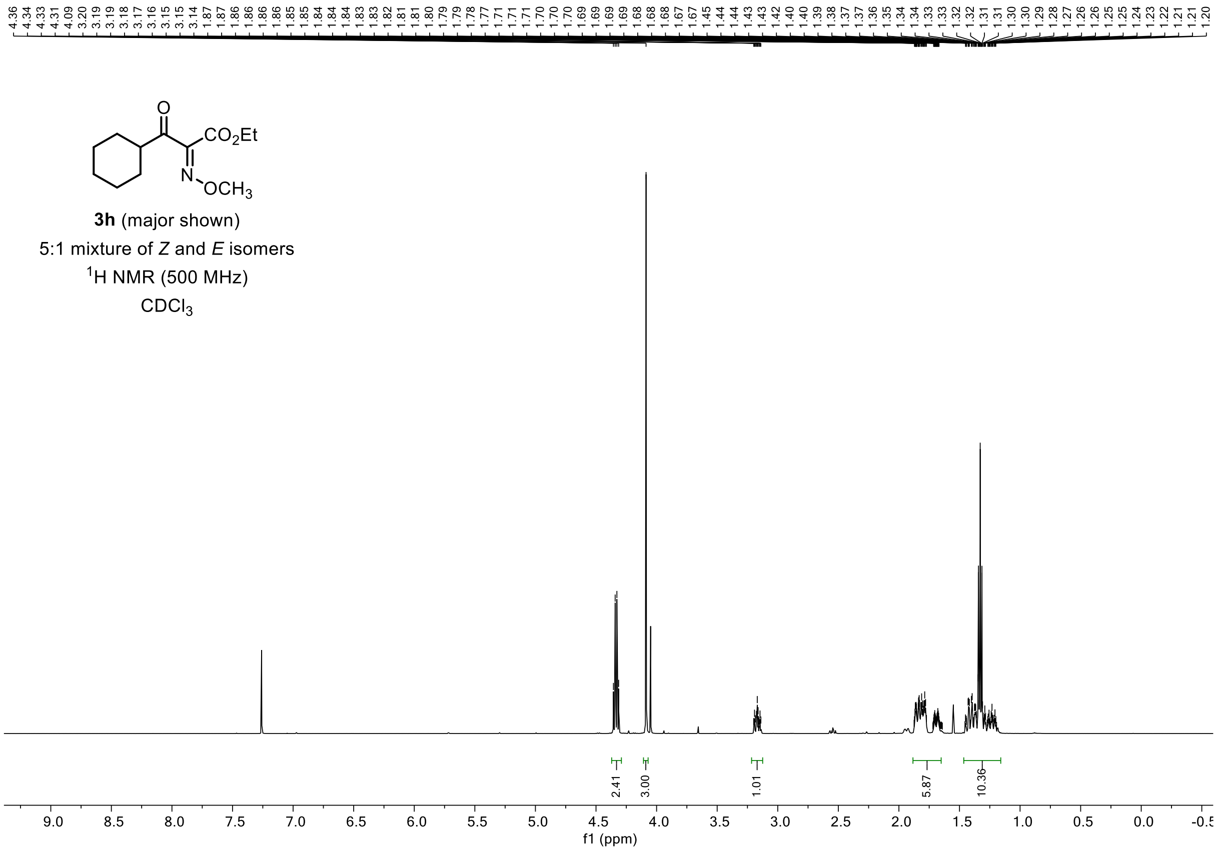

O
CO_2Et
N
OCH_3
3h (major shown)
5:1 mixture of *Z* and *E* isomers
^{1}H NMR (500 MHz)
$CDCl_3$
2.41
3.00
1.01
5.87
10.36
9.0 8.5 8.0 7.5 7.0 6.5 6.0 5.5 5.0 4.5 4.0 3.5 3.0 2.5 2.0 1.5 1.0 0.5 0.0
f1 (ppm)

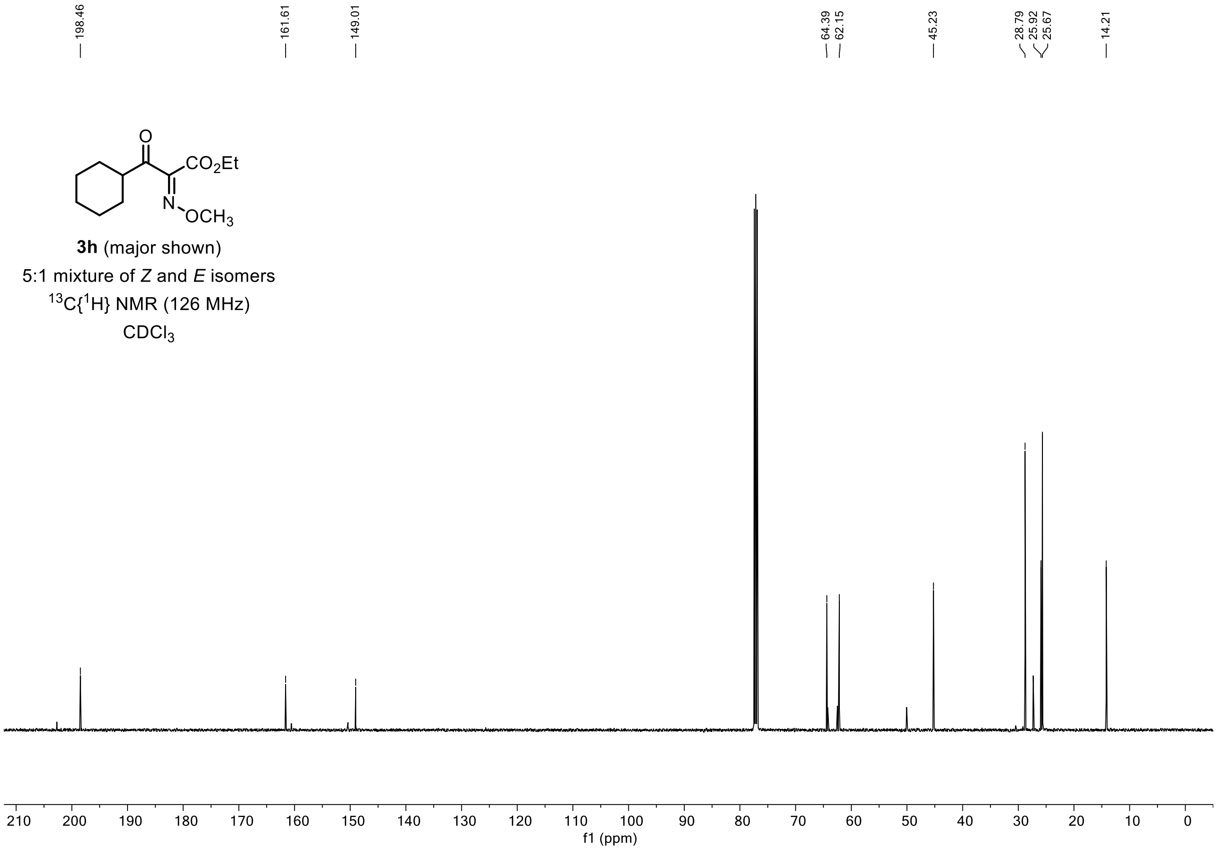

198.46
161.61
149.01
64.39
62.15
45.23
28.79
25.92
25.67
14.21
O
CO_2Et
N
OCH_3
3h (major shown)
5:1 mixture of *Z* and *E* isomers
^{13}C{^{1}H} NMR (126 MHz)
$CDCl_3$
210 200 190 180 170 160 150 140 130 120 110 100 90 80 70 60 50 40 30 20 10 0
f1 (ppm)

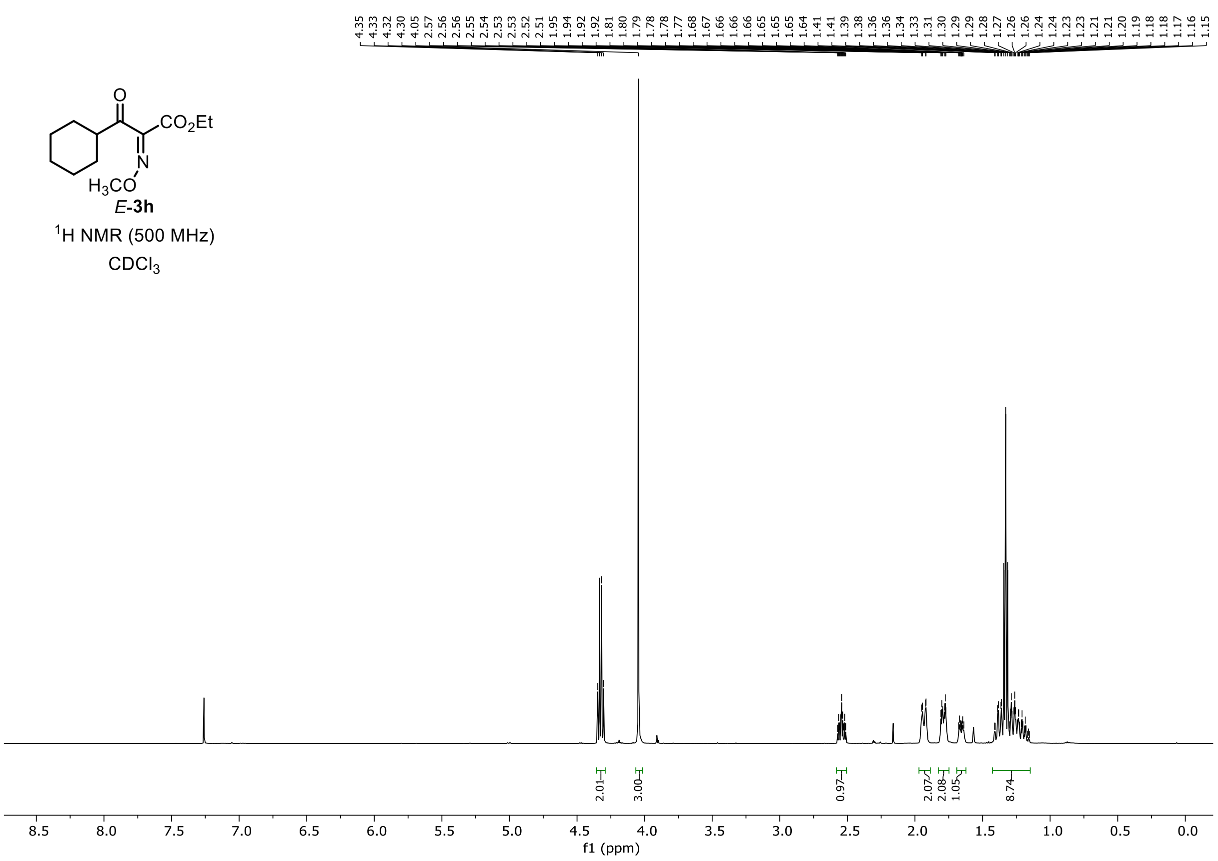
4.35 4.33 4.32 4.30 4.05 2.57 2.56 2.56 2.55 2.54 2.53 2.53 2.52 2.51 1.95 1.94 1.92 1.92 1.81 1.80 1.79 1.78 1.78 1.77 1.68 1.67 1.66 1.66 1.66 1.65 1.65 1.65 1.64 1.41 1.41 1.39 1.38 1.36 1.36 1.34 1.33 1.31 1.30 1.29 1.29 1.28 1.27 1.26 1.26 1.24 1.24 1.23 1.23 1.21 1.21 1.20 1.19 1.18 1.17 1.16 1.15
O
CO_2Et
N
H_3CO
E-**3h**
^1H NMR (500 MHz)
$CDCl_3$
2.01
3.00
0.97
2.07
2.08
1.05
8.74
f1 (ppm)

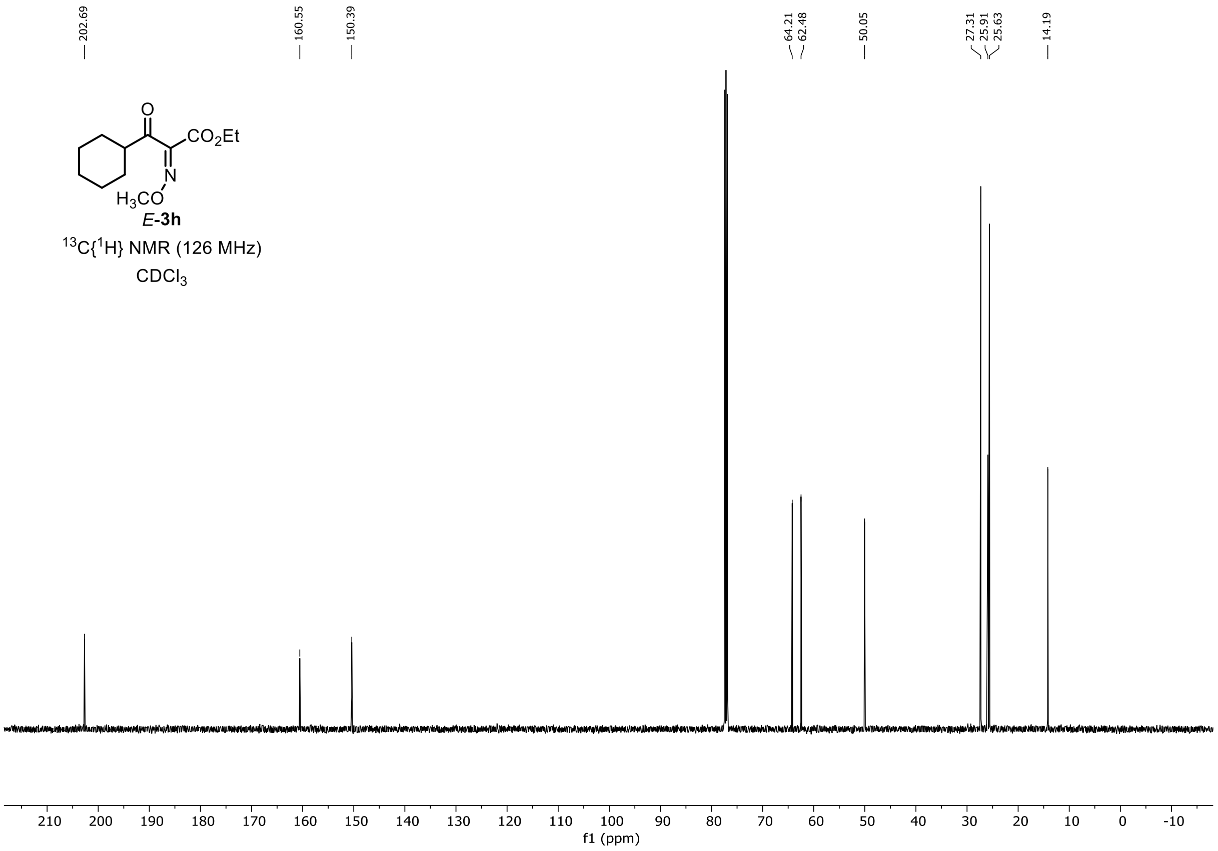
202.69
160.55
150.39
64.21
62.48
50.05
27.31
25.91
25.63
14.19
O
CO_2Et
N
H_3CO
E-**3h**
$^{13}C\{^1H\}$ NMR (126 MHz)
$CDCl_3$
f1 (ppm)

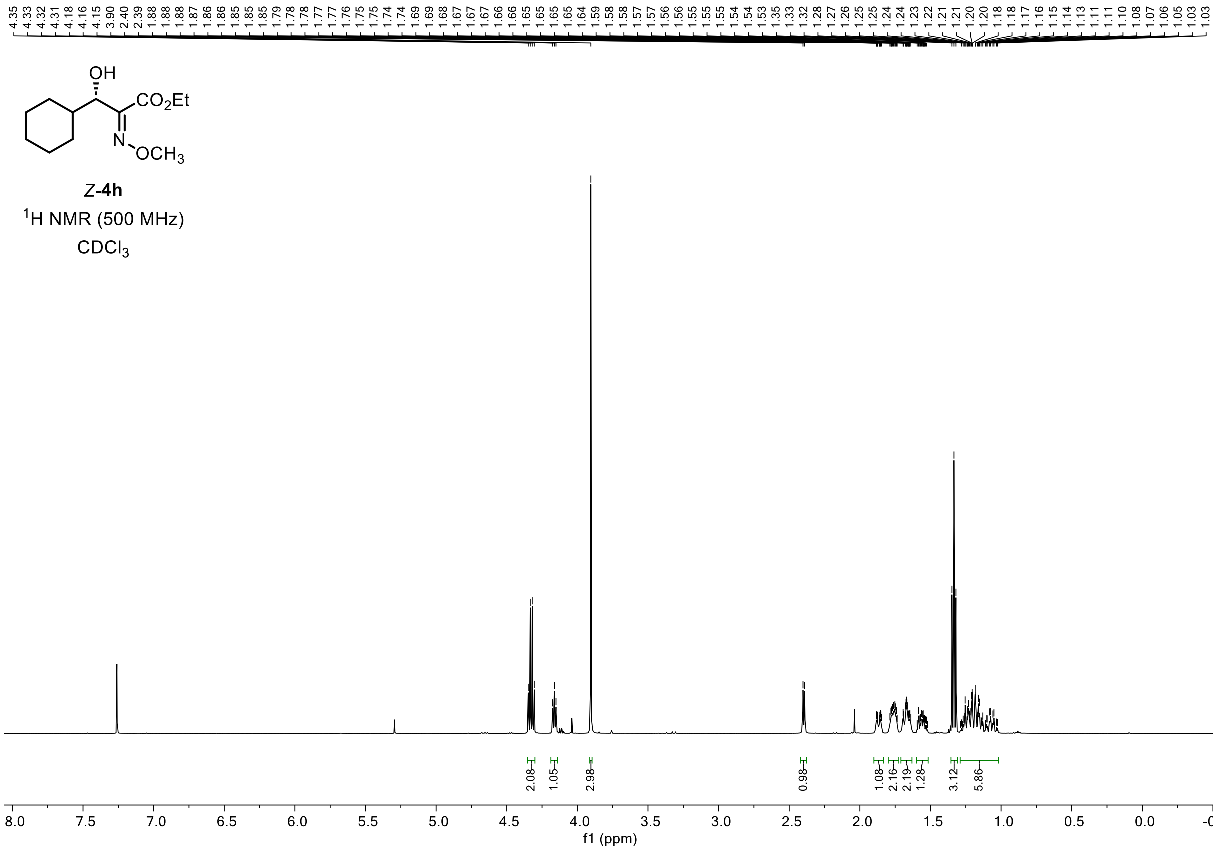
4.35 4.33 4.32 4.31 4.18 4.16 4.15 3.90 2.40 2.39 1.88 1.88 1.88 1.87 1.86 1.86 1.85 1.85 1.85 1.79 1.78 1.78 1.77 1.77 1.76 1.75 1.75 1.74 1.74 1.69 1.68 1.67 1.67 1.66 1.66 1.65 1.65 1.65 1.65 1.64 1.59 1.58 1.58 1.57 1.57 1.56 1.56 1.55 1.55 1.55 1.54 1.54 1.53 1.35 1.33 1.33 1.32 1.28 1.27 1.26 1.25 1.25 1.24 1.24 1.23 1.22 1.21 1.21 1.20 1.20 1.18 1.18 1.17 1.16 1.15 1.14 1.13 1.11 1.11 1.10 1.08 1.07 1.06 1.05 1.03 1.03
OH
CO2Et
N
OCH3
Z-4h
1H NMR (500 MHz)
CDCl3
2.08
1.05
2.98
0.98
1.08
2.16
2.19
1.28
3.12
5.86
f1 (ppm)

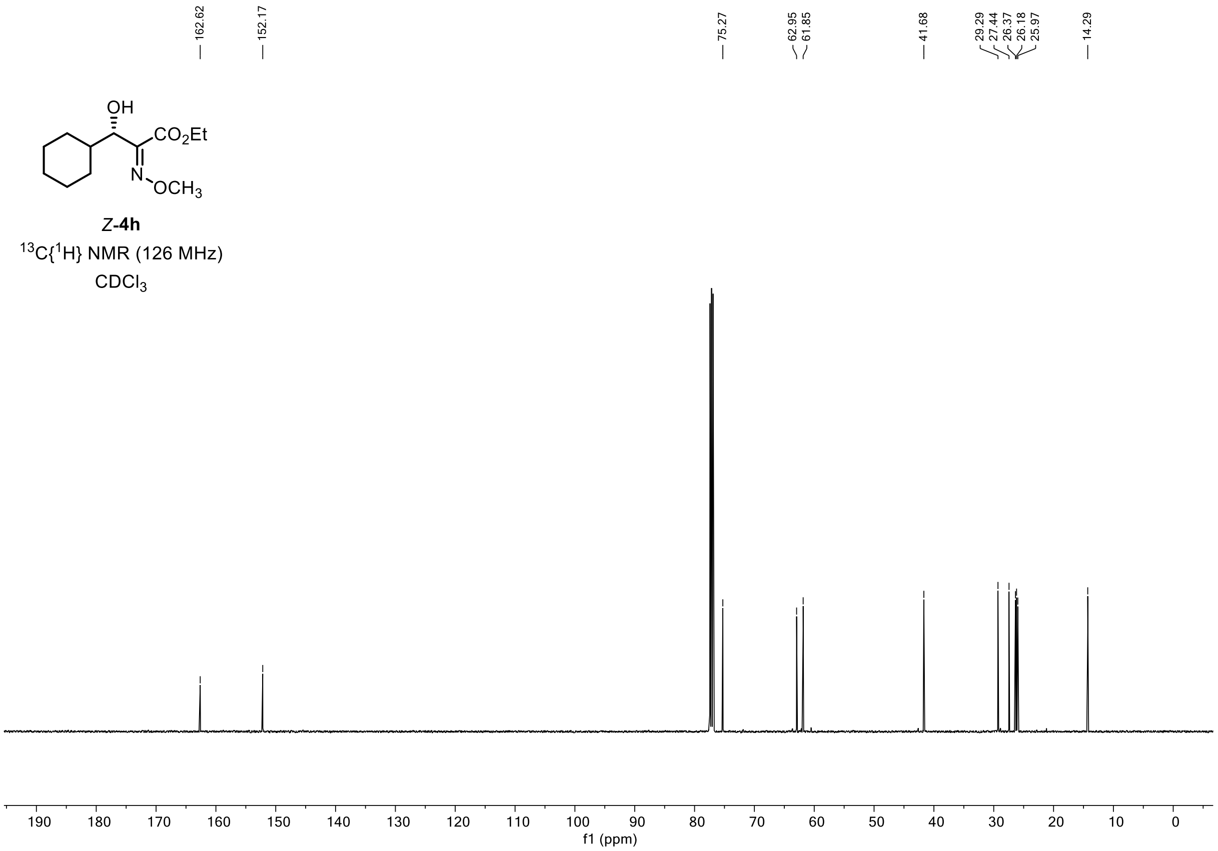
162.62
152.17
75.27
62.95
61.85
41.68
29.29
27.44
26.37
26.18
25.97
14.29
OH
CO2Et
N
OCH3
Z-4h
13C{1H} NMR (126 MHz)
CDCl3
f1 (ppm)

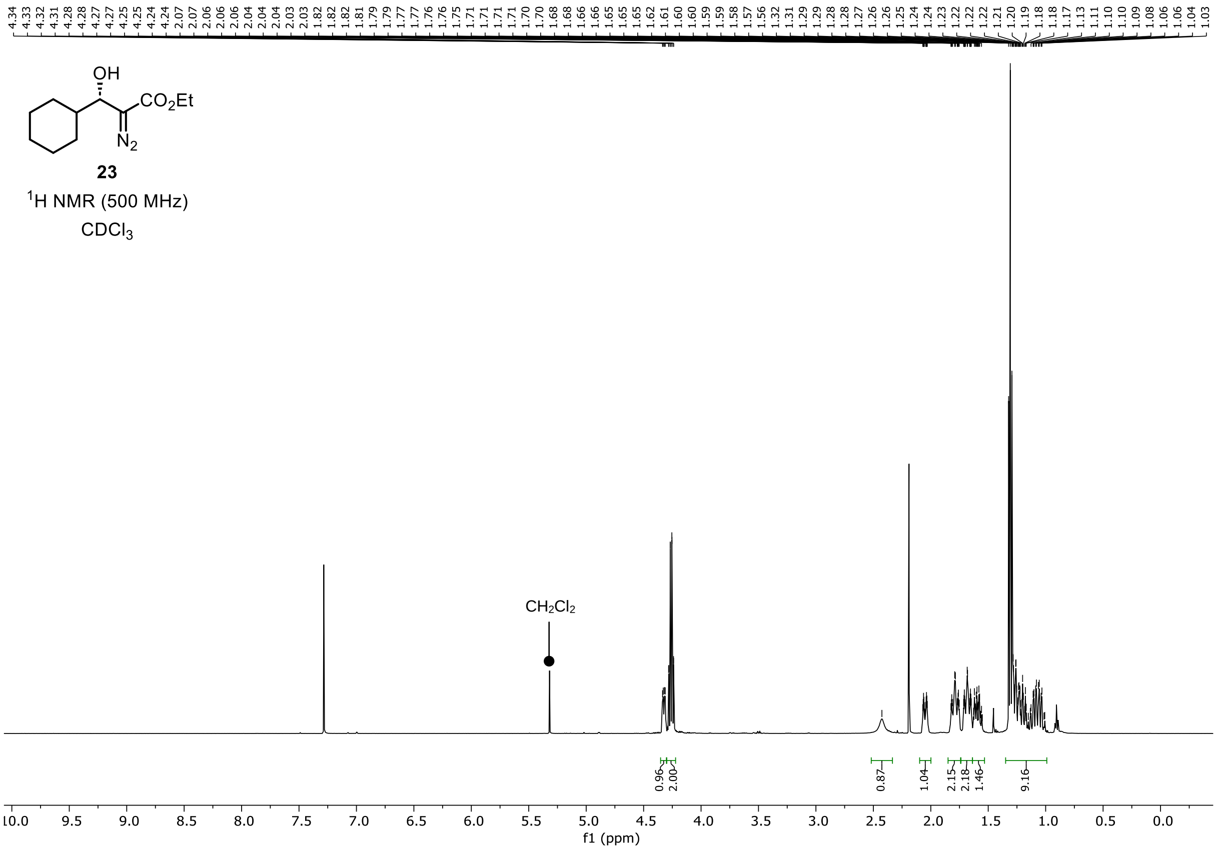
OH
CO_2Et
N_2
23
^{1}H NMR (500 MHz)
$CDCl_3$
CH_2Cl_2
0.96
2.00
0.87
1.04
2.15
2.18
1.46
9.16
f1 (ppm)

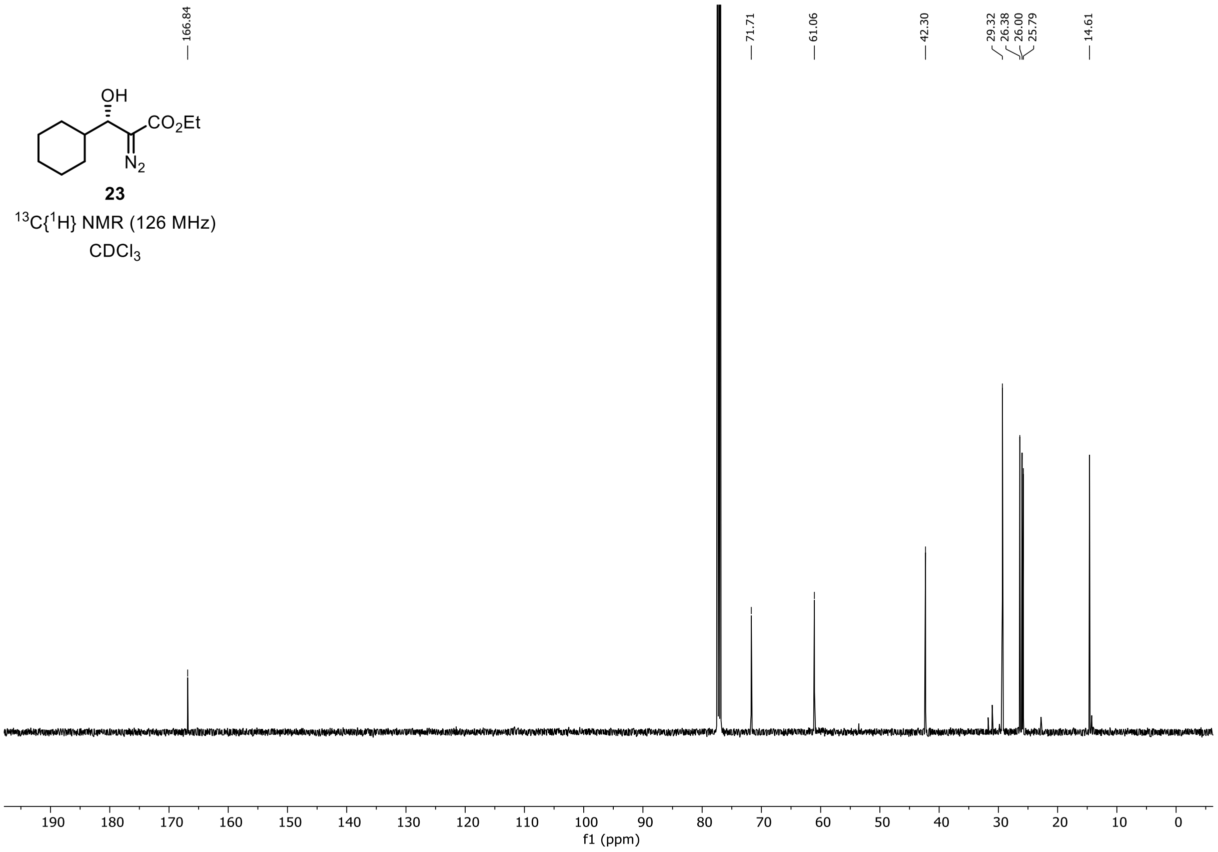
166.84
71.71
61.06
42.30
29.32
26.38
26.00
25.79
14.61
OH
CO_2Et
N_2
23
^{13}C{^{1}H} NMR (126 MHz)
$CDCl_3$
f1 (ppm)

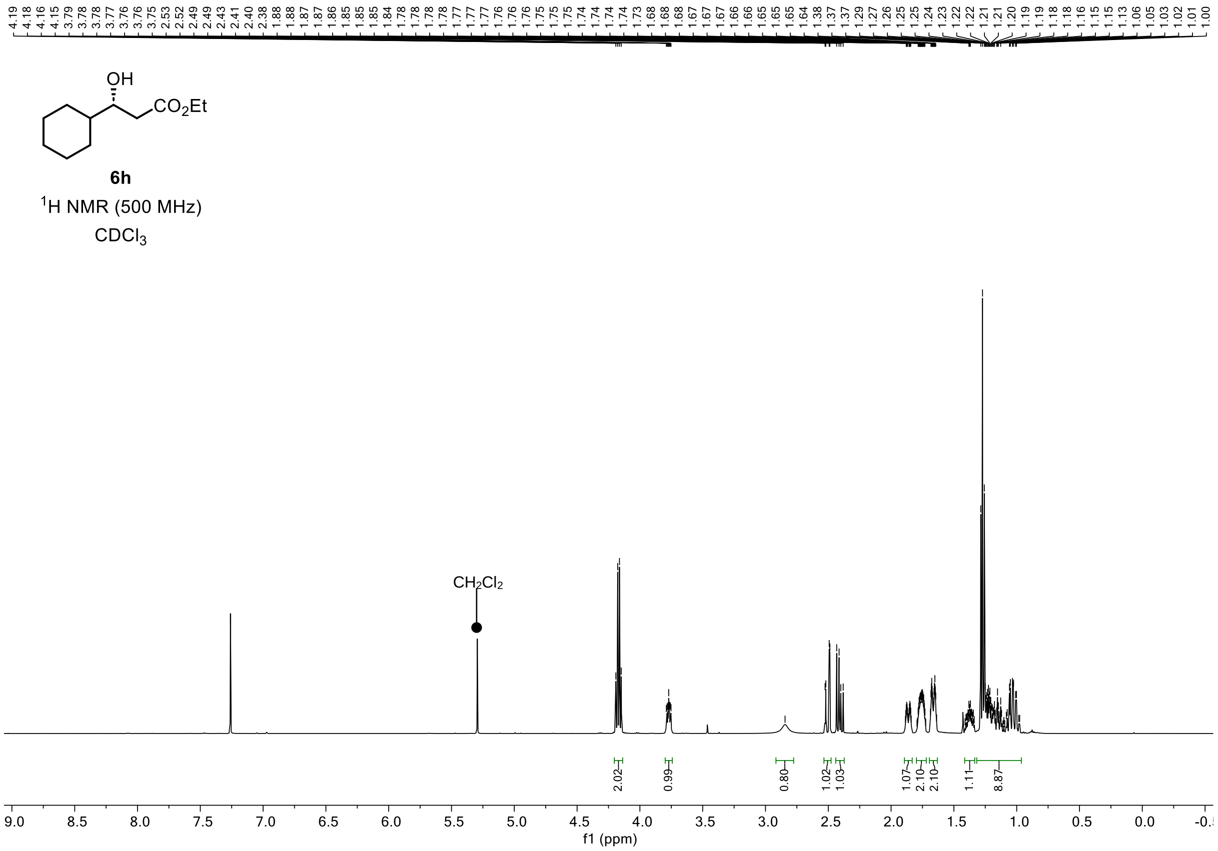
4.19 4.18 4.16 4.15 3.79 3.78 3.77 3.76 3.76 3.75 2.53 2.52 2.49 2.49 2.43 2.41 2.40 2.38 1.88 1.88 1.87 1.87 1.86 1.85 1.85 1.85 1.84 1.78 1.78 1.78 1.78 1.77 1.77 1.77 1.76 1.76 1.75 1.75 1.75 1.74 1.74 1.74 1.73 1.68 1.68 1.67 1.67 1.67 1.66 1.66 1.65 1.65 1.65 1.64 1.38 1.37 1.37 1.29 1.27 1.26 1.25 1.25 1.24 1.23 1.22 1.22 1.21 1.21 1.20 1.19 1.19 1.18 1.16 1.15 1.15 1.13 1.06 1.03 1.02 1.01 1.00
OH
CO2Et
6h
1H NMR (500 MHz)
CDCl3
CH2Cl2
2.02 0.99 0.80 1.02 1.03 1.07 2.10 2.10 1.11 8.87
9.0 8.5 8.0 7.5 7.0 6.5 6.0 5.5 5.0 4.5 4.0 3.5 3.0 2.5 2.0 1.5 1.0 0.5 0.0
f1 (ppm)

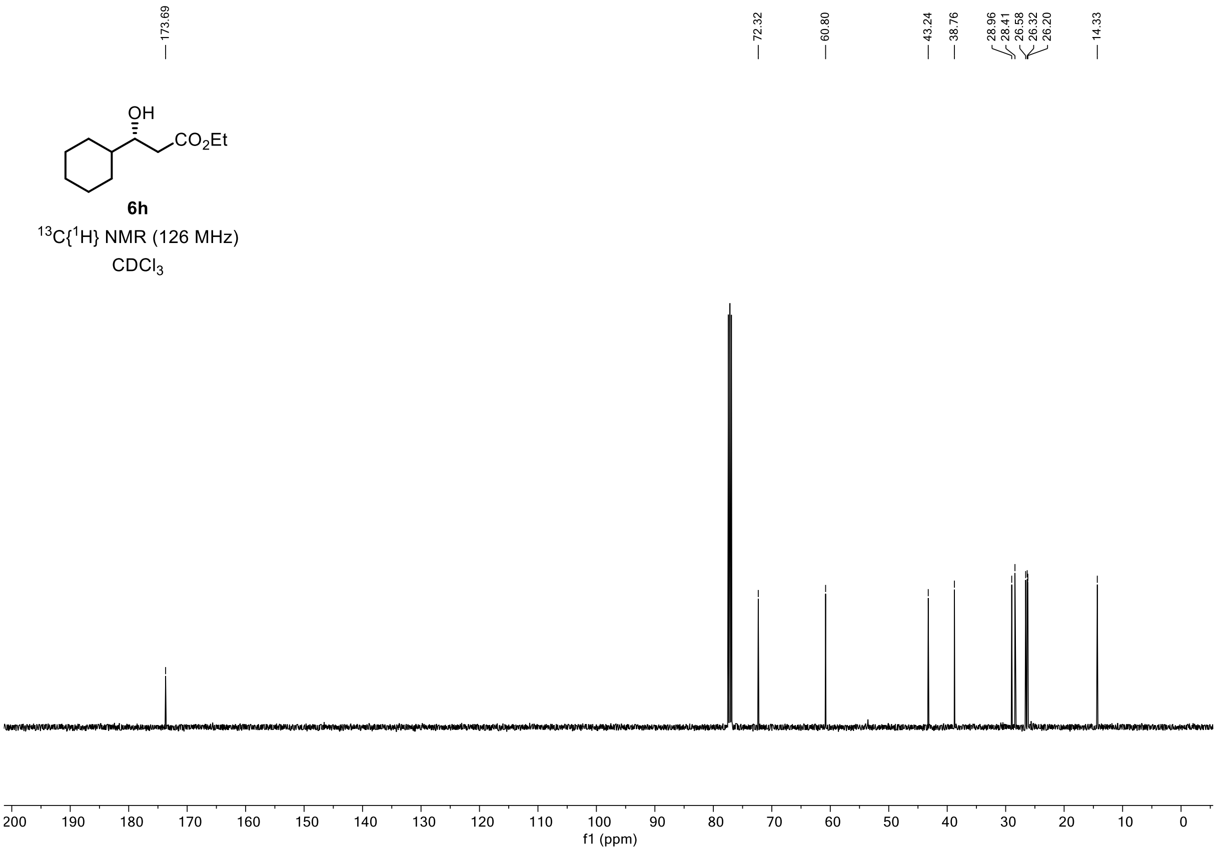
173.69 72.32 60.80 43.24 38.76 28.96 28.41 26.58 26.32 26.20 14.33
OH
CO2Et
6h
13C{1H} NMR (126 MHz)
CDCl3
200 190 180 170 160 150 140 130 120 110 100 90 80 70 60 50 40 30 20 10 0
f1 (ppm)

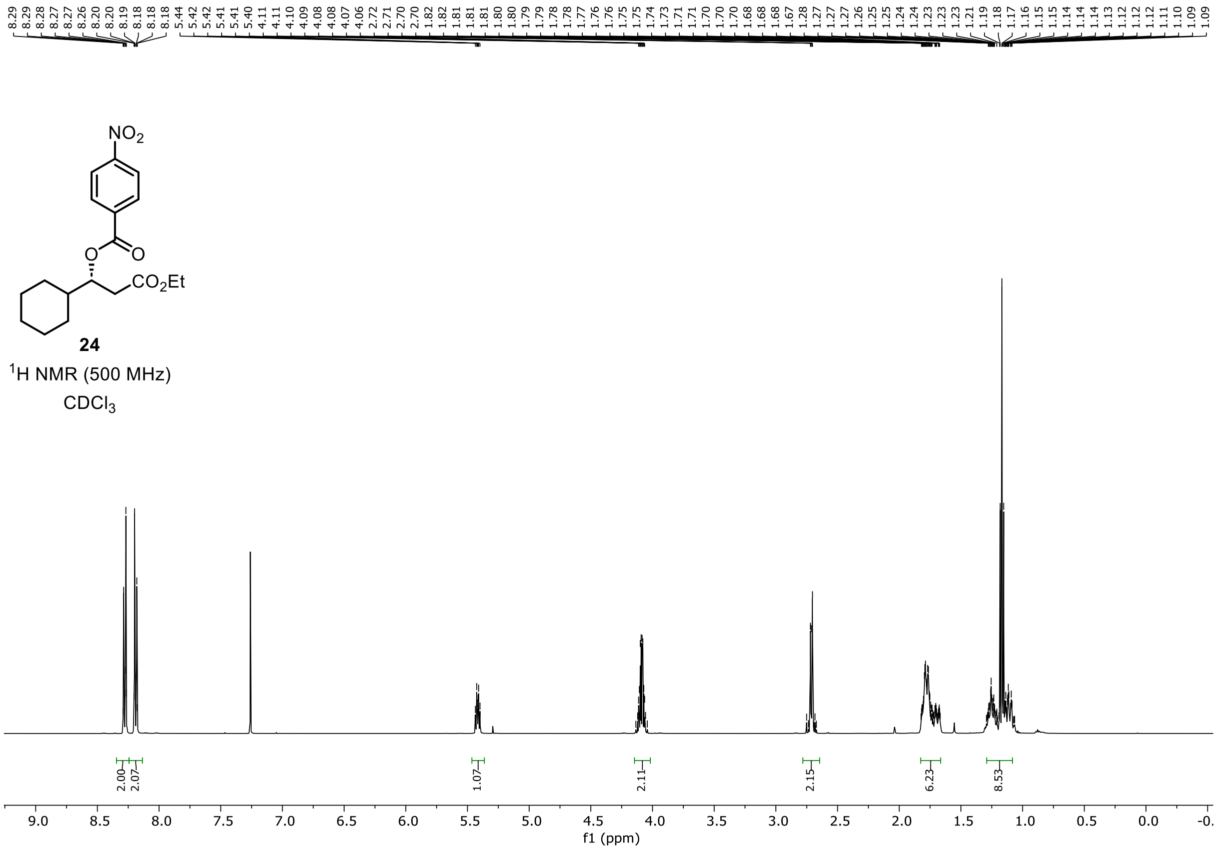
NO2
O
O
CO2Et
24
1H NMR (500 MHz)
CDCl3
f1 (ppm)

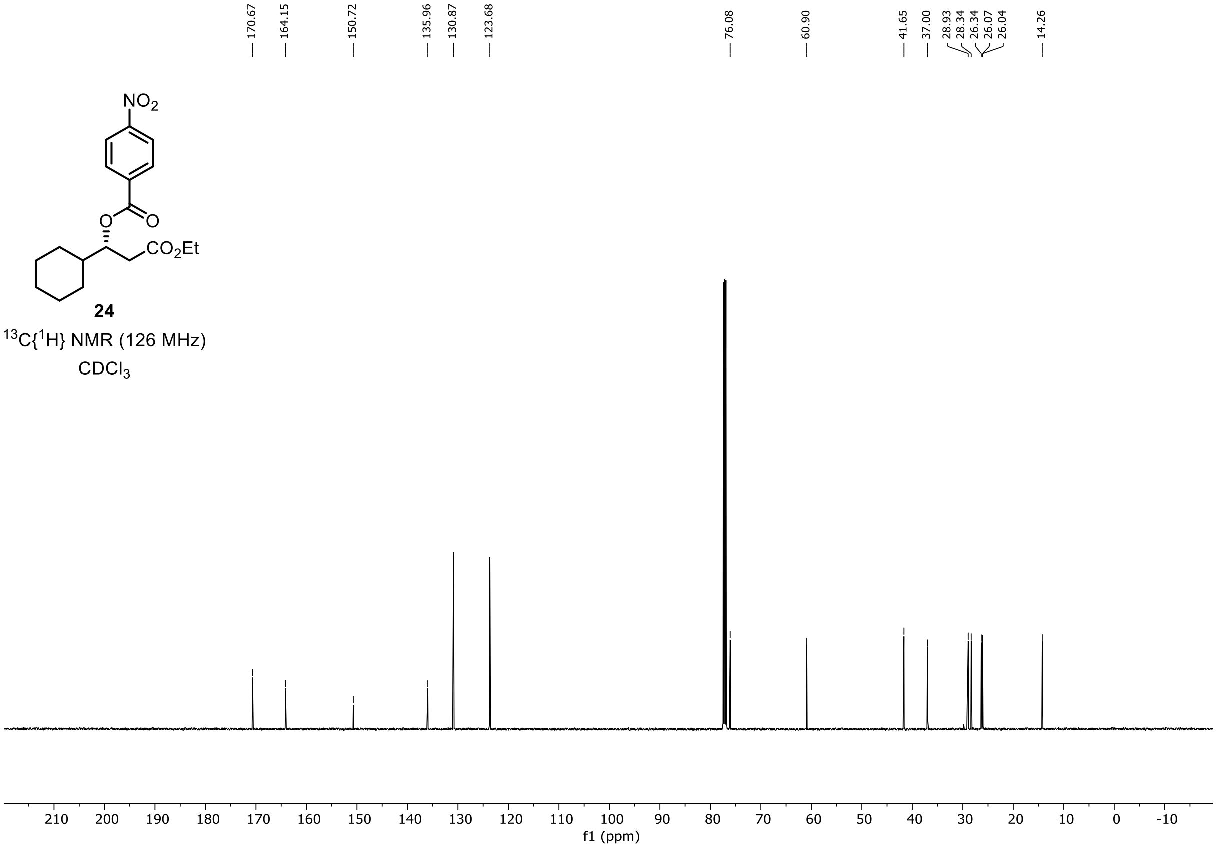
170.67
164.15
150.72
135.96
130.87
123.68
76.08
60.90
41.65
37.00
28.93
28.34
26.34
26.07
26.04
14.26
NO2
O
O
CO2Et
24
13C{1H} NMR (126 MHz)
CDCl3
f1 (ppm)

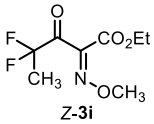

Z-**3i**

^{1}H NMR (500 MHz)

$CDCl_3$

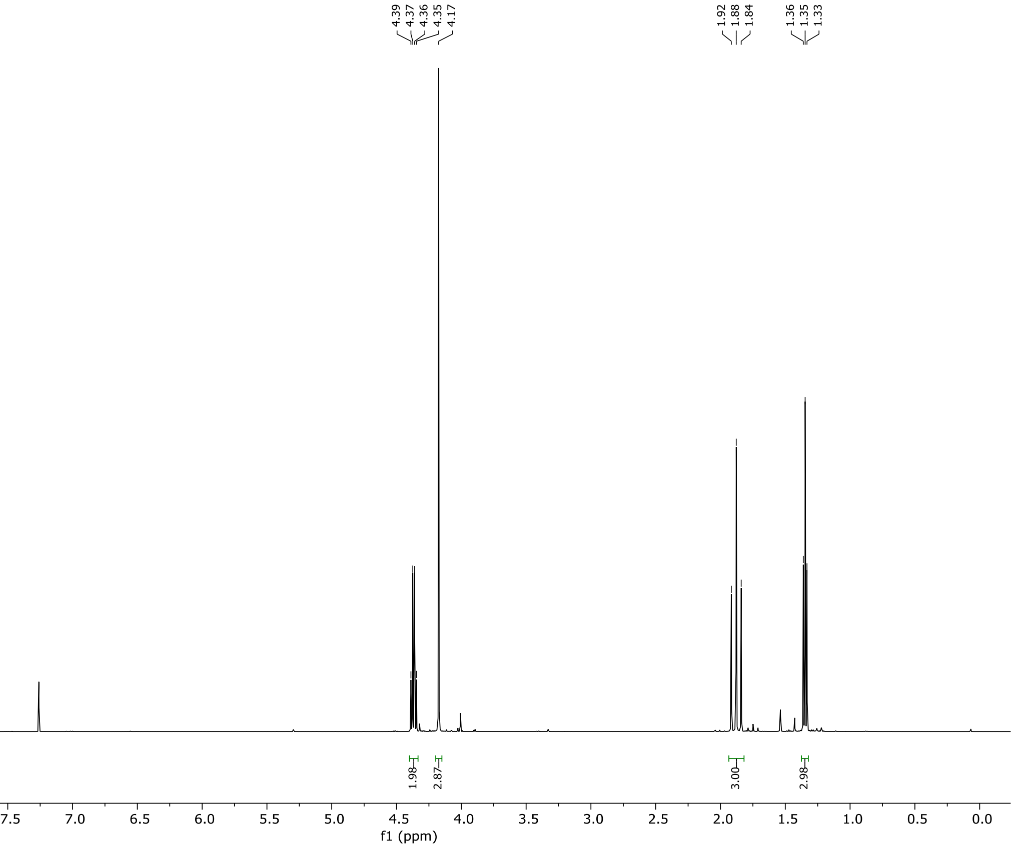

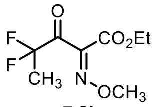

Z-**3i**

$^{13}C\{^1H\}$ NMR (126 MHz)

$CDCl_3$

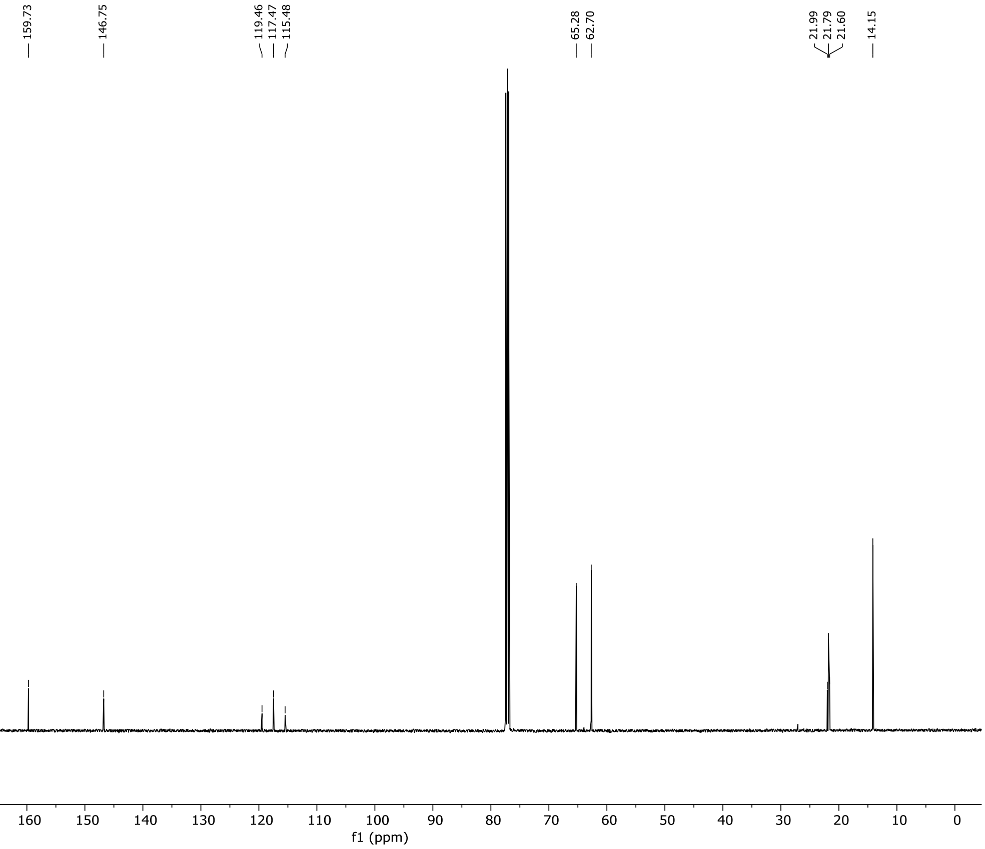

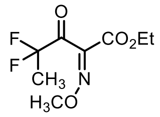

E-**3i**

^{1}H NMR (500 MHz)

$CDCl_3$

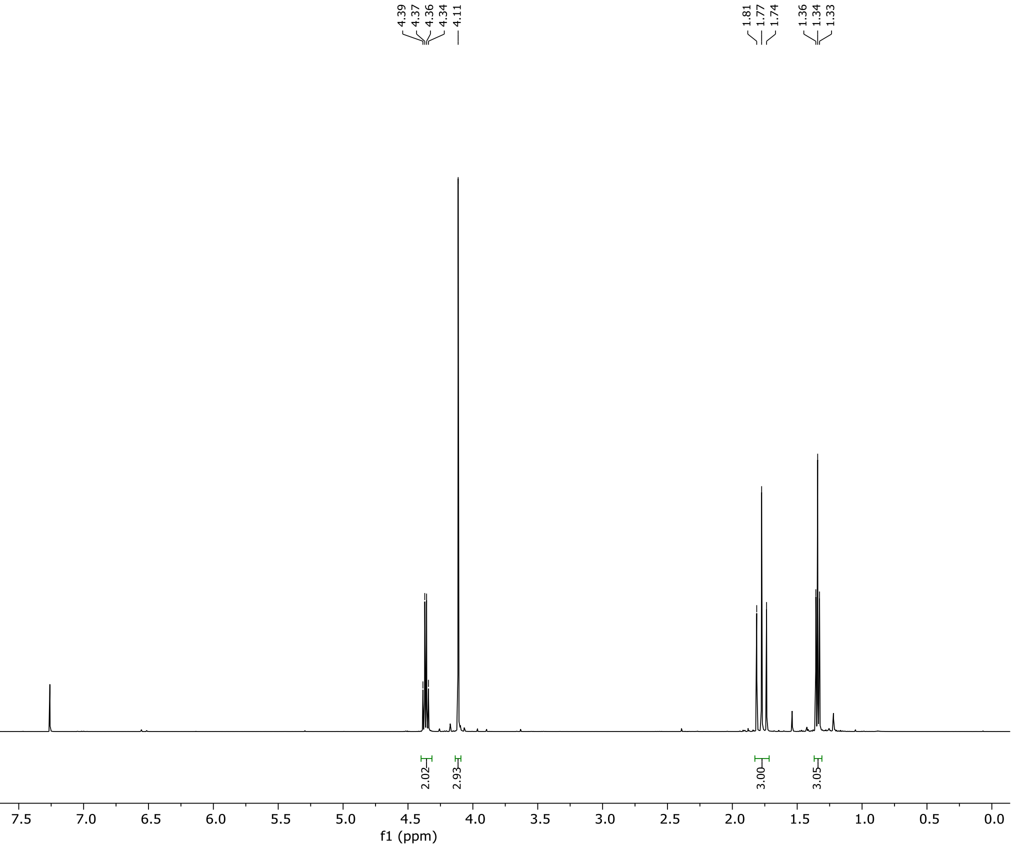

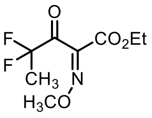

E-**3i**

$^{13}C\{^{1}H\}$ NMR (126 MHz)

$CDCl_3$

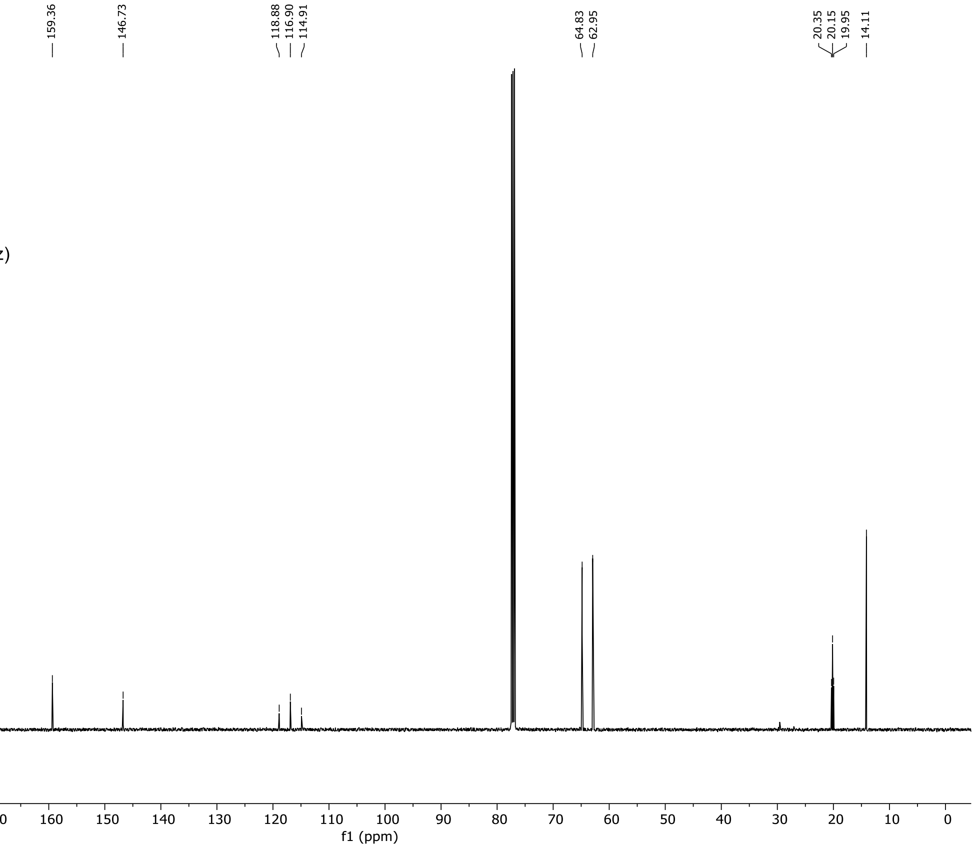

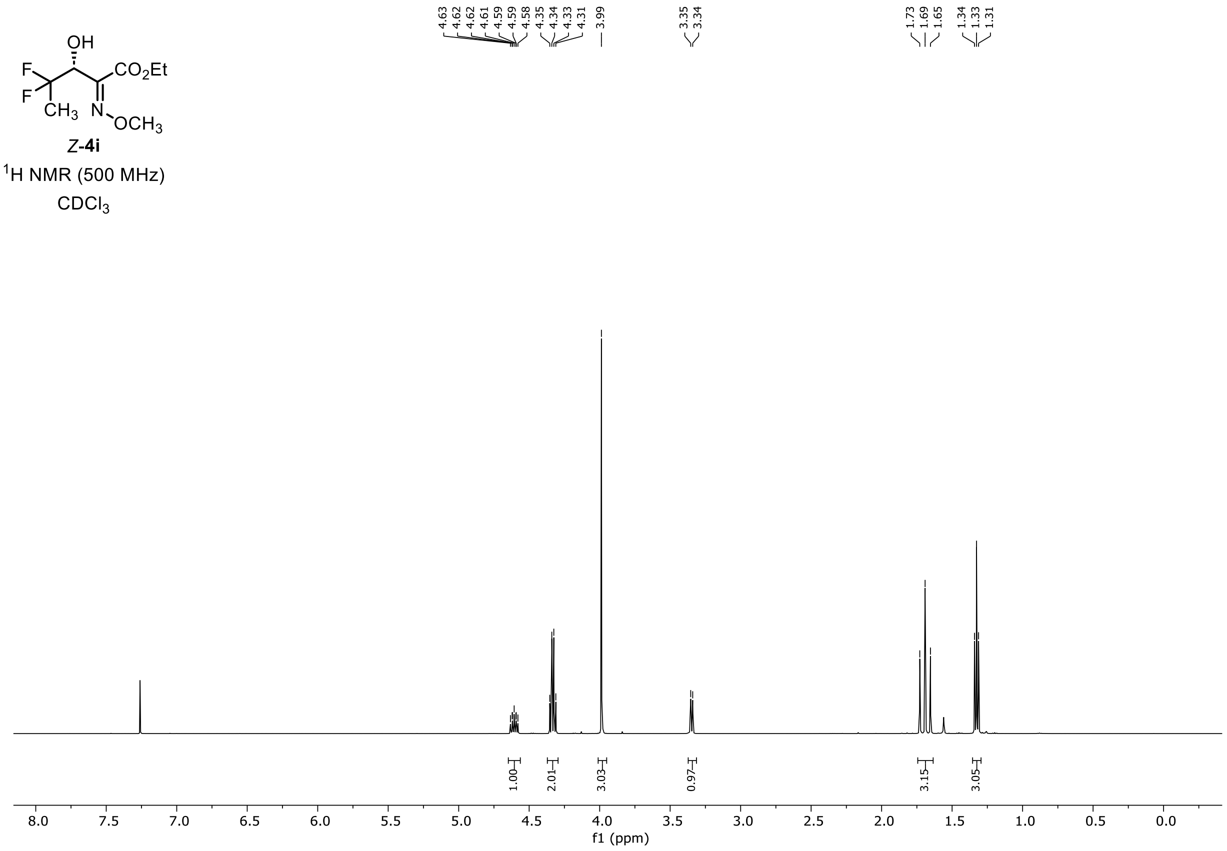
OH
F
F
CO2Et
CH3
N
OCH3
Z-4i
1H NMR (500 MHz)
CDCl3
4.63 4.62 4.62 4.61 4.59 4.59 4.58 4.35 4.34 4.33 4.31 3.99
3.35 3.34
1.73 1.69 1.65 1.34 1.33 1.31
1.00 2.01 3.03 0.97 3.15 3.05
8.0 7.5 7.0 6.5 6.0 5.5 5.0 4.5 4.0 3.5 3.0 2.5 2.0 1.5 1.0 0.5 0.0
f1 (ppm)

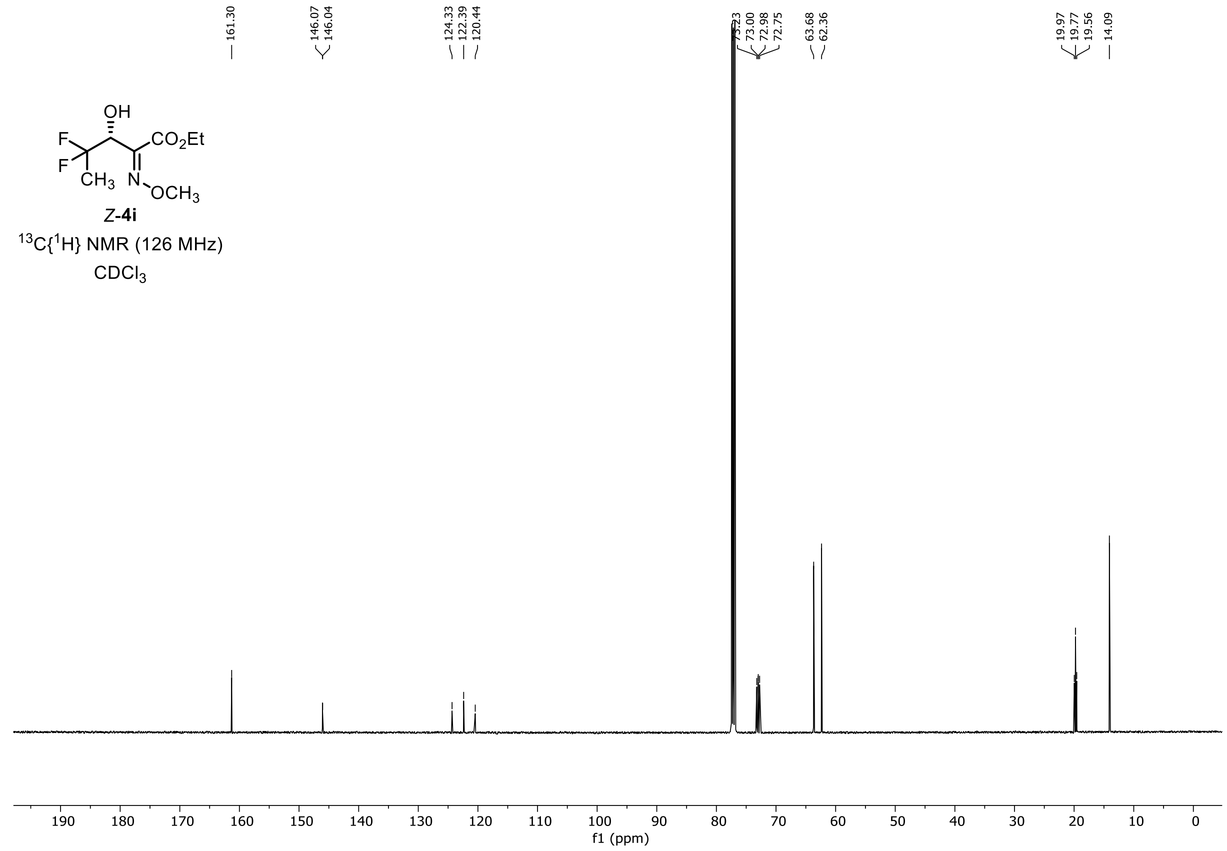
161.30
146.07 146.04
124.33 122.39 120.44
73.00 72.98 72.75
63.68 62.36
19.97 19.77 19.56 14.09
OH
F
F
CO2Et
CH3
N
OCH3
Z-4i
13C{1H} NMR (126 MHz)
CDCl3
190 180 170 160 150 140 130 120 110 100 90 80 70 60 50 40 30 20 10 0
f1 (ppm)

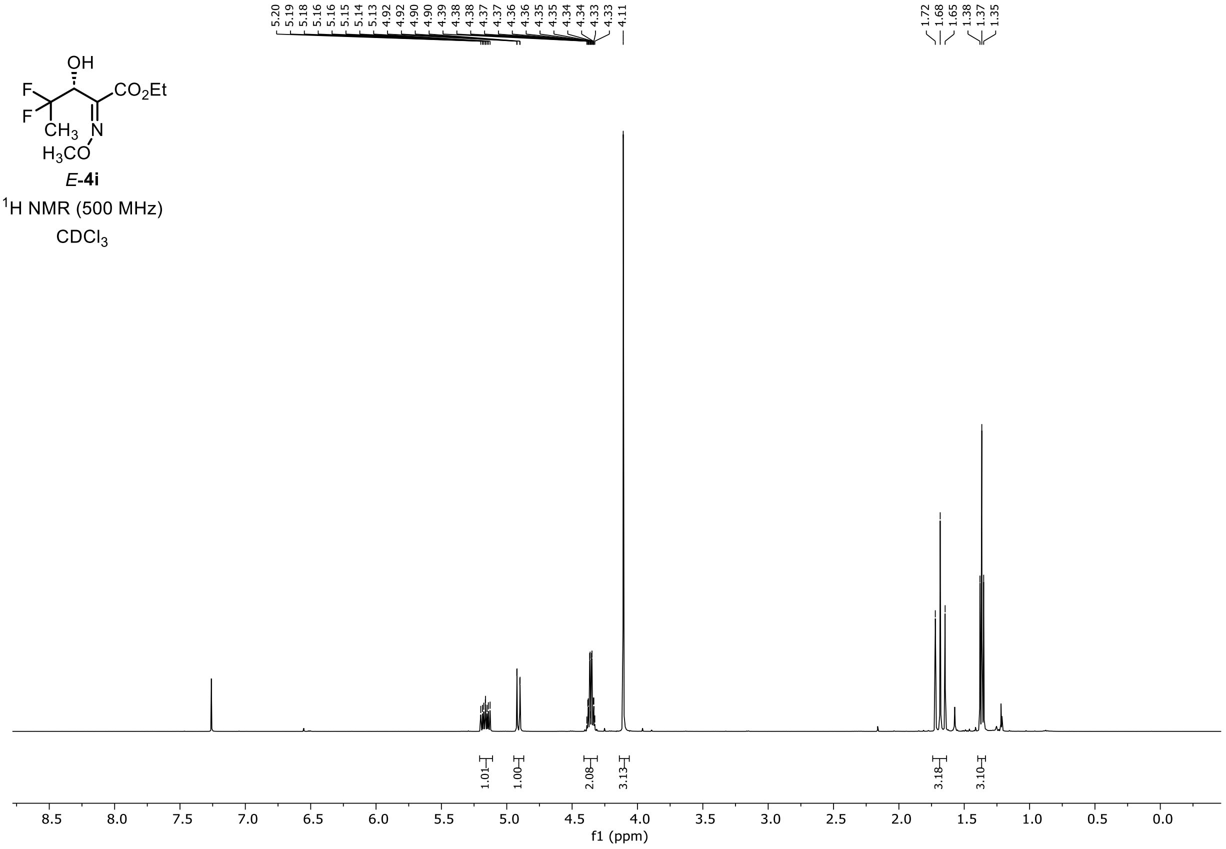

E-**4i**

^{1}H NMR (500 MHz)

$CDCl_3$

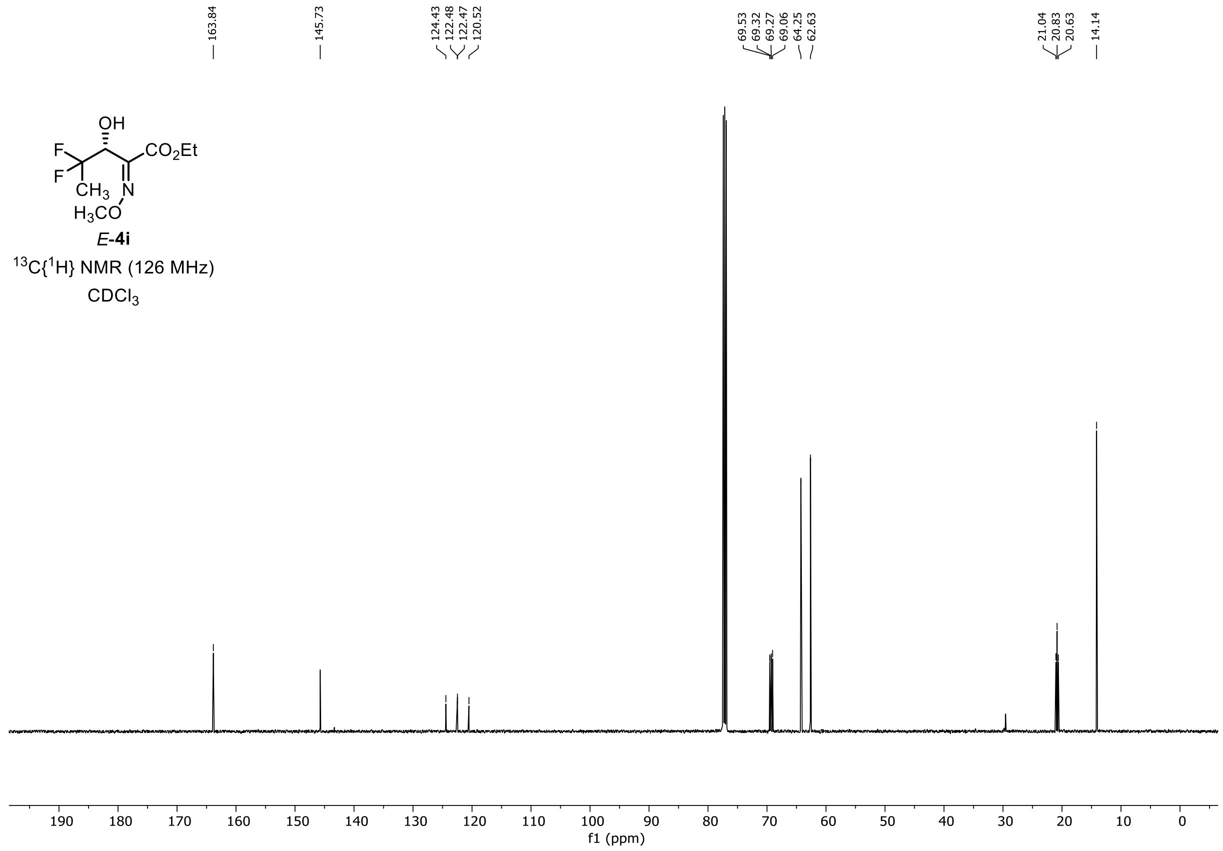

E-**4i**

$^{13}C\{^{1}H\}$ NMR (126 MHz)

$CDCl_3$

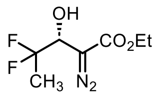

25 (ca. 85% purity)
^{1}H NMR (500 MHz)
$CDCl_3$

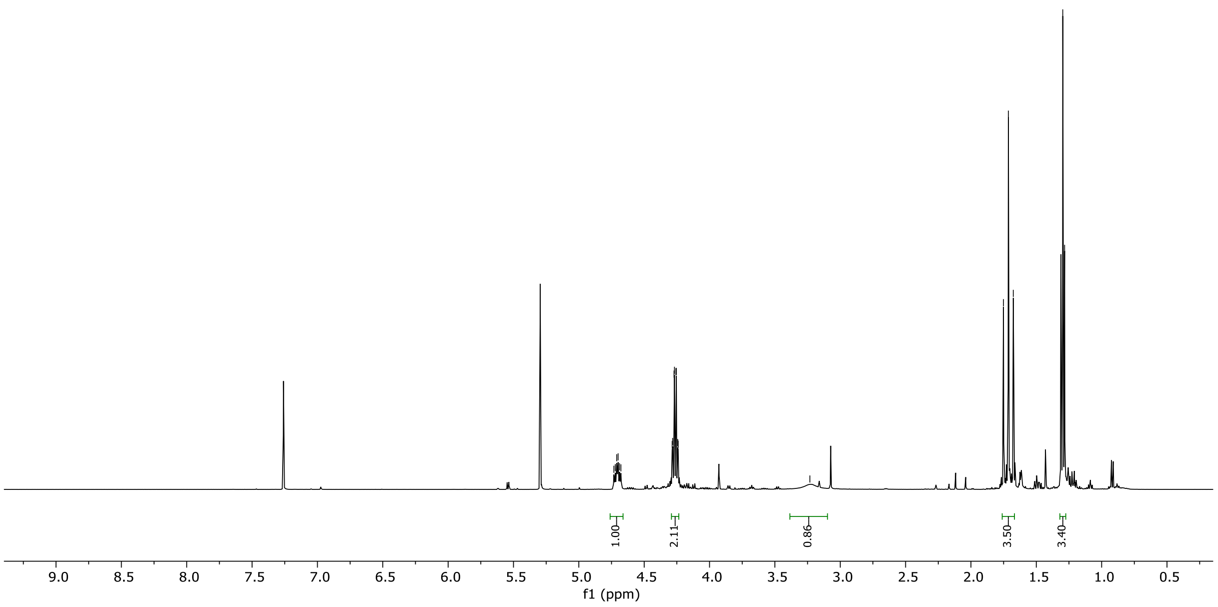

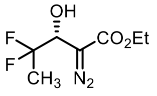

25 (ca. 85% purity)
$^{13}C\{^{1}H\}$ NMR (126 MHz)
$CDCl_3$

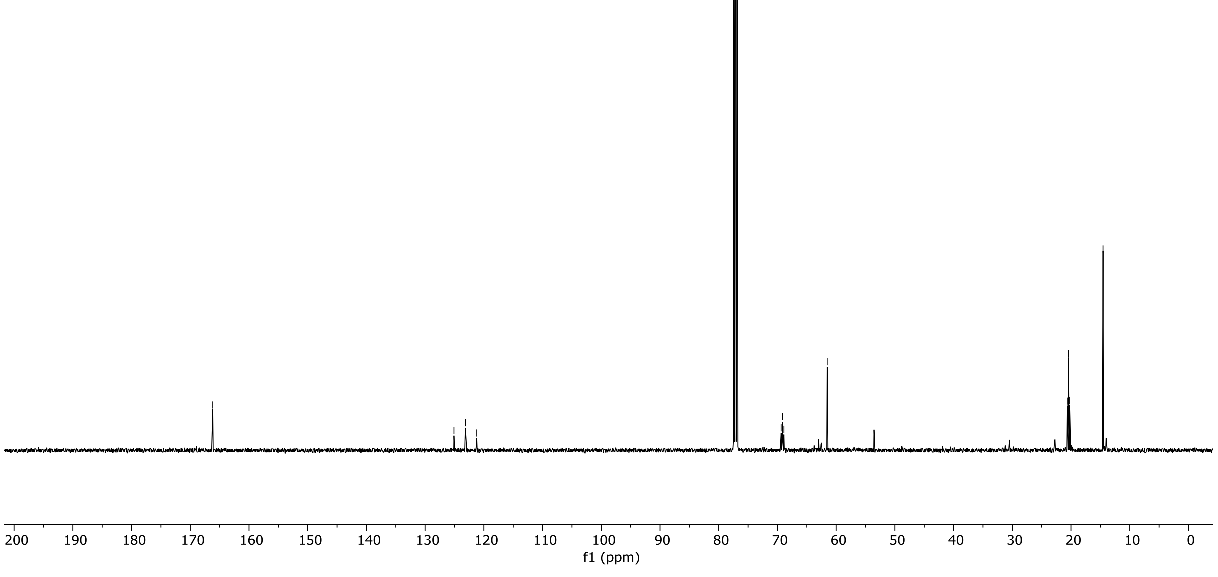

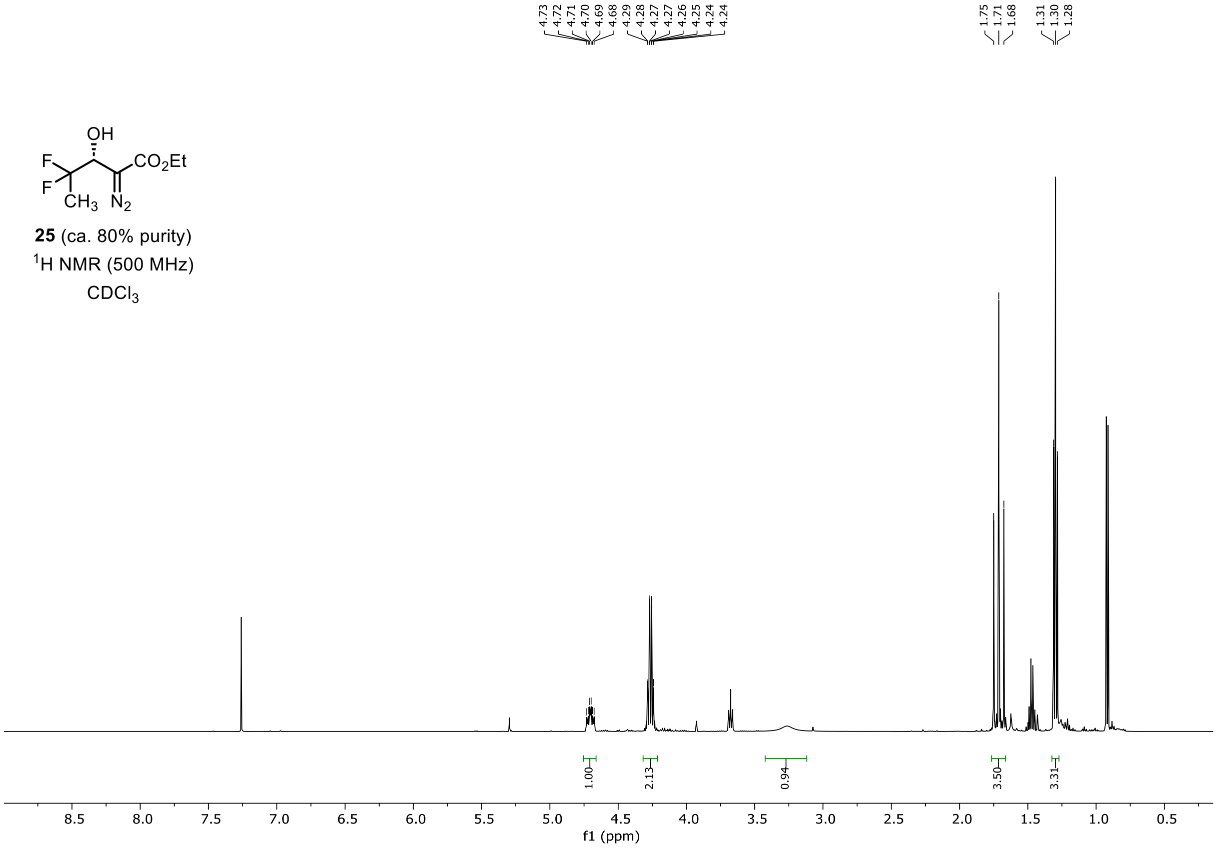
4.73 4.72 4.71 4.70 4.69 4.68 4.29 4.28 4.27 4.27 4.26 4.25 4.24 4.24
1.75 1.71 1.68
1.31 1.30 1.28
OH
F
F
CH3
CO2Et
N2
25 (ca. 80% purity)
1H NMR (500 MHz)
CDCl3
1.00
2.13
0.94
3.50
3.31
8.5 8.0 7.5 7.0 6.5 6.0 5.5 5.0 4.5 4.0 3.5 3.0 2.5 2.0 1.5 1.0 0.5
f1 (ppm)

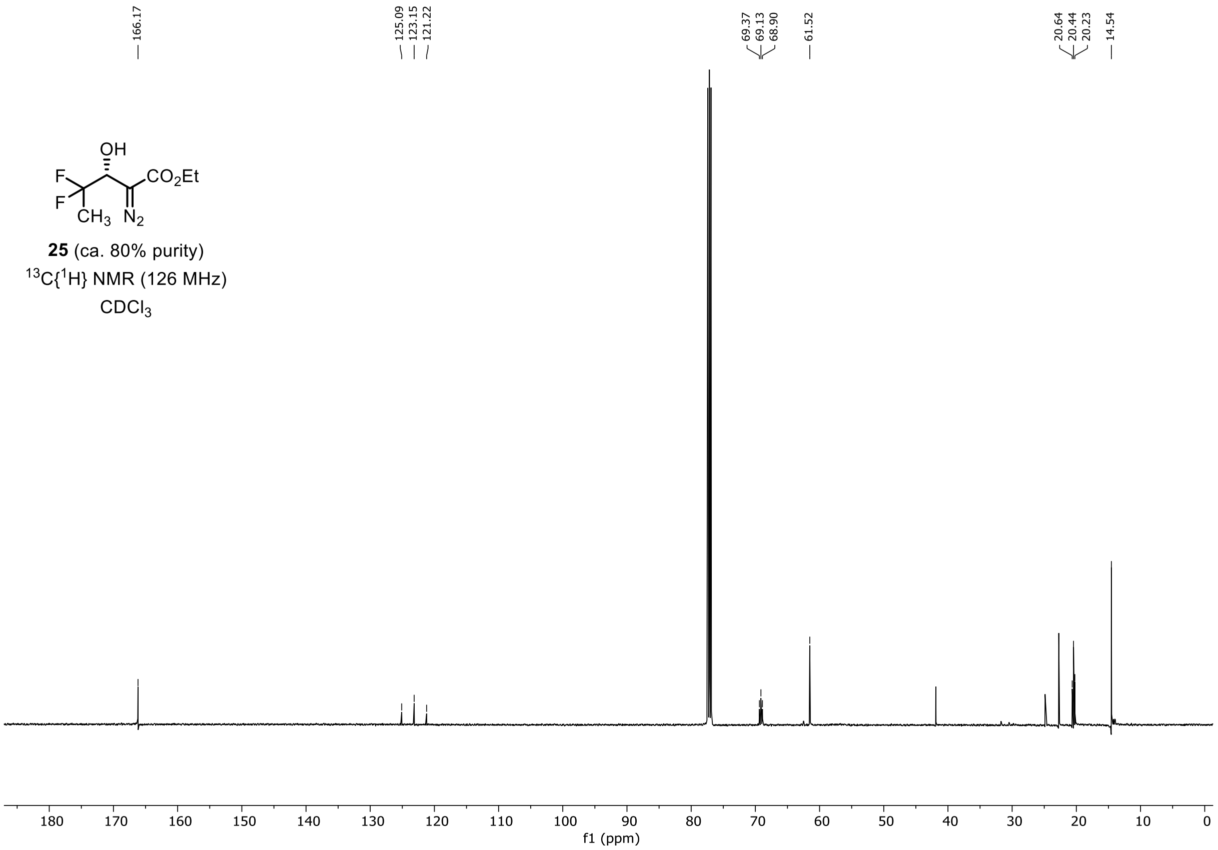
166.17
125.09 123.15 121.22
69.37 69.13 68.90
61.52
20.64 20.44 20.23
14.54
OH
F
F
CH3
CO2Et
N2
25 (ca. 80% purity)
13C{1H} NMR (126 MHz)
CDCl3
180 170 160 150 140 130 120 110 100 90 80 70 60 50 40 30 20 10 0
f1 (ppm)

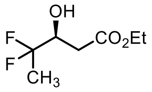

6i

^{1}H NMR (500 MHz)

$CDCl_3$

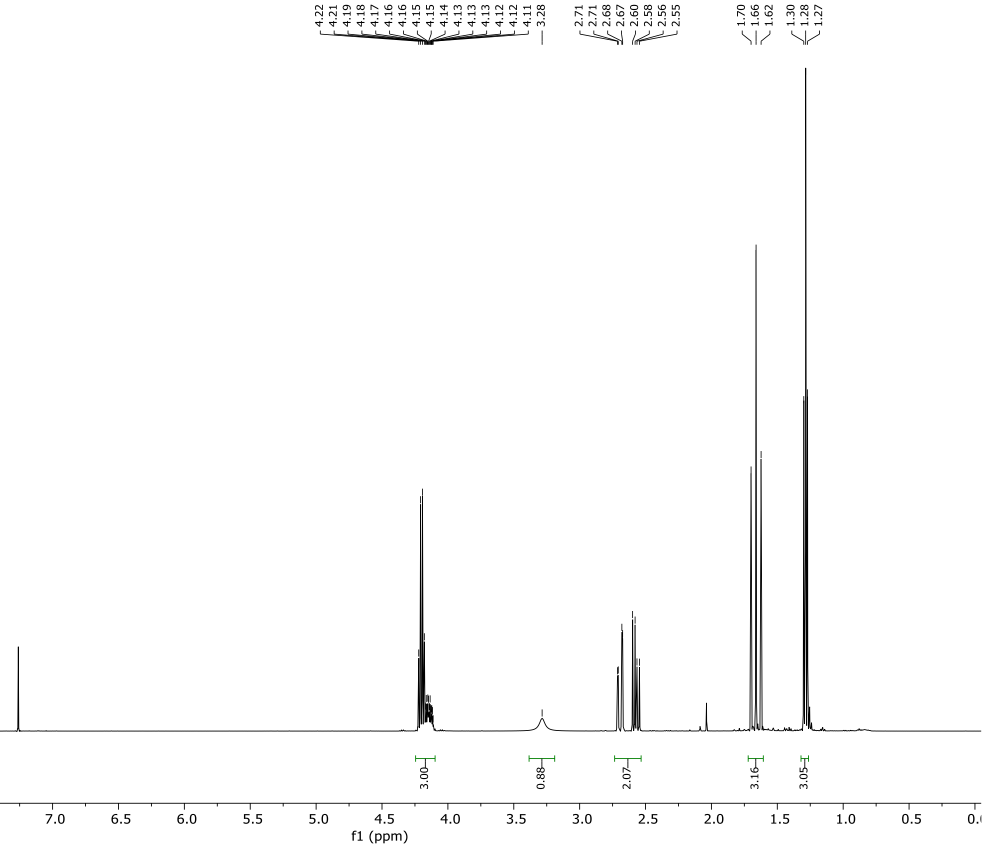

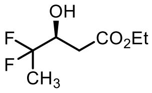

6i

$^{13}C\{^1H\}$ NMR (126 MHz)

$CDCl_3$

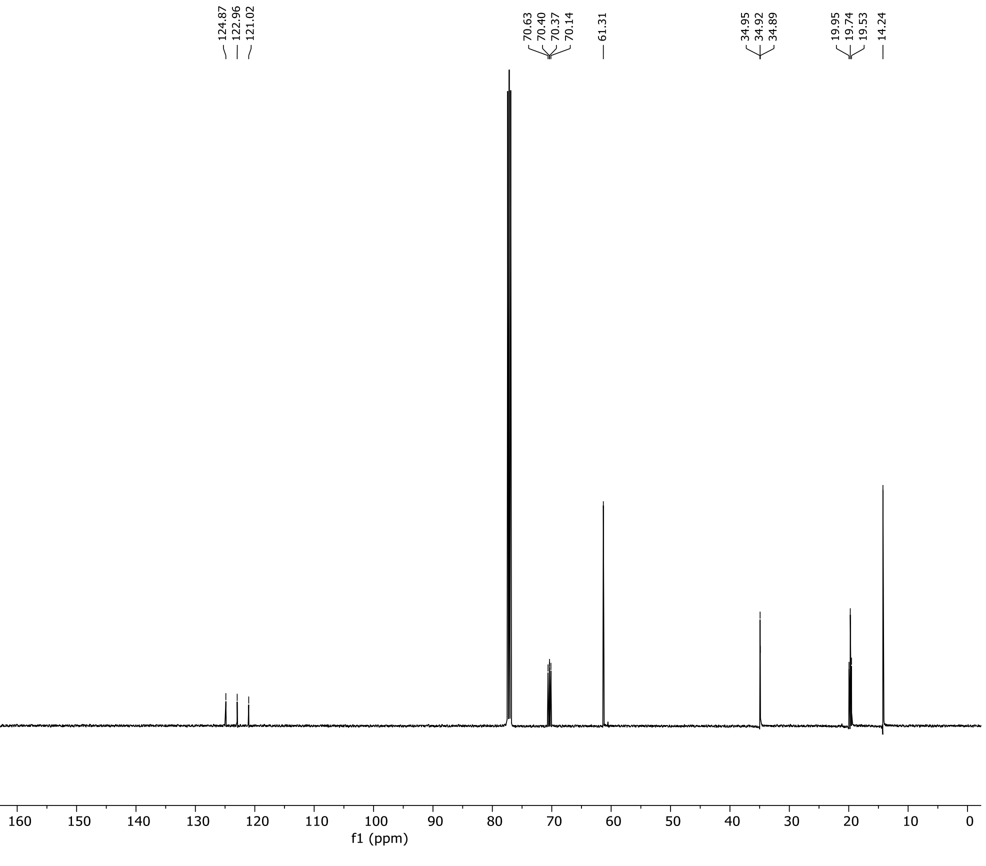

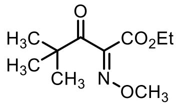

Z-**3j**

^{1}H NMR (300 MHz)

$CDCl_3$

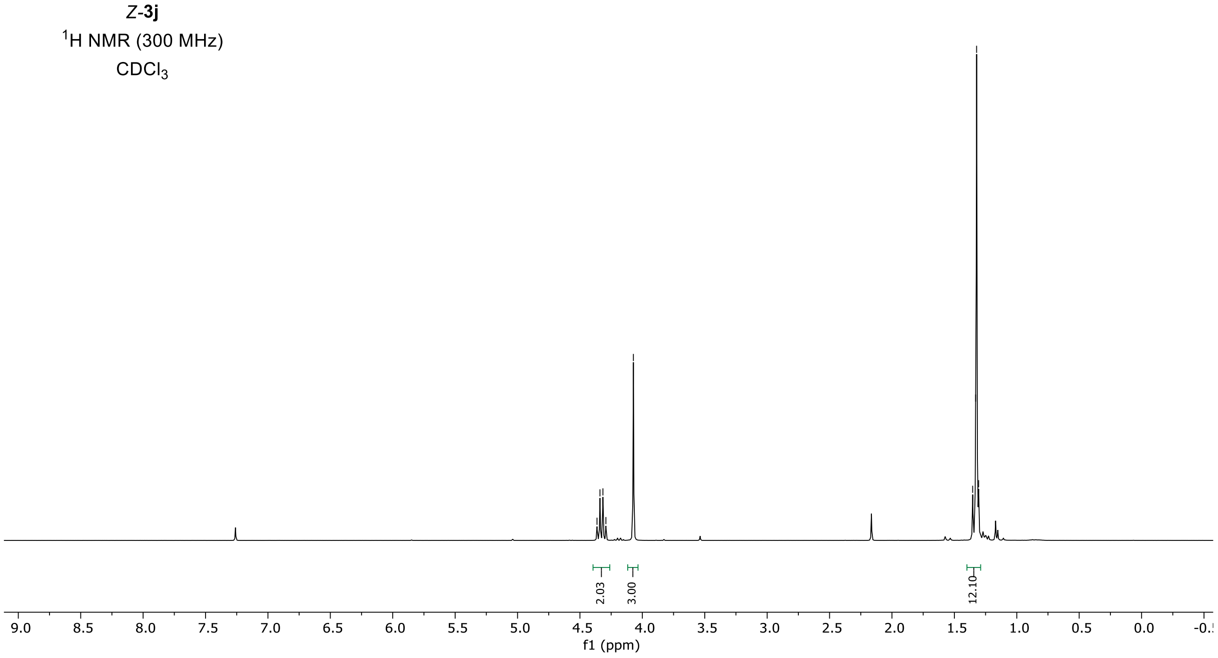

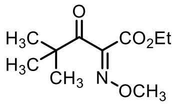

Z-**3j**

^{13}C{^{1}H} NMR (126 MHz)

$CDCl_3$

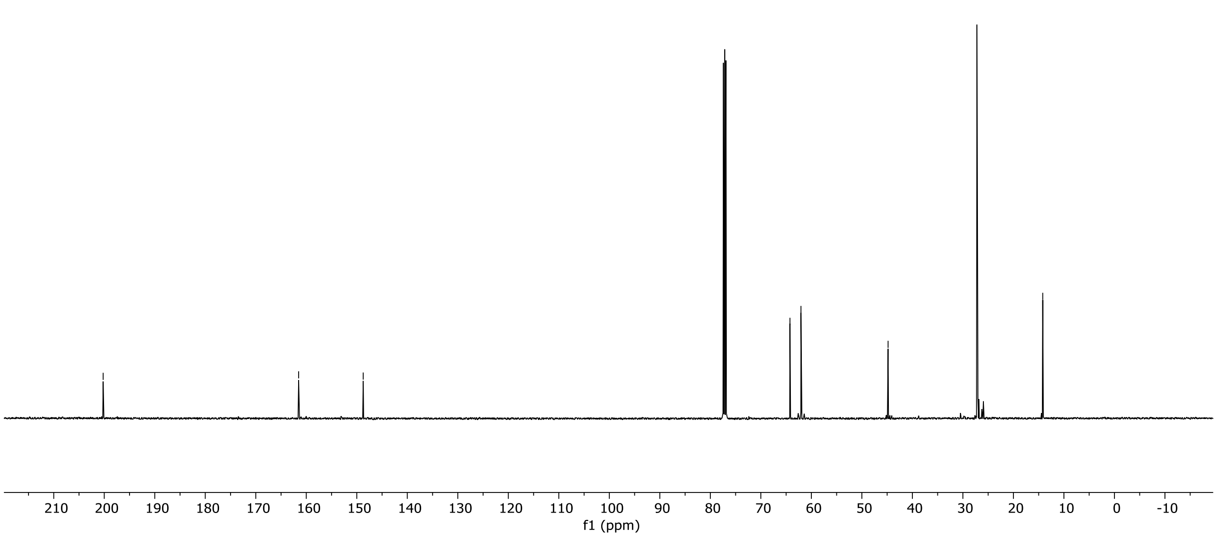

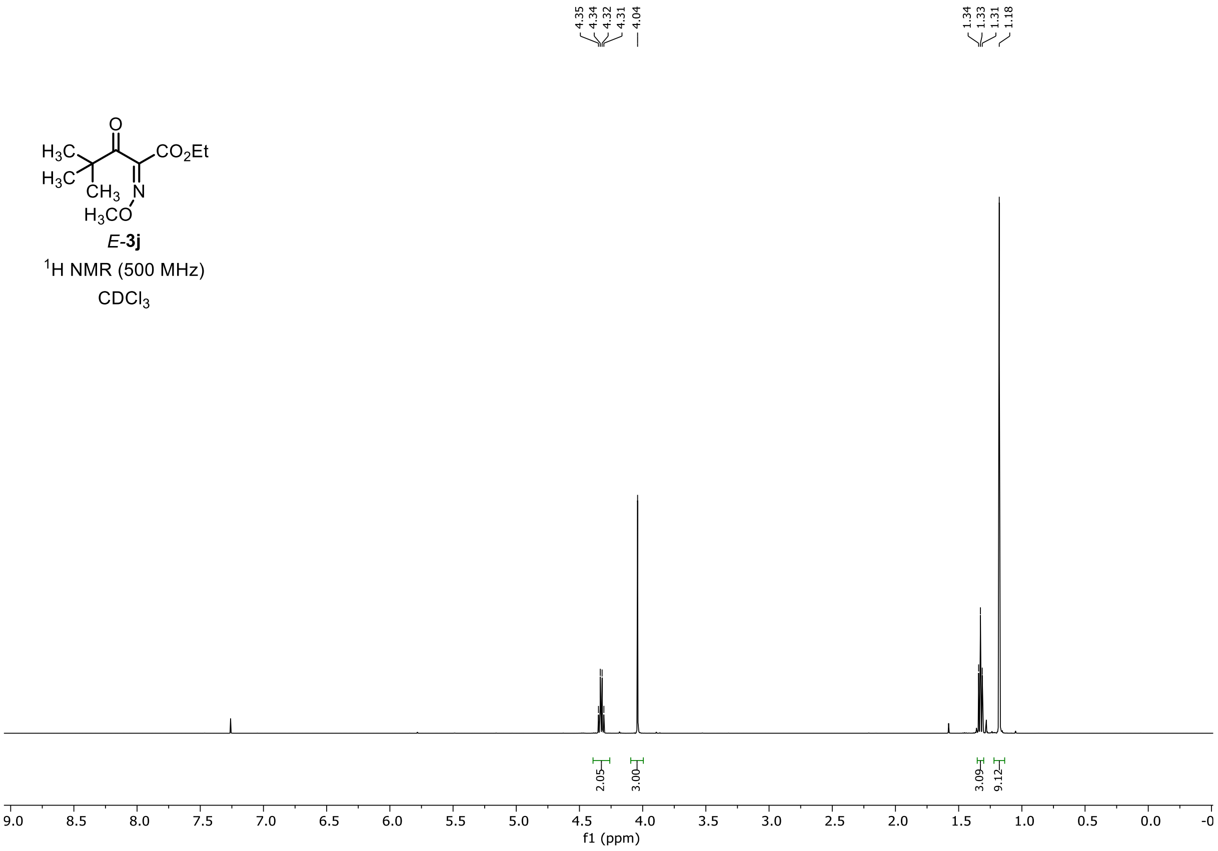
4.35
4.34
4.32
4.31
4.04
1.34
1.33
1.31
1.18
E-3j
1H NMR (500 MHz)
CDCl3
2.05
3.00
3.09
9.12
9.0 8.5 8.0 7.5 7.0 6.5 6.0 5.5 5.0 4.5 4.0 3.5 3.0 2.5 2.0 1.5 1.0 0.5 0.0
f1 (ppm)

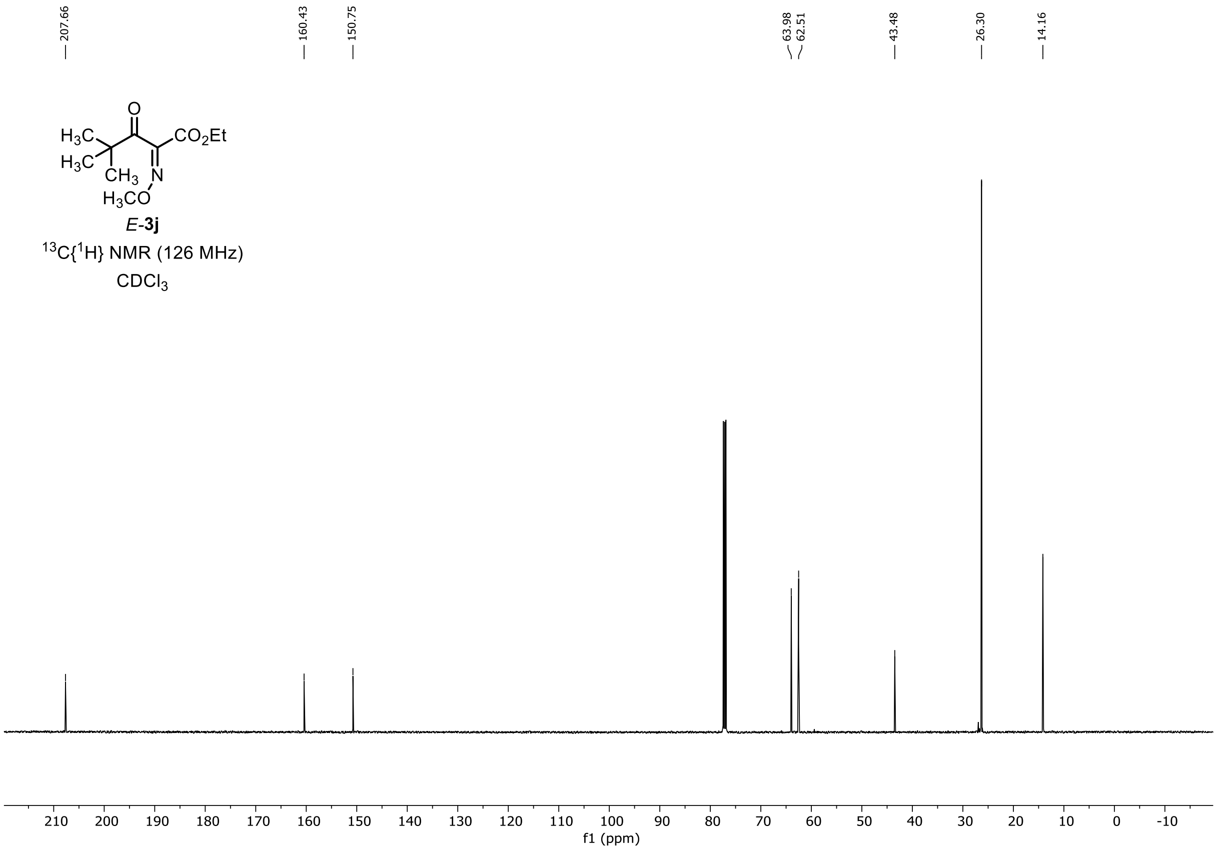
207.66
160.43
150.75
63.98
62.51
43.48
26.30
14.16
E-3j
13C{1H} NMR (126 MHz)
CDCl3
210 200 190 180 170 160 150 140 130 120 110 100 90 80 70 60 50 40 30 20 10 0 -10
f1 (ppm)

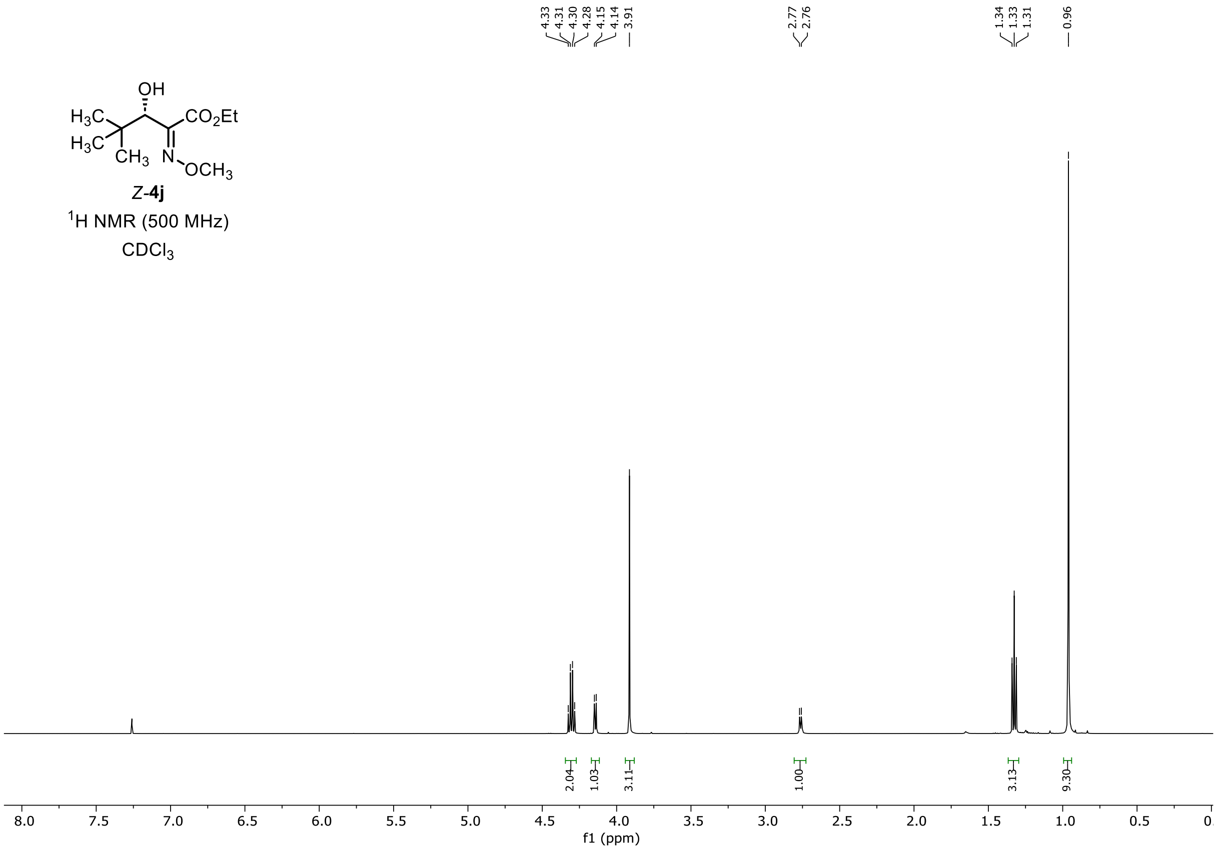
OH
H3C
CO2Et
H3C
CH3
N
OCH3
Z-4j
1H NMR (500 MHz)
CDCl3
4.33
4.31
4.30
4.28
4.15
4.14
3.91
2.77
2.76
1.34
1.33
1.31
0.96
2.04
1.03
3.11
1.00
3.13
9.30
8.0 7.5 7.0 6.5 6.0 5.5 5.0 4.5 4.0 3.5 3.0 2.5 2.0 1.5 1.0 0.5 0.
f1 (ppm)

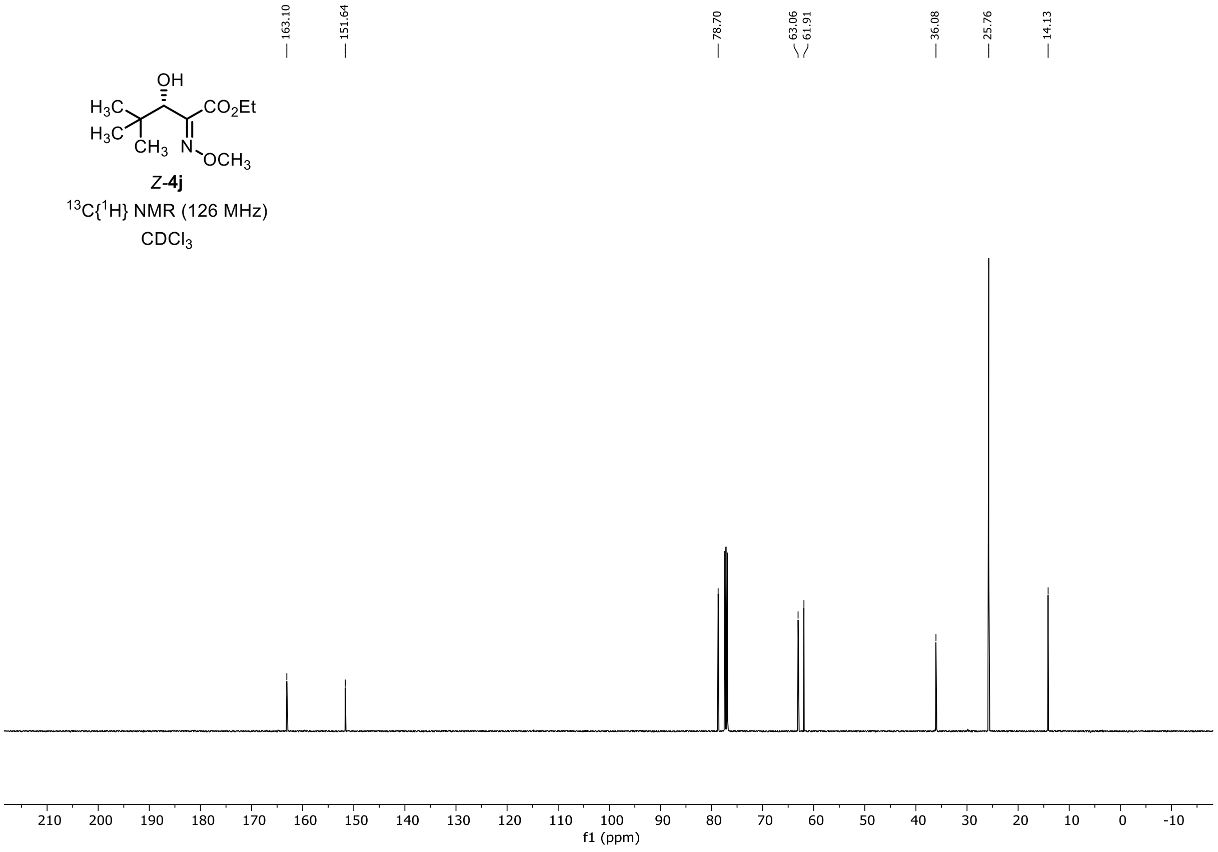
OH
H3C
CO2Et
H3C
CH3
N
OCH3
Z-4j
13C{1H} NMR (126 MHz)
CDCl3
163.10
151.64
78.70
63.06
61.91
36.08
25.76
14.13
210 200 190 180 170 160 150 140 130 120 110 100 90 80 70 60 50 40 30 20 10 0 -10
f1 (ppm)

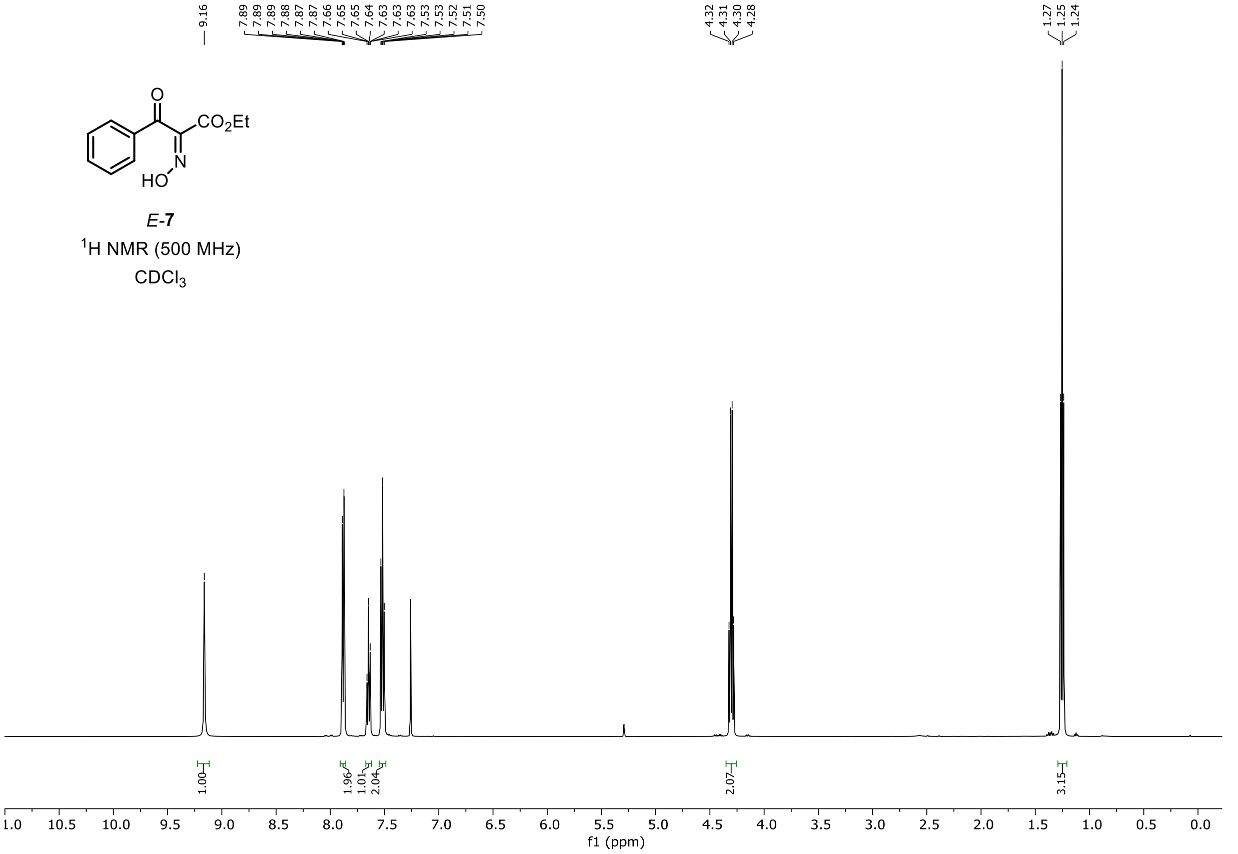
O
CO2Et
N
HO
E-7
1H NMR (500 MHz)
CDCl3
9.16
7.89
7.89
7.89
7.88
7.87
7.87
7.66
7.65
7.65
7.64
7.63
7.63
7.63
7.53
7.53
7.52
7.51
7.50
4.32
4.31
4.30
4.28
1.27
1.25
1.24
1.00
1.96
1.01
2.04
2.07
3.15
10.5
10.0
9.5
9.0
8.5
8.0
7.5
7.0
6.5
6.0
5.5
5.0
4.5
4.0
3.5
3.0
2.5
2.0
1.5
1.0
0.5
0.0
f1 (ppm)

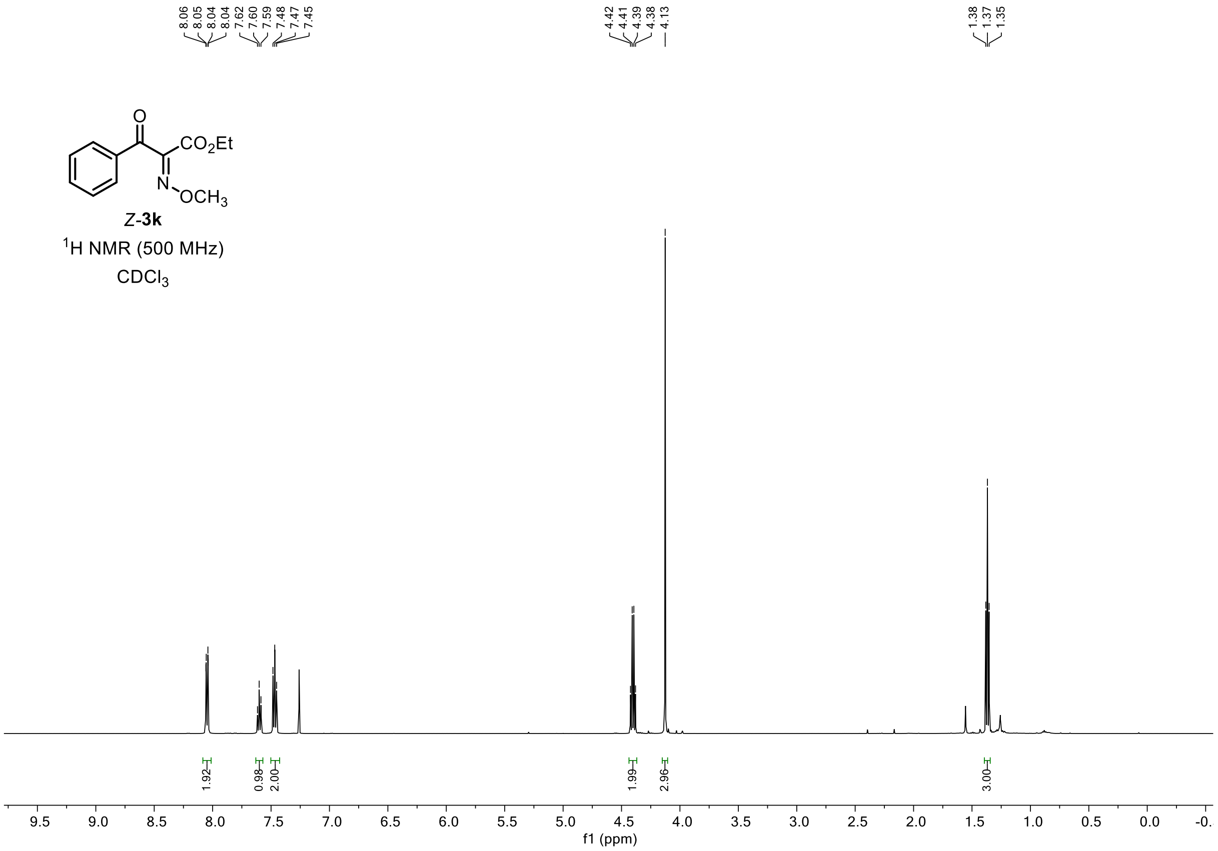
8.06
8.05
8.04
8.04
7.62
7.60
7.59
7.48
7.47
7.45
4.42
4.41
4.39
4.38
4.13
1.38
1.37
1.35
CO2Et
N
OCH3
Z-3k
1H NMR (500 MHz)
CDCl3
1.92
0.98
2.00
1.99
2.96
3.00
f1 (ppm)

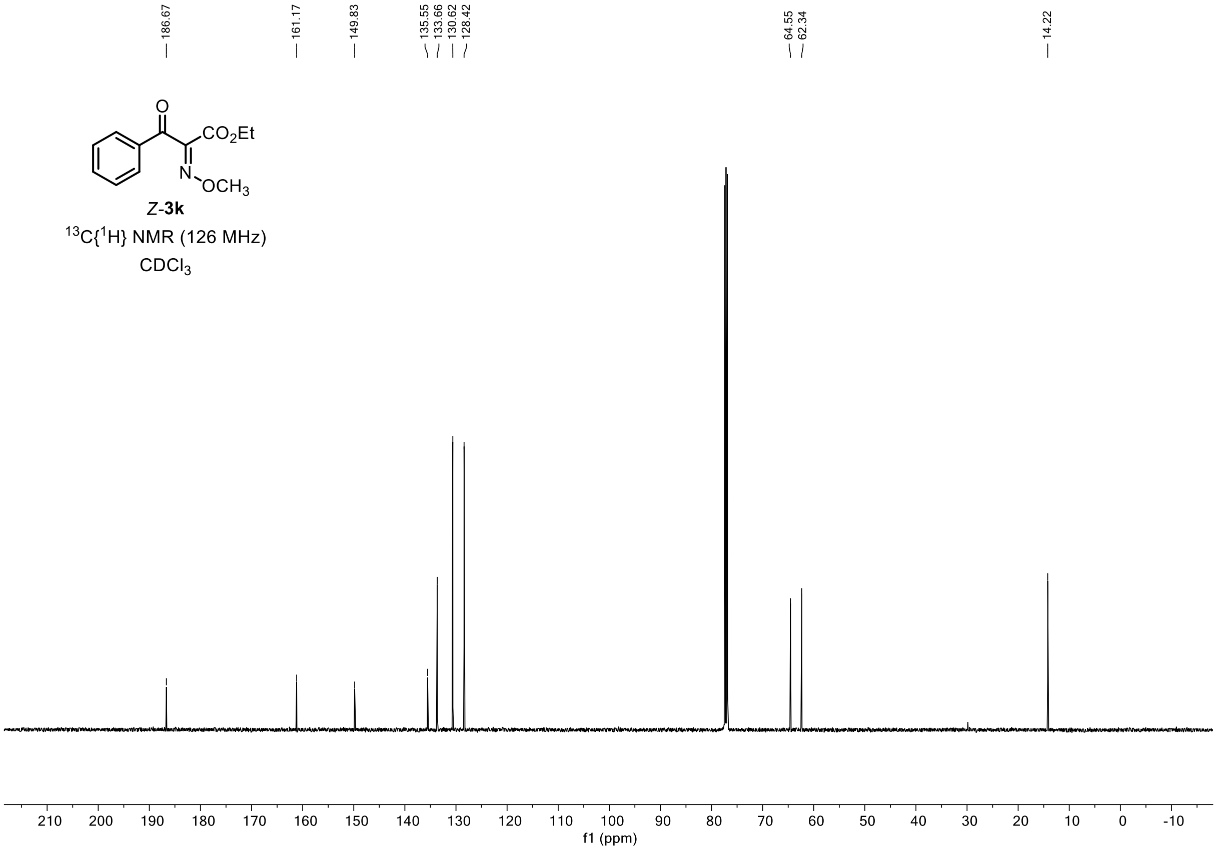
186.67
161.17
149.83
135.55
133.66
130.62
128.42
64.55
62.34
14.22
CO2Et
N
OCH3
Z-3k
13C{1H} NMR (126 MHz)
CDCl3
f1 (ppm)

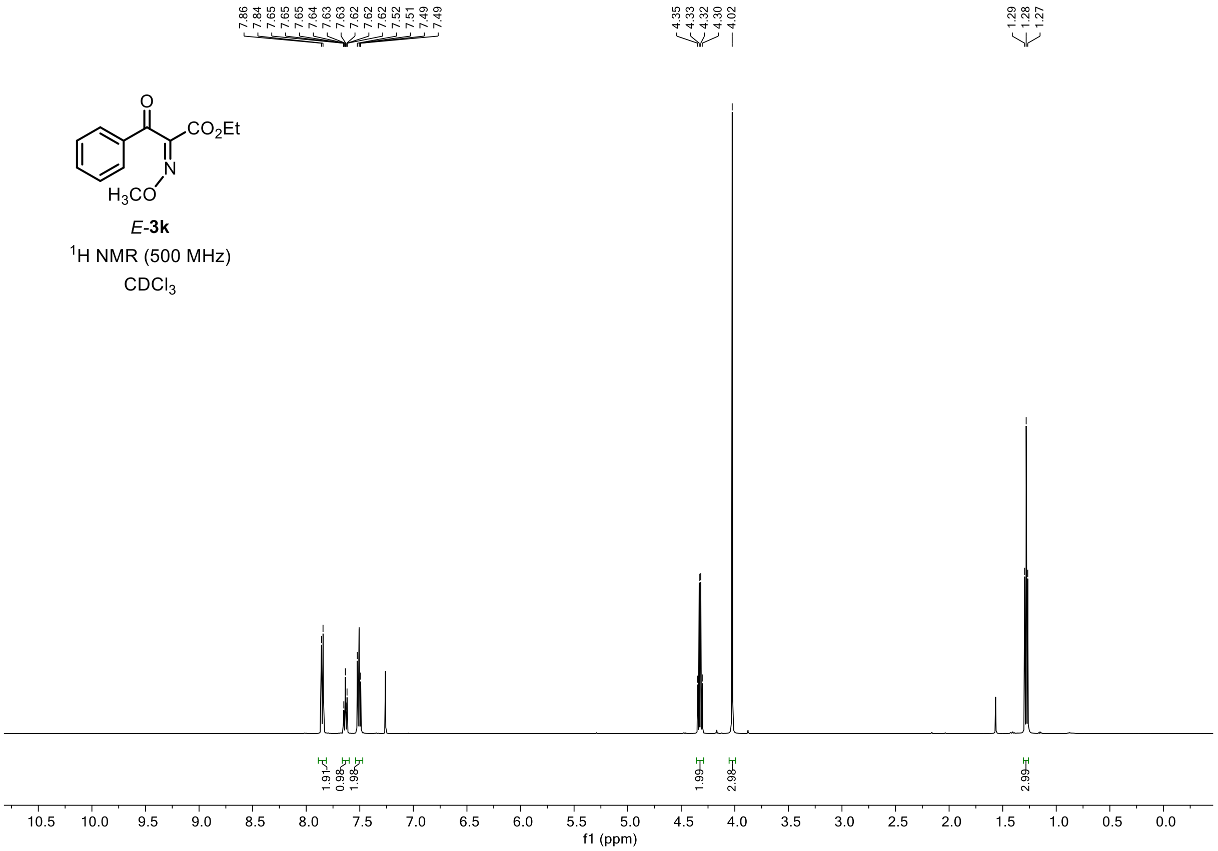

O
CO2Et
N
H3CO
E-3k
1H NMR (500 MHz)
CDCl3
f1 (ppm)

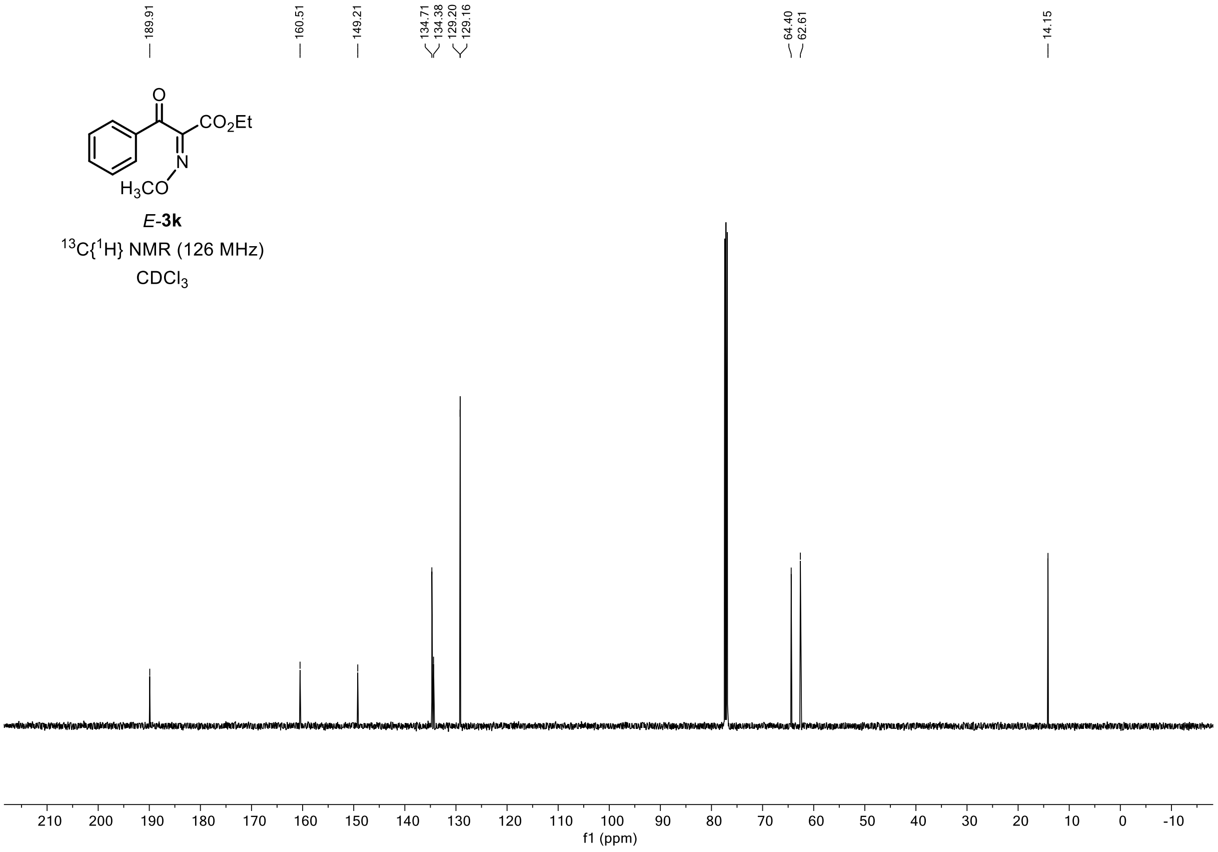

O
CO2Et
N
H3CO
E-3k
13C{1H} NMR (126 MHz)
CDCl3
f1 (ppm)

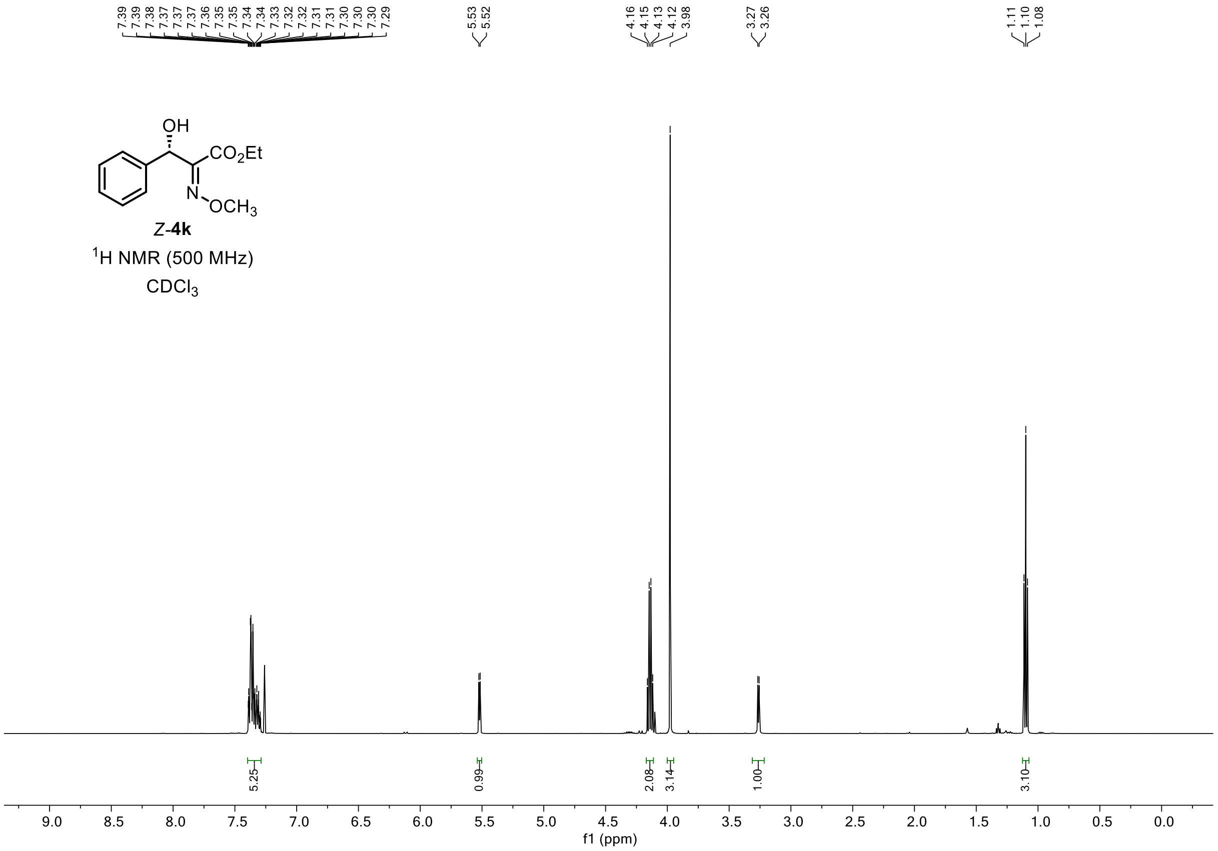
7.39 7.39 7.38 7.37 7.37 7.36 7.35 7.35 7.34 7.33 7.32 7.32 7.31 7.31 7.30 7.30 7.30 7.29
5.53 5.52
4.16 4.15 4.13 4.12 3.98
3.27 3.26
1.11 1.10 1.08
OH
CO2Et
N
OCH3
Z-4k
1H NMR (500 MHz)
CDCl3
5.25
0.99
2.08
3.14
1.00
3.10
9.0 8.5 8.0 7.5 7.0 6.5 6.0 5.5 5.0 4.5 4.0 3.5 3.0 2.5 2.0 1.5 1.0 0.5 0.0
f1 (ppm)

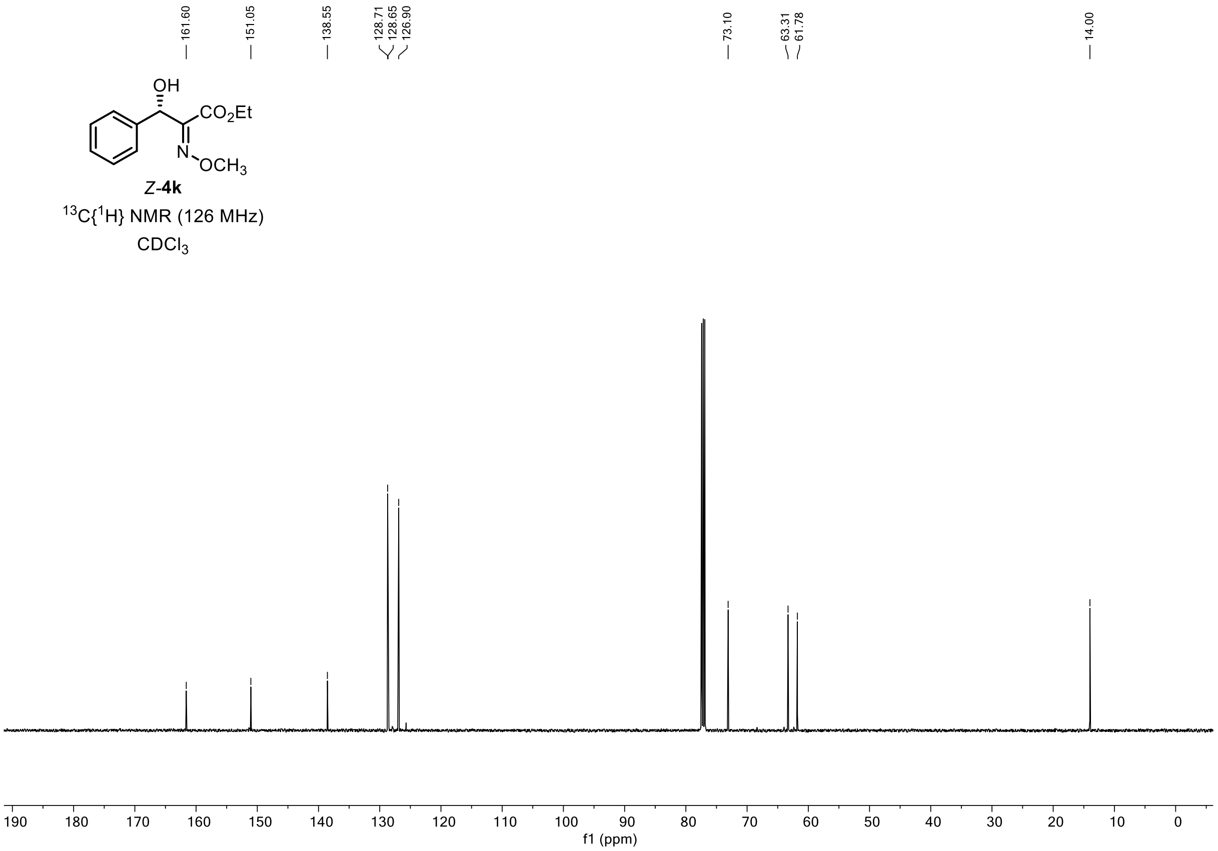
161.60
151.05
138.55
128.71 128.65 126.90
73.10
63.31 61.78
14.00
OH
CO2Et
N
OCH3
Z-4k
13C{1H} NMR (126 MHz)
CDCl3
190 180 170 160 150 140 130 120 110 100 90 80 70 60 50 40 30 20 10 0
f1 (ppm)

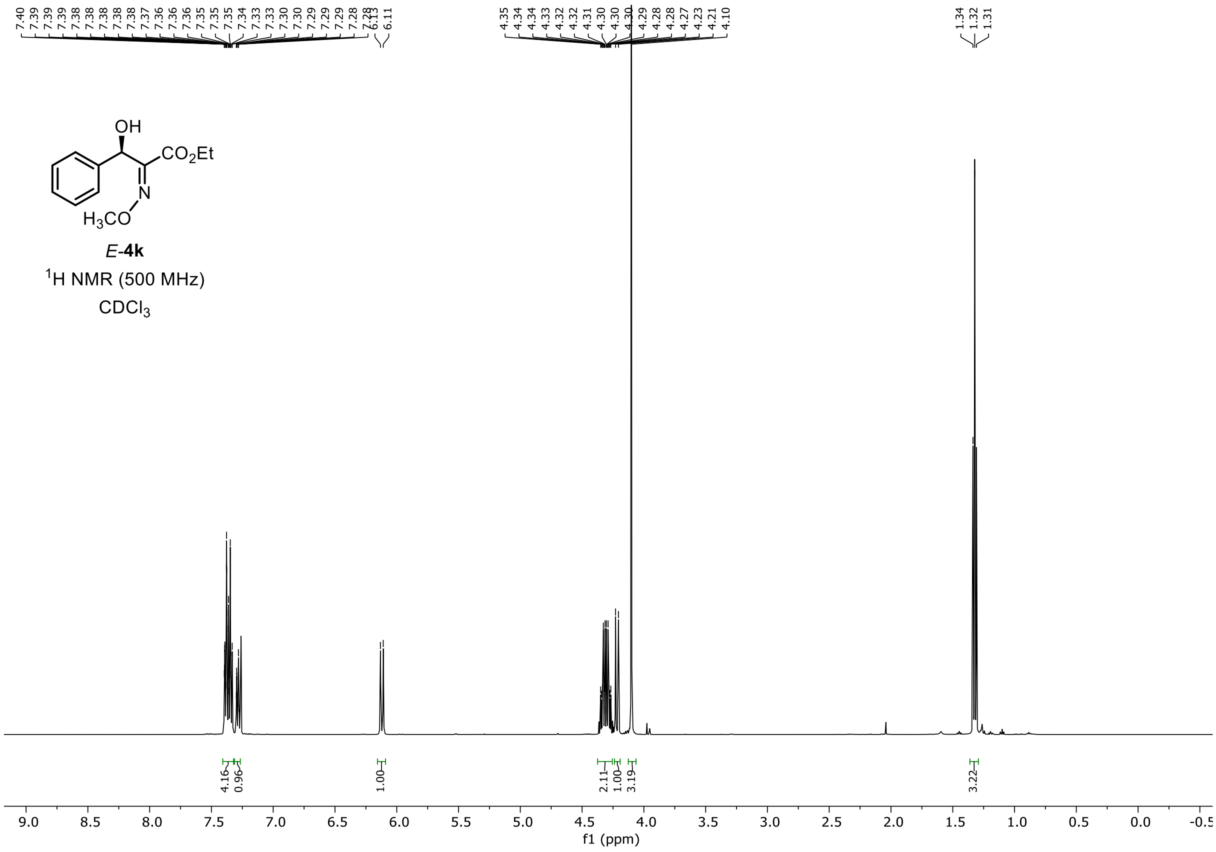
7.40 7.39 7.39 7.39 7.38 7.38 7.38 7.38 7.38 7.37 7.36 7.36 7.36 7.35 7.35 7.35 7.34 7.33 7.33 7.30 7.30 7.29 7.29 7.29 7.29 7.28 7.28 6.13 6.11
4.35 4.34 4.34 4.33 4.32 4.32 4.31 4.30 4.30 4.30 4.29 4.28 4.28 4.27 4.23 4.21 4.10
1.34 1.32 1.31
OH
CO_2Et
N
H_3CO
E-**4k**
^{1}H NMR (500 MHz)
$CDCl_3$
4.16 0.96 1.00 2.11 1.00 3.19 3.22
9.0 8.5 8.0 7.5 7.0 6.5 6.0 5.5 5.0 4.5 4.0 3.5 3.0 2.5 2.0 1.5 1.0 0.5 0.0 -0.5
f1 (ppm)

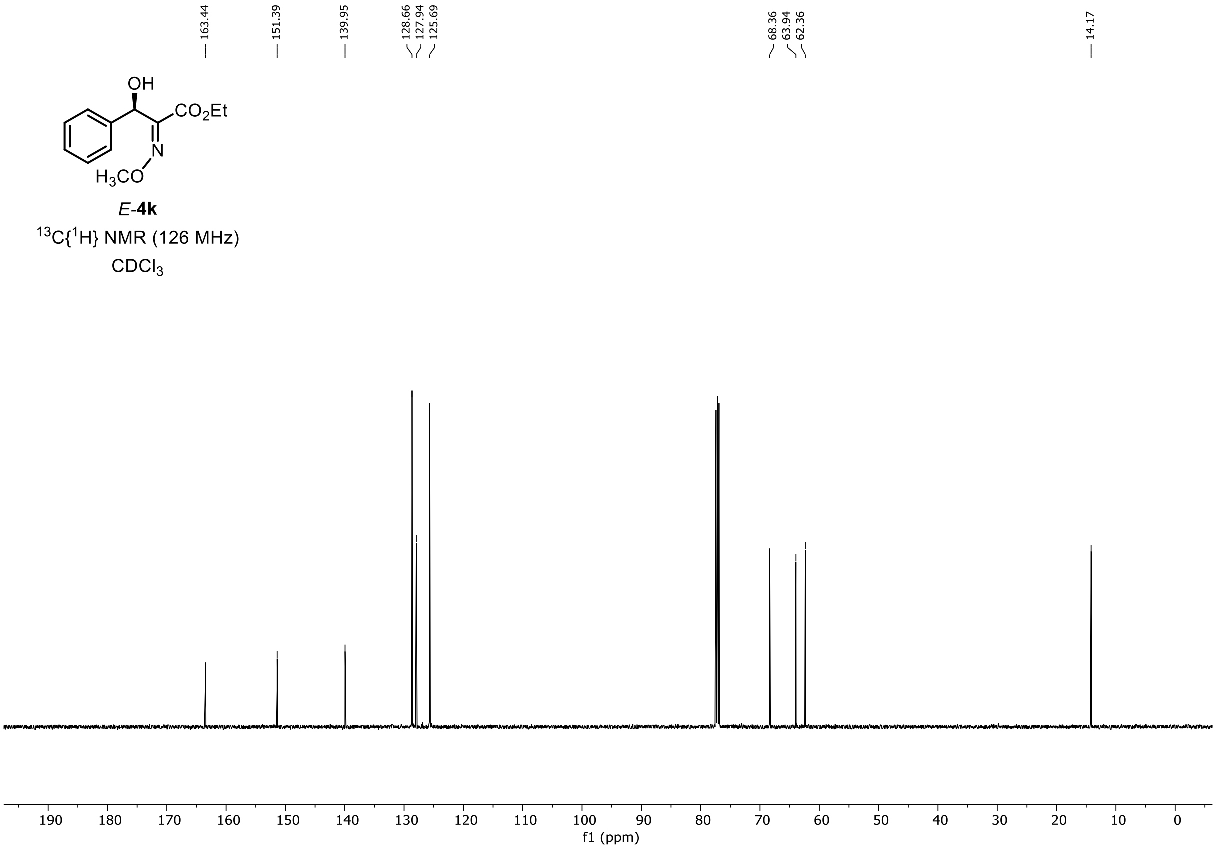
163.44
151.39
139.95
128.66 127.94 125.69
68.36 63.94 62.36
14.17
OH
CO_2Et
N
H_3CO
E-**4k**
^{13}C{^{1}H} NMR (126 MHz)
$CDCl_3$
190 180 170 160 150 140 130 120 110 100 90 80 70 60 50 40 30 20 10 0
f1 (ppm)

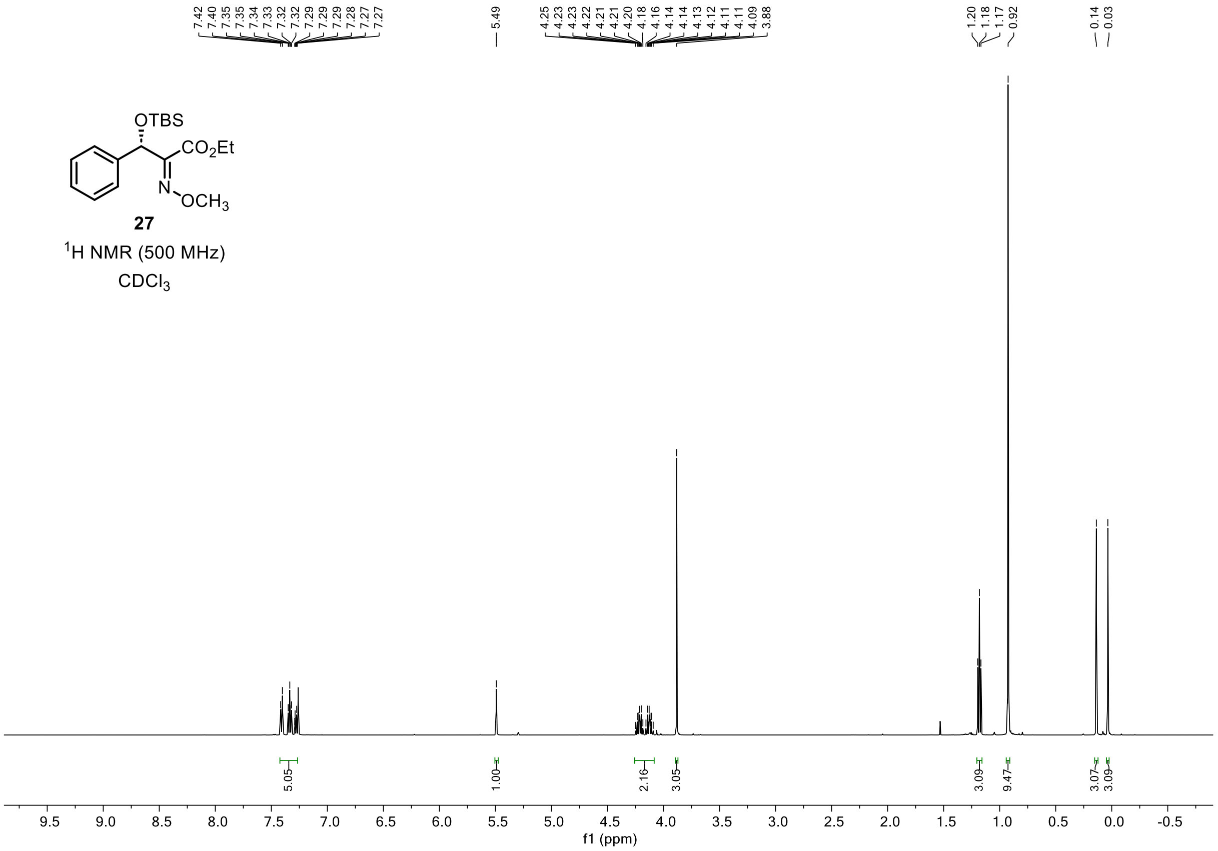

27

^{1}H NMR (500 MHz)

$CDCl_3$

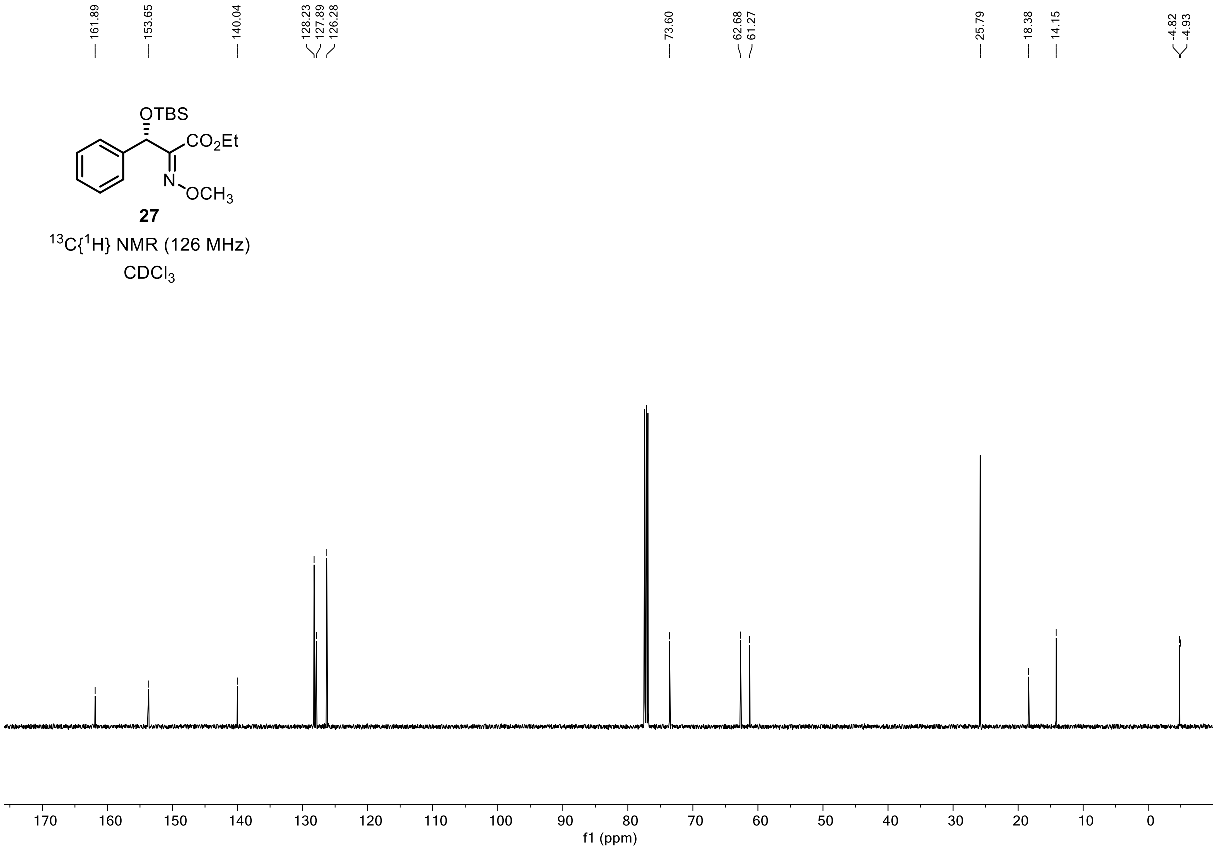

27

$^{13}C\{^{1}H\}$ NMR (126 MHz)

$CDCl_3$

26

^{1}H NMR (500 MHz)

$CDCl_3$

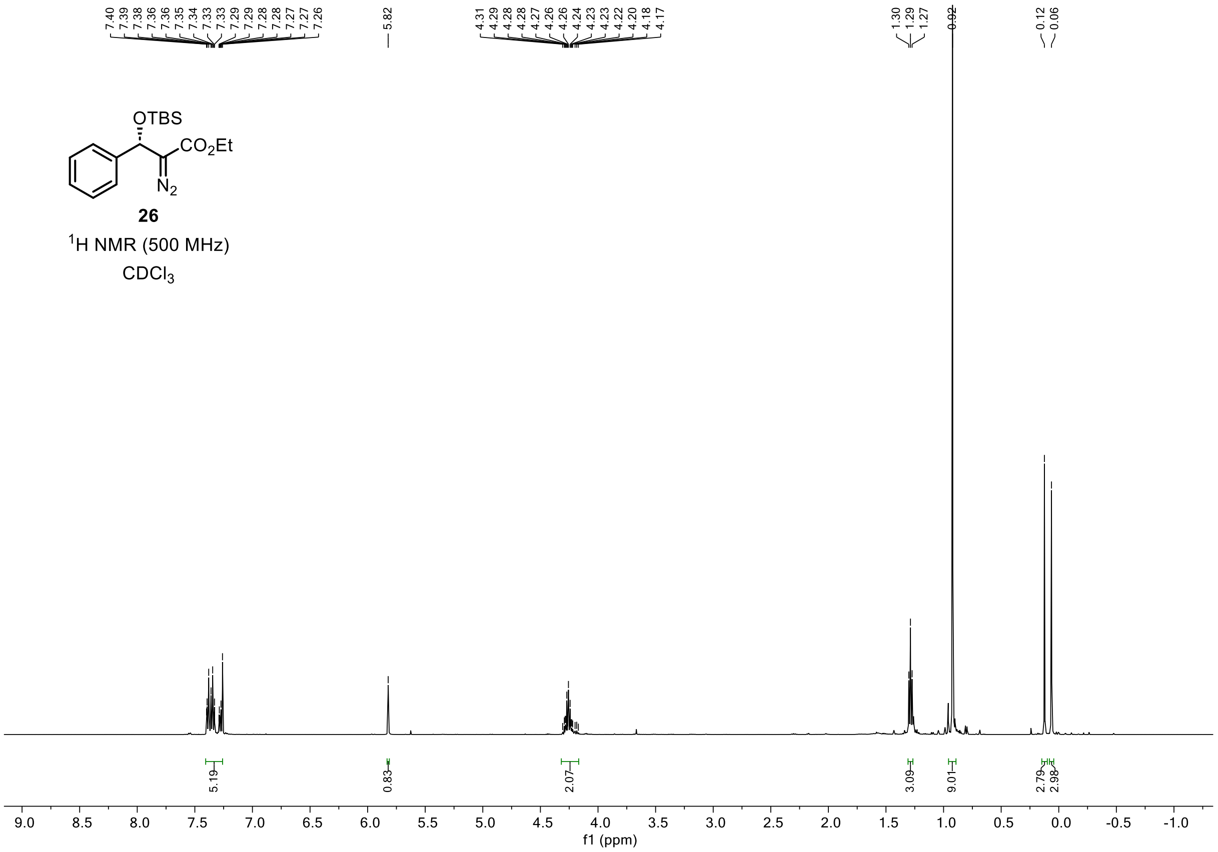

26

$^{13}C\{^{1}H\}$ NMR (126 MHz)

$CDCl_3$

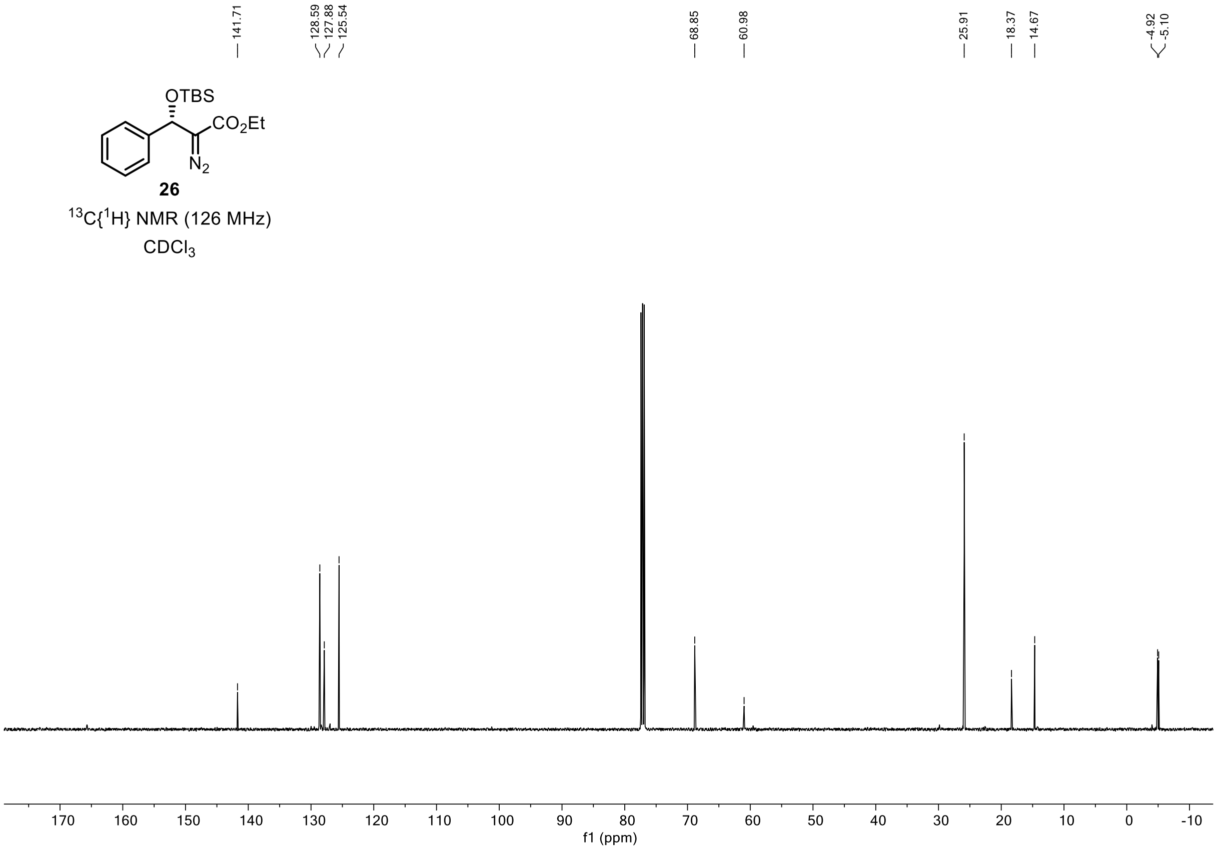

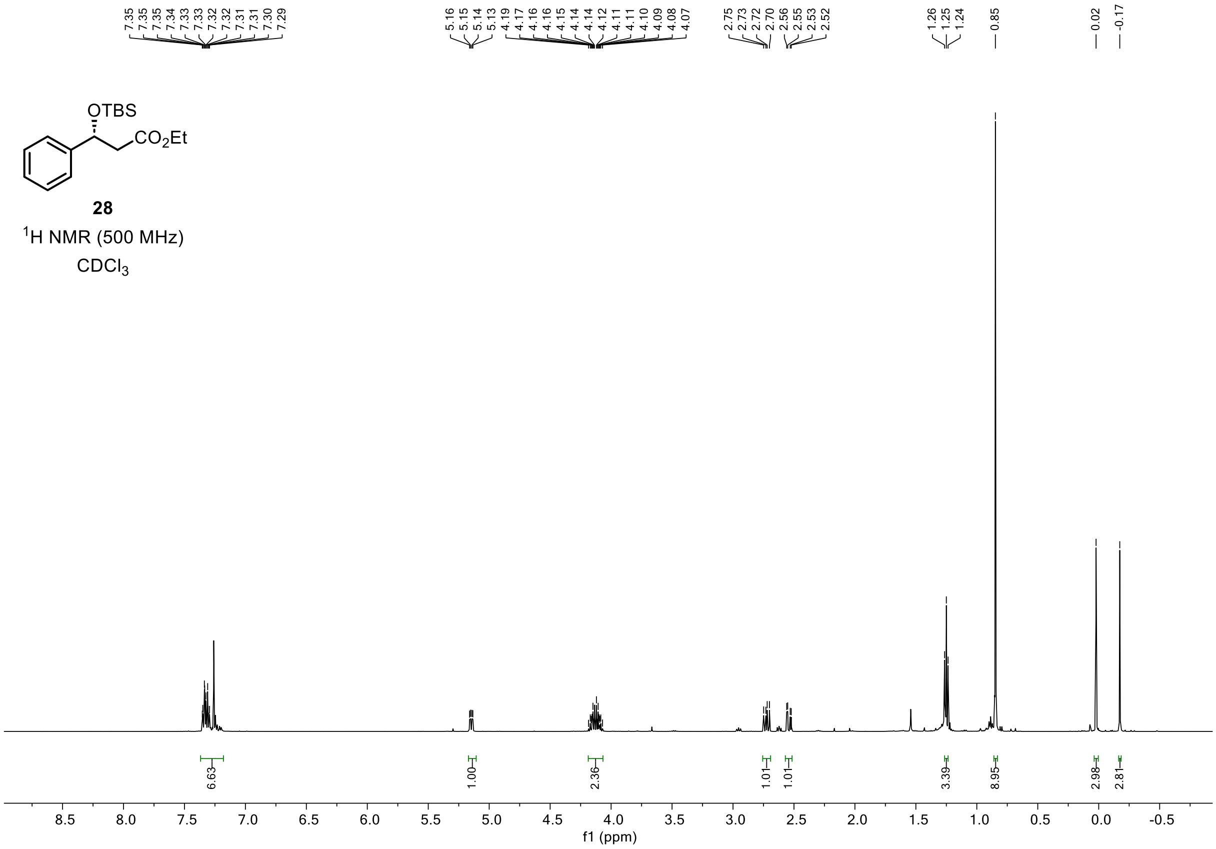
OTBS
CO_2Et
28
^{1}H NMR (500 MHz)
$CDCl_3$
7.35 7.35 7.35 7.34 7.33 7.33 7.32 7.32 7.31 7.31 7.30 7.29
5.16 5.15 5.14 5.13 4.19 4.17 4.16 4.16 4.15 4.14 4.14 4.12 4.11 4.11 4.10 4.09 4.08 4.07
2.75 2.73 2.72 2.70 2.56 2.55 2.53 2.52
1.26 1.25 1.24
0.85
0.02
-0.17
6.63 1.00 2.36 1.01 1.01 3.39 8.95 2.98 2.81
f1 (ppm)

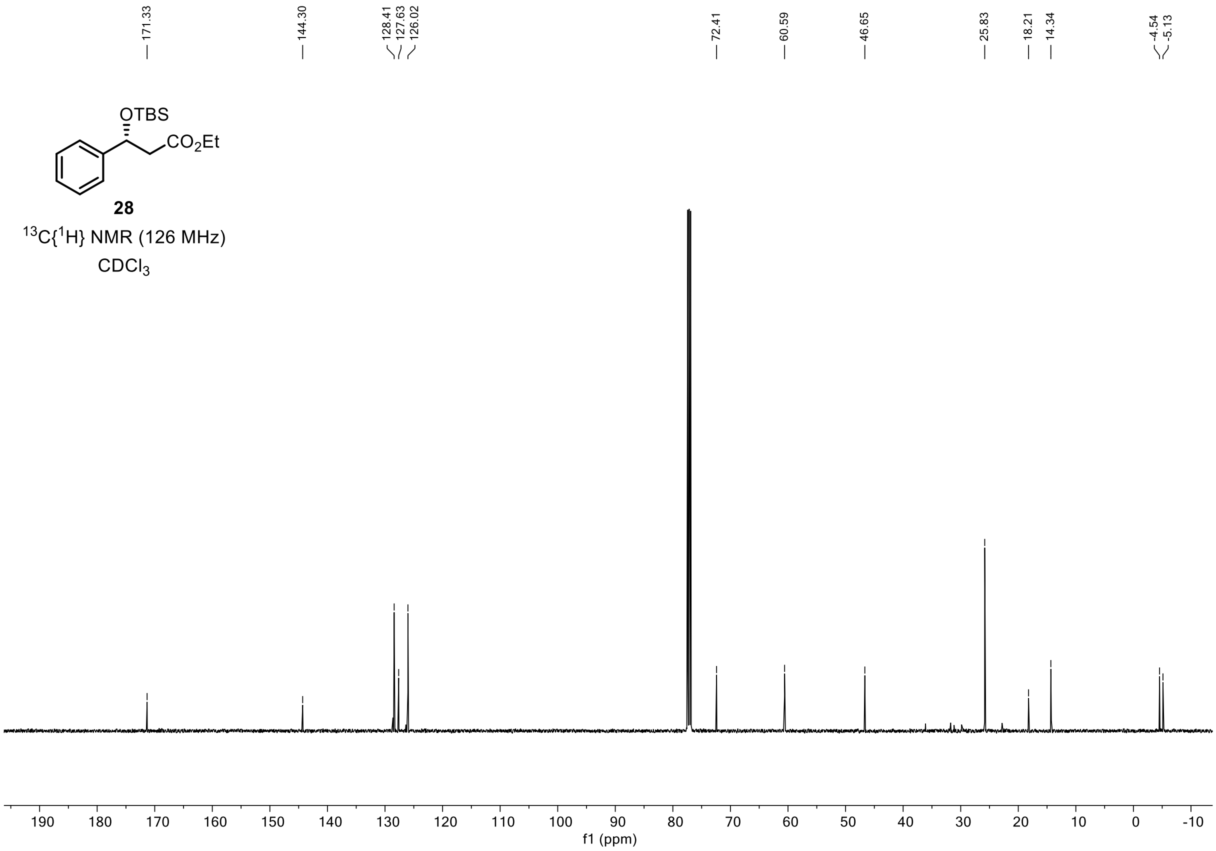
OTBS
CO_2Et
28
^{13}C{^{1}H} NMR (126 MHz)
$CDCl_3$
171.33
144.30
128.41 127.63 126.02
72.41
60.59
46.65
25.83
18.21
14.34
-4.54 -5.13
f1 (ppm)

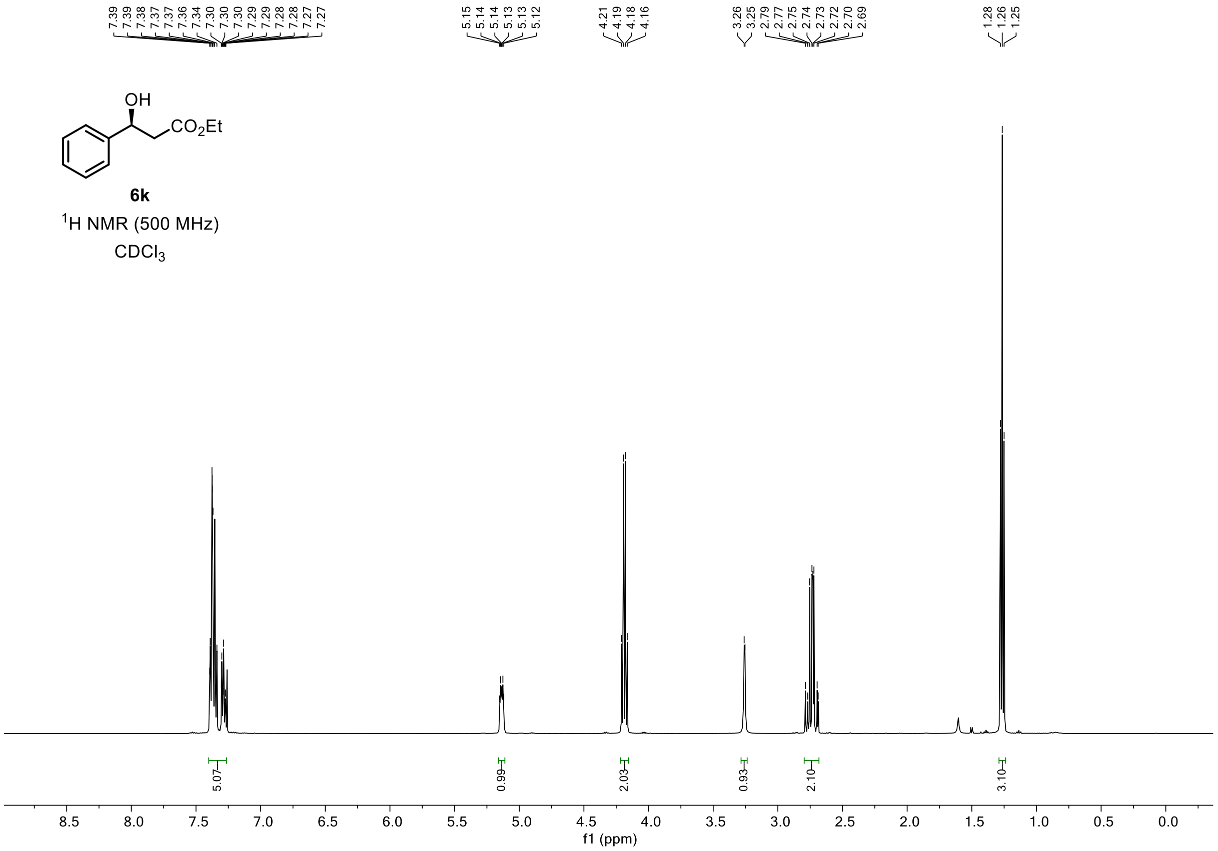
OH
CO2Et
6k
1H NMR (500 MHz)
CDCl3
7.39 7.39 7.38 7.37 7.37 7.36 7.34 7.30 7.30 7.30 7.29 7.29 7.28 7.28 7.27 7.27
5.15 5.14 5.14 5.13 5.13 5.12
4.21 4.19 4.18 4.16
3.26 3.25 2.79 2.77 2.75 2.74 2.73 2.72 2.70 2.69
1.28 1.26 1.25
5.07 0.99 2.03 0.93 2.10 3.10
f1 (ppm)

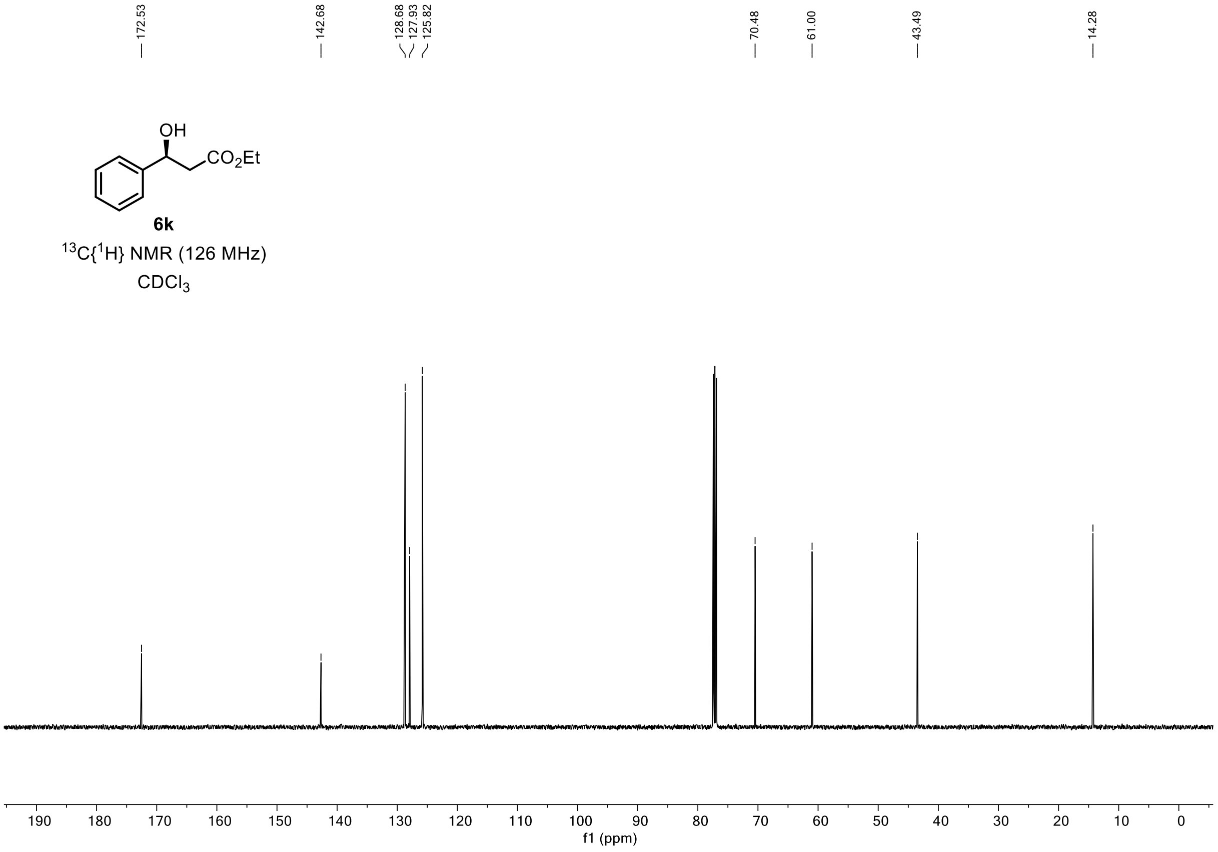
172.53 142.68 128.68 127.93 125.82 70.48 61.00 43.49 14.28
OH
CO2Et
6k
13C{1H} NMR (126 MHz)
CDCl3
f1 (ppm)

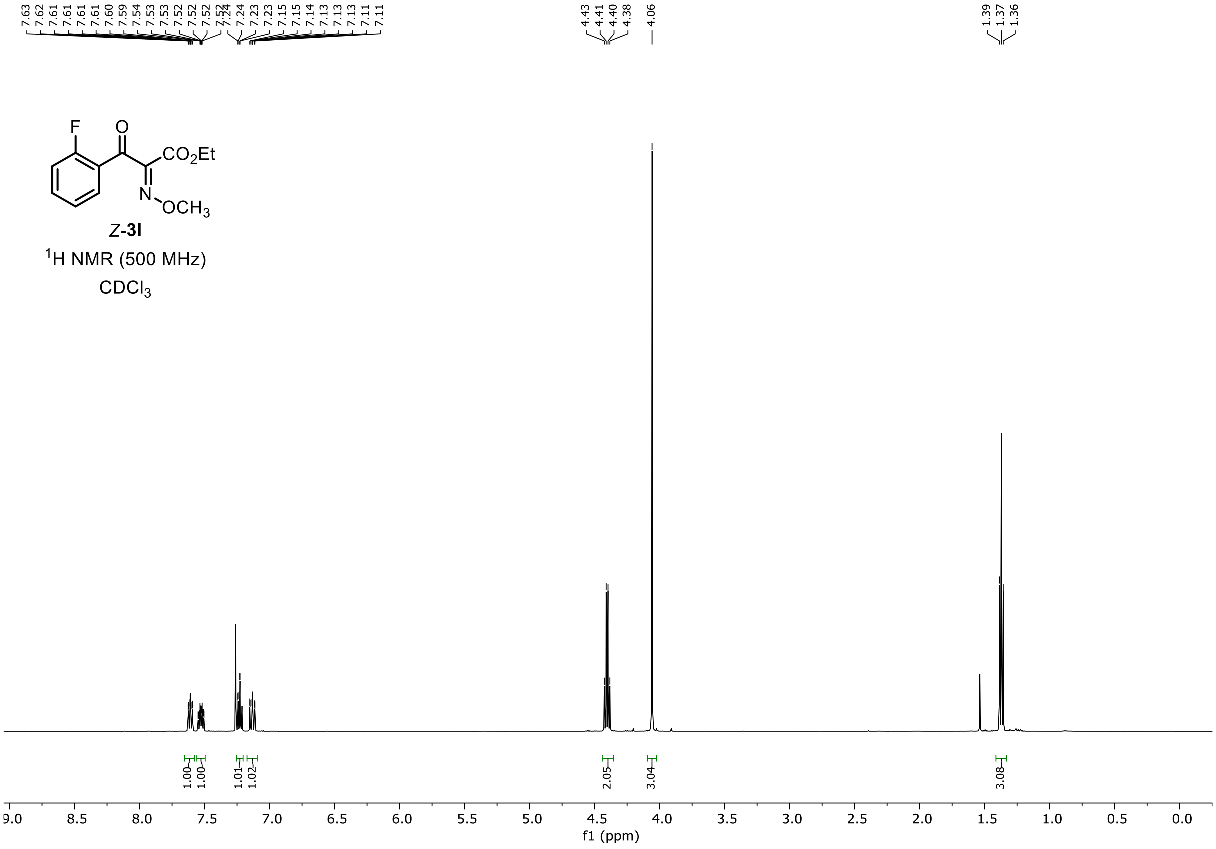

Z-**3l**
^{1}H NMR (500 MHz)
$CDCl_3$

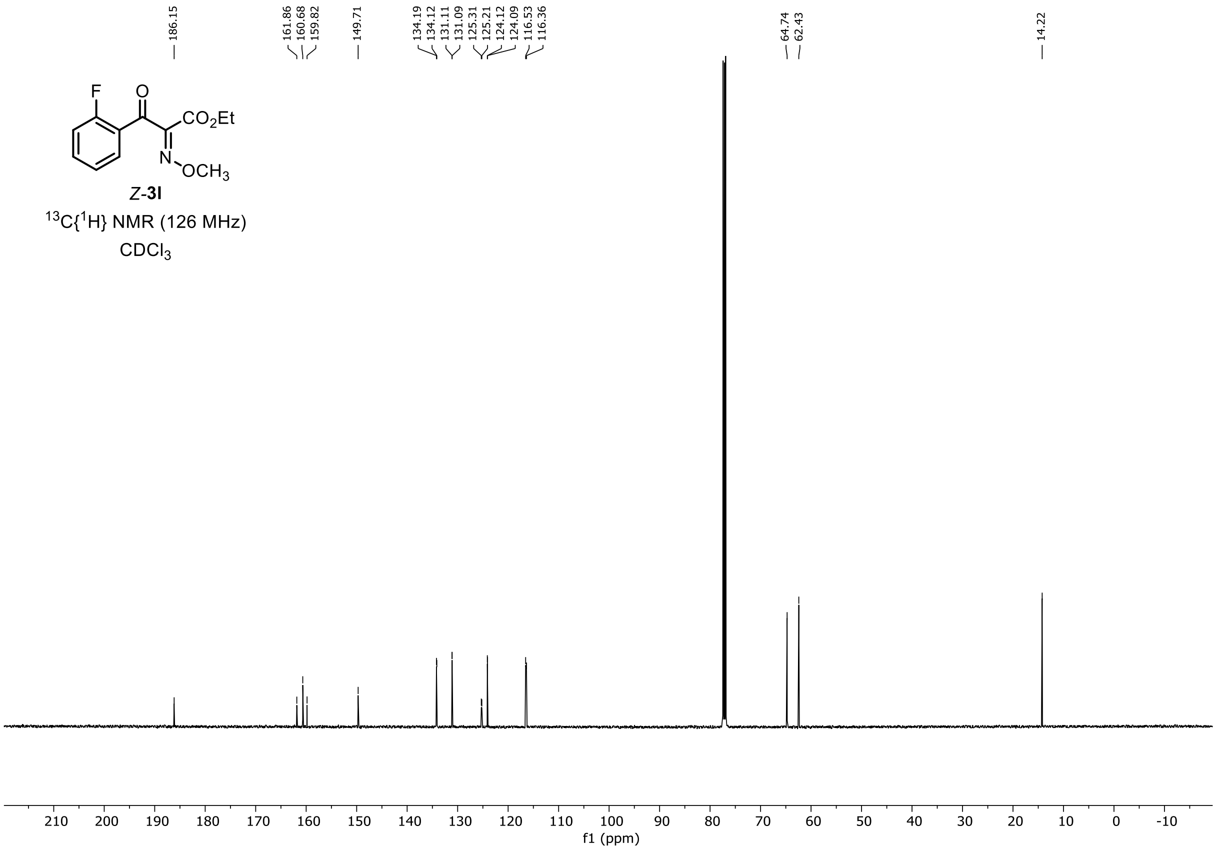

Z-**3l**
^{13}C{^{1}H} NMR (126 MHz)
$CDCl_3$

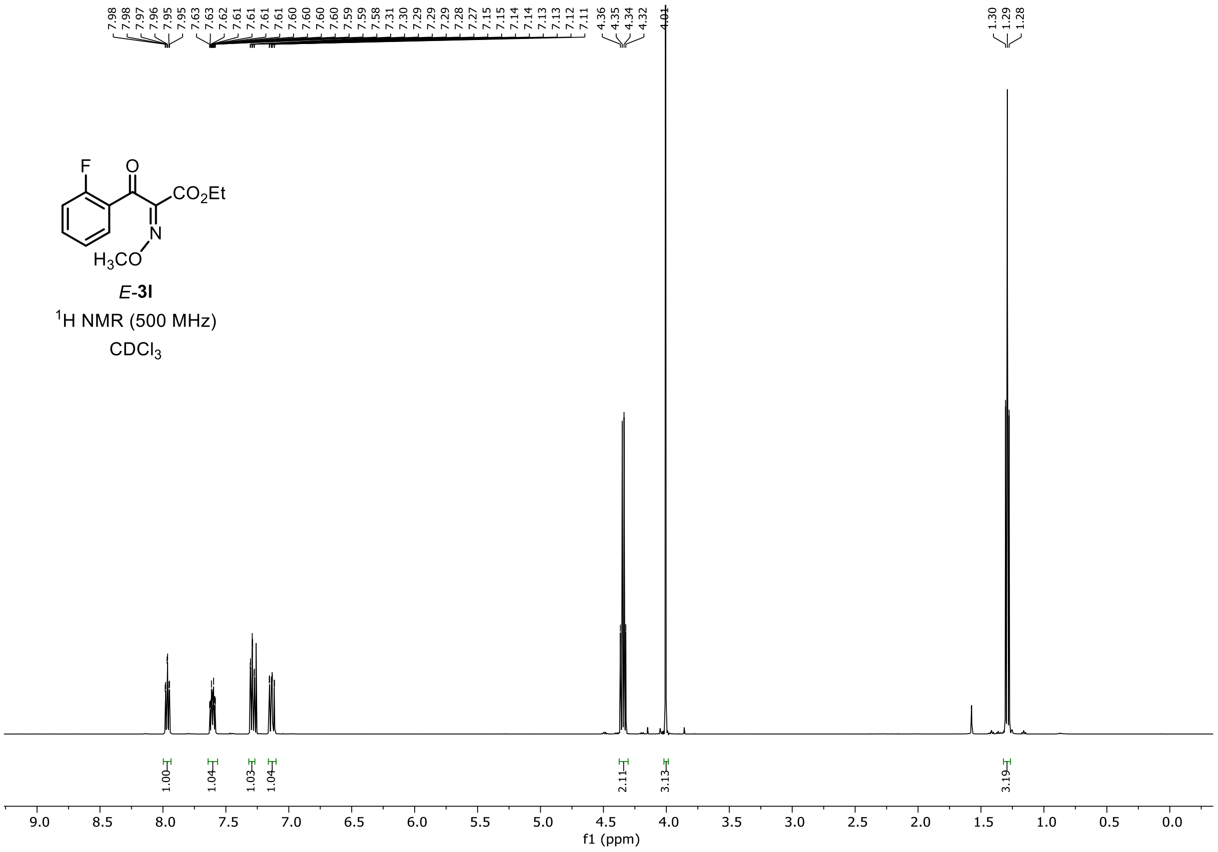
7.98 7.98 7.97 7.96 7.95 7.95 7.63 7.62 7.61 7.61 7.61 7.60 7.60 7.60 7.59 7.59 7.58 7.31 7.30 7.29 7.29 7.29 7.28 7.27 7.15 7.15 7.14 7.14 7.13 7.13 7.12 7.11 4.36 4.35 4.34 4.32 4.01
1.30 1.29 1.28
F
O
CO2Et
N
H3CO
E-3l
1H NMR (500 MHz)
CDCl3
1.00 1.04 1.03 1.04 2.11 3.13 3.19
9.0 8.5 8.0 7.5 7.0 6.5 6.0 5.5 5.0 4.5 4.0 3.5 3.0 2.5 2.0 1.5 1.0 0.5 0.0
f1 (ppm)

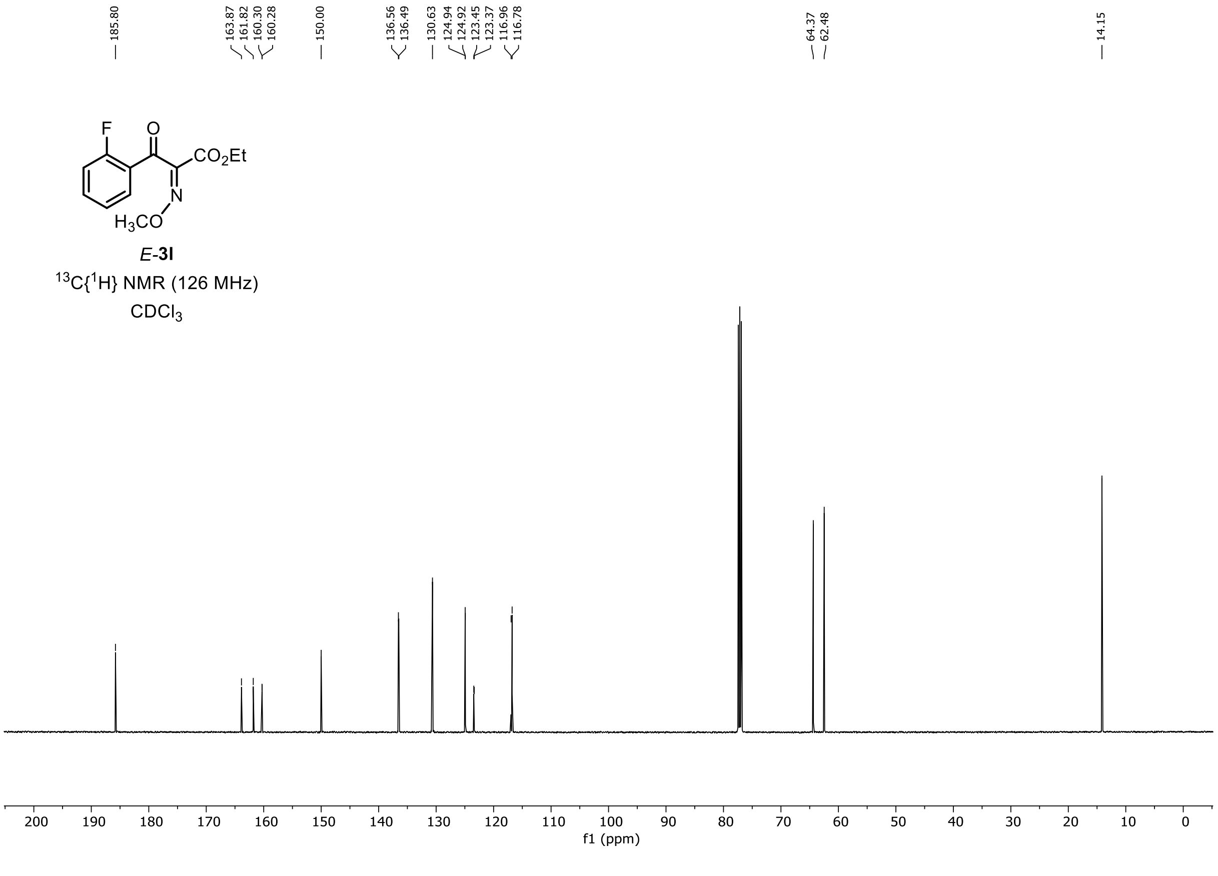
185.80
163.87 161.82 160.30 160.28
150.00
136.56 136.49
130.63 124.94 124.92 123.45 123.37 116.96 116.78
64.37 62.48
14.15
F
O
CO2Et
N
H3CO
E-3l
13C{1H} NMR (126 MHz)
CDCl3
200 190 180 170 160 150 140 130 120 110 100 90 80 70 60 50 40 30 20 10 0
f1 (ppm)

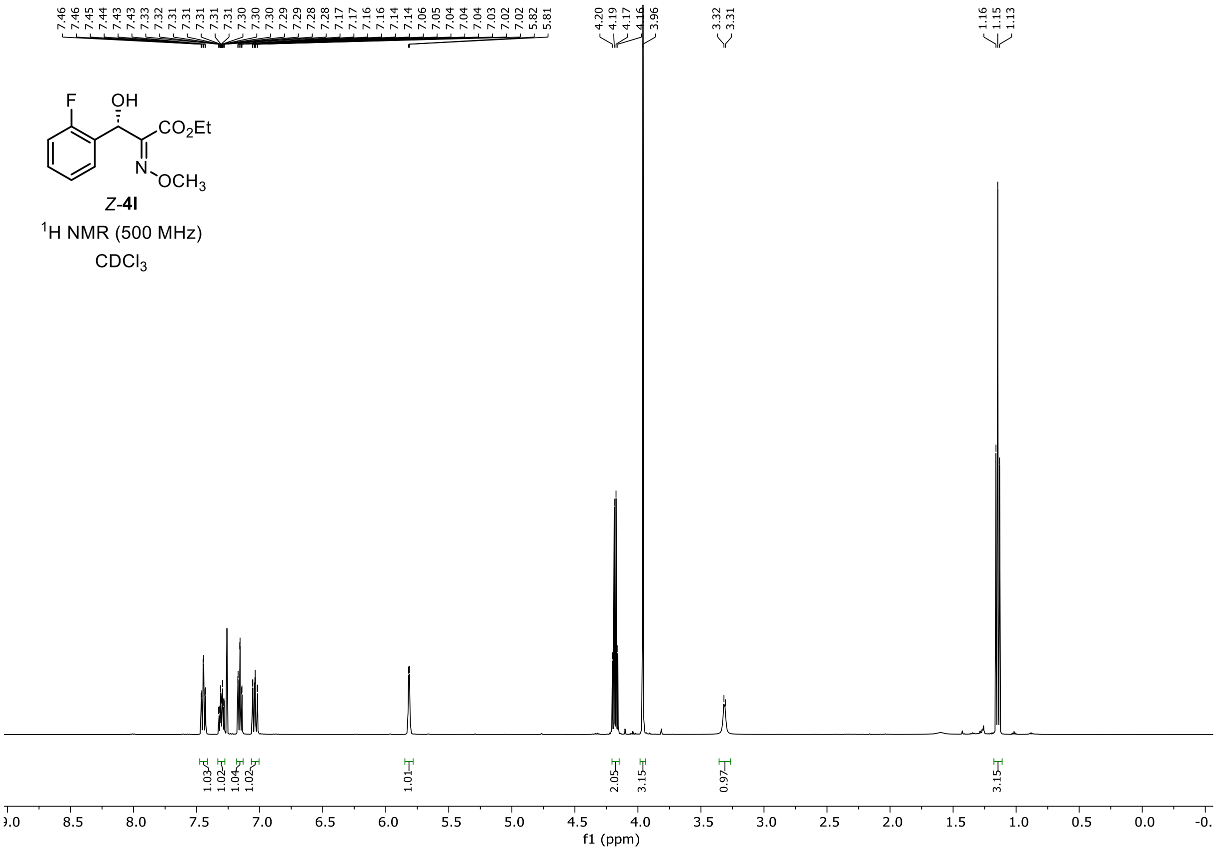
F
OH
CO2Et
N
OCH3
Z-4l
1H NMR (500 MHz)
CDCl3
f1 (ppm)

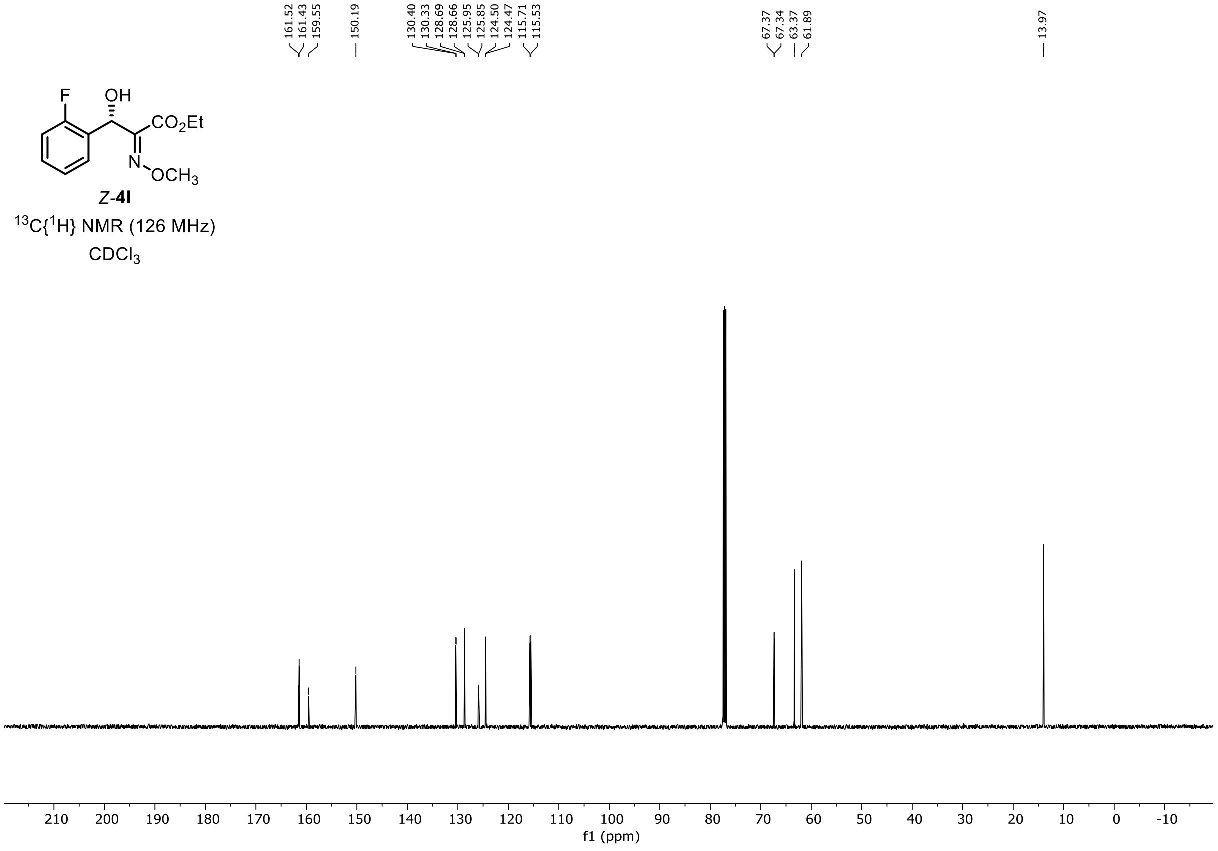
F
OH
CO2Et
N
OCH3
Z-4l
13C{1H} NMR (126 MHz)
CDCl3
f1 (ppm)

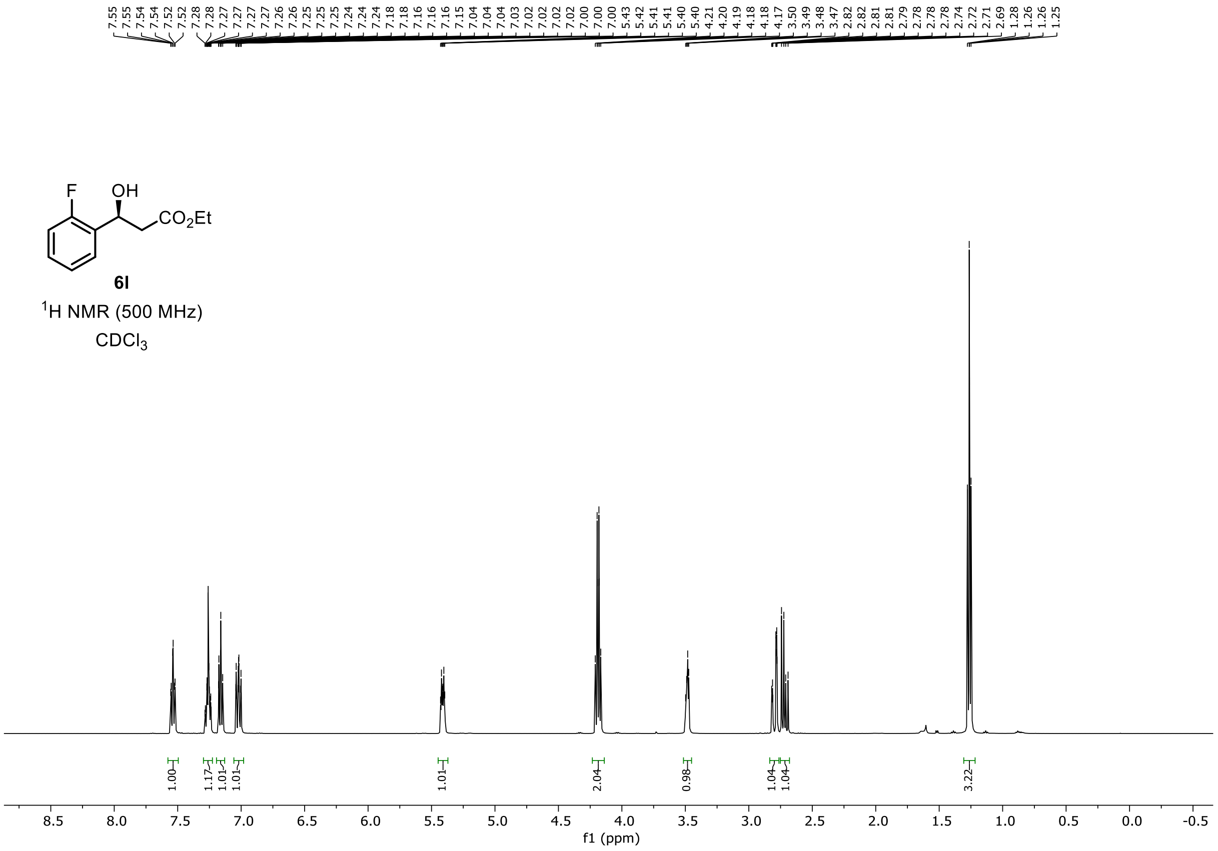
7.55 7.55 7.54 7.54 7.52 7.52 7.28 7.28 7.27 7.27 7.27 7.27 7.26 7.26 7.25 7.25 7.25 7.24 7.24 7.24 7.18 7.18 7.16 7.16 7.16 7.15 7.04 7.04 7.04 7.03 7.02 7.02 7.02 7.02 7.00 7.00 7.00 5.43 5.42 5.41 5.41 5.40 5.40 4.21 4.20 4.19 4.18 4.18 4.17 3.50 3.49 3.48 3.47 2.82 2.82 2.81 2.81 2.79 2.78 2.78 2.78 2.74 2.72 2.71 2.69 1.28 1.26 1.26 1.25
F
OH
CO2Et
6l
1H NMR (500 MHz)
CDCl3
1.00 1.17 1.01 1.01 1.01 2.04 0.98 1.04 1.04 3.22
f1 (ppm)

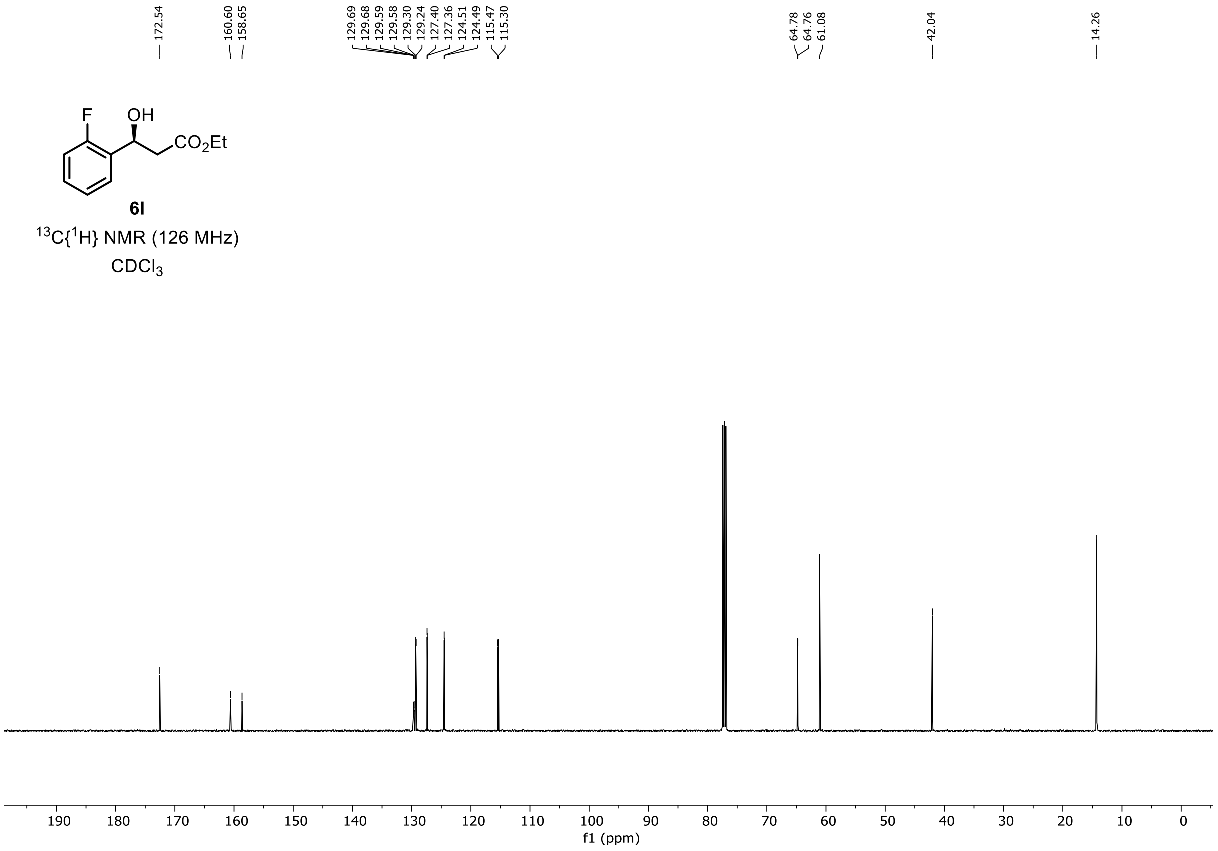
172.54
160.60 158.65
129.69 129.68 129.59 129.58 129.30 129.24 127.40 127.36 124.51 124.49 115.47 115.30
64.78 64.76 61.08
42.04
14.26
F
OH
CO2Et
6l
13C{1H} NMR (126 MHz)
CDCl3
f1 (ppm)

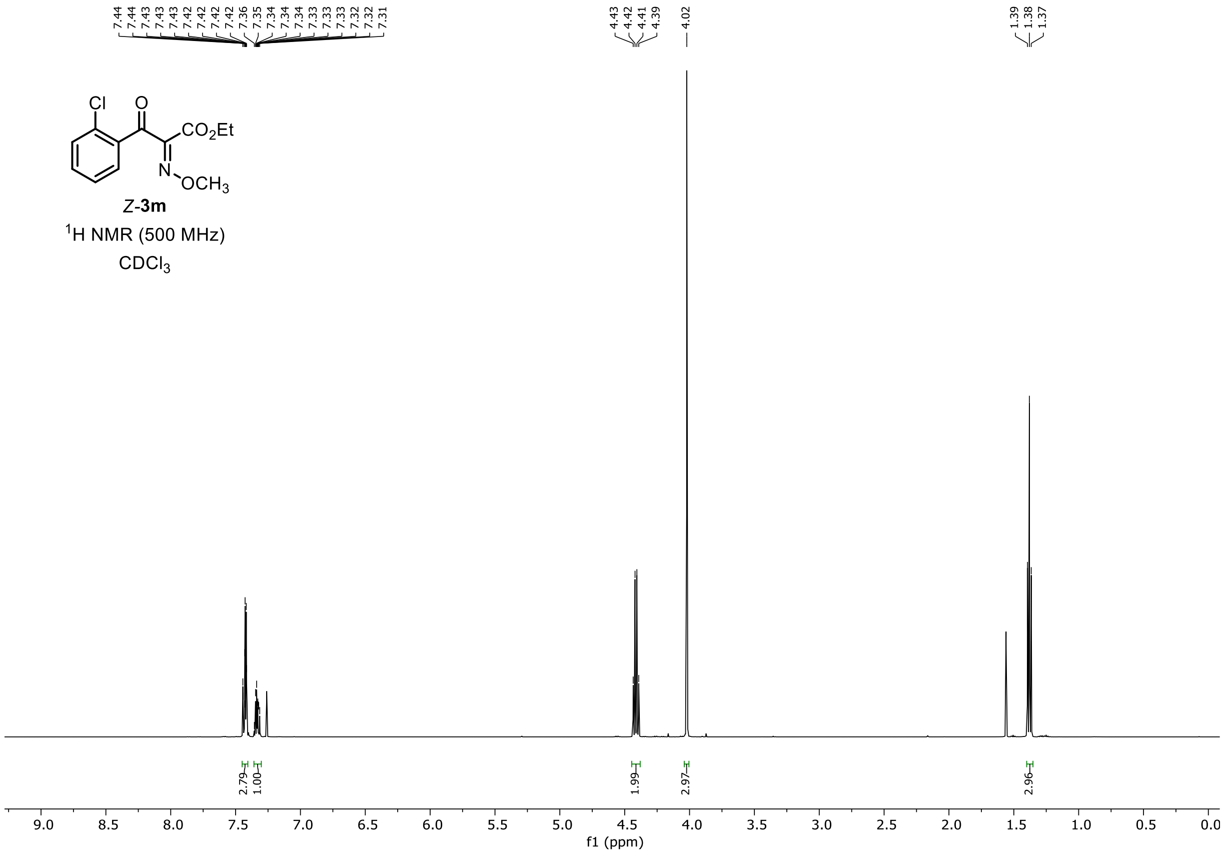

Cl
O
CO2Et
N
OCH3
Z-3m
1H NMR (500 MHz)
CDCl3
7.44
7.44
7.43
7.43
7.42
7.42
7.42
7.42
7.36
7.35
7.34
7.34
7.34
7.33
7.33
7.33
7.32
7.32
7.31
4.43
4.42
4.41
4.39
4.02
1.39
1.38
1.37
2.79
1.00
1.99
2.97
2.96
f1 (ppm)

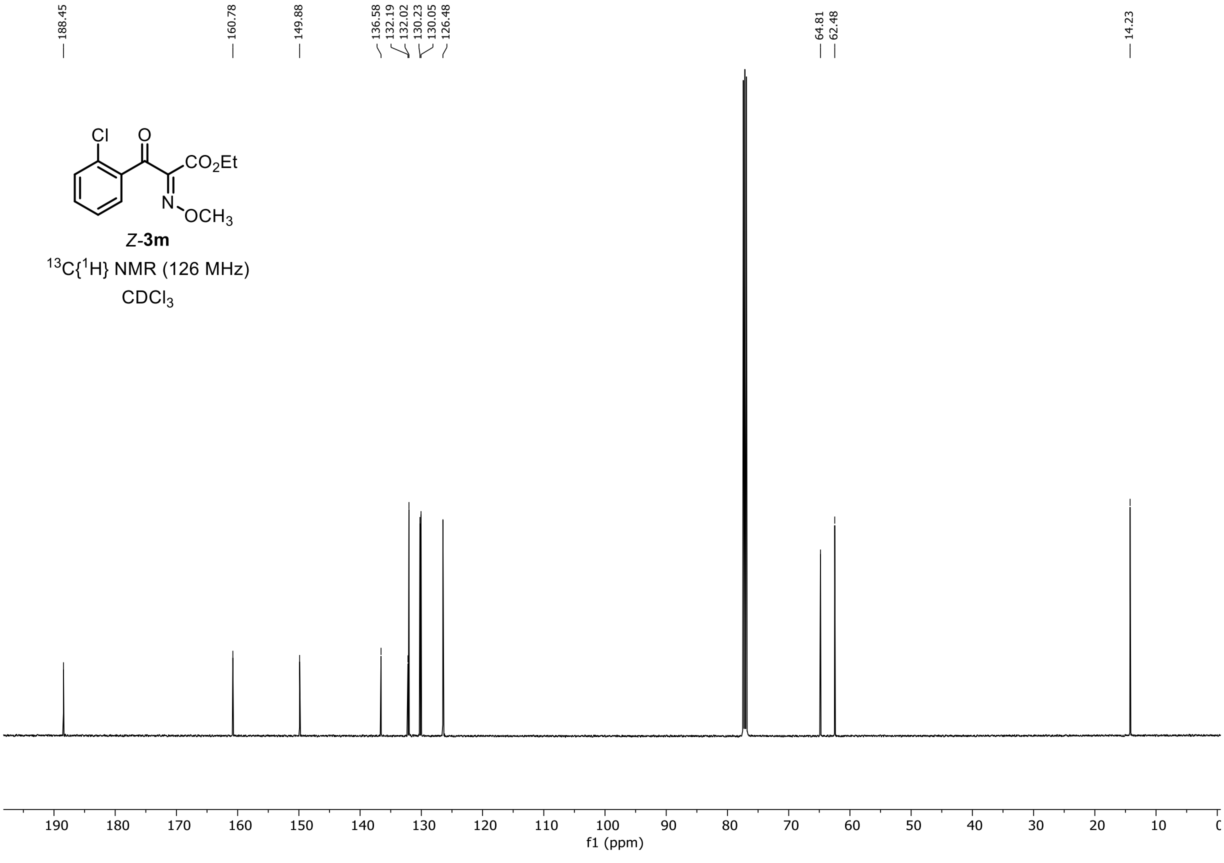

Cl
O
CO2Et
N
OCH3
Z-3m
13C{1H} NMR (126 MHz)
CDCl3
188.45
160.78
149.88
136.58
132.19
132.02
130.23
130.05
126.48
64.81
62.48
14.23
f1 (ppm)

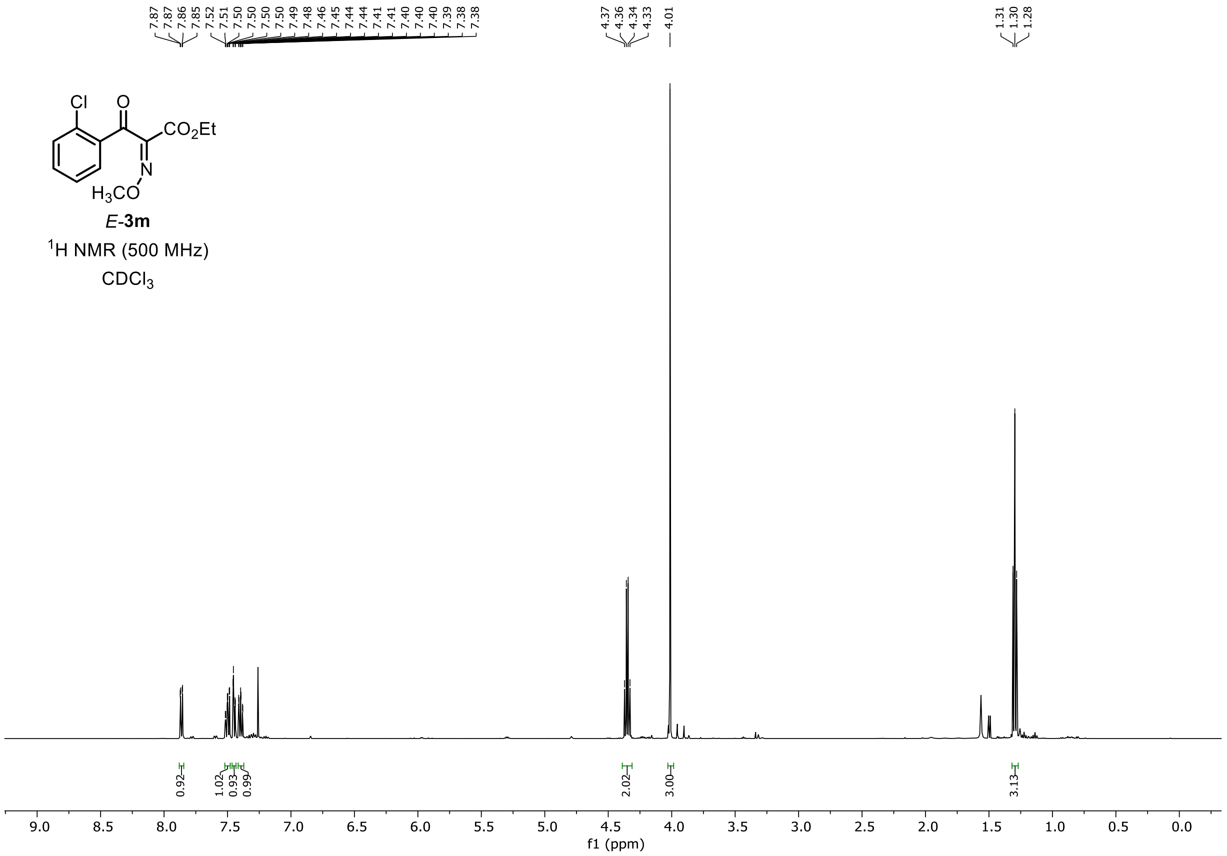
7.87
7.87
7.86
7.85
7.52
7.51
7.50
7.50
7.50
7.50
7.49
7.48
7.46
7.45
7.44
7.44
7.41
7.41
7.40
7.40
7.40
7.39
7.38
7.38
4.37
4.36
4.34
4.33
4.01
1.31
1.30
1.28
Cl
O
CO_2Et
N
H_3CO
E-**3m**
^{1}H NMR (500 MHz)
$CDCl_3$
0.92
1.02
0.93
0.99
2.02
3.00
3.13
9.0
8.5
8.0
7.5
7.0
6.5
6.0
5.5
5.0
4.5
4.0
3.5
3.0
2.5
2.0
1.5
1.0
0.5
0.0
f1 (ppm)

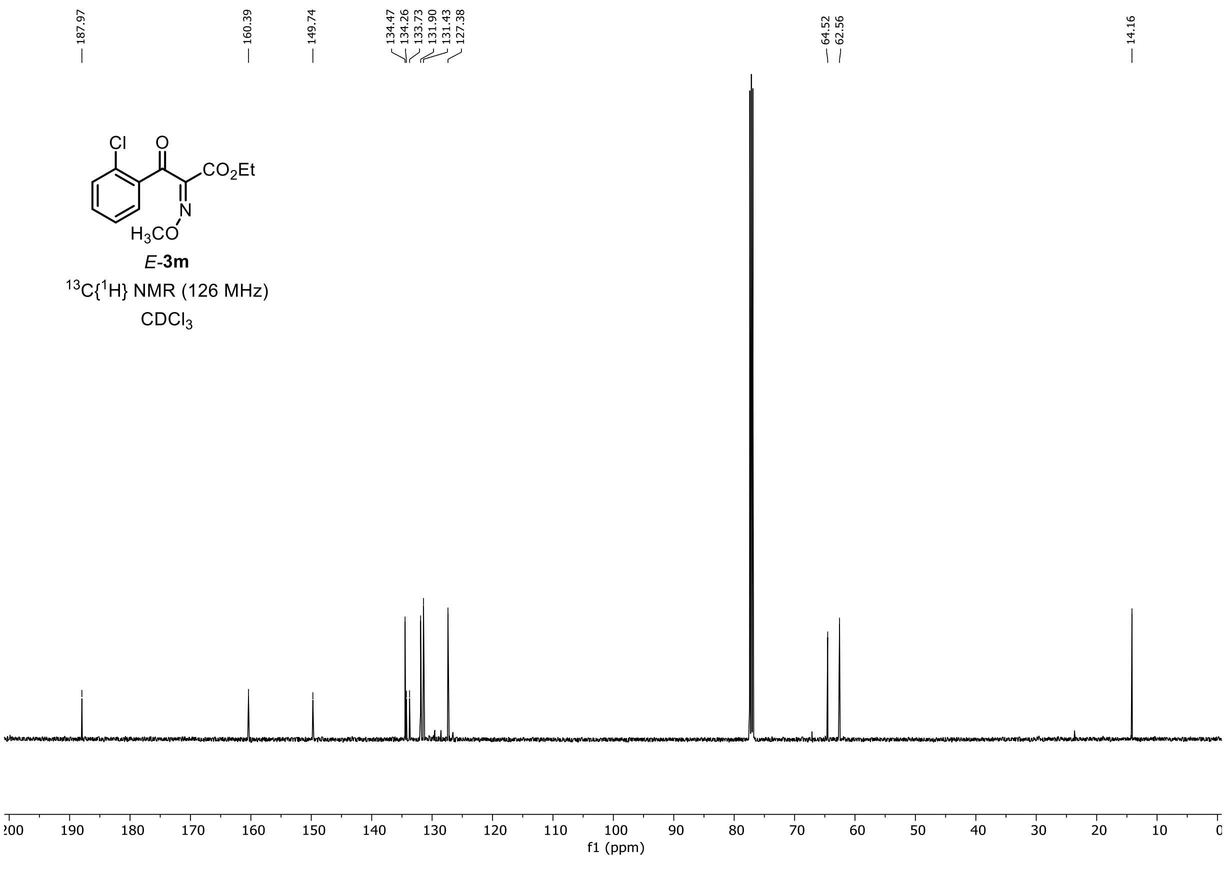
187.97
160.39
149.74
134.47
134.26
133.73
131.90
131.43
127.38
64.52
62.56
14.16
Cl
O
CO_2Et
N
H_3CO
E-**3m**
$^{13}C\{^1H\}$ NMR (126 MHz)
$CDCl_3$
200
190
180
170
160
150
140
130
120
110
100
90
80
70
60
50
40
30
20
10
0
f1 (ppm)

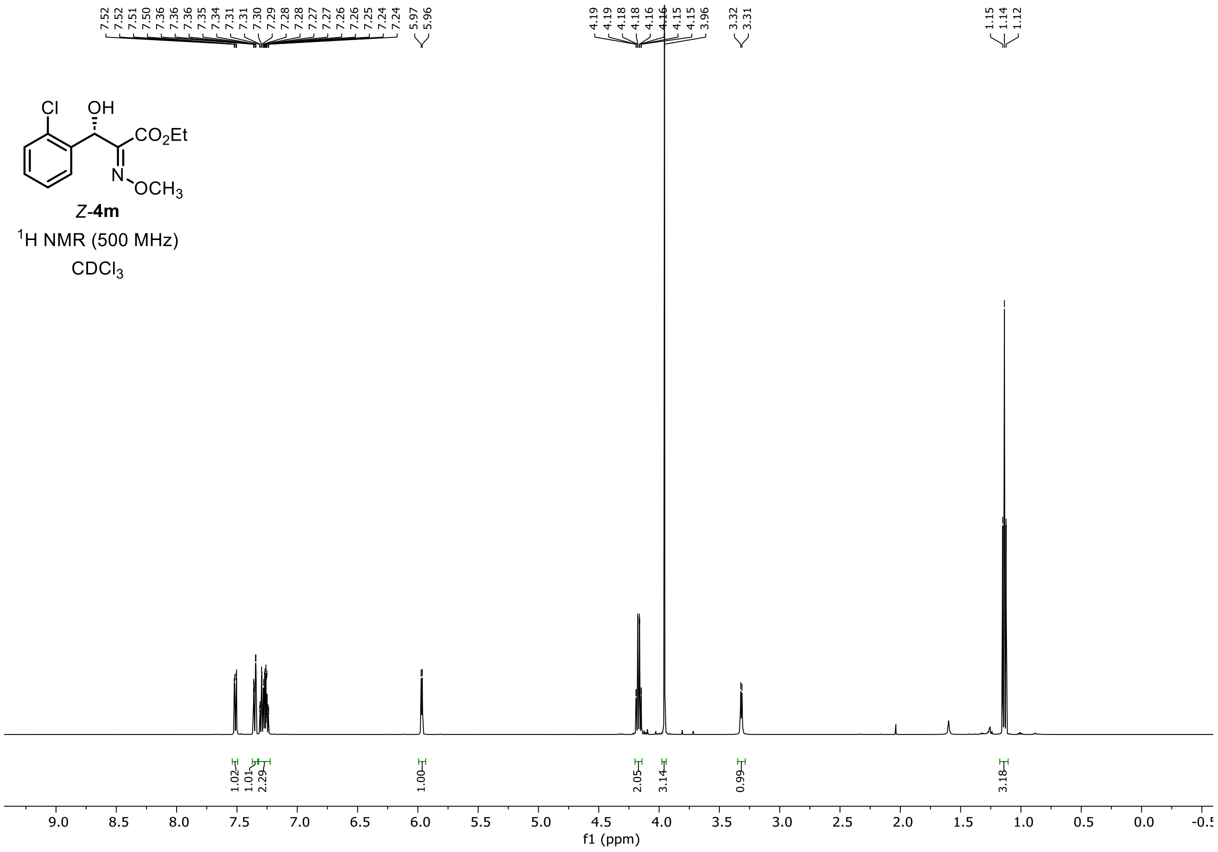
7.52 7.52 7.51 7.50 7.36 7.36 7.36 7.35 7.34 7.31 7.31 7.30 7.29 7.28 7.28 7.27 7.27 7.26 7.26 7.25 7.24 7.24
5.97 5.96
4.19 4.19 4.18 4.18 4.16 4.16 4.15 4.15 3.96
3.32 3.31
1.15 1.14 1.12
Cl
OH
CO2Et
N
OCH3
Z-4m
1H NMR (500 MHz)
CDCl3
1.02
1.01
2.29
1.00
2.05
3.14
0.99
3.18
f1 (ppm)

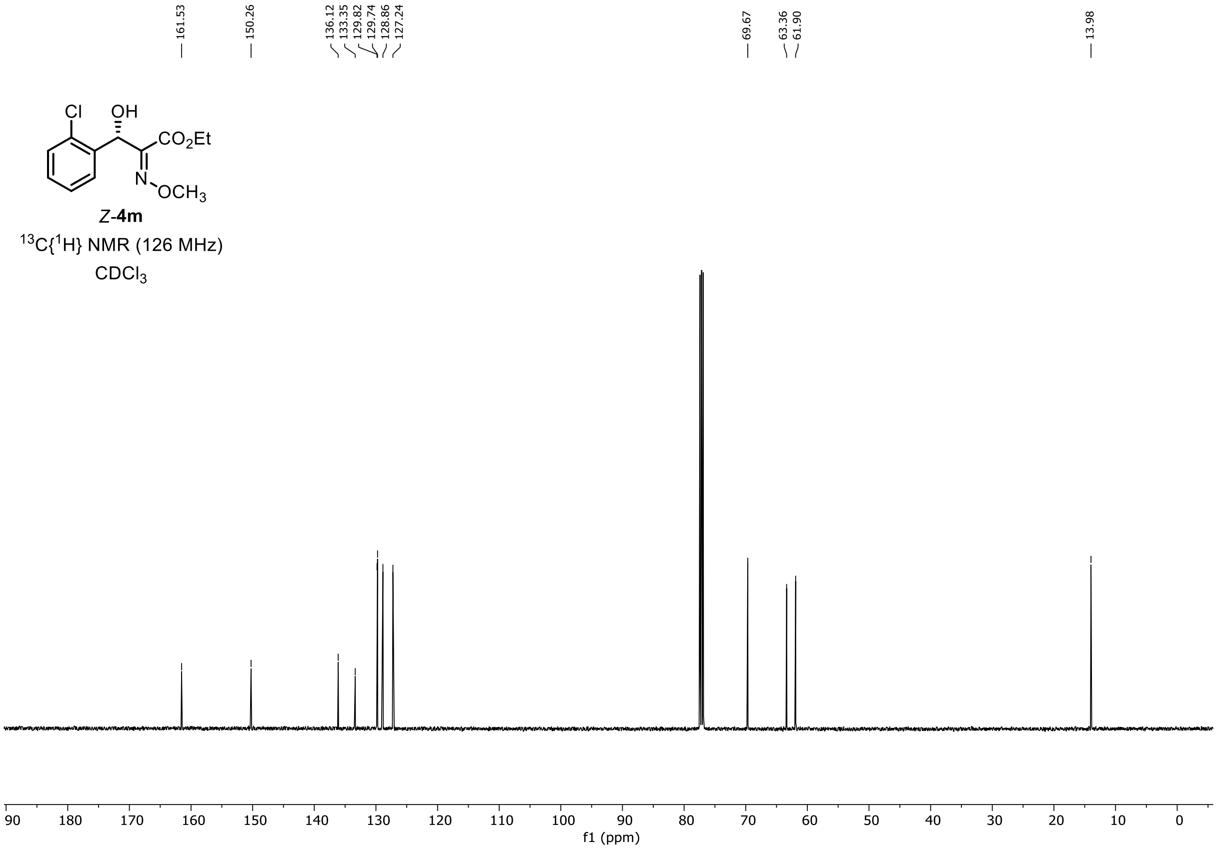
161.53
150.26
136.12 133.35 129.82 129.74 128.86 127.24
69.67
63.36 61.90
13.98
Cl
OH
CO2Et
N
OCH3
Z-4m
13C{1H} NMR (126 MHz)
CDCl3
f1 (ppm)

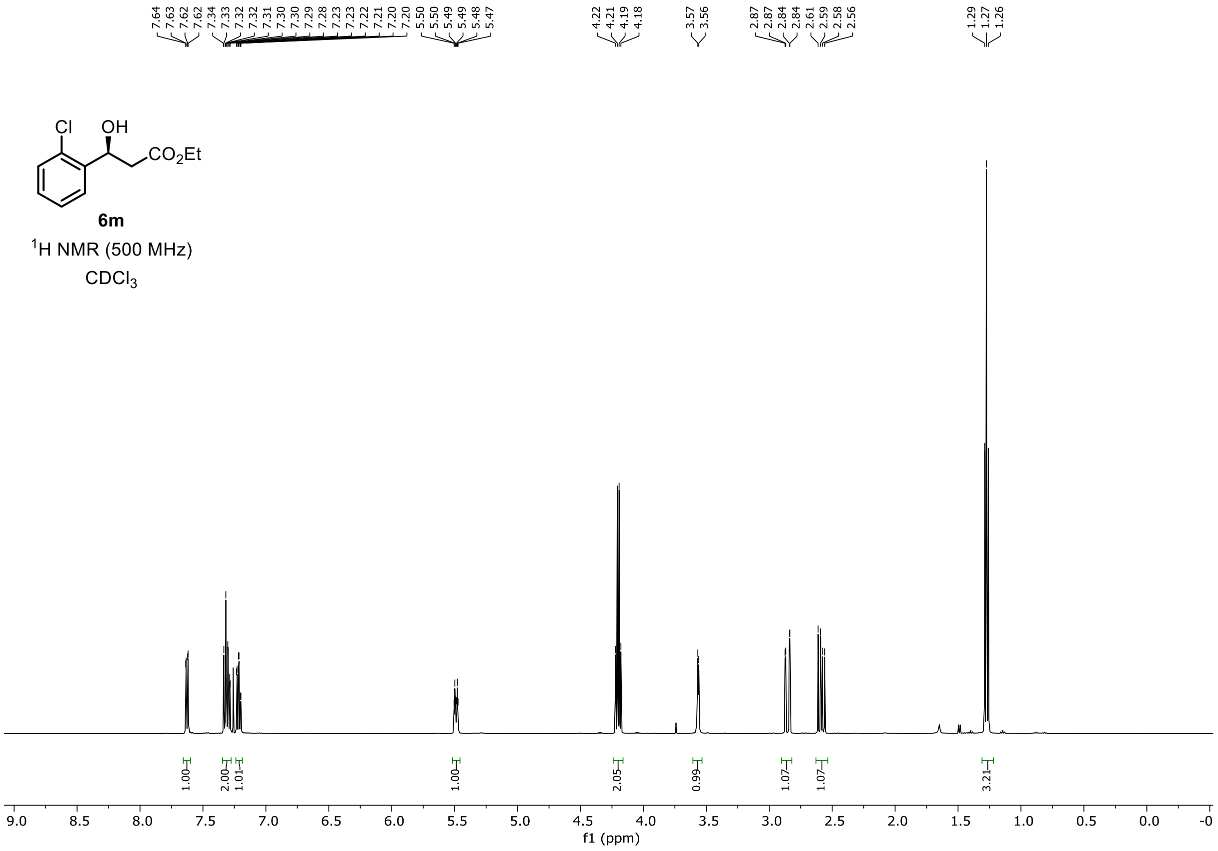
7.64 7.63 7.62 7.62 7.34 7.33 7.32 7.32 7.31 7.30 7.30 7.29 7.28 7.23 7.23 7.23 7.22 7.21 7.20 7.20 5.50 5.50 5.49 5.49 5.48 5.47
4.22 4.21 4.19 4.18
3.57 3.56
2.87 2.87 2.84 2.84 2.61 2.59 2.58 2.56
1.29 1.27 1.26
Cl
OH
CO2Et
6m
1H NMR (500 MHz)
CDCl3
1.00 2.00 1.01 1.00 2.05 0.99 1.07 1.07 3.21
f1 (ppm)

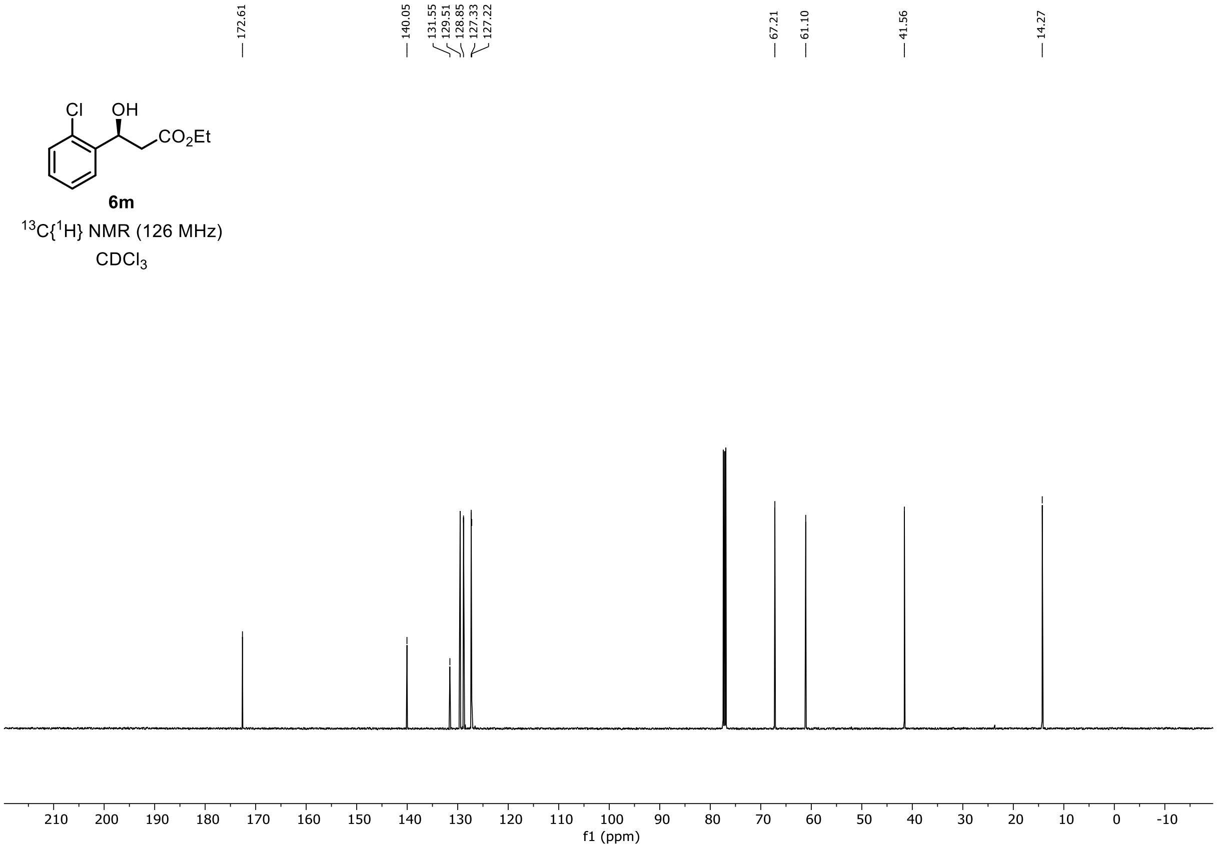
172.61
140.05 131.55 129.51 128.85 127.33 127.22
67.21 61.10
41.56
14.27
Cl
OH
CO2Et
6m
13C{1H} NMR (126 MHz)
CDCl3
f1 (ppm)

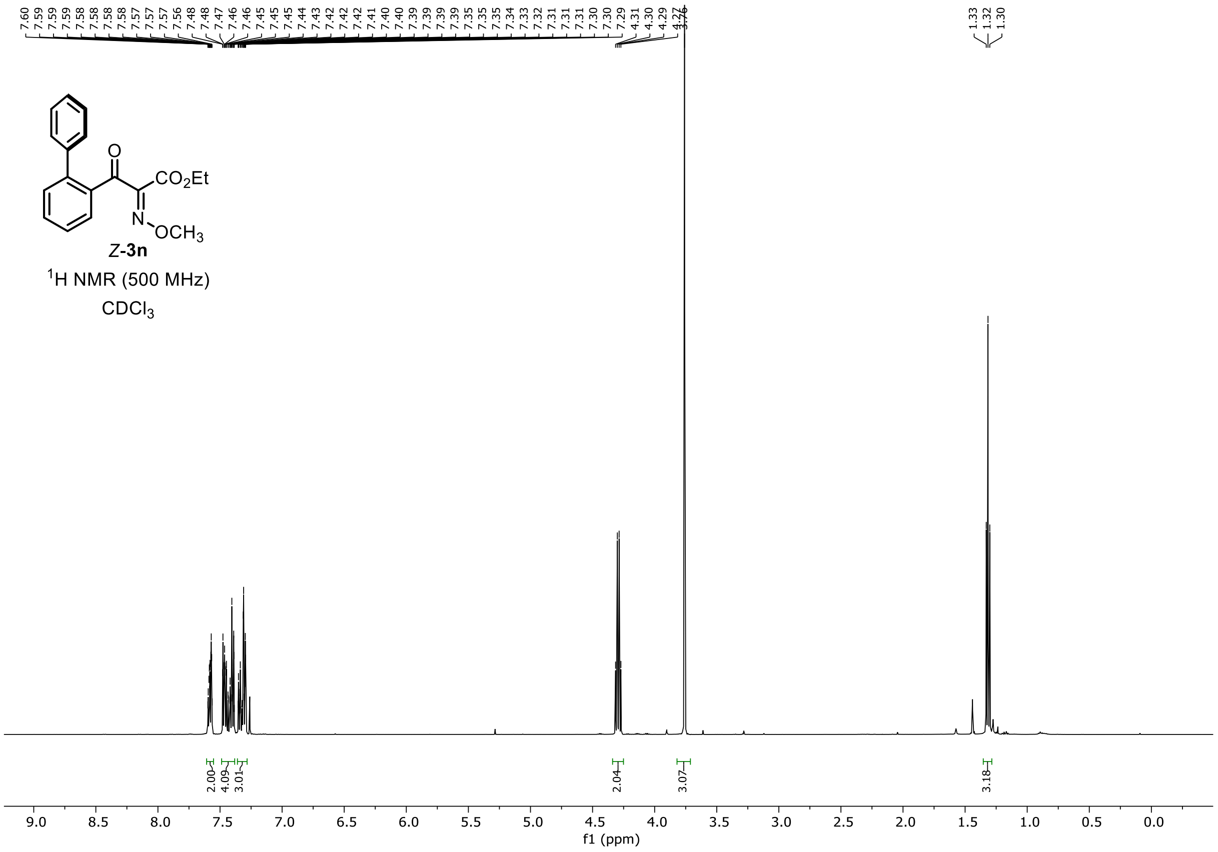
7.60 7.59 7.59 7.59 7.58 7.58 7.58 7.58 7.57 7.57 7.57 7.56 7.48 7.47 7.46 7.46 7.45 7.45 7.45 7.44 7.43 7.42 7.42 7.42 7.41 7.40 7.40 7.39 7.39 7.39 7.39 7.35 7.35 7.35 7.34 7.33 7.32 7.31 7.31 7.30 7.29 4.31 4.30 4.29 4.27 3.76
1.33 1.32 1.30
CO2Et
OCH3
Z-3n
1H NMR (500 MHz)
CDCl3
2.00 4.09 3.01 2.04 3.07 3.18
f1 (ppm)

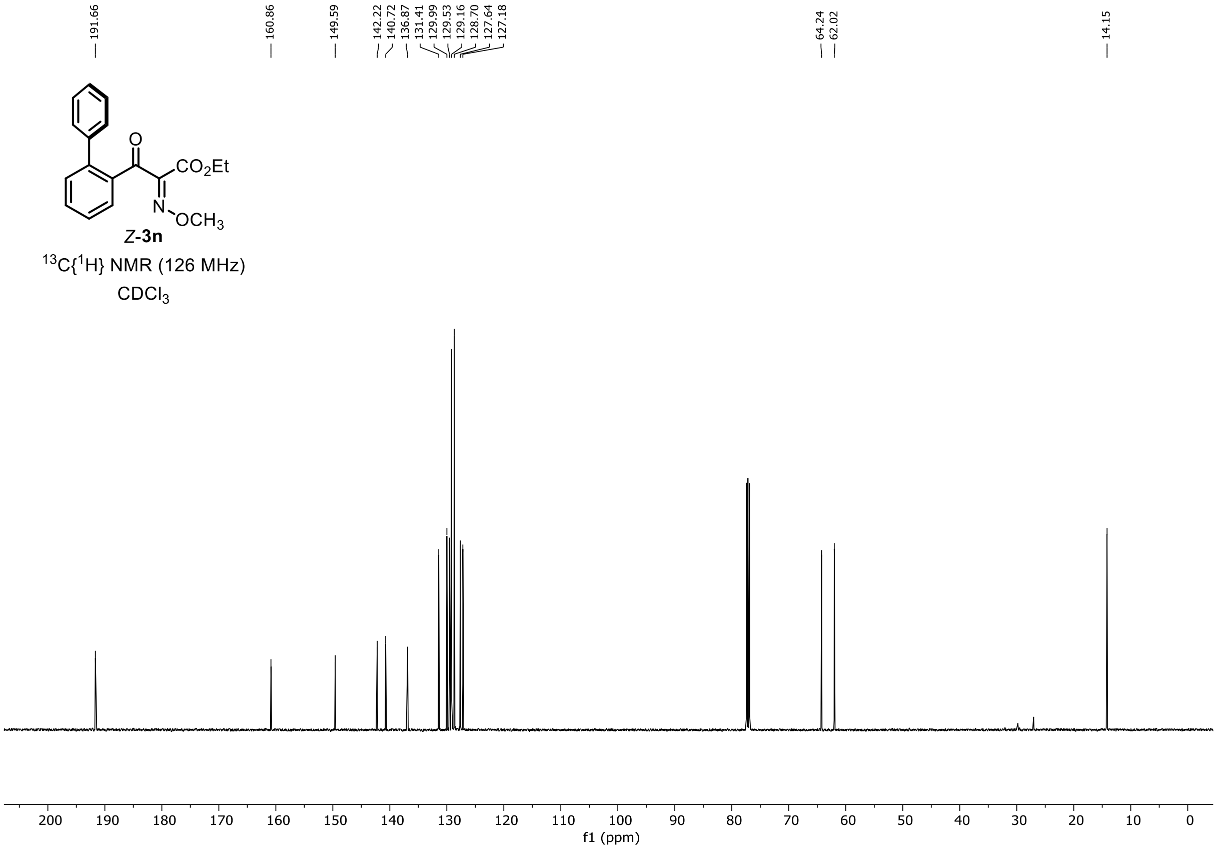
191.66
160.86
149.59
142.22 140.72 136.87 131.41 129.99 129.53 129.16 128.70 127.64 127.18
64.24 62.02
14.15
CO2Et
OCH3
Z-3n
13C{1H} NMR (126 MHz)
CDCl3
f1 (ppm)

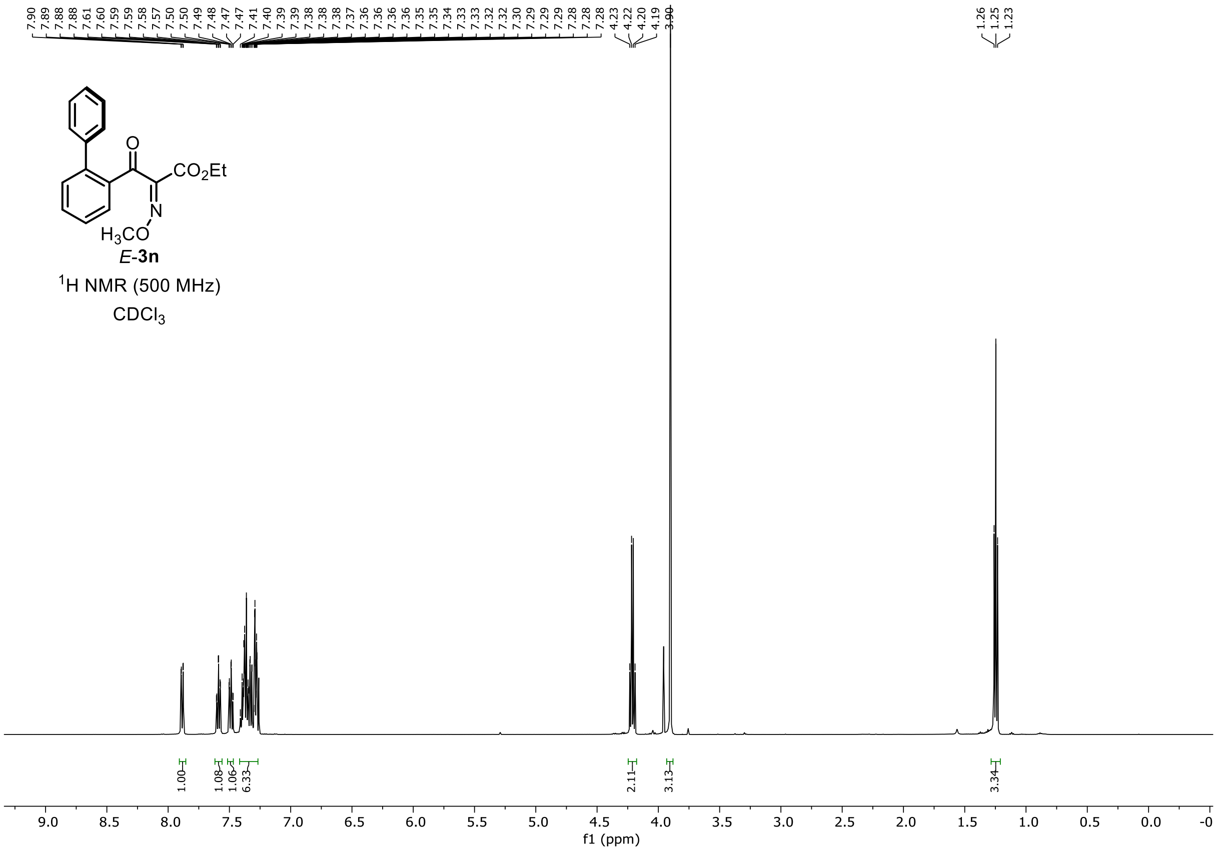
7.90 7.89 7.88 7.88 7.61 7.60 7.59 7.59 7.58 7.57 7.50 7.49 7.48 7.47 7.47 7.41 7.40 7.39 7.39 7.38 7.38 7.38 7.37 7.36 7.36 7.36 7.35 7.35 7.34 7.33 7.33 7.32 7.32 7.30 7.29 7.29 7.29 7.28 7.28 7.28 4.23 4.22 4.20 4.19 3.90
1.26 1.25 1.23
O
CO2Et
N
H3CO
E-3n
1H NMR (500 MHz)
CDCl3
1.00 1.08 1.06 6.33 2.11 3.13 3.34
9.0 8.5 8.0 7.5 7.0 6.5 6.0 5.5 5.0 4.5 4.0 3.5 3.0 2.5 2.0 1.5 1.0 0.5 0.0 -0
f1 (ppm)

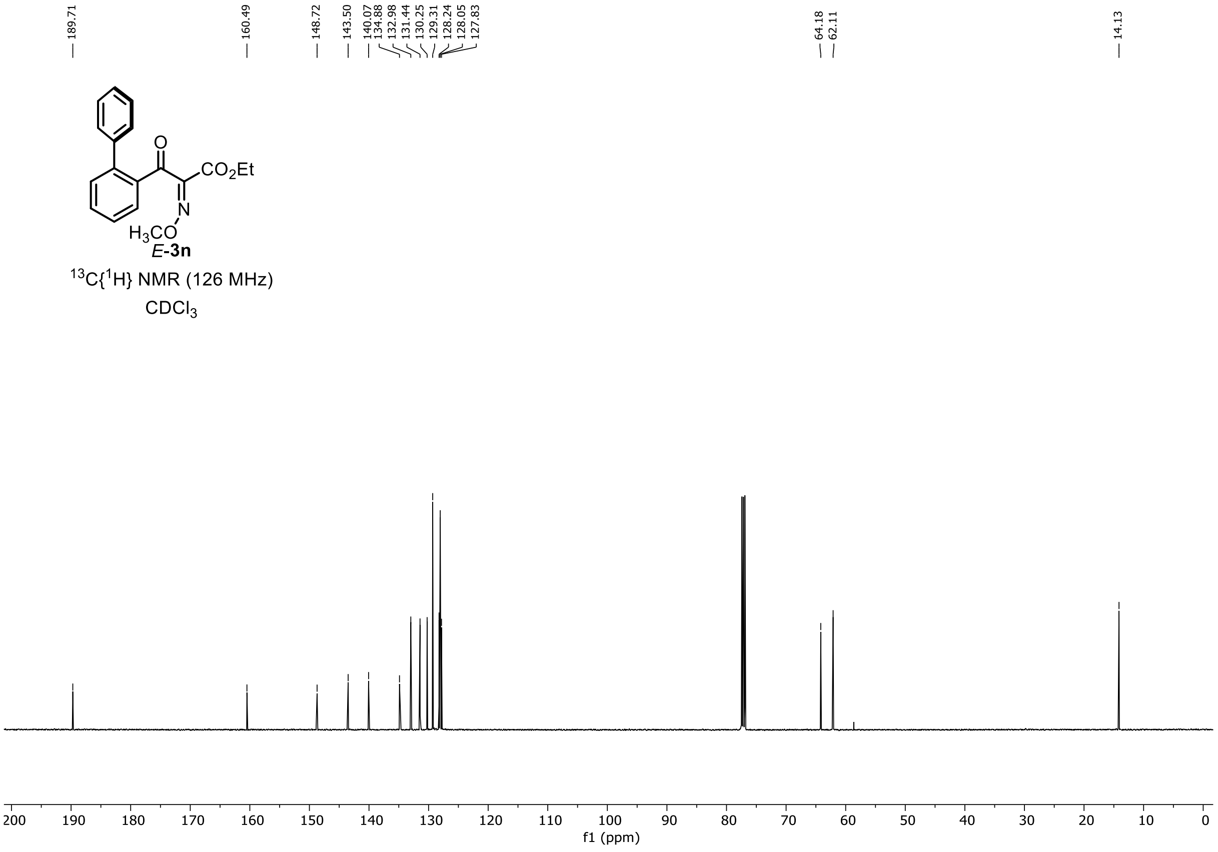
189.71
160.49
148.72
143.50
140.07 134.88 132.98 131.44 130.25 129.31 128.24 128.05 127.83
64.18 62.11
14.13
O
CO2Et
N
H3CO
E-3n
13C{1H} NMR (126 MHz)
CDCl3
200 190 180 170 160 150 140 130 120 110 100 90 80 70 60 50 40 30 20 10 0
f1 (ppm)

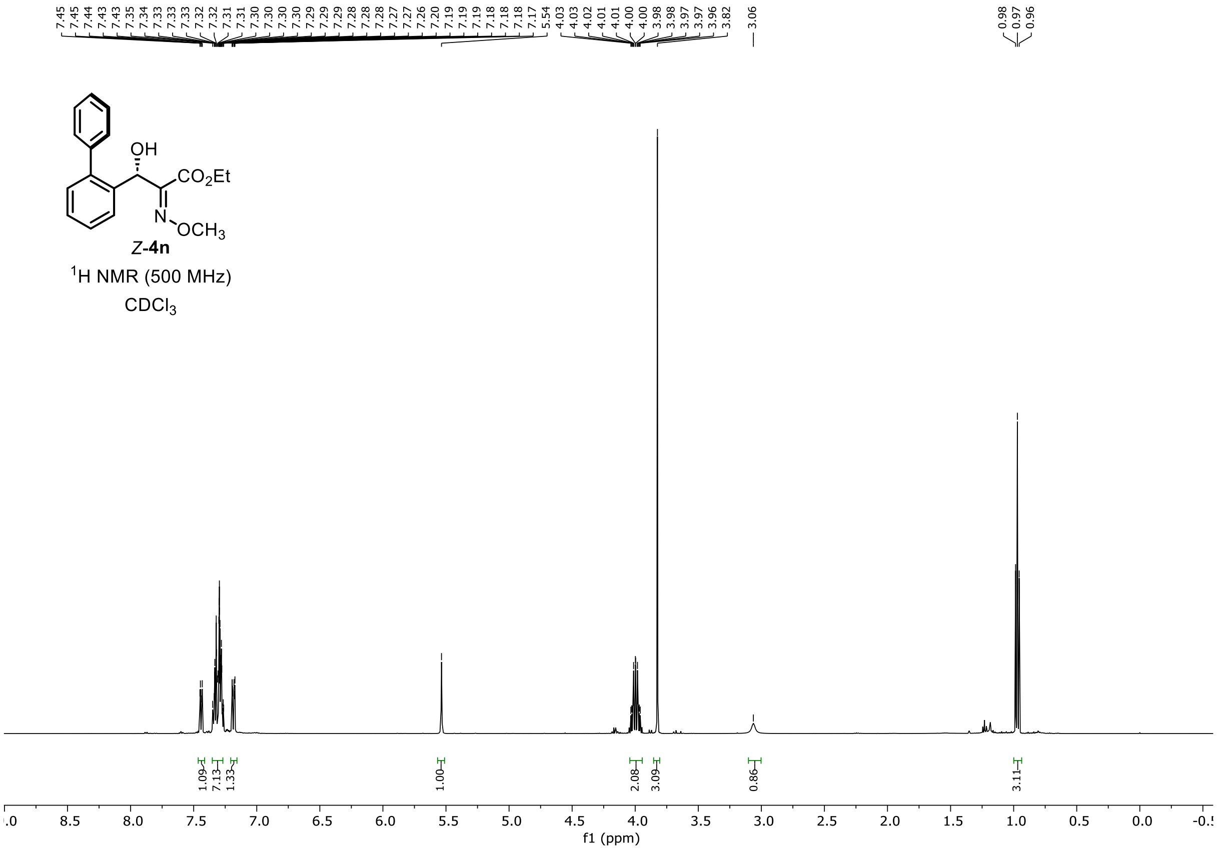

OH
CO2Et
N
OCH3
Z-4n
1H NMR (500 MHz)
CDCl3
f1 (ppm)

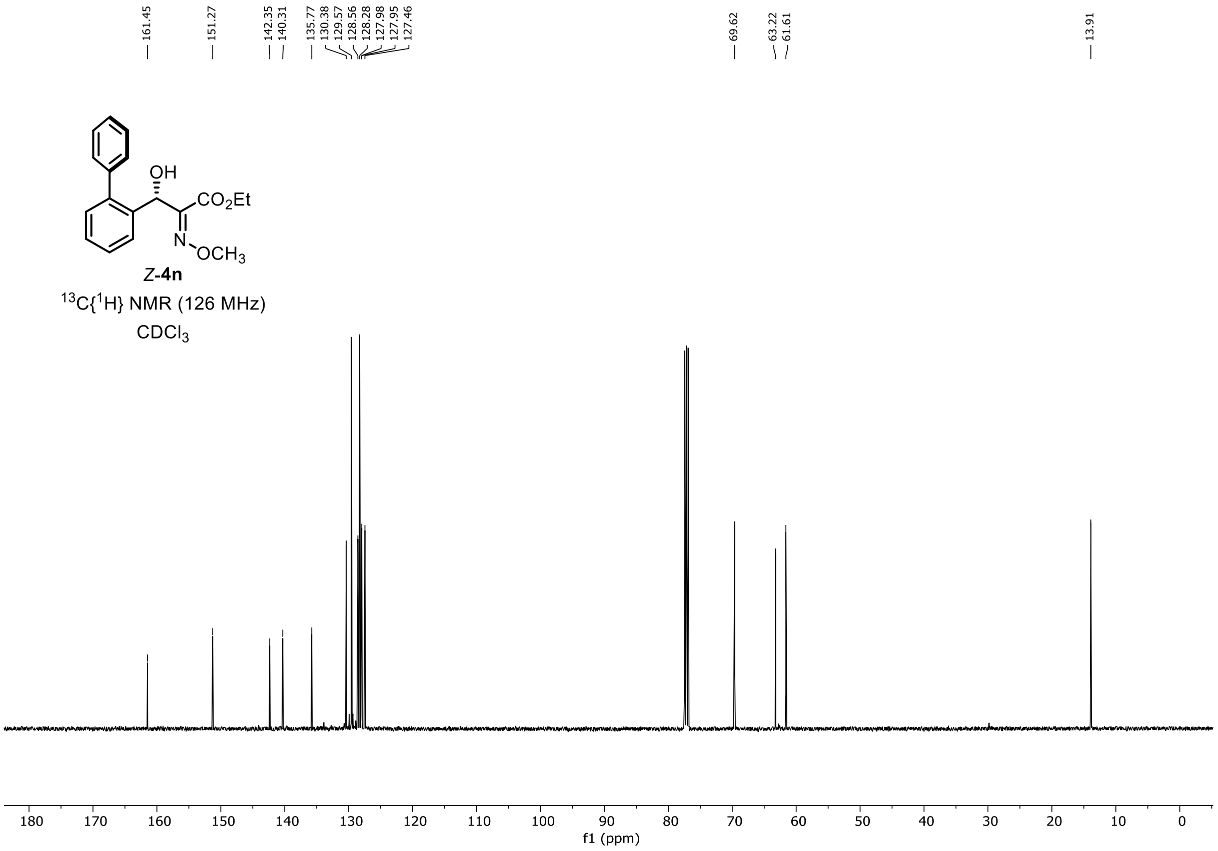

161.45
151.27
142.35
140.31
135.77
130.38
129.57
128.56
128.28
127.98
127.95
127.46
69.62
63.22
61.61
13.91
OH
CO2Et
N
OCH3
Z-4n
13C{1H} NMR (126 MHz)
CDCl3
f1 (ppm)

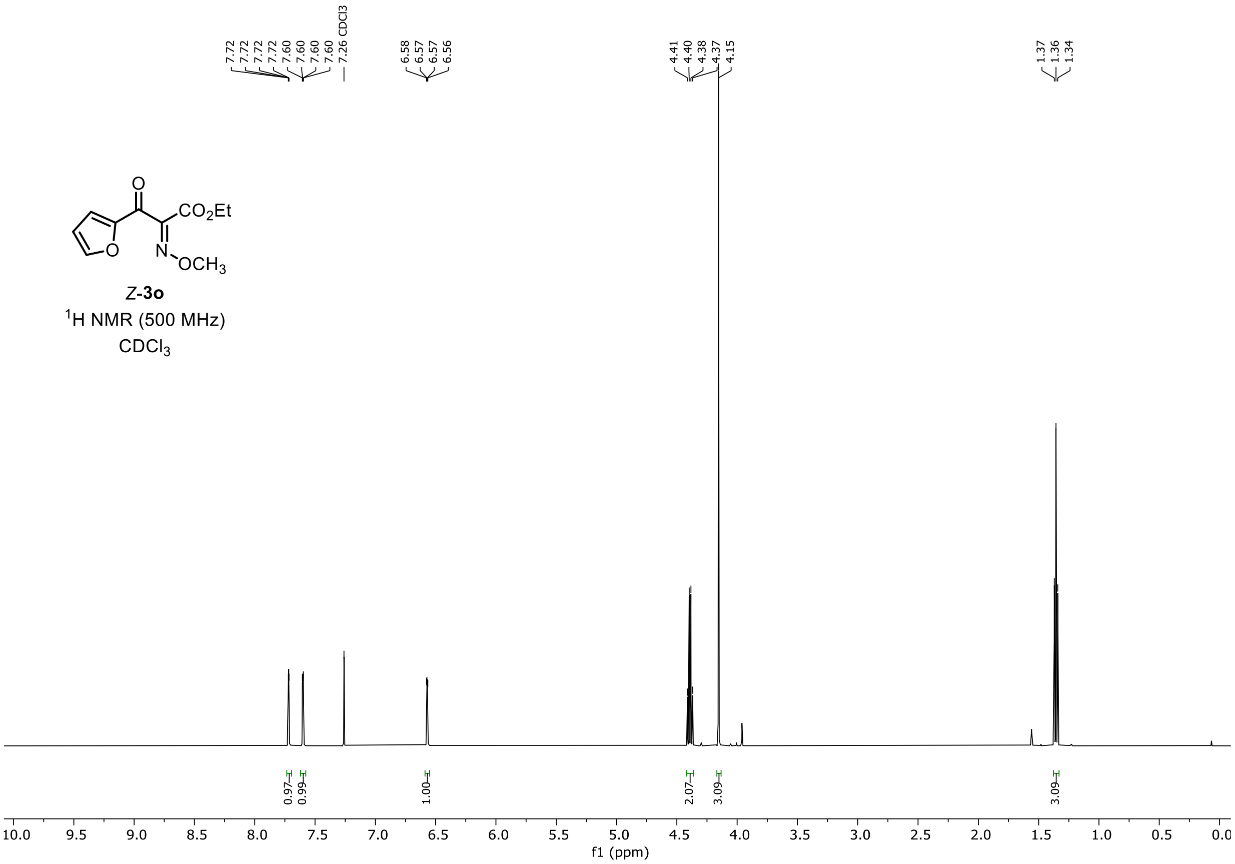
7.72 7.72 7.72 7.72 7.60 7.60 7.60 7.60 7.26 CDCl3
6.58 6.57 6.57 6.56
4.41 4.40 4.38 4.37 4.15
1.37 1.36 1.34
CO2Et
N
OCH3
O
Z-3o
1H NMR (500 MHz)
CDCl3
0.97 0.99 1.00 2.07 3.09 3.09
f1 (ppm)

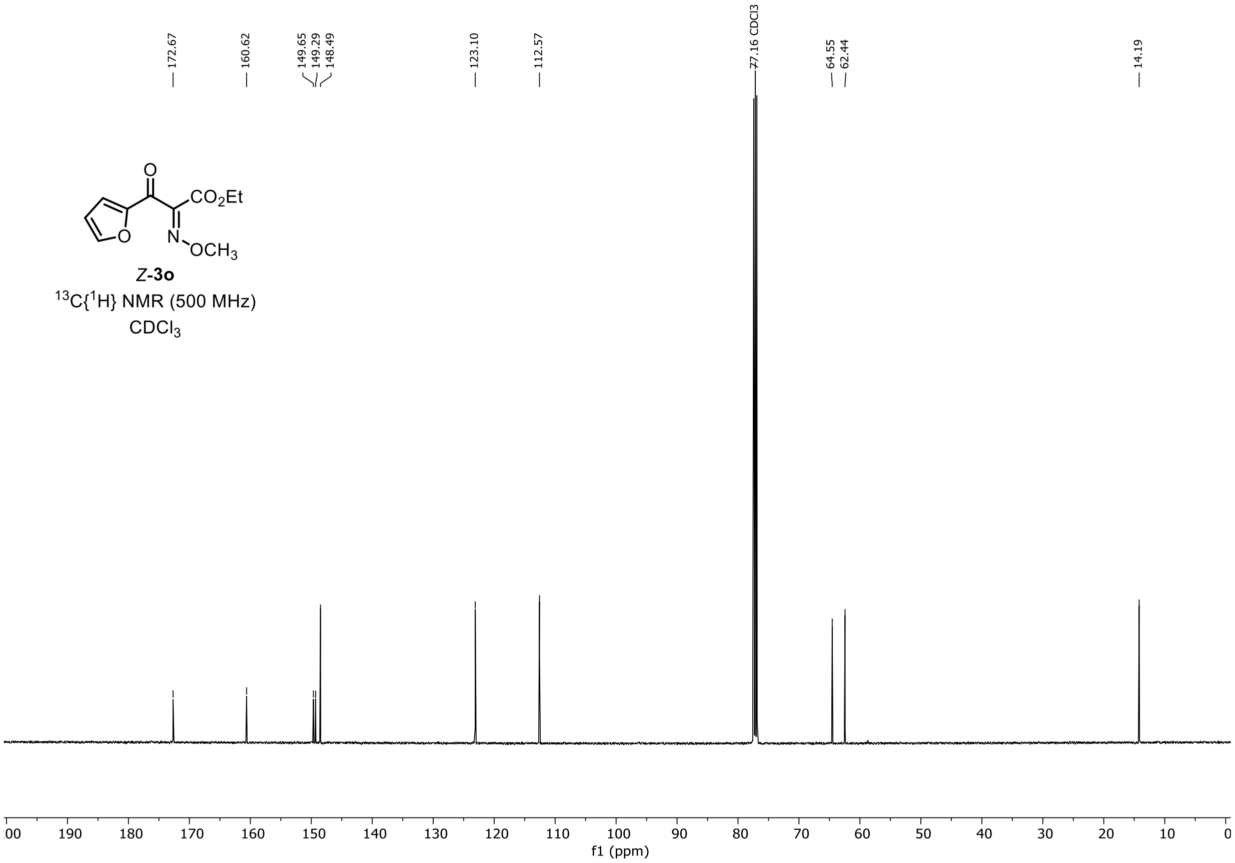
172.67
160.62
149.65 149.29 148.49
123.10
112.57
77.16 CDCl3
64.55 62.44
14.19
CO2Et
N
OCH3
O
Z-3o
13C{1H} NMR (500 MHz)
CDCl3
f1 (ppm)

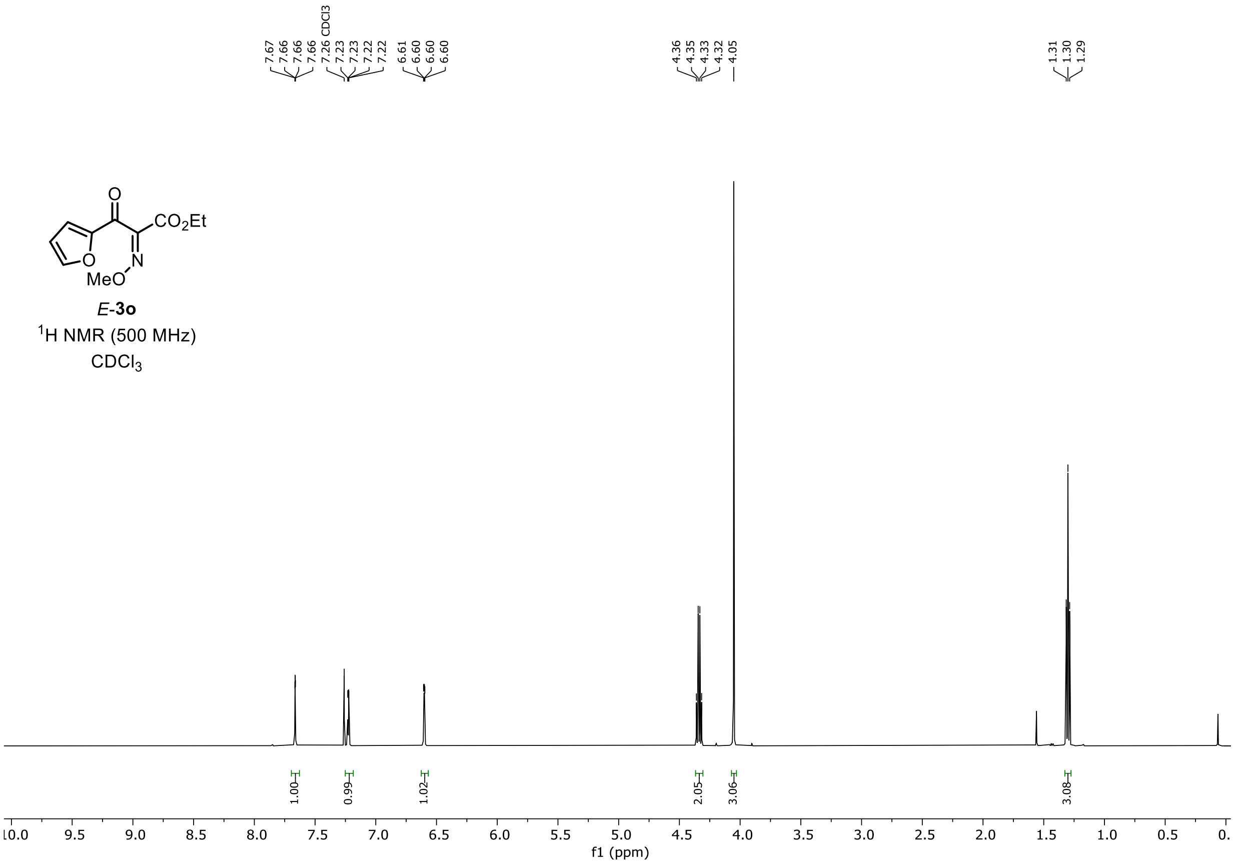
7.67 7.66 7.66 7.66 7.26 CDCl3 7.23 7.23 7.22 7.22 6.61 6.60 6.60 6.60
4.36 4.35 4.33 4.32 4.05
1.31 1.30 1.29
CO2Et
O
N
MeO
E-3o
1H NMR (500 MHz)
CDCl3
1.00 0.99 1.02 2.05 3.06 3.08
f1 (ppm)

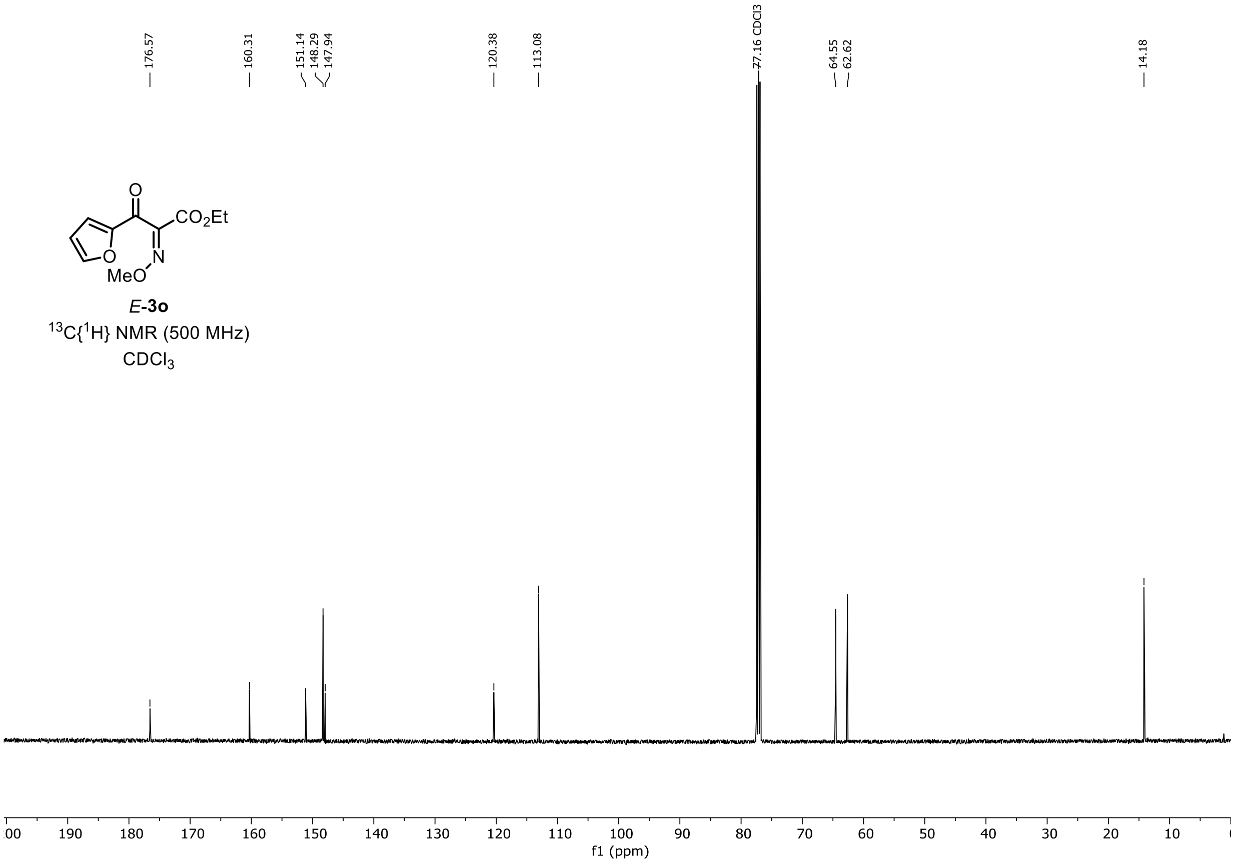
176.57
160.31
151.14 148.29 147.94
120.38
113.08
77.16 CDCl3
64.55 62.62
14.18
CO2Et
O
N
MeO
E-3o
13C{1H} NMR (500 MHz)
CDCl3
f1 (ppm)

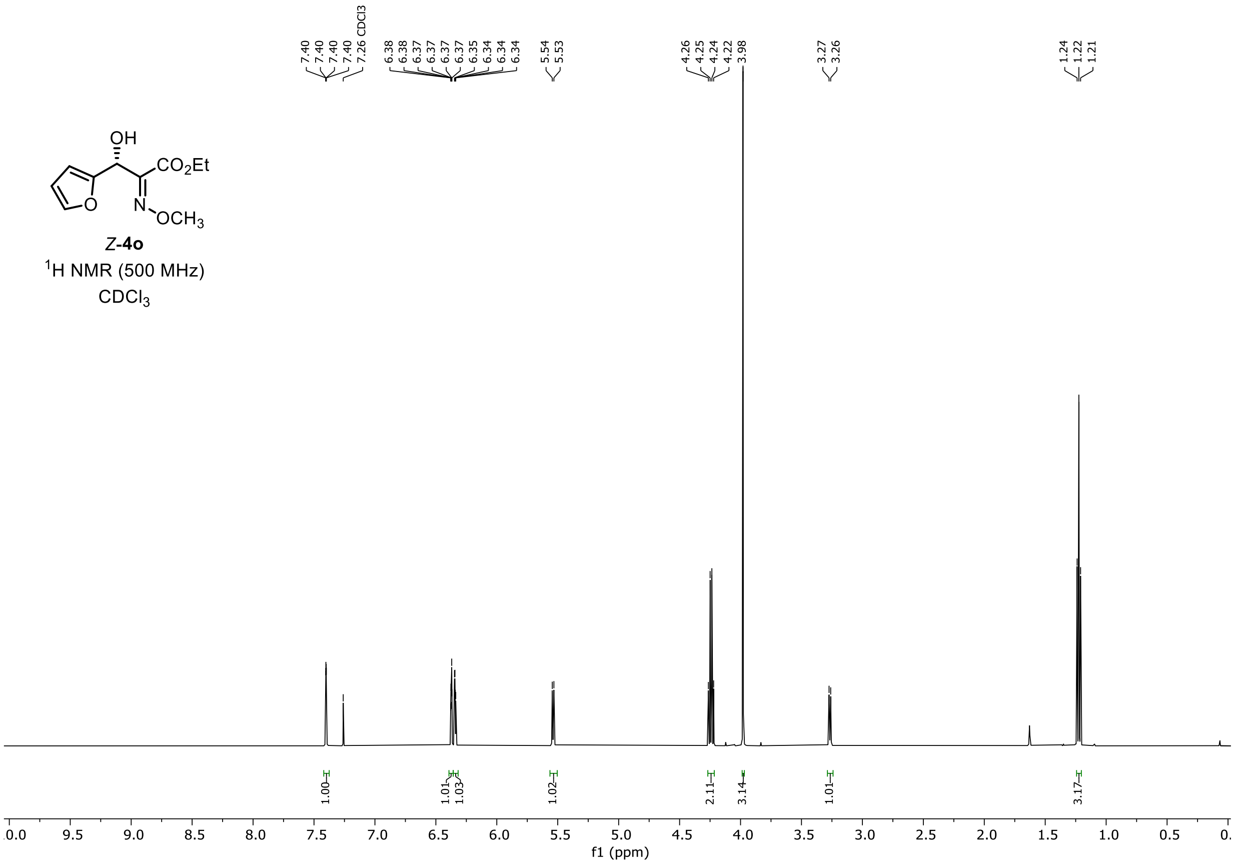

7.40
7.40
7.40
7.40
7.26 CDCl3
6.38
6.38
6.37
6.37
6.37
6.37
6.35
6.34
6.34
6.34
5.54
5.53
4.26
4.25
4.24
4.22
3.98
3.27
3.26
1.24
1.22
1.21
OH
CO2Et
O
N
OCH3
Z-4o
1H NMR (500 MHz)
CDCl3
1.00
1.01
1.03
1.02
2.11
3.14
1.01
3.17
f1 (ppm)

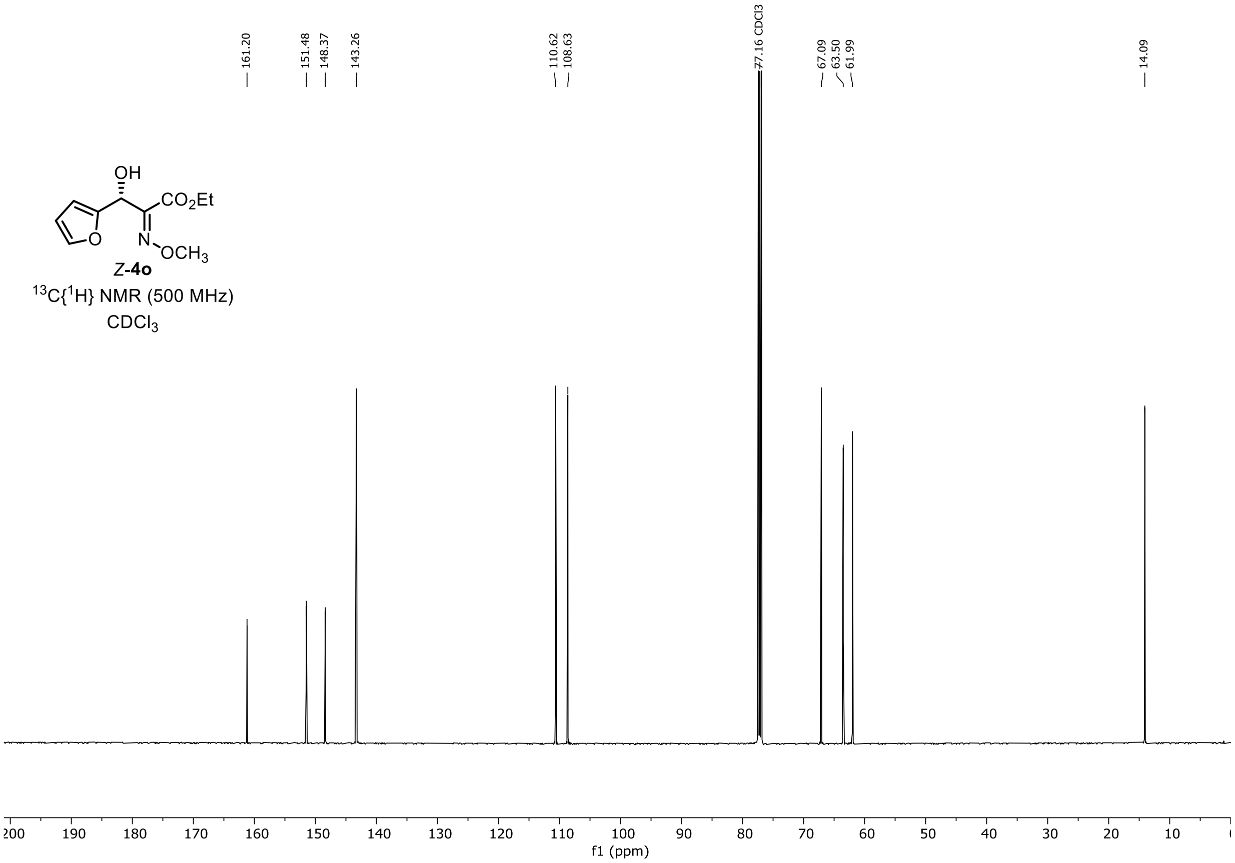

161.20
151.48
148.37
143.26
110.62
108.63
77.16 CDCl3
67.09
63.50
61.99
14.09
OH
CO2Et
O
N
OCH3
Z-4o
13C{1H} NMR (500 MHz)
CDCl3
f1 (ppm)

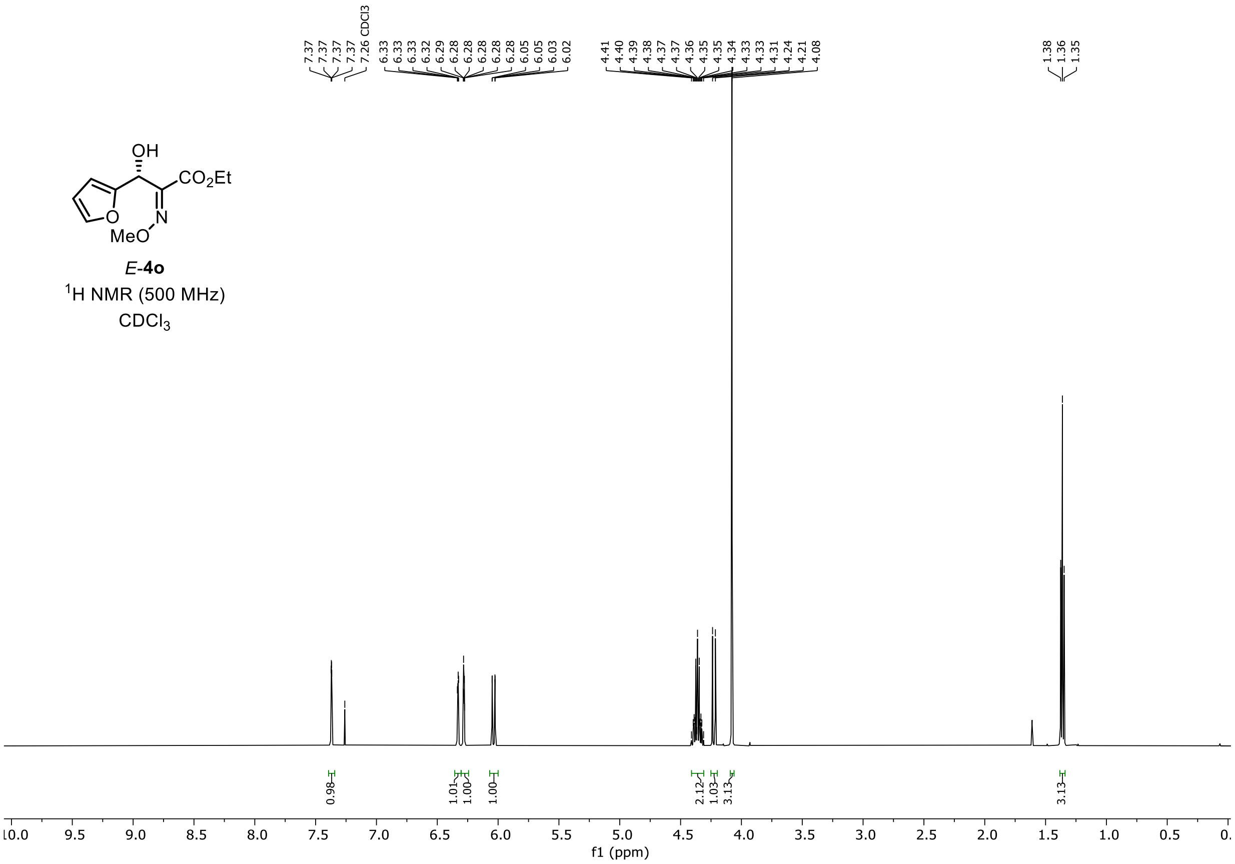
7.37 7.37 7.37 7.37 7.26 CDCl3
6.33 6.33 6.33 6.32 6.29 6.28 6.28 6.28 6.28 6.28 6.05 6.05 6.03 6.02
4.41 4.40 4.39 4.38 4.37 4.37 4.36 4.35 4.35 4.34 4.33 4.33 4.33 4.31 4.24 4.21 4.08
1.38 1.36 1.35
OH
CO2Et
O
N
MeO
E-4o
1H NMR (500 MHz)
CDCl3
0.98
1.01 1.00
1.00
2.12 1.03 3.13
3.13
f1 (ppm)

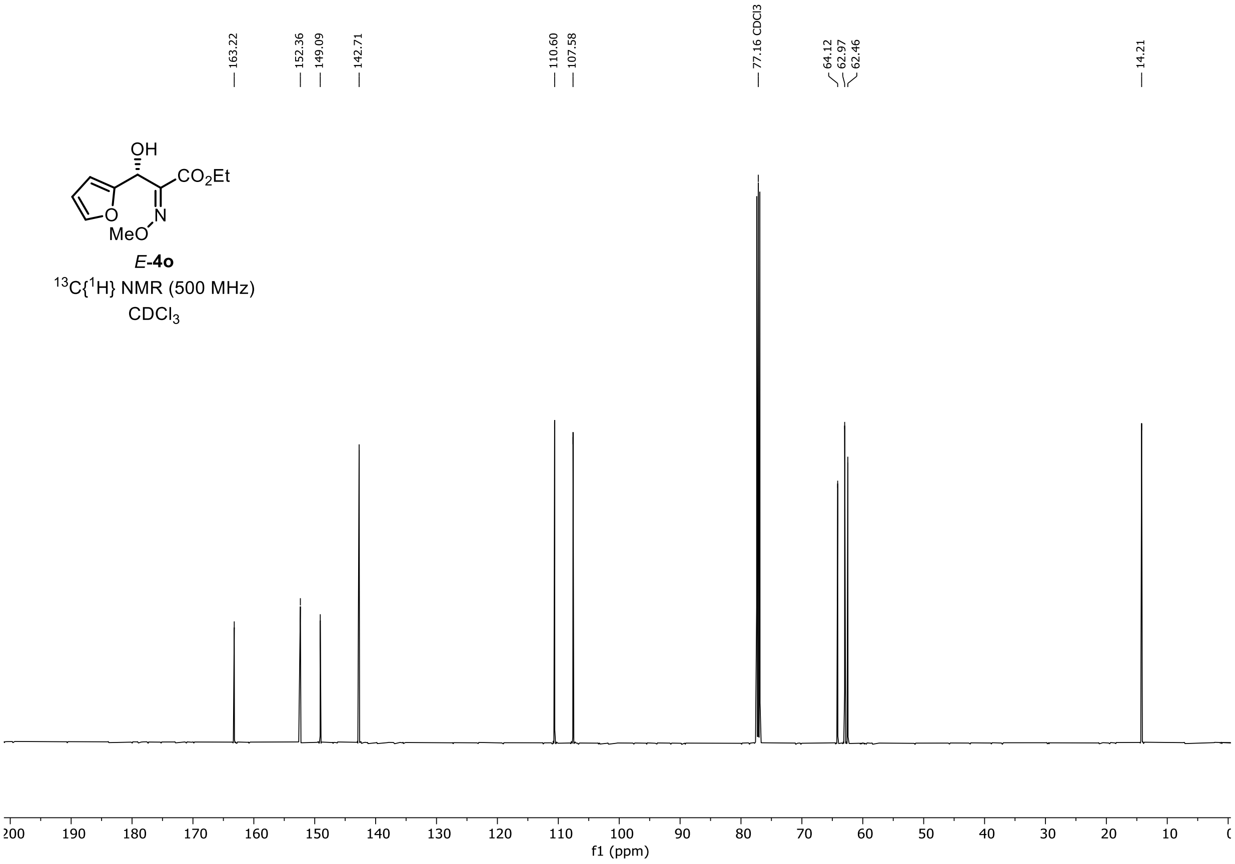
163.22
152.36 149.09
142.71
110.60 107.58
77.16 CDCl3
64.12 62.97 62.46
14.21
OH
CO2Et
O
N
MeO
E-4o
13C{1H} NMR (500 MHz)
CDCl3
f1 (ppm)

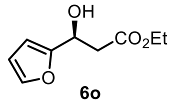

6o

^{1}H NMR (500 MHz)

$CDCl_3$

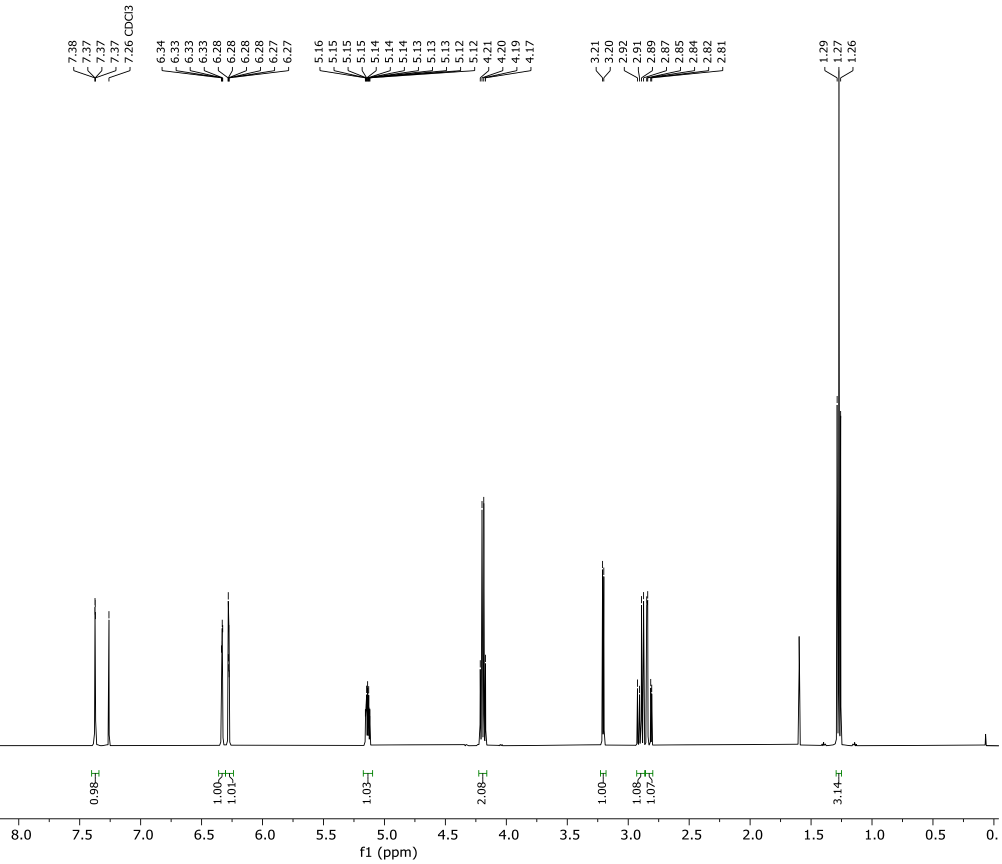

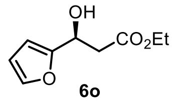

6o

$^{13}C\{^1H\}$ NMR (500 MHz)

$CDCl_3$

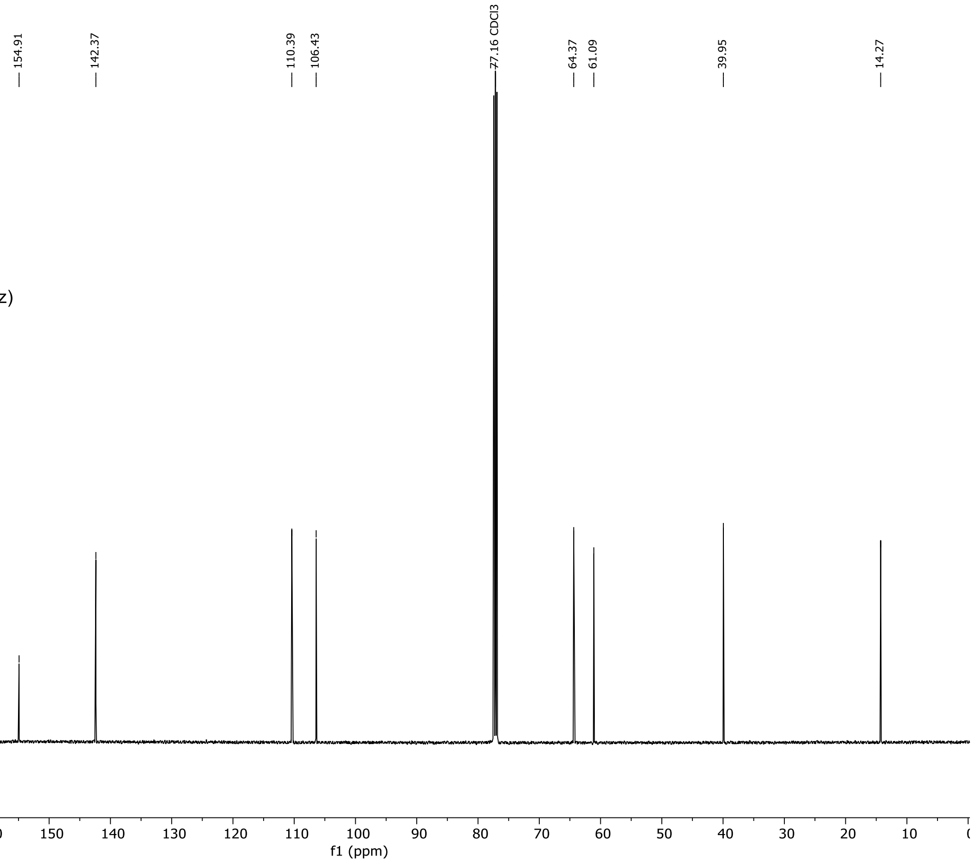

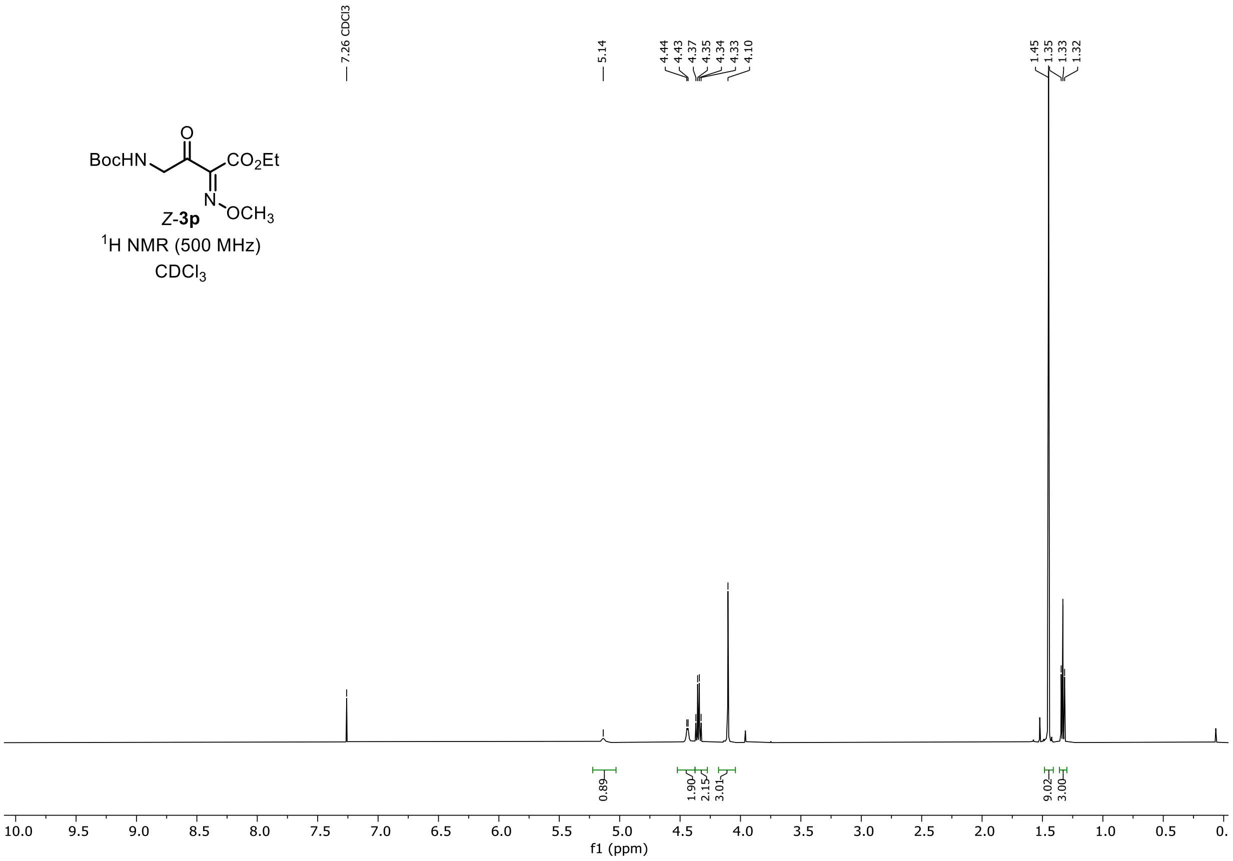
7.26 CDCl3
5.14
4.44
4.43
4.37
4.35
4.34
4.33
4.10
1.45
1.35
1.33
1.32
BocHN
O
CO2Et
N
OCH3
Z-3p
1H NMR (500 MHz)
CDCl3
0.89
1.90
2.15
3.01
9.02
3.00
10.0 9.5 9.0 8.5 8.0 7.5 7.0 6.5 6.0 5.5 5.0 4.5 4.0 3.5 3.0 2.5 2.0 1.5 1.0 0.5 0.
f1 (ppm)

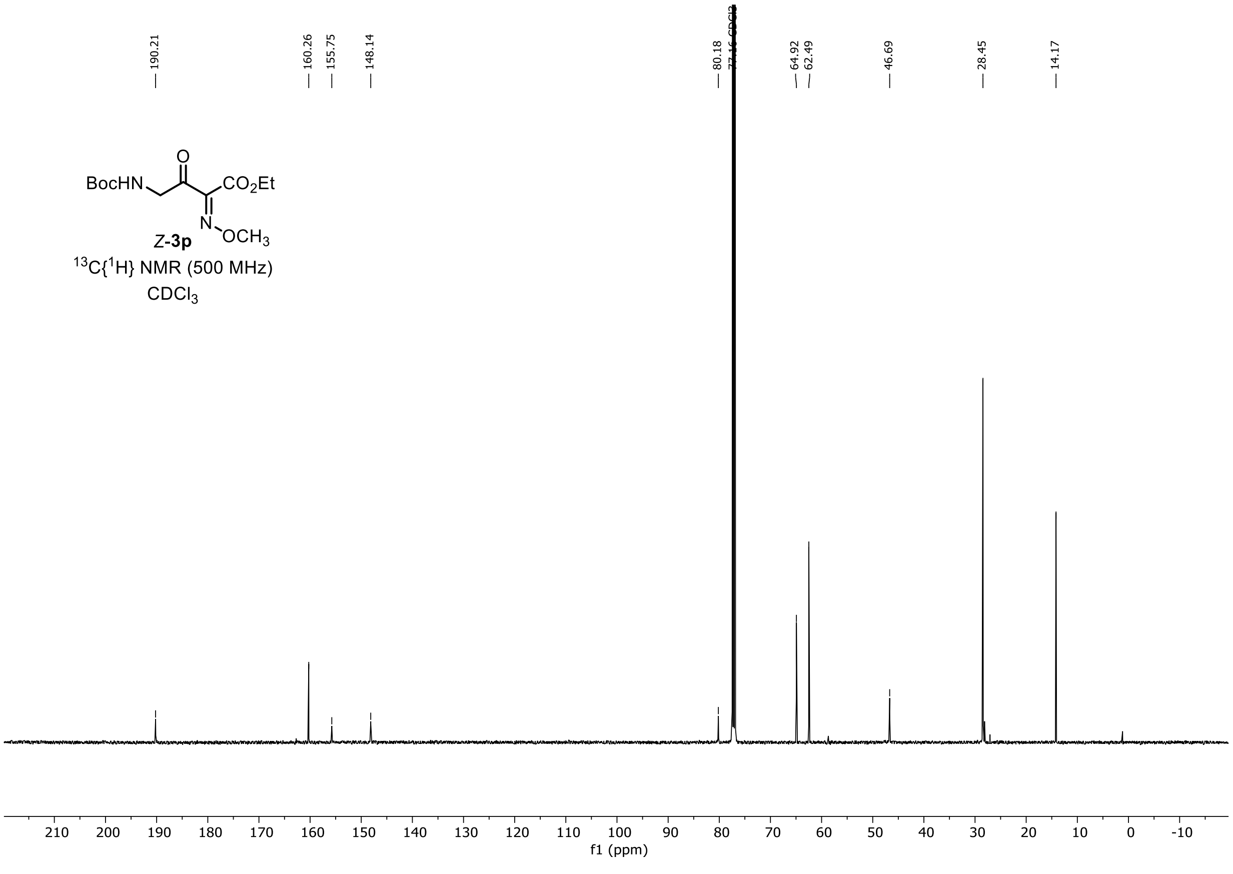
190.21
160.26
155.75
148.14
80.18
77.16 CDCl3
64.92
62.49
46.69
28.45
14.17
BocHN
O
CO2Et
N
OCH3
Z-3p
13C{1H} NMR (500 MHz)
CDCl3
210 200 190 180 170 160 150 140 130 120 110 100 90 80 70 60 50 40 30 20 10 0 -10
f1 (ppm)

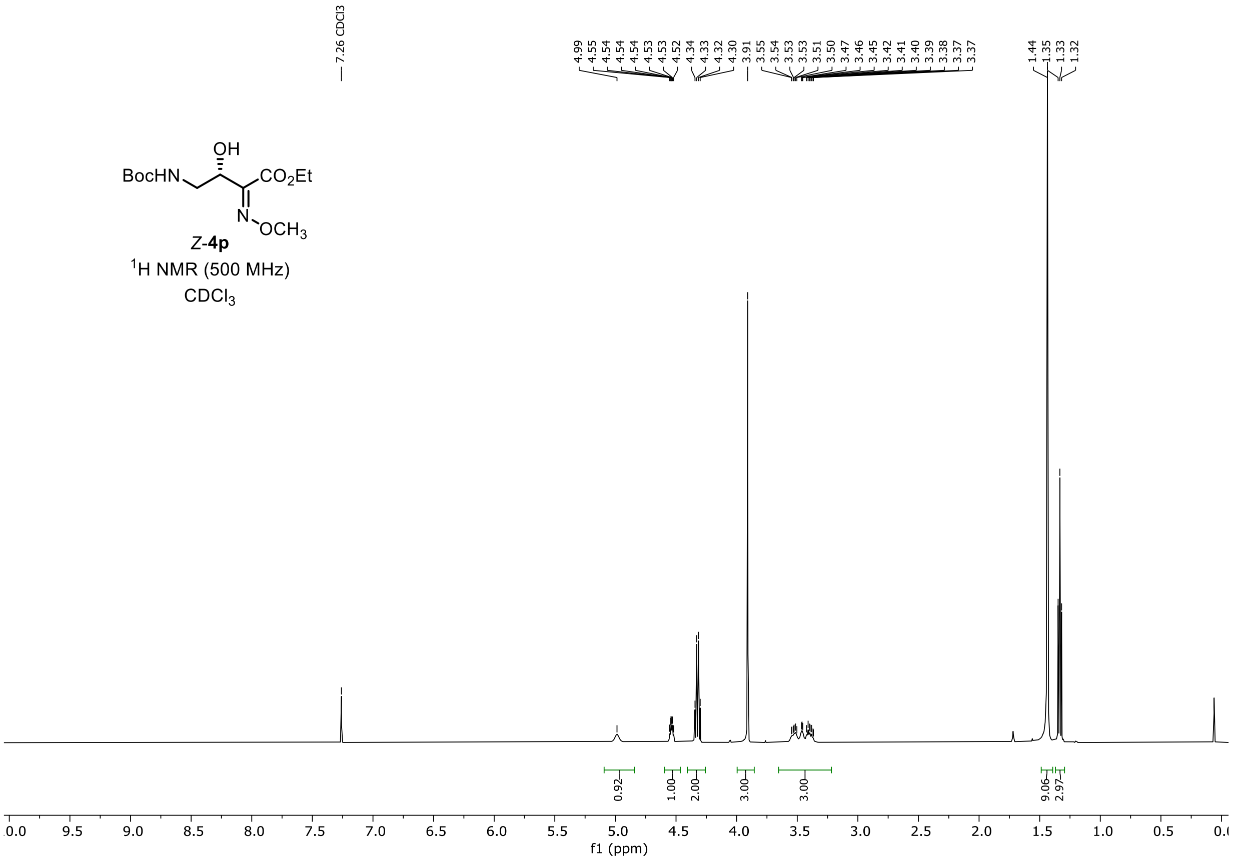
Z-4p
^{1}H NMR (500 MHz)
$CDCl_3$
f1 (ppm)

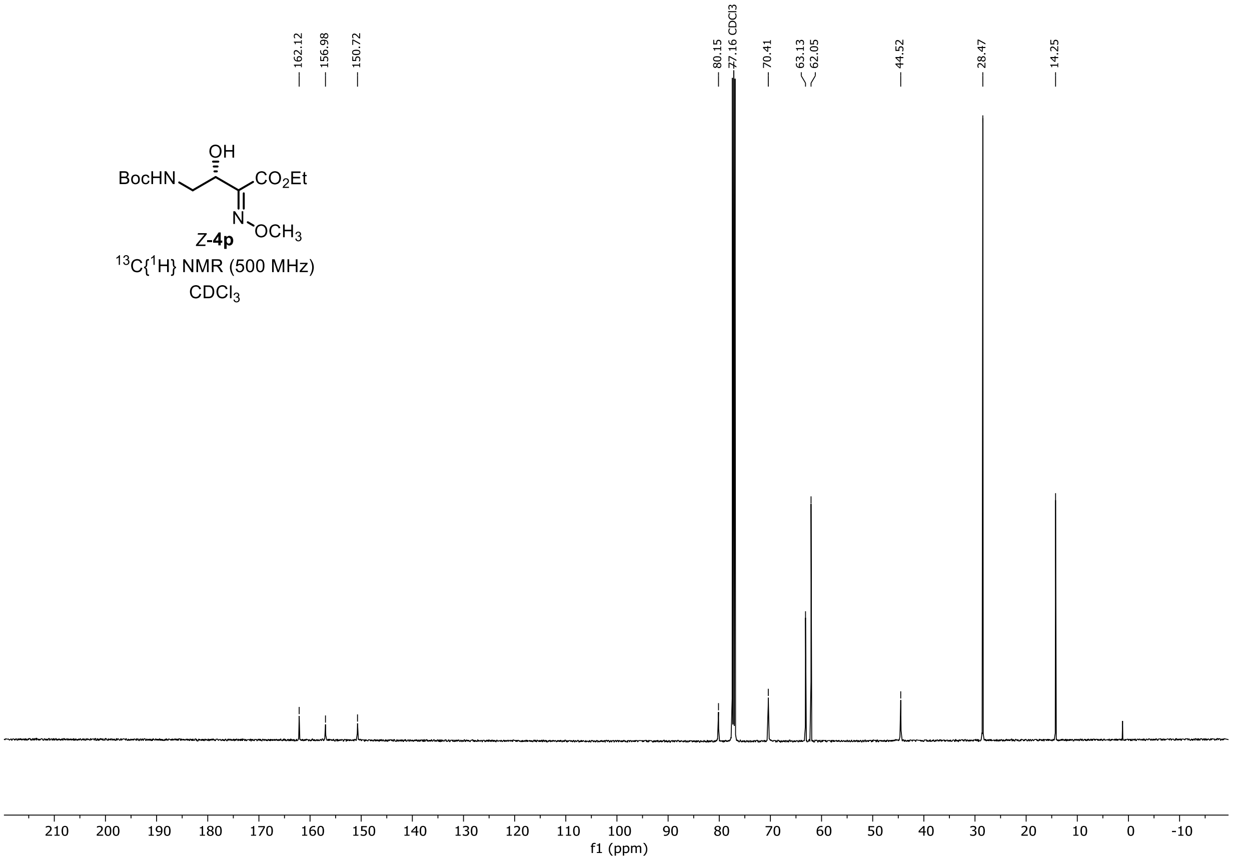
Z-4p
$^{13}C\{^1H\}$ NMR (500 MHz)
$CDCl_3$
f1 (ppm)

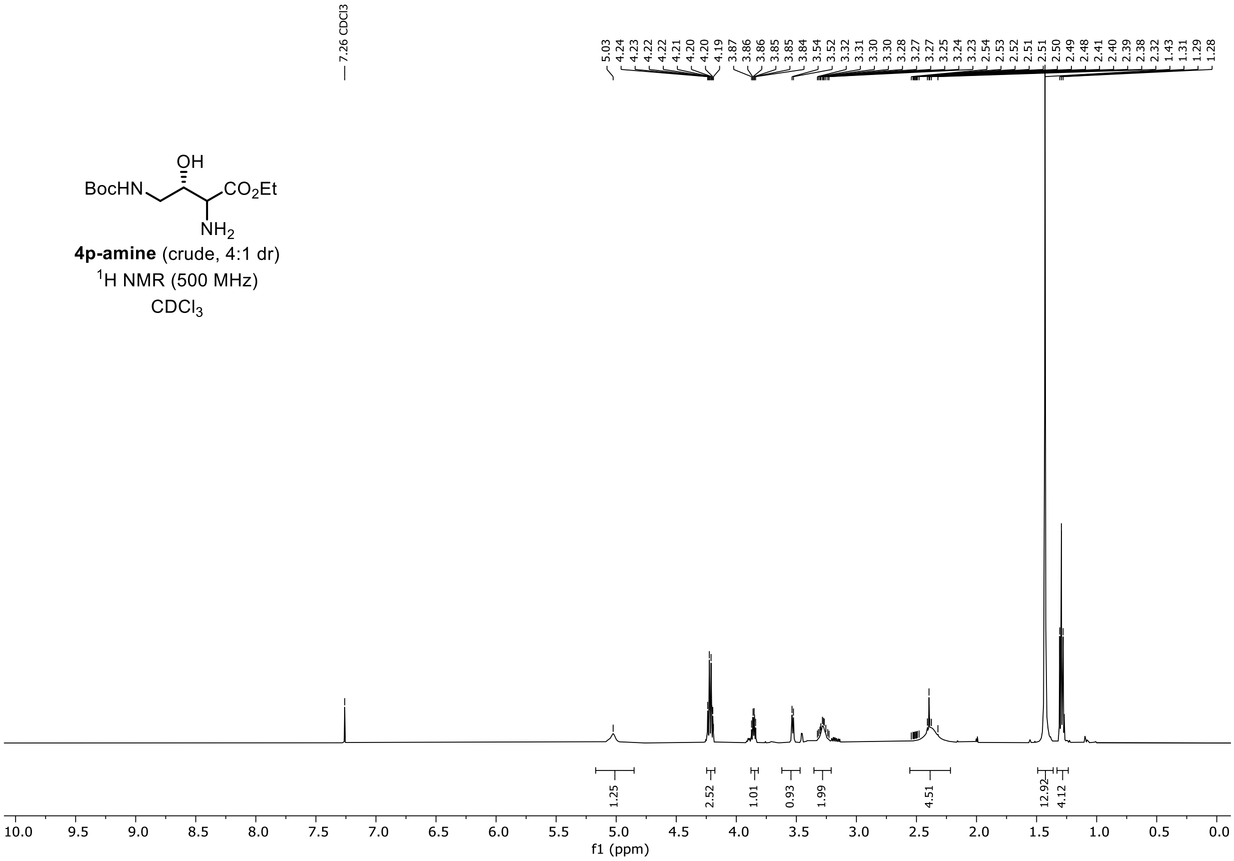

4p-amine (crude, 4:1 dr)
^1H NMR (500 MHz)
$CDCl_3$

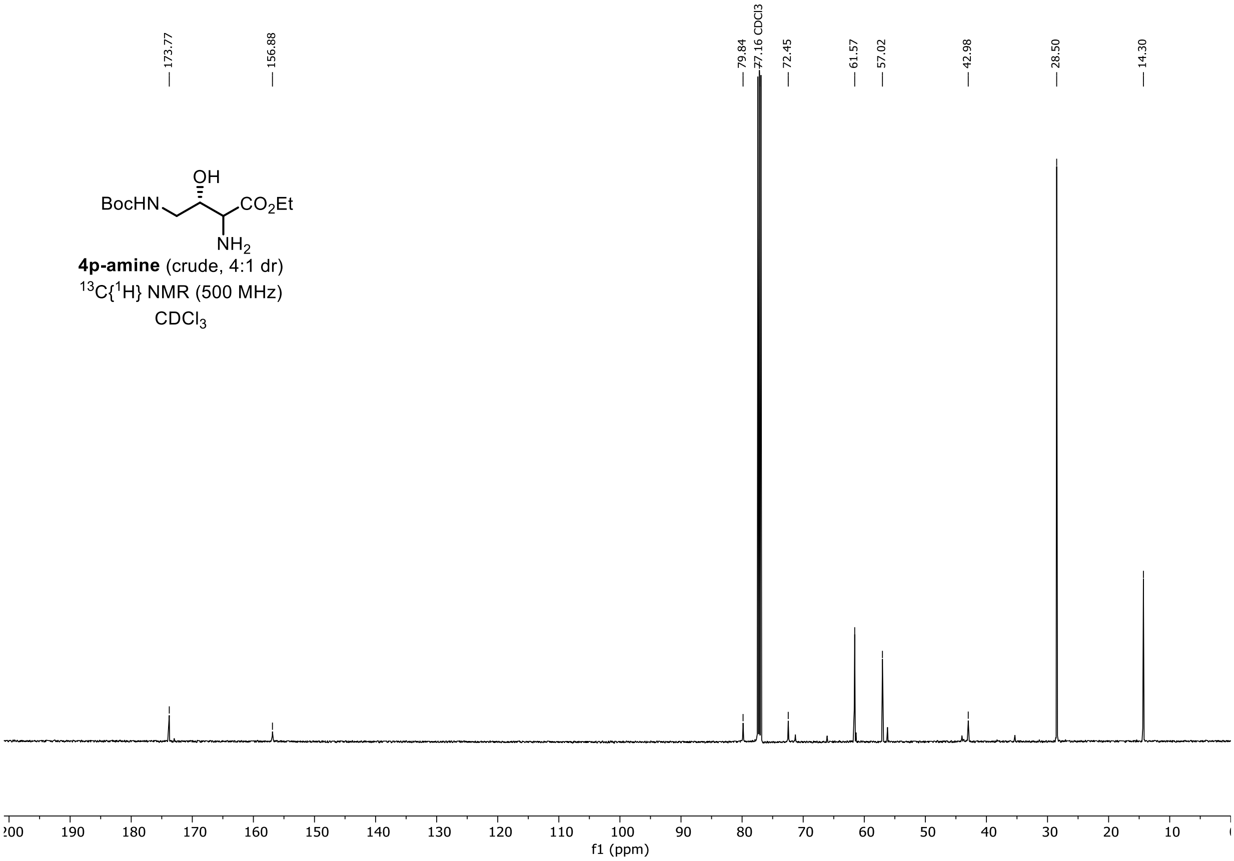

4p-amine (crude, 4:1 dr)
$^{13}C\{^1H\}$ NMR (500 MHz)
$CDCl_3$

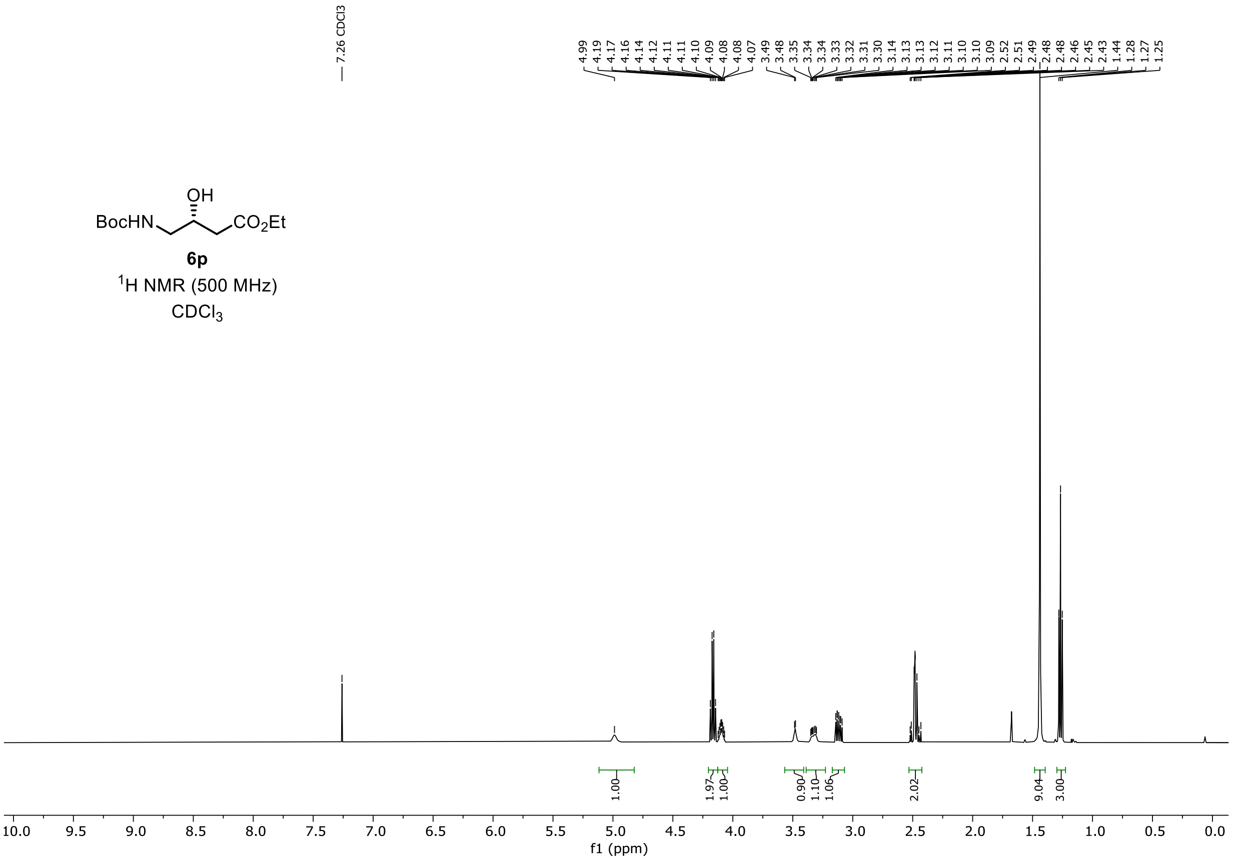
7.26 CDCl3
4.99
4.19
4.17
4.16
4.14
4.12
4.11
4.11
4.10
4.09
4.08
4.08
4.07
3.49
3.48
3.35
3.34
3.34
3.33
3.32
3.31
3.30
3.14
3.13
3.13
3.12
3.11
3.10
3.10
3.09
2.52
2.51
2.49
2.48
2.48
2.46
2.45
2.43
1.44
1.28
1.27
1.25
OH
BocHN
CO2Et
6p
1H NMR (500 MHz)
CDCl3
1.00
1.97
1.00
0.90
1.10
1.06
2.02
9.04
3.00
f1 (ppm)

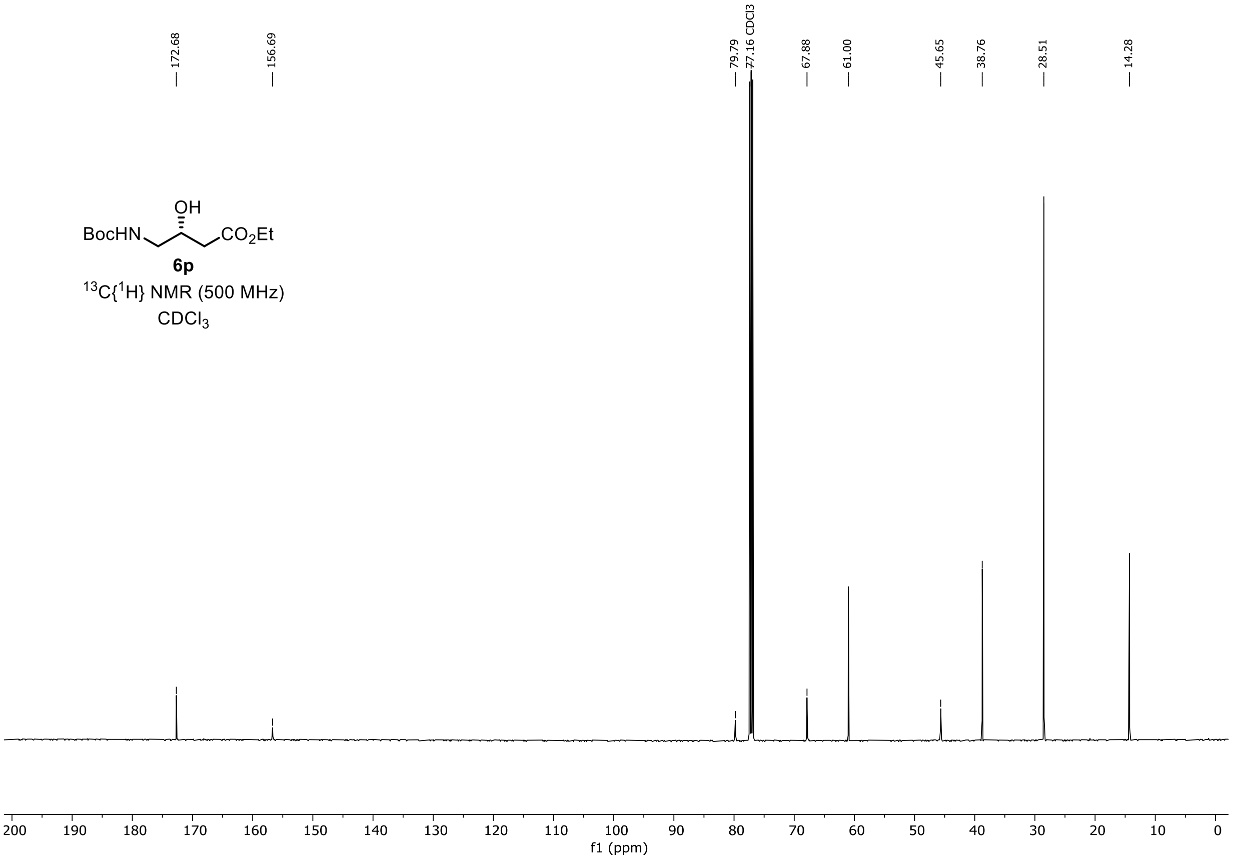
172.68
156.69
79.79
77.16 CDCl3
67.88
61.00
45.65
38.76
28.51
14.28
OH
BocHN
CO2Et
6p
13C{1H} NMR (500 MHz)
CDCl3
f1 (ppm)

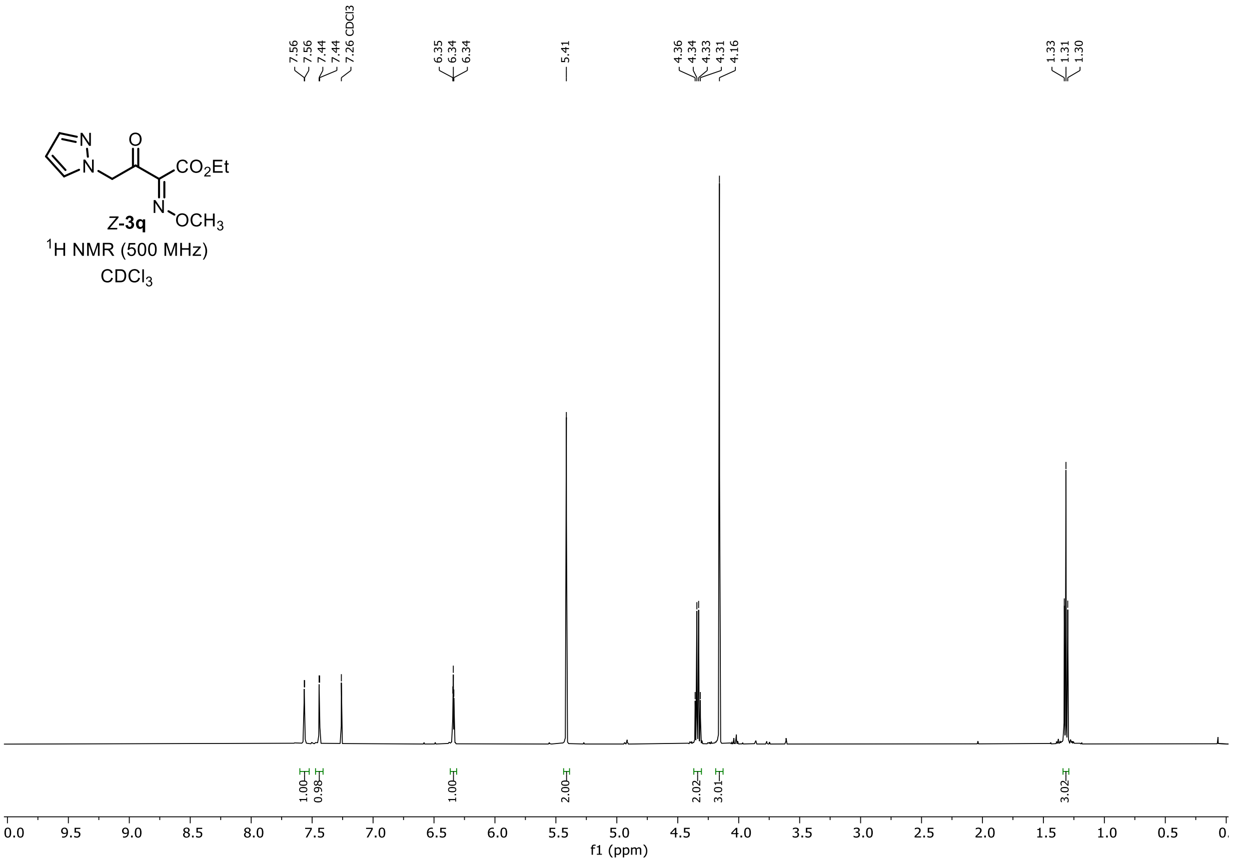
7.56
7.56
7.44
7.44
7.26 CDCl3
6.35
6.34
6.34
5.41
4.36
4.34
4.33
4.31
4.16
1.33
1.31
1.30
CO2Et
N
OCH3
Z-3q
1H NMR (500 MHz)
CDCl3
1.00
0.98
1.00
2.00
2.02
3.01
3.02
f1 (ppm)

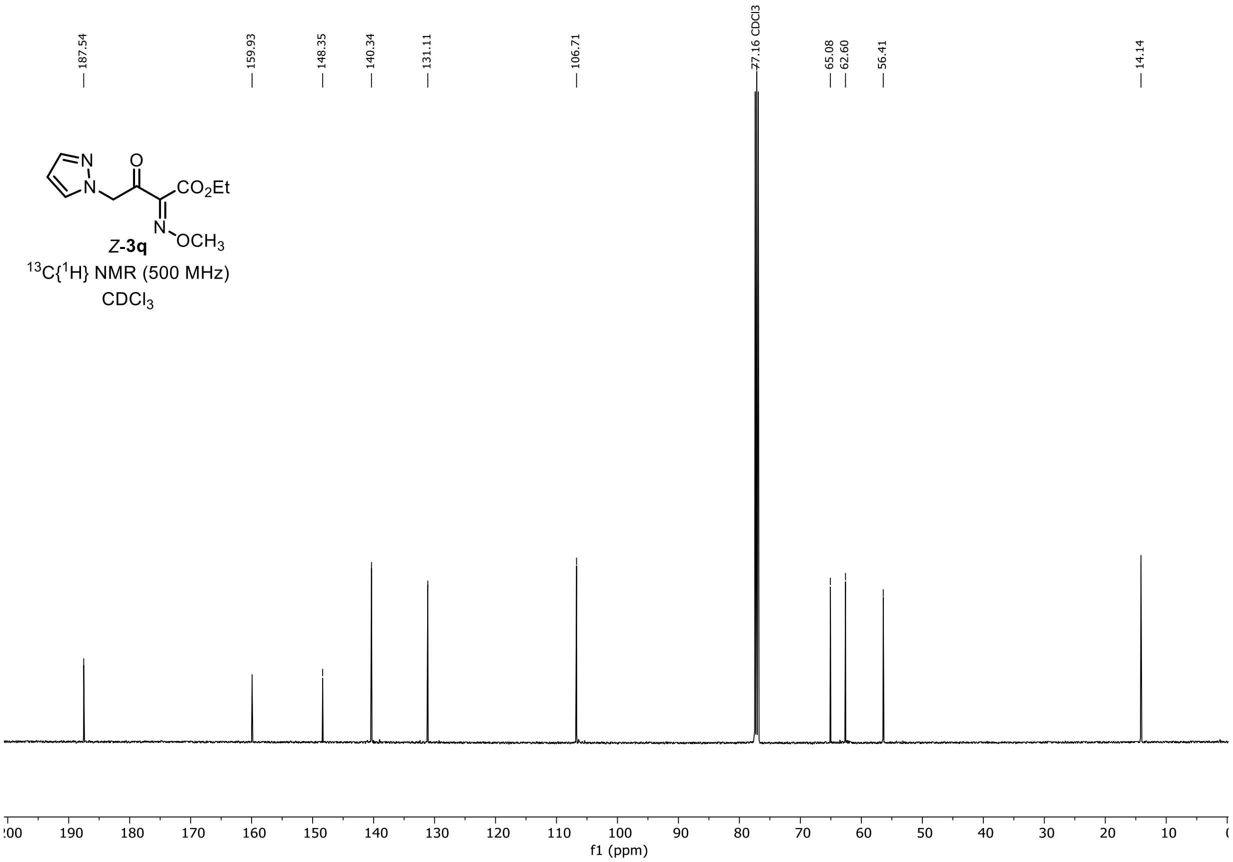
187.54
159.93
148.35
140.34
131.11
106.71
77.16 CDCl3
65.08
62.60
56.41
14.14
CO2Et
N
OCH3
Z-3q
13C{1H} NMR (500 MHz)
CDCl3
f1 (ppm)

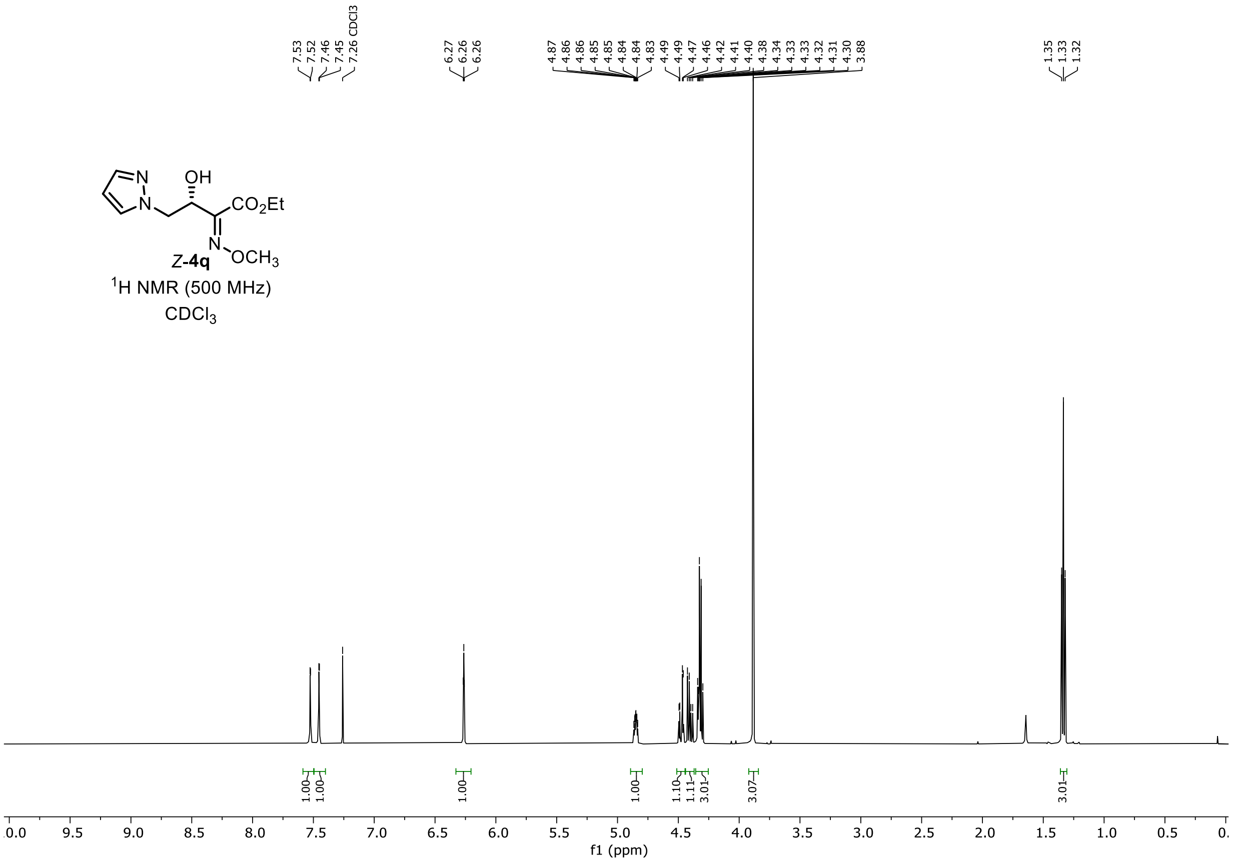
Z-4q
1H NMR (500 MHz)
CDCl3
7.53 7.52 7.46 7.45 7.26 CDCl3
6.27 6.26 6.26
4.87 4.86 4.86 4.85 4.85 4.84 4.84 4.83 4.49 4.49 4.47 4.46 4.42 4.41 4.40 4.38 4.34 4.33 4.33 4.32 4.31 4.30 3.88
1.35 1.33 1.32
1.00 1.00 1.00 1.00 1.10 1.11 3.01 3.07 3.01
f1 (ppm)

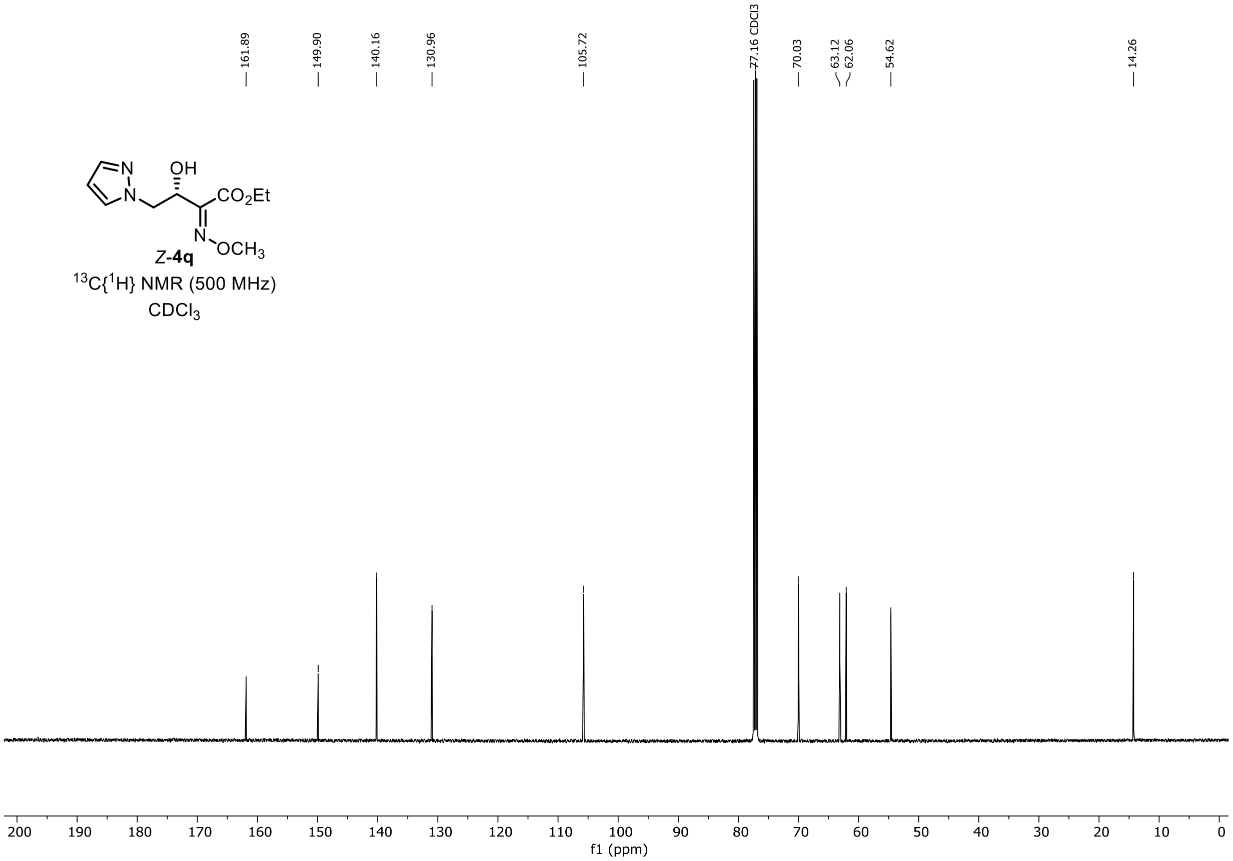
Z-4q
13C{1H} NMR (500 MHz)
CDCl3
161.89 149.90 140.16 130.96 105.72 77.16 CDCl3 70.03 63.12 62.06 54.62 14.26
f1 (ppm)

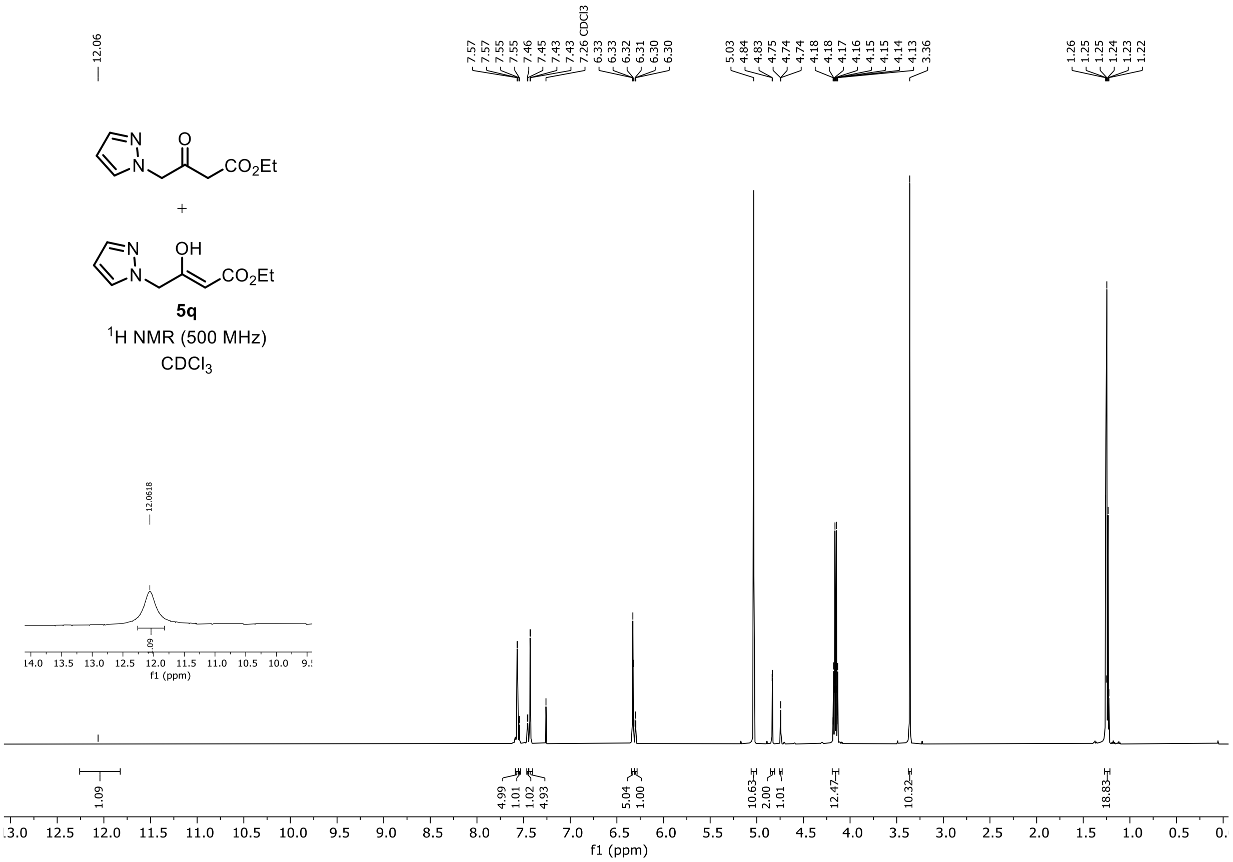
12.06
7.57 7.57 7.55 7.55 7.46 7.45 7.43 7.43 7.26 CDCl3 6.33 6.33 6.32 6.31 6.30 6.30
5.03 4.84 4.83 4.75 4.74 4.74 4.74 4.18 4.18 4.17 4.16 4.15 4.15 4.15 4.14 4.13 3.36
1.26 1.25 1.25 1.24 1.23 1.22
CO2Et
+
OH
CO2Et
5q
1H NMR (500 MHz)
CDCl3
12.0618
1.09
4.99 1.01 1.02 4.93
5.04 1.00
10.63 2.00 1.01
12.47
10.32
18.83
f1 (ppm)

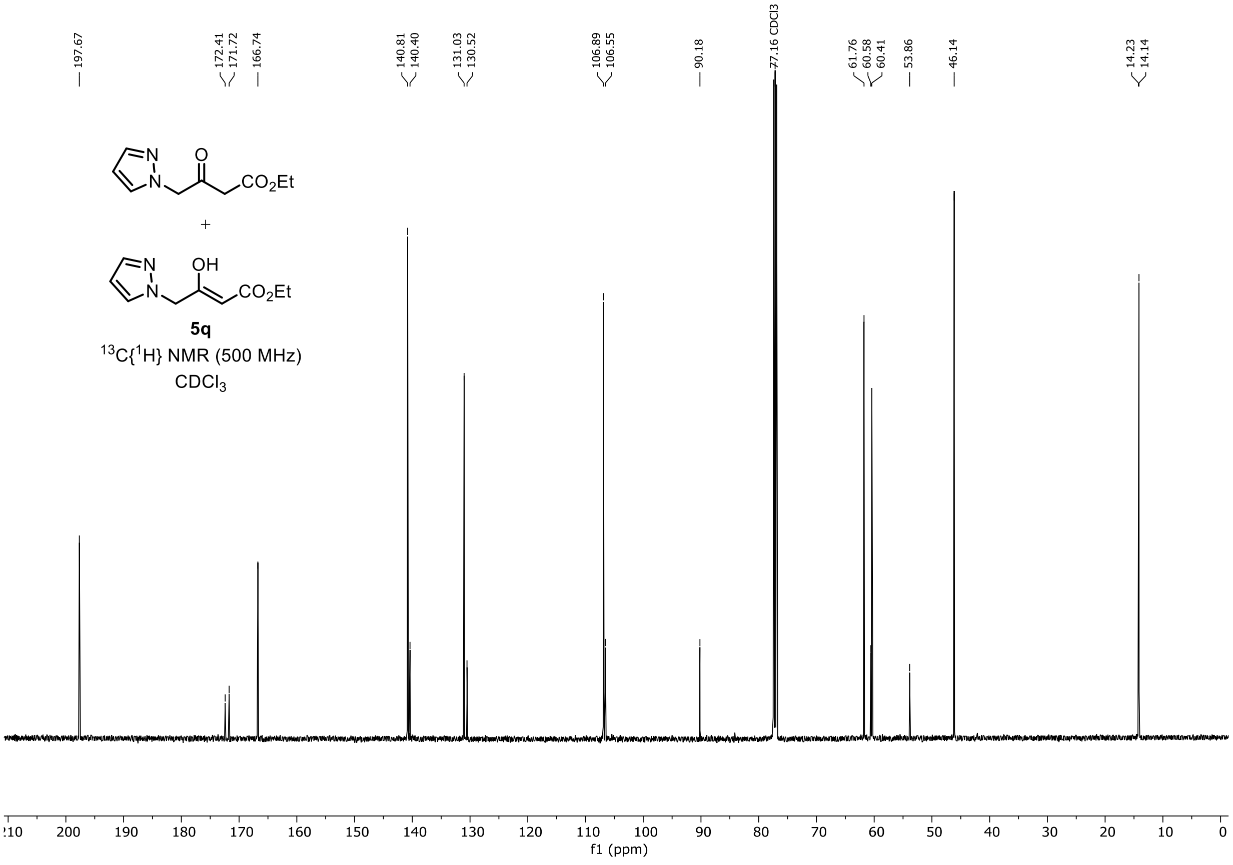
197.67
172.41 171.72 166.74
140.81 140.40
131.03 130.52
106.89 106.55
90.18
77.16 CDCl3
61.76 60.58 60.41
53.86
46.14
14.23 14.14
CO2Et
+
OH
CO2Et
5q
13C{1H} NMR (500 MHz)
CDCl3
f1 (ppm)

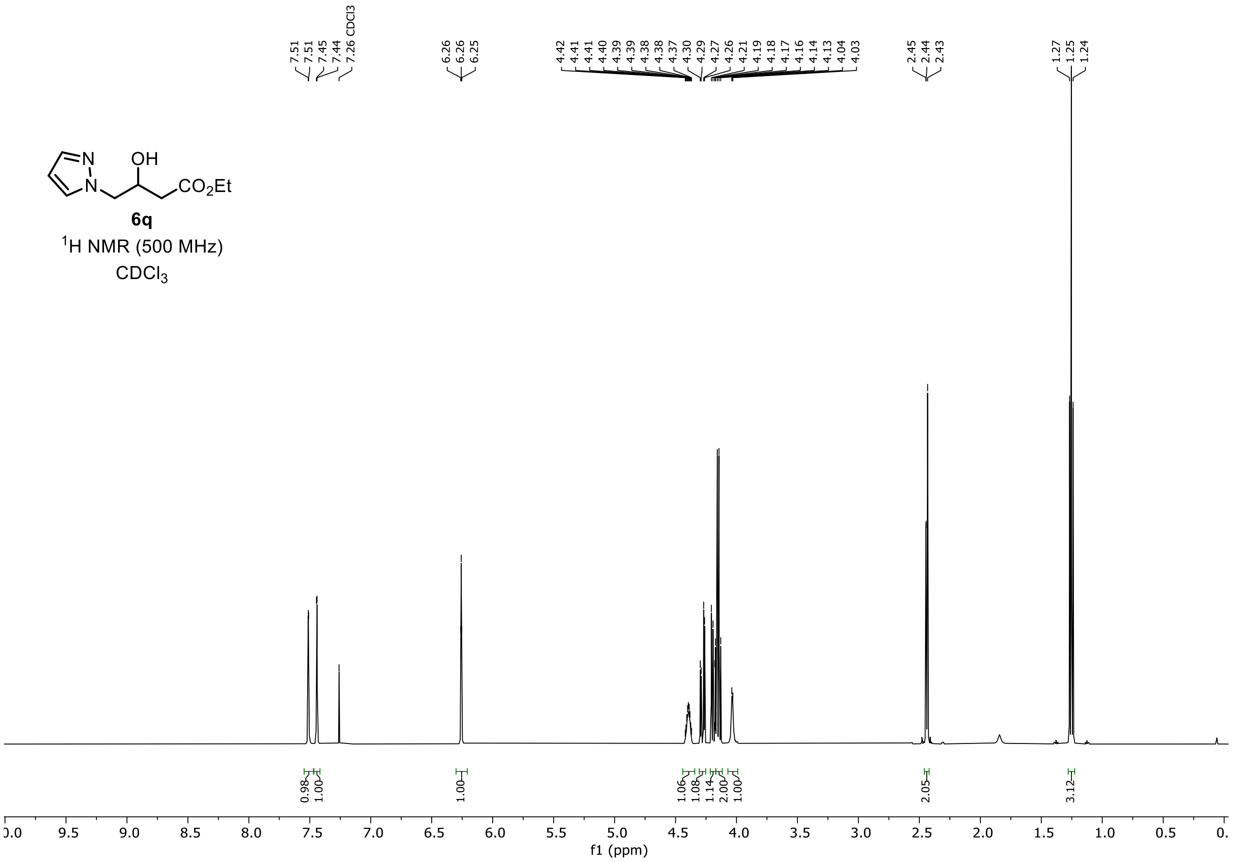
6q
^{1}H NMR (500 MHz)
$CDCl_3$
7.51
7.51
7.45
7.44
7.26 CDCl3
6.26
6.26
6.25
4.42
4.41
4.41
4.40
4.39
4.39
4.38
4.38
4.37
4.30
4.29
4.27
4.26
4.21
4.19
4.18
4.17
4.16
4.14
4.13
4.04
4.03
2.45
2.44
2.43
1.27
1.25
1.24
0.98
1.00
1.00
1.06
1.08
1.14
2.00
1.00
2.05
3.12
f1 (ppm)

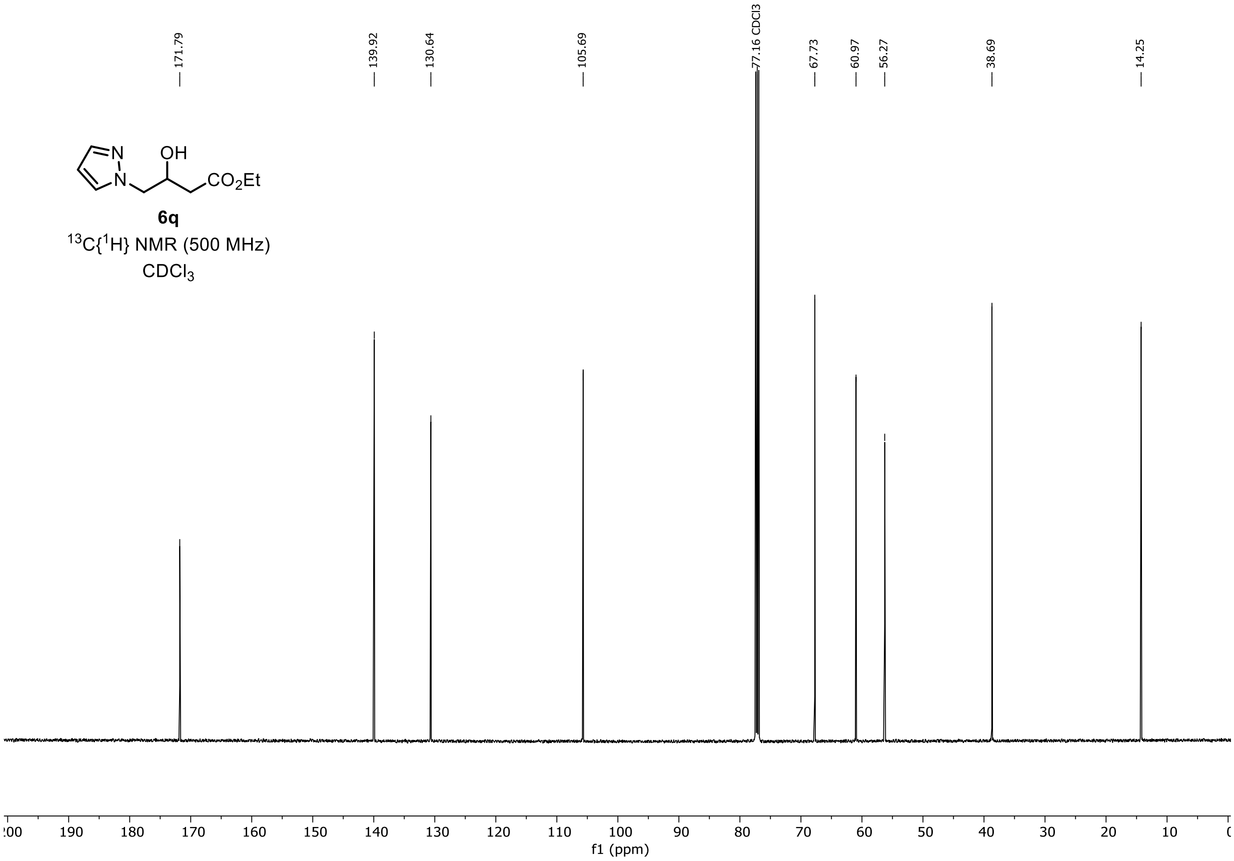
6q
$^{13}C\{^1H\}$ NMR (500 MHz)
$CDCl_3$
171.79
139.92
130.64
105.69
77.16 CDCl3
67.73
60.97
56.27
38.69
14.25
f1 (ppm)

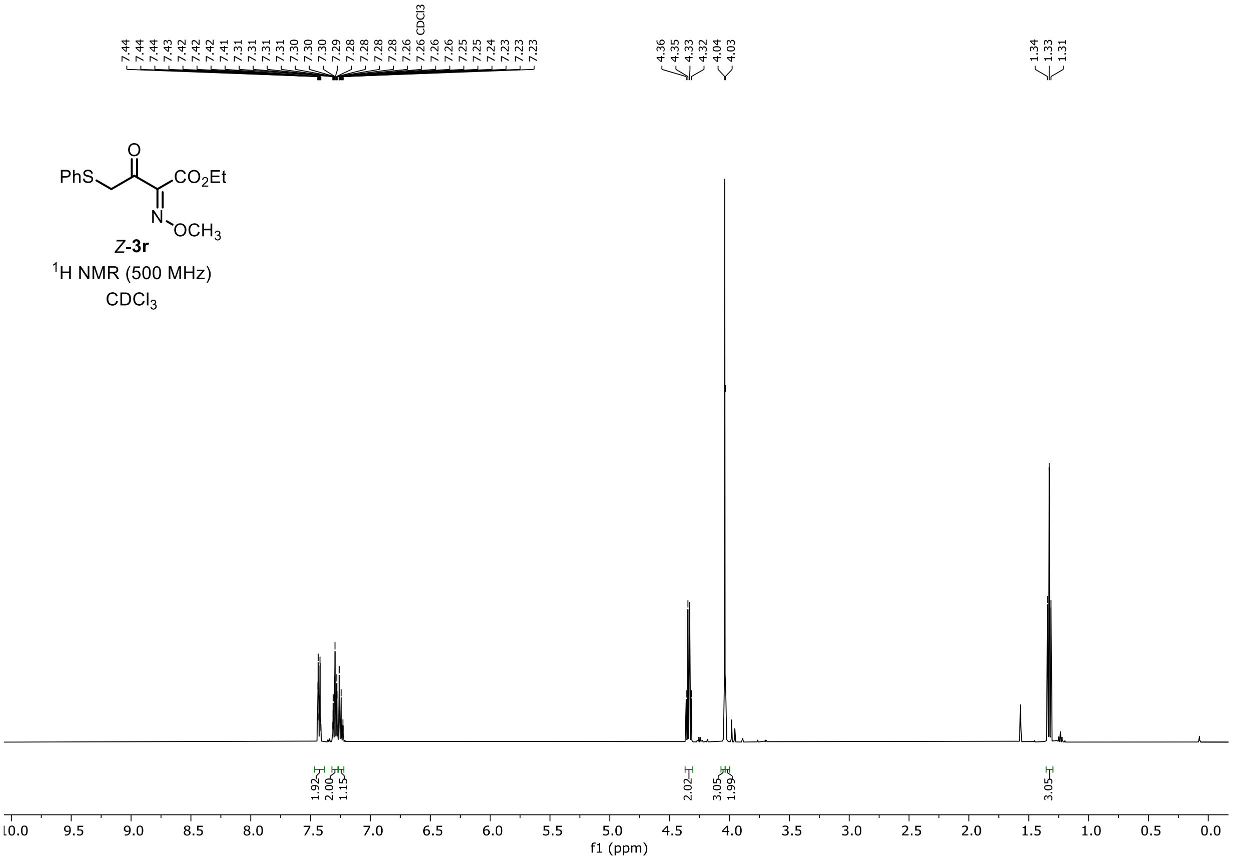
7.44 7.44 7.44 7.43 7.42 7.42 7.42 7.41 7.31 7.31 7.31 7.31 7.30 7.30 7.29 7.28 7.28 7.28 7.28 7.26 CDCl3 7.26 7.26 7.26 7.25 7.25 7.24 7.23 7.23 7.23
4.36 4.35 4.33 4.32 4.04 4.03
1.34 1.33 1.31
PhS
O
CO2Et
N
OCH3
Z-3r
1H NMR (500 MHz)
CDCl3
1.92 2.00 1.15
2.02
3.05 1.99
3.05
f1 (ppm)

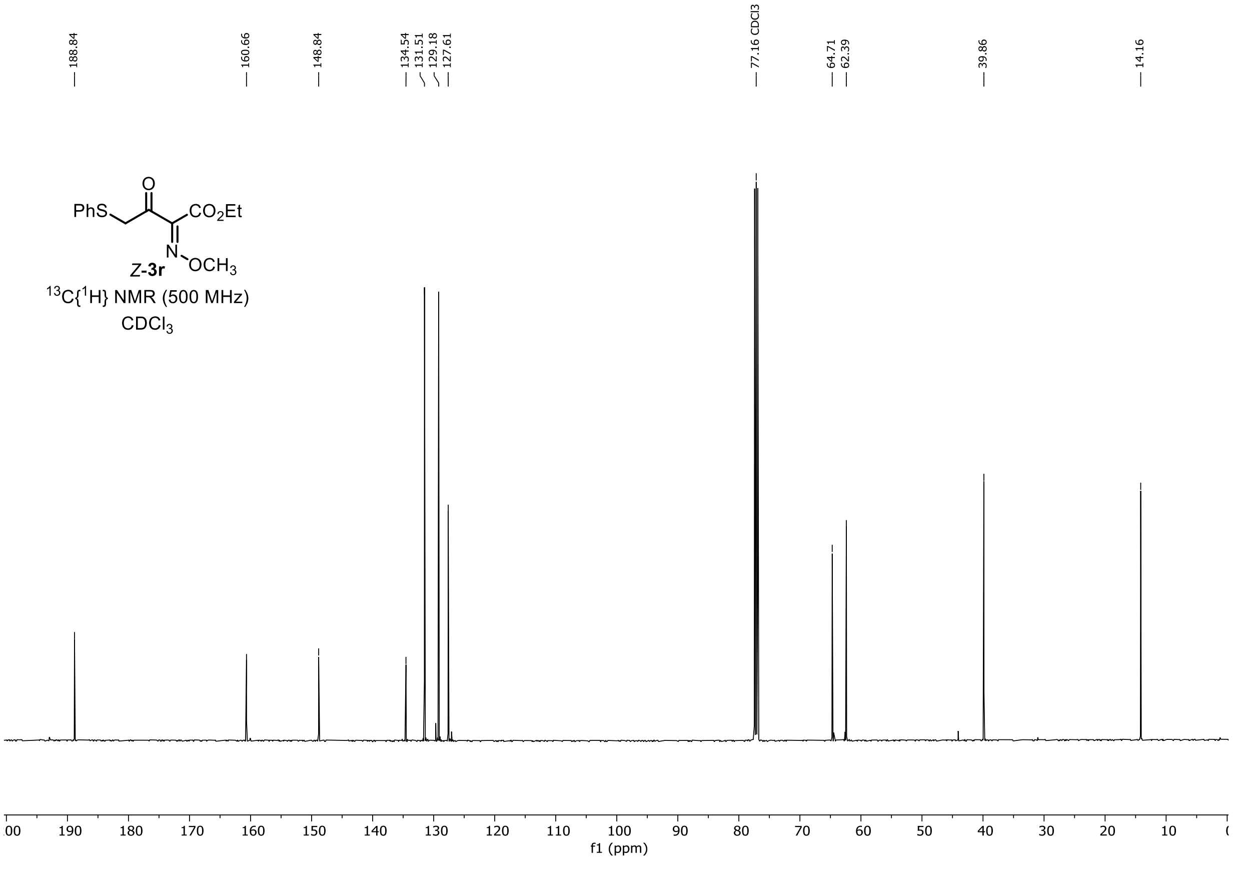
188.84
160.66
148.84
134.54 131.51 129.18 127.61
77.16 CDCl3
64.71 62.39
39.86
14.16
PhS
O
CO2Et
N
OCH3
Z-3r
13C{1H} NMR (500 MHz)
CDCl3
f1 (ppm)

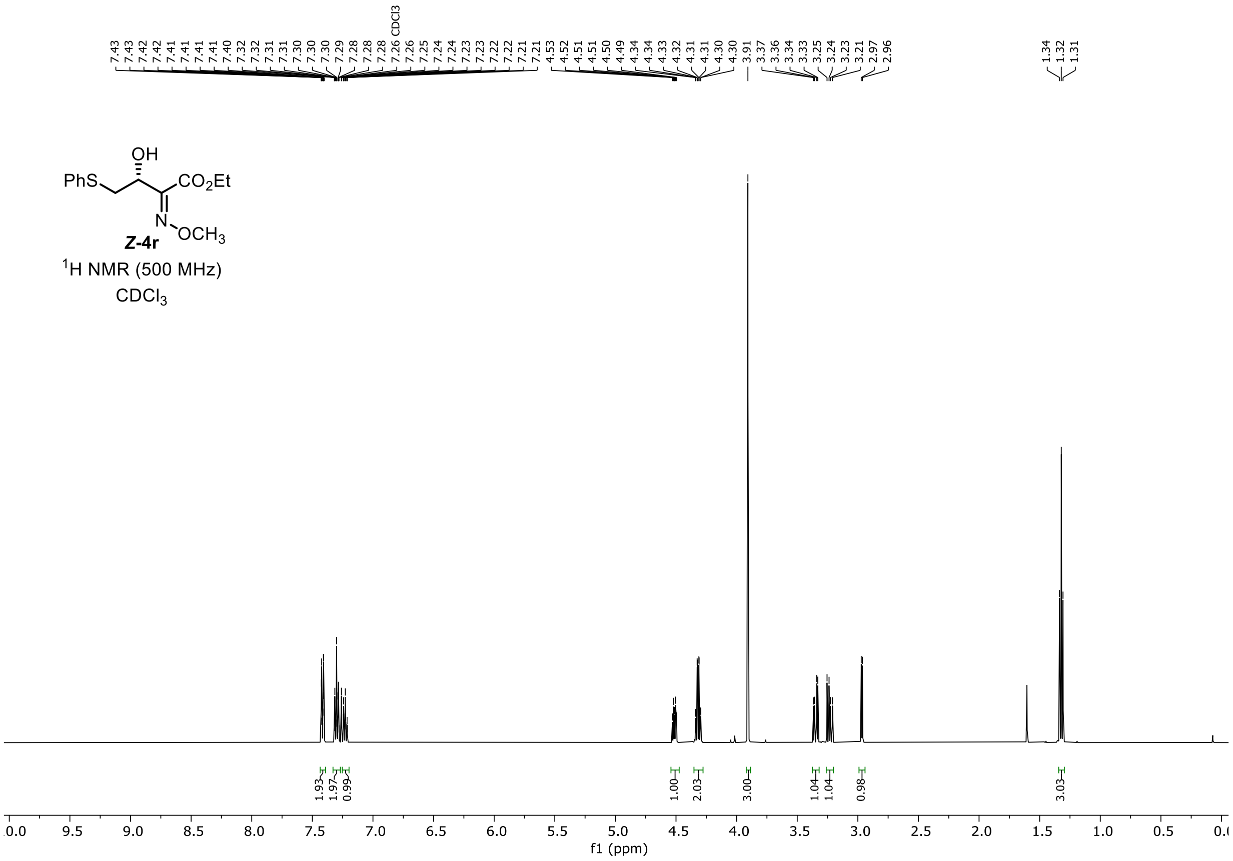
PhS
OH
CO2Et
N
OCH3
Z-4r
1H NMR (500 MHz)
CDCl3
f1 (ppm)

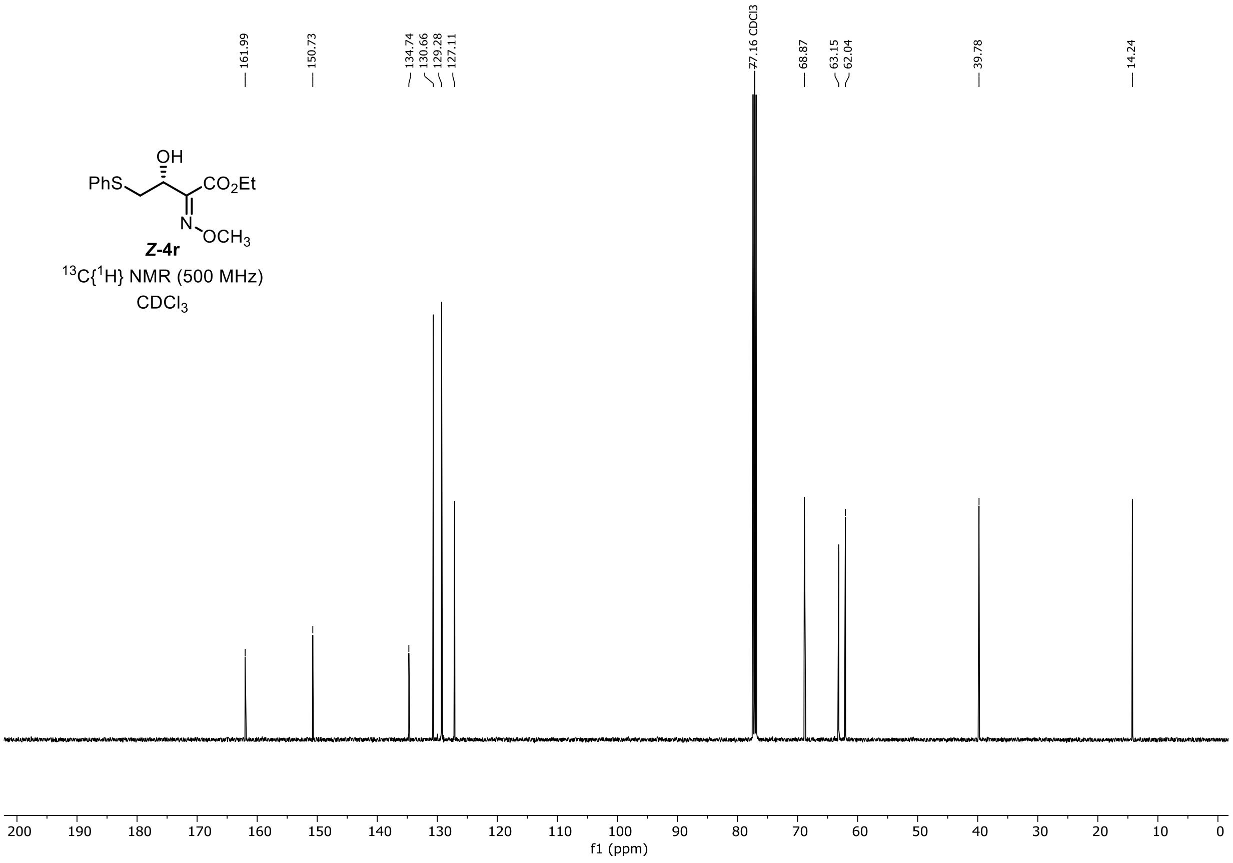
PhS
OH
CO2Et
N
OCH3
Z-4r
13C{1H} NMR (500 MHz)
CDCl3
f1 (ppm)

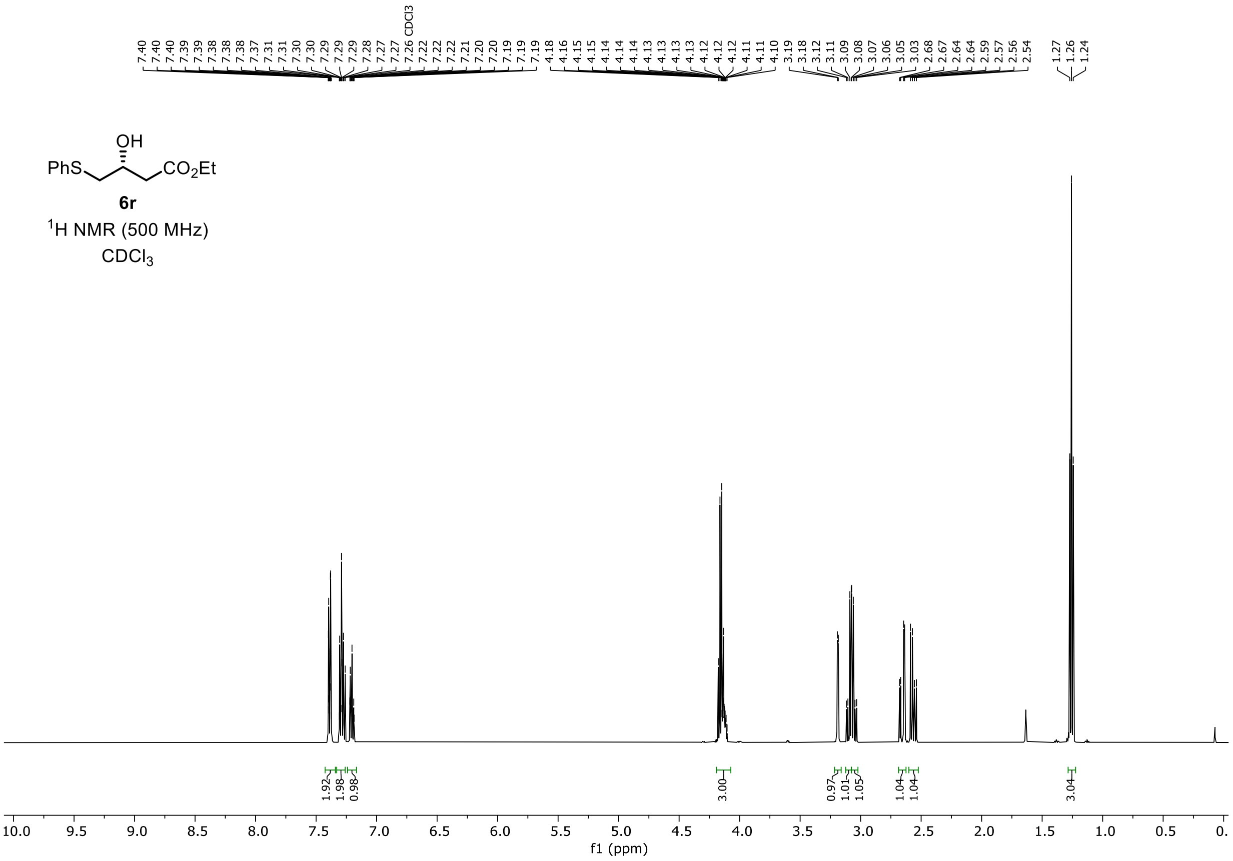
6r
^{1}H NMR (500 MHz)
$CDCl_3$
f1 (ppm)

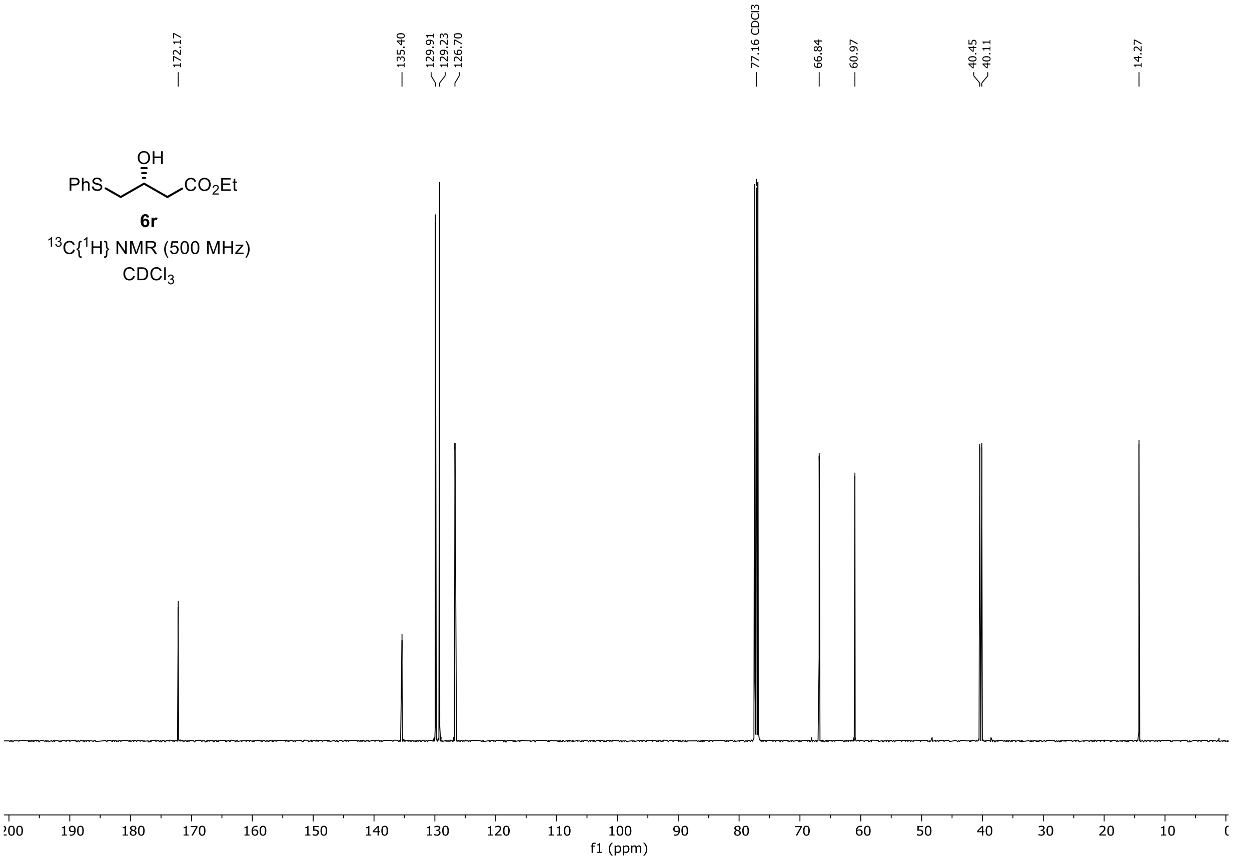
6r
$^{13}C\{^{1}H\}$ NMR (500 MHz)
$CDCl_3$
f1 (ppm)

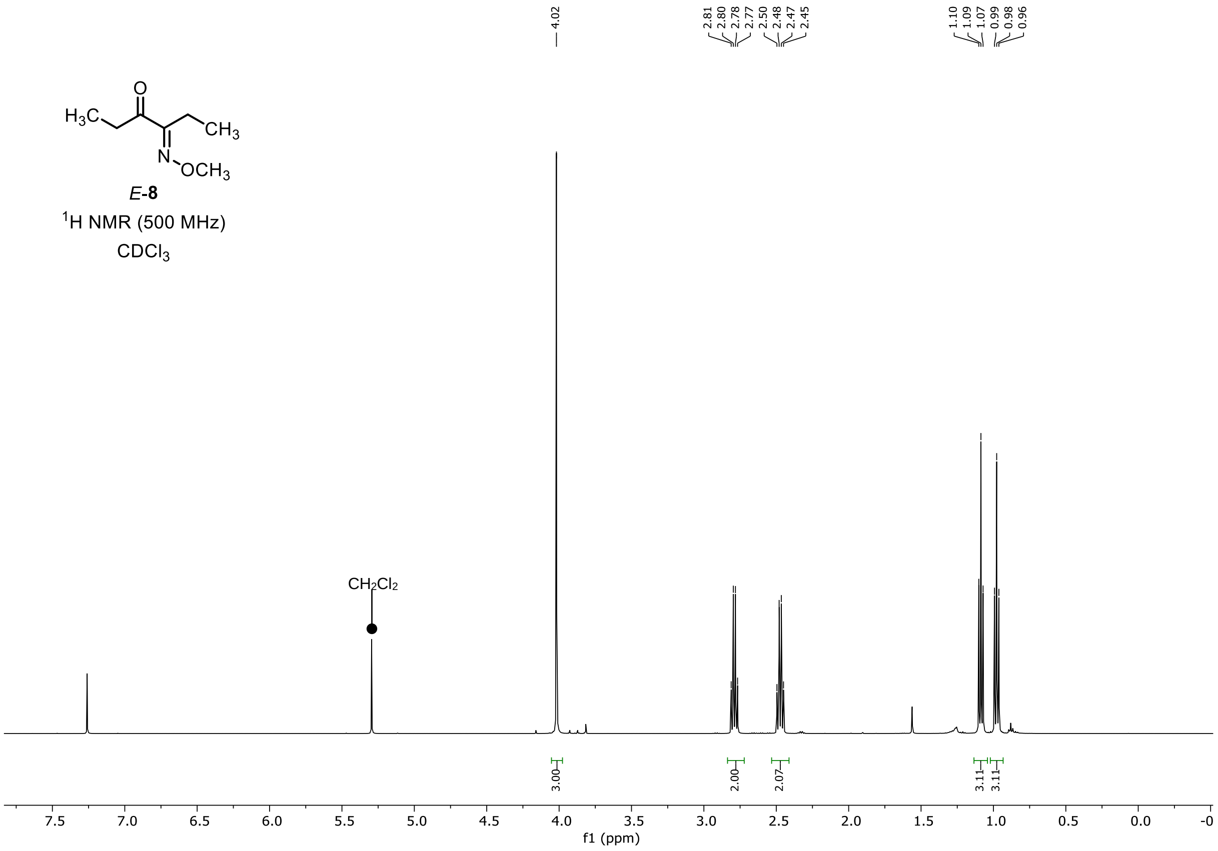

H3C CH3 O N OCH3
E-8
1H NMR (500 MHz)
CDCl3
CH2Cl2
4.02
2.81 2.80 2.78 2.77 2.50 2.48 2.47 2.45
1.10 1.09 1.07 0.99 0.98 0.96
3.00 2.00 2.07 3.11 3.11
f1 (ppm)

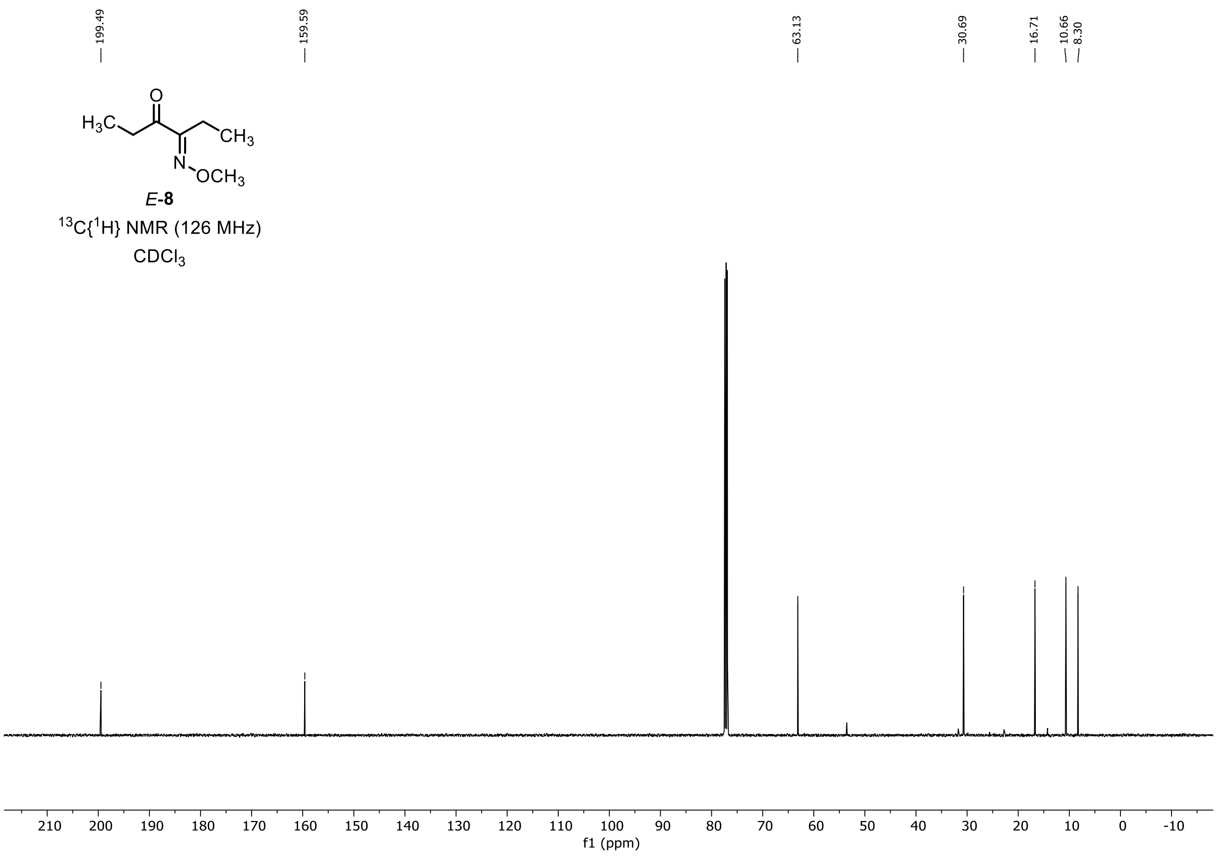

199.49
159.59
63.13
30.69
16.71
10.66
8.30
H3C CH3 O N OCH3
E-8
13C{1H} NMR (126 MHz)
CDCl3
f1 (ppm)

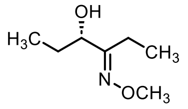

9 (major shown)
8:1 mixture of *Z/E* isomers
^{1}H NMR (500 MHz)
$CDCl_3$

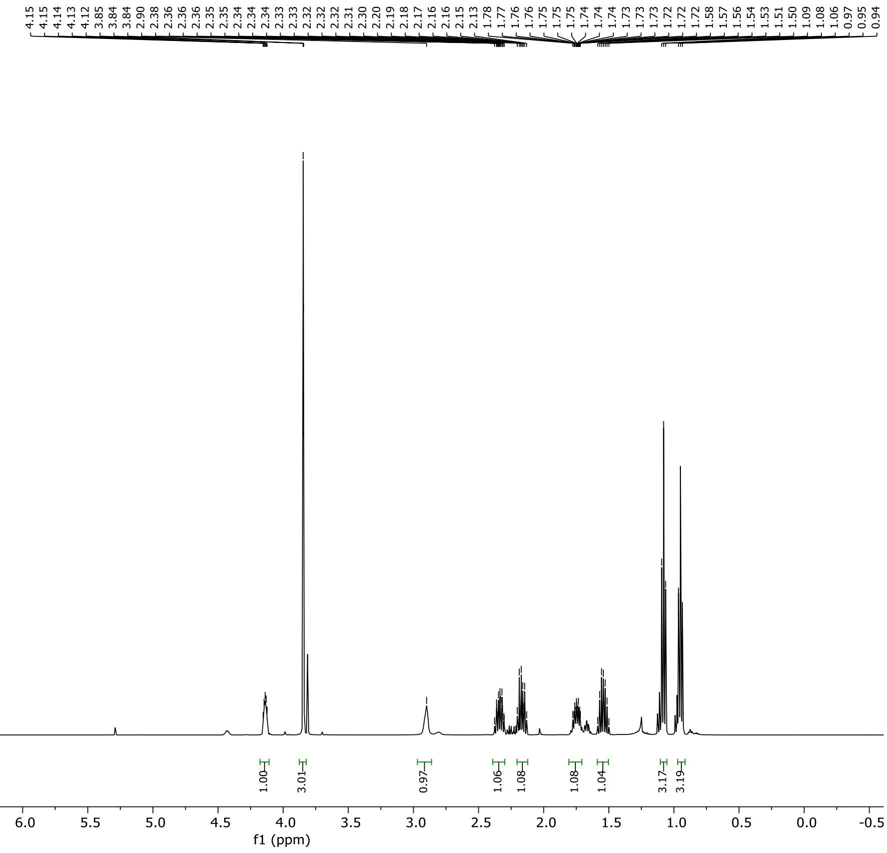

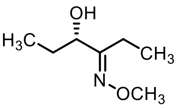

9 (major shown)
8:1 mixture of *Z/E* isomers
$^{13}C\{^{1}H\}$ NMR (126 MHz)
$CDCl_3$

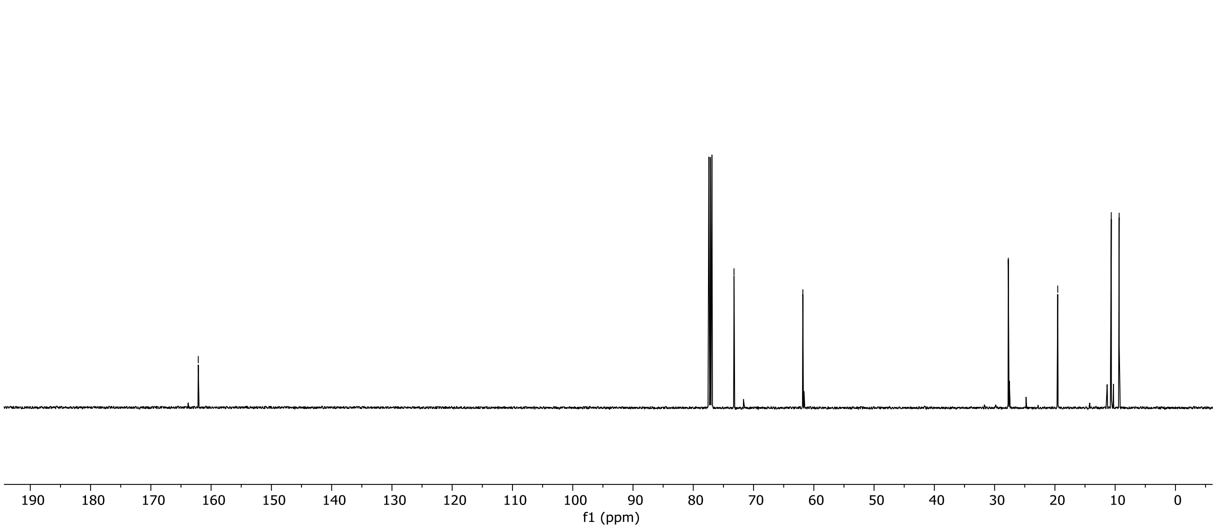

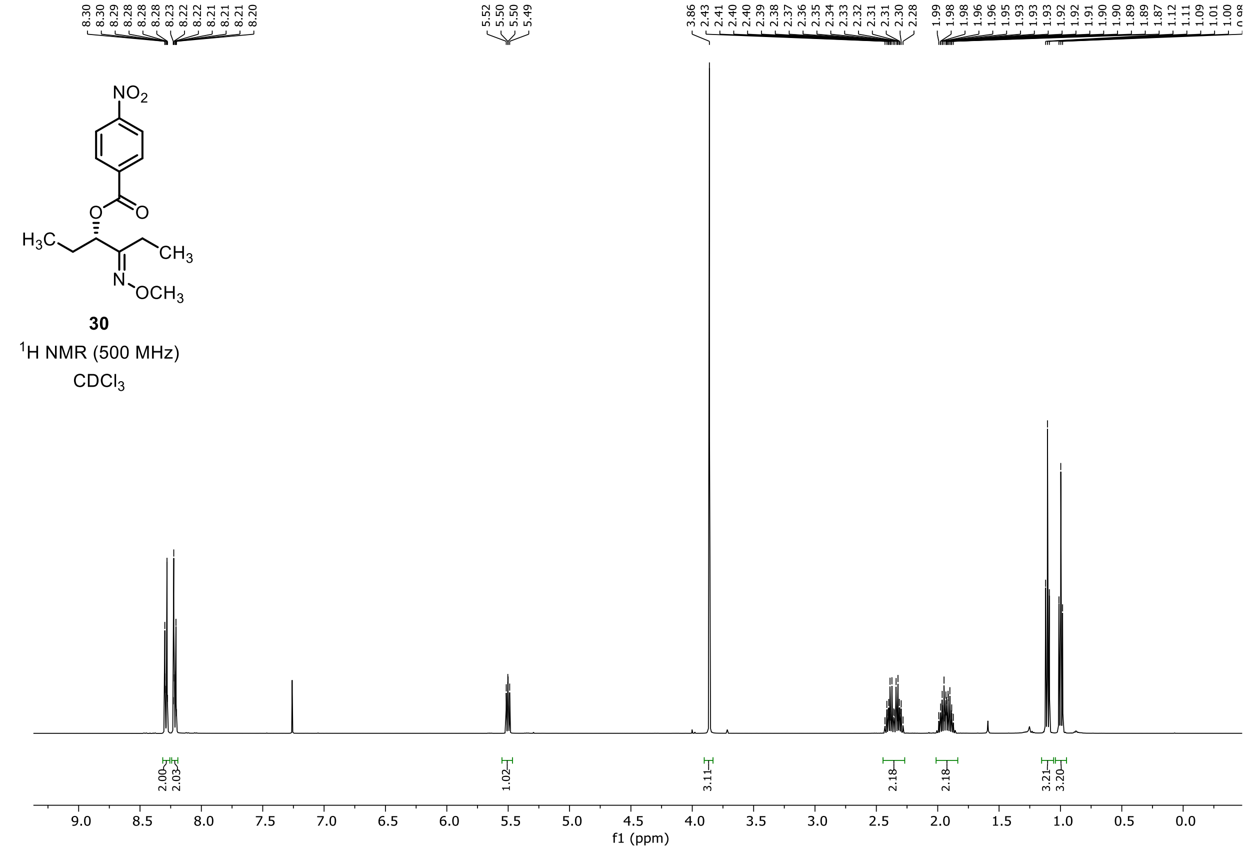

30

^{1}H NMR (500 MHz)

$CDCl_3$

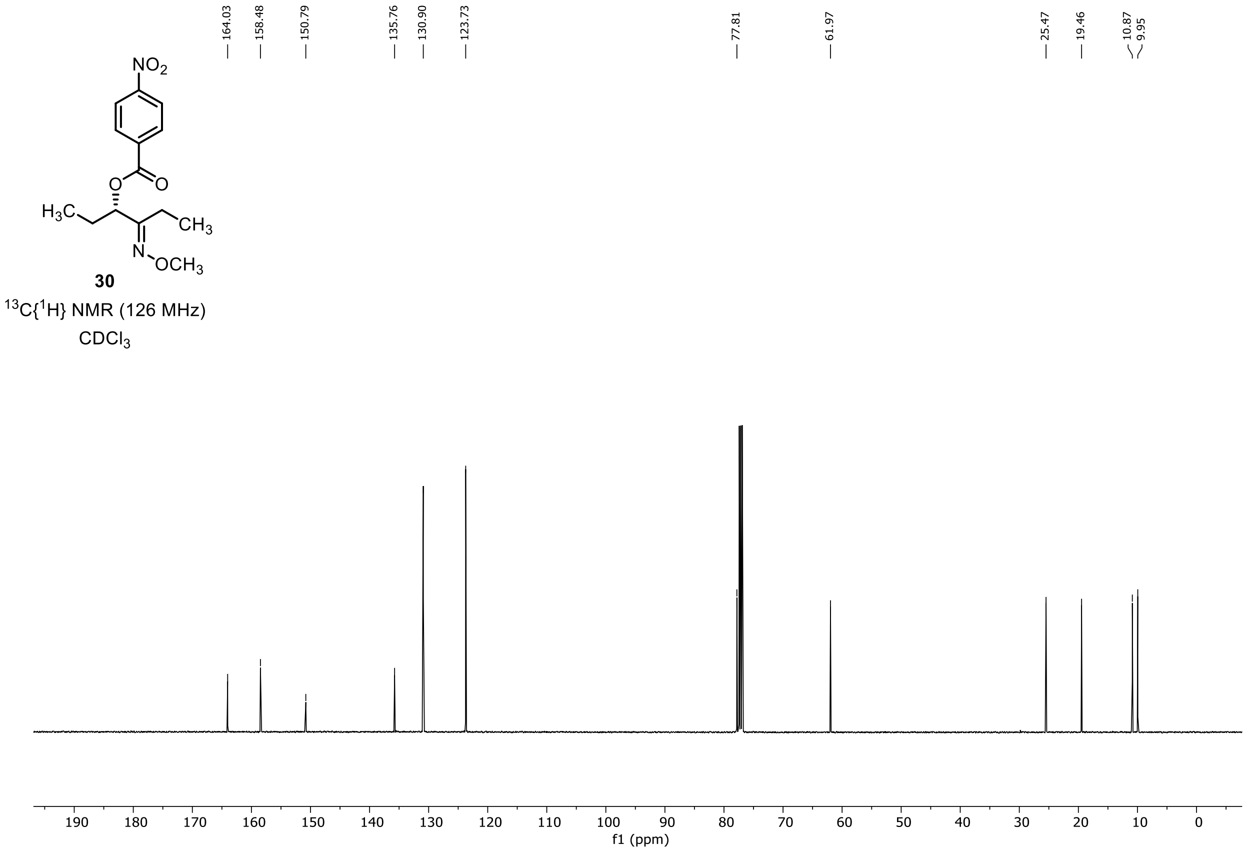

30

$^{13}C\{^{1}H\}$ NMR (126 MHz)

$CDCl_3$

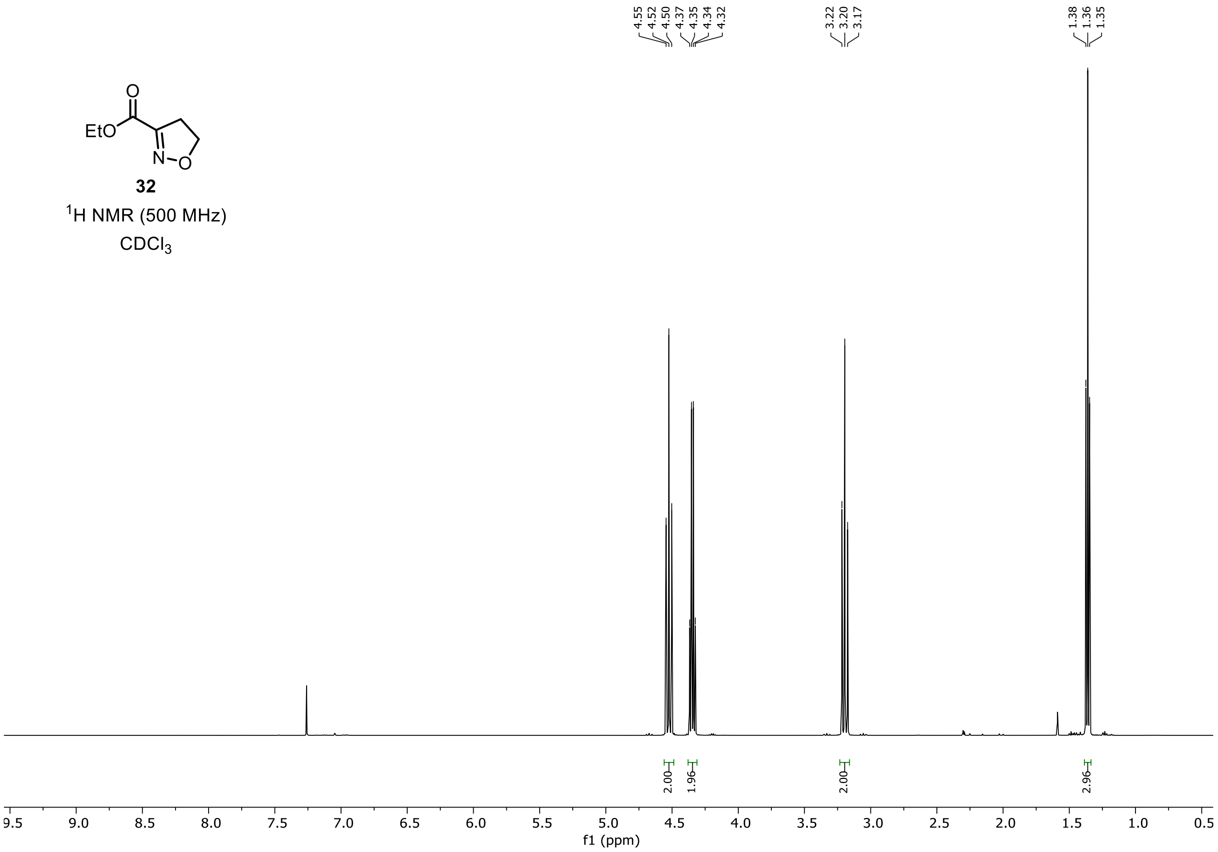
EtO
O
N
O
32
1H NMR (500 MHz)
CDCl3
4.55
4.52
4.50
4.37
4.35
4.34
4.32
3.22
3.20
3.17
1.38
1.36
1.35
2.00
1.96
2.00
2.96
9.5 9.0 8.5 8.0 7.5 7.0 6.5 6.0 5.5 5.0 4.5 4.0 3.5 3.0 2.5 2.0 1.5 1.0 0.5
f1 (ppm)

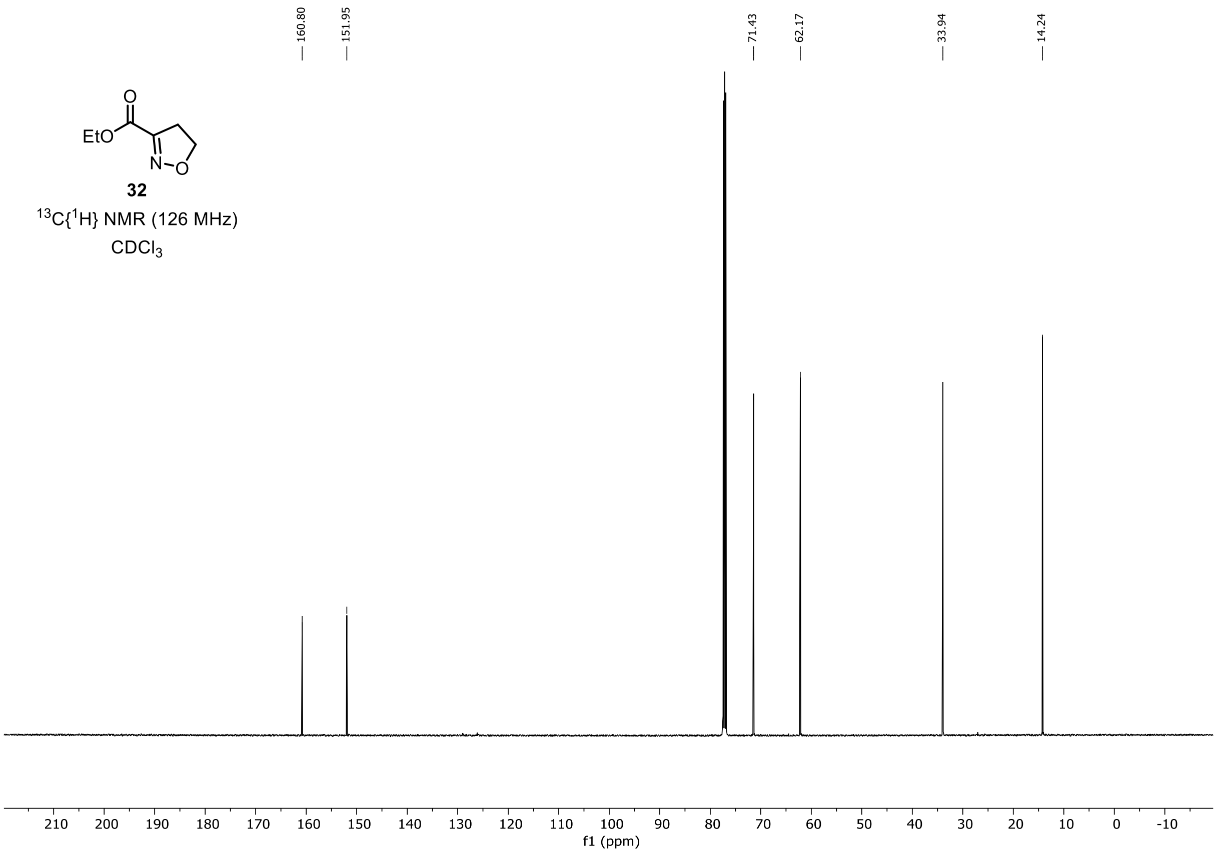
EtO
O
N
O
32
13C{1H} NMR (126 MHz)
CDCl3
160.80
151.95
71.43
62.17
33.94
14.24
210 200 190 180 170 160 150 140 130 120 110 100 90 80 70 60 50 40 30 20 10 0 -10
f1 (ppm)

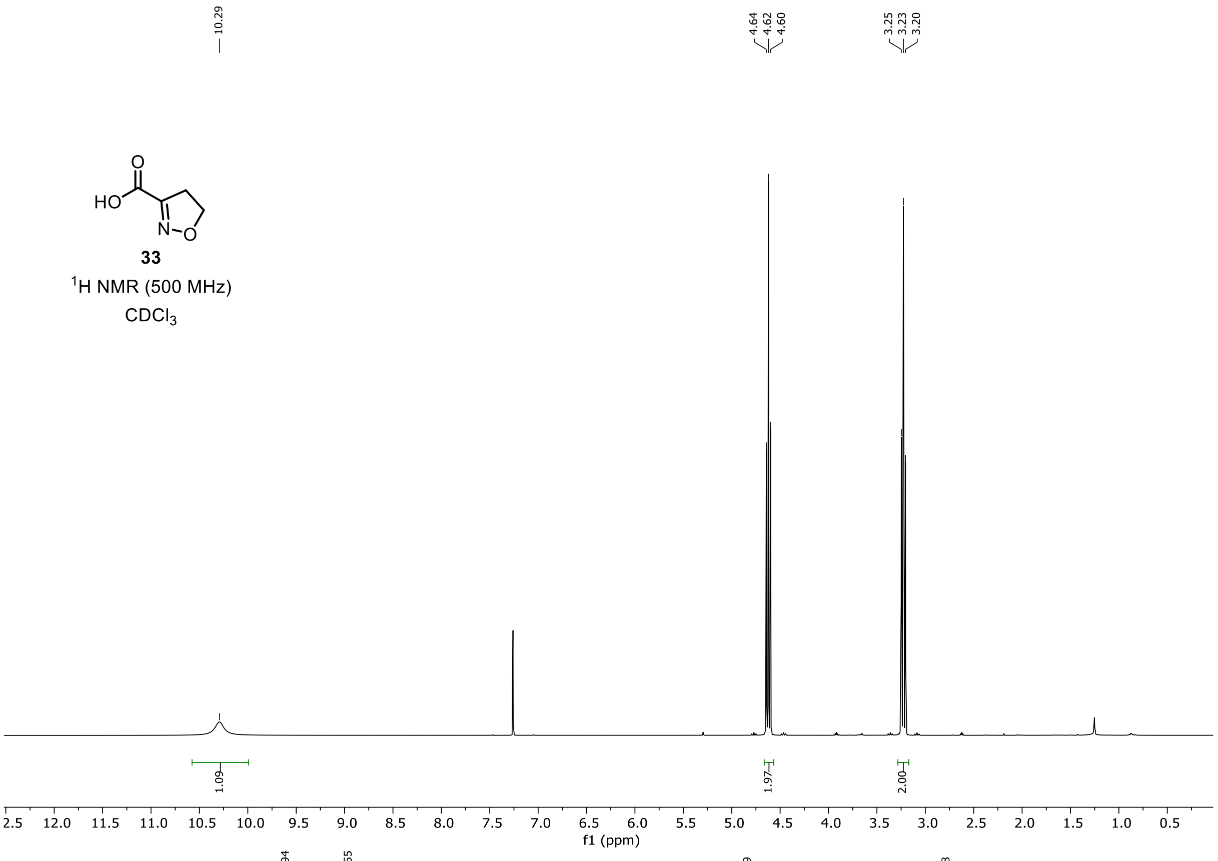
10.29
4.64
4.62
4.60
3.25
3.23
3.20
HO
O
N
O
33
^{1}H NMR (500 MHz)
$CDCl_3$
1.09
1.97
2.00
f1 (ppm)

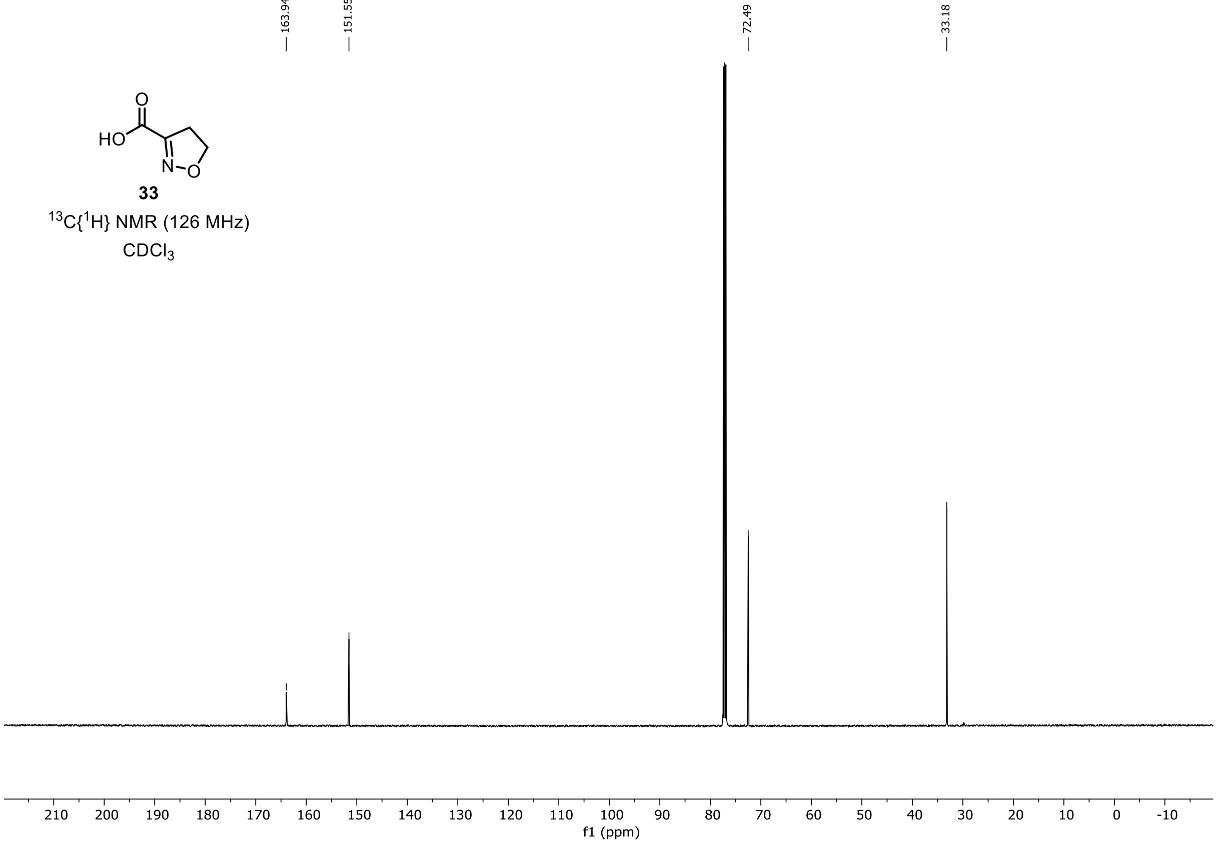
163.94
151.55
72.49
33.18
HO
O
N
O
33
^{13}C{^{1}H} NMR (126 MHz)
$CDCl_3$
f1 (ppm)

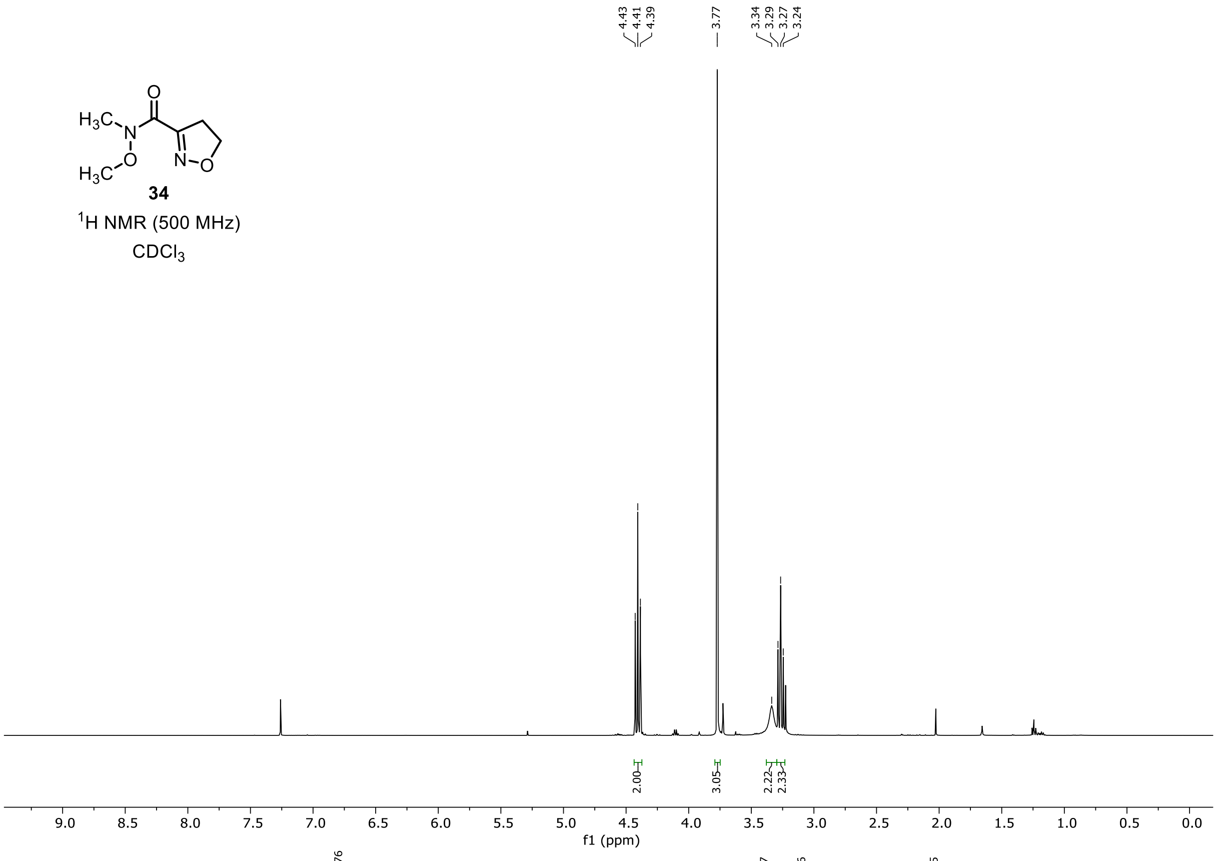
4.43
4.41
4.39
3.77
3.34
3.29
3.27
3.24
34
^{1}H NMR (500 MHz)
$CDCl_3$
2.00
3.05
2.22
2.33
f1 (ppm)

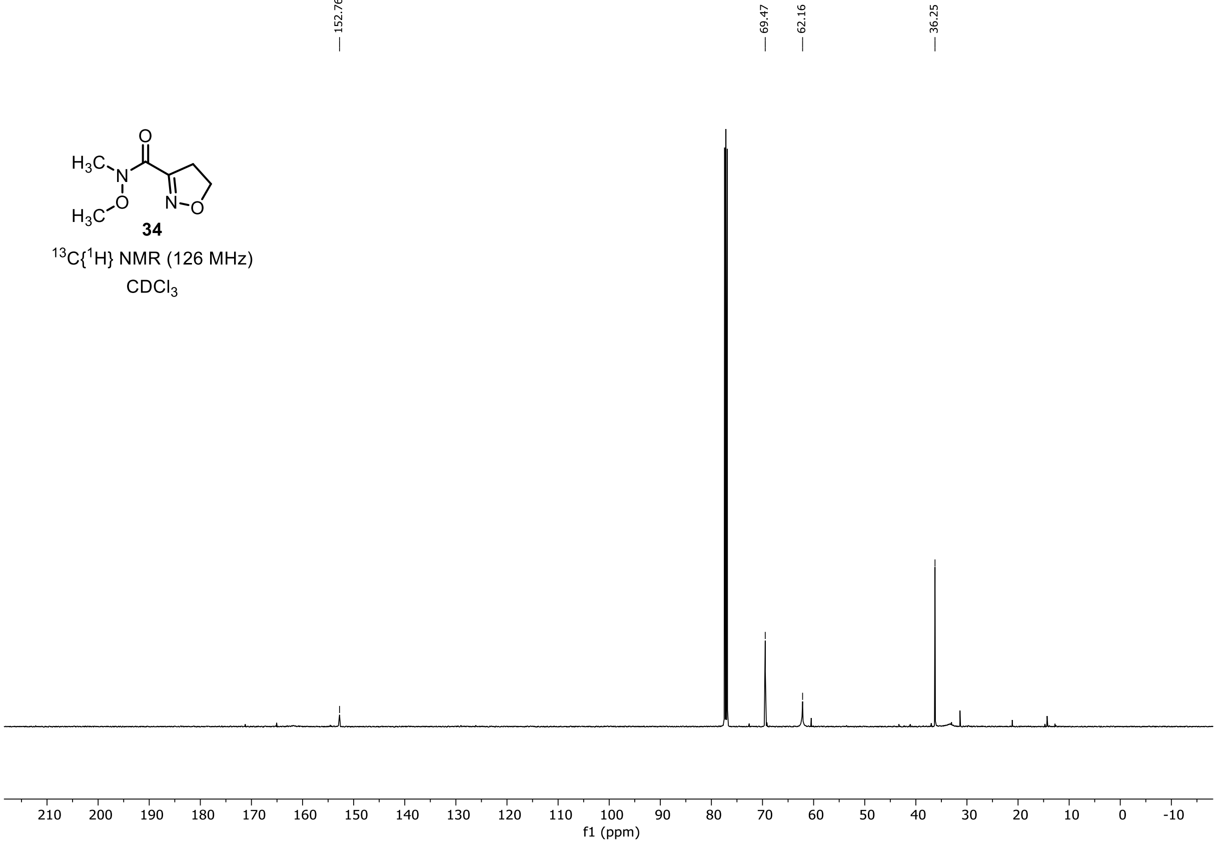
152.76
69.47
62.16
36.25
34
$^{13}C\{^1H\}$ NMR (126 MHz)
$CDCl_3$
f1 (ppm)

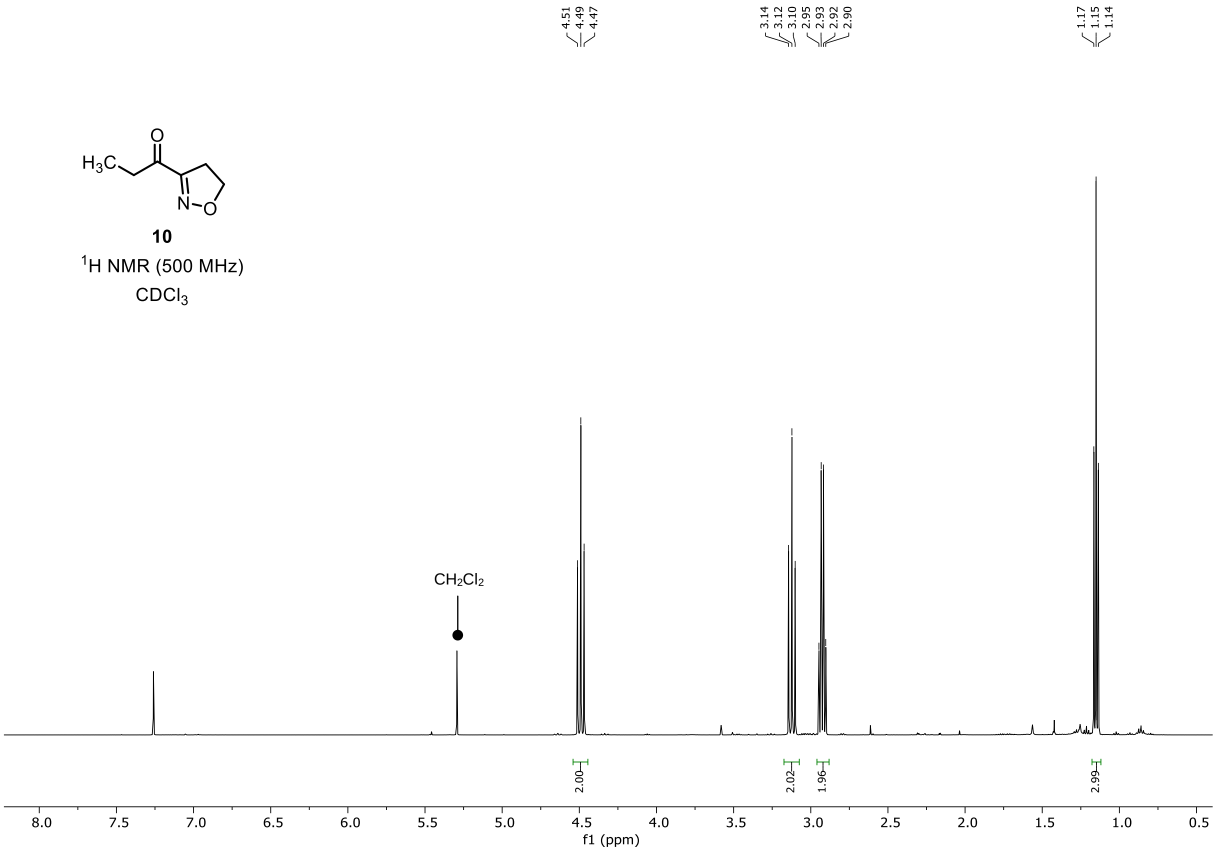
4.51
4.49
4.47
3.14
3.12
3.10
2.95
2.93
2.92
2.90
1.17
1.15
1.14
O
H3C
N-O
10
1H NMR (500 MHz)
CDCl3
CH2Cl2
2.00
2.02
1.96
2.99
8.0 7.5 7.0 6.5 6.0 5.5 5.0 4.5 4.0 3.5 3.0 2.5 2.0 1.5 1.0 0.5
f1 (ppm)

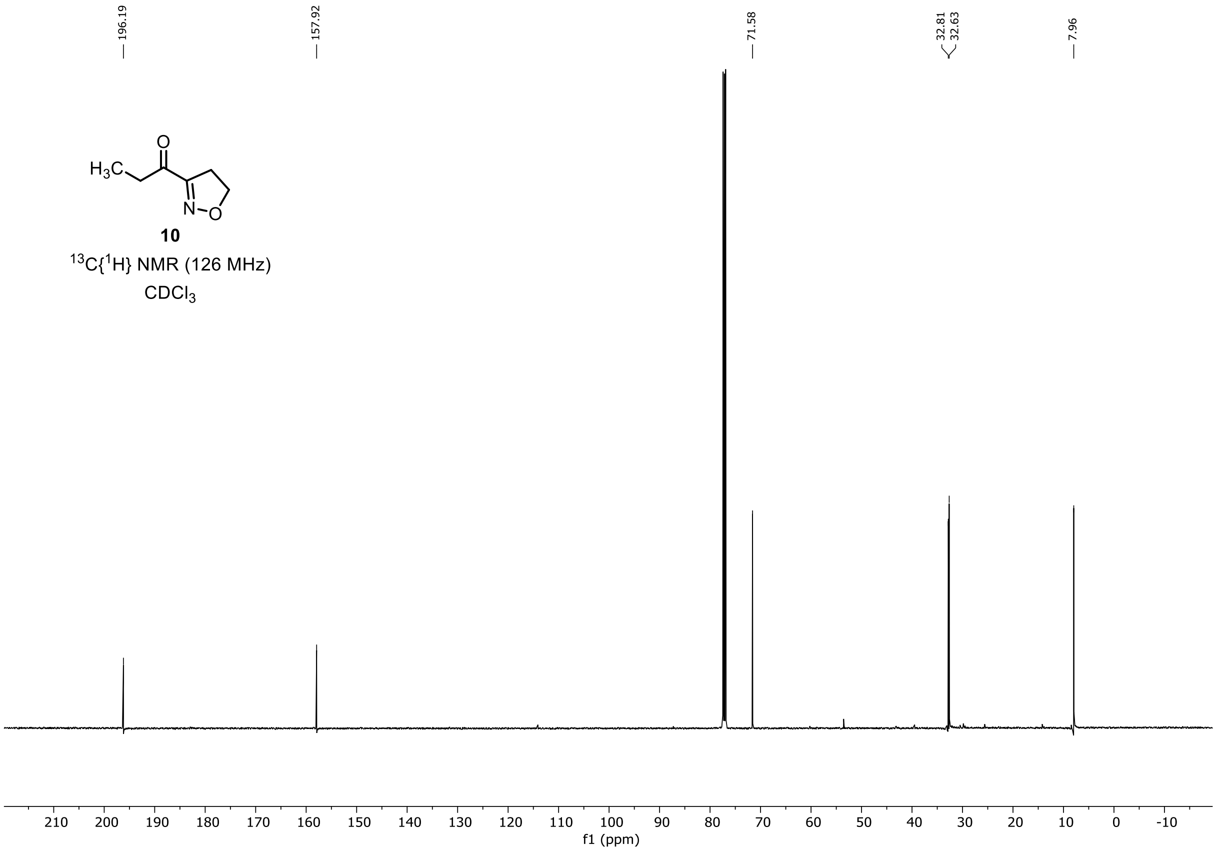
196.19
157.92
71.58
32.81
32.63
7.96
O
H3C
N-O
10
13C{1H} NMR (126 MHz)
CDCl3
210 200 190 180 170 160 150 140 130 120 110 100 90 80 70 60 50 40 30 20 10 0 -10
f1 (ppm)

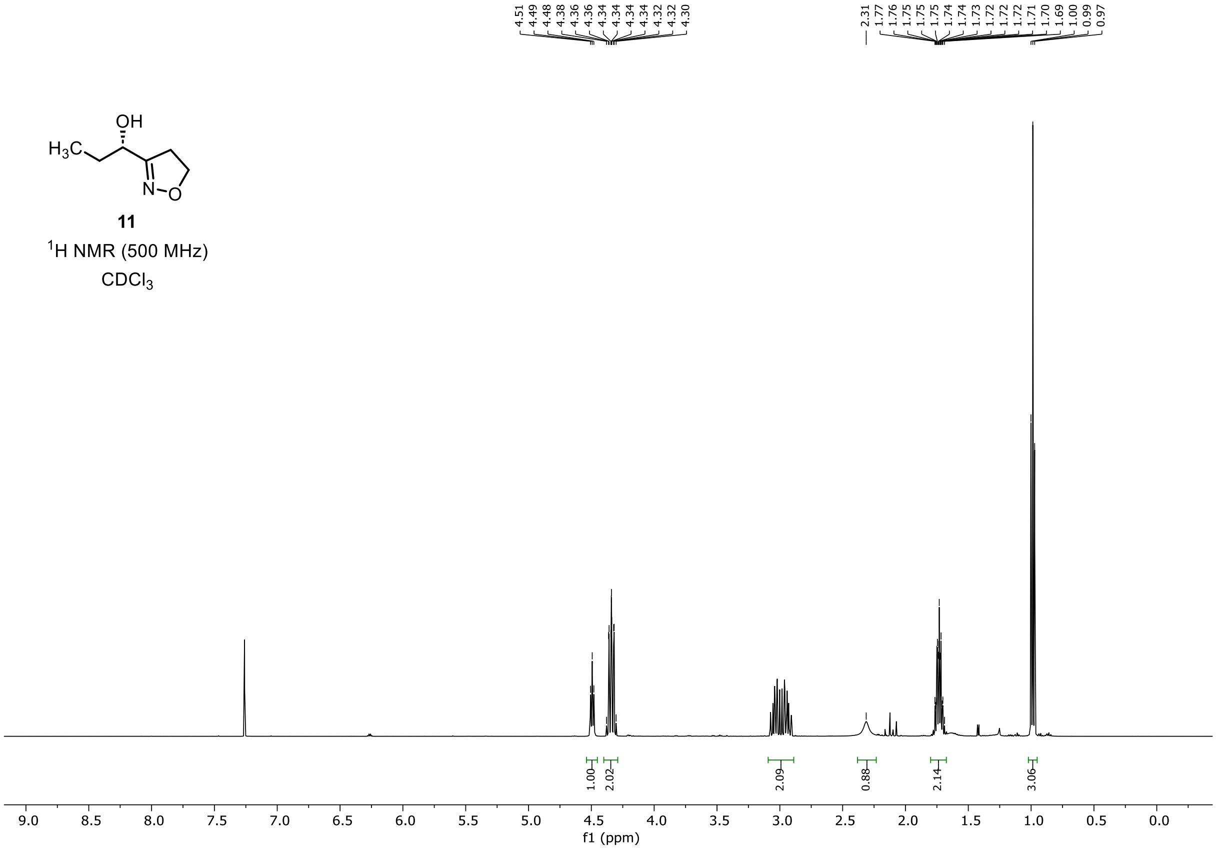

11

^{1}H NMR (500 MHz)

$CDCl_3$

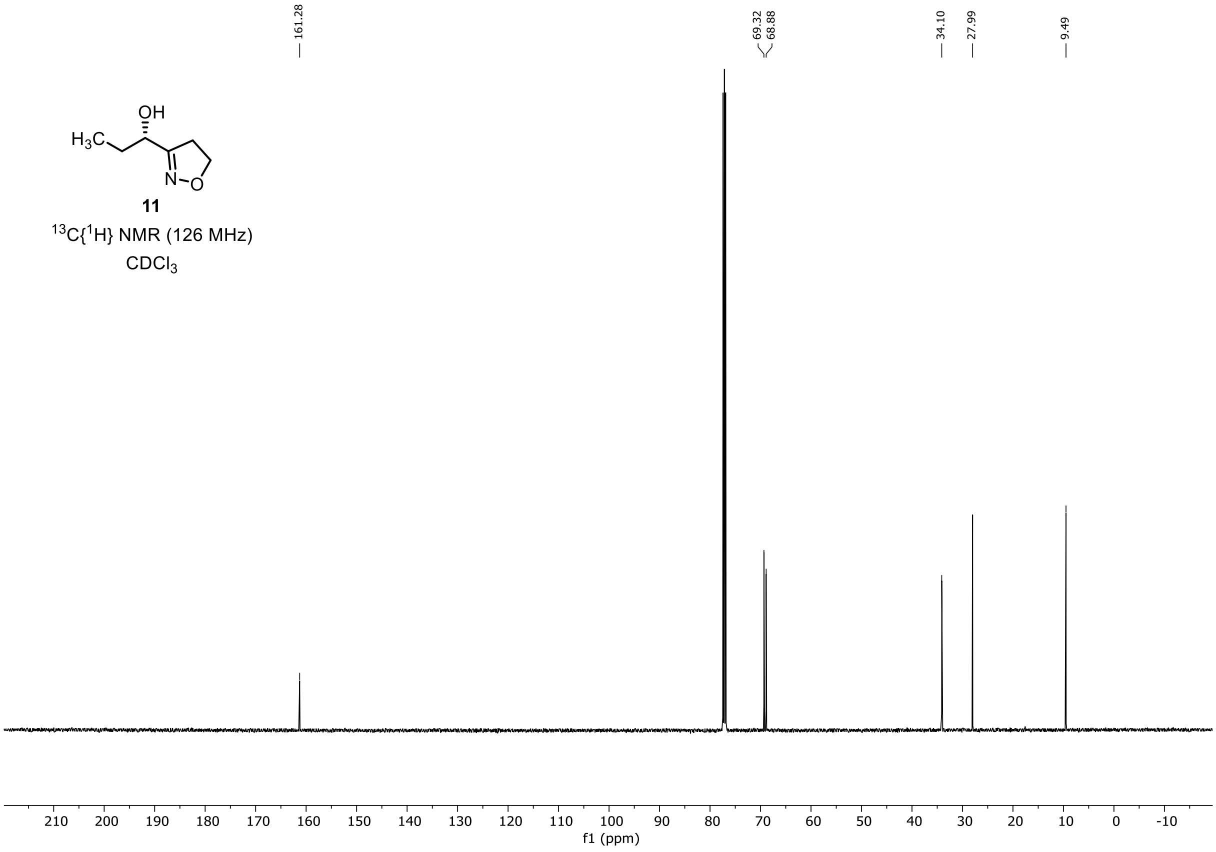

11

^{13}C{^{1}H} NMR (126 MHz)

$CDCl_3$

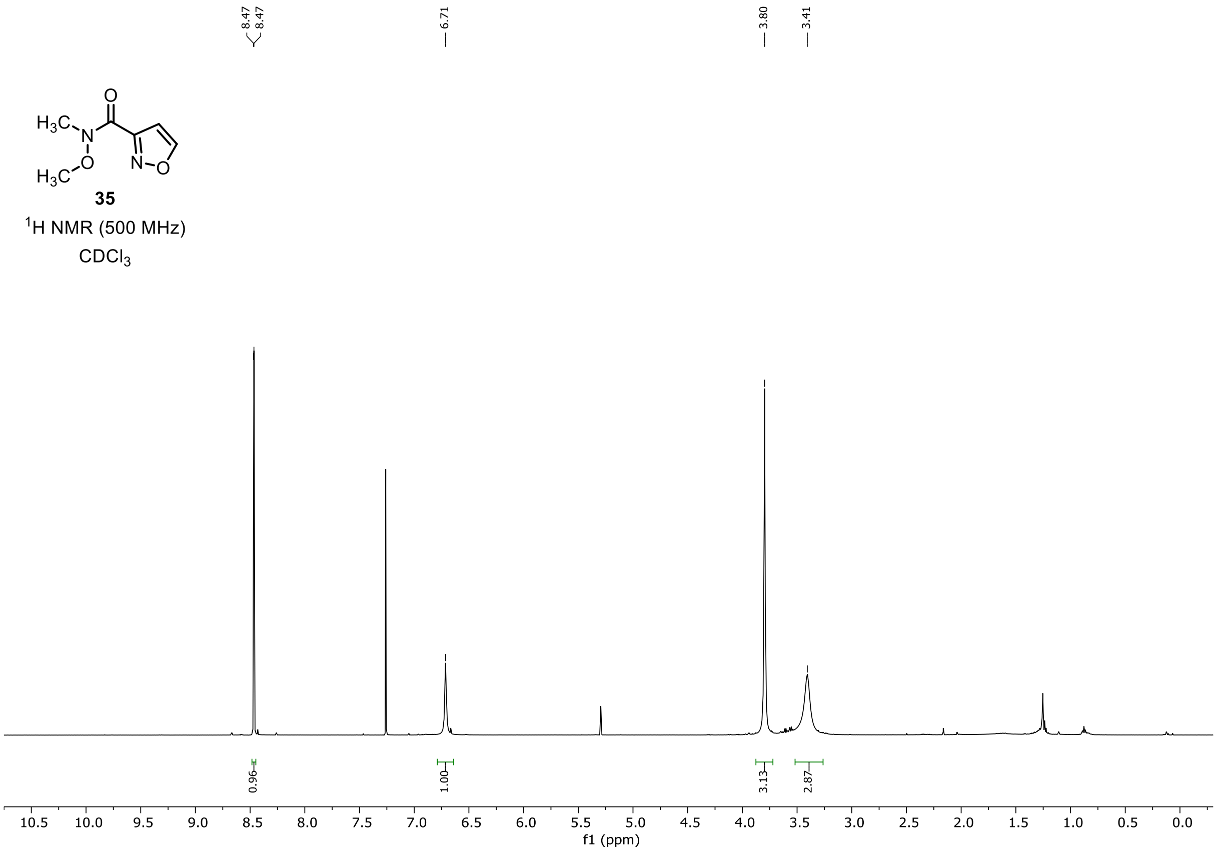
8.47
8.47
6.71
3.80
3.41
35
1H NMR (500 MHz)
CDCl3
0.96
1.00
3.13
2.87
10.5 10.0 9.5 9.0 8.5 8.0 7.5 7.0 6.5 6.0 5.5 5.0 4.5 4.0 3.5 3.0 2.5 2.0 1.5 1.0 0.5 0.0
f1 (ppm)

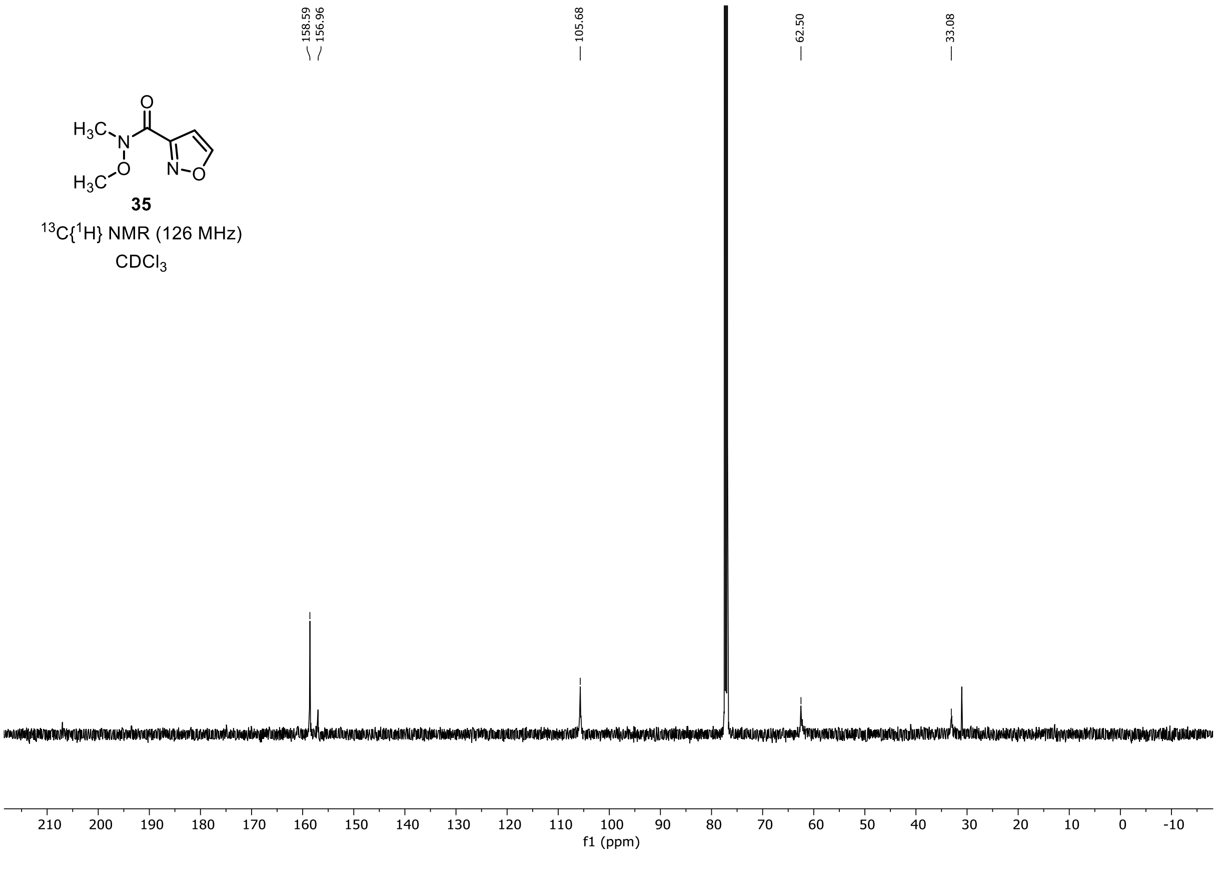
158.59
156.96
105.68
62.50
33.08
35
13C{1H} NMR (126 MHz)
CDCl3
210 200 190 180 170 160 150 140 130 120 110 100 90 80 70 60 50 40 30 20 10 0 -10
f1 (ppm)

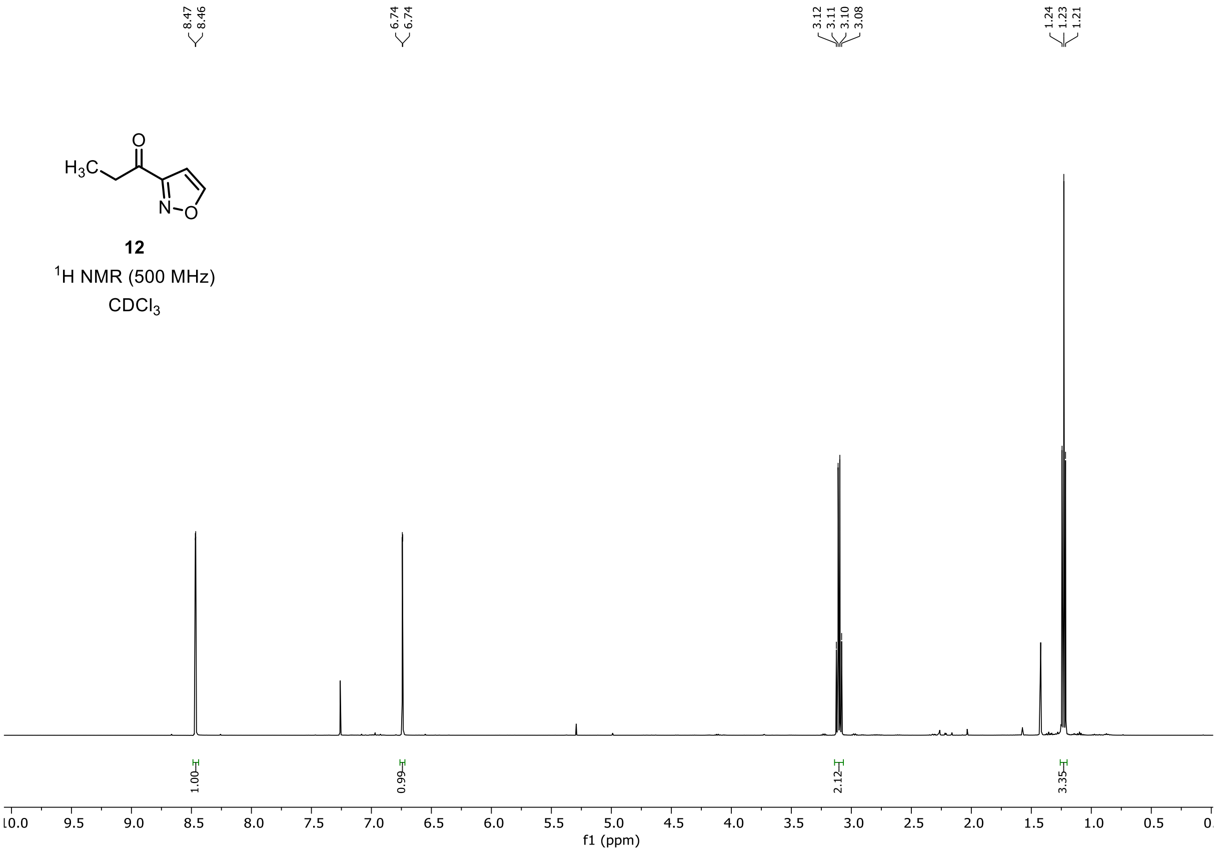
8.47
8.46
6.74
6.74
3.12
3.11
3.10
3.08
1.24
1.23
1.21
H3C
O
N
O
12
1H NMR (500 MHz)
CDCl3
1.00
0.99
2.12
3.35
f1 (ppm)

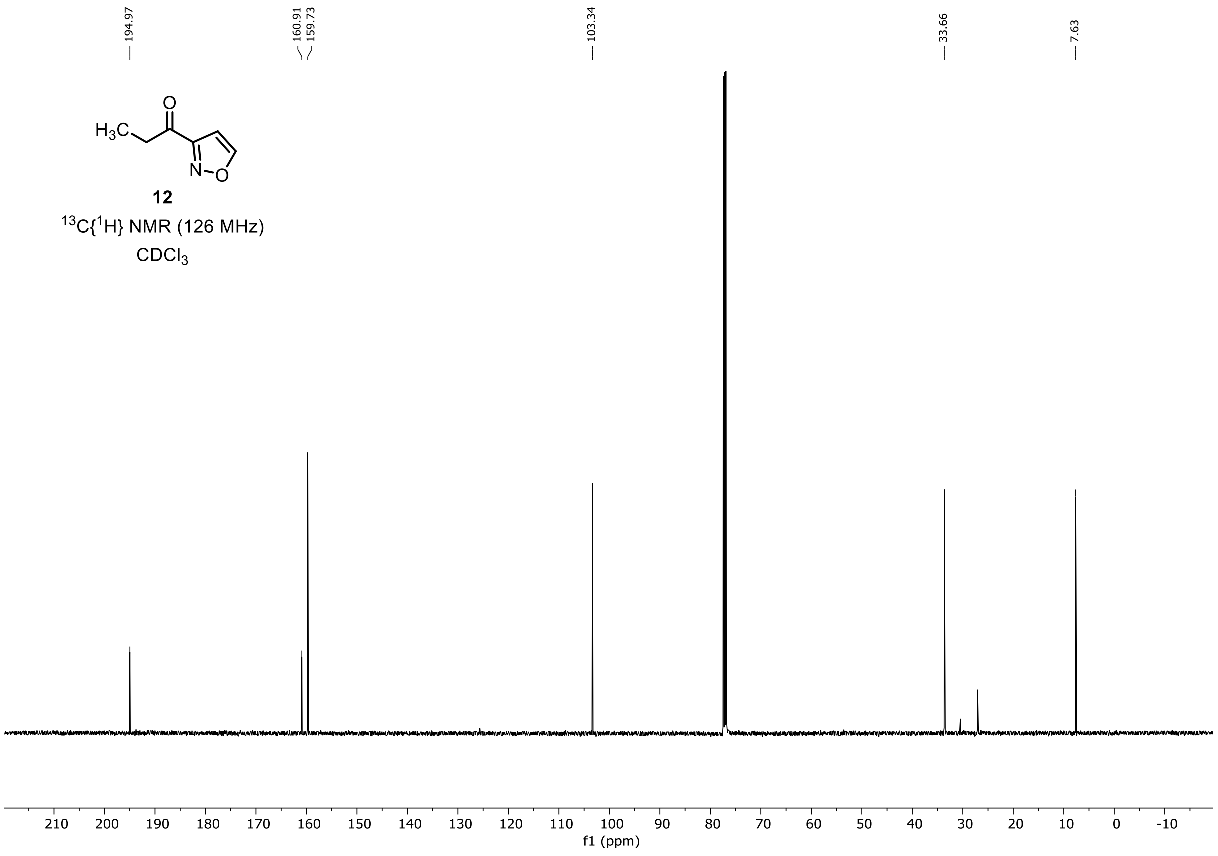
194.97
160.91
159.73
103.34
33.66
7.63
H3C
O
N
O
12
13C{1H} NMR (126 MHz)
CDCl3
f1 (ppm)

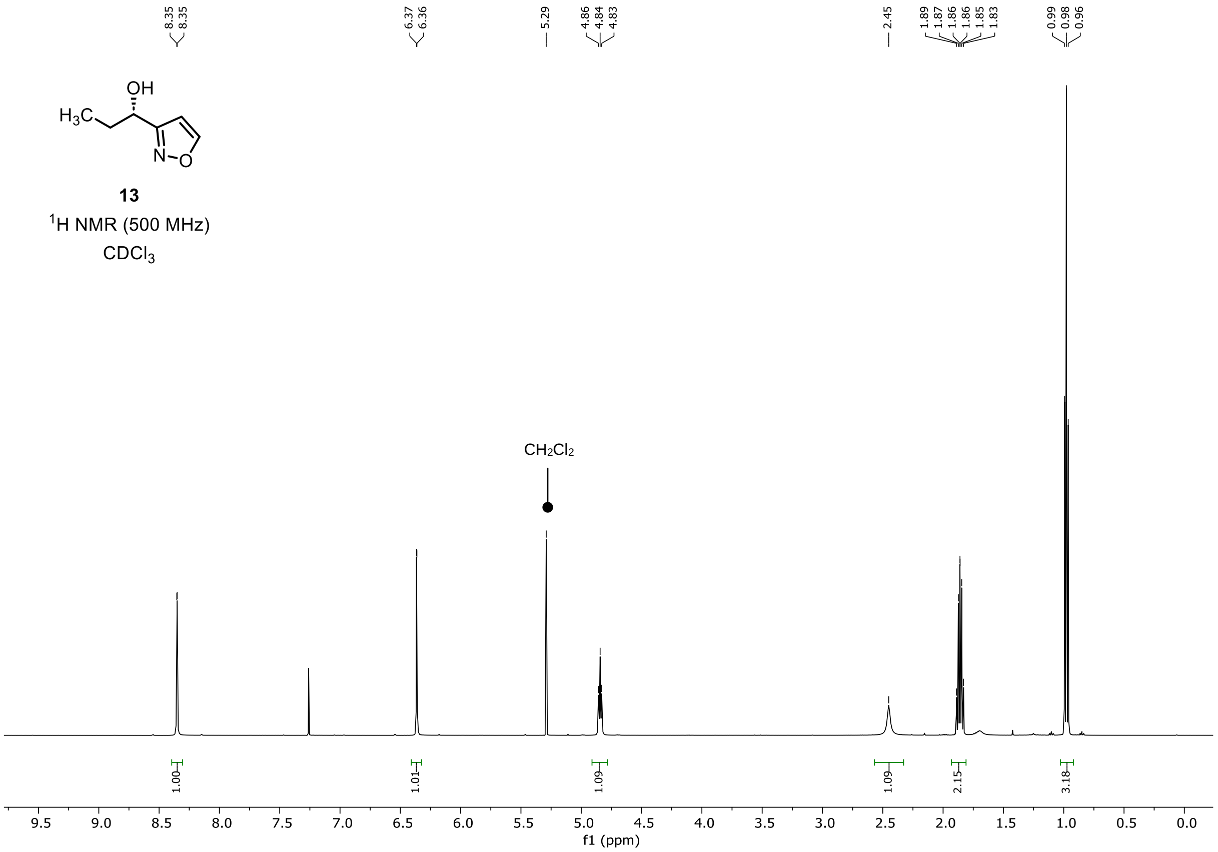

13

^{1}H NMR (500 MHz)

$CDCl_3$

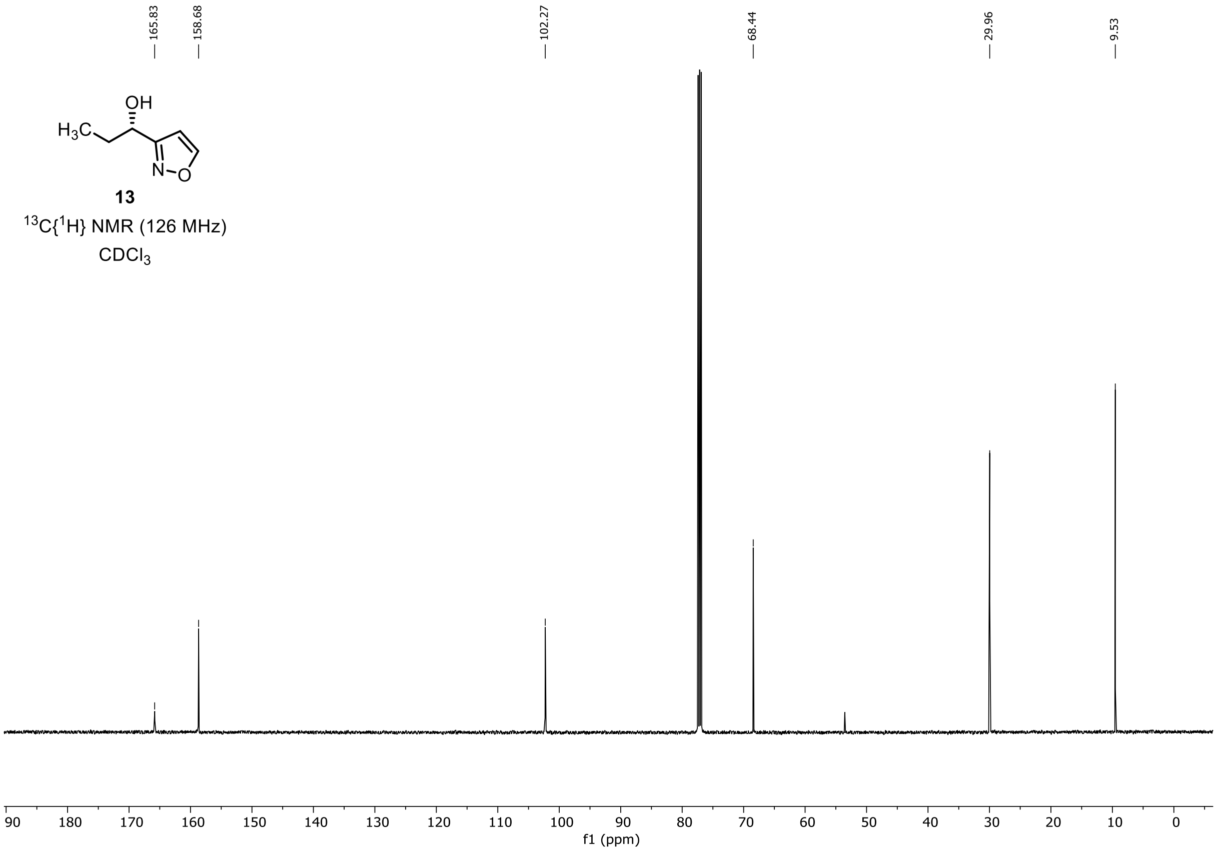

13

^{13}C{^{1}H} NMR (126 MHz)

$CDCl_3$

Copies of HPLC and GC chromatograms

HPLC analyses of racemic and enantiomerically enriched 4a

Chiralpak IA column, 250 × 4.6 mm, λ = 254 nm, 1% 2-propanol in hexane, flow rate: 1.0 mL/min

Figure S5. HPLC chromatogram of (±)-**4a** (only the major oxime isomer was analyzed)

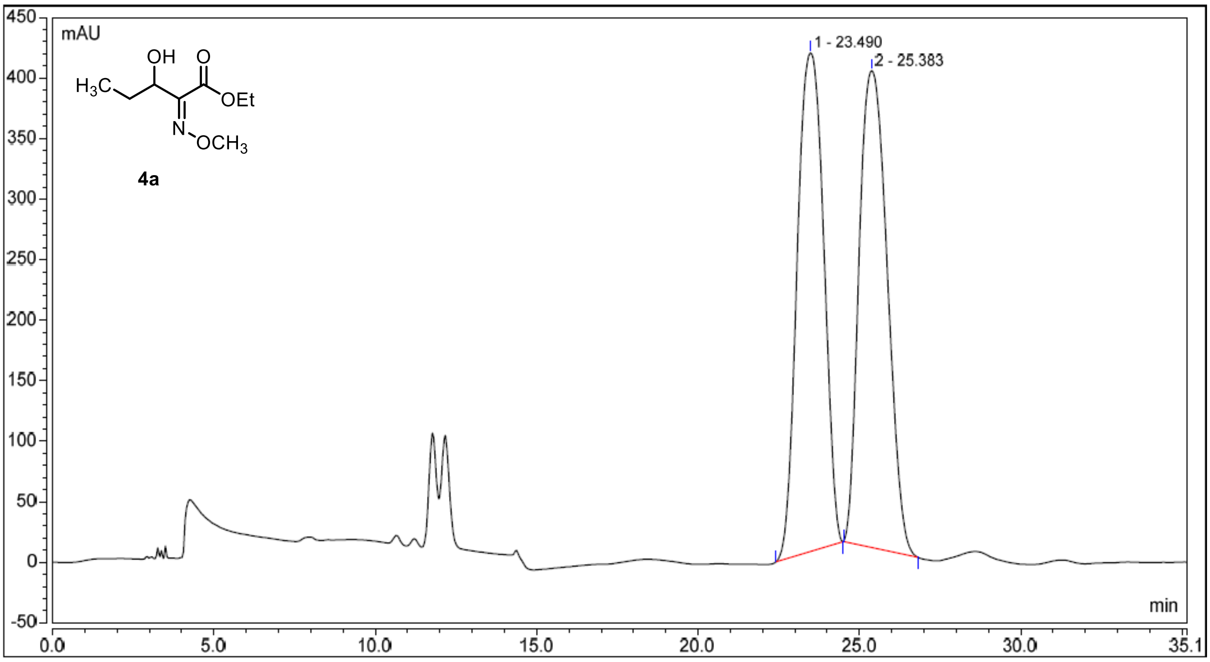

Peak	Retention Time (min)	Area (mAU × min)	Height (mAU)	Relative Area (%)
1	23.490	391.425	412.009	49.62
2	25.383	397.443	394.018	50.38
Total		788.868	806.027	100

Figure S6. HPLC chromatogram of enantiomerically enriched **4a**

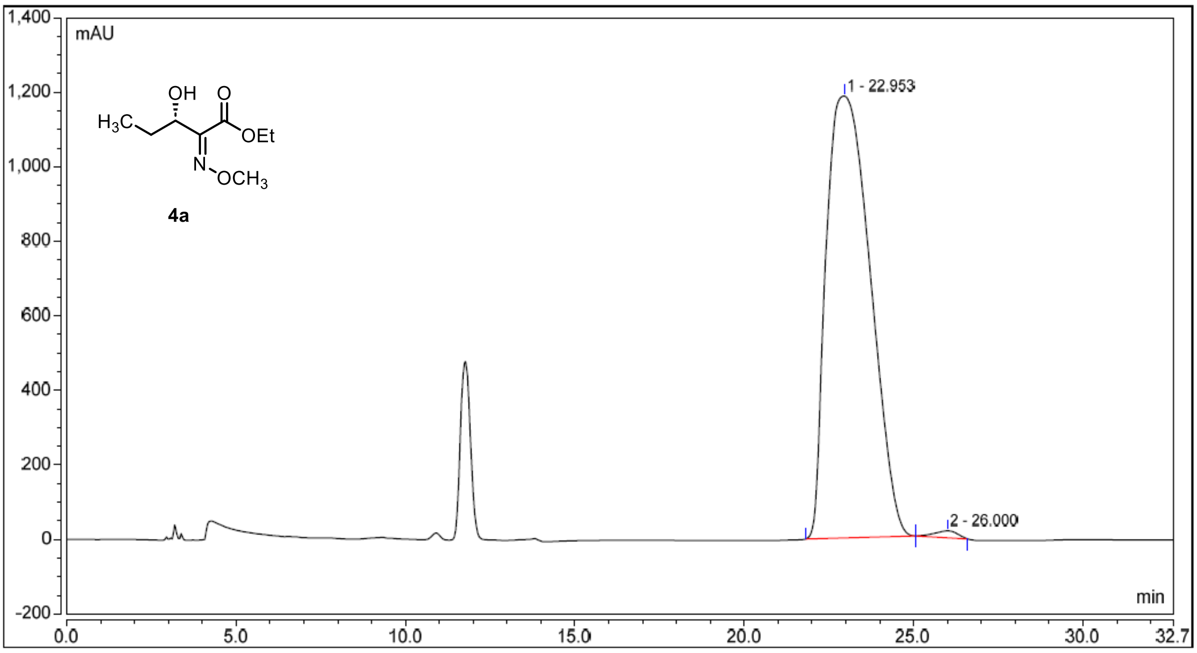

Peak	Retention Time (min)	Area (mAU × min)	Height (mAU)	Relative Area (%)
1	22.953	1785.515	1185.939	99.20
2	26.000	14.454	18.571	0.80
Total		1799.969	1204.51	100

Figure S7. HPLC chromatogram of **4a** from the transfer hydrogenation using 2-propanol instead of triethylamine–formic acid

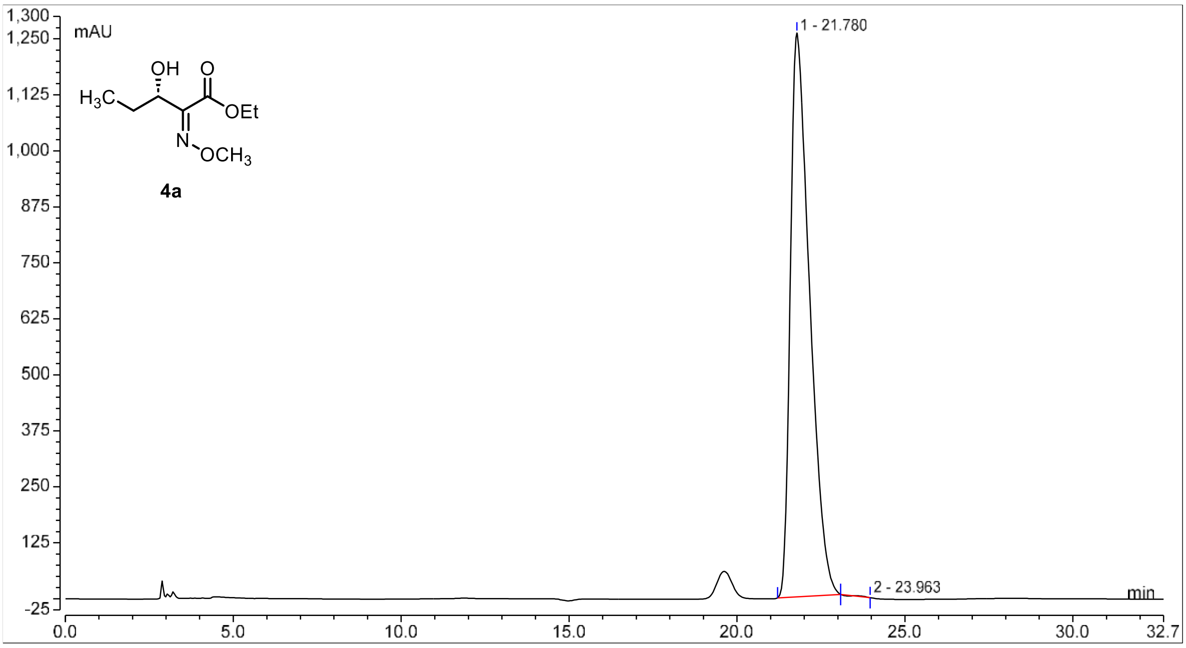

Peak	Retention Time (min)	Area (mAU × min)	Height (mAU)	Relative Area (%)
1	21.780	836.551	1258.563	99.98
2	23.963	0.204	0.00	0.02
Total		836.755	1258.563	100.00

HPLC analyses of racemic and enantiomerically enriched 4b

Chiralpak OJ column, 250 × 4.6 mm, λ = 254 nm, 1% 2-propanol in hexane, flow rate: 1.0 mL/min

Figure S8. HPLC chromatogram of (±)-**4b**

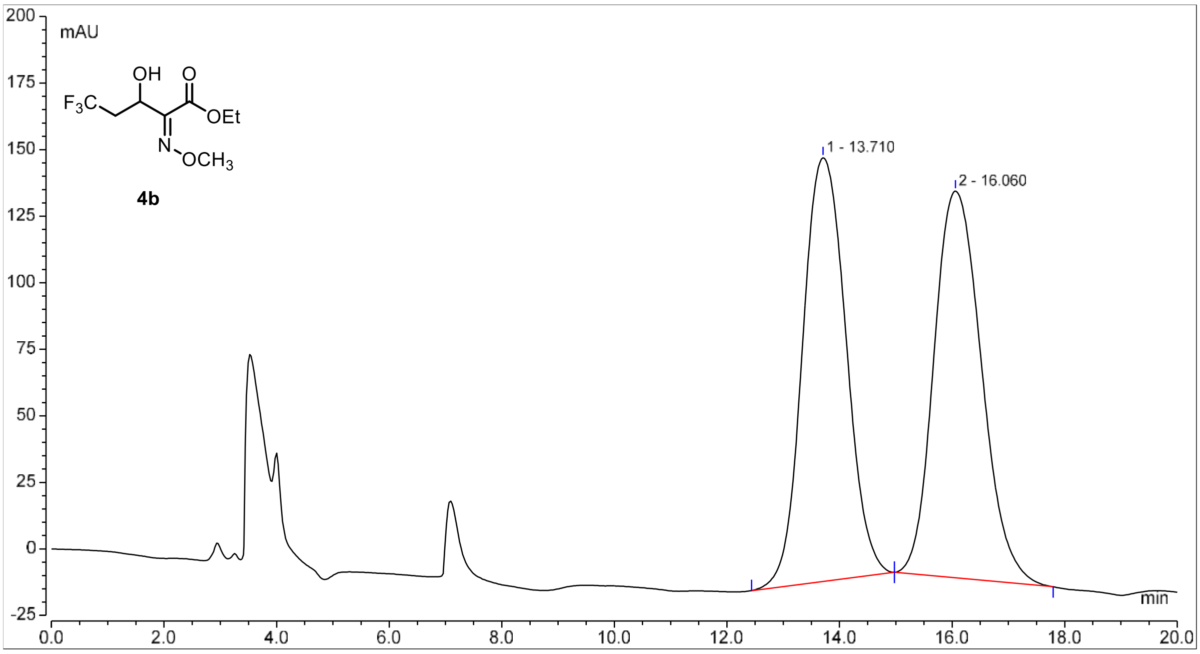

Peak	Retention Time (min)	Area (mAU × min)	Height (mAU)	Relative Area (%)
1	13.710	141.803	159.150	50.01
2	16.060	141.756	145.329	49.99
Total		283.559	304.479	100.00

Figure S9. HPLC chromatogram of enantiomerically enriched **4b**

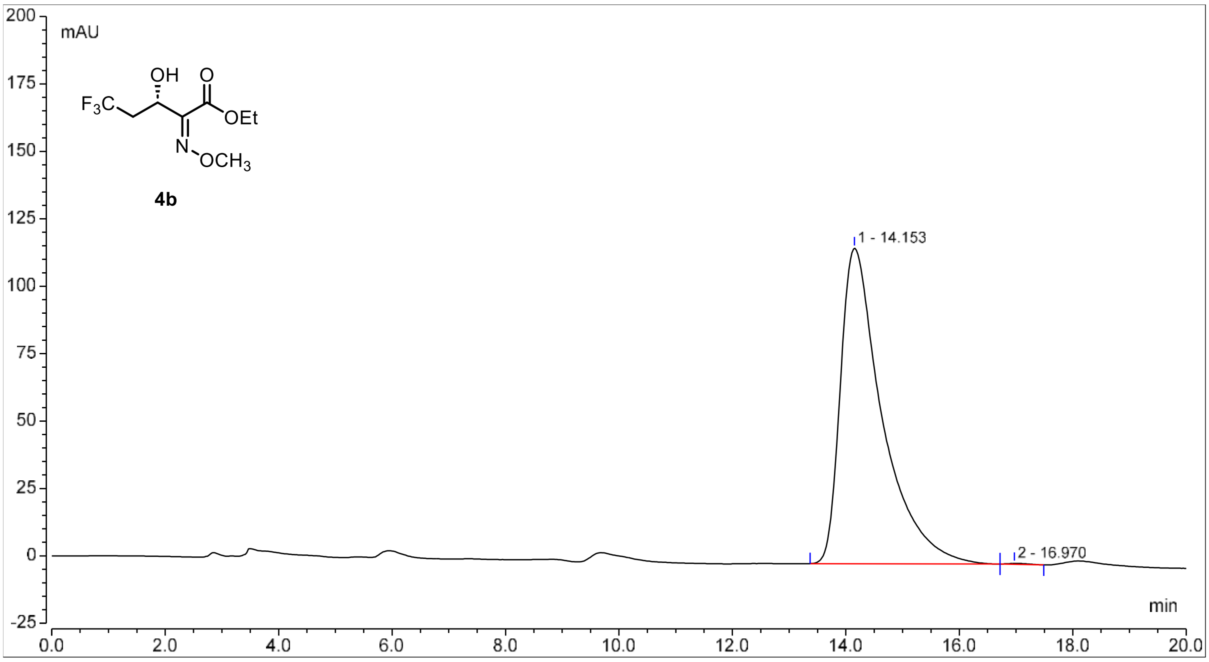

Peak	Retention Time (min)	Area (mAU × min)	Height (mAU)	Relative Area (%)
1	14.153	99.628	117.031	99.86
2	16.970	0.136	0.299	0.14
Total		99.764	117.330	100.00

HPLC analyses of racemic and enantiomerically enriched 4c

Chiralpak IA column, 250 × 4.6 mm, λ = 254 nm, 7.5% isopropyl alcohol in hexane, flow rate: 0.5 mL/min

Figure S10. HPLC chromatogram of (±)-**4c** (mixture of *Z* and *E* isomers)

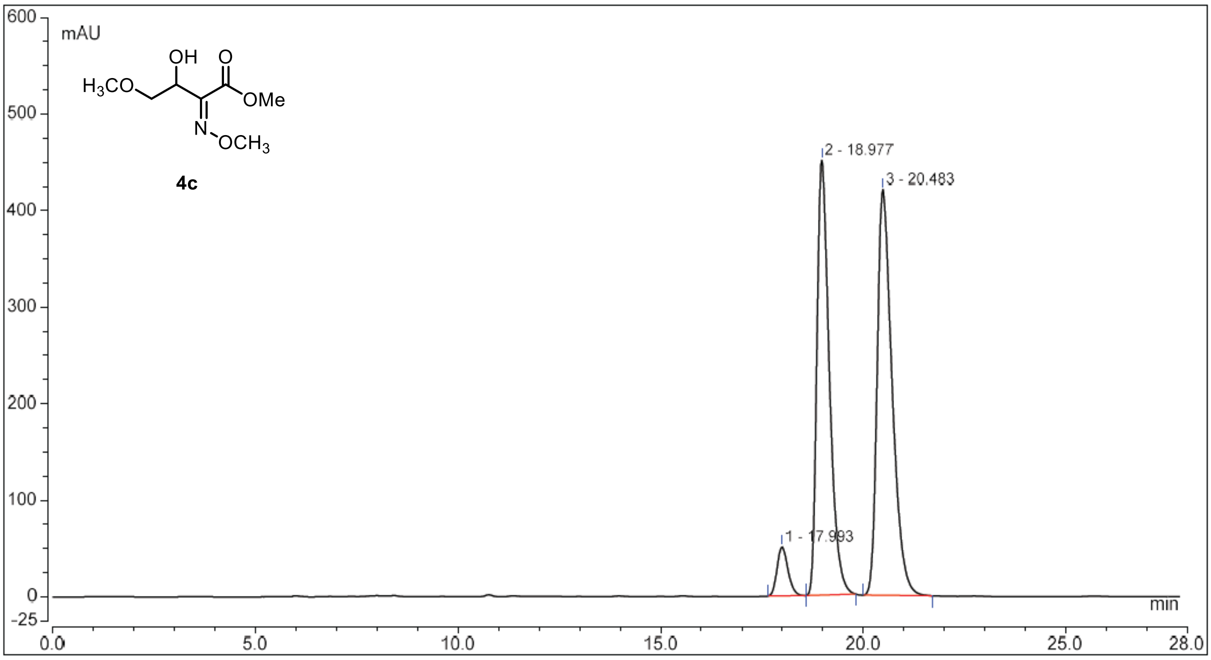

Peak	Retention Time (min)	Area (mAU × min)	Height (mAU)	Relative Area (%)
1	18.997	160.024	450.351	47.18
2	20.483	179.164	420.180	52.82
Total		339.18	870.531	100

Figure S11. HPLC chromatogram of enantiomerically enriched **4c**

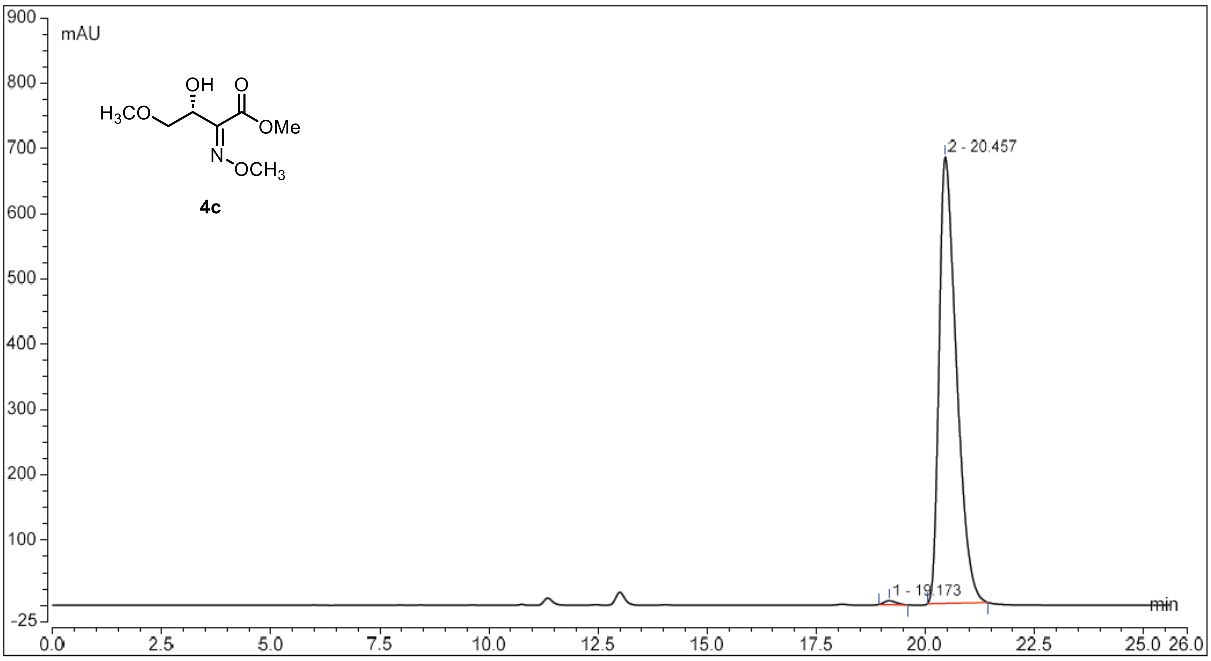

Peak	Retention Time (min)	Area (mAU × min)	Height (mAU)	Relative Area (%)
1	19.173	1.926	6.059	0.61
2	20.457	316.006	683.953	99.39
Total		317.932	690.012	100

HPLC analyses of racemic and enantiomerically enriched *Z*-4d

Chiralpak IH column, 250 × 4.6 mm, λ = 254 nm, 2% 2-propanol in hexane, flow rate: 1.0 mL/min

Figure S12. HPLC chromatogram of (±)-*Z*-**4d**

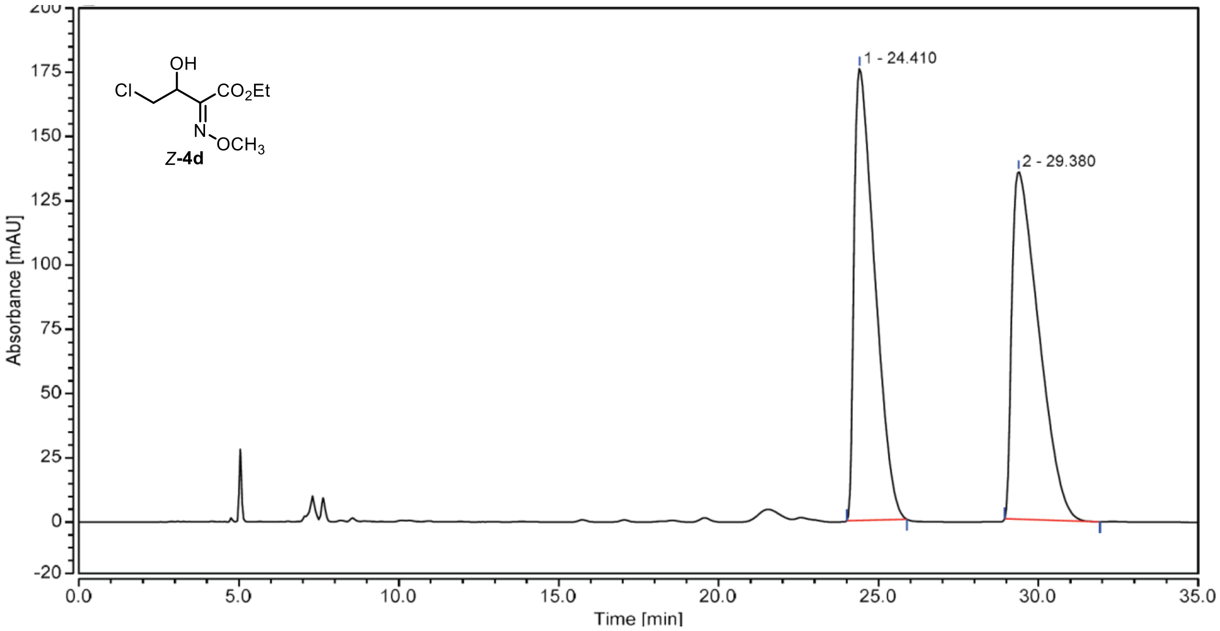

Peak	Retention Time (min)	Area (mAU × min)	Height (mAU)	Relative Area (%)
1	24.410	131.416	175.962	50.00
2	29.380	131.436	135.274	50.00
Total		262.851	311.236	100.00

Figure S13. HPLC chromatogram of enantiomerically enriched *Z*-**4d**

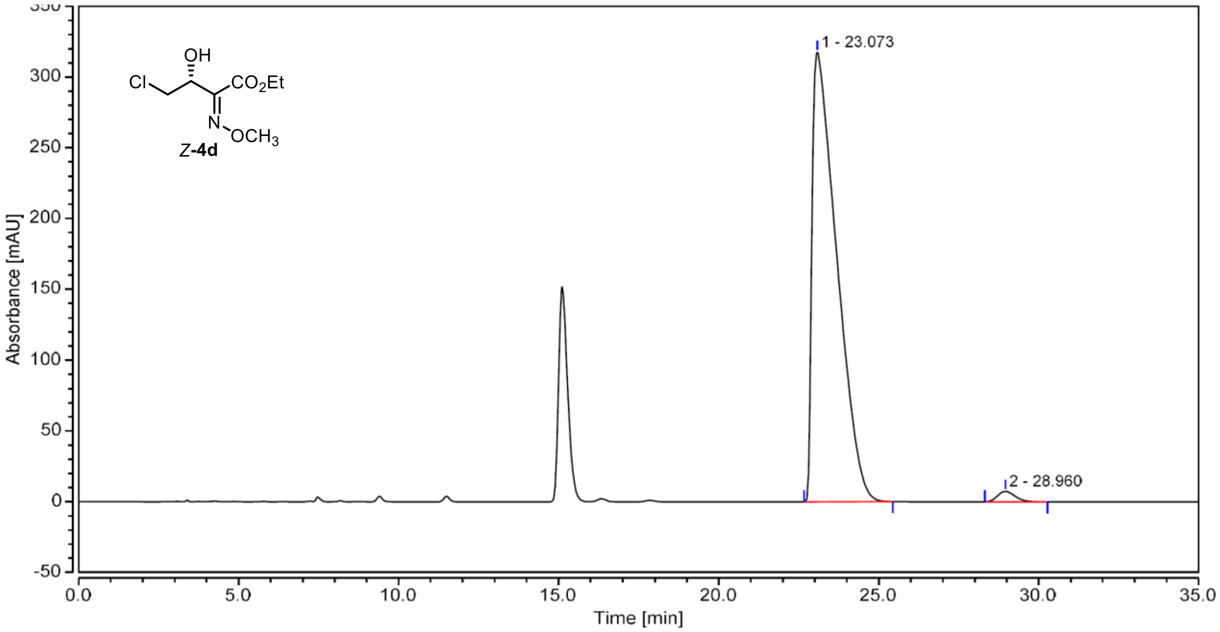

Peak	Retention Time (min)	Area (mAU × min)	Height (mAU)	Relative Area (%)
1	23.073	283.414	317.600	98.38
2	28.960	4.678	7.330	1.62
Total		288.092	324.929	100.00

HPLC analyses of racemic and enantiomerically enriched 4e

Chiralpak IA column, 250 × 4.6 mm, λ = 254 nm, 3% 2-propanol in hexane, flow rate: 1.0 mL/min

Figure S14. HPLC chromatogram of (±)-**4e**

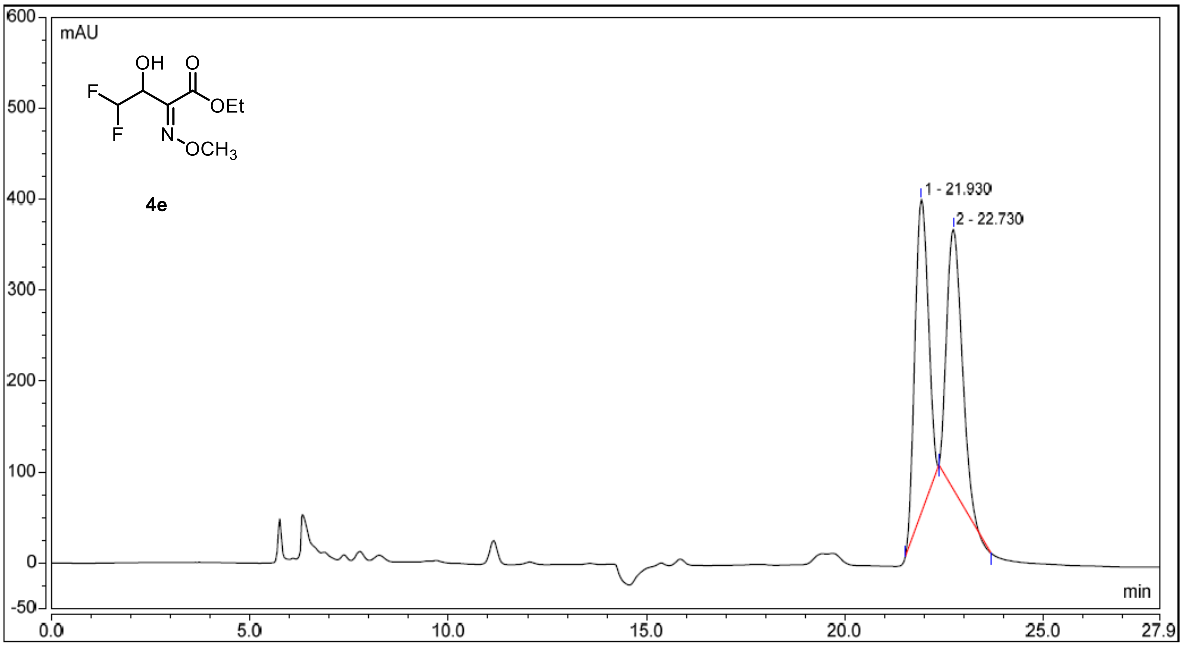

Peak	Retention Time (min)	Area (mAU × min)	Height (mAU)	Relative Area (%)
1	21.930	133.819	344.143	51.11
2	22.730	127.983	286.033	48.89
Total		261.802	630.176	100

Figure S15. HPLC chromatogram of enantiomerically enriched **4e**

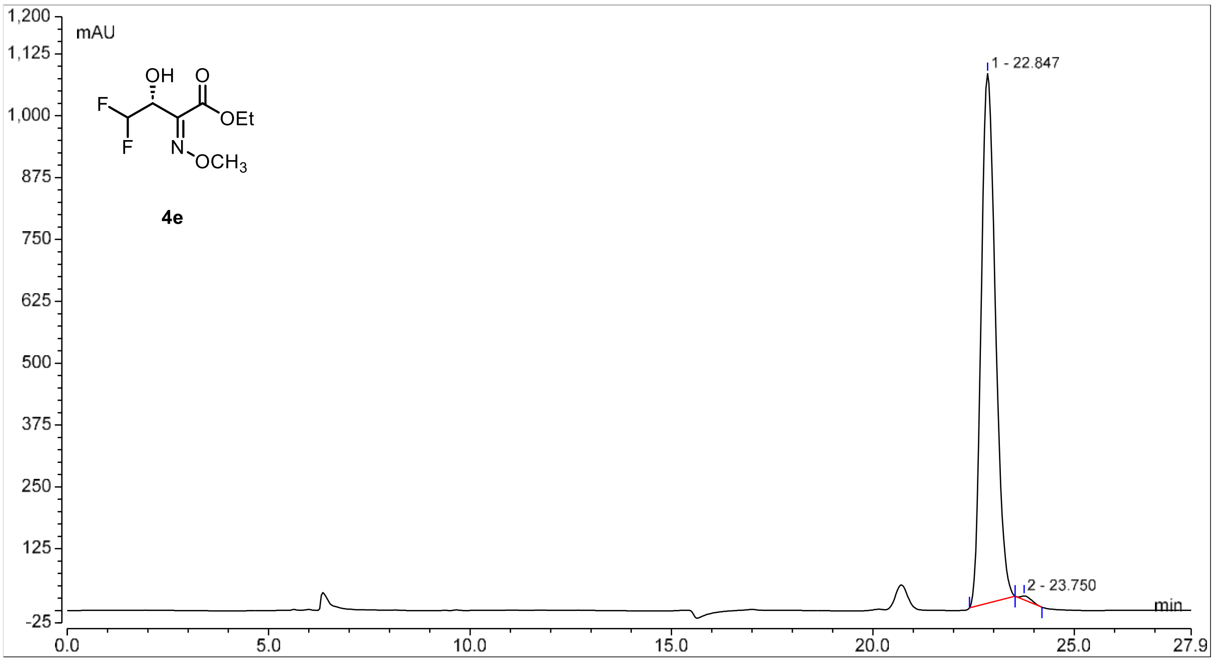

Peak	Retention Time (min)	Area (mAU × min)	Height (mAU)	Relative Area (%)
1	22.847	441.869	1070.588	99.42
2	23.750	2.592	7.747	0.58
Total		444.461	1078.335	100.00

HPLC analyses of racemic and enantiomerically enriched *Z*-4f

Chiralpak IA column, 250 × 4.6 mm, λ = 254 nm, 2% 2-propanol in hexane, flow rate: 1.0 mL/min

Figure S16. HPLC chromatogram of (±)-*Z*-**4f**

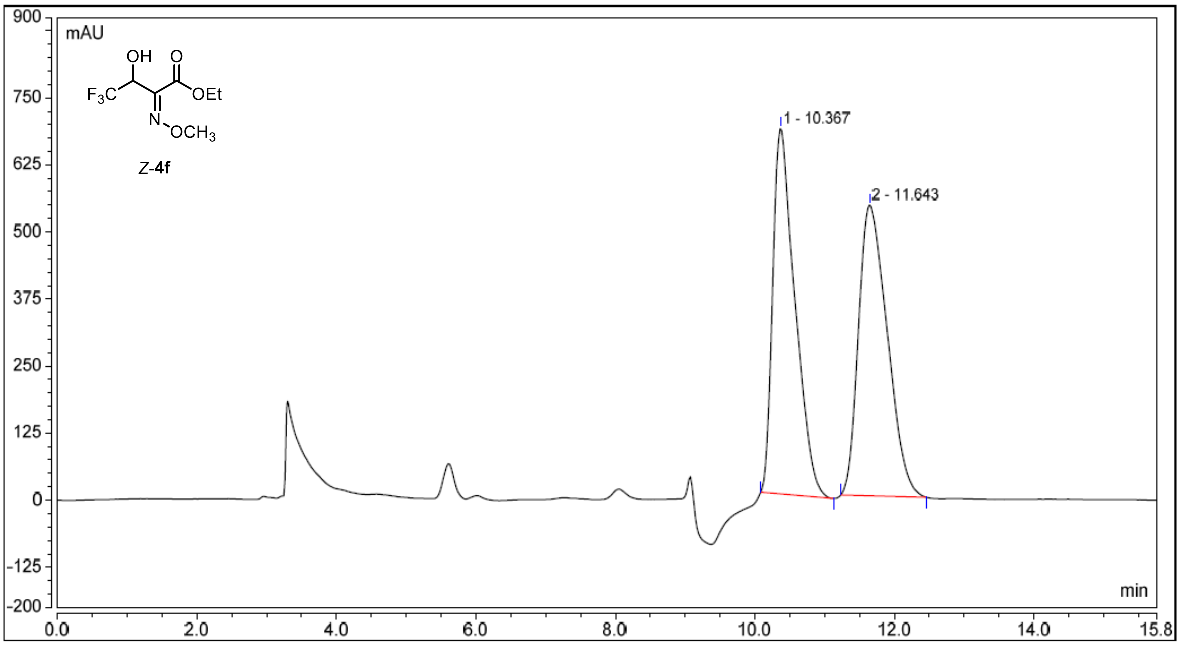

Peak	Retention Time (min)	Area (mAU × min)	Height (mAU)	Relative Area (%)
1	10.367	252.488	685.661	49.26
2	11.643	260.066	542.615	50.74
Total		512.554	1228.276	100

Chiralpak IA column, 250 × 4.6 mm, λ = 254 nm, 3% 2-propanol in hexane, flow rate: 1.0 mL/min

Figure S17. HPLC chromatogram of enantiomerically enriched *Z*-**4f**

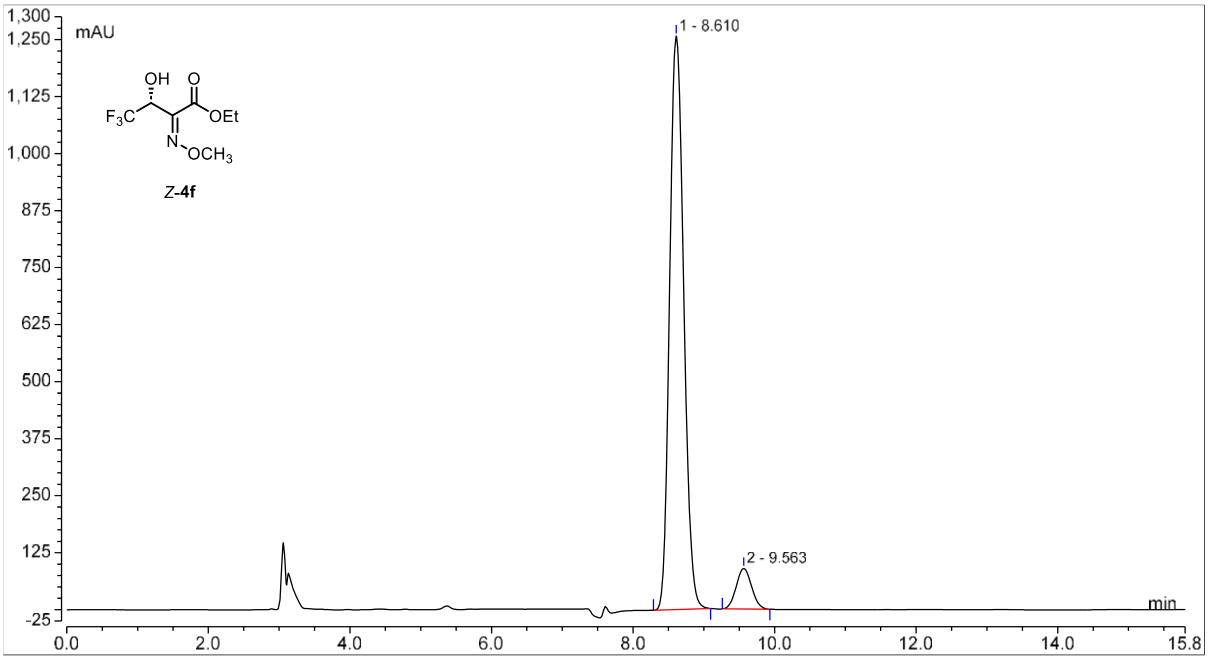

Peak	Retention Time (min)	Area (mAU × min)	Height (mAU)	Relative Area (%)
1	8.610	290.286	1257.286	92.81
2	9.563	22.476	89.200	7.19
Total		312.762	1346.486	100.00

HPLC analyses of racemic and enantiomerically enriched *Z*-4g

Chiralpak IA column, 250 × 4.6 mm, λ = 254 nm, 1% 2-propanol in hexane, flow rate: 1.0 mL/min

Figure S18. HPLC chromatogram of (±) *Z*-**4g**

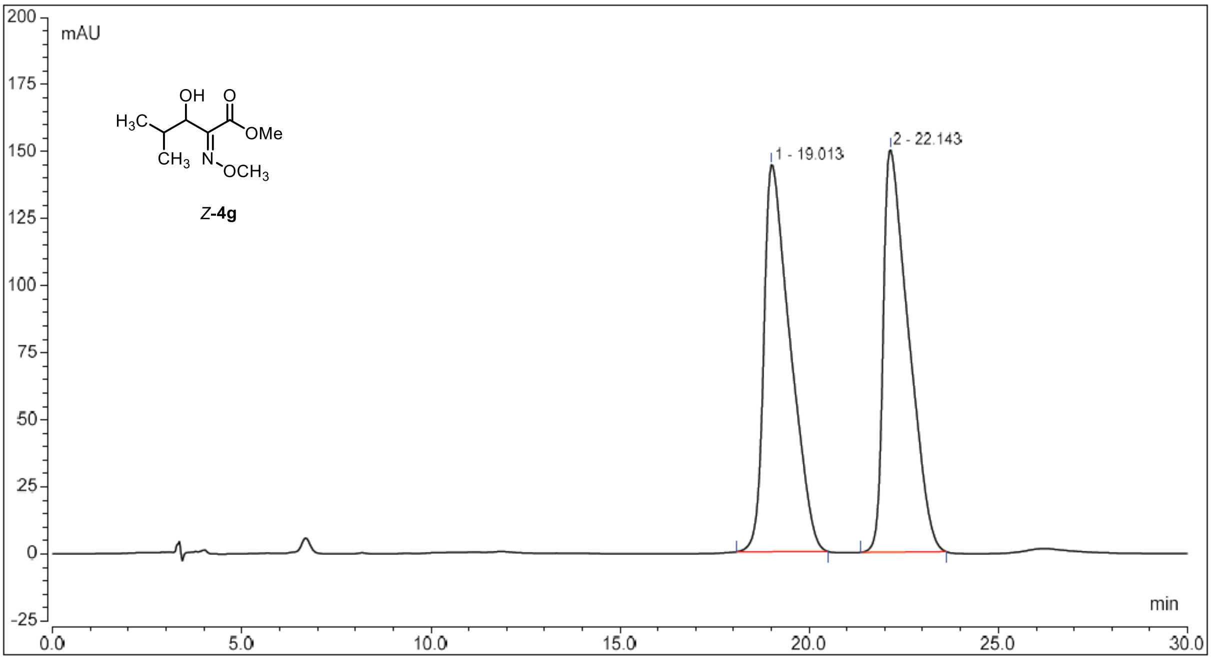

Peak	Retention Time (min)	Area (mAU × min)	Height (mAU)	Relative Area (%)
1	19.013	116.223	144.481	50.06
2	22.143	115.965	150.204	49.94
Total		232.188	294.685	100

Figure S19. HPLC chromatogram of enantiomerically enriched *Z*-**4g**

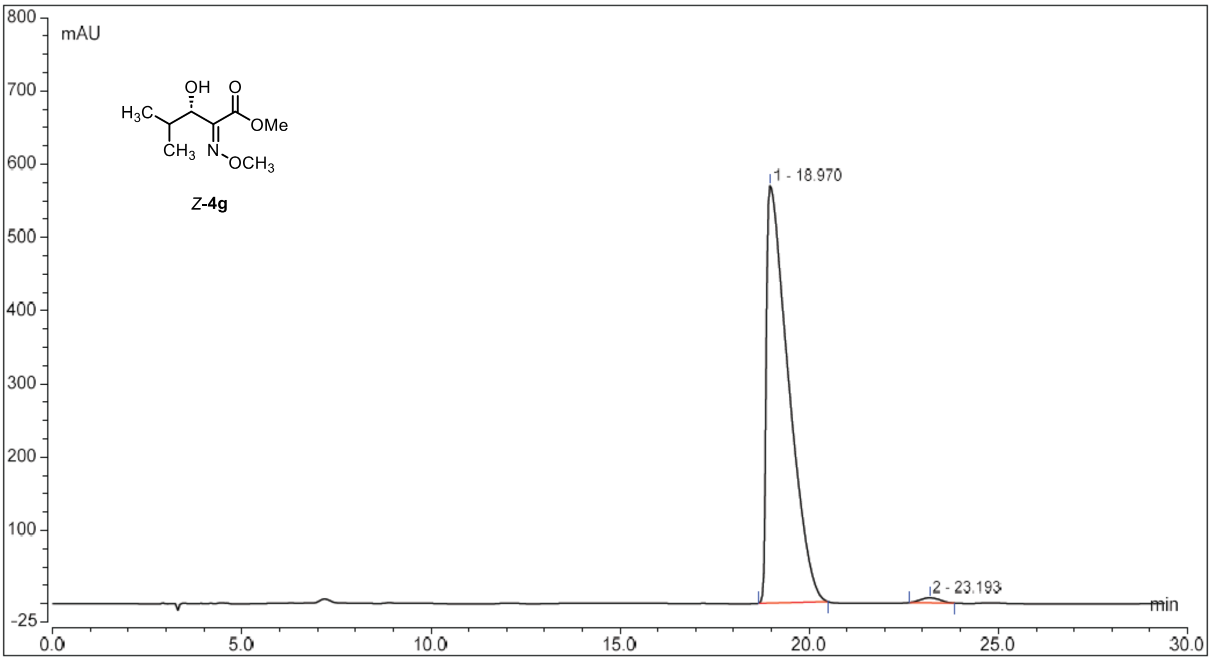

Peak	Retention Time (min)	Area (mAU × min)	Height (mAU)	Relative Area (%)
1	18.970	384.410	570.444	98.93
2	23.193	4.142	6.924	1.07
Total		388.552	577.368	100

HPLC analyses of racemic and enantiomerically enriched *E*-4g

Chiralpak IA column, 250 × 4.6 mm, λ = 254 nm, 1% isopropyl alcohol in hexane, flow rate: 1.0 mL/min

Figure S19. HPLC chromatogram of (±)-*E*-**4g**

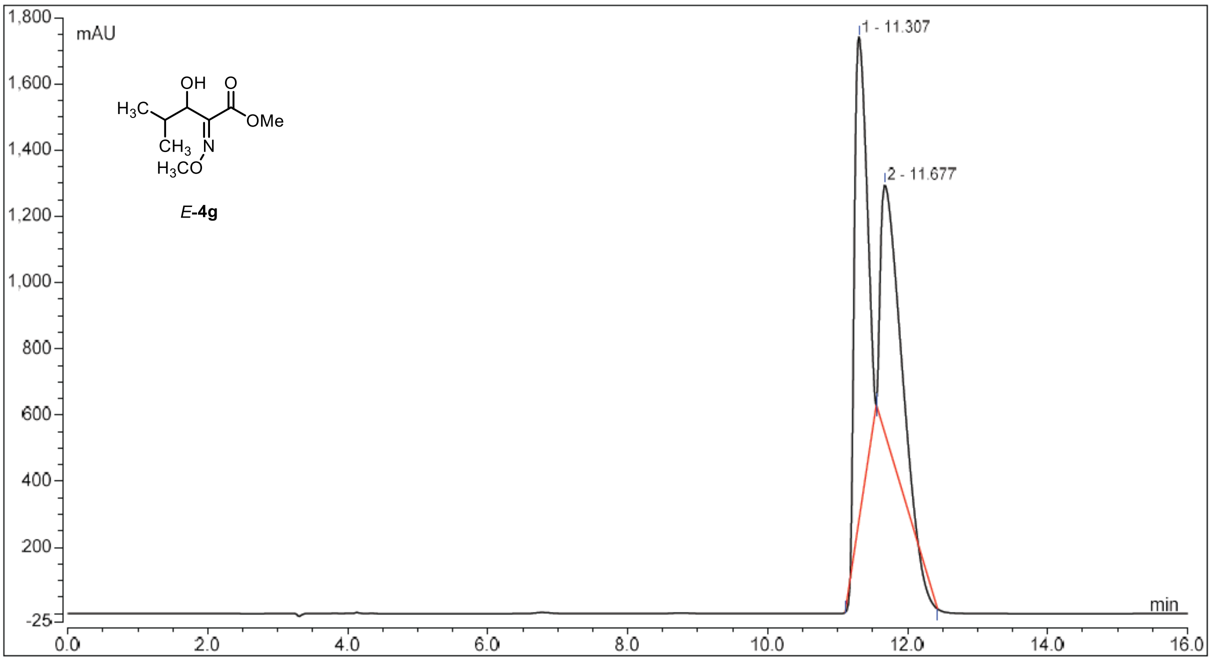

Peak	Retention Time (min)	Area (mAU × min)	Height (mAU)	Relative Area (%)
1	11.307	259.546	1457.506	50.12
2	11.677	239.668	751.352	49.88
Total		499.214	2208.858	100

Figure S20. HPLC chromatogram of enantiomerically enriched *E*-**4g**

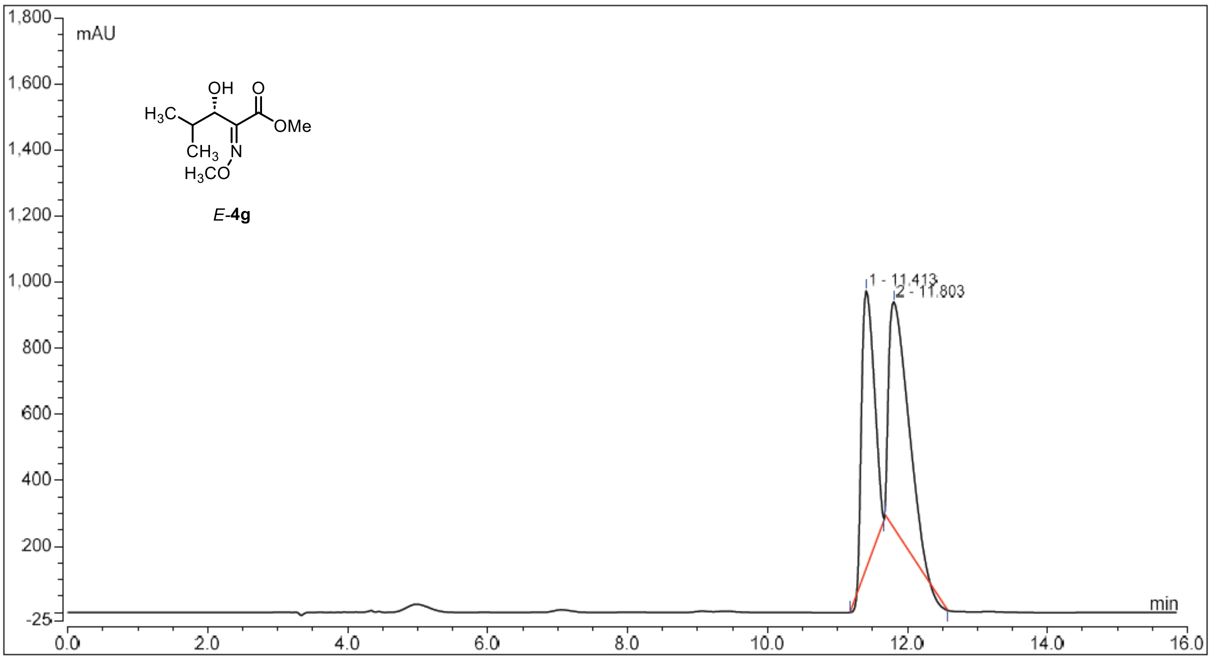

Peak	Retention Time (min)	Area (mAU × min)	Height (mAU)	Relative Area (%)
1	11.413	169.891	838.578	43.47
2	11.803	220.925	684.267	56.53
Total		390.817	1522.845	100

HPLC analyses of racemic and enantiomerically enriched *Z*-4h

Chiralpak IA column, 250 × 4.6 mm, λ = 254 nm, 1% ethanol in hexane, flow rate: 1.0 mL/min

Figure S21. HPLC chromatogram of (±)-*Z*-**4h**

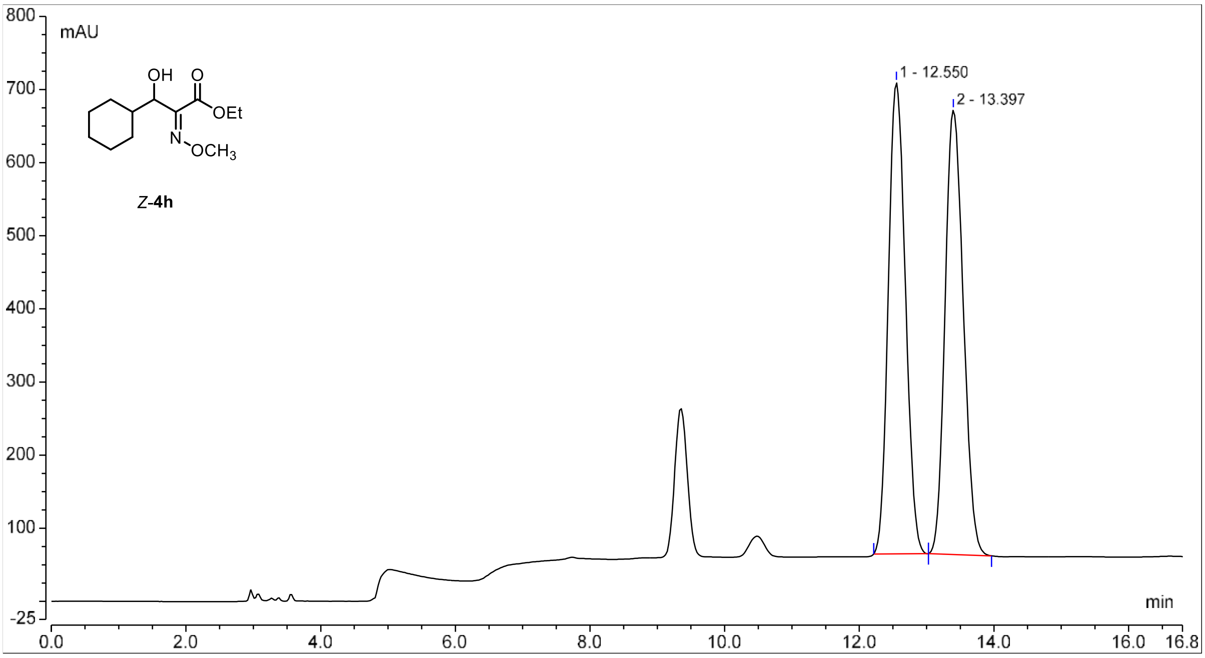

Peak	Retention Time (min)	Area (mAU × min)	Height (mAU)	Relative Area (%)
1	12.550	189.362	644.536	49.79
2	13.397	190.971	607.793	50.21
Total		380.333	1252.329	100.00

Figure S22. HPLC chromatogram of enantiomerically enriched *Z*-**4h**

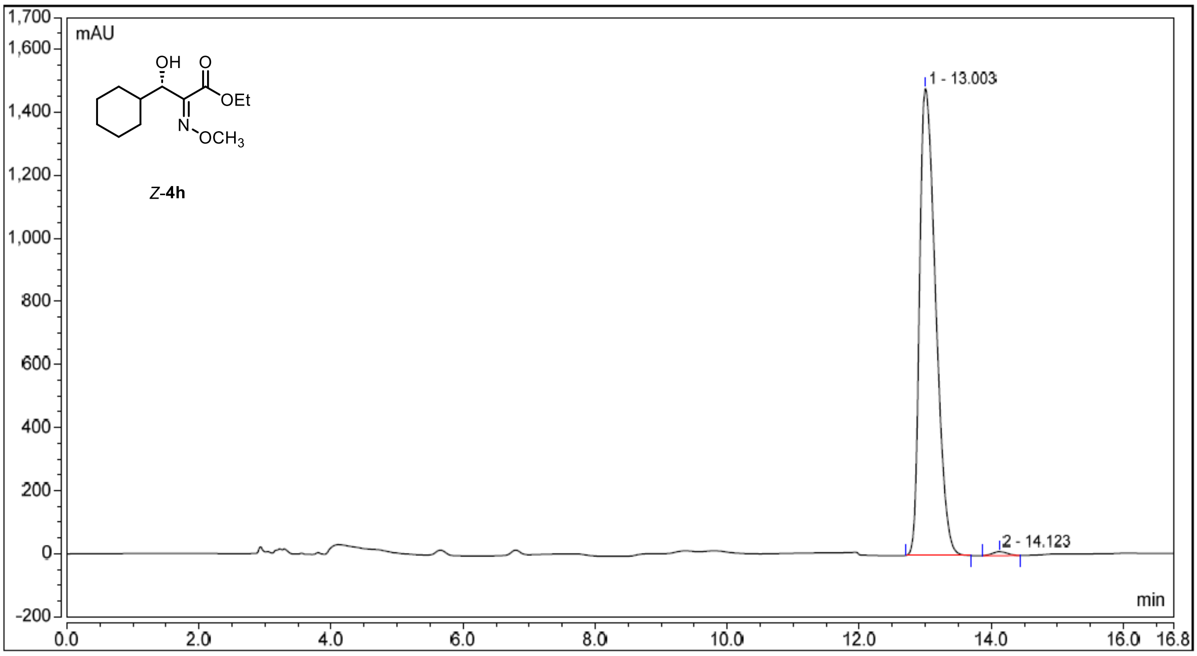

Peak	Retention Time (min)	Area (mAU × min)	Height (mAU)	Relative Area (%)
1	13.003	418.217	1480.217	99.24
2	14.123	3.193	12.439	0.83
Total		421.409	1492.741	100.00

HPLC analyses of racemic and enantiomerically enriched *Z*-4i

Chiralpak IA column, 250 × 4.6 mm, λ = 254 nm, 1% isopropyl alcohol in hexane, flow rate: 1.0 mL/min

Figure S23. HPLC chromatogram of (±)-*Z*-**4i**

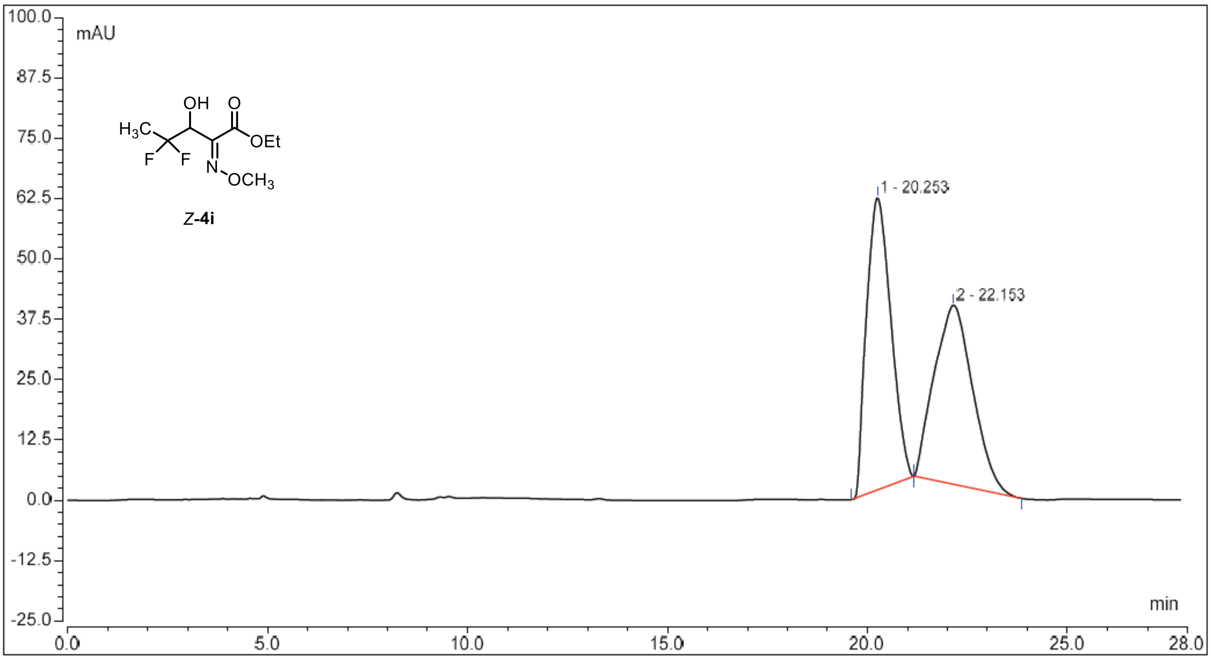

Peak	Retention Time (min)	Area (mAU × min)	Height (mAU)	Relative Area (%)
1	20.253	42.300	60.499	50.23
2	22.153	41.686	37.128	49.77
Total		83.986	97.627	100

Figure S24. HPLC chromatogram of enantiomerically enriched *Z*-**4i**

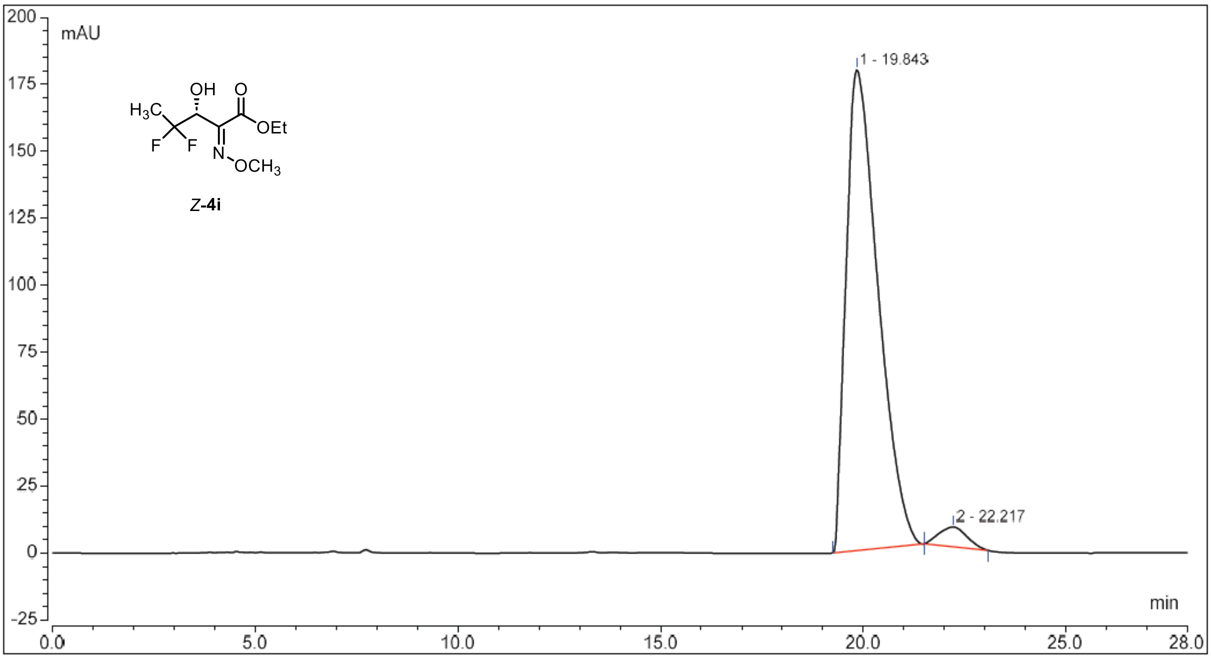

Peak	Retention Time (min)	Area (mAU × min)	Height (mAU)	Relative Area (%)
1	19.843	164.277	179.561	96.53
2	22.217	5.914	7.369	3.47
Total		170.191	186.930	100

HPLC analyses of racemic and enantiomerically enriched *E*-4i

Chiralpak IA column, 250 × 4.6 mm, λ = 254 nm, 1% ethyl alcohol in hexane, flow rate: 1.0 mL/min

Figure S25. HPLC chromatogram of (±)-*E*-**4i**

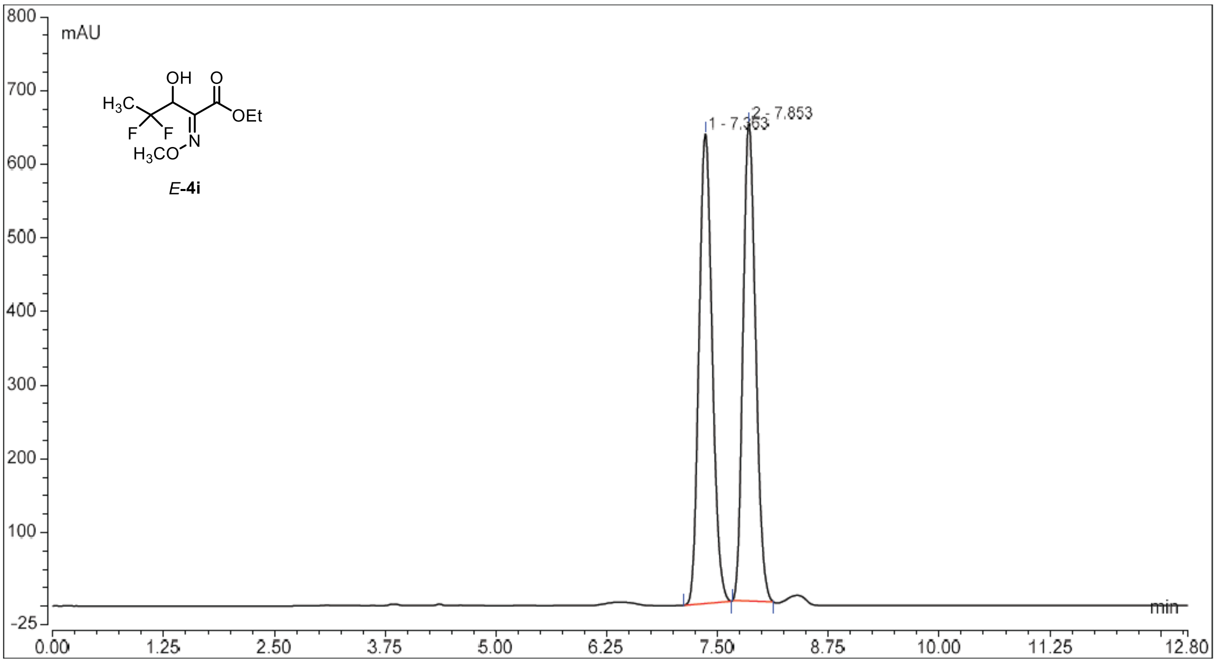

Peak	Retention Time (min)	Area (mAU × min)	Height (mAU)	Relative Area (%)
1	7.363	110.210	637.656	50.64
2	7.853	107.411	648.319	49.36
Total		217.621	1285.975	100

Figure S26. HPLC chromatogram of enantiomerically enriched *E*-**4i**

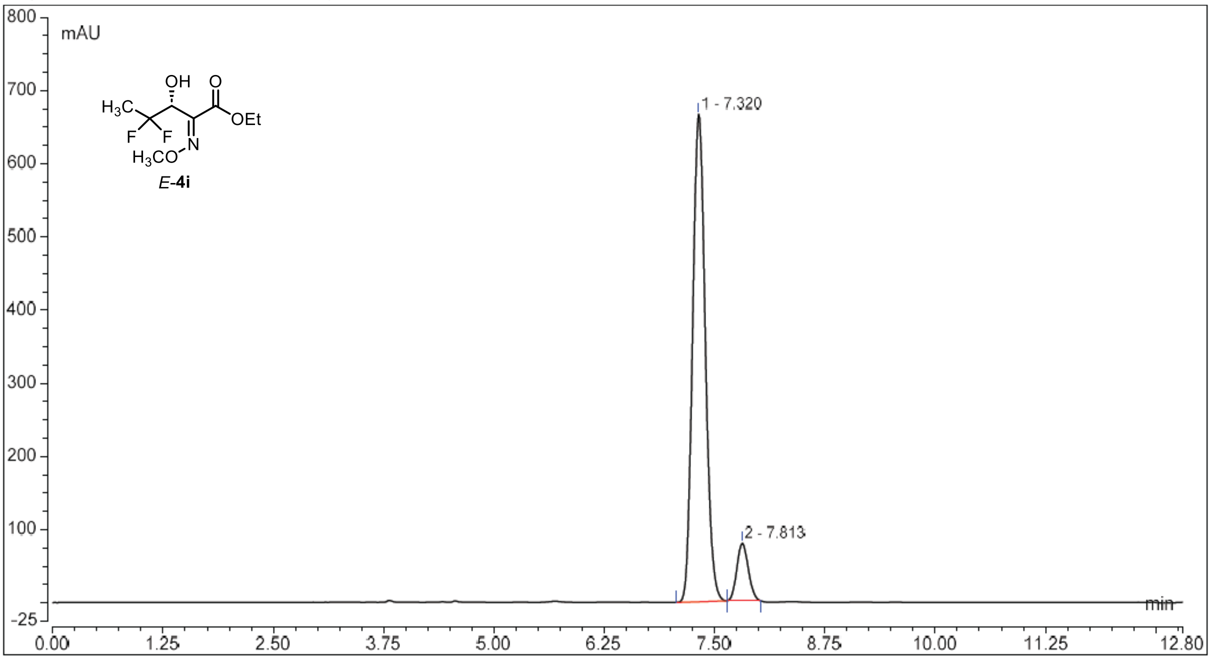

Peak	Retention Time (min)	Area (mAU × min)	Height (mAU)	Relative Area (%)
1	7.320	111.225	666.472	90.34
2	7.813	11.891	78.793	9.66
Total		123.116	745.265	100

HPLC analyses of racemic and enantiomerically enriched *Z*-4j

Chiralpak IA column, 250 × 4.6 mm, λ = 254 nm, 1% 2-propanol in hexane, flow rate: 1.0 mL/min

Figure S27. HPLC chromatogram of (±)-**4j** (mixture of *Z* and *E* isomers, optimized for *Z*-isomer)

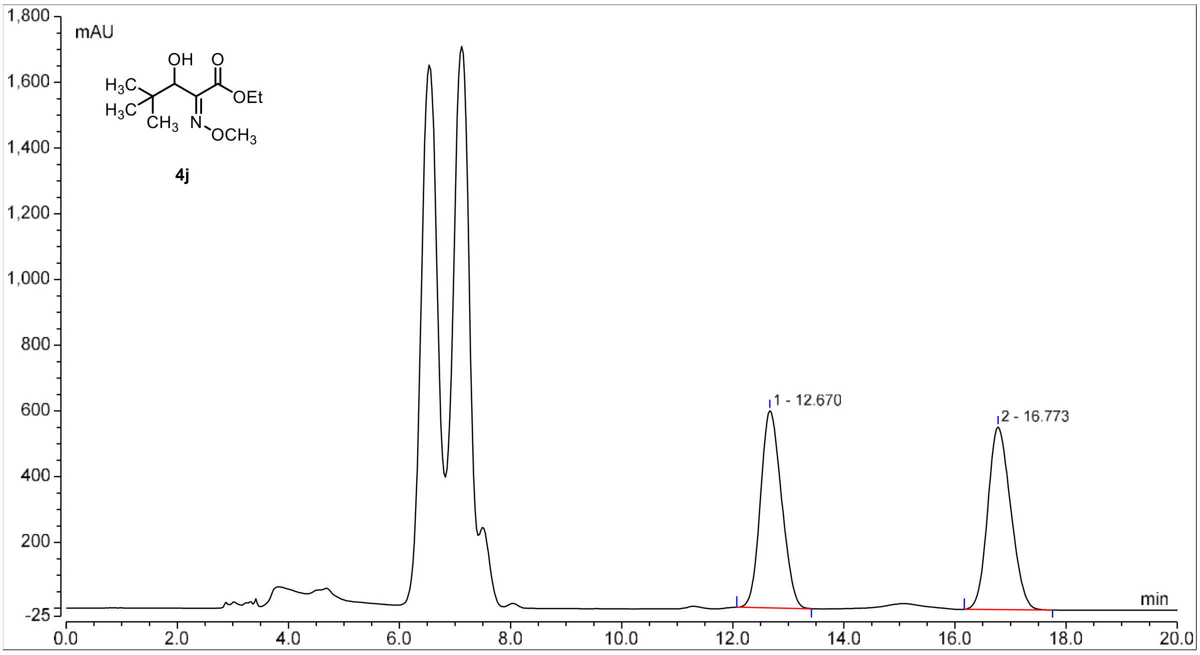

Peak	Retention Time (min)	Area (mAU × min)	Height (mAU)	Relative Area (%)
1	12.670	263.841	599.930	49.76
2	16.773	266.427	555.599	50.24
Total		530.268	1155.529	100.00

Figure S28. HPLC chromatogram of enantiomerically enriched *Z*-**4j**

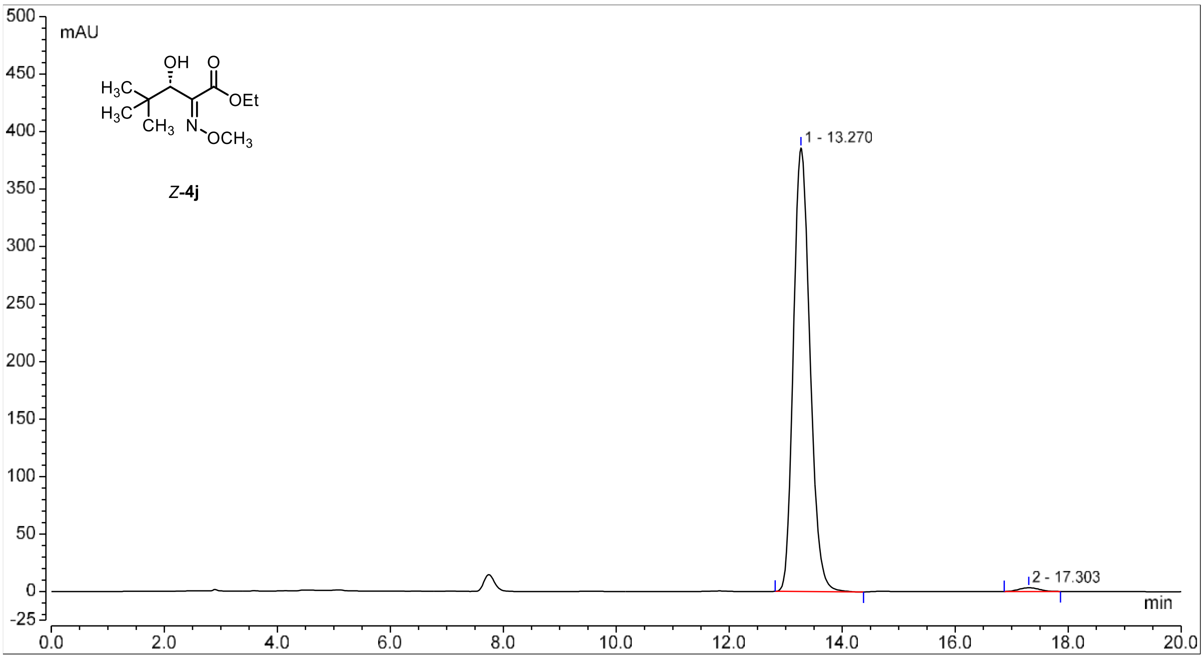

Peak	Retention Time (min)	Area (mAU × min)	Height (mAU)	Relative Area (%)
1	13.270	131.215	385.710	98.98
2	17.303	1.352	3.218	1.02
Total		132.567	388.928	100.00

HPLC analyses of racemic and enantiomerically enriched *Z*-4k

Chiralpak AD column, 250 × 4.6 mm, λ = 254 nm, 3% isopropyl alcohol in hexane, flow rate: 1.0 mL/min

Figure S29. HPLC chromatogram of (±)-**4k** (mixture of *E*-*Z* isomers, optimized for the *Z*-isomer)

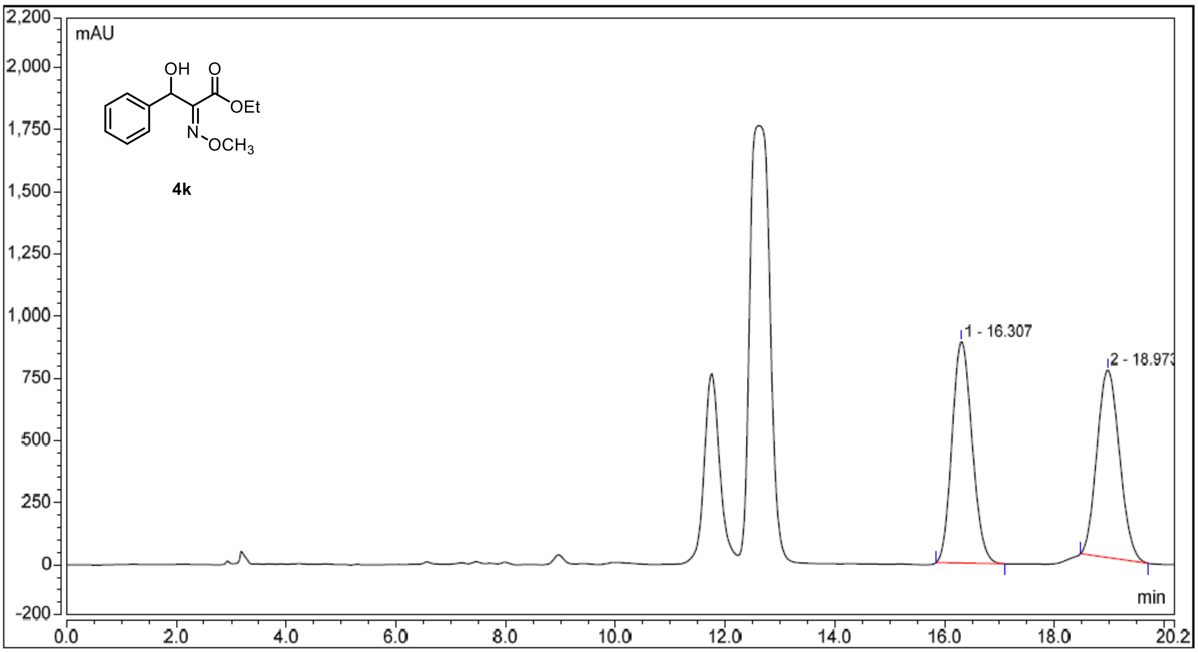

Peak	Retention Time (min)	Area (mAU × min)	Height (mAU)	Relative Area (%)
1	16.307	377.490	890.976	51.22
2	18.973	359.557	755.205	48.78
Total		737.047	1646.181	100

Figure S30. HPLC chromatogram of enantiomerically enriched *Z*-**4k** (using (*S*,*S*)-**1**)

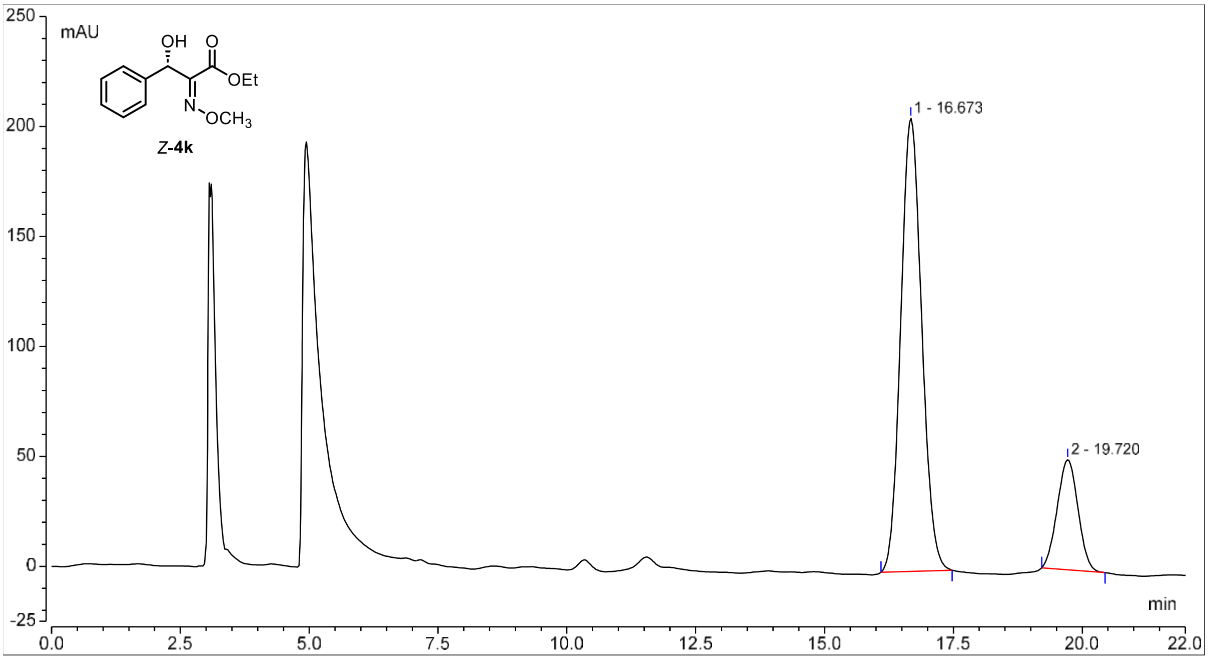

Peak	Retention Time (min)	Area (mAU × min)	Height (mAU)	Relative Area (%)
1	16.673	97.119	206.017	80.64
2	19.720	23.320	50.045	19.36
Total		120.439	256.062	100.00

Figure S31. HPLC chromatogram of enantiomerically enriched *Z*-**4k** (using (*S*,*S*)-**2**)

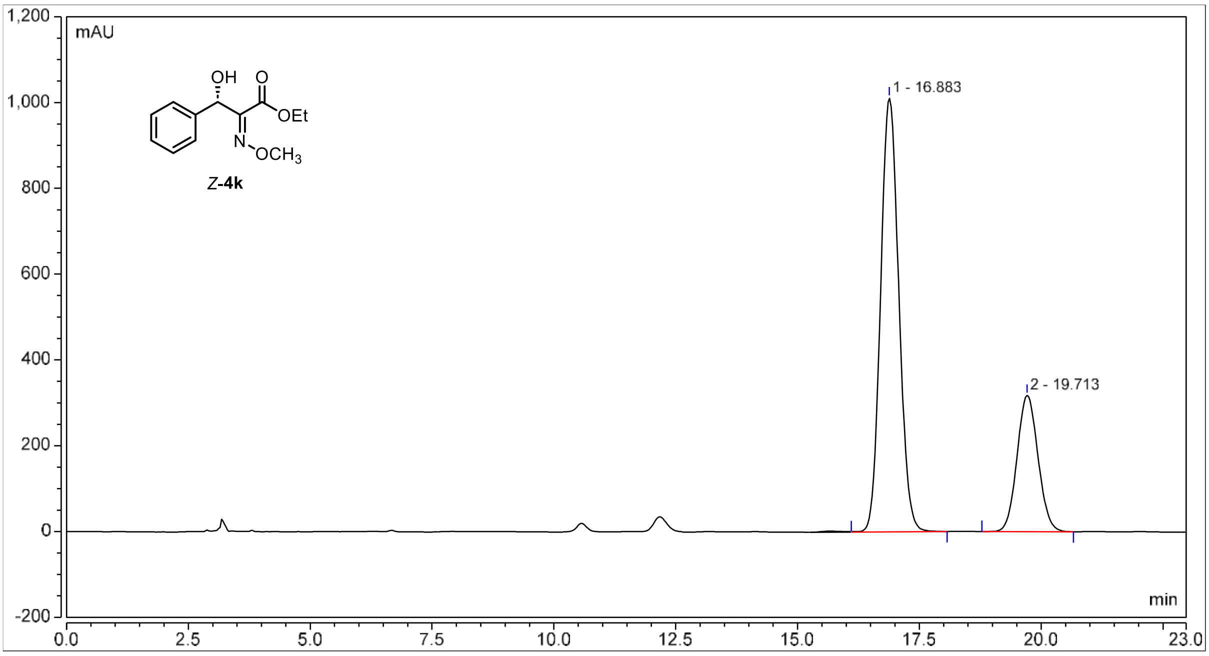

Peak	Retention Time (min)	Area (mAU × min)	Height (mAU)	Relative Area (%)
1	16.883	437.526	1011.014	73.40
2	19.713	158.563	317.808	26.60
Total		596.089	1328.822	100.00

Figure S32. HPLC chromatogram of enantiomerically enriched *Z*-**4k** (using *p*-methoxy diamine ligand)

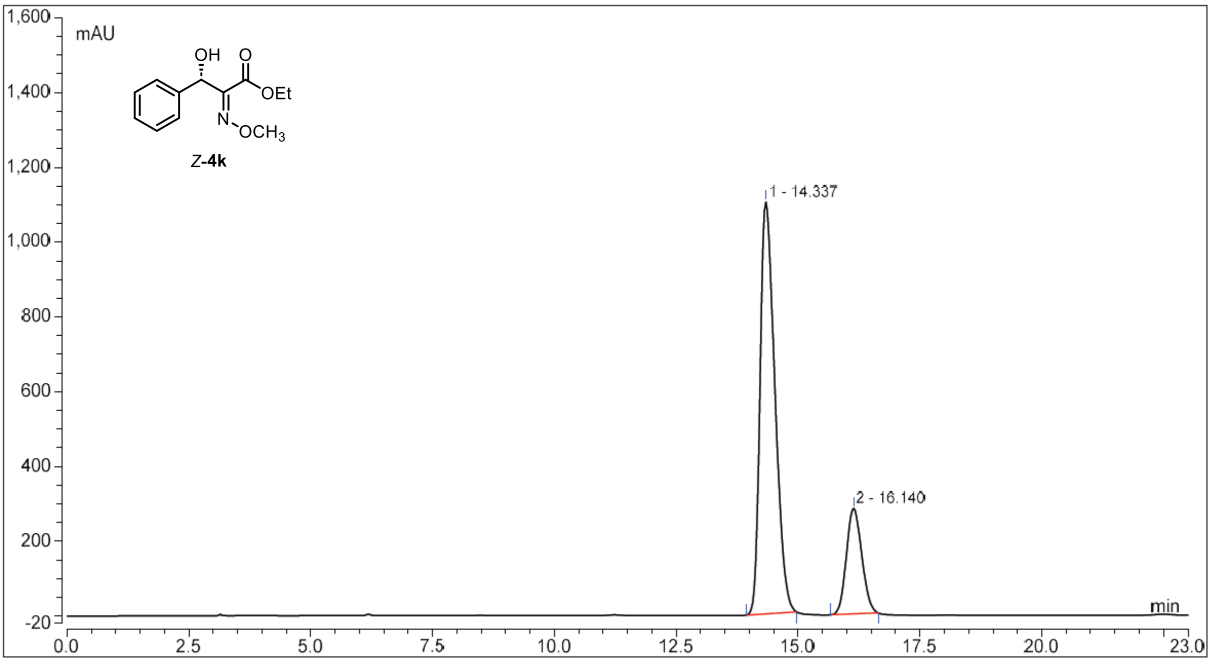

Peak	Retention Time (min)	Area (mAU × min)	Height (mAU)	Relative Area (%)
1	14.337	388.201	1101.611	79.03
2	16.140	102.988	282.589	20.97
Total		491.189	1384.200	100

Figure S33. HPLC chromatogram of enantiomerically enriched *Z*-**4k** (using *p*-trifluoromethyl diamine ligand)

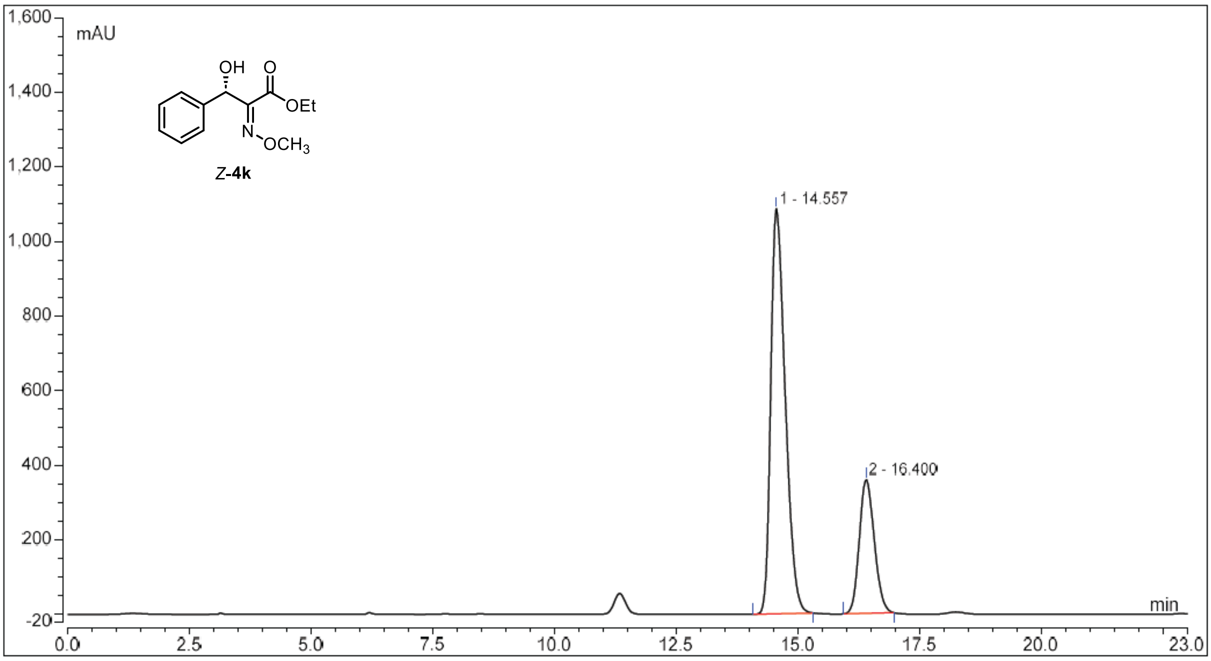

Peak	Retention Time (min)	Area (mAU × min)	Height (mAU)	Relative Area (%)
1	14.557	373.028	1086.382	74.45
2	16.400	128.031	359.016	25.55
Total		501.059	1445.398	100

Figure S34. HPLC chromatogram of enantiomerically enriched *Z*-**4k** (using *p*-nitro diamine ligand)

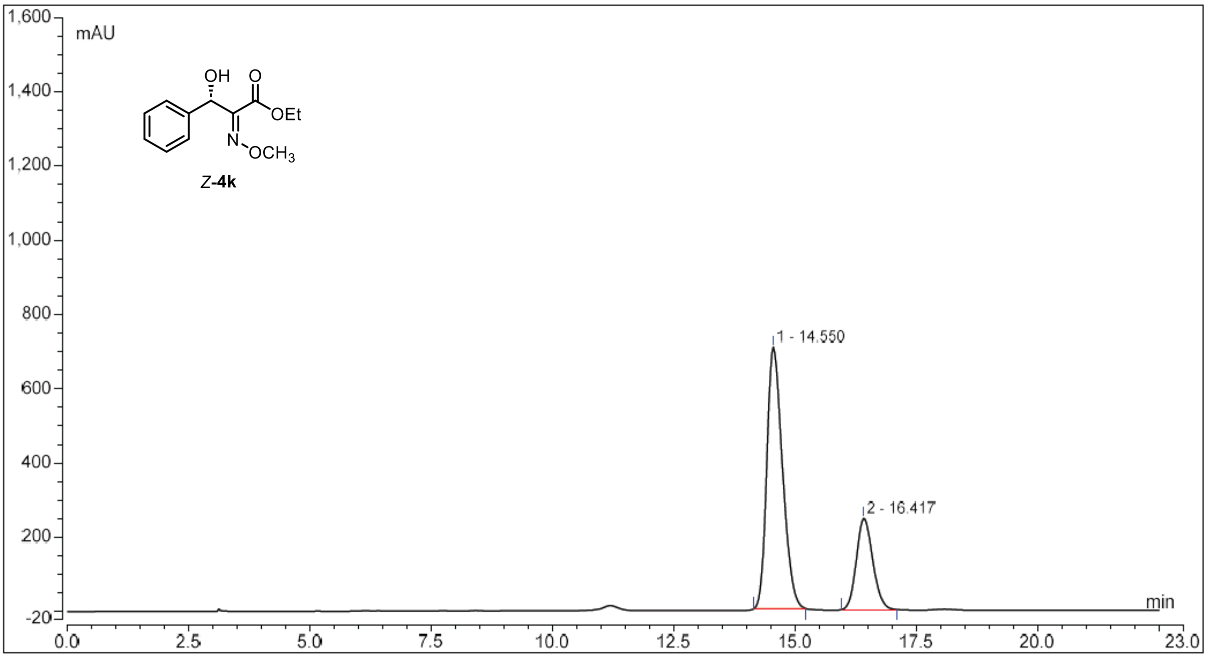

Peak	Retention Time (min)	Area (mAU × min)	Height (mAU)	Relative Area (%)
1	14.550	263.574	704.861	73.31
2	16.417	95.959	245.866	26.69
Total		359.533	950.727	100

HPLC analyses of racemic and enantiomerically enriched *E*-4k

Chiralpak IA column, 250 × 4.6 mm, λ = 254 nm, 1% isopropyl alcohol in hexane, flow rate: 1.0 mL/min

Figure S35. HPLC chromatogram of (±)-*E*-**4k**

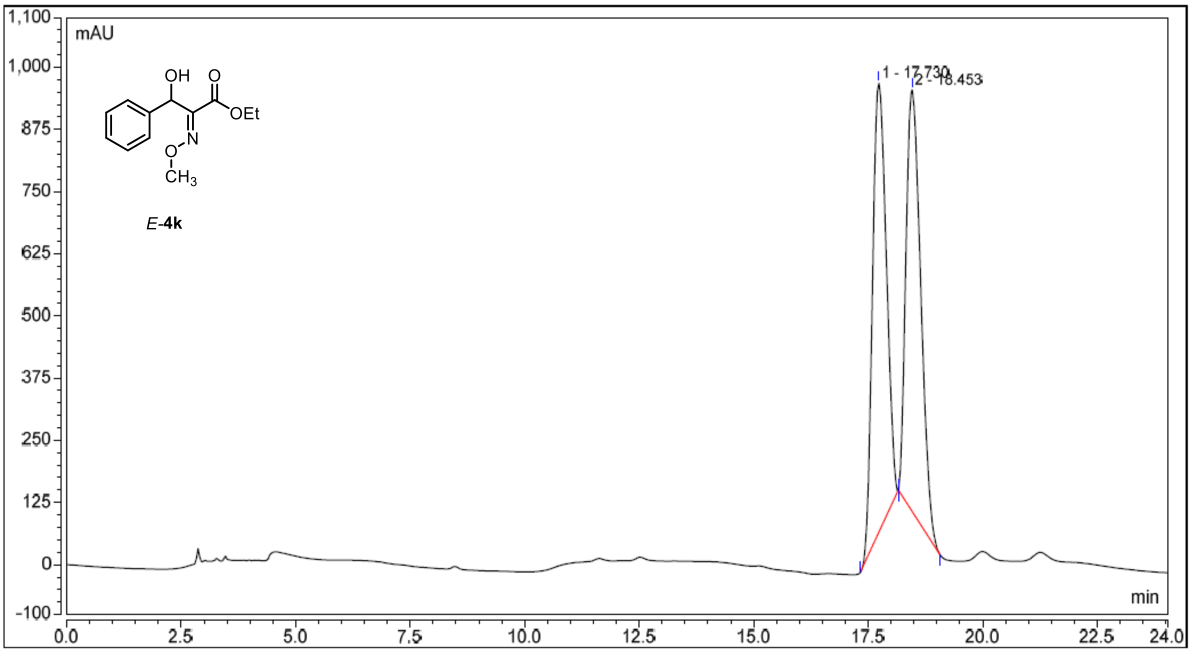

Peak	Retention Time (min)	Area (mAU × min)	Height (mAU)	Relative Area (%)
1	17.730	314.818	903.859	49.87
2	18.453	316.408	847.179	50.13
Total		631.226	1751.038	100.00

Figure S36. HPLC chromatogram of enantiomerically enriched *E*-**4k** (using *S*,*S*-**2**)

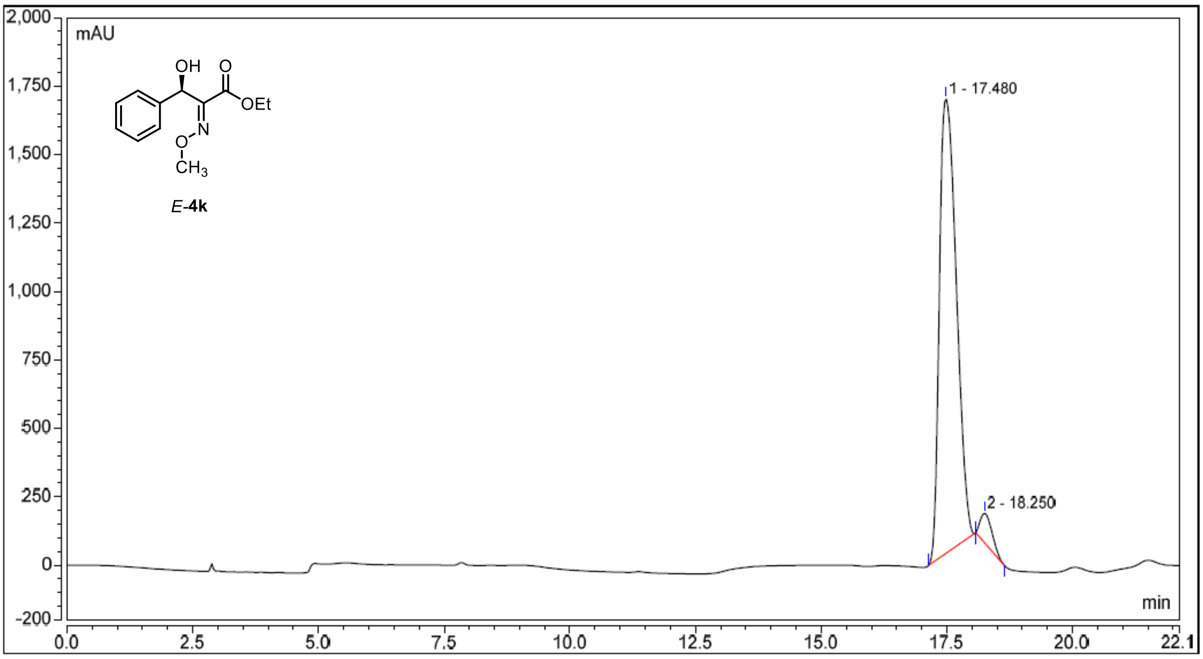

Peak	Retention Time (min)	Area (mAU × min)	Height (mAU)	Relative Area (%)
1	17.480	648.141	1661.006	93.91
2	18.250	30.183	107.688	6.09
Total		678.324	1768.694	100.00

HPLC analyses of racemic and enantiomerically enriched *Z*-4l

Chiralpak IA column, 250 × 4.6 mm, λ = 254 nm, 4% isopropyl alcohol in hexane, flow rate: 1.0 mL/min

Figure S37. HPLC chromatogram of (±)-*Z*-**4l**

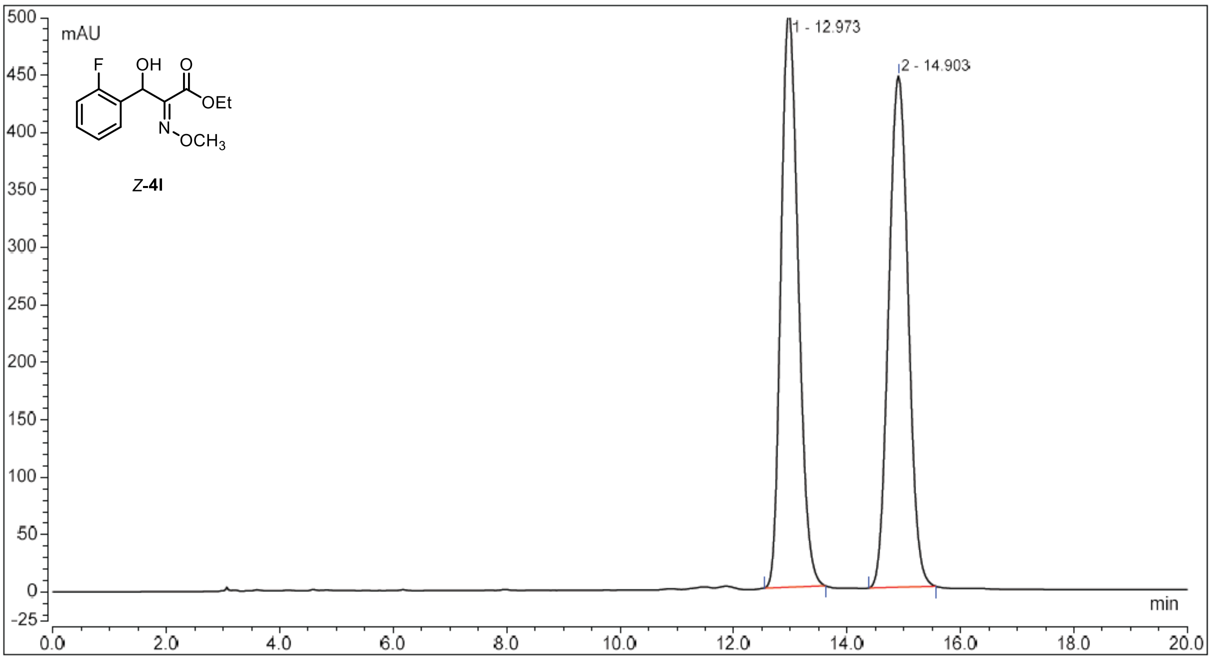

Peak	Retention Time (min)	Area (mAU × min)	Height (mAU)	Relative Area (%)
1	12.973	177.075	501.447	49.94
2	14.903	177.478	445.293	50.06
Total		354.553	946.740	100

Figure S38. HPLC chromatogram of enantiomerically enriched *Z*-**4l**

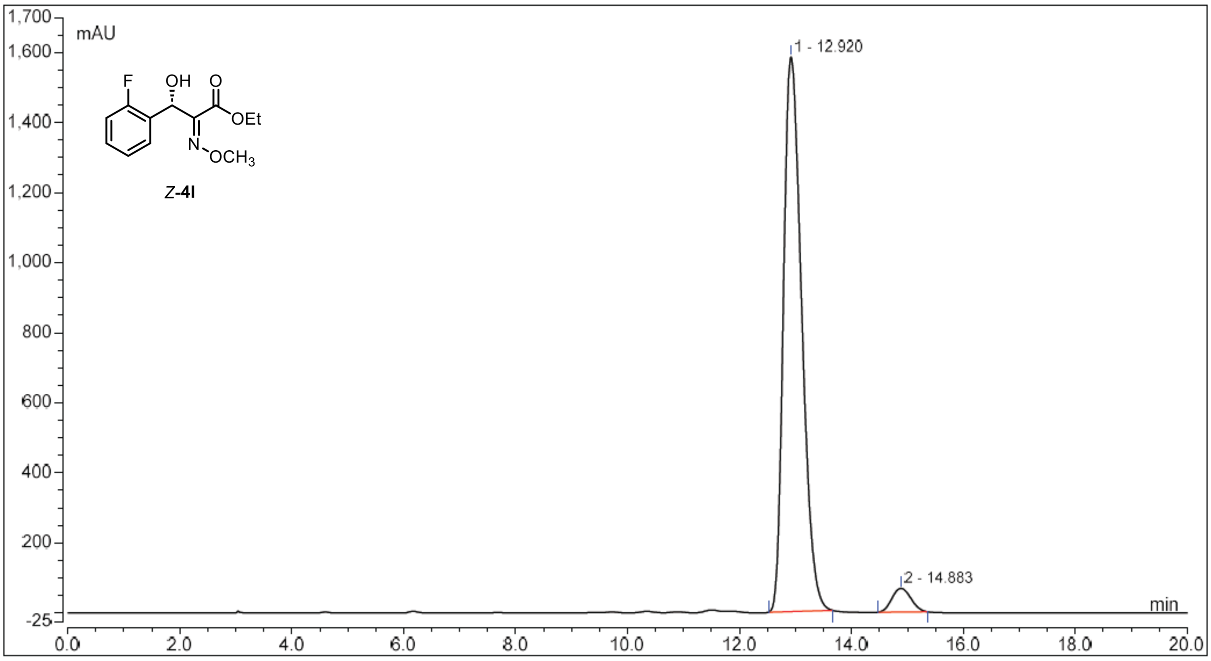

Peak	Retention Time (min)	Area (mAU × min)	Height (mAU)	Relative Area (%)
1	12.920	594.874	1583.807	95.73
2	14.883	26.503	67.691	4.27
Total		621.377	1651.498	100

HPLC analyses of racemic and enantiomerically enriched *Z*-4m

Chiralpak IA column, 250 × 4.6 mm, λ = 254 nm, 4% isopropyl alcohol in hexane, flow rate: 1.0 mL/min

Figure S39. HPLC chromatogram of (±)-*Z*-**4m**

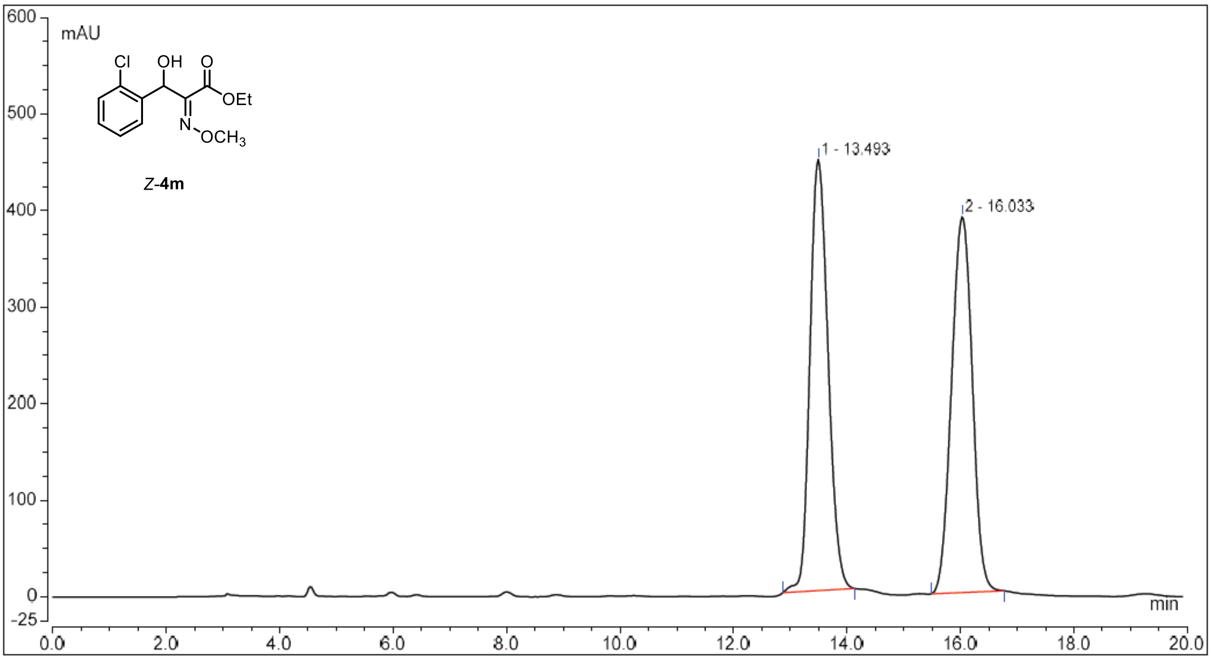

Peak	Retention Time (min)	Area (mAU × min)	Height (mAU)	Relative Area (%)
1	13.493	165.198	446.777	50.09
2	16.033	164.611	389.436	49.91
Total		329.809	836.213	100

Figure S40. HPLC chromatogram of enantiomerically enriched *Z*-**4m**

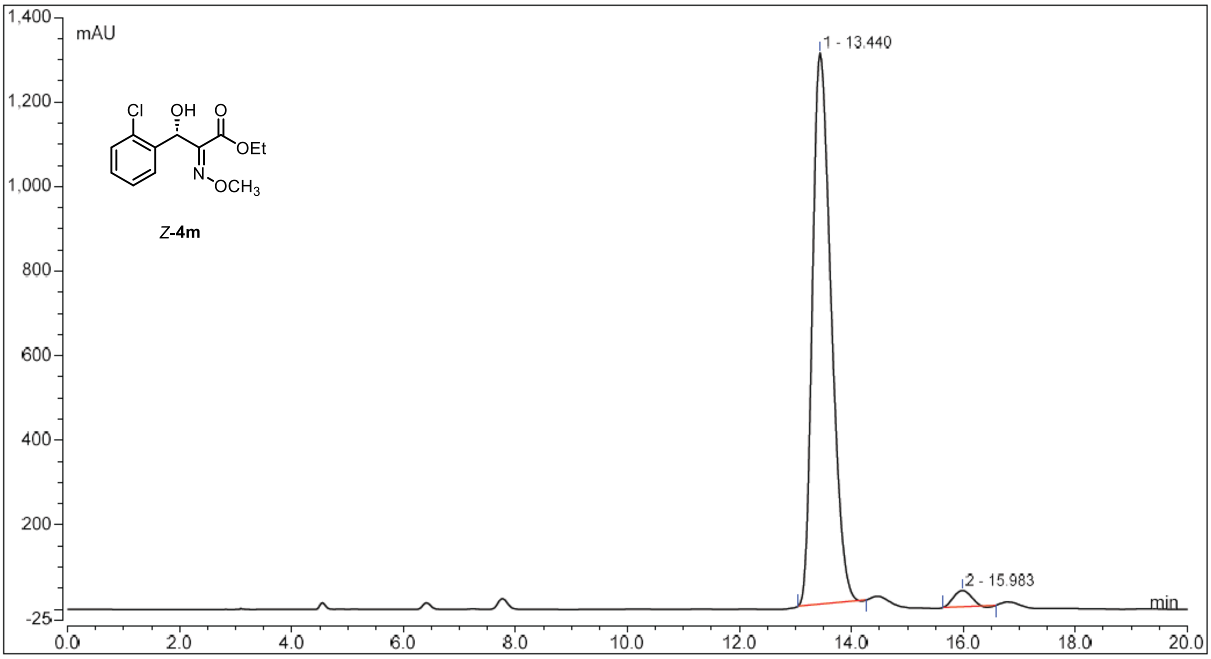

Peak	Retention Time (min)	Area (mAU × min)	Height (mAU)	Relative Area (%)
1	13.440	503.312	1304.219	97.44
2	15.983	13.266	38.162	2.56
Total		516.578	1342.381	100

HPLC analyses of racemic and enantiomerically enriched *Z*-4n

Chiralpak IA column, 250 × 4.6 mm, λ = 254 nm, 4% isopropyl alcohol in hexane, flow rate: 1.0 mL/min

Figure S41. HPLC chromatogram of (±)-*Z*-**4n**

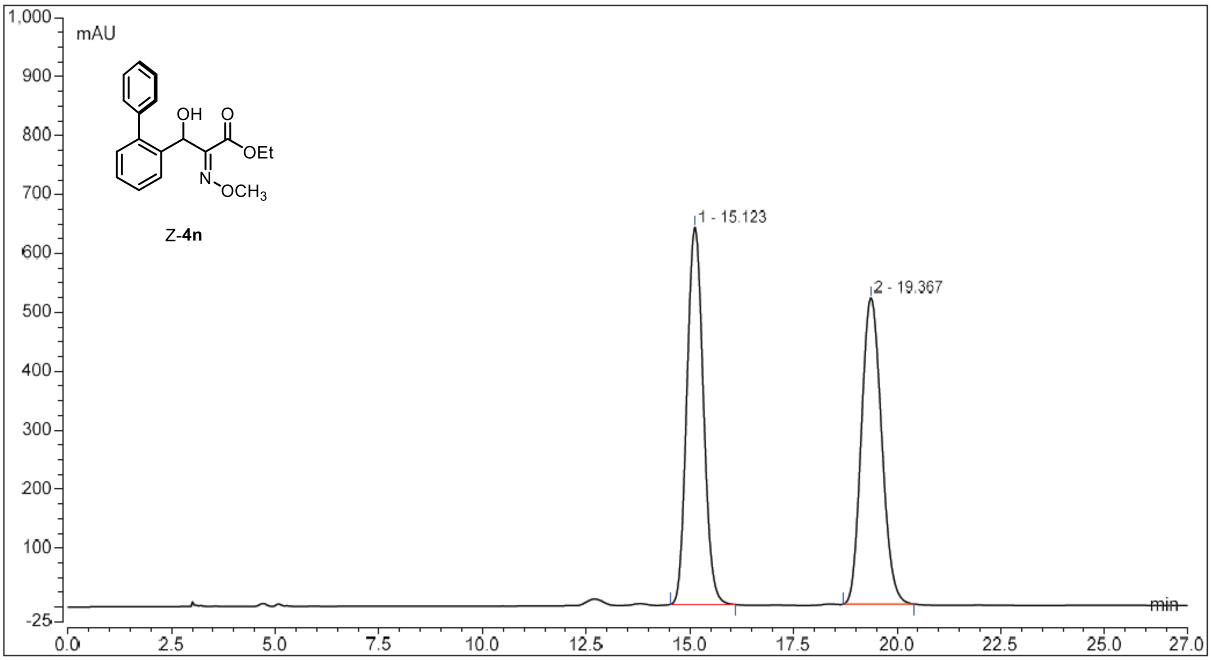

Peak	Retention Time (min)	Area (mAU × min)	Height (mAU)	Relative Area (%)
1	15.123	294.567	640.001	50.08
2	19.367	293.651	520.110	49.92
Total		588.218	1160.111	100

Figure S42. HPLC chromatogram of enantiomerically enriched *Z*-**4n**

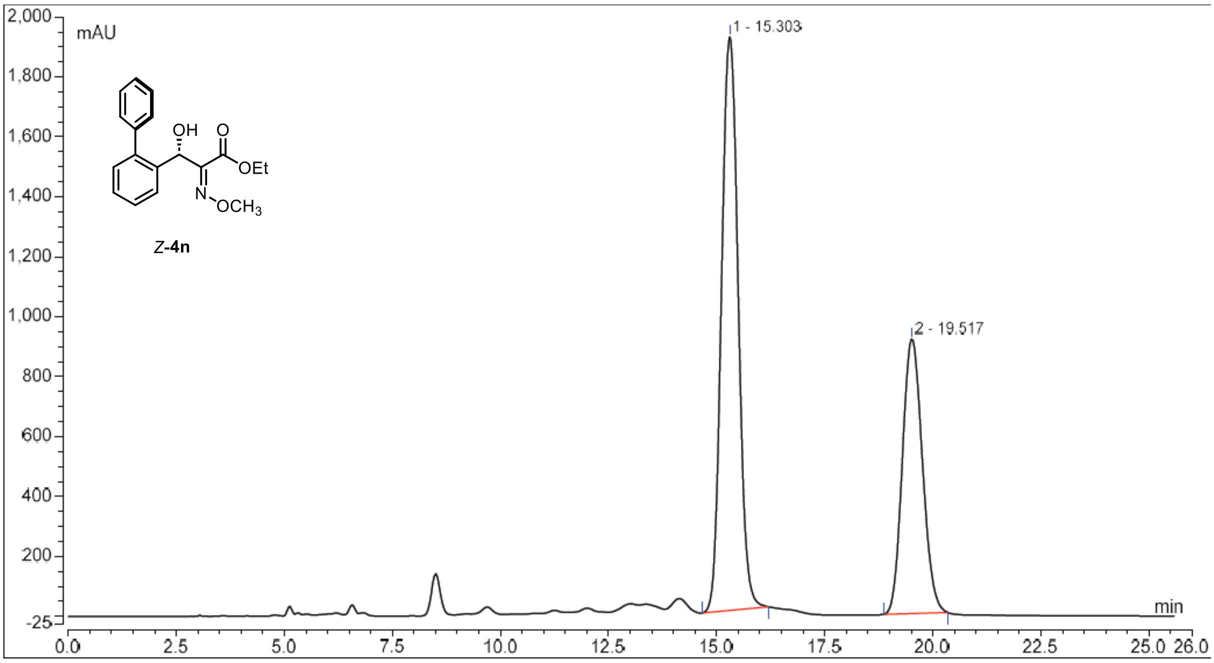

Peak	Retention Time (min)	Area (mAU × min)	Height (mAU)	Relative Area (%)
1	15.303	853.709	1913.257	63.45
2	19.517	491.776	916.754	36.55
Total		1345.485	2830.011	100

Figure S43. HPLC chromatogram of enantiomerically enriched *Z*-**4n** (using (*S*,*S*)-**2**)

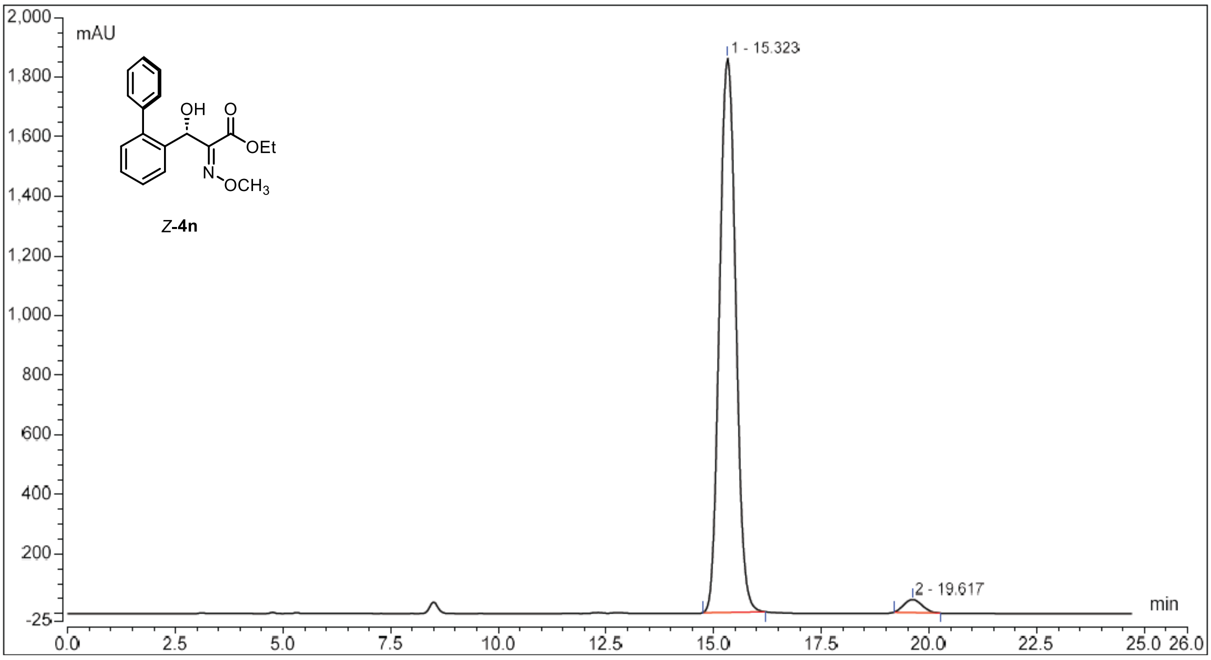

Peak	Retention Time (min)	Area (mAU × min)	Height (mAU)	Relative Area (%)
1	15.323	814.972	1859.255	97.45
2	19.617	21.339	43.733	2.55
Total		836.311	1902.988	100

HPLC analyses of racemic and enantiomerically enriched *Z*-4o

Chiralpak IH column, 250 × 4.6 mm, λ = 254 nm, 3% 2-propanol in hexane, flow rate: 1.0 mL/min

Figure S44. HPLC chromatogram of (±)-*Z*-**4o**

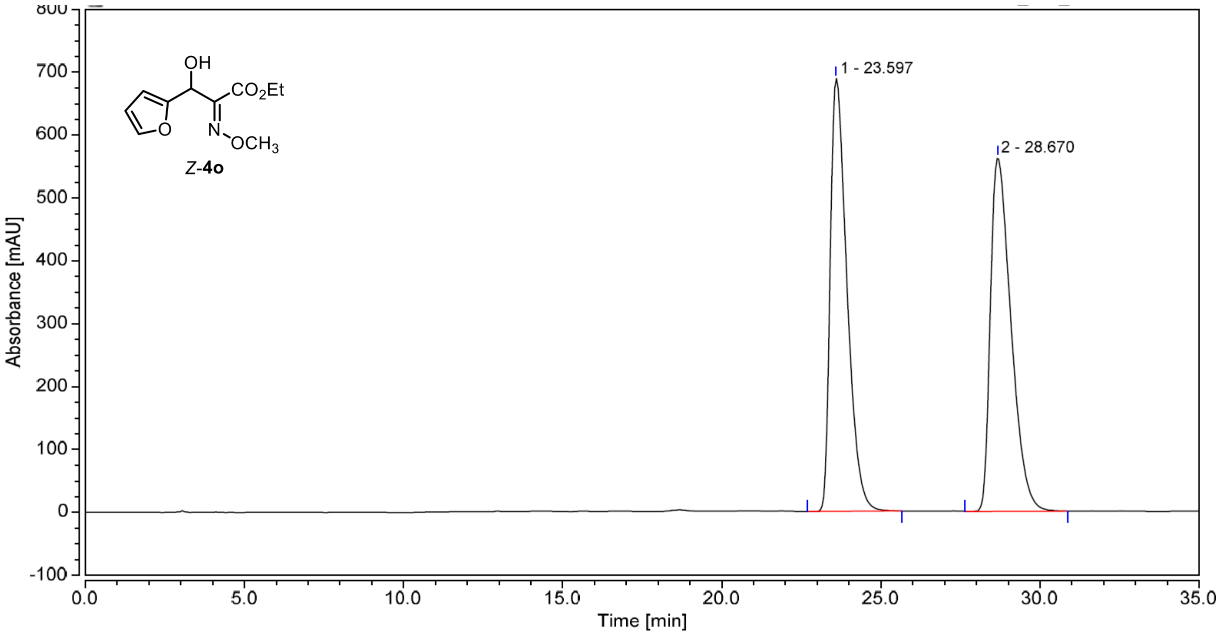

Peak	Retention Time (min)	Area (mAU × min)	Height (mAU)	Relative Area (%)
1	23.597	419.359	688.700	49.93
2	28.670	420.604	562.558	50.07
Total		839.962	1251.258	100.00

Figure S45. HPLC chromatogram of enantiomerically enriched *Z*-**4o**

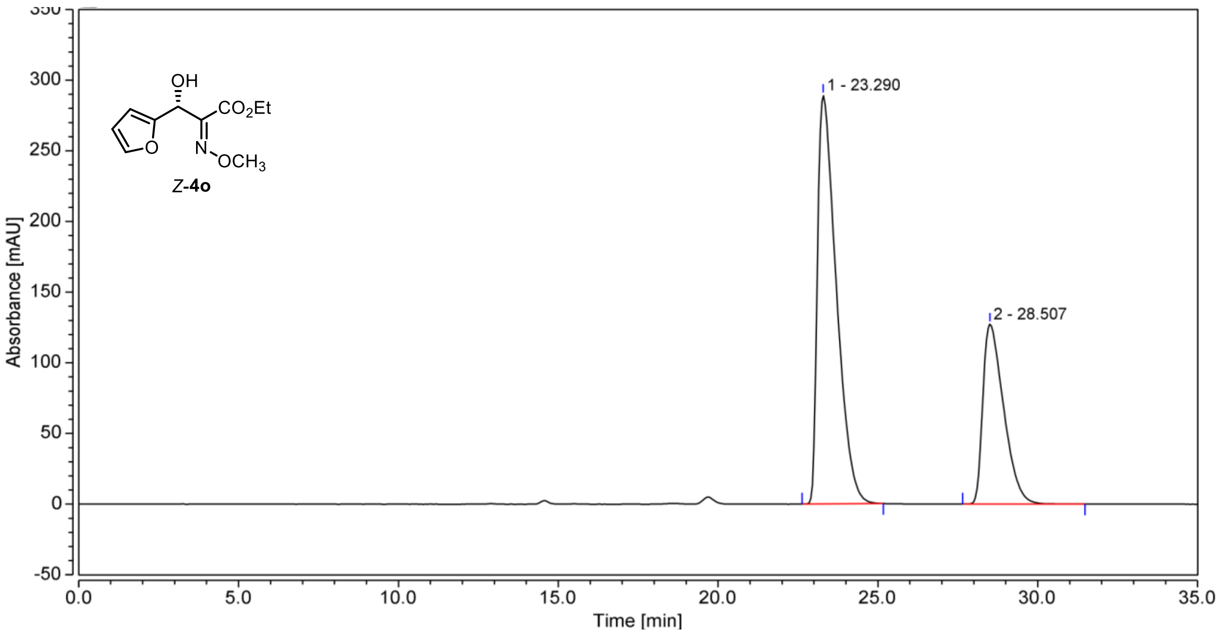

Peak	Retention Time (min)	Area (mAU × min)	Height (mAU)	Relative Area (%)
1	23.290	197.677	289.220	67.28
2	28.507	96.142	127.374	32.72
Total		293.819	416.594	100.00

HPLC analyses of racemic and enantiomerically enriched *E*-4o

Chiralpak IH column, 250 × 4.6 mm, λ = 254 nm, 3% 2-propanol in hexane, flow rate: 1.0 mL/min

Figure S46. HPLC chromatogram of (±)-*E*-**4o**

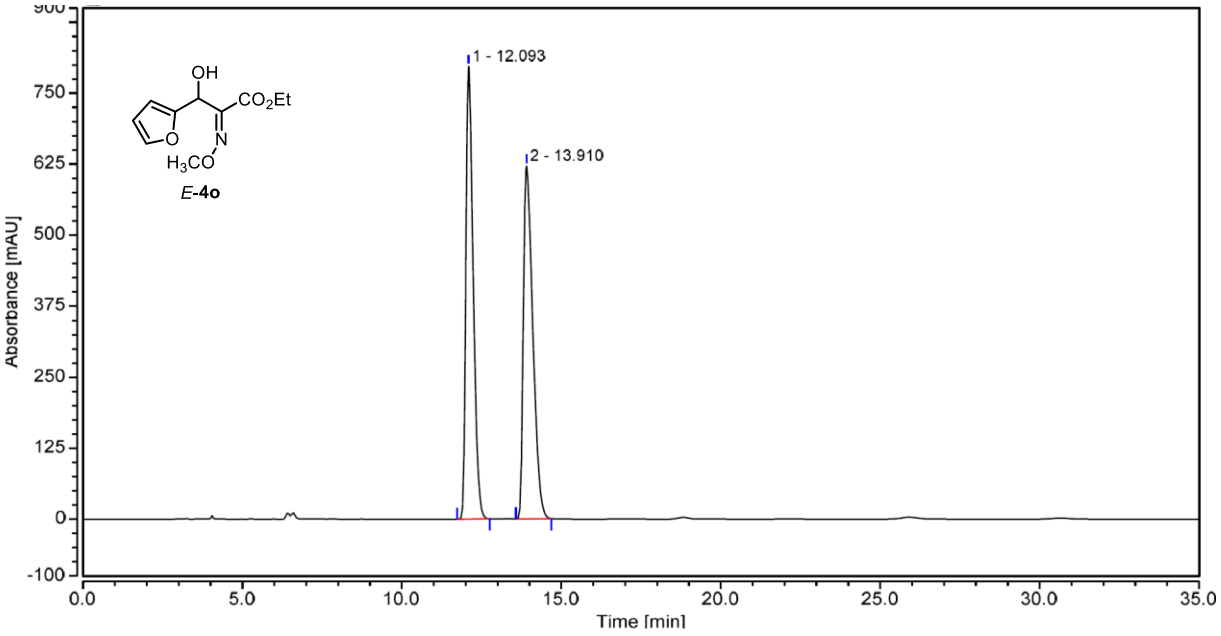

Peak	Retention Time (min)	Area (mAU × min)	Height (mAU)	Relative Area (%)
1	12.093	212.391	797.017	49.95
2	13.910	212.828	621.211	50.05
Total		425.219	1418.228	100.00

Figure S47. HPLC chromatogram of enantiomerically enriched *E*-**4o**

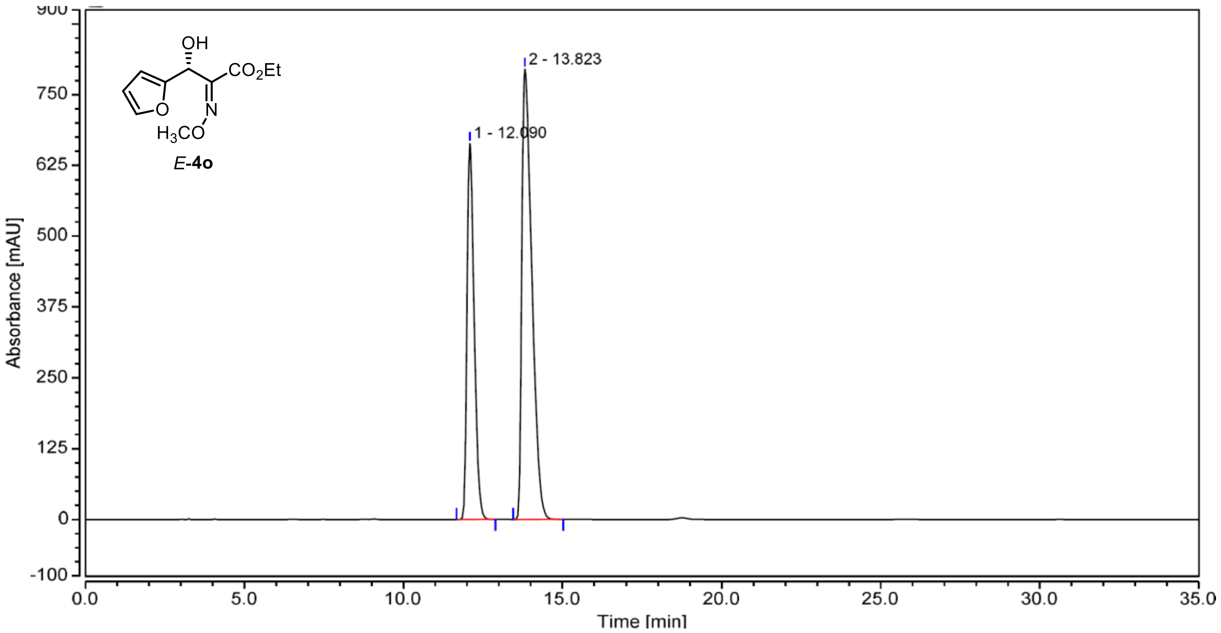

Peak	Retention Time (min)	Area (mAU × min)	Height (mAU)	Relative Area (%)
1	12.090	173.904	664.175	37.55
2	13.823	289.244	795.177	62.45
Total		463.149	1459.352	100.00

HPLC analyses of racemic and enantiomerically enriched *Z*-4p

Chiralpak IH column, 250 × 4.6 mm, λ = 210 nm, 3% 2-propanol in hexane, flow rate: 1.0 mL/min

Figure S48. HPLC chromatogram of (±)-*Z*-**4p**

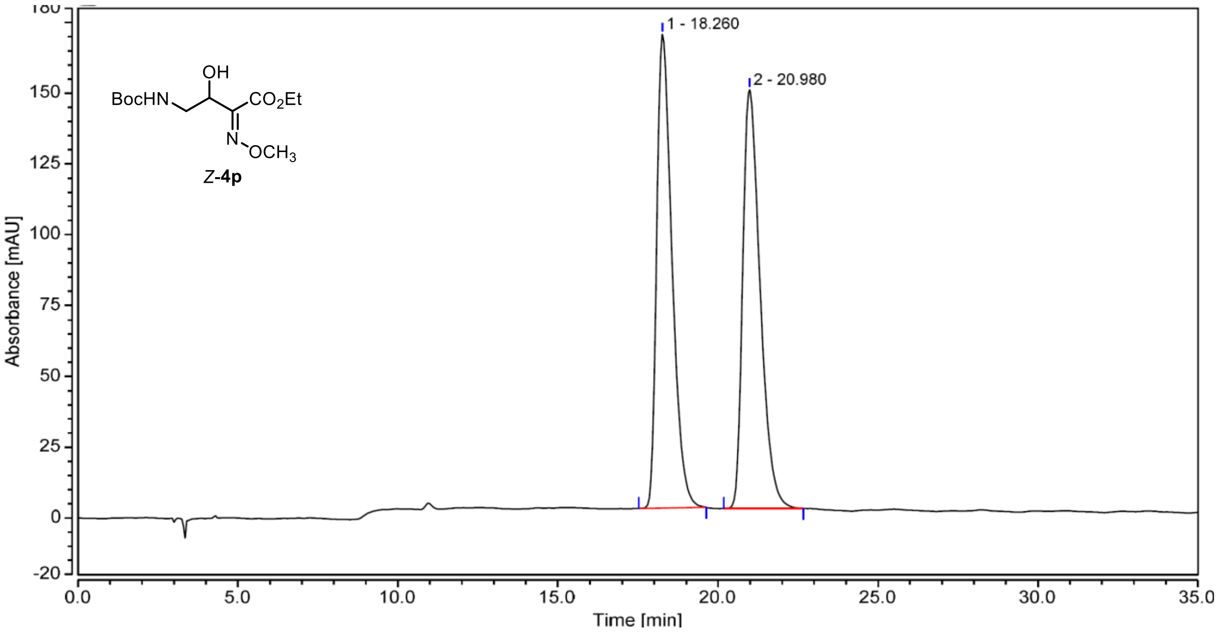

Peak	Retention Time (min)	Area (mAU × min)	Height (mAU)	Relative Area (%)
1	18.260	92.977	167.391	49.98
2	20.980	93.053	147.824	50.02
Total		186.030	315.215	100.00

Figure S49. HPLC chromatogram of enantiomerically enriched *Z*-**4p**

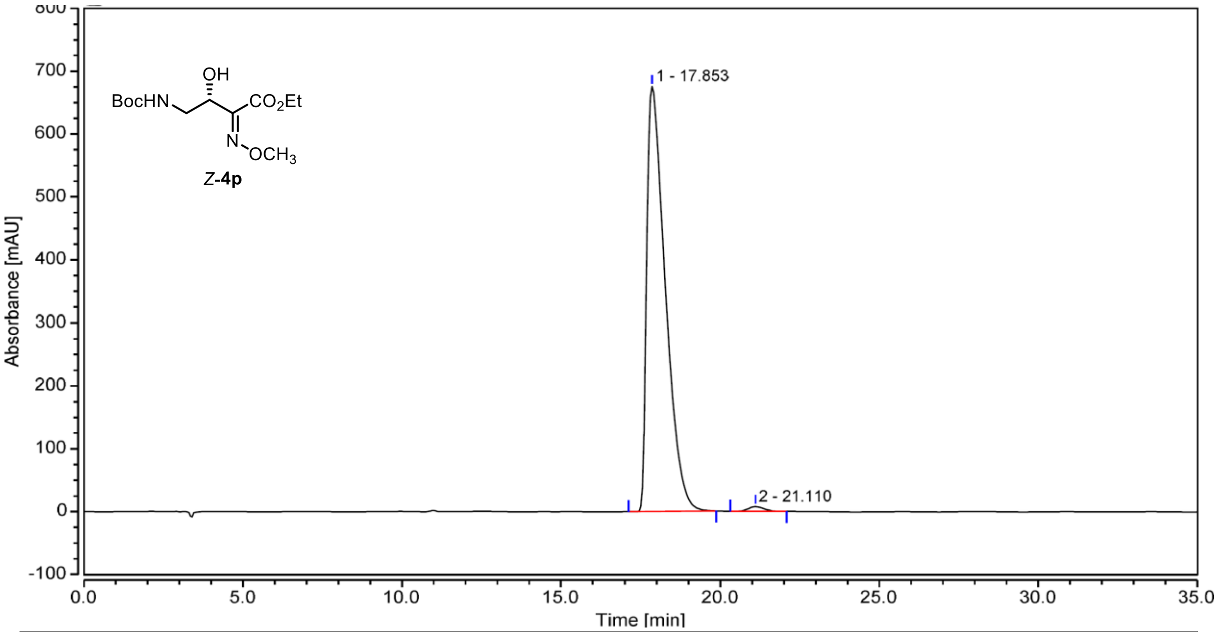

Peak	Retention Time (min)	Area (mAU × min)	Height (mAU)	Relative Area (%)
1	17.853	455.921	675.605	99.07
2	21.110	4.271	7.499	0.93
Total		460.192	683.104	100.00

HPLC analyses of racemic and enantiomerically enriched *Z*-4q

Chiralpak IH column, 250 × 4.6 mm, λ = 225 nm, 3% 2-propanol in hexane, flow rate: 1.0 mL/min

Figure S50. HPLC chromatogram of (±)-*Z*-**4q**

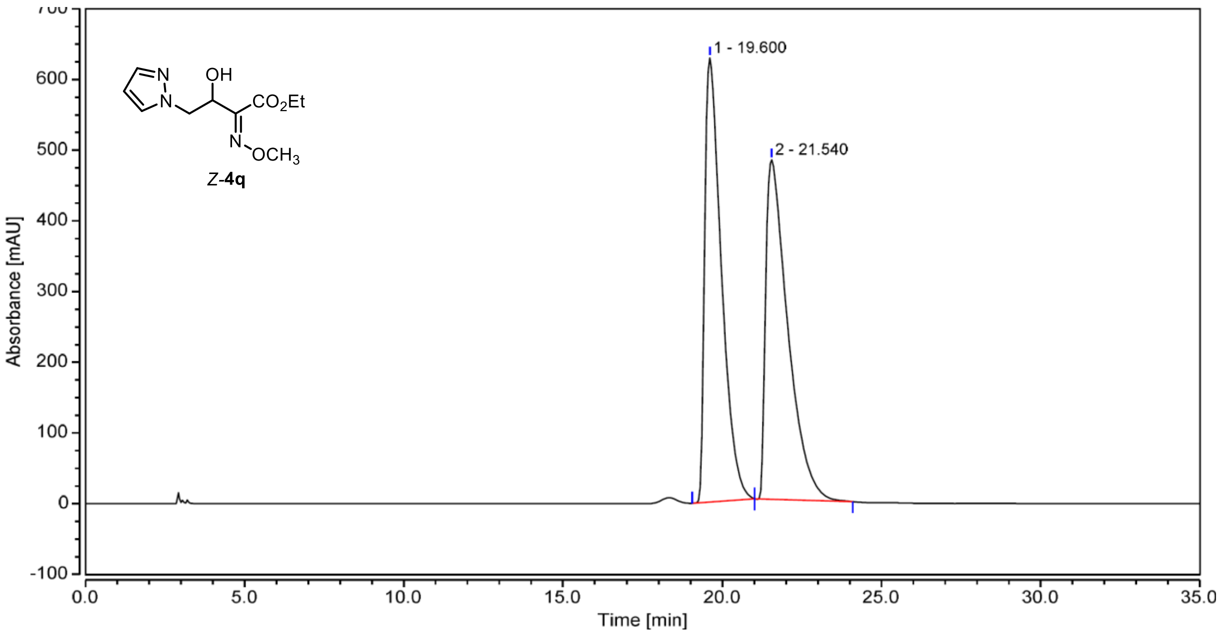

Peak	Retention Time (min)	Area (mAU × min)	Height (mAU)	Relative Area (%)
1	19.600	381.714	628.038	49.66
2	21.540	387.006	480.317	50.34
Total		768.720	1108.356	100.00

Figure S51. HPLC chromatogram of enantiomerically enriched *Z*-**4q**

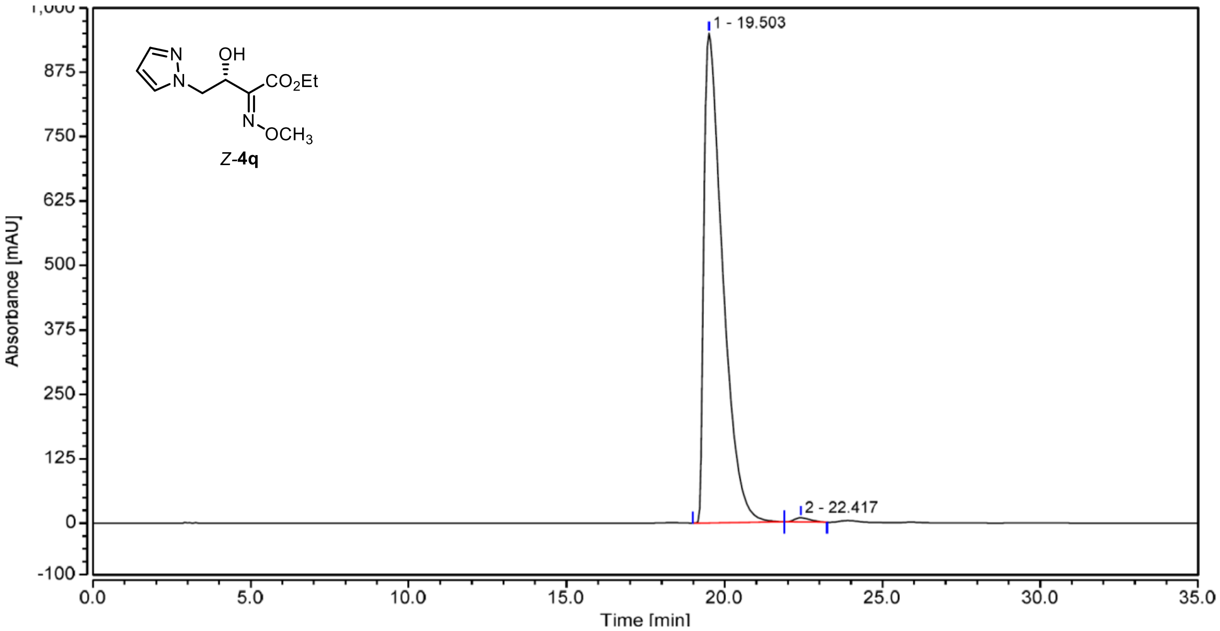

Peak	Retention Time (min)	Area (mAU × min)	Height (mAU)	Relative Area (%)
1	19.503	653.806	950.652	99.26
2	22.417	4.860	8.177	0.74
Total		658.666	958.830	100.00

HPLC analyses of racemic and enantiomerically enriched *Z*-4r

Chiralpak IH column, 250 × 4.6 mm, λ = 254 nm, 3% 2-propanol in hexane, flow rate: 1.0 mL/min

Figure S52. HPLC chromatogram of (±)-*Z*-**4r**

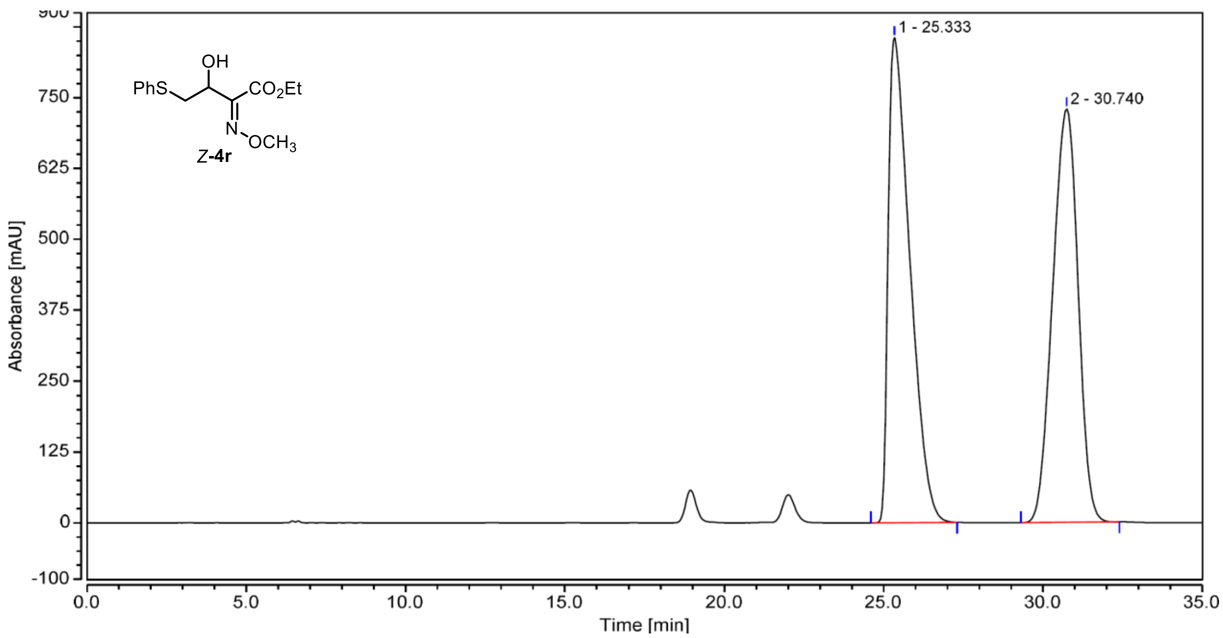

Peak	Retention Time (min)	Area (mAU × min)	Height (mAU)	Relative Area (%)
1	25.333	674.045	856.079	50.01
2	30.740	673.650	729.690	49.99
Total		1347.696	1585.769	100.00

Figure S53. HPLC chromatogram of enantiomerically enriched *Z*-**4r**

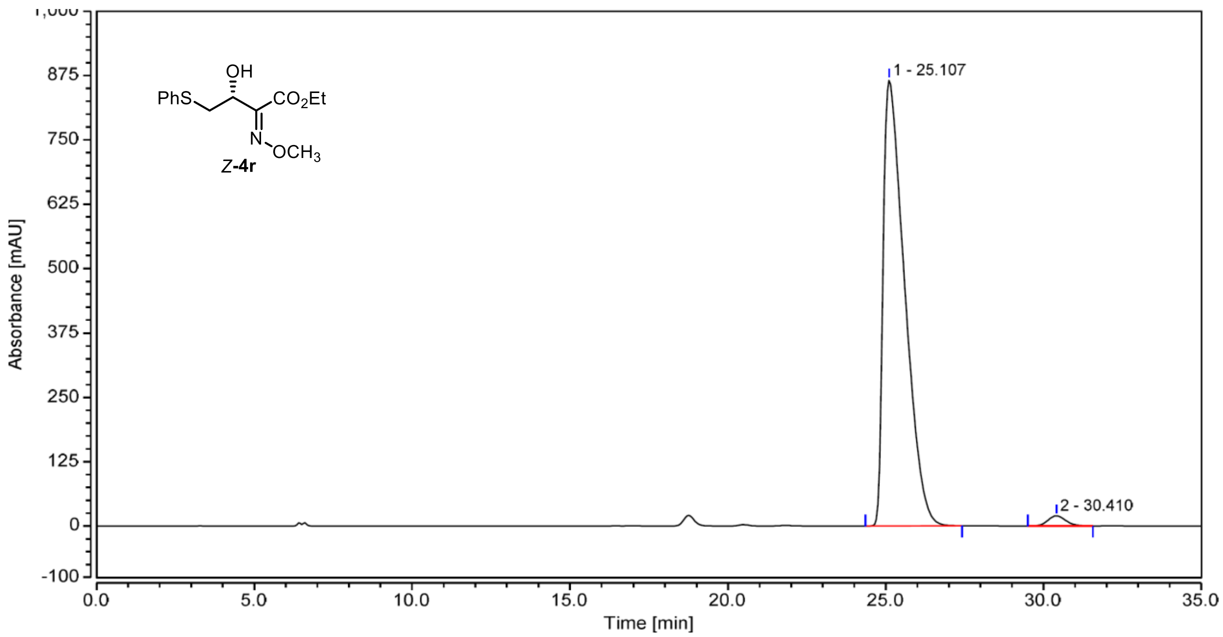

Peak	Retention Time (min)	Area (mAU × min)	Height (mAU)	Relative Area (%)
1	25.107	681.989	865.957	98.15
2	30.410	12.820	19.755	1.85
Total		694.809	885.713	100.00

HPLC analyses of racemic and enantiomerically enriched 30

Chiralpak IC column, 250 × 4.6 mm, λ = 254 nm, 1% 2-propanol in hexane, flow rate: 1.0 mL/min

Figure S54. HPLC chromatogram of (±)-**30**

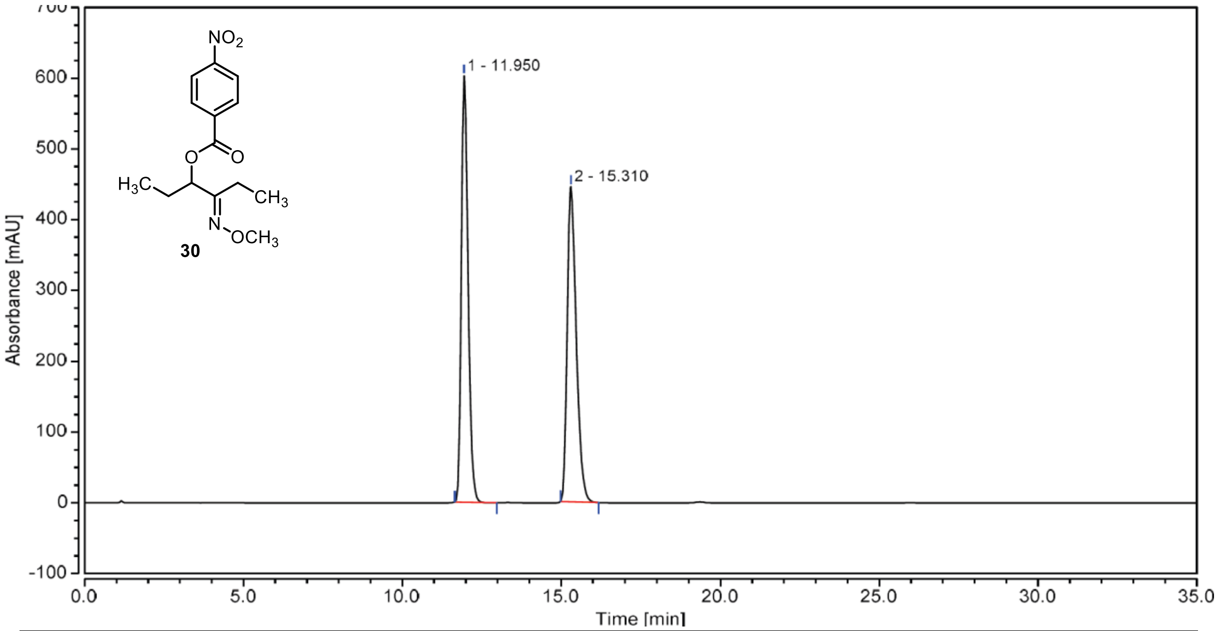

Peak	Retention Time (min)	Area (mAU × min)	Height (mAU)	Relative Area (%)
1	11.950	148.359	602.441	50.09
2	15.310	147.841	445.329	49.91
Total		296.200	1047.770	100.00

Figure S55. HPLC chromatogram of enantiomerically enriched **30**

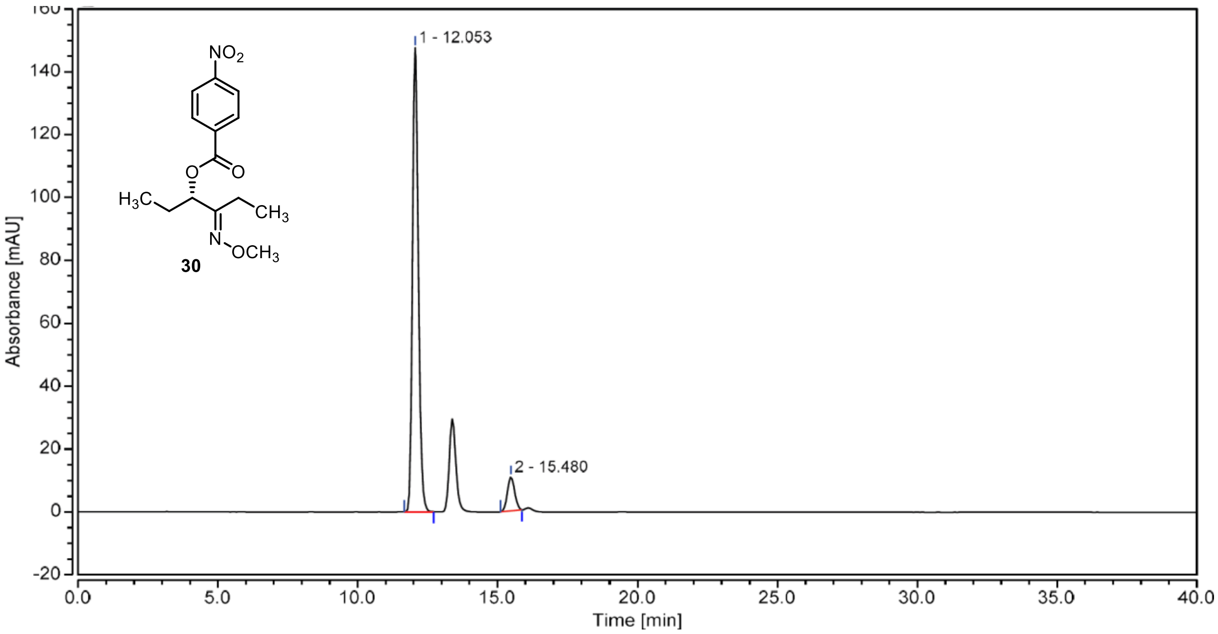

Peak	Retention Time (min)	Area (mAU × min)	Height (mAU)	Relative Area (%)
1	12.053	35.689	147.657	91.58
2	15.480	3.281	10.810	8.42
Total		38.970	158.467	100.00

HPLC analyses of racemic and enantiomerically enriched 13

Chiralpak OJ column, 250 × 4.6 mm, λ = 210 nm, 0.1% ethanol in hexane, flow rate: 1.0 mL/min

Figure S56. HPLC chromatogram of (±)-**13**

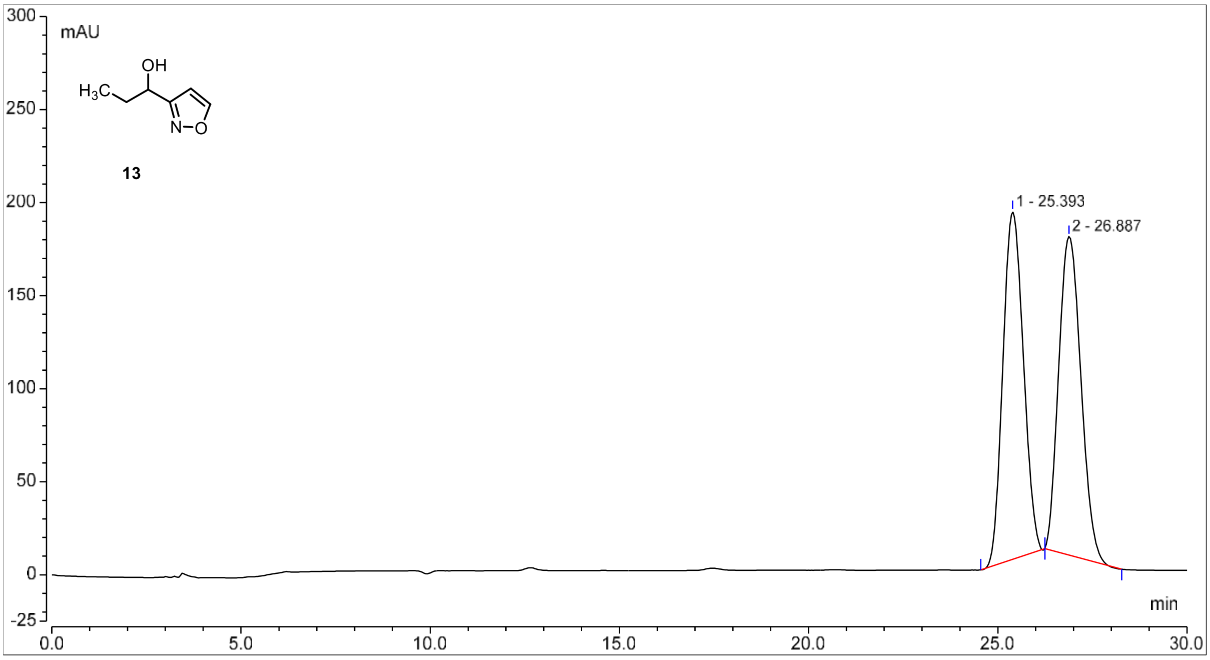

Peak	Retention Time (min)	Area (mAU × min)	Height (mAU)	Relative Area (%)
1	25.393	117.235	186.829	50.16
2	26.887	116.508	171.290	49.84
Total		233.743	358.119	100.00

Figure S57. HPLC chromatogram of enantiomerically enriched **13**

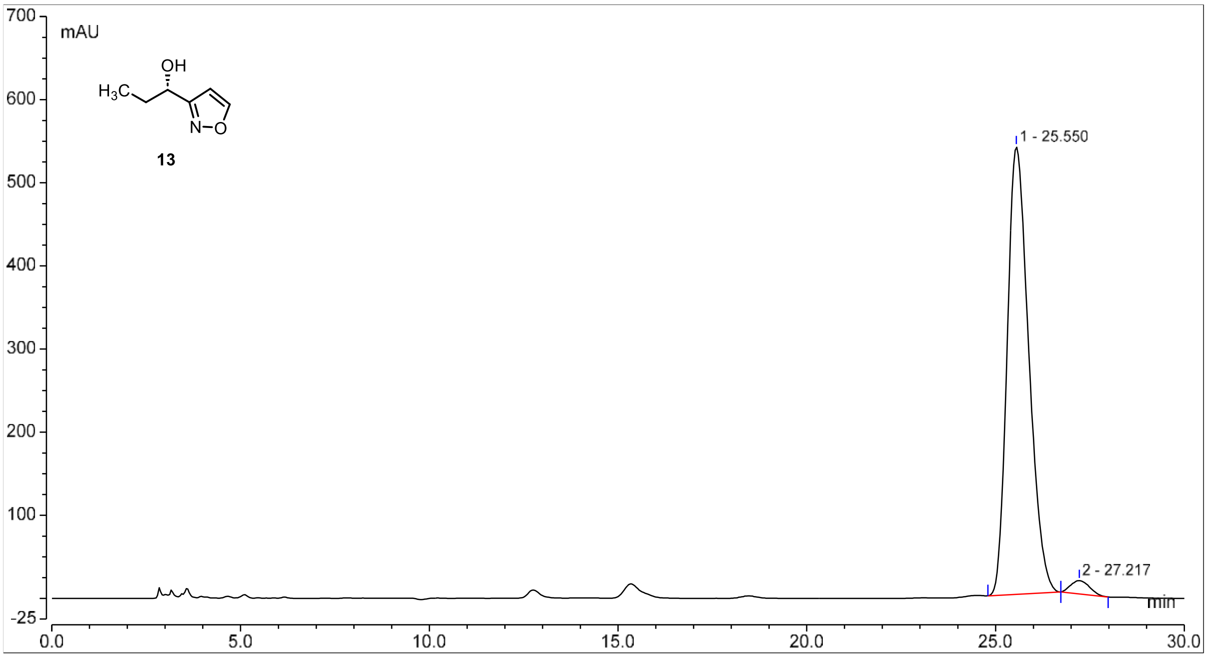

Peak	Retention Time (min)	Area (mAU × min)	Height (mAU)	Relative Area (%)
1	25.550	356.179	538.205	97.47
2	27.217	9.263	15.905	2.53
Total		365.442	554.110	100.00

GC analyses of racemic and enantiomerically enriched 11

BetaDex 120 column, 127 °C for 60 mins, flow rate 20 cm/sec

Figure S58. GC chromatogram of (±)-**11**

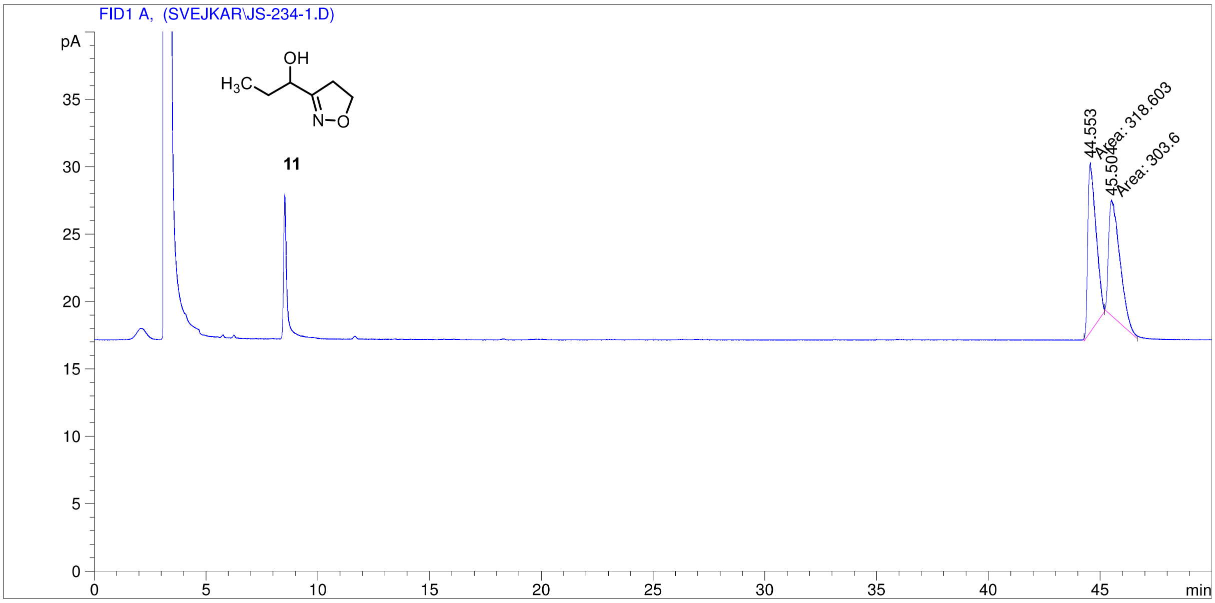

Peak	Retention Time (min)	Area (mAU × min)	Height (mAU)	Relative Area (%)
1	44.553	318.603	12.577	51.21
2	45.504	303.600	8.553	48.79
Total		622.203	21.130	100.00

Figure S59. GC chromatogram of enantiomerically enriched **11**

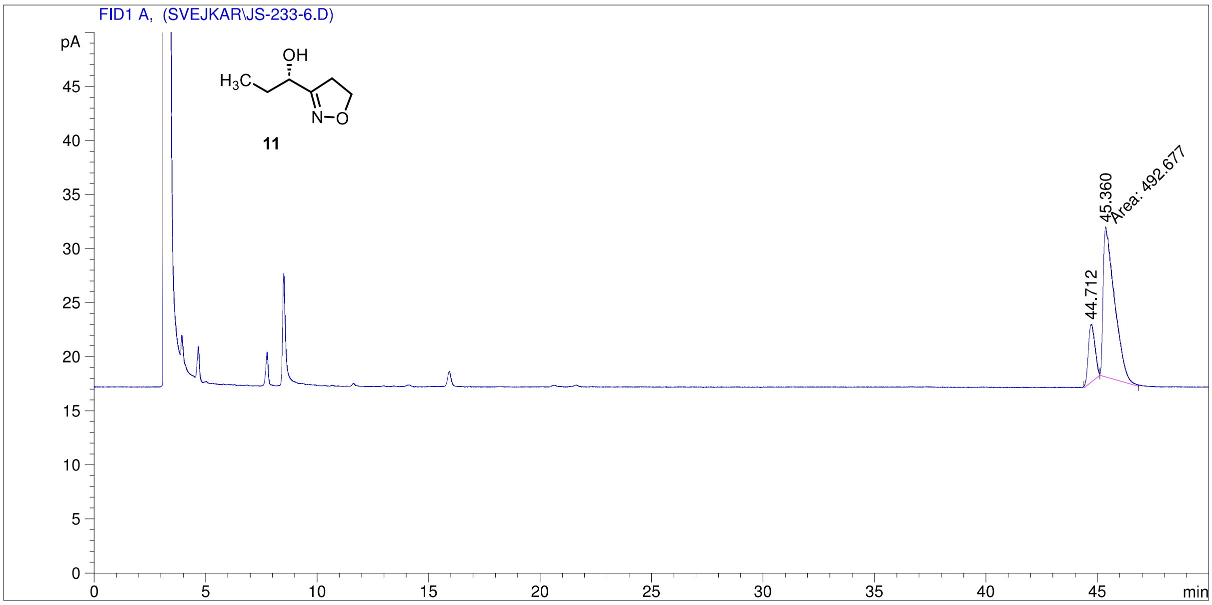

Peak	Retention Time (min)	Area (mAU × min)	Height (mAU)	Relative Area (%)
1	44.712	110.237	5.374	18.28
2	45.360	492.677	13.867	81.72
Total		602.773	19.241	100.00

GC analyses of racemic and enantiomerically enriched 6a

BetaDex 120 column, 95 °C for 60 mins, then 200 °C for 3 min, flow rate 20 cm/sec

Figure S60. GC chromatogram of (±)-**6a**

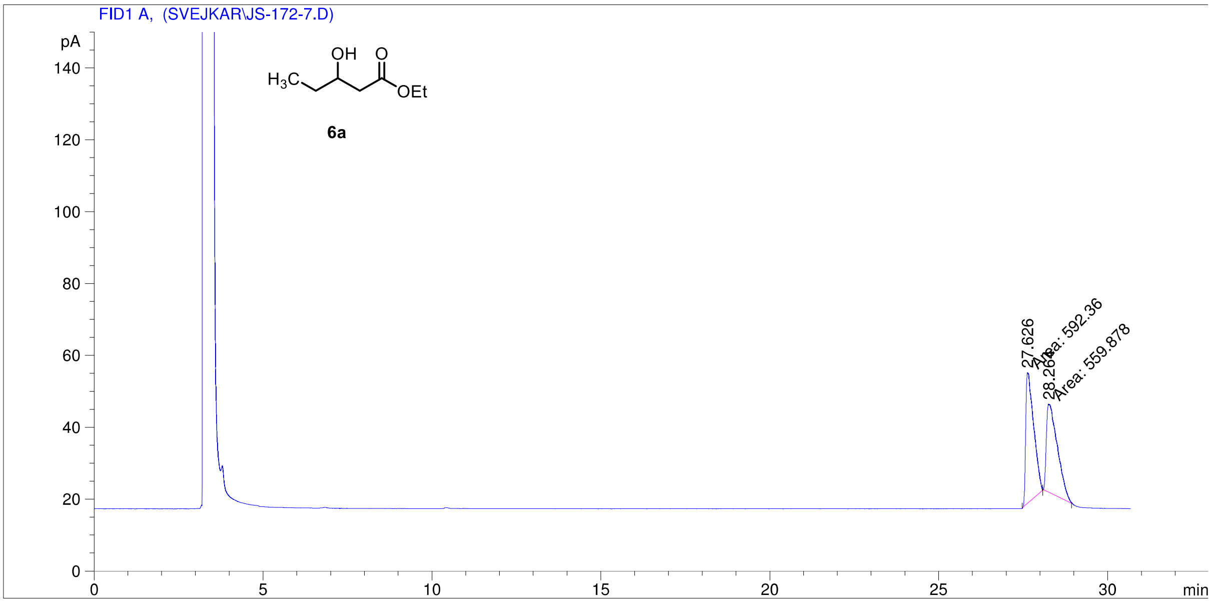

Peak	Retention Time (min)	Area (mAU × min)	Height (mAU)	Relative Area (%)
1	27.626	592.360	36.428	51.41
2	28.264	559.878	24.516	48.59
Total		1152.238	60.944	100

Figure S61. GC chromatogram of enantiomerically enriched **6a**

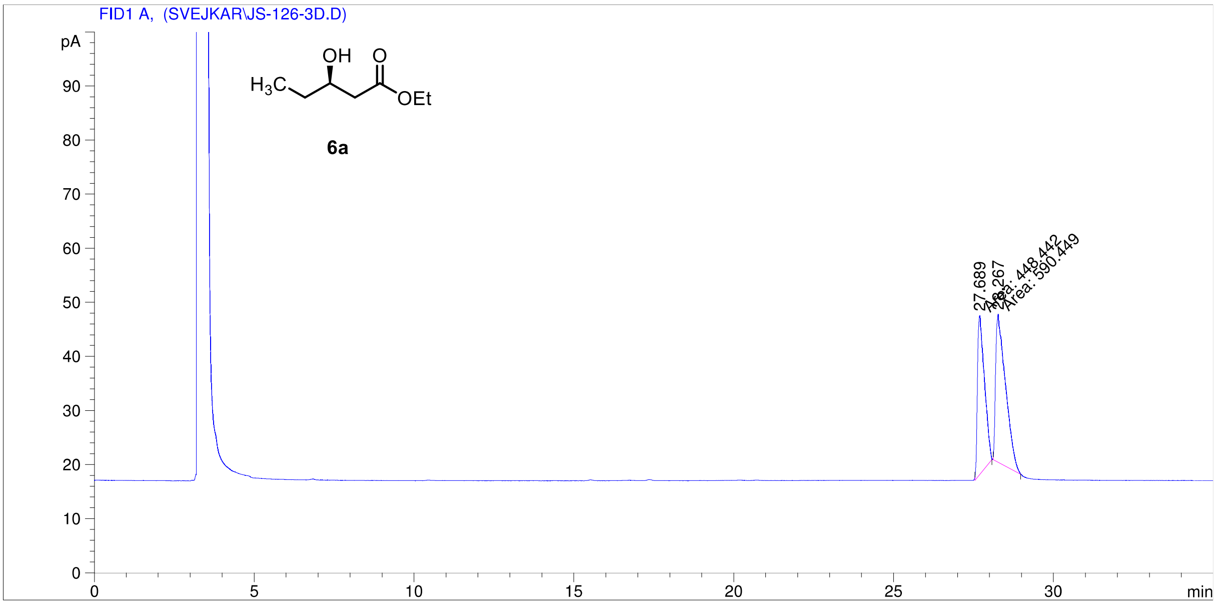

Peak	Retention Time (min)	Area (mAU × min)	Height (mAU)	Relative Area (%)
1	27.689	448.442	29.388	43.17
2	28.267	590.449	27.354	56.83
Total		1038.891	56.742	100

HPLC analyses of racemic and enantiomerically enriched 16

Chiralpak IA column, 250 × 4.6 mm, λ = 254 nm, 2% isopropyl alcohol in hexane, flow rate: 1.0 mL/min

Figure S62. HPLC chromatogram of (±)-**16**

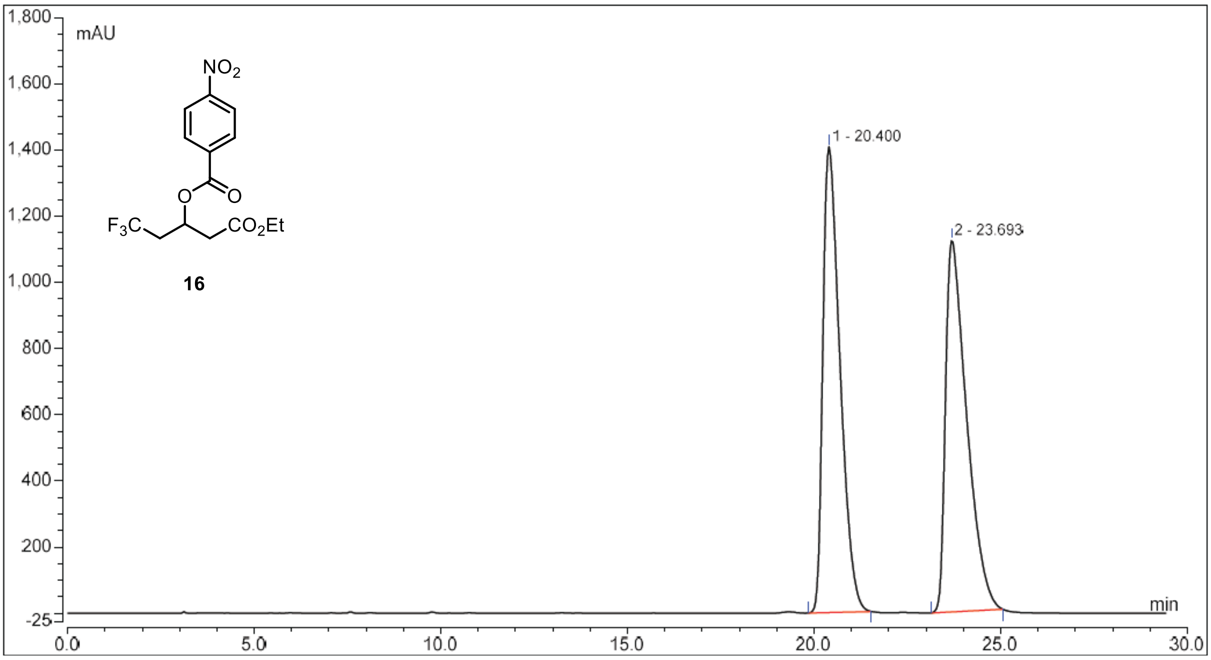

Peak	Retention Time (min)	Area (mAU × min)	Height (mAU)	Relative Area (%)
1	20.400	746.635	1407.730	50.36
2	23.693	735.821	1122.960	49.64
Total		1482.456	2530.690	100

Figure S63. HPLC chromatogram of enantiomerically enriched **16**

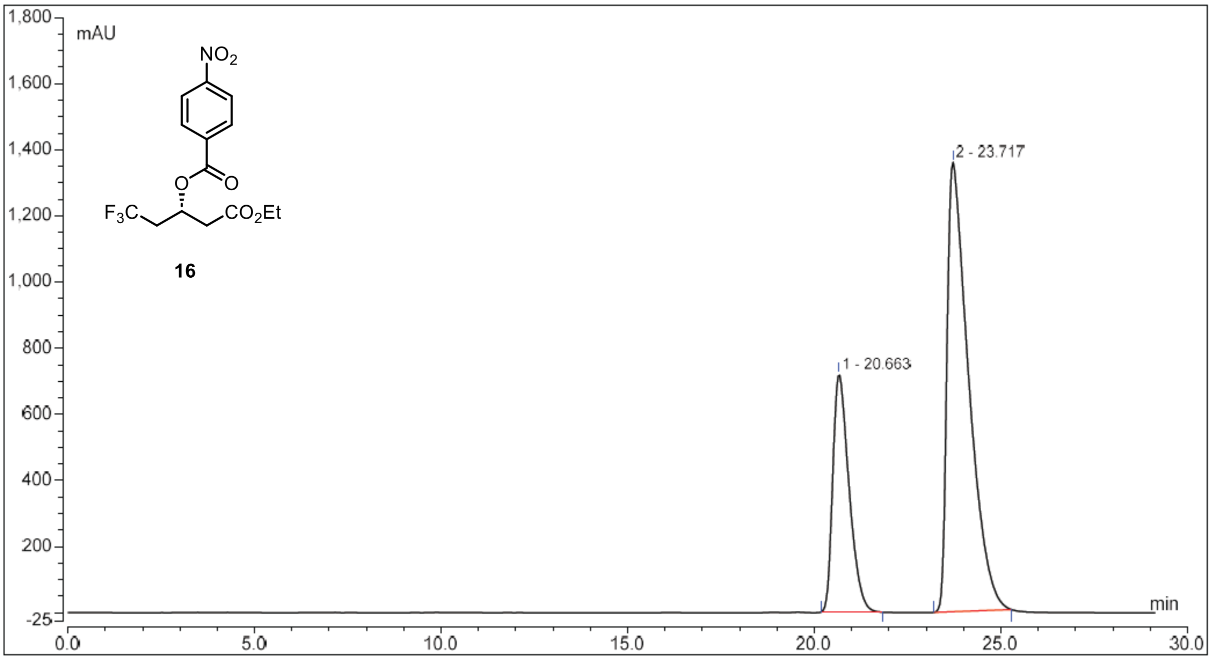

Peak	Retention Time (min)	Area (mAU × min)	Height (mAU)	Relative Area (%)
1	20.663	363.273	718.916	28.50
2	23.717	911.293	1360.012	71.50
Total		1274.566	2078.928	100

GC analyses of racemic and enantiomerically enriched 6c
BetaDex 120 column, 95 °C for 60 mins, flow rate 20 cm/sec
Figure S64. GC chromatogram of (±)-**6c**

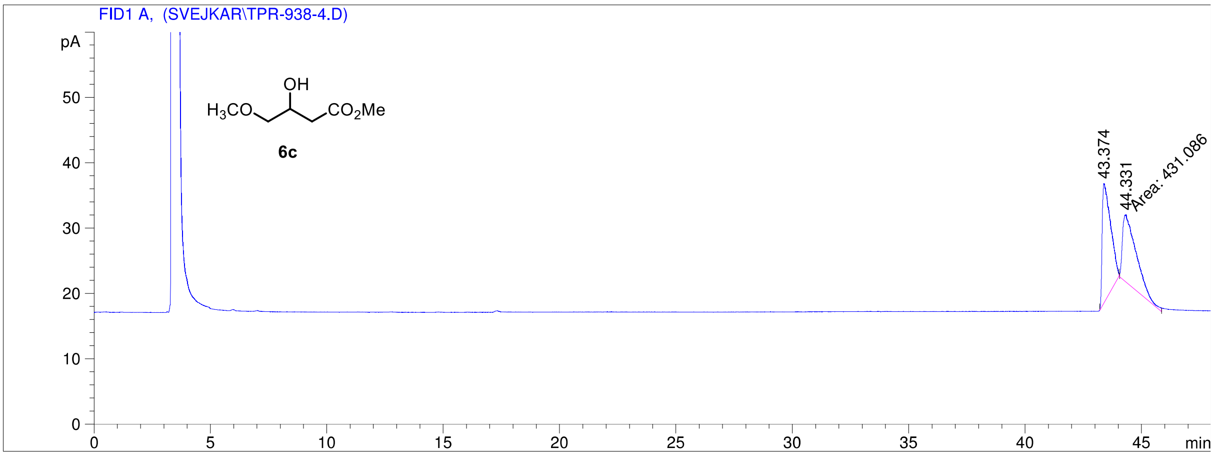

Peak	Retention Time (min)	Area (mAU × min)	Height (mAU)	Relative Area (%)
1	43.374	456.849	18.395	51.451
2	44.331	431.086	10.337	48.549
Total		887.935	28.732	100

Figure S65. GC chromatogram of enantiomerically enriched **6c**

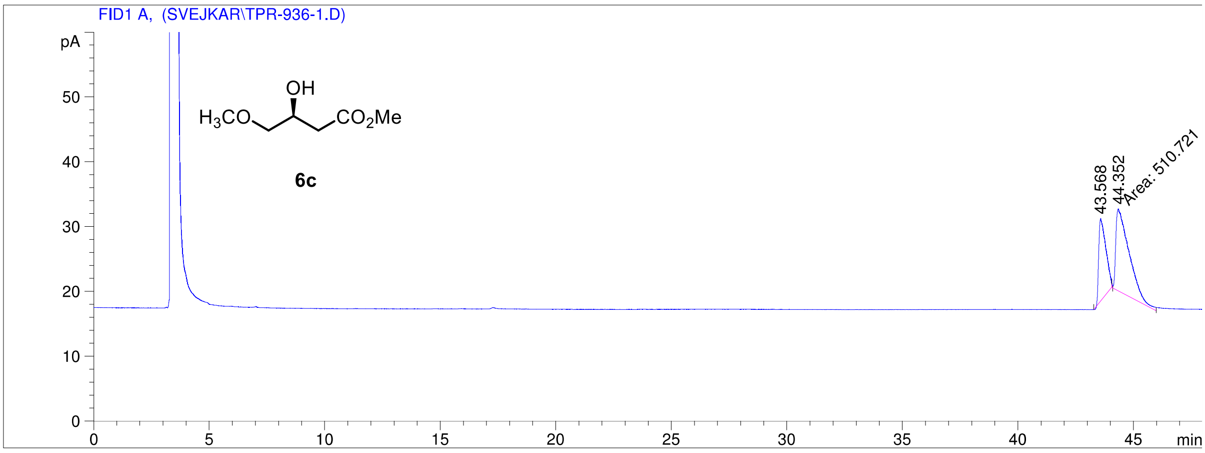

Peak	Retention Time (min)	Area (mAU × min)	Height (mAU)	Relative Area (%)
1	43.568	269.290	12.766	34.524
2	44.352	510.721	12.651	65.476
Total		780.011	25.417	100

HPLC analyses of racemic and enantiomerically enriched 18

Chiralpak IA column, 250 × 4.6 mm, λ = 254 nm, 3% isopropyl alcohol in hexane, flow rate: 1.0 mL/min

Figure S66. HPLC chromatogram of (±)-**18**

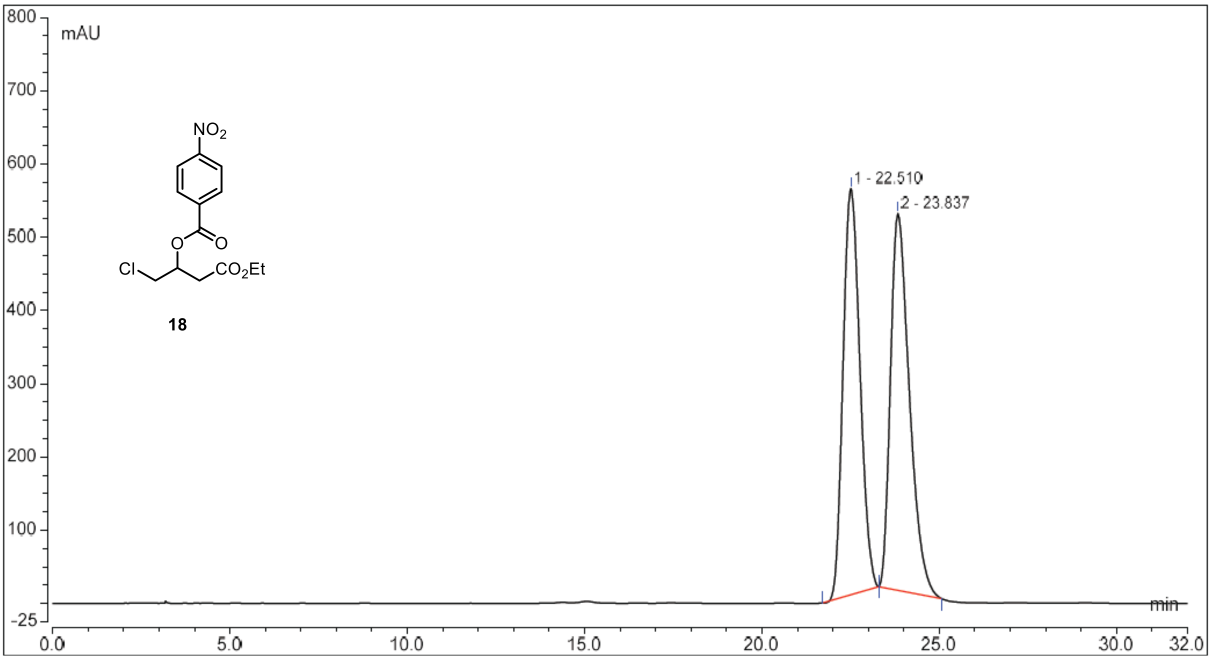

Peak	Retention Time (min)	Area (mAU × min)	Height (mAU)	Relative Area (%)
1	22.510	311.388	554.686	49.86
2	23.837	312.401	514.714	50.14
Total		623.789	1069.400	100

Figure S67. HPLC chromatogram of enantiomerically enriched **18**

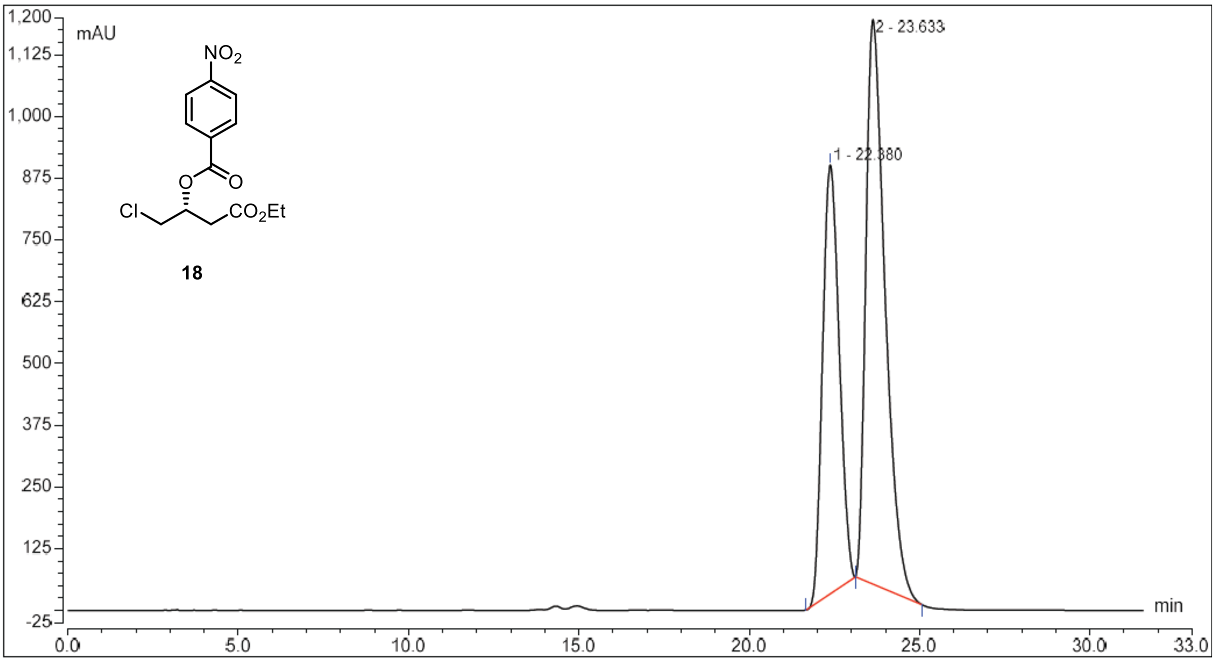

Peak	Retention Time (min)	Area (mAU × min)	Height (mAU)	Relative Area (%)
1	22.380	489.862	868.276	39.56
2	23.633	748.559	1142.567	60.44
Total		1238.421	2010.843	100

HPLC analyses of racemic and enantiomerically enriched 20

Chiralpak IA column, 250 × 4.6 mm, λ = 254 nm, 2% isopropyl alcohol in hexane, flow rate: 1.0 mL/min

Figure S68. HPLC chromatogram of (±)-**20**

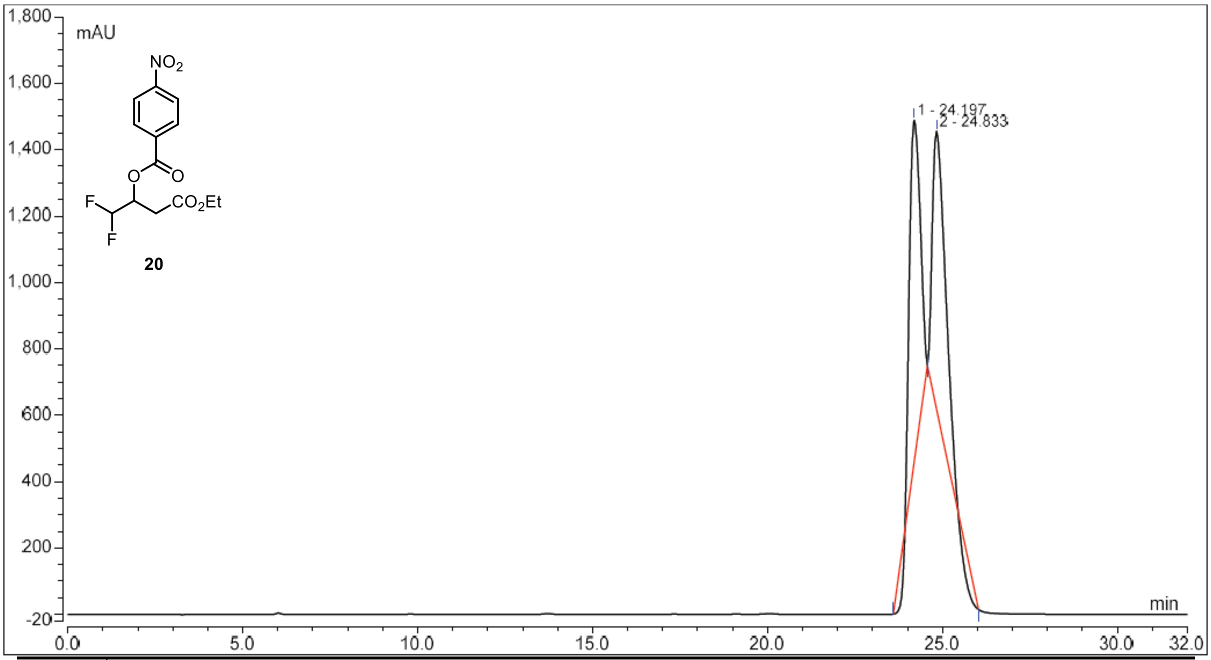

Peak	Retention Time (min)	Area (mAU × min)	Height (mAU)	Relative Area (%)
1	24.197	338.965	1025.561	50.03
2	24.833	338.497	837.404	49.97
Total		677.462	1862.965	100

Figure S69. HPLC chromatogram of enantiomerically enriched **20**

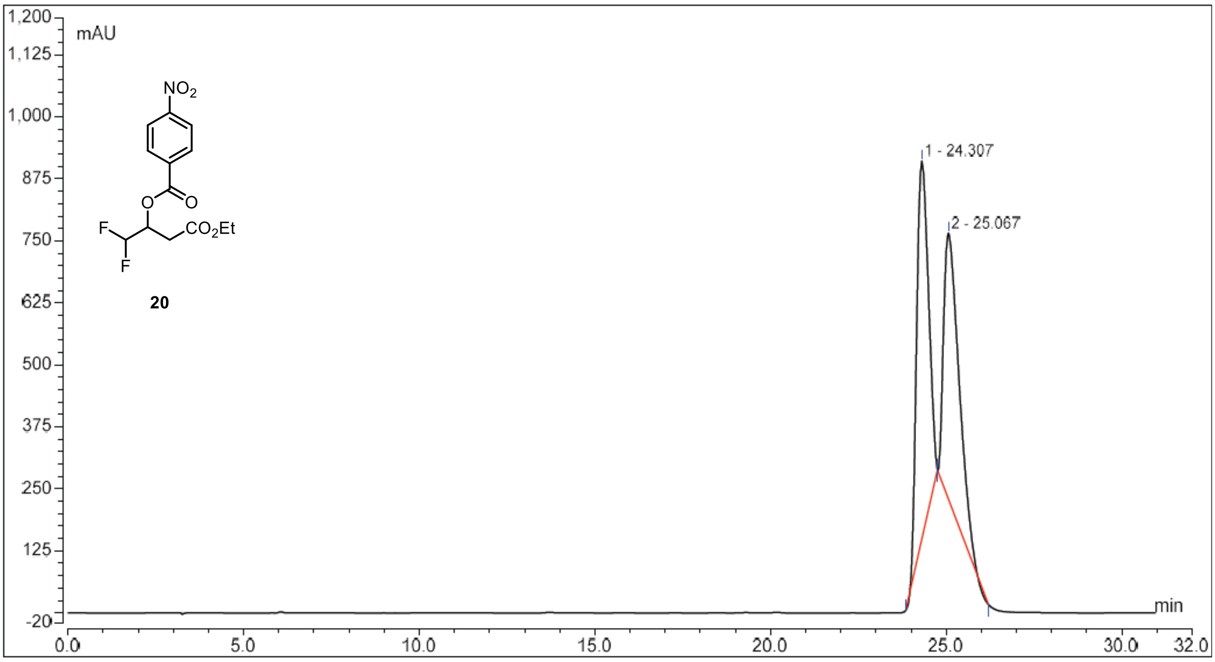

Peak	Retention Time (min)	Area (mAU × min)	Height (mAU)	Relative Area (%)
1	24.307	295.074	762.067	52.06
2	25.067	271.728	536.010	47.94
Total		566.802	1298.077	100

GC analyses of racemic and enantiomerically enriched 6f

BetaDex 120 column, conditions: 100 °C for 25 mins, flow rate 20 cm/sec

Figure S70. GC chromatogram of (±)-**6f**

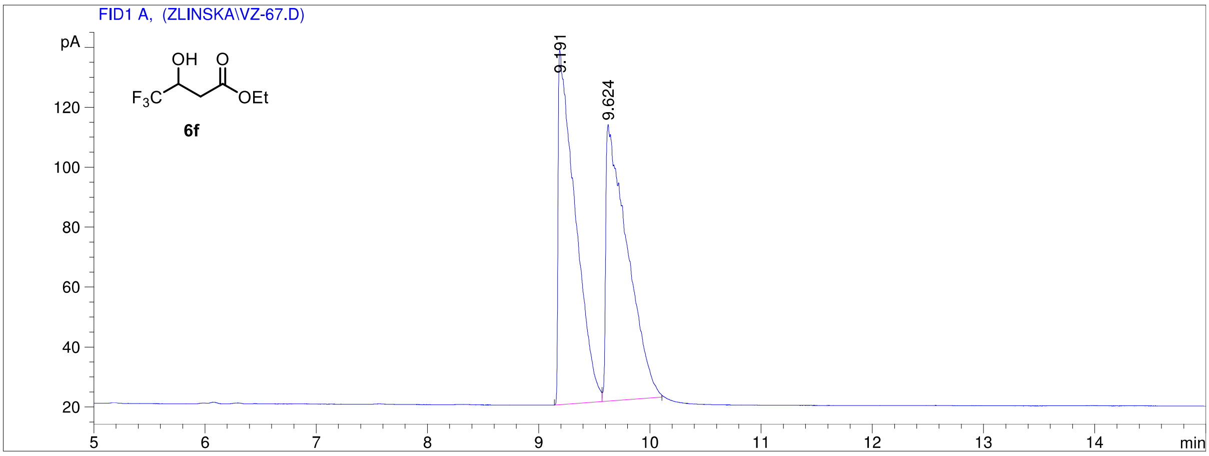

Peak	Retention Time (min)	Area (mAU × min)	Height (mAU)	Relative Area (%)
1	9.191	1258.303	118.038	50.245
2	9.624	1246.005	92.223	49.755
Total		2504.308	210.261	100

Figure S71. GC chromatogram of enantiomerically enriched **6f**

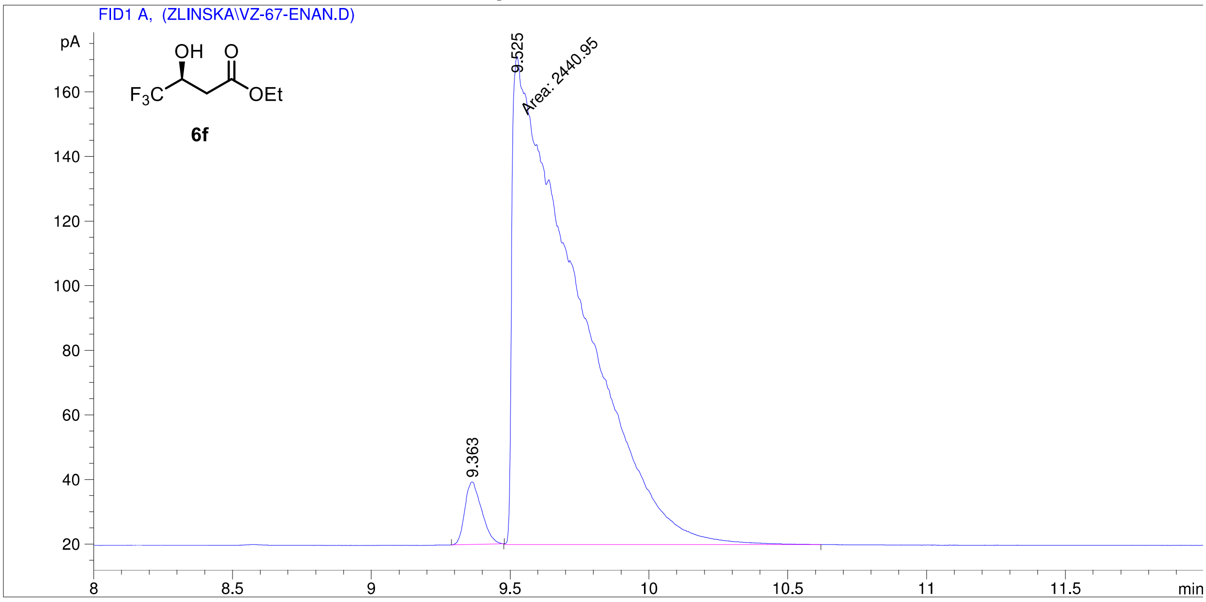

Peak	Retention Time (min)	Area (mAU × min)	Height (mAU)	Relative Area (%)
1	9.363	81.095	19.356	3.215
2	9.525	2440.953	151.177	96.785
Total		2522.048	170.533	100

GC analyses of racemic and enantiomerically enriched 6g

BetaDex 120 column, 82 °C for 60 mins, then 200 °C for 3 mins, flow rate 40 cm/sec

Figure S72. GC chromatogram of (±)-**6g**

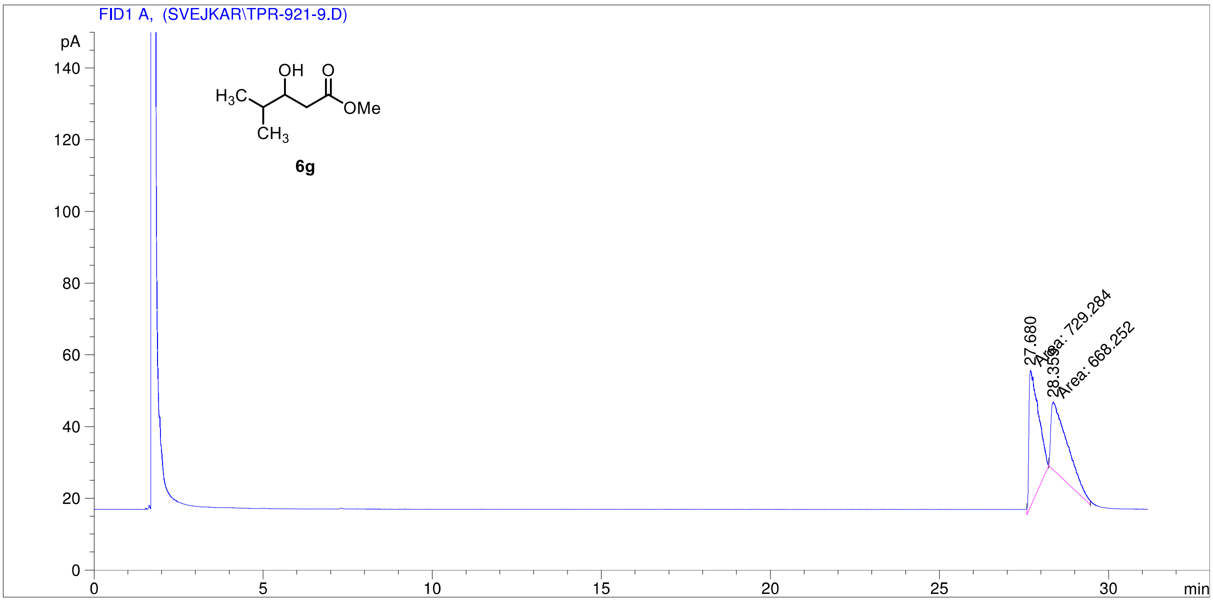

Peak	Retention Time (min)	Area (mAU × min)	Height (mAU)	Relative Area (%)
1	27.680	729.284	38.295	52.18
2	28.359	668.252	18.886	47.82
Total		1397.536	57.181	100

Figure S73. GC chromatogram of enantiomerically enriched **6g**

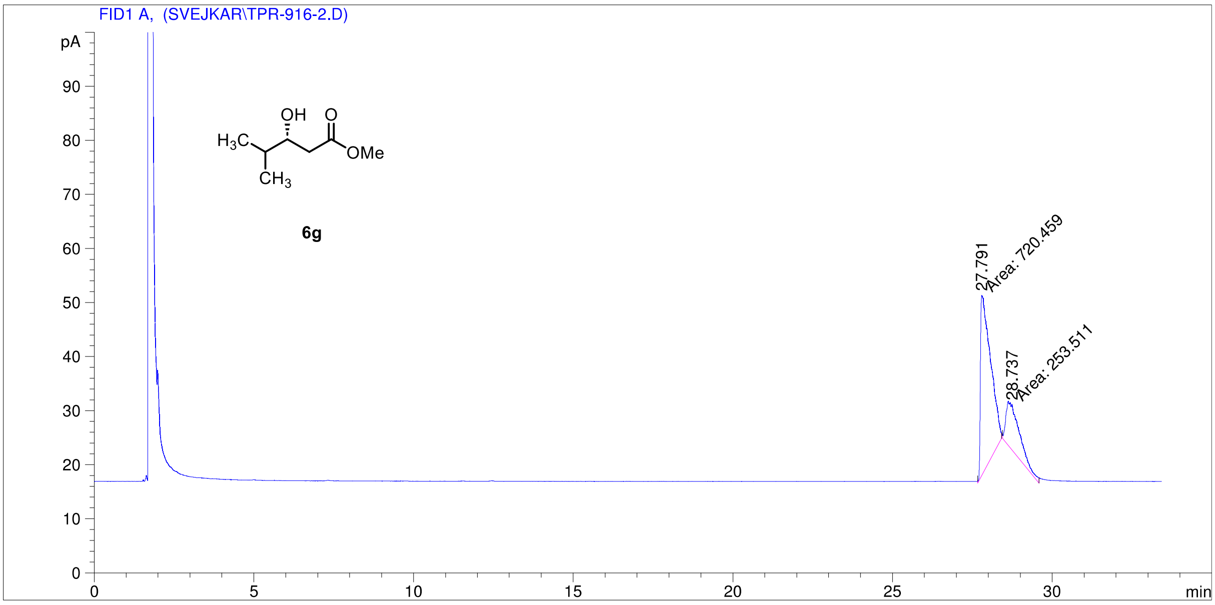

Peak	Retention Time (min)	Area (mAU × min)	Height (mAU)	Relative Area (%)
1	27.791	720.459	33.366	73.97
2	28.737	253.511	8.346	26.03
Total		973.970	41.712	100

HPLC analyses of racemic and enantiomerically enriched 24

Chiralpak IA column, 250 × 4.6 mm, λ = 254 nm, 1% isopropyl alcohol in hexane, flow rate: 1.0 mL/min

Figure S74. HPLC chromatogram of (±)-**24**

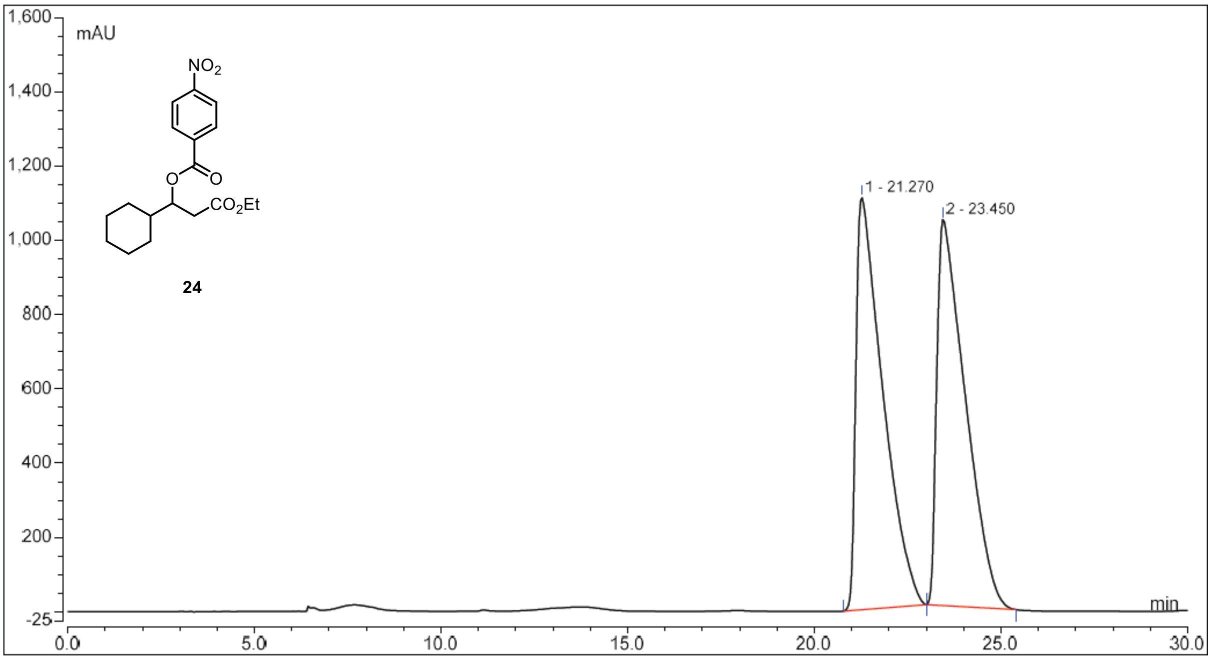

Peak	Retention Time (min)	Area (mAU × min)	Height (mAU)	Relative Area (%)
1	21.270	921.765	1110.421	50.14
2	23.450	916.770	1039.919	49.86
Total		1838.535	2150.340	100

Figure S75. HPLC chromatogram of enantiomerically enriched **24**

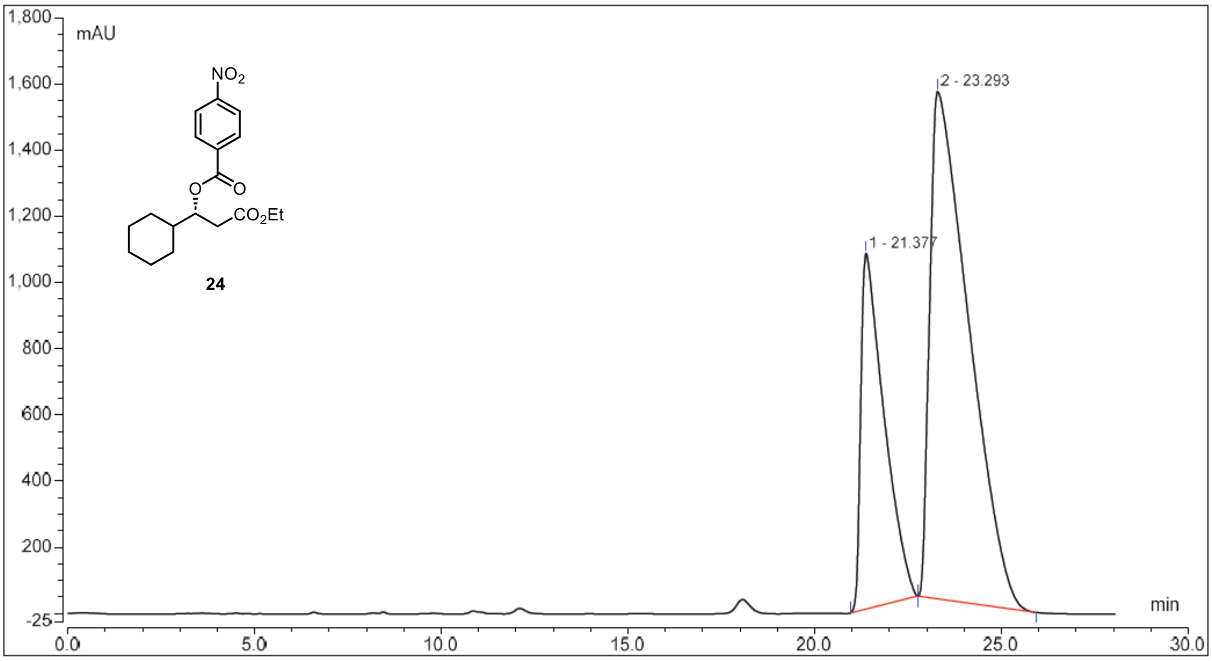

Peak	Retention Time (min)	Area (mAU × min)	Height (mAU)	Relative Area (%)
1	21.377	776.088	1074.916	29.52
2	23.293	1852.573	1533.912	70.48
Total		2628.661	2608.828	100

GC analyses of racemic and enantiomerically enriched 6i

BetaDex 120 column, 100 °C for 60 mins, then 200 °C for 3 min, flow rate 25 cm/sec

Figure S76. GC chromatogram of (±)-**6i**

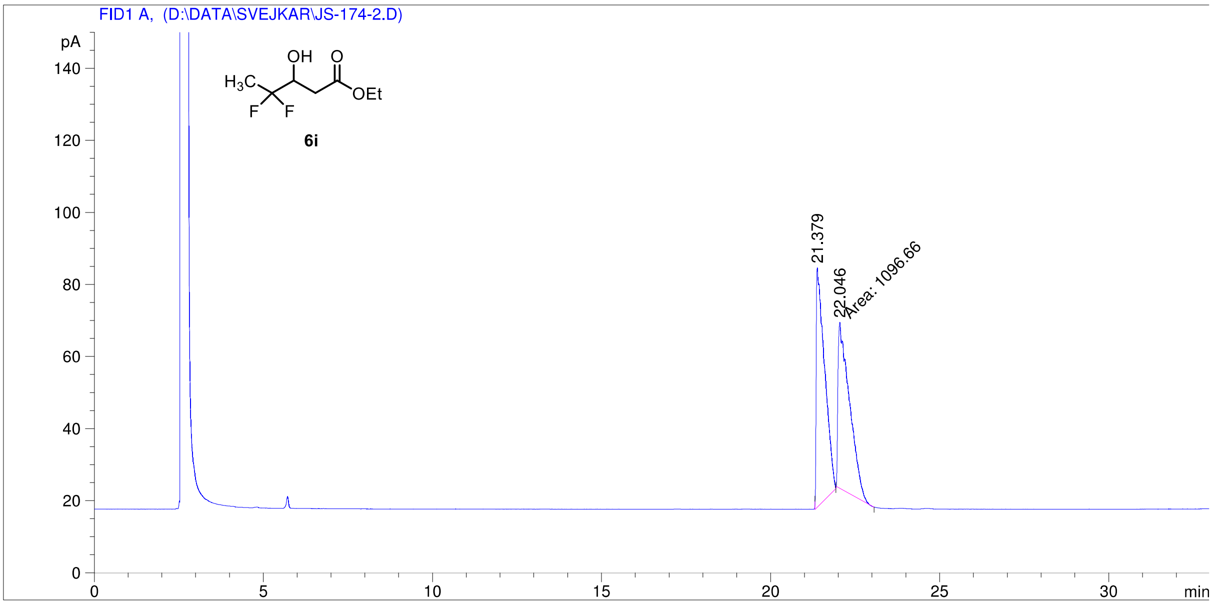

Peak	Retention Time (min)	Area (mAU × min)	Height (mAU)	Relative Area (%)
1	21.379	1146.531	66.464	51.112
2	22.046	1096.662	46.059	48.888
Total		2243.193	112.523	100

Figure S77. GC chromatogram of enantiomerically enriched **6i**

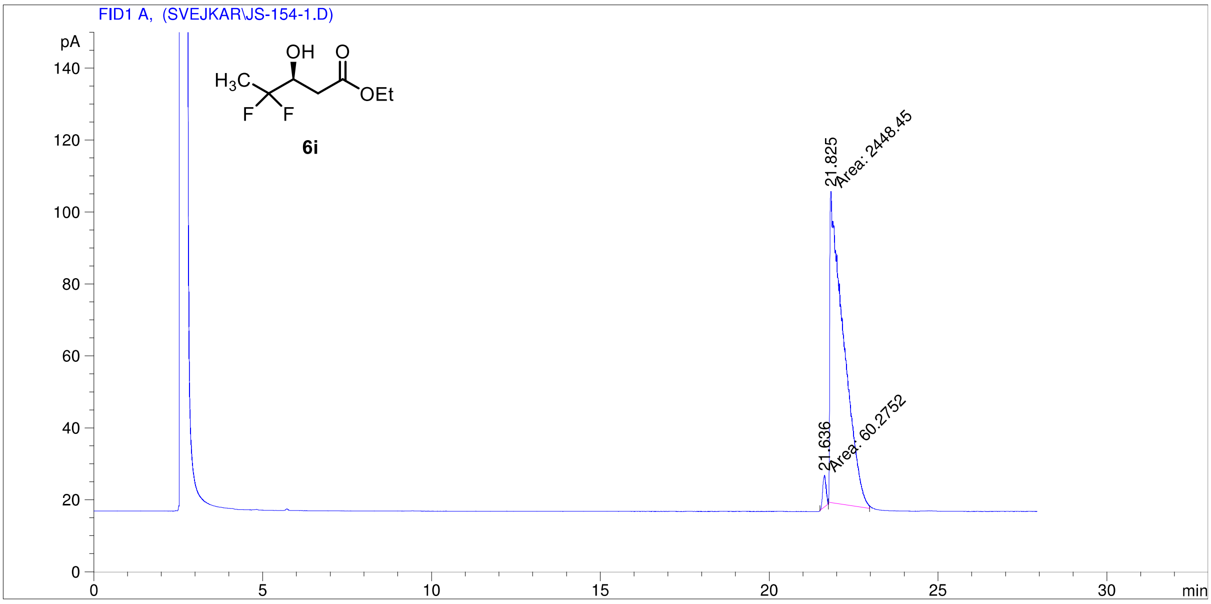

Peak	Retention Time (min)	Area (mAU × min)	Height (mAU)	Relative Area (%)
1	21.636	60.275	8.818	2.40
2	21.825	2448.446	86.555	97.60
Total		2508.721	95.373	100

HPLC analyses of racemic and enantiomerically enriched 6k

Chiralpak IA column, 250 × 4.6 mm, λ = 254 nm, 4% ethanol in hexane, flow rate: 1.0 mL/min

Figure S78. HPLC chromatogram of (±)-**6k**

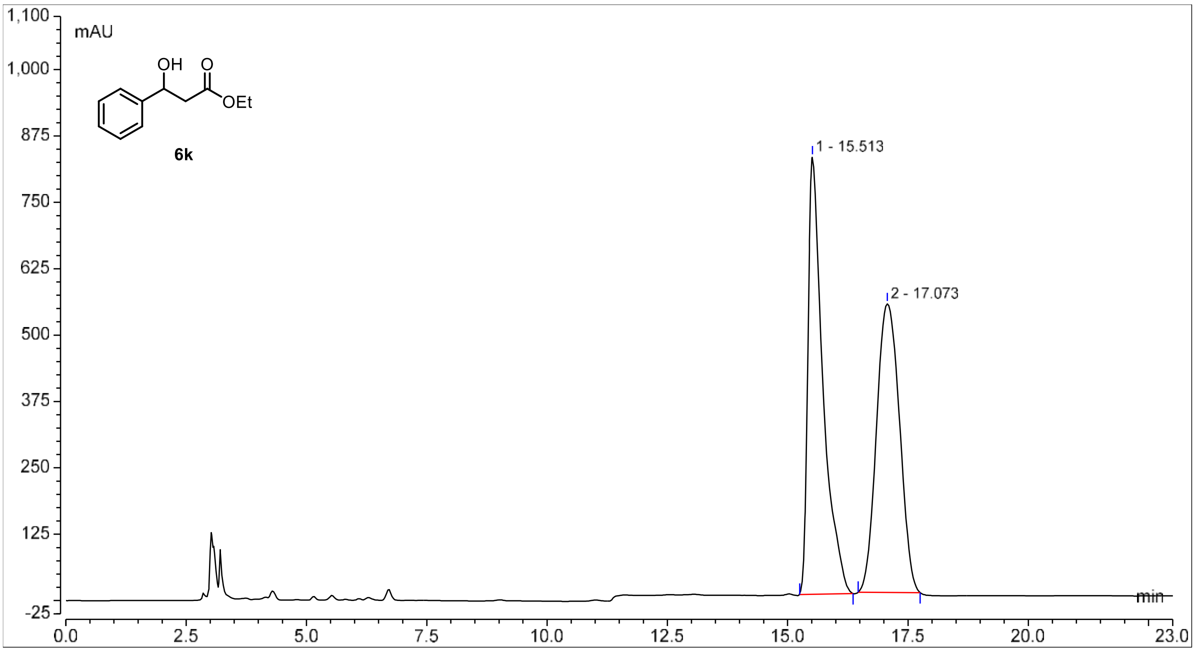

Peak	Retention Time (min)	Area (mAU × min)	Height (mAU)	Relative Area (%)
1	15.513	298.929	823.363	49.82
2	17.073	301.094	543.378	50.18
Total		600.023	1366.741	100.00

Figure S79. HPLC chromatogram of enantiomerically enriched **6k**

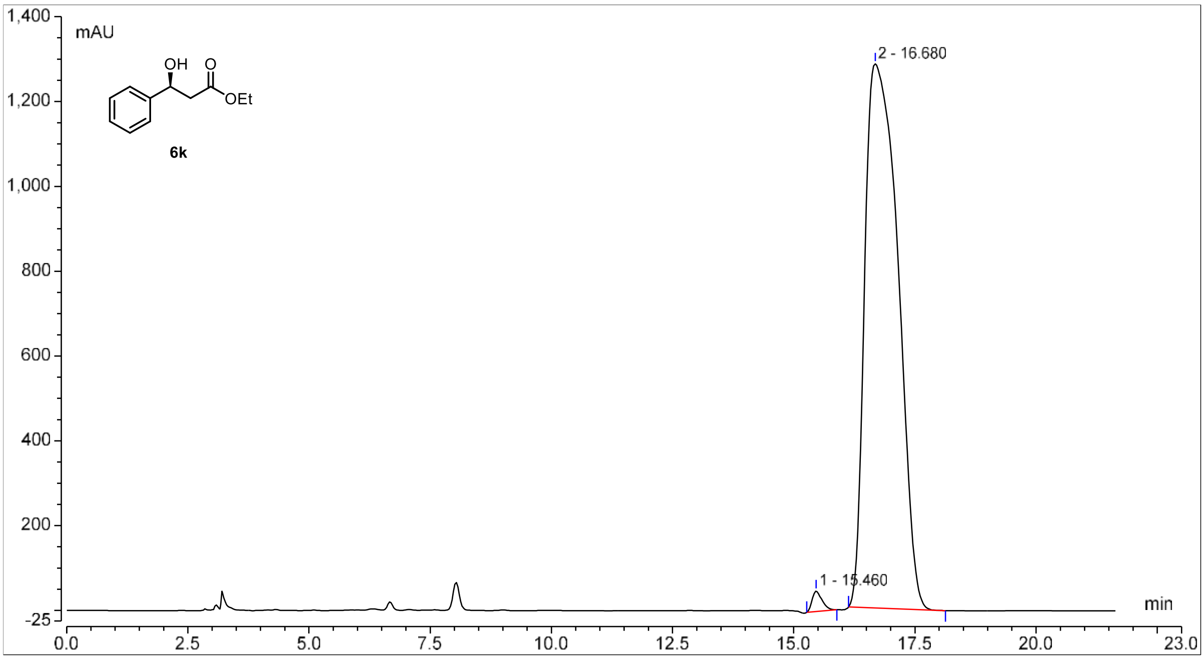

Peak	Retention Time (min)	Area (mAU × min)	Height (mAU)	Relative Area (%)
1	15.460	11.599	48.543	1.13
2	16.680	1011.630	1282.724	98.87
Total		1023.229	1331.267	100.00

HPLC analyses of racemic and enantiomerically enriched 6l

Chiralpak OJ column, 250 × 4.6 mm, λ = 254 nm, 2% isopropyl alcohol in hexane, flow rate: 1.0 mL/min

Figure S80. HPLC chromatogram of (±)-**6l**

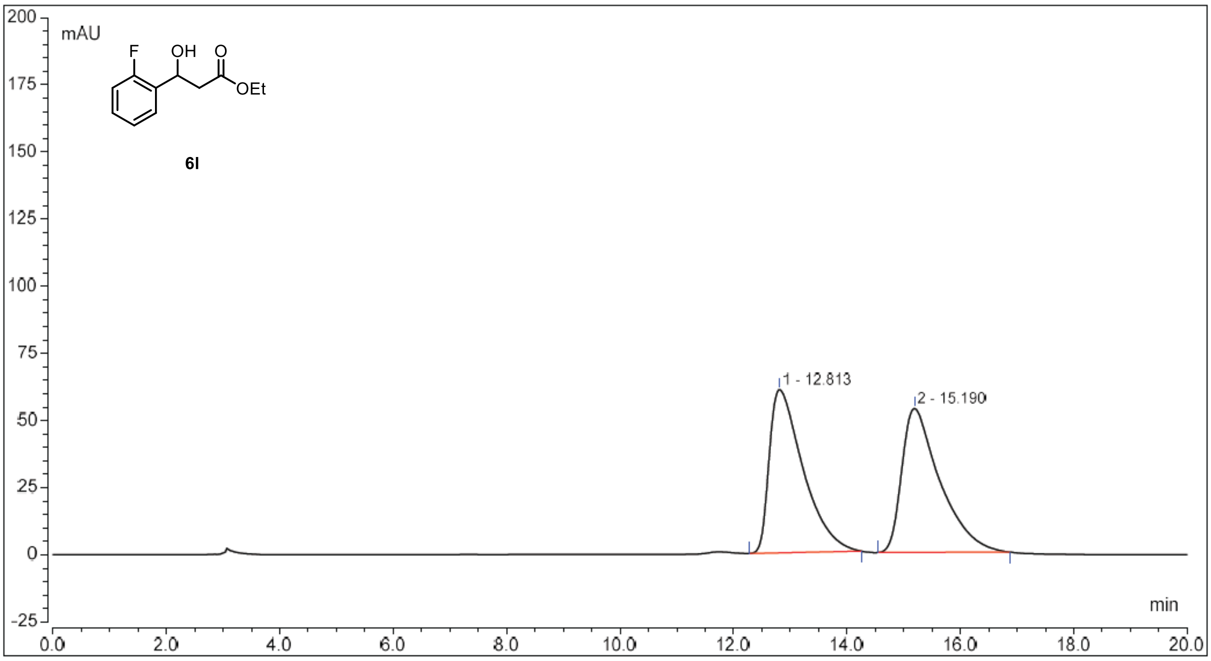

Peak	Retention Time (min)	Area (mAU × min)	Height (mAU)	Relative Area (%)
1	12.813	41.930	60.757	50.11
2	15.190	41.753	53.613	49.89
Total		83.683	114.370	100

Figure S81. HPLC chromatogram of enantiomerically enriched **6l**

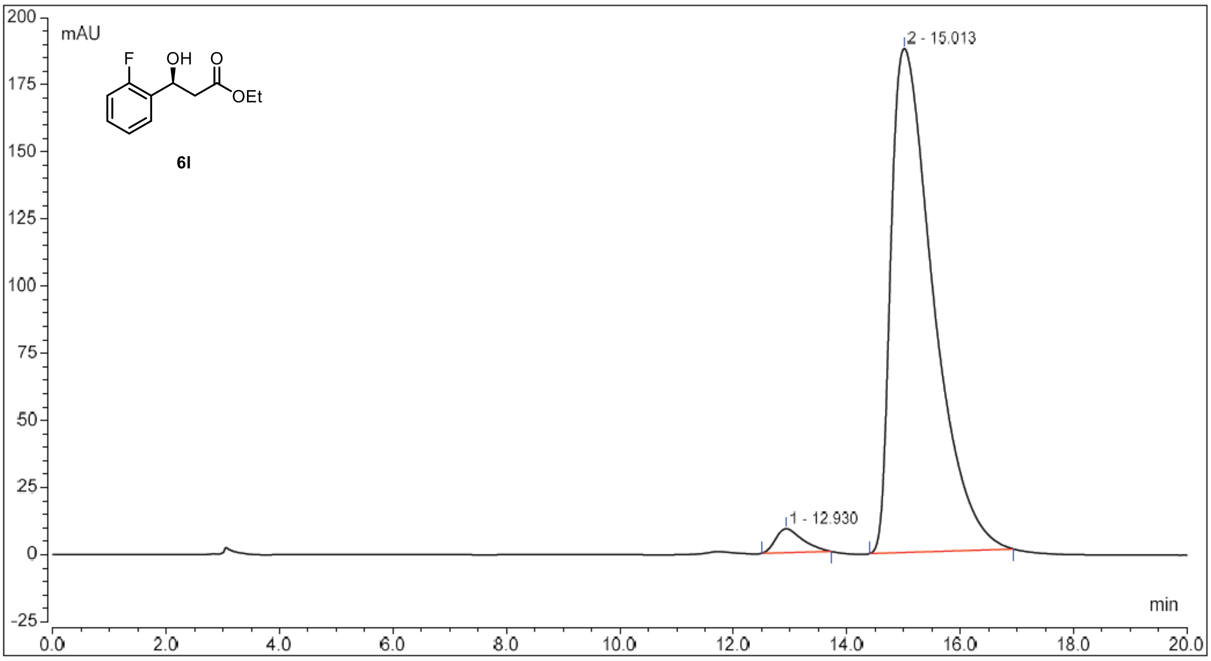

Peak	Retention Time (min)	Area (mAU × min)	Height (mAU)	Relative Area (%)
1	12.930	4.917	8.905	2.95
2	15.013	161.851	187.809	97.05
Total		166.768	196.714	100

HPLC analyses of racemic and enantiomerically enriched 6m

Chiralpak OJ column, 250 × 4.6 mm, λ = 254 nm, 2% isopropyl alcohol in hexane, flow rate: 1.0 mL/min

Figure S82. HPLC chromatogram of (±)-**6m**

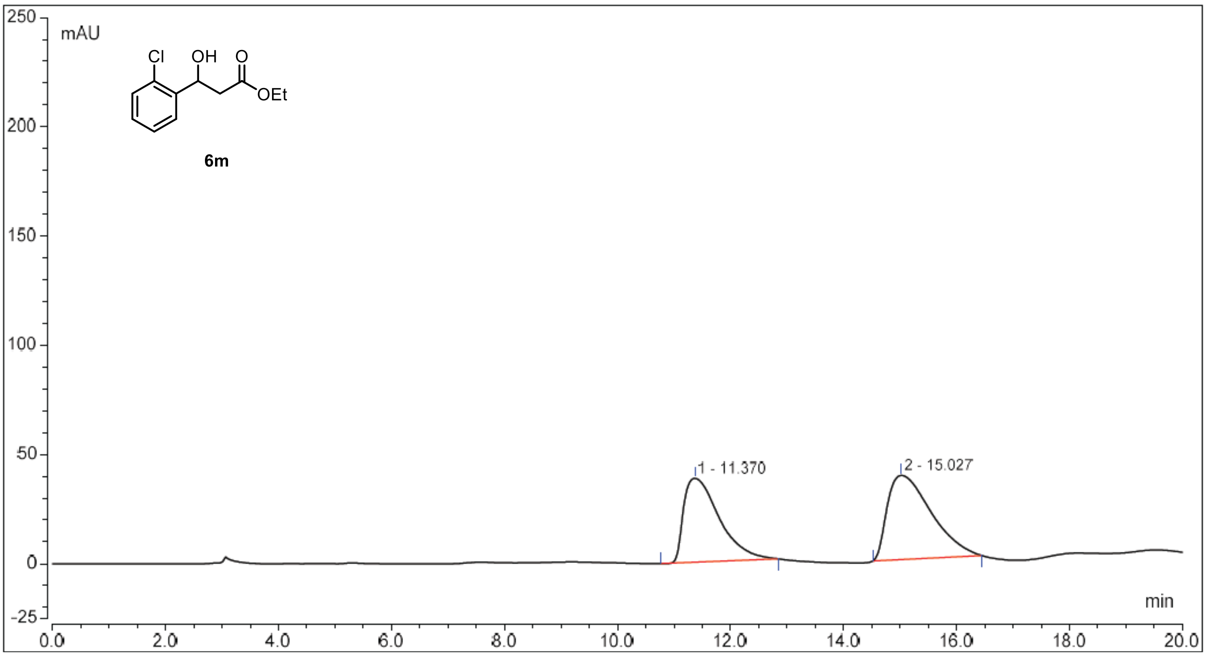

Peak	Retention Time (min)	Area (mAU × min)	Height (mAU)	Relative Area (%)
1	11.370	29.026	38.408	49.56
2	15.027	31.701	38.548	49.44
Total		60.727	76.956	100

Figure S83. HPLC chromatogram of enantiomerically enriched **6m**

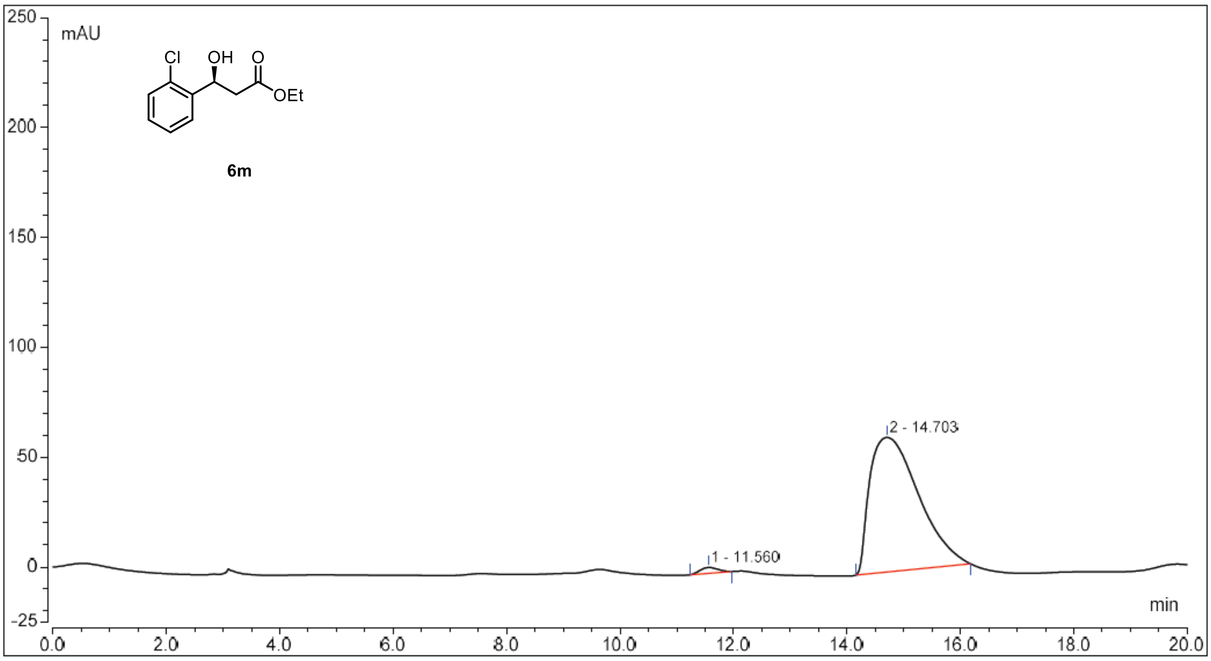

Peak	Retention Time (min)	Area (mAU × min)	Height (mAU)	Relative Area (%)
1	11.560	0.959	2.641	1.55
2	14.703	61.052	61.243	98.45
Total		62.011	63.884	100

HPLC analyses of racemic and enantiomerically enriched 6o

Chiralpak IC column, 250 × 4.6 mm, λ = 225 nm, 3% 2-propanol in hexane, flow rate: 1.0 mL/min

Figure S84. HPLC chromatogram of (±)-**6o**

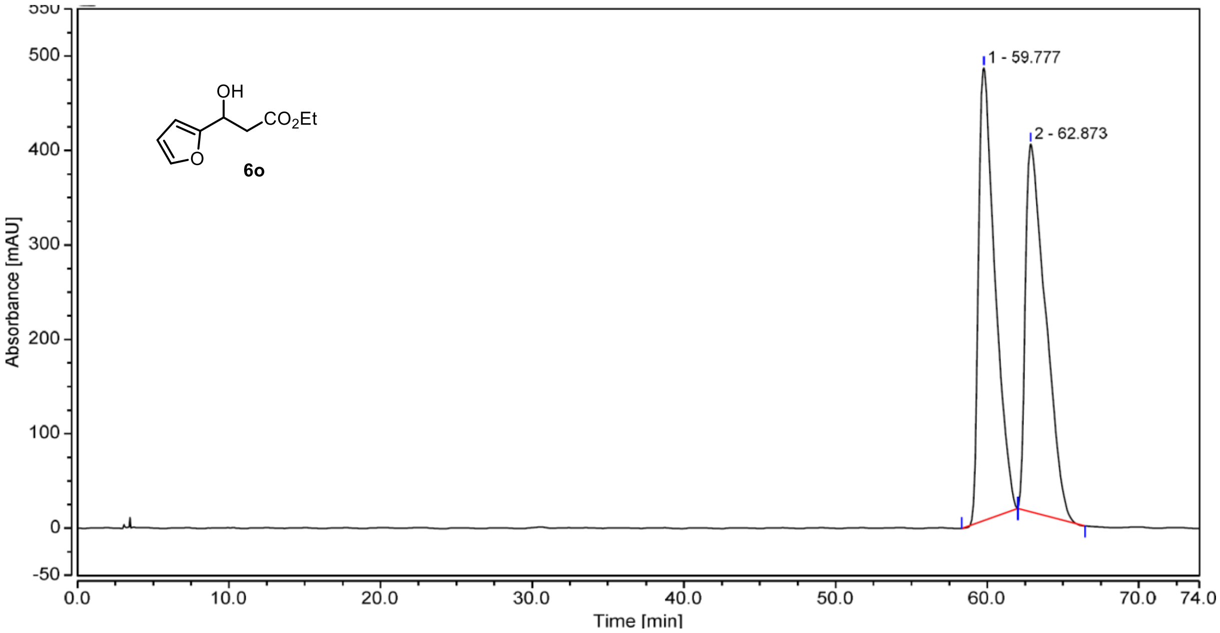

Peak	Retention Time (min)	Area (mAU × min)	Height (mAU)	Relative Area (%)
1	59.777	605.423	479.378	50.22
2	62.873	600.007	389.455	49.78
Total		1205.430	868.833	100.00

Figure S85. HPLC chromatogram of enantiomerically enriched **6o**

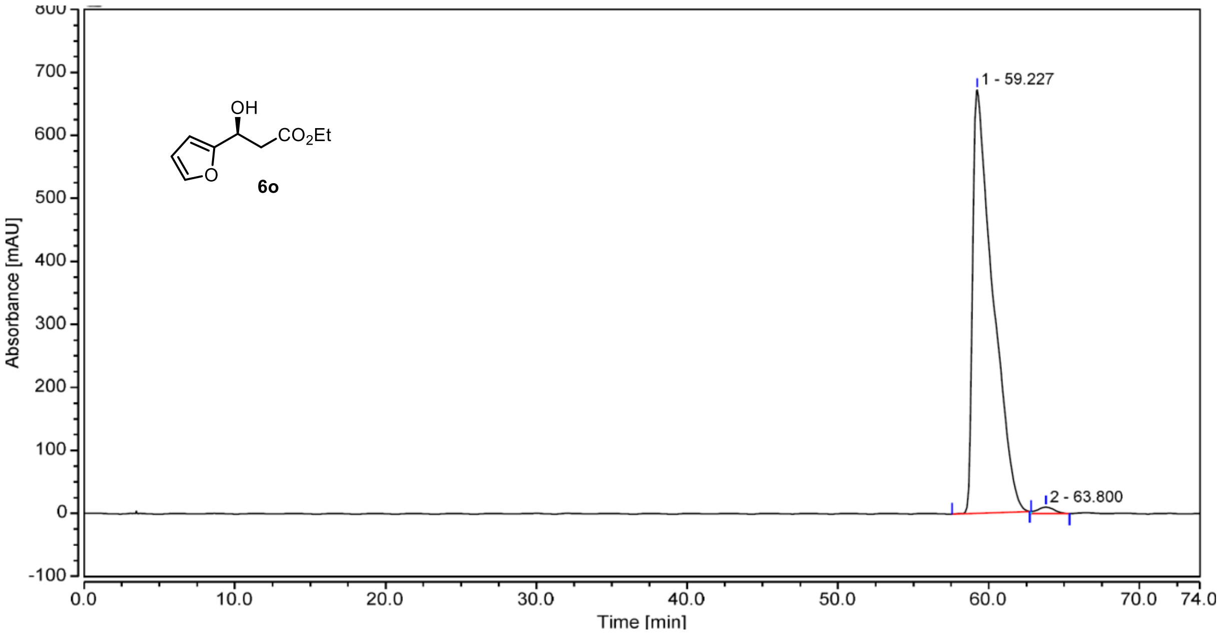

Peak	Retention Time (min)	Area (mAU × min)	Height (mAU)	Relative Area (%)
1	59.227	1037.240	672.104	98.75
2	63.800	13.166	10.213	1.25
Total		1050.406	682.317	100.00

HPLC analyses of racemic and enantiomerically enriched 6p

Chiralpak IC column, 250 × 4.6 mm, CAD, 7% 2-propanol in hexane, flow rate: 1.0 mL/min

Figure S86. HPLC chromatogram of (±)-**6p**

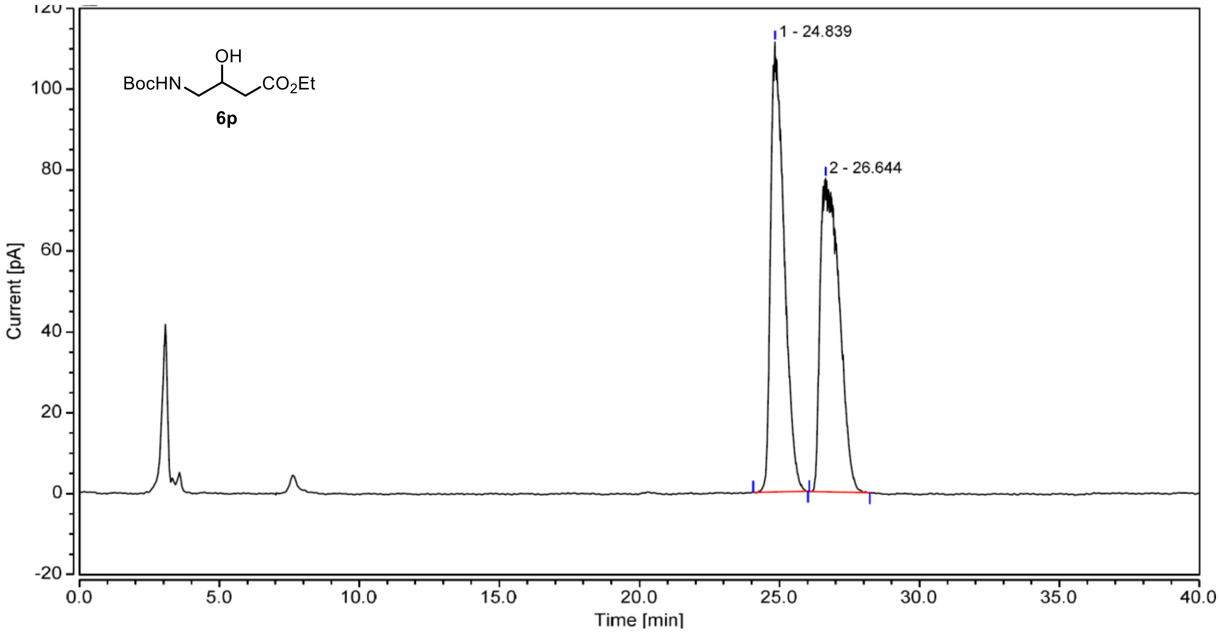

Peak	Retention Time (min)	Area (mAU × min)	Height (mAU)	Relative Area (%)
1	24.839	61.453	111.265	50.14
2	26.644	61.101	77.567	49.86
Total		122.554	188.832	100.00

Figure S87. HPLC chromatogram of enantiomerically enriched **6p**

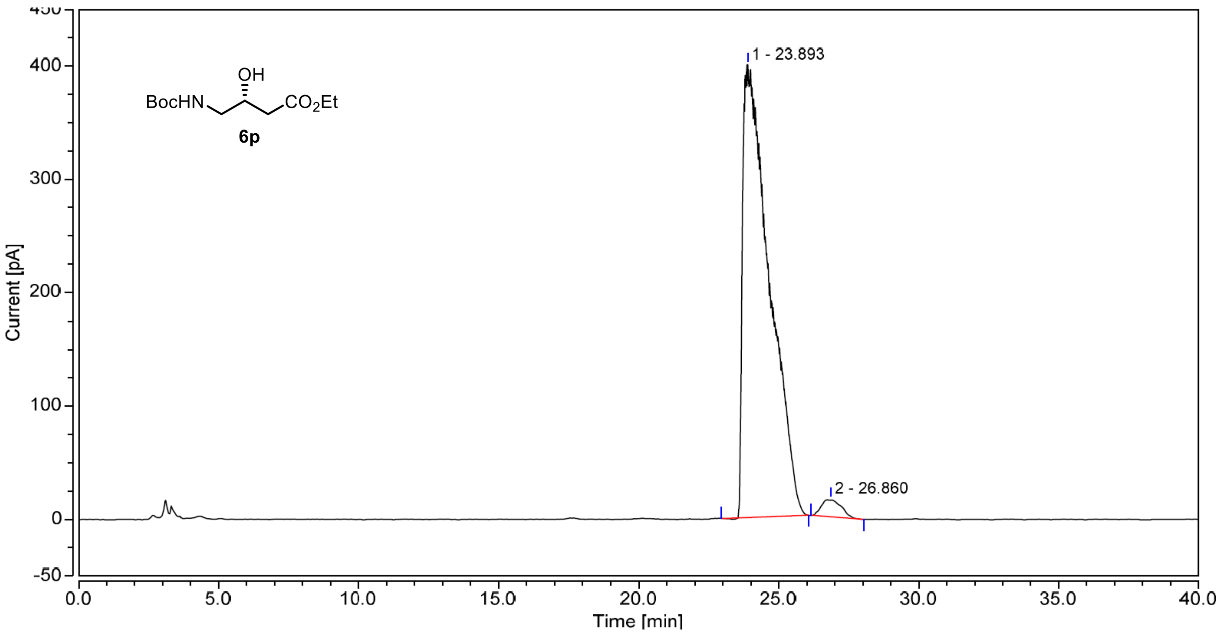

Peak	Retention Time (min)	Area (mAU × min)	Height (mAU)	Relative Area (%)
1	23.893	439.982	399.890	97.23
2	26.860	12.531	15.945	2.77
Total		452.513	415.835	100.00

HPLC analyses of racemic and enantiomerically enriched 6q

Chiralpak IC column, 250 × 4.6 mm, λ = 225 nm, 10% 2-propanol in hexane, flow rate: 1.0 mL/min

Figure S88. HPLC chromatogram of (±)-**6q**

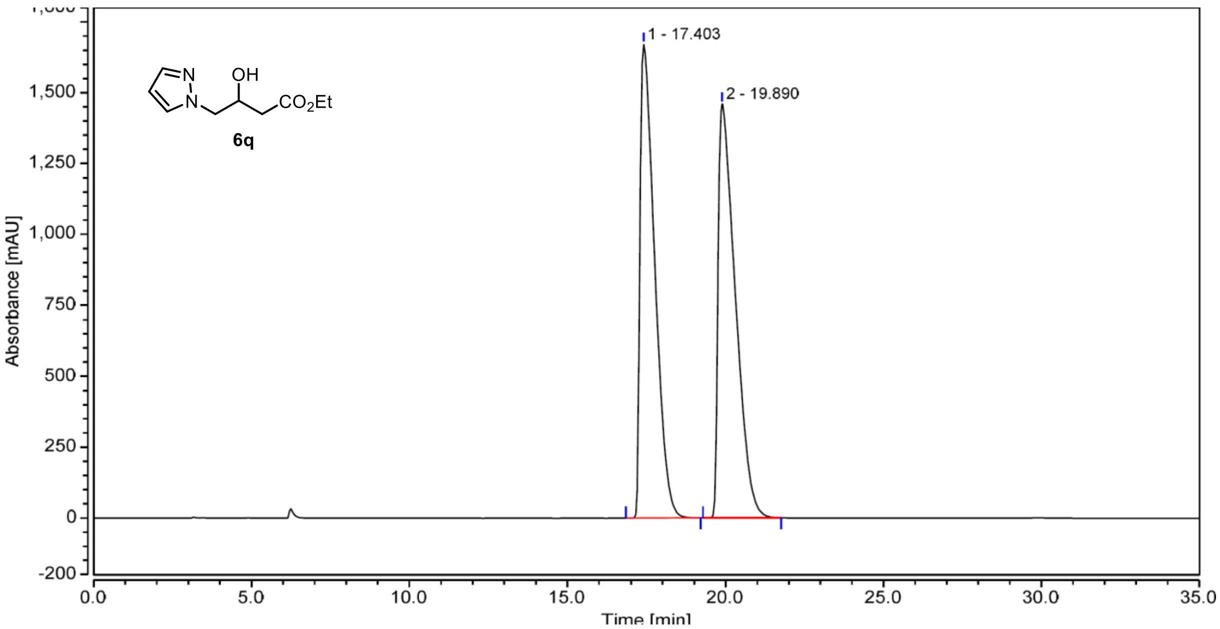

Peak	Retention Time (min)	Area (mAU × min)	Height (mAU)	Relative Area (%)
1	17.403	902.374	1671.827	49.44
2	19.890	922.744	1460.504	50.56
Total		1825.119	3132.331	100.00

Figure S89. HPLC chromatogram of enantiomerically enriched **6q**

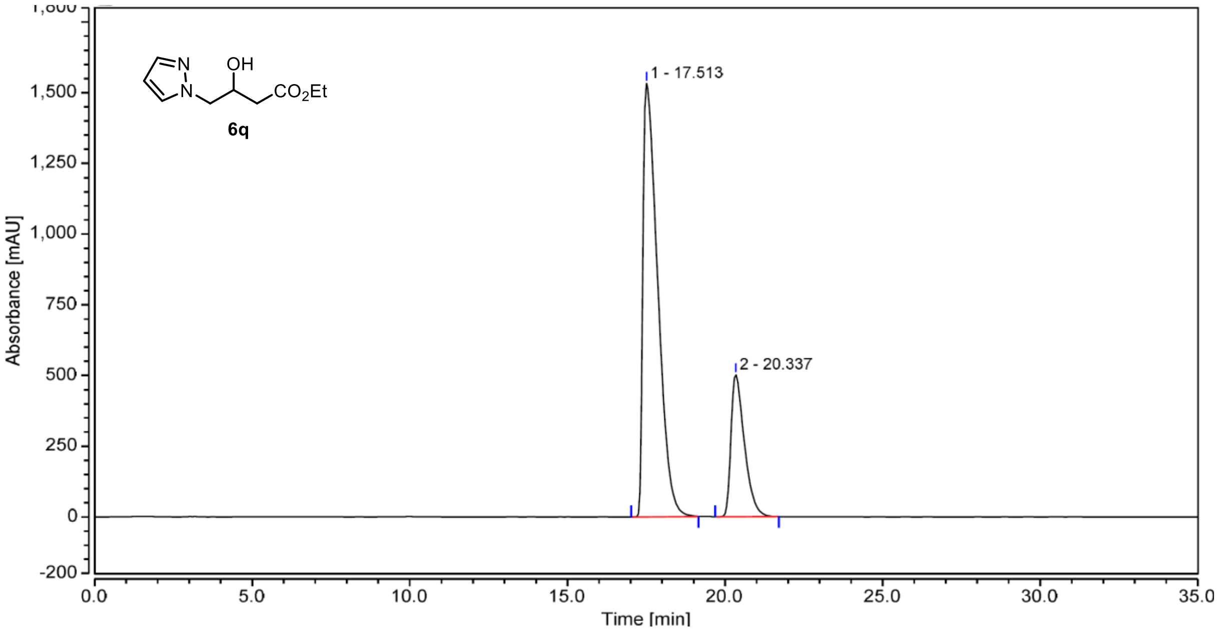

Peak	Retention Time (min)	Area (mAU × min)	Height (mAU)	Relative Area (%)
1	17.513	835.632	1532.470	77.07
2	20.337	248.582	502.412	22.93
Total		1084.214	2034.882	100.00

HPLC analyses of racemic and enantiomerically enriched 6r

Chiralpak IC column, 250 × 4.6 mm, λ = 225 nm, 3% 2-propanol in hexane, flow rate: 1.0 mL/min

Figure S90. HPLC chromatogram of (±)-**6r**

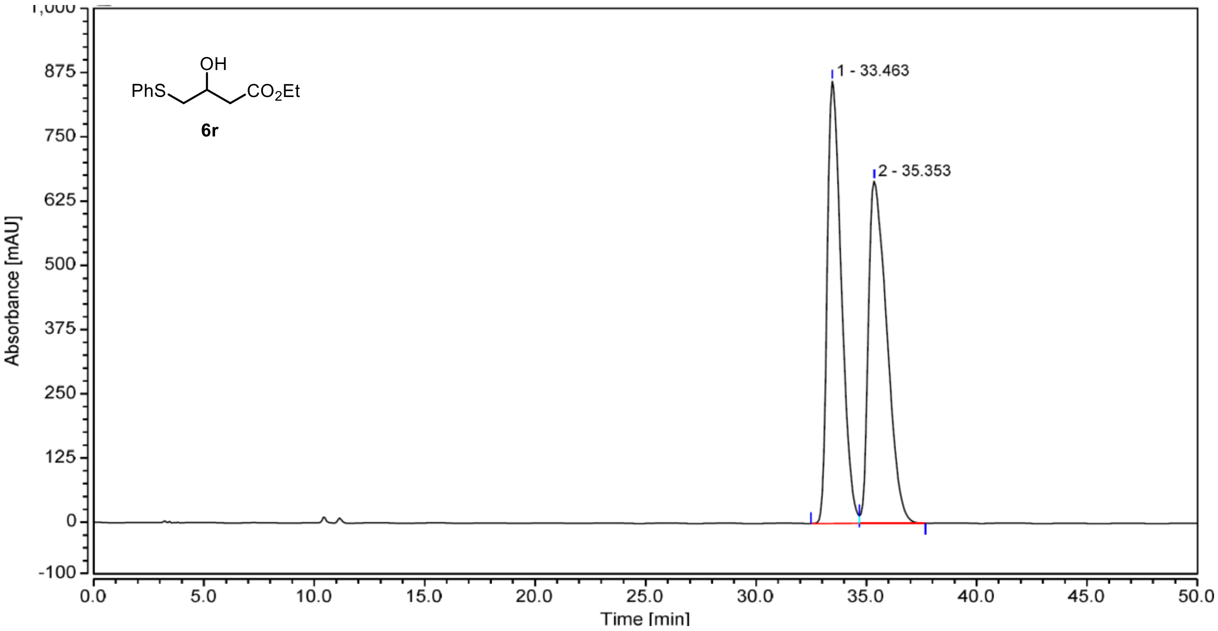

Peak	Retention Time (min)	Area (mAU × min)	Height (mAU)	Relative Area (%)
1	33.463	640.535	860.580	49.82
2	35.353	645.188	666.321	50.18
Total		1285.723	1526.902	100.00

Figure S91. HPLC chromatogram of enantiomerically enriched **6r**

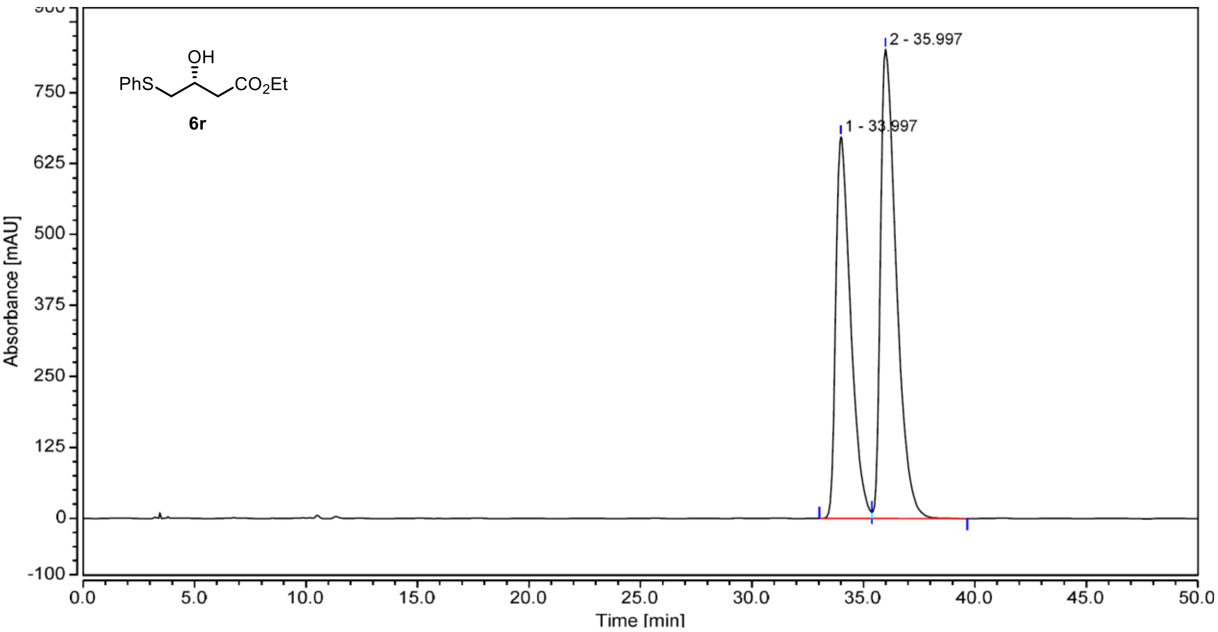

Peak	Retention Time (min)	Area (mAU × min)	Height (mAU)	Relative Area (%)
1	33.997	519.794	672.916	43.16
2	35.997	684.426	827.063	56.84
Total		1204.220	1499.978	100.00
